# Experience with Subgam, a Subcutaneously Administered Human Normal Immunoglobulin (ClinicalTrials.gov - NCT02247141)

**DOI:** 10.1371/journal.pone.0131565

**Published:** 2015-07-29

**Authors:** Clive Dash, Ernie Gascoigne, Kate Gillanders, Hock Gooi

**Affiliations:** 1 CD Consultants, St Albans, United Kingdom; 2 Medical Department, Bio Products Laboratory Limited (BPL), Elstree, Hertfordshire (Herts.), United Kingdom; 3 Department of Clinical Immunology and Allergy, King's College Hospital, London, United Kingdom; Imperial College, London, UNITED KINGDOM

## Abstract

**Background and Objectives:**

A multi-centre, non-comparative study examining the efficacy and safety of Subgam, a normal immunoglobulin (IgG) given weekly as a rapid subcutaneous infusion to patients with primary immune deficiency (PID), is reported. Also included is a summary of adverse drug reactions associated with the use of marketed Subgam in the UK.

**Materials and Methods:**

50 patients with stable PID on IgG therapy were enrolled: Stage 1 included three infusions with prior IgG product followed by 6 months with Subgam, Stage 2 involved long-term Subgam therapy up to 4 years.

**Results:**

Stage 1, 85% of the subjects aged >12 years and 93% of the subjects aged <12 years achieved IgG levels ≥6 and ≥4 g/L, respectively at all observations. There were 3.62 infections/patient/year during Subgam treatment. The most common product-related events were infusion site reactions (50% of patients). Recent post-hoc pharmacokinetics analysis of the post-infusion serum total IgG concentration indicated that the mean dose-normalised incremental IgG AUCτ following intravenous dosing (120.5 g.day/L) was 1.64-fold that of the dose-normalised mean incremental IgG AUCτ following subcutaneous dosing (73.6 g.day/L), corresponding to an estimated IgG bioavailability for subcutaneous dosing of 61%. Only 34 post-licensing adverse reactions have been received in 30 patients over a period of 10 years; fourteen were classed as serious as defined by the ICH guidelines on good clinical practice. The most common post-licensing adverse reaction was infusion site reaction (7 reports). There were 7 reports of flu-like symptoms (pyrexia/shivering/rigors/feeling hot or cold), 2 other reports of combined flu-like symptoms and infusion site reactions, 5 reports of generalised skin reactions, and 3 reports of combined infusion site and skin reactions. There were also reports of anaphylaxis (2 reports) and 8 other adverse events (including headache). In conclusion, Subgam is effective and well tolerated in the treatment of PID.

**Trial Registration:**

ClinicalTrials.gov NCT02247141

## Introduction

Patients who are diagnosed with primary immune deficiency (PID) require regular immunoglobulin replacement therapy in order to prevent or reduce the severity/frequency of infection and complications [1]. The efficacy of life-long intravenous immunoglobulin (IVIG) therapy is well established and is a major contributor to improved health and quality of life for these patients [2]. Although home-infusion for IVIG treatment is available, IVIG is more commonly given in a hospital setting where the infusion can take several hours to administer depending on the dose and tolerance to treatment. Since then, subcutaneous (SCIG) infusion (20 mL/hour) has been shown not only to be effective in PID but also to be well tolerated, to be very suitable for self-infusion at home, to improve quality of life, to give a high degree of patient satisfaction and also to reduce costs [3–7].

In this paper, we describe clinical experience with Subgam, a 16% human normal immunoglobulin product, before and after marketing, in the management of patients with PID. The data consists of the results of a study (SCIG01) on its efficacy and tolerability when given subcutaneously by syringe driver every week, and also post-licensing clinical experience.

## Methods and Materials

The protocol for this trial and supporting TREND checklist are available as supporting information; see S1 TREND Checklist and S1 Protocol. Registration of clinical trials was not required at the time the study was active, therefore when this became a requirement the data was entered onto the clinicaltrials.gov database retrospectively (NCT02247141). The authors confirm that all ongoing and related trials for this drug are registered. The study was modelled on the existing guidelines for clinical development of IVIG, because European Medicines Agency (EMA) guidelines on the clinical development of subcutaneous IgG were not available at the time [8].

### Ethical statement

The study was carried out in accordance with the International Conference on Harmonisation (ICH) Guideline for Good Clinical Practice [9] and the Declaration of Helsinki (South Africa, 1996).

### Regulatory and ethical approvals

The study protocol was reviewed and approved by the UK Medicines Control Agency (now known as the Medicines and Healthcare products Regulatory Agency, or MHRA). In addition, ethical approval was obtained for the protocol, consent forms and any other material issued to the patient from the UK Multicentre Research Ethics Committee (MREC) and local Research Ethics Committees (LRECs).

Ethical approval was obtained from the following institutions:

Northern & Yorkshire MREC (Dr Gooi, being the lead investigator, approval granted 11 November 1999); London—Surrey Borders Research Ethics Committee, formerly Merton & Sutton LREC (Dr Bansal, LREC approval 16 June 2000); Central Oxford Research Ethics Committee (Dr Chapel, LREC approval 15 January 2001); South Birmingham LREC (Dr Darbyshire, LREC approval 15 June 2000); Leicestershire Research Ethics Committee (Dr Duddridge, LREC approval 16 May 2001); Northern Sheffield Research Ethics Committee (Dr Egner, LREC approval 8 May 2000); Huntingdon LREC (Dr Exley, LREC approval 23 August 2000); Leeds (East Research ethics Committee, formerly Leeds Health Authority, St. James’s and Seacroft University Hospitals Clinical Research Ethics Committee (Dr Gooi, LREC approval 8 February 2000); Salford & Trafford LREC (Dr Haeney, LREC approval 10 May 2000); East London & The City Health Authority Research Ethics (Dr Longhurst, LREC approval 22 June 2000); The Great Ormond Street Hospital for Children NHS Trust/Institute of Child Health LREC (Dr Jones, LREC approval 5 September 2001); Dudley LREC (Dr Tsakona, LREC approval 22 March 2000); Preston, Chorley & South Ribble LREC (Dr Vijayadurai, LREC approval 6 December 2000); Southampton & South West Hants LREC (Dr Warner, LREC approval 14 August 2000); South East Wales Research Ethics Committee, formerly Bro Taf Health Authority LREC (Dr Williams, LREC approval 19 June 2000).

All patients gave written informed consent to participate. For patients under 18 years of age, written informed consent was obtained from the patient’s parent or legal representative.

### Study design

The open, non-comparative study was conducted at 14 centres in the UK.

At the time of preparing the study design there were no guidelines for the evaluation of subcutaneous immunoglobulins (SCIG). The sample size was based on the CPMP (Committee for Proprietary Medicinal Products, now CHMP, Committee for Human Medicinal Products) guidelines for the evaluation of IVIG. The protocol was subsequently modified to ensure that sufficient numbers of children were enrolled according to the subsequent CPMP guidelines for subcutaneous IgG.

#### Patient selection

Recruitment of patients into the study for each of the sites was initiated after obtaining LREC approval. Patients were initially approached by an investigator. The first patient was enrolled into the study on 14 June 2000 and last patient last visit (including follow-up period) was conducted on 12 January 2005. In accordance with the 1997 WHO Primary Immunodeficiency Diseases report [10], patients had to have had a diagnosis of PID with stable disease and been receiving immunoglobulin (IVIG or SCIG) therapy for at least 6 months. There were no age restrictions.

Grounds for exclusion included intolerance to IgA (to minimise the risk of a reaction to the small amount of IgA, typically <0.02% w/w, present in Subgam); pregnancy or breast-feeding; a history of clinically significant renal or hepatic disease or with known renal or hepatic abnormalities; history of infection within the last 2 months, requiring IV antibiotics; and known requirement for any other blood product during the course of the study.

The majority of protocol deviations were related laboratory samples not being taken at the correct time (263). Thirty-eight other deviations were related to visit/other procedures (excluding sample collection) not being conducted at the scheduled timepoints. Three deviations related to incorrect consenting procedures. The remaining deviations (33) were related to infusion schedules/dosing errors. All the deviations were logged and appropriate follow-up action was taken. None were deemed to have a significant effect on the outcome of the study.

### Treatment

Subgam was given subcutaneously at an initial weekly dose of 100 mg/kg bodyweight. Doses were then adjusted for each patient according to their clinical condition and to maintain a pre-infusion serum IgG level of at least 4 g/L (<12 years age group) or 6 g/L (≥12 years age groups). Subgam is produced by BPL.

The initial infusion rate in subjects ≥12 years old was 10 mL/h, increasing over the next few infusions to a recommended maximum of 20 mL/h. Two body sites could be infused simultaneously (via two syringe drivers) giving a combined maximum rate of 40 mL/h.

The initial infusion rate in the < 12 years age group was 5mL/h, increasing over the next few infusions to a recommended maximum of 20mL/h.

Stage 1 of the study comprised three infusions with prior IgG product (IVIG or SCIG) followed by 6 months with Subgam, typically given once weekly beginning one week after the last dose of the previous therapy. Initial infusions of Subgam were given in a hospital clinic, with patients switching to self-infusion at home after appropriate training and when assessed as safe to do so. Stage 2 involved home treatment with Subgam allowing long-term follow-up of patients under clinical trial conditions. After the launch of Subgam in the UK (2004), adverse drug reactions were reported to BPL by patients or healthcare professionals either directly or via the UK Medicines and Healthcare products Regulatory Authority (MHRA), using the yellow card reporting system.

In a separate study (previously unpublished data), 20 patients with PID were dosed with IVIG (Vigam liquid) with an actual dose range of 106 to 586 mg/kg per infusion.

### Assessments

Pre-infusion serum IgG levels were measured from samples taken weekly prior to the infusion administered at the hospital and 4-weekly prior to the infusion administered at home for the first 6 months of the study. Thereafter, serum IgG levels were measured approximately 3-monthly.

All infusion details, pre-infusion temperature, adverse events, if they felt unwell (including infections), concomitant treatments, days off school or work were recorded. During home therapy, the data were recorded in patient diary cards; in the case of younger patients this was conducted under supervision of the parents/guardians. No diagnostic tests for infection were conducted, consequently most infections were self-diagnosed by the patients/legal representative and site staff.

Patients/parents were asked to complete ‘treatment satisfaction’ questionnaires at pre-study (Questionnaire 1), Infusion 16 (Questionnaire 2) and at the End of Stage 1 (Questionnaire 3). In Questionnaire 1, patients categorised their current product as: (a) extremely comfortable; very comfortable; quite comfortable; not very comfortable; or extremely uncomfortable and (b) very convenient; quite convenient; neither convenient nor inconvenient; quite inconvenient; or very inconvenient;

In Questionnaires 2 and 3, patients categorised Subgam on the same scales and also indicated their preference for it (compared with their previous therapy) as: I like it much more; I like it more; I like it about the same; I don’t like it as much; or I don’t like it at all. Patients also assessed their symptoms as: much better; better; about the same; worse; or much worse (compared with previous therapy).

Patients who elected to continue in Stage 2 were asked to attend study visits at the clinic every 3 months for clinical follow-up when any changes since the last visit would be recorded.

### Analysis of data

The primary endpoint was the proportion of assessments at each time point where serum IgG was ≥ 4 g/L for the <12 years age group and ≥6 g/L for the ≥12 years age group.

Secondary efficacy endpoints included: the change in Subgam dose required to maintain target IgG levels; the mean change from baseline in serum IgG level and the time taken for each patient to reach a steady state serum IgG level, defined as the occurrence of three consecutive occasions when the IgG levels were within 1 g/L of each other.

Other efficacy endpoints were: the incidence of infections, days on antibiotics, days off work/school (if appropriate) and Patient Satisfaction Questionnaire responses. Patients recorded on their diary cards when they thought they had an infection, which was not always confirmed by their doctor but their ‘diagnoses’ were accepted for the analysis. No specific diagnostic tests were made to validate whether a suspected infection was a proven infection: thus, data on infections represent a worst-case analysis. Infections (and other adverse events) were classed as serious or non-serious in accordance with ICH definitions [9,11].

### Pharmacokinetic analysis (PK)

An optional pharmacokinetic assessment was performed in a group of patients who chose to take part. Blood samples were taken for serial measurement of serum IgG on two separate occasions (once during the week of the first infusion of Subgam and once after approximately 3–4 months’ treatment). On each of these occasions, a pre-dose blood sample was taken and then a sample on each of the following seven days (except weekends). Areas under the curve (AUCs) were determined by non-compartmental analysis using WinNonlin Enterprise (Version 6.2), Pharsight Corporation, USA. The linear trapezoidal rule was used to calculate AUC for this (SCIG01) study. IVIG AUCτ (AUC to the end of dosing period) was calculated from PID patients given liquid IVIG (Vigam liquid), previously unpublished data. For both Subgam and Vigam liquid, AUC was calculated using incremental changes in serum IgG using WinNonlin. Incremental values were calculated by subtracting the pre-infusion value from all post-infusion values for a given visit. Individual AUCs were normalised for individual dose, and the mean of these values was taken. Mean AUCs follow SCIG infusions were then compared with IVIG infusions.

### Post marketing data collection

The post-marketing ADR data was collated from spontaneous reports of incidents involving Subgam submitted to the regulatory authority (in the UK using the yellow card system) or directly to BPL from health care professionals and patients.

## Results

### Patients

**Fig 1:**
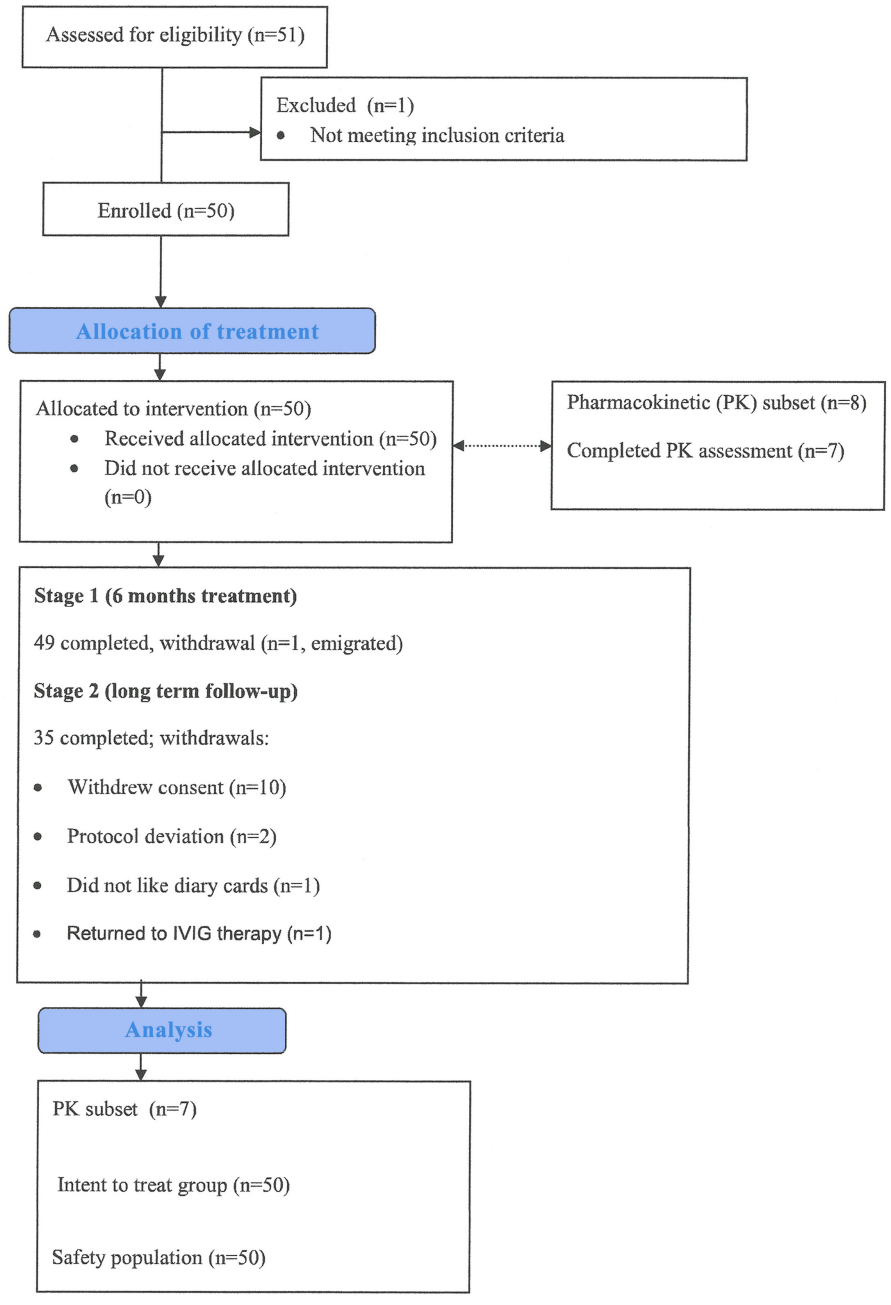
This is the flow diagram for patients enrolled in the SCIG01 clinical trial.

Fifty-one patients entered the study and were treated up to a maximum of 54 months treatment (Fig 1): One patient was withdrawn prior to receiving study medication and excluded from the analysis. The characteristics of the remaining 50 patients are shown in Table 1: there were 28 adults (≥20 years), 7 teenagers (≥12 and <20 years) and 15 children (<12 years). Subjects participated for a total of 51,655 study days; 45 patients (23 adults, 7 teenagers and 15 children) were in the study for at least a year, 44 (23 adults, 7 teenagers and 14 children) for at least 2 years, 32 (21 adults, 6 teenagers and 5 children) for at least 3 years and 8 (5 adults and 3teenagers) for at least 4 years. The most common diagnosis was common variable immunodeficiency (CVID). Fourteen patients had other SCIG therapy immediately prior to Subgam, and 36 received IVIG previously (Table 1).

**Table 1:** Patient characteristics.

Characteristic	All	Subjects ≥20 y Adults	Subjects ≥12 <20 y Teenagers	Subjects <12 y Children
	(n = 50)	(n = 28)	(n = 7)	(n = 15)
**Sex**				
Male/female N/N	25/25	10/18	3/4	12/3
%/%	50%/50%	36%/64%	43%/57%	80%/20%
**Age (years)**				
Mean	29.5	45.5	15.2	6.2
Min to max	0.8–75.2	21.3–75.2	12.1–18.0	0.8–10.6
**Weight (kg)**				
Mean	57.6	77.1	53.7	23.1
Min to max	10.1–132.7	46.0–132.7	37.2–70.1	10.1–48.2
**Diagnosis**				
CVID/XLA	33	20	6	7
CVID	30	20	6	4
XLA	3	0	0	3
Other diagnosis	17	8	1	8
IgG subclass deficiency	5	3	0	2
Specific antibody deficiency	7	4	1	2
CD40 ligand deficiency	2	0	0	2
IgG heavy chain deficiency	1	1	0	0
Ataxia telangiectasia	1	0	0	1
Combined immunodeficiency	1	0	0	1
**Prior therapy**				
SCIG	14	6	1	7
Home	10	4	1	5
Hospital	4	2	0	2
IVIG	36	22	6	8
Home	7	4	3	0
Hospital	29	18	3	8

CVID = common variable immunodeficiency

XLA = X-linked agammaglobulinaemia

n = number of patients

y = years

### Dose of Subgam

**Fig 2:**
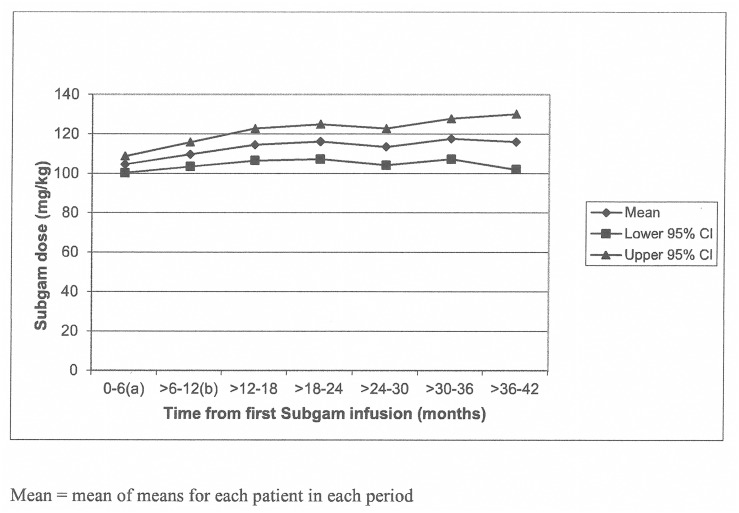
This is the graph showing Subgam dose per infusion (mg/kg) in 6-monthly intervals up to 42 months.

The majority of patients received a starting dose of or close to that recommended in the study protocol: 47 (94%) received 100mg/kg ± 20 mg/kg of which 36 (72%) received a dose of 100 mg/kg ± 5 mg/kg. However, three subjects (aged 15.9 to 22.5 years), all at the same centre, were started on doses of 32–42 mg/kg, although their doses were increased to that recommended by the third Subgam infusion. The dose of Subgam for subsequent infusions was adjusted according to clinical requirements, and increased slightly from a mean of 104.6 mg/kg per infusion during the first 6 months, to 115 mg/kg at 18–24 months of treatment (Fig 2).

Overall, the mean dose per infusion was 110.9 mg/kg and the mean number of infusions per patient was 146.7 (range: 22 to 317); this exposure was equivalent to 141.5 patient years.

Only one patient was consistently poorly compliant; this patient had also been poorly compliant on his prior IVIG therapy.

### IgG levels

During Stage 1, 29/34 (85%) of the ≥12 years age group and 14/15 (93%) of <12 years age group achieved target serum IgG levels at all observations (Table 2).

**Table 2:** Number of observations of serum IgG below the target.

Period of study	Subjects < 12 y				Subjects ≥ 12 y			
	Observations		Patients (N = 15)		Observations		Patients (N = 34)	
	n	IgG <4 g/L	n	IgG <4 g/L	n	IgG <6 g/L	n	IgG <6 g/L
*Pre-Subgam*	21	1	11	1	75	5	34	3
*Stage 1*, *months 0 to 6* ^a^	122	3	15	1	463	16	34	5
*Stage 2*, *months*								
>6 to 12^b^	18	0	13	0	61	5	30	3
>12 to 18	16	0	11	0	61	5	27	4
>18 to 24	16	0	10	0	64	8	27	4
>24 to 30	14	0	8	0	58	8	28	4
>30 to 36	16	0	10	0	46	2	26	2
>36 to 42	5	0	4	0	32	7	21	4
>42 to 48	2	0	2	0	22	3	16	3
>48 to 54	0	0	0	0	8	1	7	1
Total in Stage 2	87	0	13	0	352	39	30	6

^a^ Stage 1 lasted approximately 6 months, depending upon the number of infusions received by the patient and contains post-Subgam data only

^b^ This interval lasted approximately 6 months, from the first infusion in Stage 2 until 12 months after the first infusion of Subgam

**Fig 3:**
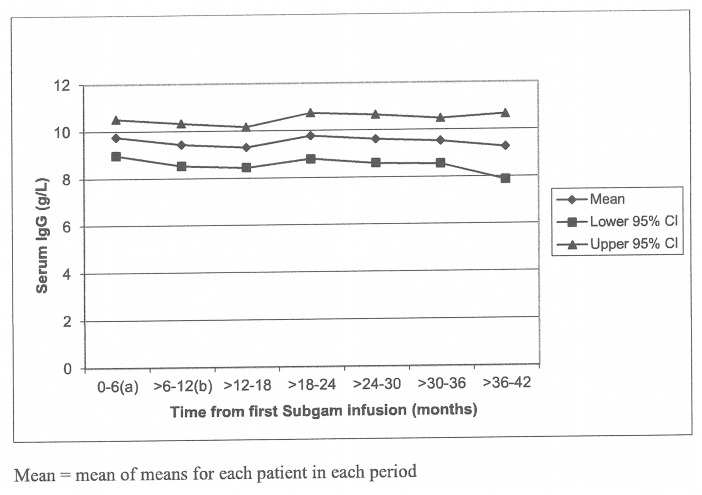
This is the graph showing Subgam IgG levels (g/L).

Mean serum IgG level increased from 9.2 g/L to 9.75 g/L by the end of Stage 1 with Subgam treatment and was maintained above the pre-Subgam level for 30 months of treatment (Fig 3). The overall mean serum IgG value was sustained for the 30–36 month period.

The overall mean serum IgG level in Stage 1 was slightly lower in patients with CVID/XLA than those with other PIDs (9.24 g/L [SD 2.10; n = 32] and 10.70 g/L [SD 3.42; n = 17], respectively). The difference was also apparent in the pre-Subgam phase of the study. However the difference in serum IgG between the groups was relatively small with a large overlap of standard deviations. Also, mean values for both groups were within laboratory normal ranges.

### Infections

A total of 513 infections were reported by the 50 patients: 329 (64%) by adults; 66 (13%) in teenagers and 118 (23%) by children/parents. Most infections were of the upper and lower respiratory tract (40.4% and 34.9% respectively); 20 were severe, with 19 reported by 10 subjects in the ≥20 years of age group. Out of the 513 infections, 22 were classified as serious and were experienced by 9 patients; these comprised of 17 lower respiratory infections, 1 gastrointestinal infection, 2 skin infections and 2 infections categorised as other. There was no difference in the incidence of non-serious infections between patients with CVID/XLA compared patients with Other diagnoses. Serious infections were more common in the Other group compared with CVID/XLA.

Among the 50 patients, there were 3.62 infections per patient per year during Subgam treatment; patients experienced 0.16 serious infections per patient per year during Subgam treatment.

### Antibiotic use

During the Subgam treatment phase, 49 of the 50 patients received antibiotics for a total of 40.3% of study days. Only 10 (7 with CVID or XLA) patients required intravenous antibiotics for 0.9% of all study days.

### Time off school/work

In total, 28 of the 33 applicable patients took at least one day off work/school. The median percentage of days taken off was 1.2%, and was similar for prior IVIG and prior SCIG patients.

### Patient questionnaires

Questionnaires were completed by 38 patients after 3 months and 43 after 6 months. Most patients liked Subgam more or much more than their previous medication (89% and 77% after 3 and 6 months, respectively), while symptoms were described as being better/much better on Subgam by 50% and 58% of patients, respectively.

Of the 36 patients who completed questionnaires 1 and 2, 56% gave Subgam a higher rating for convenience and 36% gave it a higher rating for comfort than their previous medication.

### Pharmacokinetic data (PK)

Eight subjects agreed to participate in the pharmacokinetic study, 7 of whom completed the repeat pharmacokinetic assessments after approximately 3 months’ Subgam treatment. Due to the number of daily serum samples which were not collected from subjects after repeat dosing, individual AUCτ values could not be generated directly. AUC values were generated in each subject for the period between the first and the last actual sample measured. Since the Coefficient of Variation of the intra-individual variability in serum IgG concentrations was <10% for all subjects, the AUC was then normalised to seven days and this value was assumed to be representative of the AUCτ. The actual doses given to the seven PK subjects were used to dose-normalise the AUCτ values. In the seven PK subjects there were six steady state trough serum IgG concentrations measured, four at the start of the dosing period and two at the end. The mean of these six values was 9.5 g/L. The mean incremental AUCτ value generated was 8.9 g.day/L. The mean dose given to those subjects whose repeat dose pharmacokinetics was determined was 121 mg/kg. Thus the mean incremental IgG AUCτ (8.9 g.day/L), corrected for mean dose given (121 mg/kg), provides a dose-normalised value of 73.6 g.day/L/(g/kg).

The mean dose normalised-incremental IgG AUCτ following intravenous dosing with Vigam liquid (120.5 g.day/L) was 1.64-fold that of the dose normalised mean incremental IgG AUCτ following subcutaneous dosing with Subgam (73.6 g.day/L).

### Safety and tolerability

In the clinical study on Subgam in PID, a total of 7334 infusions were administered in 50 patients. There were 84 infusion site events (including 2 local infections) in 25 patients (50%); these included: rash, swelling, inflammation, erythema, tenderness, pain, itchiness, bruising and bleeding. Of these 84 events, 76 in 21 patients (42%) were classed as related to Subgam. When expressed per infusion, the approximate rate for infusion site events (irrespective of cause) was 1 per 87 infusions. A further 68 adverse events (in 23 patients, 46%) were also described as related to Subgam: the most commonly reported symptoms were: headache (eight reports in seven patients, 14%), vomiting (five reports in three patients, 6%), pruritus (seven reports in two patients, 4%), and dizziness (three reports in two patients, 4%). The number of infusion site reactions was highest during Stage 1, with 59 of the 84 reactions (70%) occurring during this stage; thereafter the number of infusion site reactions fell and none were reported after 36 months in the study. None of the product-related adverse events was serious and no subject withdrew from the study because of adverse events.

There was no notable overall change in haematology or biochemistry parameters before and after participation in the study. There was no evidence of the transmission of HIV, Hepatitis B virus, Hepatitis C virus or Parvovirus B19.

### Post-licensing data

Subgam was launched in the UK in July 2004. From launch until the end of May 2014 sales of Subgam (commercial stock) amounted to 867.6 kg. Assuming an average infusion dose of 100 mg/kg per week, this would equate to approximately 115,700 infusions (for a 75 kg patient) or 433,800(for a 20kg child), equivalent to an exposure of 2,225 and 8,342 patient.years respectively. During the post-licensing period a total of 34 adverse drug reactions were reported in 30 patients. The patients were aged from 5 to 77 years (age was not given in four cases). Fourteen reactions were rated serious according to the ICH definition [9]: Four of the serious reactions required hospitalisations, one was considered life threatening (anaphylaxis), two resulted in persistent or significant disability/incapacity (rigors, infusion site dermatitis) and the remaining seven were classed as other important medical conditions. The doses at the time of the adverse drug reactions, where given, ranged from 17.1 to 195.7 mg/kg.

The most commonly reported adverse reaction from post-marketing reporting was infusion site irritation (7 reports). There were also 7 reports of flu-like symptoms (such as pyrexia, sweating, shivers, rigors, feeling hot or cold), 2 additional reports of combined flu-like symptoms and infusion site reactions, 5 reports of skin reactions, 3 reports of combined generalised skin and infusion site reaction, 8 reports of other adverse events (including headache) and 2 reports of anaphylaxis. Of the latter, one case involved a patient who had previously experienced anaphylaxis with an intravenous immunoglobulin and subsequently experienced a similar reaction to another subcutaneous immunoglobulin. The other case involved a patient with documented IgA deficiency. There was no association between the adverse reactions and particular batches; in total, 23 different batches were involved in post-marketing product-related events; no batch was associated with more than three reactions.

## Discussion

Replacement therapy with IVIG is now accepted practice for patients with PID [2]. However this mode of administration is not suitable for all patients for a range of reasons, including poor venous access, systemic adverse reactions and the need for frequent hospital visits. Our study found Subgam to be a suitable replacement therapy and an alternative to IVIG in PID patients and the 10 years since marketing has confirmed conclusion.

Target serum IgG levels were achieved at all observations in Stage 1 of the study in the great majority of patients: 85% of subjects ≥12 years of age and 93% of subjects <12 years of age. Mean IgG trough increased from 9.2 to 9.75 g/L by the end of Stage 1. However, the overall serum IgG level before Subgam and in Stage 1 was slightly lower in patients with CVID/XLA than those with other PIDs. The difference in serum IgG between the groups was relatively small with a large overlap of standard deviations. Also, mean values for both groups were in labaratory normal ranges. No evidence that marginally lower IgG levels in CVID group was associated with higher incidence of infections (non-serious) or infections (serious). Other studies also demonstrate maintenance of adequate serum IgG concentrations by using subcutaneous infusions [6,12–14]. Gardulf et al [14] showed increases in serum IgG levels from 7.8 to 9.2 g/L in adults at doses of 100mg/kg/week. Thépot et al [15] administered doses of 108mg/kg and observed serum IgG increases from 8.37 to 8.82 g/L. These are similar to the IgG increases seen with Subgam.

The mean dose normalised incremental IgG AUCτ following intravenous dosing was 1.64-fold that of the dose normalised mean incremental IgG AUCτ following Subgam dosing (73.6 g.day/L), which corresponds to an estimated subcutaneous IgG bioavailability of 61%. This is consistent with previous measurements of the bioavailability of subcutaneously dosed immunoglobulins. For example, mean subcutaneous bioavailabilities of 69.9% and 65.5% were calculated for 16% and 20% subcutaneous products respectively [16,17]. Similarly, for a 10% subcutaneously administered IgG preparation the bioavailability was 64.9% [18,19]. Another 10% IgG product had a quoted subcutaneous bioavailability of 69.3% [16,19].

As previously stated, no specific diagnostic tests were conducted in this study to confirm an infection documented by a patient and so the data on infections represent a worst-case. There was no marked increase in the frequency, severity or seriousness of bacterial infections during Subgam treatment. The number of infections during Subgam treatment (3.62 infections per patient per year) was similar to the rates reported by other workers during SCIG treatment [13,20] and similar to IVIG [13]. The great majority of infections were of the upper and lower respiratory tract, again in line with the experience of other workers [13,14,20], and only 22 of the 513 infections were classified as serious.

In total, all applicable patients took a median of 4.4 days per patient per year off work or school; this result is broadly comparable to that reported by Ochs and colleagues [16], in whose study 62.7% of patients missed days off school or work, with the annual rate being 3.7 missed days/patient.

In general, compliance was very good; only one patient was consistently poorly compliant. Good compliance may well have been related to the high degree of patient satisfaction with their treatment as assessed by the patient questionnaires. The great majority of infusions (93.6%) were given at home. Other workers have found a clear patient preference for home treatment over clinic therapy, and also for SCIG over IVIG, even in those patients already being treated at home [6]. A switch from hospital-based IVIG to home-based SCIG therapy has been shown to improve quality of life for both adults and children with PID [7].

During the study covering 141.5 patient.years, Subgam has been shown to be effective, safe and well tolerated, with few systemic adverse reactions. The majority of adverse reactions were infusion site reactions (predominantly local rash and inflammation), and none of those were severe. The number of reported infusion site reactions was highest during Stage 1, the period of more intensive observation; the number then fell and none were reported after 36 months, consistent with other studies [21]. The overall safety profile of Subgam was similar to that described by other workers studying SCIG in PID [12,14,20,21,22]. The post-licensing information showed infusion site reactions to be the most frequently reported adverse reactions (12 out of the 34, reports involving infusion site reaction), in line with the clinical study data. Consistent with previously published clinical studies of subcutaneous administration [13,14], the most commonly reported systemic adverse reactions to Subgam were flu-like symptoms such as pyrexia, rigors, shivering and/or similar which featured in ten cases. These may have been due, at least in some cases, to infusion in subjects with overt active infection [1].

In conclusion, the results indicate that the subcutaneous administration of Subgam is a safe, well-tolerated, convenient and effective replacement therapy for a range of immune deficiencies.

## Supporting Information

S1 File Clinical Study Report (part 1 of 2). This is part 1 of 2 of SCIG01 clinical study report. A multi-centre open study to assess the safety and efficacy of Subgam given via the subcutaneous route in primary antibody deficient patients (study code SCIG01).
(ZIP)

S2 File Clinical Study Report (part 2 of 2). This is part 2 of 2 of SCIG01 clinical study report. A multi-centre open study to assess the safety and efficacy of Subgam given via the subcutaneous route in primary antibody deficient patients (study code SCIG01)
(ZIP)

S1 Protocol This the SCIG01 protocol. A multi-centre, open study to assess the safety and efficacy of 16% immunoglobulin product given via the subcutaneous route in primary antibody deficient patients.
(PDF)

S1 TREND Checklist This is the TREND statement checklist. (PDF)
