# Supplementary material for: Experience with Subgam, a Subcutaneously Administered Human Normal Immunoglobulin (ClinicalTrials.gov - NCT02247141)
Source: PLoS One. 2015 Jul 29;10(7):e0131565. doi: 10.1371/journal.pone.0131565 (PMC4519338; doi:10.1371/journal.pone.0131565)
Supplement: S1 File — This is part 1 of 2 of SCIG01 clinical study report. A multi-centre open study to assess the safety and efficacy of Subgam given via the subcutaneous route in primary antibody deficient patients (study code SCIG01). (ZIP) [file pone.0131565.s001.zip › SCIG01 -clinical study report - S2 part 1 of 2.pdf]

CONFIDENTIAL

A MULTI-CENTRE OPEN STUDY TO ASSESS THE SAFETY AND EFFICACY OF SUBGAM® GIVEN VIA THE SUBCUTANEOUS ROUTE IN PRIMARY ANTIBODY DEFICIENT PATIENTS (STUDY CODE SCIG01)

CLINICAL STUDY REPORT

5 JULY 2007

Bio Products Laboratory
Dagger Lane
Elstree
Herts WD6 3BX

1 Title Page

**Study Title:**	A multi-centre, open study to assess the safety and efficacy of Subgam® given via the subcutaneous route in primary antibody deficient patients
**Name of Investigational Product:**	Human Normal Immunoglobulin (Subgam®)
**Indication Studied:**	Primary antibody deficiency
**Design of Study:**	A multi-centre, open, non-comparative safety and efficacy study in 50 patients with primary antibody deficiency
**Sponsor:**	Bio Products Laboratory (BPL), Dagger Lane, Elstree, Herts, WD6 3BX, United Kingdom
**Protocol Number:**	SCIG01
**Development Phase:**	Phase III (Continuing as Phase IV)
**Study Initiation:**	14 June 2000
**Date of Early Study Termination:**	N/A
**Study Completion:**	12 January 2005
**Principal Investigator:**	Dr HC Gooi, Consultant Clinical Immunologist, St James' University Hospital, Leeds

....

**Sponsor's Responsible Medical Officer:**	Dr C H Dash MB ChB FFPM (Consultant Medical Director, BPL)
**Regulatory Signatory:**	Dr C H Dash MB ChB FFPM (Consultant Medical Director, BPL)

....

Tel [redacted]

Fax [redacted]

CONFIDENTIAL

Statistician: [redacted]

....

Constella Group Ltd

Tel: [redacted]

Fax: [redacted]

Medical Writers: [redacted]

....

[redacted]

....

Bio Products Laboratory

Tel: +[redacted]

Fax: [redacted]

[redacted]

....

Constella Group Ltd

Tel: [redacted]

Fax: [redacted]

Date of Report: 5 July 2007

Replaces: SCIG01 Clinical Study Report dated 22 May 2003

This study was conducted in compliance with GCP CPMP/ICH/137/95.

2 Synopsis

Name of Sponsor/Company:

Bio Products Laboratory (BPL)

Dagger Lane, Elstree

Herts, WD6 3BX,

United Kingdom

Name of Finished Product:

Subgam®

Name of Active Ingredient:

Human Normal Immunoglobulin (for subcutaneous infusion).

Title of Study:

A multi-centre open study to assess the safety and efficacy of Subgam® given via the subcutaneous route in primary antibody deficient patients.

Investigators:

All investigative sites were in the United Kingdom.

Dr A Bansal – St Helier Hospital, Carshalton, Surrey

Dr H Chapel – John Radcliffe Hospital, Oxford

Dr P Darbyshire – Birmingham Children's Hospital, Birmingham

Dr M Duddridge – Leicester Royal Infirmary, Leicester

Dr W Egner – Northern General Hospital, Sheffield

Dr A Exley – Papworth Hospital, Papworth Everard, Cambridgeshire

Dr H Gooi – St James' University Hospital, Leeds

Dr M Haeney – Hope Hospital, Salford

Dr M Helbert / Dr H Longhurst – St Bartholomew's Hospital, London

Dr A Jones – Great Ormond Street Hospital, London

Dr V Nagendran / Dr C Tsakona – Guest Hospital, Dudley, West Midlands

Dr P Vijayadurai – Royal Preston Hospital, Preston, Lancashire

Prof J Warner – Southampton General Hospital, Southampton

Dr P Williams – University Hospital of Wales, Cardiff

Publications:

Dash CH, Gooi HC and Lynch TM. Subcutaneous immunoglobulin G (SCIG) in primary antibody deficiency: the UK multicentre study. Poster presentation at the 10th meeting of the European Society for ImmunoDeficiencies (ESID). Weimar, Germany, 17–20 October 2002.

Dash CH, Gooi HC, and Lynch TM. Subcutaneous immunoglobulin (SCIG) in primary antibody deficiency: the UK multicentre study. Poster presentation at the Joint Congress

of the British Society for Immunology and the British Society for Allergy and Clinical Immunology. Harrogate, UK, 3–6 December 2002.

Johnson N, Dash CH, Gooi HC et al. Patients views on subcutaneous IgG (SCIG) Home Therapy: results from the UK Subgam® study. Poster presentation at the 11th meeting of the European Society for Immunodeficiencies (ESID), Paris, France, 21–24 October 2004.

Dash CH, Johnson N, Gooi HC et al. Self-administration of subcutaneous immunoglobulin G (SCIG) in patients with primary antibody deficiency (PAD): The UK Subgam® Study. Poster presentation at the 11th meeting of the European Society for Immunodeficiencies (ESID), Paris, France, 21-24 October 2004.

Dash CH, Johnson N, Gooi HC et al. Patients views on subcutaneous IgG (SCIG) Home Therapy: results from the UK Subgam® study. Poster presentation at the Joint Congress of the British Society for Immunology and the British Society for Allergy and Clinical Immunology. Harrogate, UK, 8–10 December 2004.

Dash CH, Johnson N, Gooi HC et al. Self-administration of subcutaneous immunoglobulin G (SCIG) in patients with primary antibody deficiency (PAD): The UK Subgam® Study. Poster presentation at the Joint Congress of the British Society for Immunology and the British Society for Allergy and Clinical Immunology. Harrogate, UK, 8–10 December 2004.

Study period (for this report): Pre-study phase: 3 infusions on current immunoglobulin therapy; then Subgam®, starting 1 week after last dose of previous therapy. Patients received Subgam® for a mean of 147.7 infusions over a mean of 147.5 weeks.

The overall study duration was 4.58 years (calculated from the date of first enrolment to the date the last subject completed, i.e. 53 months).

Date of first enrolment: 14 June 2000

Date of last completed patient: 12 January 2005

Phase of development: Phase III (Continuing as Phase IV)

Objectives:

The primary objective was to determine the efficacy of Human Normal Immunoglobulin (Subgam®) given subcutaneously by weekly infusion to patients with primary antibody deficiency.

The secondary objective was to determine the safety of Subgam® given subcutaneously by weekly infusion to patients with primary antibody deficiency.

Methodology:

The study design was multi-centre, open and non-comparative. The study was performed in two parts: Stage 1 (safety and efficacy) and Stage 2 (long-term follow-up until product launch in the UK).

Safety was assessed by the number and types of adverse events, monitoring of vital signs, haematology, clinical chemistry, virology and local tolerability assessment.

Efficacy was measured by serum immunoglobulin G (IgG) levels, the number and magnitude of changes in Subgam® dose required, the number and type of infections incurred, antibiotic usage, specific antibody (*Streptococcus pneumoniae* [anti-pneumococcus] and *Haemophilus influenzae* type B [anti-HIB]) levels, the length of time off work or education, time spent on home therapy and patients' satisfaction with Subgam®.

Diary cards were used by patients to record doses of Subgam® administered at home, adverse events, infections and other relevant information.

Trough serum immunoglobulin G (IgG) levels were measured from samples taken weekly during hospital infusions and 4-weekly during home infusions for the first six months of the study. Thereafter, IgG levels were measured from samples taken during the patients' approximately 3-monthly routine hospital visits.

An optional pharmacokinetic assessment was performed in a group of patients who chose to take part. Blood samples were taken for serial measurement of serum IgG on two separate occasions (once during the week of the first infusion of Subgam® and once after approximately 3-4 months' treatment). On each of these occasions, a pre-dose blood sample was taken and then a sample on each of the following seven days (except weekends).

The data have been analysed in different ways: the whole population; adults, teenagers and children separately; those receiving previous IVIG or SCIG separately; patients with conditions associated with relatively low endogenous production of IgG (e.g. XLA, CVID) and others (e.g. specific antibody deficiency).

Number of patients:

A total of 50 patients: 15 paediatric patients <12 years (12 males and 3 females), 7 teenagers 12-<20 years (3 male, 4 female) and 28 adults (10 male, 18 female) received study medication. The mean age of the paediatric patients was 6.2 years (range 0.8-10.6), the mean age of teenagers was 15.2 years (range 12.1-18.0) and the mean age of adults was 45.5 years (range 21.3-75.2). All patients whose data were used in the efficacy analysis had a diagnosis of primary antibody deficiency and had received at least 6 months immunoglobulin (IVIG or SCIG) therapy prior to starting the study.

Diagnosis and main criteria for inclusion:

The main criteria for inclusion in the study were as follows:

- A diagnosis of primary antibody deficiency;
- No lower or upper age limit (any age was eligible);
- With stable disease and receiving immunoglobulin (IVIG or SCIG) therapy for at least six months prior to starting the study;
- Written informed consent (patient/parent/guardian).

Test product, dose and mode of administration, batch numbers:

Subgam® was given subcutaneously at an initial weekly dose of 100 mg/kg bodyweight. Doses were then adjusted for each patient in order to maintain adequate serum IgG levels.

A total of 12 separate batches of Subgam® were used during the 4.58 years of the study as follows:
SCBN276, SCBN276A SCBN5303, SCBN5565, SCBN5679, SCBN5786, SCBN5922, SCBN6004, SCBN6077, SCBN6079, SCBN6205, and SCBN6243.

SCBN276 and SCBN276A were manufactured as part of the same batch but were given different batch numbers as they were packaged and labelled in two separate lots.

Duration of treatment:

Stage 1: At least three infusions with prior IgG product (baseline) followed by six months (approx 27 infusions) with Subgam®, beginning one week after the last dose of the previous therapy, whether IVIG or SCIG.

Stage 2: Long term safety follow-up until Subgam® was marketed in the UK.

Patients received a mean of 147.7 infusions of Subgam®(range 22 to 317) over a mean of 147.5 weeks (range 24 to 208 weeks).

Reference therapy, dose and mode of administration, batch numbers:

There was no specific reference therapy.

Criteria for evaluation:

Efficacy:

Trough IgG levels:

The primary endpoint was the proportion of trough levels at each time point where serum IgG was ≥ 4 g/L for children and ≥ 6 g/L for adults.

Secondary efficacy measurements included: the change in Subgam® dose required to maintain trough levels at a minimum of ≥ 4 g/L for children and ≥ 6 g/L for adults; the proportion of trough levels at each time point where the IgG was ≥ 4 g/L for children and ≥ 6 g/L for adults; the time taken for each patient to reach a steady state IgG trough level, defined as the occurrence of three consecutive occasions when the IgG trough levels were within 1 g/L of each other; and the mean change in IgG trough level as compared to the baseline level at each time point.

Infections: the incidence of infections, days on antibiotics, and days off work/school.

Antibiotic usage: antibiotics included systemic antibiotics, but excluded topical fungicides.

Specific antibodies: during Stage 1 only, blood samples were taken for assay of antibodies to anti-pneumococcus and anti-HIB.

Immunology parameters: during Stage 1, and if clinically indicated in Stage 2, blood samples were taken for serum IgA and IgM measurements.

Length of time off work or education: the number of days each patient was absent from work or school (where applicable, some patients attended neither work nor school and the status of some patients changed during the 4.58 years of the study).

Home therapy: the length of home therapy training period and duration of home therapy.

Patient Satisfaction Questionnaires: patients' perception of using Subgam® compared to their previous IgG therapy.

Pharmacokinetics:

Inter-infusion serum IgG levels (optional) measured in a subgroup of patients who attended the hospital daily for blood sampling up to a week after the first Subgam® infusion and after an infusion approximately 3 months later.

Safety:

Monitoring of adverse events, infusion site reactions, laboratory assessments of haematology, clinical chemistry, and immunological and viral markers, and vital signs.

Statistical methods:

As this was a non-comparative study, data are presented with descriptive statistics only. The Pearson Chi-square test was used to test patients' perceptions of the comfort and convenience of infusions, their symptoms and how they liked Subgam® at the Month 6 visit. As expected cell frequencies were anticipated to be low for some categories, the exact version of this test was used.

Summary - Results:

In total 51 patients entered the study from 14 study centres; one patient was withdrawn before receiving study mediation, and has been excluded from all data analysis. Of the remaining 50 patients, 35 (70%) completed the study (up to 4.58 years). Fifteen patients withdrew prematurely; one patient withdrew from Stage 1 (patient 9 after Infusion 25) because he emigrated, and the remaining 14 patients withdrew during Stage 2. Of the 14 patients that withdrew in Stage 2, 10 were at the patient's request, 2 for protocol deviations, 1 at the request of the investigator, and 2 for other reasons (one patient returned to IVIG therapy and the other patient did not wish to continue completing diary data after 995 days in the study).

Efficacy Results:

During Stage 1 of treatment, 85% of adults/teenagers and 93% of children achieved target trough serum IgG levels at all observations. The mean time to achieve a steady state serum IgG level was 6.14 infusions for patients who had previously received IVIG treatment and 6.50 infusions for patients who had previously received SCIG treatment.

Mean IgG level increased from 9.2 g/L to 9.75 g/L with Subgam® treatment and was maintained above the pre-Subgam® level for 30 months of treatment. For patients completing at least 36 months on Subgam®, the mean IgG levels for the periods 30-36 months and 0-6 months were compared by calculating the mean level for each patient in the 6 month period and then the overall mean for all patients who completed 36 months. The value for the 30-36 month period was similar (95.9%) to that in the first 6 months.

Overall, 93.6% of infusions were given at home. The dose of Subgam® required to maintain IgG levels increased with time from 104.6 mg/kg during the first 6 months, with the required dose reaching a plateau of around 115 mg/kg at around 18-24 months of treatment. The trend in increased dose was seen in adults and children; however there was no noticeable increase in dose with time observed in teenagers.

There was no notable change in serum IgA or IgM levels during Subgam® treatment.

There was no marked increase in the frequency, severity or seriousness of bacterial infections prior to and during Subgam® treatment. Among the 50 patients there were 3.40 infections per patient per year in the pre-Subgam® treatment phase and 3.62 infections per patient per year during Subgam® treatment; similarly patients experienced 0.19 serious infections per patient per year pre Subgam® treatment, and 0.16 serious infections per patient per year during Subgam® treatment. There were 18 potential serious acute bacterial infections in 11 patients during Subgam® treatment, equivalent to 0.13 serious acute bacterial infections per patient per year.

During the pre-Subgam® treatment phase, 24 patients received antibiotics for 32.8% of all study days. During the longer Subgam® treatment phase, 49 of the 50 patients received antibiotics for a total of 40.3% of study days. Ten patients required intravenous antibiotics during the Subgam® treatment phase for 0.9% of all study days.

Applicable patients who had previously received SCIG treatment experienced a higher percentage of days off work or school than patients who had previously received IVIG. In total, all applicable patients took a mean of 2.9% of study days off work or school, prior IVIG patients took 1.7% of days off work or school and prior SCIG patients took 7.5% of study days off work or school. Adults, teenagers and children took approximately the same amount of time off work or school (2.9%, 1.8% and 3.7% of days off school or work respectively). Patients were not randomised to treatment in this study, so these differences can only be considered as descriptive of the populations.

The majority of patients preferred Subgam® treatment to their previous therapy. After 3 months of treatment, 34 of 38 patients (who answered the questionnaire) preferred Subgam® "more" or "much more" than their previous treatment and after 6 months of

treatment patients continued to prefer Subgam®. There was no noteworthy difference between patients previously treated with SCIG or IVIG.

Safety Results:

Overall, Subgam® was safe and well tolerated, with no increase in adverse events experienced during the treatment phase. Patients receiving Subgam® therapy experienced a similar number of adverse events and SAEs per study day compared with patients pre-Subgam® therapy. The most common adverse events were headache, cough and pharyngitis and the majority of these were considered unrelated to Subgam®. There were 82 reports of infusion site reactions in 25 of the 50 patients receiving Subgam® therapy. Apart from infusion site reactions, the most common product-related adverse events were headache (8 reports in 7 patients), pruritus (7 reports in 2 patients) and vomiting (5 reports in 3 patients). There were no withdrawals due to adverse events or safety considerations and no product-related SAEs.

There was no notable overall change in haematology or biochemistry parameters before and after participation in the study. There were no clinically relevant changes in individual haematology or biochemistry parameters that were considered to be related to Subgam®.

There was no evidence of the transmission of HIV, Hepatitis B virus, Hepatitis C virus or Parvovirus B19.

Conclusion:

Subgam® was safe and well tolerated and was effective in increasing and maintaining serum IgG levels with only moderate dose increases required over time. The incidence of bacterial infections remained low during long-term treatment with Subgam®. The majority of patients in the study preferred Subgam® treatment to their previous treatments.

Date of Report: 5 July 2007

3 Table of Contents

1 Title Page 2

2 Synopsis 4

3 Table of Contents 10

4 List of Abbreviations and Definitions of Terms 15

5 Ethics 18
5.1 Independent Ethics Committee (IEC) 18
5.2 Ethical Conduct of the Study 18
5.3 Patient Information and Consent 18

6 Investigators and Study Administration Structure 20

7 Introduction 22

8 Study Objectives 23
8.1 Primary Objective 23
8.2 Secondary Objective 24

9 Investigational Plan 24
9.1 Overall Study Design and Plan Description 24
9.2 Discussion of Study Design and Choice of Controls 26
9.3 Selection of Study Population 29
9.3.1 Inclusion Criteria 29
9.3.2 Exclusion Criteria 30
9.3.3 Removal of Patients from Therapy or Assessment 31
9.4 Treatments 32
9.4.1 Treatments Administered 32
9.4.2 Identity of Investigational Product 32
9.4.3 Method of Assigning Patients to Treatment Groups 33
9.4.4 Selection of Doses in the Study 33
9.4.5 Selection and Timing of Dose for each Patient 34
9.4.6 Prior and Concomitant Medication 34
9.4.7 Treatment Compliance 35
9.5 Efficacy and Safety Variables 35
9.5.1 Efficacy and Safety Variables Assessed and Flow Chart 35
9.5.2 Drug Concentration Measurements 43
9.5.3 Appropriateness of Measurements 43
9.6 Data Quality Assurance 44
9.7 Statistical Methods Planned in The Protocol and Determination of Sample Size 45
9.7.1 Determination of Sample Size 45
9.7.2 General Considerations 46
9.7.3 Analysis Population 46
9.7.4 Subgroups 47
9.7.5 Demographics and Baseline Characteristics 48
9.7.6 Pharmacokinetic Data 48
9.7.7 Efficacy 48
9.7.8 Safety 53
9.8 Changes in Study Conduct or Planned Analysis 54
9.8.1 Changes to the Protocol 54
9.8.2 Changes to Sample Size 55

9.8.3 Changes to Planned Analyses 55

10 Study Patients 56
10.1 Disposition of Patients 56
10.2 Protocol Deviations 57

11 Efficacy Evaluation 58
11.1 Data Sets Analysed 58
11.1.1 Completers Subgroup 59
11.2 Demographic and other Baseline Characteristics 59
11.2.1 Demography 59
11.2.2 Primary Antibody Deficiency Disease Characteristics 60
11.2.3 Direct Coombs' Test and Blood Group Serology 62
11.2.4 Prior and Current Medical Conditions 63
11.2.5 Prior IgG Therapy and Baseline Serum IgG Levels 63
11.3 Treatment Compliance 64
11.4 Efficacy Evaluation 65
11.4.1 Analysis of Efficacy 65
11.4.2 Tabulation of Individual Efficacy Response Data 102
11.4.3 Drug Dose, Drug Concentration and Relationships to Response 102
11.4.4 Drug-Dose and Drug-Disease Interactions 102
11.4.5 By-Patient Displays 102
11.4.6 Efficacy Conclusions 102

12 Safety Evaluation 104
12.1 Extent of Exposure 104
12.1.1 Total Subgam® Exposure Over Study Duration 104
12.1.2 Interval Between Subgam® Infusions 105
12.2 Adverse Events 106
12.2.1 Summary of Adverse Events 106
12.2.2 Display of Adverse Events 107
12.2.3 Product-Related Adverse Events 109
12.2.4 Infusion Site Reactions 109
12.3 Deaths and Other Serious Adverse Events 111
12.3.1 Listings of Deaths and Other Serious Adverse Events 111
12.3.2 Narratives of Deaths and Other Serious Adverse Events 112
12.4 Clinical Laboratory Evaluations 112
12.4.1 Listing of Individual Laboratory Measurements by Patient and Each Abnormal Laboratory Value 113
12.4.2 Evaluation of Each Laboratory Parameter 113
12.4.3 Virology 119
12.5 Vital Signs, Physical Findings, Other Observations related to Safety 119
12.5.1 Vital Signs 119
12.5.2 Concomitant Therapy 122
12.6 Safety Conclusions 122

13 Discussion and Overall Conclusion 123

14 Tables, Figures and Graphs 126
14.1 Demographic Data 126
14.2 Efficacy Data 126
14.2.1 Narratives of Potential Serious Acute Bacterial Infections 126
14.3 Safety Data 175
14.3.1 Adverse Events (Excluding Infections) 175
14.3.2 Deaths and Other Serious and Significant Adverse Events (Excluding Infections) 175
14.3.3 Infusion Site Reactions 175
14.3.4 Laboratory Data 175
14.3.5 Vital Signs 175

15 References 175

16 Appendices 177
16.1 Study Information 177
16.1.1 Protocol and Protocol Amendments 177
16.1.2 Sample Case Report Form (Unique Pages Only) and Other Forms Used for Data Collection 177
16.1.3 List of IECs and MRECs 177
16.1.4 List and Description of Investigators 177
16.1.5 Responsible Persons at BPL Signature Page 177
16.1.6 List of Batch Numbers Received by All Patients 177
16.1.7 Randomization Scheme and Codes 177
16.1.8 Audit Certificates 177
16.1.9 Documentation of Statistical Methods (including Statistical Analysis Plan and Filenotes 001, 002, 003 and 004) 177
16.1.10 Documentation of Inter-Laboratory Standardization Methods and Quality Assurance Procedures 177
16.1.11 Publications Based on the Study 177
16.1.12 Important Publications Referenced in the Report 177
16.2 Patient Data Listings 178
16.2.1 Patients Who Discontinued Therapy 178
16.2.2 Protocol Deviations 178
16.2.3 Listings for Patients Excluded from Efficacy and Safety Analysis 178
16.2.4 Demographic Data 178
16.2.5 Compliance and Drug Concentration Data 178
16.2.6 Individual Efficacy Response Data 178
16.2.7 Adverse Event Listings 178
16.2.8 Laboratory Measurements 178
16.2.9 Other Data Listings 178
16.3 Case Report Forms 179
16.3.1 CRFs of Deaths, Withdrawals Due to SAEs 179
16.3.2 Other CRFs 179

List of in Text Tables

Table 1: Schedule of Assessments 36
Table 2: Time Taken to Start Home Therapy 51
Table 3: Patient Identification 56
Table 4: Patient Disposition (All Patients) 56
Table 5: Reasons for Withdrawal from Study 57
Table 6: Duration of Patients' Participation in the Study 57
Table 7: Summary of Demography (at Enrolment) 59
Table 8: Summary of Disease Characteristics: History of Primary Antibody Deficiency 60
Table 9: Time from Onset and Diagnosis of Primary Antibody Deficiency to Study Start by Gender and Age Group 61
Table 10: Summary of Blood Group Serology 62
Table 11: Summary of Coombs' Test Results by Gender 62
Table 12: Prior IgG Therapy and Mean (Range) Baseline Serum IgG Levels (g/L) by Age Group and Prior Therapy (Infusions 1-3) 64
Table 13: Number of Observations of <4 g/L Serum IgG levels and Patients who had at Least 1 Observation <4 g/L Serum IgG Levels Across the Whole Study - Children (<12 y) 65
Table 14: Number of Observations of <6 g/L Serum IgG levels and Patients who had at Least 1 Observation <6 g/L Serum IgG Levels Across the Whole Study - Teenagers and Adults (≥12 y) 67
Table 15: Mean Serum IgG Levels (g/L) by 6-Monthly Intervals 76
Table 16: Daily Inter-Infusion Total Serum IgG Values (g/L) 79
Table 17: Mean Subgam® Dose (mg/kg) by 6-Monthly Intervals 81
Table 18: Number of Increases and Decreases in Subgam® Dose (mL) Across the Whole Study 84
Table 19: Minimum and Maximum Doses as a Percentage of the Starting Dose (All Patients) 85
Table 20: Minimum and Maximum Doses for Those Patients with a Minimum Subgam® Dose of <50% of Starting Dose or a Maximum Subgam® Dose of >150% of Starting Dose 86
Table 21: Number of Subgam® Infusions Compared with Target for Weekly Dosing 87
Table 22: Number of Infections Documented Across the Study - All Patients 89
Table 23: Potential Serious Acute Bacterial Infections During Subgam® Treatment 90
Table 24: Days on Antibiotics During Subgam® Treatment 93
Table 25: Summary of Numbers of Days Off Work or School During Subgam® Treatment by Prior Therapy 95
Table 26: Summary of Numbers of Days Off Work or School by Age During Subgam® Treatment. 96
Table 27: Subgam® Infusions Given as Home Therapy 97
Table 28: Preference of Subgam® Compared to Previous Medication (After 3 Months) 97
Table 29: Preference of Subgam® Compared to Previous Medication (After 6 Months) 98
Table 30: Patients' Perception of Symptoms on Subgam® Compared to Previous Medication (After 3 Months) 99
Table 31: Patients' Perception of Symptoms on Subgam® Compared to Previous Medication (After 6 Months) 100
Table 32: Patients' Perception of Convenience on Subgam® Compared to Previous Medication (after 3 Months) 101
Table 33: Patients' Perception of Comfort on Subgam® Compared to Previous Medication (After 3 Months) 102
Table 34: Total Subgam® Exposure – All Patients 105
Table 35: Interval Between Infusions (Days) 106
Table 36: Summary of Adverse Events Excluding Infections 107
Table 37: Infusion Site Reactions (Regardless of Causality) in All Patients During Subgam® (Infusion 4 to End of Study) 107
Table 38: Summary of Adverse Events by System Organ Class – During Subgam® (Infusion 4 to End of Study) 108
Table 39: Common Adverse Events (>5% of total) by Severity and Relationship 109
Table 40: Summary of Product-Related Infusion Site Reactions in 6-Monthly Intervals – by Diagnosis of PAD, Age Group and Prior Therapy 110
Table 41: Serious Adverse Events by Severity (Infusion 1 to the End of Study) 112
Table 42: Haematology Parameters 113

Table 43: Biochemistry Parameters 116
Table 44: Pre- and Post-Subgam® Vital Signs and Physical Findings 120
Table 45: Pre- and Post-Subgam® Body Temperatures >37°C During Stage 1 120
Table 46: Adverse Events During Subgam® With Preferred Term of Pyrexia or Body Temperature Increased 121
Table 47: Potential Serious Acute Bacterial Infection by Narrative Number 126

List of in Text Figures

Figure 1: Flow Chart (Stage 1) 27
Figure 2: Flow Chart (Stage 2) 28
Figure 3: Mean of Means Serum IgG Levels in Stage 1, by Diagnosis of PAD 74
Figure 4: Mean of Means Serum IgG Levels in Stage 1, by Age Group 75
Figure 5: Mean of Means Serum IgG Levels in 6-Monthly Intervals up to 54 months, by Diagnosis of PAD 77
Figure 6: Mean of Means Serum IgG Levels in 6-Monthly Intervals up to 54 months, by Age Group 78
Figure 7: Mean of Means of Subgam® Dosages (mg/kg) Received in 6-Monthly Intervals up to 54 months, by Age Group 82
Figure 8: 'Mean of Means' of Subgam® Dosage (mg/kg) Received in 6-Monthly Intervals up to 54 months, by Prior Therapy 83

4 List of Abbreviations and Definitions of Terms

ABO (D)	Blood Group Nomenclature
AE	Adverse Event
ALT	Alanine Aminotransferase
AST	Aspartate Aminotranferase
B19	Parvovirus B19
bd	*bis die* (twice a day)
BMI	Body Mass Index
BP	Blood Pressure
BPL	Bio Products Laboratory (the Sponsor)
bpm	Beats Per Minute or Breaths Per Minute (depending on context)
CI	Confidence Interval (*e.g.* 95% CI)
COPD	Chronic Obstructive Pulmonary Disease
CPA	Clinical Pathology Accreditation
CPMP	Committee for Proprietary Medicinal Products (now CHMP, Committee for Human Medicinal Products)
CRA	Clinical Research Associate
CRF	Case Report Form
CRP	C-Reactive Protein
CTA	Clinical Trial Authorisation
CTX	Clinical Trial Certificate Exemption
CV	Curriculum Vitae
CVID	Common Variable Immunodeficiency
DBP	Diastolic Blood Pressure
DCT	Direct Coombs' test
dL	Decilitre
dp	Decimal point
e.g.	*Exempli gratia* (for example)
ENT	Ear, Nose and Throat
EOP1	End of Phase 1
EOS	End of Study
EOS1	End of Stage 1
etc	*Et cetera* (and so on)
F	Female
fl	Femtolitre
g	Gram
GCP	Good Clinical Practice
GI	Gastrointestinal
GMP	Good Manufacturing Practice
GGT	Gamma Glutamyl Transferase
GP	General Practitioner
h	Hour
HAV	Hepatitis A virus
Hb	Haemoglobin
HBsAg	Hepatitis B Surface Antigen
HBV	Hepatitis B Virus
HCT	Haematocrit
HCV	Hepatitis C Virus
HIB	*Haemophilus influenzae* type b
HIV	Human Immunodeficiency Virus
HPA	Health Protection Agency

HRT	Hormone Replacement Therapy
ICH	International Conference on Harmonisation
ICI	If Clinically Indicated
IEC	Independent Ethics Committee
IgA	Immunoglobulin A
IgG	Immunoglobulin G
IgM	Immunoglobulin M
IM	Intramuscular
IMIG	Intramuscular Immunoglobulin
INF	Infusion
IRB	Institutional Review Board
ITT	Intention to Treat
IV	Intravenous
IVIG	Intravenous Immunoglobulin
kg	Kilogram
L	Litre
LDH	Lactate Dehydrogenase
LFT	Liver Function Test
LREC	Local Research Ethics Committee
LRTI	Lower Respiratory Tract Infection
M	Male
max	Maximum
MCA	Medicines Control Agency (now the MHRA)
μg	Microgram
MedDRA	Medical Dictionary for Drug Regulatory Affairs
mg	Milligram
MHRA	Medicines and Healthcare products Regulatory Agency (formerly the MCA)
min	Minimum
mL	Millilitre
mmHg	Millimetres of Mercury
mmol	Millimoles
μmol	Micromoles
MREC	Multi-Centre Research Ethics Committee
MSU	Mid Stream Urine
n	Sample Size
NBA	National Blood Authority (now NHSBT, National Health Service Blood and Transplant)
ND	Not Done
NEC	Not Elsewhere Classified
NHS	(UK) National Health Service
nocte	At night
NOS	Not Otherwise Specified
NS	Not Specified
od	*omnes dies* (every day)
qds	*quater die sumendus* (to be taken four times daily)
qid	*quater in die* (four times a day)
PAD	Primary Antibody Deficiency Syndrome
PCR	Polymerase Chain Reaction
pg	Picogram
PHLS	Public Health Laboratory Service (now HPA, Health Protection Agency)
PICC	Peripherally Inserted Central Catheters
PK	Pharmacokinetics

PL	Product Licence
prn	*pro re nata* (as needed)
RBC	Red Blood Cell
RNA	Ribonucleic Acid
SABI	Serious Acute Bacterial Infection
SAD	Secondary Antibody Deficiency
SAE	Serious Adverse Event
SAP	Statistical Analysis Plan
SC	Subcutaneous
SCIG	Subcutaneous Immunoglobulin
SD	Standard Deviation
S/D-HNIG	BPL's Solvent/Detergent Treated Human Normal IgG
SDV	Source Document Verification
ST	Summary Table
SD	Standard Deviation
Subgam®	Subgam® is a registered trademark of the NHS Blood & Transplant, trading as BPL, for Human Normal Immunoglobulin
tds	*ter die sumendum* (to be taken three times a day)
U/L	Units per Litre
URTI	Upper Respiratory Tract Infection
UTI	Urinary Tract Infection
UK	United Kingdom
y	Year
vCJD	Variant Creutzfeldt-Jakob Disease
Vigam®	Vigam® is a registered trademark of the NHS Blood & Transplant, trading as BPL, for Normal Immunoglobulin, Intravenous
WBC	White Blood Cell
WHO	World Health Organisation
w/w	Weight for Weight
XLA	X-linked Agammaglobulinaemia

5 Ethics

5.1 Independent Ethics Committee (IEC)

Before the start of the study, the protocol (SCIG01, version 1 dated April 1999) was reviewed and conditionally approved by the Northern and Yorkshire Multi-Centre Research Ethics Committee (MREC). On implementing the changes requested by the MREC, the amended protocol (version 2 dated September 1999) was approved on 11th November 1999.

Final Version 3 of the protocol (April 2000, incorporating Amendment 1, dated 31st March 2000) was approved by the MREC on 18th April 2000.

Local Research Ethics Committee (LREC) approval was obtained at each site prior to enrolment. The list of committees is given in Appendix 16.1.3.1.

As a result of emerging guidelines (see Section 7), further amendment to the protocol (Amendment 2, dated 25th July 2001) was approved by the MREC on 16th August 2001. The final amendment to the protocol (Amendment 3, dated 29th January 2004) was approved by the MREC on 8th March 2004.

Further details on the nature of the protocol amendments are found in Section 9.8.1.

5.2 Ethical Conduct of the Study

The study was carried out in accordance with the International Conference on Harmonisation (ICH) Guideline for Good Clinical Practice and the Declaration of Helsinki (South Africa, 1996).

This study was initiated after obtaining approval from the UK Medicines Control Agency (now known as the Medicines and Healthcare products Regulatory Agency, or MHRA), under UK Clinical Trial Certificate Exemption (CTX) number 08801/0046/A.

5.3 Patient Information and Consent

Before they were screened for the study, all patients gave written informed consent to participate. For patients under 18 years of age, written informed

consent was obtained from the patient's parent or legal representative. Before giving consent, patients were given the opportunity to read and digest the Patient Information Leaflet, which contained information about the trial product and the study design. They then discussed the study with the investigator and had the opportunity to ask questions.

There were separate Patient Information Leaflets and Consent Forms for adults, children under the age of 18, and parents/guardians of children under the age of 18. A separate home therapy consent form was signed when the patient was ready to start home therapy training. In addition, patients who chose to take part in the optional inter-infusion serum immunoglobulin G (IgG) pharmacokinetic assessment were required to sign an additional consent form.

The text contained within the patient information leaflets and the consent forms was approved by the MREC prior to the start of the study (examples are included in Appendices 16.1.3.2 and 16.1.3.3 respectively). If requested by the LREC, modifications were made to information leaflets and consent forms prior to their use at specific investigator sites (examples of the changes requested can be found in Appendix 16.1.3.4).

6 Investigators and Study Administration Structure

**PRINCIPAL INVESTIGATOR**	Dr H Gooi<br>Address: St James' University Hospital, Leeds
**Investigators**	Dr A Bansal<br>Address: St Helier Hospital, Carshalton
	Dr H Chapel<br>Address: John Radcliffe Hospital, Oxford
	Dr P Darbyshire<br>Address: Birmingham Children's Hospital, Birmingham
	Dr M Duddridge<br>Address: Leicester Royal Infirmary, Leicester
	Dr W Egner<br>Address: Northern General Hospital, Sheffield
	Dr A Exley<br>Address: Papworth Hospital, Papworth Everard
	Dr M Haeney<br>Address: Hope Hospital, Salford
	Dr M Helbert, replaced by Dr H Longhurst in January 2003<br>Address: St Bartholomew's Hospital, London
	Dr A Jones<br>Address: Great Ormond Street Hospital, London
	Dr V Nagendran, replaced by Dr C Tsakona in October 2003<br>Address: Guest Hospital, Dudley
	Dr P Vijayadurai<br>Address: Royal Preston Hospital, Preston
	Prof J Warner<br>Address: Southampton General Hospital, Southampton
	Dr D Webster<br>Address: Royal Free Hospital, London<br>*(This site did not recruit any patients.)*
	Dr P Williams<br>Address: University Hospital of Wales, Cardiff

All investigative sites were in the United Kingdom. All investigators were either Consultant Immunologists or Consultant Paediatric Immunologists and one was a Professor of Child Health. All had specific training in their respective specialties. Details of investigators are given in Appendix 16.1.4.1. Each investigator signed a copy of the protocol, before embarking on the study, as part of their agreement to participate.

At some sites, sub-investigators (departmental registrars or consultants) assisted with the study under the supervision of the investigator. For most sites, patient infusions, blood sample collections, clinical measurements, home therapy training and much of the administrative paperwork were the responsibility of the Nurse

Specialist for that department. The signatures of all key participating staff were also collected at each investigative site. Details are given in Appendix 16.1.4.2.

Clinical monitoring (collating and reporting of adverse events) was carried out by the sponsor's Consultant Medical Director (Dr CH Dash) and Medical Affairs Manager (Dr EW Gascoigne). Details are given in Appendix 16.1.4.3.

Statistical aspects were carried out by the Constella Group Ltd (formerly Origin Pharmaceutical Services Ltd), 20 Milton Park, Abingdon, Oxfordshire, OX14 5RL [Mark Baillet (Head of Biometrics) in collaboration with the sponsor's Medical Affairs Manager (Dr EW Gascoigne)].

Audit and GCP training was carried out by Orla Cantwell, Datawise Consultants Ltd. (Co. Wicklow, Ireland).

The polymerase chain reaction (PCR) tests for Parvovirus B19, human immunodeficiency virus (HIV), hepatitis C virus (HCV) as well as the tests for hepatitis B surface antigen (HBsAg) were carried out by the UK Public Health Laboratory Service (PHLS) Colindale, London, now known as the Health Protection Agency (HPA). All other blood tests were carried out by local hospital laboratories (see Appendix 16.1.10 for details).

The clinical trial supply was prepared under GMP conditions at BPL's manufacturing facility at Elstree, UK. This facility is regularly inspected by the Inspectorate Division of the UK Medicines and Healthcare products Regulatory Agency (MHRA). Vials for clinical trial use were segregated at the manufacturing plant from other materials. They were released and despatched to the investigative sites upon request from a responsible person in the Medical Department of BPL. Records of despatch and receipt at the hospital pharmacy were maintained, as were the records of dispensing to patients. Drug reconciliation was performed by the Clinical Research Associates (CRA) assigned to the study, when monitoring the sites.

The study was conducted under the UK CTX Scheme (CTX number: 08801/0046/A) and was converted to the CTA scheme by the MHRA in May 2004.

Details of relevant personnel are given in Section 16.1.4.

7 Introduction

Patients who are diagnosed with primary antibody deficiency require regular immunoglobulin replacement therapy in order to prevent, or reduce the severity / frequency of infection and complications. The efficacy of life-long intravenous immunoglobulin (IVIG) therapy is well established and is a major contributor to improved health and quality of life for these patients. IVIG treatment for most patients requires regular visits to hospital where the infusion can take many hours to administer depending on the dose and tolerance to treatment. Some patients are able to have home IVIG therapy. However the disadvantages with this form of treatment are: relative immobility during infusions; possible difficulty with venous access and the length of time required.

Intramuscular and subcutaneous infusions of immunoglobulins are alternatives to IVIG. Intramuscular injections are often painful, the dose that can be comfortably delivered is limited and the side effects can be troublesome. Previously, subcutaneous infusions of immunoglobulin were given by slow infusion, but although they were preferable to intravenous infusions as they had a better safety profile, they were time-consuming, usually requiring overnight infusions.

Rapid subcutaneous infusions of immunoglobulin preparations intended for intramuscular use were first described by Gardulf *et al.* (1991). Patients were given simultaneous infusions totalling 34–40 mL/h via dual portable pumps. The duration of infusion was about one hour. In addition to there being very few (0.93%) mild systemic reactions experienced with this method of administration, patients were also able to infuse the immunoglobulin readily at home after a period of training.

The trial product (Subgam®) was manufactured by Bio Products Laboratory (BPL) and was based on one of their licensed Normal Immunoglobulin products (product licence number PL08801/0005) for intramuscular injection (IMIG). The major constituent of the IMIG product is IgG, and one of its licensed indications is as replacement therapy in primary antibody deficiency. Plasma for manufacture of the product was obtained from screened donors in the United States and during manufacture a solvent/detergent step was included, which inactivates lipid-

enveloped viruses such as HIV, hepatitis C (HCV) and hepatitis B (HBV). The product does not contain any mercurial preservative, or stabilisers such as albumin or sugars, and has a very low IgA content (<0.02% w/w).

The purpose of this study was to determine the efficacy and safety of this licensed 16% Human Normal Immunoglobulin product given by a different route of administration (namely as a 'rapid' subcutaneous infusion) to patients with primary antibody deficiency.

It was decided that Subgam® would be given subcutaneously at an initial weekly dose of 100 mg/kg bodyweight; this was based on the study by Gardulf *et al.* (1991). Unit dose and/or dose frequency were adjusted according to the patient's clinical condition and to maintain a pre-infusion serum IgG level of at least 4 g/L (children) or 6 g/L (adults).

At the time of preparing the study protocols there were no Committee on Proprietary Medicinal Products (CPMP) guidelines for the evaluation of subcutaneous immunoglobulins (SCIG). Therefore, the study protocol was based on the guidelines for the evaluation of intravenous immunoglobulins (CPMP/388/95, 1996 and CPMP/BPWG/388/95 revision 1, 2000). The protocol was subsequently modified to ensure that sufficient numbers of children were enrolled according to the subsequent draft and then final subcutaneous IgG (SCIG) guidelines; namely CPMP/BPWG/283/00 (2001) and CPMP/BPWG/280/00 (2002).

8 Study Objectives

The objectives of the study, as stated in the protocol, were as follows:

8.1 Primary Objective

The primary objective of the study was to determine the efficacy of giving patients with primary antibody deficiency syndrome, weekly subcutaneous infusions of a 16% immunoglobulin product at a dose tailored to achieve a minimum IgG trough level of 4 g/L for young children and 6 g/L for adults. (The dose was adjusted to achieve minimum serum IgG trough levels as described in Section 9.4.4).

8.2 Secondary Objective

The secondary objective of the study was to determine the safety of weekly subcutaneous infusions of Subgam® in patients with primary antibody deficiency syndrome.

9 Investigational Plan

9.1 Overall Study Design and Plan Description

The protocol and amendments can be seen in Appendix 16.1.1 and a sample Case Report Form (CRF) is provided in Appendix 16.1.2.

This multi-centre study was open, and non-comparative in design. Fifty-one patients entered the study. One patient was withdrawn prior to receiving study medication and has been excluded from the data analysis. Fifty patients, 35 adults / teenagers (aged ≥ 12 years) and 15 children (aged < 12 years at enrolment) received study medication. All patients whose data were used in the efficacy analysis had a diagnosis of stable primary antibody deficiency syndrome and a history of at least six months prior IgG therapy for their condition before entering the study.

Prior to entering the study, patients were asked to attend a screening visit where they gave consent to participate. Patients gave their medical and medication history, and they underwent a physical examination (including height, weight, seated blood pressure, pulse, temperature and respiration rate). Blood samples were taken for routine haematology, biochemistry, virology and immunology tests, and the patients completed 'Patient Satisfaction Questionnaires' for their current IgG therapy.

For children entering the study, where the investigator determined them to be of sufficient maturity to understand the study, then the child signed their own consent. For children who were not able to understand the full implication in taking part in the study, the parent / guardian gave consent.

If the patient fulfilled all of the entry criteria, they were then entered into the study.

The study was divided into two stages:

Stage 1 (Six Months Safety and Efficacy)

Patients received their usual IgG therapy for the first three infusions of the study; baseline data were collected during this period. Following these baseline infusions, patients received weekly infusions of Subgam® (i.e., Infusions 4–30); initially, infusions were given at the hospital. Suitable patients were trained by an experienced Immunology Nurse Specialist to self-infuse with Subgam®. Patients were then able to move onto Home Therapy where infusions were self-administered at home with the assistance of a trained adult.

Vital signs were measured before, during and after all infusions in hospital. Blood samples were taken prior to each weekly infusion for measurement of trough immunoglobulin levels and 4-weekly for anti-pneumococcus and *Haemophilus influenzae* type b (anti-HIB) specific antibodies, aspartate aminotransferase (AST) and/or alanine aminotransferase (ALT). Patient Satisfaction Questionnaires were completed at Infusion 16 and End of Stage 1 (after three and six months treatment with Subgam®).

While on Home Therapy, patients completed diary cards to record infusion details, adverse events and concomitant medication. Samples for trough immunoglobulins, anti-pneumococcus and anti-HIB specific antibodies and AST and/or ALT were taken every four weeks.

Adult patients who lived near to their hospital were asked to take part in an additional pharmacokinetic inter-infusion pharmacokinetic sub-study to determine inter-infusion IgG levels. For those patients who consented to take part, blood samples for measurement of total serum IgG were taken once daily (except weekends) between two consecutive infusions on two separate occasions (once during the week of the first infusion with study medication and once more after the patient had been on study treatment for approximately 3–4 months). On each occasion, a blood sample was taken prior to infusion with study medication, and then blood samples were taken on each of the following seven days (except weekends).

Stage 2 (Long-Term Safety Follow-up)

At the End of Stage 1, patients were invited to enter the safety follow-on study (Stage 2), during which they continued receiving Subgam®, which could be given either in hospital or at home. Patients continued in Stage 2 until such time that they wished to discontinue treatment or the product was available on the UK market. Patients were asked to attend study visits at the clinic every three months throughout Stage 2, at which they were asked whether they had experienced any adverse events since the last visit. If clinically indicated, blood samples for haematology, biochemistry and immunology would also be taken. On withdrawal from the study (whether during Stage 1 or Stage 2), or at the End of Study visit, vital signs were recorded and a blood sample for virology and an archive blood sample were collected.

While the study was ongoing, the two stages of the study were referred to as Phase 1 and Phase 2. To avoid confusion with the different phases of clinical research, the terminology was changed to Stage 1 and Stage 2; however, some references to End of Phase 1 (EOP1) and End of Phase 2 (EOP2) persist in Appendix 16.2.

9.2 Discussion of Study Design and Choice of Controls

The study design selected (open, uncontrolled) was based on the CPMP guidelines for assessing the efficacy and safety of normal intravenous immunoglobulin products for modified marketed products (see CPMP/388/95, 1996) in the absence of any guidelines for subcutaneous IgG.

The study design used was also based on experience from previous SCIG studies, namely: Gardulf *et al.* (1991); Gardulf *et al.* (1995); Gasper *et al.* (1998). The draft CPMP guidelines for the evaluation of SCIG were released for consultation in March 2001 (CPMP/BPWG/283/00, 2001), and came into operation in January 2003. However, the design of the current study was very similar to that recommended in the final CPMP guidelines (CPMP/BPWG/283/00, 2002).

Figure 1: Flow Chart (Stage 1)

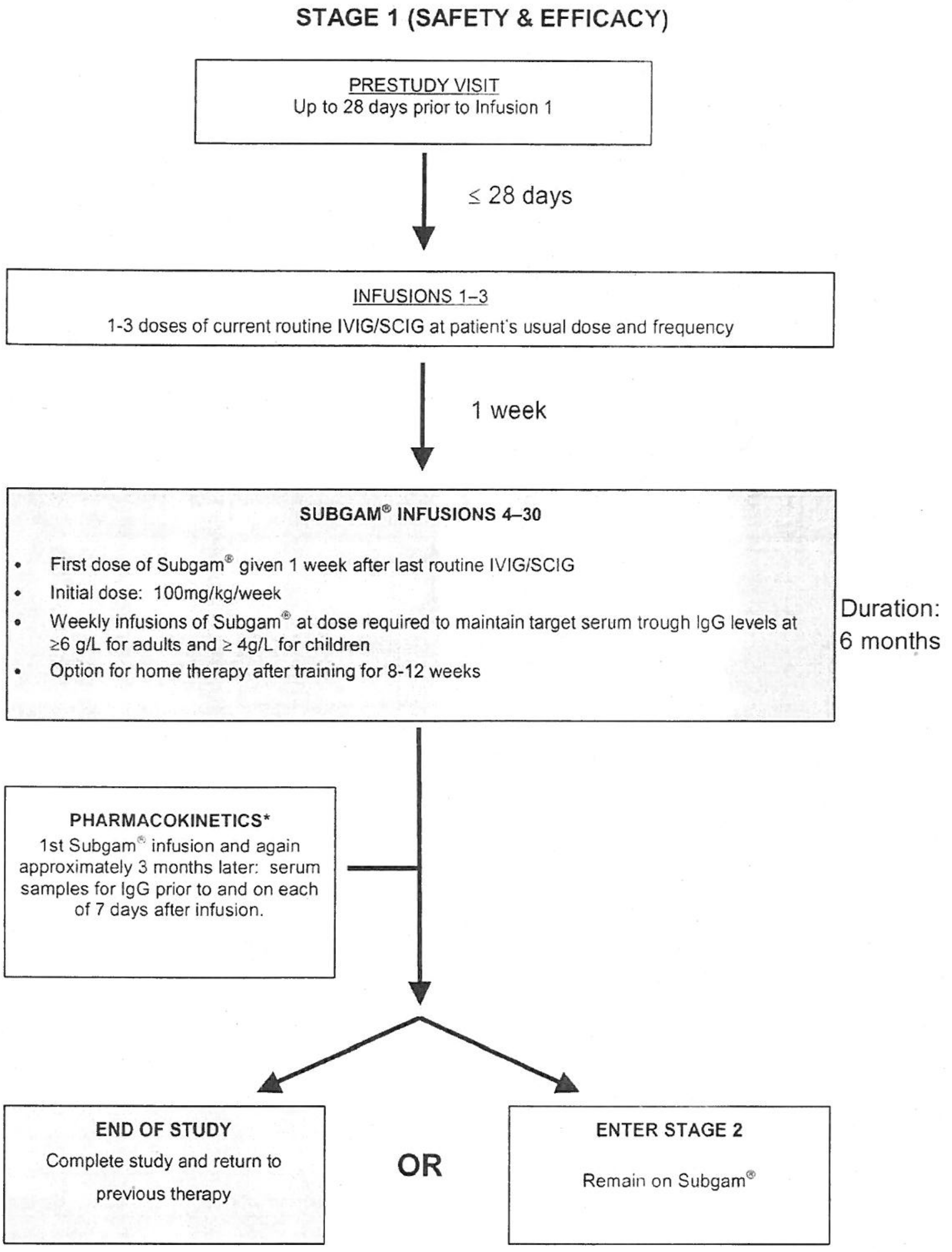

*Optional: only for patients who wished to take part.

Figure 2: Flow Chart (Stage 2)

STAGE 2 (LONG TERM SAFETY FOLLOW UP)

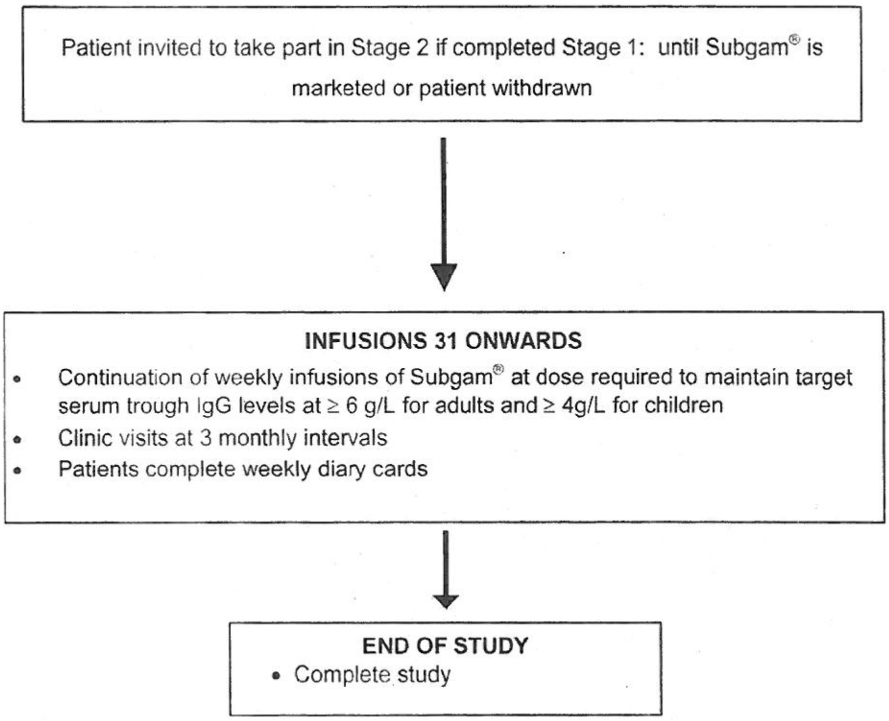

9.3 Selection of Study Population

It is regarded as good management of patients with primary antibody deficiency syndrome to minimise the number of blood products to which they are exposed. In general, patients only change product for good clinical reason or the route of administration for practical considerations. Therefore, investigators did not enrol all patients who met the entry criteria, but only those who needed a change of therapy according to these broad criteria. Nevertheless, among the study population there was a wide range of clinical diagnoses within the umbrella term of primary antibody deficiency syndrome. Thus, the study results are applicable to a range of patient types. No age range for patients was specified, as the study aimed to recruit both adults and children. Children have been included in the target population because they are expected to be the main beneficiaries of the subcutaneous route, as their veins tend to be less accessible. In addition, the mode of administration by the subcutaneous route gives more freedom of movement during infusions.

9.3.1 Inclusion Criteria

In order to be eligible for the study a patient was required to meet all of the following inclusion criteria:

1. *A diagnosis of primary antibody deficiency (e.g., congenital X-linked agammaglobulinaemia, common variable immunodeficiency (CVID), symptomatic IgG subclass deficiency).*

   This criterion was chosen in accordance with the 1997 WHO Primary Immunodeficiency Diseases report (WHO Scientific Group, 1997).

2. *Aged 0 to no upper age limit.*

   Primary antibody deficiency can be present at any age and always requires treatment. The mode of administration in this study was not expected to cause any systemic adverse events, e.g. thromboembolism, renal problems etc., and therefore no age restrictions were needed.

3. *Stable disease and receiving immunoglobulin (IVIG or SCIG) for the past 6 months.*

After 6 months of treatment, the patient's disease and immunoglobulin concentrations should be stable.

4. *Written informed consent.*

   This was a regulatory and ethical requirement.

9.3.2 Exclusion Criteria

If a patient met any of the following exclusion criteria they were considered ineligible for the study:

1. *Any patient known to be intolerant to IgA.*

   This criterion was to minimise the risk of a reaction to the small amount of IgA (<0.02% w/w) present in Subgam®.

2. *Any patient known to be pregnant or breast feeding at entry.*

   This was a 'generic' criterion that was used unless the aim of the study was for specific medication during pregnancy or the puerperium.

3. *Any patient with a history of clinically significant renal or hepatic disease or with known renal or hepatic abnormalities.*

   This was a 'generic' criterion that was used unless the specific aim of the study was to explore the product in patients with hepatic or renal failure. In addition, hepatic dysfunction might be caused by viral infection, which would reduce the opportunity of assessing viral safety of Subgam®.

4. *Any patient unsuitable for the purposes of the study (in the opinion of the investigator).*

   This criterion was to exclude patients for whom, in the investigator's opinion, Subgam® was not an appropriate treatment or those who were likely to be unreliable or non-compliant.

5. *Any patient participating in another clinical trial within the last 30 days of study entry.*

   This was a 'generic' criterion to minimise confusion about the interpretation of any adverse events or serious adverse events.

6. *Any patient who had a known history of allergic reactions to intravenous blood products.*

   Such patients may be at risk of suffering an adverse reaction to therapeutic IgG.

   To minimise the risk to patients of reacting non-specifically to Subgam®.

7. *Any patient with a known history of infection within the last 2 months, requiring IV antibiotics.*

   To exclude patients who might experience acute bacterial antigen/antibody reactions.

8. *Any patient known at entry to require any other blood product during the course of the study.*

   To minimise confusion as to the cause of any seroconversion to virus marker(s).

9.3.3 Removal of Patients from Therapy or Assessment

The clinical investigator could remove a patient if, in his/her opinion, it was in the best interest of the patient. A patient may have been withdrawn from the study for any of the following reasons:

- Withdrawal of consent - any patient could withdraw from the study at any time;
- Deviation from the protocol;
- Incidental illness;
- An adverse experience.

If there was a medical reason for withdrawal, the patient remained under the supervision of the investigator until in satisfactory health. After receiving the patient's consent, his/her general practitioner was informed of the patient's withdrawal from the study. Every effort was made to contact patients who failed to attend an appointment to ensure that they were in good health. In any eventuality, the investigator had to inform the Medical Department at BPL and record the withdrawal on the CRF.

9.4 Treatments

9.4.1 Treatments Administered

The investigative treatment was the subcutaneous immunoglobulin called Subgam®, manufactured by the sponsor at their facility in Elstree, UK. This product is prepared from human plasma. During the production of Subgam®, a solvent/detergent step is included which is recognised to be active against any lipid-enveloped viruses such as HIV, HBV and HCV.

The initial dosage was 100 mg/kg given subcutaneously at weekly intervals. The dose and frequency of dosing was adjusted, depending on the patient's clinical condition, to maintain a pre-infusion serum IgG level of at least 4 g/L for children and 6 g/L for adults, depending on patient needs. The treatment period with Subgam® during Stage 1 was six months (approx. 27 infusions). During Stage 2, treatment was to be continued until Subgam® was marketed in the UK.

9.4.2 Identity of Investigational Product

Subgam® (16% Immunoglobulin solution) is a sterile liquid immunoglobulin derived from human plasma collected from screened donors in the United States, the same plasma source that was used for the manufacture of BPL's licensed IVIG products, Vigam® Liquid and Vigam® -S. The methods of manufacture for Subgam® and Vigam® products are very similar.

Each batch of Subgam® solution contained the following ingredients (approximate concentrations):

Ingredient	Range (mg/mL)
Human protein (at least 95% IgG)	140–180
Sodium chloride	5.8–11.6
Glycine	3–5
Sodium acetate	1.2–2.0
Polysorbate 80	0.005–0.05

The above specifications provide ranges for the excipients that were not specified in the protocol.

Vials were stored in the pharmacy department at the investigator site until they were dispensed to study patients. Vials were stored away from light at 2–8°C, although short-term storage for up to 1 week at 25°C was permitted within the shelf-life period.

A total of 12 batches of Subgam® were used as follows:

SCBN276, SCBN276A, SCBN5303, SCBN5565, SCBN5679, SCBN5786, SCBN5922, SCBN6004, SCBN6077, SCBN6079, SCBN6205, SCBN6243. Batches SCBN276 and SCBN276A were manufactured as part of the same batch but were given different batch numbers as they were packaged and labelled in two separate lots.

Batch numbers received by each patient are listed in Appendix 16.1.6.

9.4.3 Method of Assigning Patients to Treatment Groups

As this was an open, non-comparative study, all patients were assigned to the same treatment and there was no randomisation code.

9.4.4 Selection of Doses in the Study

The initial dose (weekly infusions of 100 mg/kg body weight) and rate of infusion chosen were those used by Gardulf *et al.* (1991). The equivalent of 100 mg/kg body weight per week is a well-established standard starting dose when giving IVIG to patients with primary antibody deficiency syndrome. Thereafter, the dose and frequency of dosing could be changed by the investigator in order to maintain adequate serum IgG levels. Details of infusion rates are given below:

- For adults, an initial infusion rate of 10 mL/h was used, increasing over the next few infusions to a recommended maximum of 20 mL/h. Two body sites could be infused simultaneously (via 2 syringe drivers) giving a combined maximum rate of 40 mL/h.
- For children, an initial infusion rate of 5 mL/h was used, increasing over the next few infusions to a recommended maximum of 10 mL/h. Two body sites could be infused simultaneously (via 2 syringe drivers) giving a combined maximum rate of 20 mL/h.

Either the abdomen or the thigh could be used as the site of infusion for both adults and children. Most patients generally prefer abdominal siting for infusions, although very young children tolerate the infusions better in the thigh than in the abdomen (Gaspar *et al.*, 1998).

Self-infusion (home therapy) of SCIG was offered to suitable patients, following a period of training for the patient, parent or guardian, which took place over 8-12 weeks.

9.4.5 Selection and Timing of Dose for each Patient

The dose or frequency of dosing for individual patients could be modified by the investigator if adequate serum trough IgG concentrations were not maintained on the recommended dose (although weekly infusions were encouraged where possible). Patients could infuse at any convenient time of the day either before or after food.

9.4.6 Prior and Concomitant Medication

No concomitant medication was specifically contraindicated, although, as detailed in the protocol, immunoglobulins can reduce the immune system response to live virus vaccines, and attempts to vaccinate patients with primary antibody deficiency may not be completely effective. Of course, patients with primary immunodeficiency may not have a normal response to vaccination. All medications (prescription and over-the-counter) taken by the patient at the pre-study screening visit and during the course of the study were recorded in the CRF as concomitant medication.

Patients on home therapy recorded concomitant medications in the patient diary card. Diary entries were transcribed into the CRF by the Study Nurse at the patient's next visit.

All antibiotics, whether used for prophylaxis or treatment of an infection, were recorded as concomitant medication.

Patients had the option of using Emla® (lidocaine/prilocaine) cream or Ametop® (tetracaine) cream as local anaesthetic prior to infusion. Use of these was not recorded as concomitant medication.

9.4.7 Treatment Compliance

Infusions at the investigator site were prepared and supervised by the investigator or Nurse Specialist. The treatment given, including batch number and dose, were recorded in the patients' notes, nursing notes and study dosage booklet. Patients on home therapy infused themselves and recorded details (infusion site[s], batch number, dose and rate of infusion) in patient diary cards. The results of the concentrations of serum IgG (Appendix 16.2.6; Listing 16.2.6.1) were used to confirm overall compliance.

Drug accountability records were maintained in the pharmacy at each site to ensure that accurate records were kept of the total number of Subgam® vials dispatched, received and returned. For each patient the number of vials and batch number were recorded on a dispensing log. This was then cross-checked with entries in the patient notes, nursing notes or home care folder for each patient.

9.5 Efficacy and Safety Variables

9.5.1 Efficacy and Safety Variables Assessed and Flow Chart

The schedule of assessments carried out during the study is presented in Table 1.

Table 1: Schedule of Assessments

Assessment	Stage 1				Stage 2		Pharmacokinetics[9]	
	Routine IVIG/SCIG		Subgam®					
	Pre-Study	Infusions 1–3	Infusions 4–30	End of Stage 1 (EOS1)	3 Monthly	End of Study (EOS)	PK 1 After Infusion 4	PK 2 After ~ 3 Months on Subgam®
Informed Consent	X							
Inclusion /Exclusion Criteria	X							
Medical History	X							
Physical Examination	X			X				
Vital Signs	X	X	X	X		X	X	X
Patient Satisfaction Questionnaire	X		X (infusion 16)	X				
Site Inspection		X	X		X		X	X
Home therapy training			X					
Concomitant medications	X	X	X	X	X		X	X
Adverse event reporting	X	X	X	X	X		X	X
Haematology[1]	X	X[10]	X[10]	X	X[10]		X[10]	X[10]
Biochemistry[2]	X	X[10]	X[10]	X	X[10]		X[10]	X[10]
Blood Group Serology[3]	X							
Immunology: IgG, IgA, IgM	X	X	X	X	X[10]		X	X
Liver Function: ALT (and/or AST)	X	X[10]	X (every 4 weeks)	X	X[10]		X[10]	X[10]
Anti-pneumococcus & anti-HIB antibodies	X		X (every 4 weeks)					
Virology[4]			X (pre infusion 4)	X[5]	X[6]			
Parvovirus B19 (PCR)			X[7]					
Archive: Store at –70°C for 15 years			X (pre infusion 4)	X[5]	X[6]			
Repeat sample: Store at –20°C	X	X	X	X	X[8]		X	X

1 Haemoglobin, haematocrit, RBC, WBC, neutrophils, lymphocytes, monocytes, eosinophils, basophils, platelet count, reticulocytes;
2 Sodium, potassium, creatinine, LDH, GGT, total bilirubin, alkaline phosphatase, haptoglobin;
3 ABO (D) blood grouping system, Direct Coombs;
4 HBsAg, HIV (PCR) adults only, HCV (PCR);
5 Sample taken only if patient withdrew from Stage 1 or did **not** enter Stage 2;
6 Sample taken if patient withdrew or completed Stage 2;
7 Pre-first dose & 1 week post-first dose;
8 Taken only if required;
9 If patients gave consent;
10 If clinically indicated.

9.5.1.1 Efficacy Variables

Efficacy variables included:

Pre-Dose IgG Levels:

Samples were taken immediately prior to each weekly infusion where possible (although on occasions blood was taken up to 48 hours before infusion). For patients on home therapy, blood was collected approximately once every four weeks when the patient attended the hospital. This sampling occurred in Stage 1 only.

Inter-Infusion IgG Levels:

For those patients who consented to take part, blood samples for measurement of total serum IgG were taken once daily (except weekends) between two consecutive infusions on two separate occasions (once during the week of the first infusion with Subgam® and once more after the patient had been on study treatment for 3-4 months). On each occasion a blood sample was taken prior to infusion with study medication; then blood samples were taken on the following 7 days (except weekends). This sampling occurred in Stage 1 only.

Specific Antibodies:

Samples for assay of serum anti-pneumococcus and anti-HIB antibodies were taken at the pre-study screening visit and at 4-weekly intervals during Stage 1 only.

Dose Change:

The number and magnitude of changes in doses of Subgam® required to maintain target trough serum IgG levels at ≥ 4 g/L (children) and ≥ 6 g/L (adults).

Infections:

No pre-defined criteria (such as measures of C-reactive protein or bacterial cultures) were specified to clinically define infections. All types of infections (e.g. suspected or proven) were reported on the forms designed for AEs, and were coded as 'upper respiratory', 'lower respiratory', 'GI', 'skin' or 'other' following a manual review of the Higher Level Terms as defined in MedDRA v5.1.

Potential serious acute bacterial infections were identified as follows:

1. Adverse events were selected that EITHER:
   - Were classed as serious, in accordance with the ICH classifications for serious adverse events

   OR
   - Had a severity category of 'severe'
2. Of these, adverse events were selected that EITHER:
   - Contained one of the following 'keywords' in the 'Lower Level Term' in MedDRA v5.1:
     - Pneumonia
     - Bacteraemia
     - Sepsis
     - Osteomyelitis
     - Septic arthritis
     - Abscess
     - Meningitis

   OR
   - Coded to one of the following 'Higher Level Term' (HLT) or 'Preferred Term' (PT):
   - **HLT: Lower respiratory tract infection NEC**
     - PT: Bronchial infection
     - PT: Lower respiratory tract infection NOS
     - PT: Lung infection NOS.
   - **HLT: Sepsis, bacteraemia and viraemia**
     - PT: Amniotic infection syndrome of Blane
     - PT: Endotoxic shock
     - PT: Neutropenic shock
   - **HLT: Bone and joint infections**
     - PT: Arthritis infective NOS
     - PT: Bone infection NOS
     - PT: Bursitis infective NOS
     - PT: Infective chondritis
     - PT: Purulent synovitis

3. The CRFs for adverse events identified by points (1) and (2) above were then subject to medical review to assess whether they were classed as possible serious acute bacterial infections.

Further potential serious acute bacterial infections were identified by manual review of adverse event listings.

Antibiotics:

All antibiotics were reported as concomitant medications. Antibiotics were identified on review of the listings and were divided into the following categories:

- Long term prophylaxis – those used for >90% of study days in any study year
- Intravenous – those whose delivery was via the intravenous route
- Acute – those given orally for a maximum of 28 consecutive days
- Other – antibiotics not falling into any of the above categories

Days Off Work/School:

From infusion 5 onwards, patients were asked the question 'Have you had any days off work or school?' – either at the hospital visit if infusing at hospital, or in the patient diary card if infusing at home, and the number of days off work/school was recorded in the CRF or in the diary, as appropriate. Of the 50 patients in the ITT population, 15 did not attend either work or school, as detailed in filenote 001, Section 16.1.9, and were therefore excluded from this analysis.

Patient Satisfaction Questionnaire:

Patients were asked to complete satisfaction questionnaires at pre-study (Questionnaire 1), Infusion 16 (Questionnaire 2) and at the End of Stage 1 (Questionnaire 3). In the case of young children, the parent or guardian answered the questions.

In Questionnaire 1, patients were asked to select one option for each of the following questions:

- How comfortable do you find your current infusions? Extremely comfortable; very comfortable; quite comfortable; not very comfortable; extremely uncomfortable;

- How convenient do you find your current infusions? Very convenient; quite convenient; neither convenient nor inconvenient; quite inconvenient; very inconvenient;

In Questionnaires 2 and 3, patients were asked to select one option for each of the following questions:

- In general how does this subcutaneous treatment compare to your previous treatment? I like it much more; I like it more; I like it about the same; I don't like it as much; I don't like it at all;
- How comfortable do you find these subcutaneous infusions? Extremely comfortable; very comfortable; quite comfortable; not very comfortable; extremely uncomfortable;
- How convenient do you find this treatment? Very convenient; quite convenient; neither convenient nor inconvenient; quite inconvenient; very inconvenient;
- Compared with your previous treatment, how do you think your symptoms have been on this subcutaneous treatment? Much better; better; about the same; worse; much worse.

Home Therapy Training and Compliance:

Suitable patients were given the option of learning to self-infuse, if accompanied by a responsible adult who had also been trained in the procedure. The protocol suggested a minimum training period of 8-12 weeks, but this varied according to the competence of each patient. The time taken to train each patient and the number of weeks spent on home therapy in Stage 1 were documented. Compliance during home therapy was also noted.

9.5.1.2 Safety Variables

Safety variables were vital signs, adverse events, haematology and biochemistry, liver function, virology, physical examination, local tolerability and concomitant medication.

Vital signs:

Vital signs comprised seated blood pressure, pulse rate, oral temperature and respiratory rate. These measurements were taken by the investigator or designee

as appropriate. For patients on home therapy, vital signs were measured only when the patient attended the hospital (approximately every 4 weeks). In addition, patients on home therapy were asked to measure their temperature prior to each home therapy infusion. Patients were instructed not to infuse if their body temperature fell outside a pre-determined range (set for each patient by the investigator).

Adverse Events:

Each patient recorded daily events on a patient diary sheet throughout the study, from pre-study screening until the end of study assessment. Patients recorded any adverse events/concomitant medication or unusual happenings between hospital visits in their diary cards and the data were transcribed into the CRF by the investigator or research nurse.

The intensity of each adverse event was assessed by the investigator or designee. The following guidelines were used to assess intensity:

Mild:	Awareness of signs or symptoms that are easily tolerated
Moderate:	Discomfort enough to cause interference with usual activity
Severe:	Incapacitating with inability to do usual work/activity

The investigator also assessed whether the adverse event was related to the study drug according to the following criteria (based on Karch & Lasagna, 1975).

Probable:	A reaction that follows a reasonable temporal sequence from administration of the drug and follows a known response pattern to the suspected drug. The reaction cannot reasonably be explained by the known characteristics of the patient's clinical state or other modes of therapy administered to the patient.
Possible:	Plausible temporal sequence. Follows a known response pattern to the suspected drug. The adverse event might have been produced by the patient's clinical state or other modes of therapy administered to the patient.
Unlikely:	The current state of knowledge indicates that a relationship is unlikely.
Unknown:	It is not possible to assign the adverse event to any of the categories.

Not related: In the opinion of the investigators the adverse event is unrelated to the study drug.

Adverse events (serious or non-serious) classed as probably or possibly related to the study medication were regarded as product related. All other adverse events were regarded as unrelated.

For each adverse event, the System Organ Class and Preferred Term were coded using MedDRA version 5.1.

Virology:

Samples for analysis of HBsAg, HCV PCR and HIV PCR (adults only) were collected pre-Infusion 4 and at the end of study/withdrawal visit. Samples taken at pre-Infusion 4 were only analysed if the end of study / withdrawal visit sample gave a positive result. Samples for analysis of Parvovirus B19 PCR were collected pre-Infusion 4 and 1 week after Infusion 4.

Virology samples were analysed by the Public Health Laboratory Service (PHLS), Colindale, London.

Physical Examination:

Patients were given a physical examination at the pre-study screen and at the end of Stage 1.

Haematology:

Blood samples for routine haematology were taken at the pre-study screen and at the end of Stage 1; additional samples were taken during the study only if clinically indicated. The following were measured: haemoglobin, haematocrit, red blood cells (RBC), white blood cells (WBC), neutrophils, lymphocytes, monocytes, eosinophils, basophils, platelets and reticulocytes. Blood samples for Direct Coombs' tests were taken at pre-study.

Biochemistry:

Blood samples for routine biochemistry were taken at the pre-study screen and at the End of Stage 1; additional samples were taken during the study only if clinically indicated. The following were measured: sodium, potassium, creatinine, LDH, GGT, total bilirubin, alkaline phosphatase and haptoglobin.

Liver Function Tests:

Blood samples for AST and/or ALT were taken every 4 weeks during Stage 1 of the study. Samples were taken during Stage 2 of the study only if clinically indicated.

Local Tolerability:

The infusion site was inspected at each hospital visit. Patients on home therapy were asked to record all infusion site reactions in the diary. All infusion site reactions are reported as AEs and are tabulated separately.

Concomitant Medication:

Patients were questioned about concomitant medication at each clinic visit. Patients on home therapy were also required to record concomitant medication on a patient diary card. Concomitant medications were transcribed into the CRF at clinic visits.

9.5.2 Drug Concentration Measurements

The serum concentrations of total IgG were measured in the local hospital laboratories by either immunoturbidimetry or rate nephelometry.

9.5.3 Appropriateness of Measurements

All the tests used standard methodology and, with the exception of those for virology markers, were carried out in the routine laboratory of each institution. Normal ranges for each analyte were obtained from each laboratory before the study started.

9.5.3.1 Primary Efficacy Variable

The primary efficacy variable, according to the initial protocol, was the proportion of trough levels at each time point where serum IgG was ≥ 4 g/L. The minimum target levels for children and adults were modified in Protocol Amendment 1 to 4 g/L and 6 g/L, respectively (see Section 9.8.1).

The trough levels of IgG are important as they correlate with clinical activity and protection from recurrent infections. It is well established that minimum trough levels required to maintain the health of primary antibody deficiency patients and minimise the risk of recurrent infections are 4-6 g/L (as recommended in

CPMP/388/95 Final, 1996; and CPMP/BPWG/388/95 revision 1, 2000). Below this level, there is an increased risk of infections. Furthermore, in the UK, the minimum trough levels required to maintain the health of paediatric and adult patients are generally regarded as 4 and 6 g/L respectively.

9.6 Data Quality Assurance

Before the start of the study, all investigators and Nurse Specialists were invited to attend an Investigators' Meeting to discuss and finalise protocol design. The study staff at each investigator site, including laboratory and pharmacy staff, were given appropriate training by the responsible CRA on the principles of GCP, interpretation of the protocol, completion of the CRF, and the collecting, preparing and dispatching of blood samples to local and central laboratories. Representatives from each centre were also invited to attend a formal GCP training course run by Datawise Consultants Ltd (see Section 6 for details) and held at BPL.

During the study, the responsible CRA visited the sites regularly to ensure that: CRFs were completed accurately and CRF entries were consistent with source data; supplies were being stored and accounted for correctly; serum samples were being stored correctly; and that there were no problems with local laboratory tests.

Two of the study sites (Dudley Guest Hospital and St Helier Hospital, Carshalton) were audited by Datawise Consultants Ltd (see Section 6 for details). Copies of the Audit Certificates are presented in Appendix 16.1.8.

Data management was initially performed in-house at BPL. Data were double entered (by two individuals) into a database designed for the study in Clintrial Version 4.2 (Phase Forward Europe Ltd, UK); the database was then validated. Electronic and manual logistical checks were run on the data, and data discrepanices were resolved either in-house or by the study site. The system included an in-built audit trail of all changes made. The database was then locked for analysis.

The database was then transferred to the Constella Group Ltd (formerly Origin Pharmaceutical Services Ltd), Abingdon, Oxon, UK (Constella) for analysis and

reporting. Constella carried out further data management activities including: serious adverse event and adverse event reconciliation, serious adverse event and adverse event medical coding using MedDRA version 5.1, coding of concomitant medications using the WHO drugs dictionary, entry and quality check of normal ranges, and further general edits to the database after identifying a number of data discrepancies.

Tables, listings, figures and analyses were produced using SAS®. The data needed for pharmacokinetic analysis were manually transferred to WinNonLin® Professional version 4.1 (Pharsight Corporation, 5520 Dillard Road, Suite 210, Cary, North Carolina 27511, USA) and manually checked for accuracy before analysis.

A central laboratory was used to ensure consistency of certain sample analyses. The Central Public Health Laboratory in Colindale, London, UK carried out the PCR tests for Parvovirus B19, HIV-1 and HCV as well as the serology tests for HBsAg. Inter-laboratory standardisation methods and Quality Assurance procedures are given in Appendix 16.1.10.

9.7 Statistical Methods Planned in The Protocol and Determination of Sample Size

Details of the statistical analysis plan (dated 1 June 2006) are provided in Appendix 16.1.9.

9.7.1 Determination of Sample Size

No formal calculations were performed to determine the overall sample size. The overall total was based upon treating the minimum number of children (<12 years) in the study in accordance with draft CPMP SCIG guidelines (IVIG; CPMP/388/95, 1996 and, CPMP/BPWG/388/95 revision 1, 2000) (see Section 7). Fifty-one patients were recruited into the study; one patient (number 36) did not receive any study medication because he was withdrawn from the study shortly after being enrolled (see Section 9.7.3).

9.7.2 General Considerations

Continuous data such as age, height and weight are summarised by subgroup (diagnosis subgroup, age subgroup or prior therapy subgroup) using summary statistics (i.e., number of patients [n], mean, median, standard deviation, minimum and maximum). For all data except demographic data, 95% confidence intervals were calculated. Categorical data are summarised by dose cohort using frequency counts (with percentages).

Descriptive statistics for all endpoints that are continuous data have the following summary statistics in the following order: n, mean (1 decimal point [dp]), median (1 dp), standard deviation (2 dp), minimum (0 dp), maximum (0 dp), lower 95% CI (1 dp), upper 95% CI (1 dp). The number of dps shown are for raw continuous data with no decimal places. For raw data with more decimal places, the statistics are presented with the appropriate number of decimal places.

Where data are presented as 'total mean of means', this is calculated by calculating the mean value for each patient individually, and then taking the mean of all patients' mean values for the specified interval.

All percentages in summary tables are calculated out of the number of non-missing observations.

All analyses are descriptive. Unless otherwise stated, all testing was two-sided and performed at the 5% level of statistical significance.

9.7.3 Analysis Population

All patients who received at least one dose of study medication (i.e., Subgam® or the patient's previous treatment during Infusions 1-3) were included in the Intention-to-Treat (ITT) population. All safety and efficacy analyses were based on the ITT population.

One patient (number 36), who did not meet all the entry criteria, withdrew from the study prior to receiving Subgam®. As this protocol violation rendered this patient ineligible for the safety and efficacy analysis, this patient's data are listed separately from those of the other patients, under Section 16.2.3 (Listings for Patients Excluded from the Efficacy and Safety Analysis).

9.7.4 Subgroups

The following subgroups were derived for use in summary output:

Primary Antibody Deficient Diagnosis Subgroups:

- CVID & XLA
- Others

Age Subgroups:

- Adults (≥ 20 years)
- Teenagers (≥ 12 to <20 years)
- Children (< 12 years)

Age of the patient at the time of first dose of Subgam® was used in assigning the patient to an age subgroup. The interim study report described two subgroups of patients for most parameters: children (<12 years) and adults/teenagers (≥12 years). However, for satisfaction questionnaire and home therapy data only, the teenagers' subgroup (≥12 to <20 years) was also studied. The teenagers' subgroup was applied to all data in this study, as many clinicians have found this age group to present difficulties in compliance and attitudes.

Prior Therapy Subgroups:

- SCIG
- IVIG

Where more than one prior therapy during the 6 months prior to study entry was recorded, the most recent therapy was used in assigning the patient to a prior therapy subgroup.

Completers:

A subgroup of patients was used to compare changes in serum IgG levels and Subgam® usage across the duration of the study. This patient subgroup, termed 'completers', was identified on review of the study listings and met both of the following criteria:

- Compliance with protocol;

- Received Subgam® and provided regular serum IgG level data for a period of at least one year.

9.7.5 Demographics and Baseline Characteristics

All baseline summaries are presented for the ITT population. The demographic and baseline data summarised are: age, weight, height, body mass index (BMI), prior therapy (IVIG or SCIG), diagnosis, time from diagnosis to study entry, time from onset of PAD to study start, ABO blood group and RhD status, previous/present medical conditions, Coombs' test results and baseline serum IgG levels. Baseline serum IgG levels were calculated from the mean of the pre-Subgam® serum IgG levels at baseline (Infusions 1–3, when patients were on prior therapy). These consisted of 3 serum IgG values for most patients.

9.7.6 Pharmacokinetic Data

Inter-infusion IgG levels were measured in a subgroup of patients who attended the hospital daily for blood sampling up to a week after the first Subgam® infusion and after an infusion approximately 3 months later. Pre-infusion IgG levels (i.e., immediately prior to infusion of Subgam®) and daily post-infusion levels are displayed for each patient and with summary statistics at both time-points.

9.7.7 Efficacy

9.7.7.1 Efficacy Endpoints

The primary endpoints (efficacy) were:

- The proportion of trough levels at each time-point where the IgG ≥ 4 g/L for children and ≥ 6 g/L for adults;
- The change in Subgam® dose required in order to maintain trough levels at a minimum of 4 g/L for children and 6 g/L for adults. The number of occasions when the dose was adjusted, the size of the dose change and the overall range of dose changes;
- The time taken for each patient to reach a steady state IgG trough level. This was defined as when three consecutive occasions occur when the IgG trough levels were within 1 g/L of each other;

- The mean change in IgG trough levels as compared to the baseline level at each time-point;
- The incidence of infections reported throughout the study;
- The number of days off work/school.

Other endpoints that are considered measures of efficacy of Subgam® but were not defined as such in the protocol, were as follows:

- The use and duration of antibiotics used to treat infections;
- The number of days spent on home therapy;
- Patient perception of using Subgam® compared to their previous IgG therapy (see Section 9.8.3);
- Changes in levels of anti-HIB antibodies [changes in levels of anti-pneumococcus antibodies could not be assessed due to the variety of different methods of assay used by local laboratories, but all results can be found in Listing 16.2.6.4];
- Changes in levels of IgA and IgM.

9.7.7.2 General Considerations

For serum IgG levels, separate analyses were performed for the different stages of the study, as the number of data-points varied throughout the study.

In most cases, the first infusion of Subgam® was preceded by monitoring three infusions of the patients' previous medication (SCIG or IVIG). However, for two patients, more than three infusions of the previous medication were recorded due to logistical reasons at the investigational site, giving rise to infusion numbers 3A and 3B. Five patients also received fewer than the specified three infusions; four patients (Patients 1, 81, 82 and 83) received two infusions and one patient (Patient 13) received just one infusion.

For most patients, the expected duration of Stage 1 was 6 months from the date of Infusion 4. However, due to variations between patients in the intervals between infusions, the duration of Stage 1 varied between patients, and in some patients lasted beyond 6 months after Infusion 4. Therefore the duration of the

intervals entitled '0-6 months' and '>6 to 12 months' in tables and figures varied by patient. All subsequent intervals were exactly 168 days in duration.

Due to patients' holidays and other absences, not all infusions were administered regularly on a weekly basis, especially during Stage 2. This resulted in some variation between patients in the timing of study measurements and hospital visits in both Stage 1 and Stage 2. Moreover, as patients progressed to home therapy, a disparity arose between the number of infusions and the number of serum IgG levels (which were only measured during hospital visits). For this reason, tables comparing the serum IgG levels by infusion number have varying numbers of data-points around the time of each infusion, depending on the number of patients attending the hospital for that infusion. Furthermore, in Stage 2, serum IgG levels were collected retrospectively from patient notes and therefore, cannot be confirmed as immediately preceding a Subgam® infusion. To allow for these disparities when comparing serum IgG levels from the different patient subgroups across the whole study, the mean serum IgG levels within monthly (for Stage 1) and 6-monthly (for the whole study) time intervals were compared. Nevertheless, the descriptive statistics are likely to be representative of the achieved IgG levels because levels between infusions do not vary much, unlike after IV administration (see later).

The number of patients with analysable IgG data decreased as the study progressed since patients were recruited over a period of 22 months and participated in the study for varying lengths of time. Therefore, to allow an analysis of changes in serum IgG levels and Subgam® usage during the study, the patients who complied with the protocol and who provided regular serum IgG level data for a large portion of the study (at least one year) were analysed separately from the remaining patients. This subgroup of patients ('completers'; see Section 9.7.4) was identified on review of the data listings. This allowed comparisons of the same patients at different periods in the study.

During the early part of Stage 1, serum IgG levels were measured pre-infusion during the patients' weekly visits to the hospital. Serum IgG levels were therefore described as 'pre-infusion' or 'trough' levels for the interim analysis. During Stage 2, however, while patients were on home therapy, serum IgG levels were measured only during periodic hospital visits, and did not always have a

temporally corresponding Subgam® infusion. Therefore, for this final analysis, serum IgG levels are not described as 'pre-infusion' or 'trough'. An exception is made for the serum IgG value measured prior to the infusions used in the pharmacokinetic assessments, which are termed 'pre-infusion'.

Where tables split data by monthly or 6-monthly time periods, any percentages were calculated based on the number of observations during the appropriate time period.

9.7.7.3 Exclusion of Data from Analysis

Some data were excluded from the efficacy analysis where their inclusion was considered inappropriate. All data were displayed in the data listings, and all data were shown in the data tables (where displayed by patient number), but data excluded from the efficacy analysis were not used in the calculation of summary statistics or other analyses. Full details are provided in filenotes 001 and 002, Section 16.1.9. A summarised version of the exclusions and the reasons for them is presented here:

Time Taken to Reach Steady State (see Section 11.4.1.2)

The following patients' data were excluded for this analysis only: 01, 05, 06, 08, 12, 13, 25, 26, 27, 28, 43, 53, 57, 58, 61, 65, 73, 74, 75, 76, 77, 78, 81, 82, 83, 84, 85, 86. This is because these patients did not have a sufficient number of consecutive weekly serum IgG levels measured in Stage 1 to identify the first three consecutive weekly serum IgG levels within 1 g/L of one another. Usually this is because the patient progressed rapidly to home therapy, and did not visit the hospital site for every weekly infusion (see Table 2, which indicates the time taken to start home therapy).

Table 2: Time Taken to Start Home Therapy

Time Taken to Start of Home Therapy (days)	Number of Patients	Patient Numbers[1]
0 - 10	2	01, 28
11 - 20	5	25, 26, 27, 65, 86
21 - 30	8	06, 08, 58, 73, 74, 76, 84, 85
31 - 40	8	12, 41, 42, 43, 75, 81, 82, 83
41 - 50	7	13, 21, 23, 33, 34, 49, 78
51 - 60	5	09, 22, 35, 54, 77
61 - 70	6	05, 07, 11, 53, 57, 61
71 – 80	3	24, 55, 56
81 – 90	1	29
> 91	4	10 (93 days), 17 (204 days), 18 (112 days), 30 (117 days)

[1] Patient 31 did not go onto home therapy
Source data: Listing 16.2.6.10

Target Serum IgG Levels (see Section 11.4.1.1) and Serum IgG Levels (see Section 11.4.1.3).

Patient 42's data were excluded, as this patient was known to be non-compliant throughout the study. For other patients, data were excluded where the date of the serum IgG sample was after the date of the last recorded Subgam® dose in that patient. All post-infusion data (e.g. during pharmacokinetic assessments) were also excluded. Some patients received IVIG during the study, either as a 'top up' during a period of particularly poor health, or prior to and after a holiday. Any serum IgG values taken between the IVIG infusion and the next Subgam® infusion were excluded from the analysis.

Dose of Subgam® Across the Whole Study (see Section 11.4.1.5)

Patient 42's data were excluded, as this patient was known to be non-compliant throughout the study. Patients 54, 55 and 56 (all from the same centre, Oxford) started on low doses of Subgam® (32 mg/kg, 42 mg/kg and 32 mg/kg, respectively), rather than 100 mg/kg as recommended in the protocol. Doses were increased so that by the third Subgam® infusion (Infusion 6 of the study) all patients were receiving the recommended dose. However, this means that if the starting dose of Subgam® is used in Tables 14.2.6.5-14, the minimum and maximum dose as a percentage of the starting dose would be artificially high. For this reason, for these three patients only, the dose at Infusion 6 was used as the starting dose in Tables 14.2.6.5-14.

Changes in Subgam® Dose Across the Whole Study (see Section 11.4.1.5)

Patient 42's data were excluded, as this patient was known to be non-compliant throughout the study. Selected data for Patient 11 and all data for Patient 58 were excluded, because these patients received two doses, of different volume, of Subgam® a week, resulting in an inflated number of changes in the Subgam® dose. The changes in Subgam® dose across the whole study are presented in Tables 14.2.6.1 to 14.2.6.14.

Dose-Corrected Mean Serum IgG Levels (see Section 11.4.1.6)

Patient 42's data were excluded, as this patient was known to be non-compliant throughout the study. For other patients, data were excluded where the date of the serum IgG sample was after the date of the last recorded Subgam® dose in that patient. All post-infusion data (e.g. during pharmacokinetic assessments)

were also excluded. Some patients received IVIG during the study, either as a 'top up' during a period of particularly poor health, or prior to and after a holiday. Any serum IgG values taken between the IVIG infusion and the next Subgam® infusion were excluded from this analysis.

Total Subgam® Exposure (see Section 12.1.1) and Interval Between Infusions and 12.1.2)

Patient 42's data were excluded, as this patient was known to be non-compliant throughout the study. Otherwise, the numbers of days between consecutive infusions was calculated for the patients and summarised by descriptive statistics.

Specific Antibodies (see Section 11.4.1.11), Inter-Infusion IgG Levels (see Section 11.4.1.4) and Other Immunology Parameters (see Section 11.4.1.10)

Patient 42's data were excluded, as this patient was known to be non-compliant throughout the study. All post-infusion data (e.g. during pharmacokinetic assessments) were also excluded. For all other data, descriptive statistics were used where possible.

Time Off Work/School (see Section 11.4.1.12)

Data were excluded for 15 patients who were known not to attend work or school, often due to long-term sickness, incapacity or age. Patient 42's data were excluded, as this patient was known to be non-compliant throughout the study.

Time on Home Therapy (see Section 11.4.1.13)

Patient 31 withdrew from the study, as she had no one to help her with home therapy, therefore the data from this patient has been excluded.

9.7.8 Safety

9.7.8.1 Safety Endpoints

The safety and tolerability endpoints were:

- Adverse events, evaluation of the incidence and type reported throughout the study;
- Laboratory safety tests: haematology, clinical chemistry and virology;
- Vital signs.

Another endpoint that can be considered a measure of safety of Subgam® but was not defined as such in the protocol is evaluation of infusion site reactions (see Section 9.8.3).

9.7.8.2 General Considerations

The ITT patient population was used in the safety analyses. The number, type, severity and duration of adverse events excluding infections, together with the laboratory monitoring of haematology, blood biochemistry and virology markers, vital signs and infusion site reactions, were used to assess safety.

9.8 Changes in Study Conduct or Planned Analysis

9.8.1 Changes to the Protocol

Version 1 of the protocol (April 1999) was replaced by Version 2 (September 1999), which incorporated changes requested by the MREC; none of which affected the scientific rationale of the study.

Version 2 of the protocol was replaced by Version 3 (April 2000), which incorporated Amendment 1 to reflect a change in the minimum target trough serum IgG for children from 6 g/L to 4 g/L, as recommended by the Principal Investigator. The accepted target level in the UK for management of children with Primary Antibody Deficiency is ≥ 4 g/L.

Amendment 2 (25th July 2001) to Version 3 stated that there would be an increase in the total number of patients enrolled onto the study (from 40 to 50) so that a larger paediatric population might be studied (see Section 9.8.2).

Towards the end of the study it was noted that IgG data had not been specified to be collected prospectively during Stage 2; because it was expected that this would be done at intervals as a method of monitoring management. However, as the primary efficacy endpoint was the proportion of serum IgG levels ≥ 4 g/L for children and ≥ 6 g/L for adults, an amendment to the protocol (Amendment 3, dated 29th January 2004) allowed the retrospective collection of serum IgG levels during the period of the study from patients' notes. This amendment also allowed for collection of one additional blood sample at each patient's final study visit, for measurement of serum IgG levels.

The Final Protocol, version 3 dated April 2000 and incorporating Amendment 1 (dated 31st March 2000), can be seen in Appendix 16.1.1. This was the version available before any patient was enrolled into the study. Amendments 2 and 3 (dated 25th July 2001 and 29th January 2004 respectively) are also shown in Appendix 16.1.1.

9.8.2 Changes to Sample Size

The Final Protocol, April 2000 (see Appendix 16.1.1), stated that 40 patients would take part in the study. However, the proportion of paediatric patients recruited in this number was smaller than expected. In Amendment 2 to the protocol (25th July 2001) the sample size was increased to 50 to enable recruitment of additional patients aged under 12 years in order to fulfil the requirements of the draft CPMP guideline for SC and IM IgG (CPMP/BPWG/283/00) which was published during the conduct of the study.

9.8.3 Changes to Planned Analyses

The protocol stated that samples collected prior to Subgam® treatment for HBsAg, HIV and HCV testing would be assayed only if the corresponding sample collected at the end of the study was positive for any of these virology parameters. However, a number of pre-Subgam® samples for HBsAg, HIV and HCV were assayed on receipt at the central laboratory; these samples can be identified from Listing 16.2.8.4a (infusion number = INF4).

The study protocol listed patient perception of using Subgam® compared to their previous IgG therapy under safety measurements. However, the patients' assessments of how they liked Subgam®, their symptoms while on Subgam®, and the convenience and comfort of administering Subgam® are debatably more a measure of efficacy assessments, and are considered with other efficacy data.

Evaluation of infusion site reactions was assessed as a measure of safety of Subgam® although it was not defined as such in the protocol.

The definition of an "infection" was expanded beyond that in the SAP (see Filenote 19 October 2006). Some potential serious acute bacterial infections were identified by manual review of the adverse event listings although this was not specified in the protocol or statistical analysis plan.

10 Study Patients

10.1 Disposition of Patients

In total, 51 patients entered the study from 14 study centres in the UK. One patient with CVID (Patient 36) was withdrawn prior to receiving study medication. The patient should not have entered the study as he failed to meet the entry criteria (he had been diagnosed less than 6 months prior to the start of the study and had not received any IgG therapy during that time). Data from this patient is therefore not included in the rest of the report.

Table 3 shows the disposition of the 50 patients who received study medication and form the basis of this report.

Table 3: Patient Identification

Hospital	Number of Patients	Patient Numbers
Great Ormond St, London	6	81, 82, 83, 84, 85, 86
Papworth Hospital	6	21, 22, 23, 24, 77, 78
University Hospital of Wales, Cardiff	6	09, 10, 11, 12, 57, 58
Birmingham Children's Hospital	5	25, 26, 27, 28, 65
Barts & Royal London NHS Trust	4	05, 06, 07, 08
John Radcliffe Hospital, Oxford	4	53, 54, 55, 56
Leicester Royal Infirmary	4	73, 74, 75, 76
St Helier Hospital, Carshalton	4	33, 34, 35, 49
Dudley Guest Hospital	3	29, 30, 31
Hope Hospital, Salford	3	41, 42, 43
Northern General Hospital, Sheffield	2	17, 18
Royal Preston Hospital	1	61
Southampton General Hospital	1	13
St James' University Hospital, Leeds	1	01

Source: Table 14.1.2

The number of patients per centre ranged from 1 to 6. Table 14.1.3 presents the identification of patients enrolled at each hospital, by diagnosis.

Of the 50 patients who received study medication, 35 (70%) completed the study, and the other 15 patients withdrew prematurely (Table 4).

Table 4: Patient Disposition (All Patients)

	No. Patients					
	Adults (≥20 y) (n=28)		Teenagers (≥12-<20 y) (n=7)		Children (<12 y) (n=15)	
**Prior Therapy**	SCIG	IVIG	SCIG	IVIG	SCIG	IVIG
**Withdrawn from Study**	3	7	0	1	2	2
**Completed the Study**	3	15	1	5	5	6
**Total**	6	22	1	6	7	8

Source: Table 14.1.1

The reasons for withdrawal are shown in Appendix 16.2.9 (Listing 16.2.9.7) and were: patients' request (10 patients – Patients 9, 11, 12, 31, 33, 43, 55, 61, 75, 76), protocol violations (2 patients – Patients 42 and 65), investigator's request (1

patient [Patient 5] – to see if immunodeficiency in an infant had resolved) and other reasons (2 patients – Patient 26 returned to IVIG, and for Patient 53 data completion had become tedious after 995 days in the study). All patients with the exception of Patient 9 completed Stage 1 of the study (see Table 5 for duration of participation in study prior to withdrawal); 10 of the withdrawn patients completed more than one year.

Table 5: Reasons for Withdrawal from Study

Patient Number	Reason for Withdrawal	Number of Days in Study Before Withdrawal
5	Physician's request	569
9	Patient's request	225
11	Patient's request	1304
12	Patient's request	251
26	Other	928
31	Patient's request	288
33	Patient's request	1079
42	Protocol violation	1416
43	Patient's request	348
53	Other	995
55	Patient's request	1141
61	Patient's request	835
65	Protocol violation	824
75	Patient's request	945
76	Patient's request	211

Source data: Listing 16.2.1

The duration of patients' participations in the study is given in Table 6:

Table 6: Duration of Patients' Participation in the Study

	No. Patients			
	Adults	Teenagers	Children	Total
Stage 1, months 0 to 6[a]	28	7	15	50
Stage 2, months				
>6 to 12[b]	25	7	15	47
>12 to 18	23	7	15	45
>18 to 24	23	7	15	45
>24 to 30	23	7	14	44
>30 to 36	23	7	12	42
>36 to 42	21	6	5	32
>42 to 48	13	4	2	19
>48 to 54	5	3	0	8

a. This interval lasted approximately 6 months, depending on the number of infusions received by the patient during Stage 1, and contains post-Subgam® data only

b. This interval lasted approximately 6 months, from the first infusion in Stage 2 until 12 months after the first infusion of Subgam®

Source data: Table 14.2.5.6 (source table 14.2.5.6 exclude Patient 42, who was non-complaint). Data from Patient 42 are included in the above table.

10.2 Protocol Deviations

Protocol deviations are shown in Appendix 16.2.2 (Listing 16.2.2.1) and exclude elapsed days between infusions (Listing 16.2.2.2), and elapsed days between final infusion and end of study assessment (Listing 16.2.2.3). The majority of

deviations related to missing assessments or test results, or assessments not being carried out at within protocol-defined time intervals.

Nineteen patients (numbers 07, 08, 11, 13, 17, 28, 41, 53, 56, 58, 61, 73, 74, 75, 81, 82, 83, 84 and 86) received infusions from two separate batches of study medication on at least one occasion (see Listing 16.2.5.1), whereas the protocol recommended any infusion being from only a single batch.

Five patients received fewer than the specified three infusions of their previous medication prior to commencing Subgam® therapy. Four patients received two infusions (Patients 1, 81, 82 and 83) and one patient (Patient 13) received just one infusion.

One patient (number 36), who did not meet all the entry criteria, withdrew from the study prior to receiving Subgam®. The patient was withdrawn prior to receiving Infusion 1 of his current IVIG therapy. As this protocol violation rendered this patient ineligible for the safety and efficacy analysis, the patient's data are listed separately from those of the other patients (Listings 16.2.3.1–16.2.3.15).

Three patients (numbers 18, 34 and 74) were inadvertently given clinical trial product instead of licensed product after their end of the study visit (last dose of clinical trial product was given 4, 12 and 5 days after the end of study visit, respectively). For reporting purposes, they were considered as participating in the study until their last dose of clinical trial product.

11 Efficacy Evaluation

11.1 Data Sets Analysed

All efficacy evaluations were based on the ITT population. A total of 50 patients who enrolled in the study and received at least one dose of study medication i.e., Subgam® or the patient's previous treatment during Infusions 1-3, were included in the ITT population (see Listing 16.2.5.1).

The study population included a subgroup of 15 patients aged <12 years at enrolment into the study.

11.1.1 Completers Subgroup

The Completers Subgroup can be found in Table 14.2.2.3 and was identified on review of the data. This cohort was chosen to provide a high number of patients followed for a long period of time to allow evaluation of possible changes over time of dosage, IgG levels, tolerance *etc.*, as a within-patient comparison. The rationale for exclusion of patients from this subgroup is described in filenote 003, Section 16.1.9. The period of time up to and including >30 to 36 months in the study yielded data for a high number of patients (at least 29) for this subgroup (Table 14.2.2.3). Data from later time intervals for this cohort are also displayed for completeness, but patient numbers diminish, so within-patient comparison is not possible and these later data have to be recognised as unsuitable for comparative purposes.

11.2 Demographic and other Baseline Characteristics

11.2.1 Demography

An overview of demographic characteristics of the patients is provided in Table 7.

Table 7: Summary of Demography (at Enrolment)

	All (n=50)	Adults (≥20 y) (n=28)	Teenagers (≥12-<20 y) (n=7)	Children (<12 y) (n=15)
**Sex** Male/female N/N (%/%)	25/25 (50%/50%)	10/18 (36%/64%)	3/4 (43%/57%)	12/3 (80%/20%)
**Age (years)** Mean (Range)	29.5 (0.8-75.2)	45.5 (21.3-75.2)	15.2 (12.1-18.0)	6.2 (0.8-10.6)
**Height (cm)** Mean (Range)	148.6 (72-186)	163.9 (131-186)	161.4 (153-173)	113.9 (72-148)
**Weight (kg)** Mean (Range)	57.6 (10.1-132.7)	77.1 (46.0-132.7)	53.7 (37.2-70.1)	23.1 (10.1-48.2)

Source: Table 14.1.4

The patients' ages ranged from 0.8 to 75.2 years (mean 29.5). On enrolment to the study, of the 50 patients: 15 were children (<12 years of age, defined as up to their 12th birthday); 7 were teenagers (≥12 to 20 years of age, defined as from their 12th birthday up to their 20th birthday); and 28 were adults (≥20 years of age, defined as having reached their 20th birthday). There were an equal number of males and females. All patients, with the exception of one, were Caucasian (Listing 16.2.4.1).

Additional summaries of demographic data are presented in Table 14.1.5 (by specific diagnosis of primary antibody deficiency syndrome) and Table 14.1.6 (by prior therapy).

11.2.2 Primary Antibody Deficiency Disease Characteristics

All 50 patients had a diagnosis of primary antibody deficiency. The characteristics of primary antibody deficiency at baseline are presented by gender and age group in Table 8.

Table 8: Summary of Disease Characteristics: History of Primary Antibody Deficiency

Diagnosis	n	Adults (≥20 y) (n=28)		Teenagers (≥12-<20 y) (n=7)		Children (<12 y) (n=15)	
		Male	Female	Male	Female	Male	Female
**All Patients**	50	10	18	3	4	12	3
**CVID/XLA**	33	7	13	2	4	6	1
**CVID**	30	7	13	2	4	3	1
**XLA**	3	0	0	0	0	3	0
**Other**	17	3	5	1	0	6	2
**IgG Subclass Deficiency**	5	1	2	0	0	2	0
**Specific Antibody Deficiency**	7	1	3	1	0	1	1
**CD40 Ligand Deficiency**	2	0	0	0	0	2	0
**IgG Heavy Chain Deficiency**	1	1	0	0	0	0	0
**Ataxia Telangiectasia**	1	0	0	0	0	0	1
**Combined Immunodeficiency**	1	0	0	0	0	1	0

Source: Table 14.1.7

The majority of the patients in the study, including children <12 years and teenagers aged ≥12 to <20 years, had a diagnosis of CVID (30/50 [60%]), which is by far the most common form (60-70%) of primary immune deficiency.

The durations between onset of symptoms and start date and eventual diagnosis of primary antibody deficiency are summarised in Table 9.

Table 9: Time from Onset and Diagnosis of Primary Antibody Deficiency to Study Start by Age Group

	No. Patients		
	**Adults** (≥20 y) (n=28)	Teenagers (≥12-<20 y) (n=7)	**Children** (<12 y) (n=15)
**Diagnosis to Study Start (Years)**			
>0.5 to ≤1.0	2	0	3
>1.0 to ≤2.0	7	3	2
>2.0 to ≤3.0	2	0	2
>3.0 to ≤4.0	4	0	1
>4.0 to ≤5.0	4	0	4
>5.0 to ≤10.0	4	2	3
>10.0	5	2	0
Not known	0	0	0
**Onset of symptoms to study start (years)**			
>0.5 to ≤1.0	0	0	1
>1.0 to ≤2.0	0	0	0
>2.0 to ≤3.0	0	1	2
>3.0 to ≤4.0	2	0	3
>4.0 to ≤5.0	3	0	1
>5.0 to ≤10.0	5	1	6
>10.0	12	3	2
Not known	6	2	0

Source: Table 14.1.8 and Table 14.1.9

The study included both patients who had been suffering from the disease for some time and those that were recently diagnosed. However, the minimum time for diagnosis to study start was 6 months as this was a requirement for entry into the study.

The duration between diagnosis and study start date ranged from between 0.5 to one year (5 patients) to greater than ten years (7 patients), with about half (26/50) of the patients being diagnosed for no more than four years. The duration between disease onset and study start date was much longer, with the majority (29/42) of the patients with data having an onset date of at least five years before the start of the study. It is widely recognised that PAD patients often have clinical problems for many years before being diagnosed, and many of the patients in this study had symptoms for a considerable period of time before they were diagnosed.

11.2.3 Direct Coombs' Test and Blood Group Serology

Patients' ABO blood groups and Rhesus (RhD) status are given in Appendix 16.2.4 (Listing 16.2.4.6) and summarised in Table 10. The majority of patients were either A-positive (19/50) or O-positive (17/50). One patient (Patient 57) did not have a sample taken for blood typing.

Table 10: Summary of Blood Group Serology

ABO Blood Group	Rhesus status			
	Negative	Positive	Not known	Total
A	3	19	0	22
O	3	17	0	20
B	1	4	0	5
AB	1	1	0	2
Not known	0	0	1	1
Total	8	41	1	50

Source: Table 14.1.11

A sample for Direct Coombs' Test (DCT) was collected at the pre-study screening visit. DCT results are also listed in Appendix 16.2.4.6 and are summarised in Table 11. Samples for Direct Coombs' test were not taken for Patients 30, 31, 33, 34, 57, 73, 75 and 76 – a deviation from the protocol. Of the 42 patients who had the test, 6 patients had a positive result (Patients 11, 22, 54, 56, 74, 78). Five of the 6 patients with a positive pre-study DCT were on prior IVIG therapy; the exception was Patient 74 who was on prior SCIG therapy (see Table 14.1.12 and Listing 16.2.4.3). These results were not related to study treatment as all samples were taken before the patient started Subgam®; none of them were considered by the respective investigators to be clinically relevant. The protocol did not specify any follow-up samples.

Table 11: Summary of Coombs' Test Results by Gender

Sex	n	Negative	Positive	Not Done
Male	25	21	1	3
Female	25	15	5	5
Total	50	36	6	8

Source: Table 14.1.12

11.2.4 Prior and Current Medical Conditions

Patients' previous and current medical conditions are summarised in Table 14.1.13.

Many of the patients suffered from recurrent infections particularly Ear Nose and Throat (ENT) infections, other respiratory infections and gastrointestinal infections; these were probably related to the patients' underlying antibody deficiency. Additionally, several of the patients suffered from conditions such as bronchiectasis, sinusitis, lymphadenopathy and splenomegaly, which could have been induced by recurrent infections or occurred as a complication of their immunological state.

11.2.5 Prior IgG Therapy and Baseline Serum IgG Levels

Prior IgG treatment is shown in Appendix 16.2.4 (Listing 16.2.4.8) and summarised in Table 14.1.10 and Table 12 below. The majority (36/50) of the patients had received IVIG immediately prior to starting treatment with Subgam®. There was no difference in the proportion of patients receiving IVIG between subgroups of diagnosis of PAD; there was, however, a marked difference in the age subgroups. Children were equally likely to have received SCIG as IVIG, whereas teenagers and adults were far more likely to receive IVIG than SCIG. Baseline serum IgG was somewhat lower in children compared with teenagers or adults, which may reflect slightly lower IgG levels generally seen for young children compared to adults.

Overall, the mean of means pre-Subgam® IgG level (i.e. during Infusions 1 to 3) was 9.2 g/L (SD, 3.0; n=47; Table 14.2.1.7). Pre- Subgam® serum IgG levels (during Infusions 1 to 3) were somewhat higher in adults (mean 9.85 g/L) and teenagers (mean 12.80 g/L) compared with the children (mean 6.91 g/L), but this difference has not been tested for statistical significance.

Table 12: Prior IgG Therapy and Mean (Range) Baseline Serum IgG Levels (g/L) by Age Group and Prior Therapy (Infusions 1-3)

	No. Patients			
	All Patients (n=50)	Adults (≥20 y) (n=28)	Teenagers (≥12-<20 y) (n=7)	Children (<12 y) (n=15)
All Patients, n	50	28	7	15
IgG, mean (range), g/L	9.87 (2.96-18.68)	10.51 (5.82-18.68)	10.52 (7.30-12.97)	8.39 (2.96-11.70)
Prior SCIG, n	14	6	1	7
Home Therapy	10	4	1	5
Hospital Therapy	4	2	0	2
IgG, mean (range), g/L	8.59 (2.96-12.80)	9.85 (9.30-12.43)	12.80 (12.80-12.80)	6.91 (2.96- 9.70)
Prior IVIG, n	36	22	6	8
Home Therapy	7	4	3	0
Hospital Therapy	29	18	3	8
IgG, mean (range), g/L	10.37 (5.82-18.68)	10.69 (5.82-18.68)	10.14 (7.30-12.97)	9.68 (7.45-11.70)

Source: Table 14.1.10 and Table 14.1.14

11.3 Treatment Compliance

The majority of patients received an initial Subgam® starting dose of 100 mg/kg body weight as recommended by the protocol. Subsequent infusions were to be administered at weekly intervals, with the size and frequency of the dose adapted by the investigator to the individual patient in order to maintain adequate serum IgG levels and control of symptoms. There were, however, many cases in which infusions appeared to be given outside of a 4-9 day time-window (see Listing 16.2.2.2). Many of these cases may have been due to the loss or non-completion of diary cards, although in some cases, poor compliance with the prescribed dosing regimen was confirmed (for example, in the case of Patient 42).

Changes in Subgam® dose (mL) across the whole study are shown in Table 14.2.6.1. Of the 48 patients included in the analysis, 35 (73%) experienced dose increases of >1 mL at some point in the study, with a mean of 2.2 dose increases per patient (across the whole study). Also, 22 patients (46%) had their doses decreased by >1 mL, with a mean of 1.1 dose decreases per patient. Nineteen patients (40%) experienced both increased and decreased Subgam® dosages. Listing 16.2.5.1 displays the administration of Subgam® in more detail and displays dose changes in both mL and mg/kg.

There were no instances where increased dose led to withdrawal for reasons of an adverse event.

Full details of dosing deviations are provided as narratives in Appendix 16.2.5.

11.4 Efficacy Evaluation

11.4.1 Analysis of Efficacy

Full summary tables are available in Section 14.2.

11.4.1.1 Target Serum IgG Levels

The numbers of children (<12 years) and adults/teenagers (≥12 years) whose serum IgG levels fell below 4 g/L and 6 g/L, respectively, are summarised in Table 13 and Table 14, respectively.

Table 13: Number of Observations of <4 g/L Serum IgG levels and Patients who had at Least 1 Observation <4 g/L Serum IgG Levels Across the Whole Study - Children (<12 y)

	No. Observations		No. Patients (n=15)		
	n	IgG <4 g/L	n	IgG <4 g/L	Patient ID Number
Pre-Subgam®	21	1	11	1	57
Stage 1, months (0 to 6)[a]	122	3	15	1	57
Stage 2, months					
>6 to 12[b]	18	0	13	0	
>12 to 18	16	0	11	0	
>18 to 24	16	0	10	0	
>24 to 30	14	0	8	0	
>30 to 36	16	0	10	0	
>36 to 42	5	0	4	0	
>42 to 48	2	0	2	0	
>48 to 54	0	0	0	0	

a. This interval lasted approximately 6 months, depending on the number of infusions received by the patient during Stage 1, and contains post-Subgam® data only

b. This interval lasted approximately 6 months, from the first infusion in Stage 2 until 12 months after the first infusion of Subgam®

Source: Table 14.2.1.1, Table 14.2.2.1

During the pre-Subgam® treatment phase, target serum IgG levels of at least 4 g/L were met in 10 of the 11 children with data (91%) and in 20 of 21 observations (95%) in this patient subgroup. Likewise, during Stage 1 of the study, target serum IgG levels of at least 4 g/L were met in 14 of the 15 children

(93%) and in 119 of 122 observations (98%) in this patient subgroup. During Stage 1, there were three reports of serum IgG levels falling below 4 g/L, all in one patient, as follows:

- **Patient 57**, a male, 3 years old, with X-linked agammaglobulinaemia, previously on 2-weekly IVIG. This patient had very low serum IgG before starting Subgam® (trough levels were 2.53, 2.80, and 3.11 g/L immediately prior to Infusions 1, 3, and 4 respectively. He started on 8 mL Subgam® (81 mg/kg bodyweight) per week, which was increased at Infusion 9 to 10 mL (101 mg/kg bodyweight) and at Infusion 11 to 12 mL (120 mg/kg bodyweight). Trough levels slowly increased after the patient started on Subgam® and were 3.56 and 3.46 g/L prior to Infusions 7 and 11 respectively. By Infusion 16 (after 3 months on Subgam®), his serum IgG level had increased to greater than 6 g/L (7.02 g/L), though this sample was not considered an immediate pre-infusion value. Prior to Infusion 20, serum IgG levels were once again less than 6 g/L (5.47 g/L). However, IgG levels remained greater than or equal to 4 g/L (target level for children) from Infusion 16 until the end of the study, illustrating that good levels of IgG could be achieved with Subgam®. The patient remained in the study for 136 weeks in Stage 2.

During Stage 2 of the study, target serum IgG levels of ≥4 g/L were met in all children (Table 13).

During the pre- Subgam® treatment phase, target serum IgG levels of at least 6 g/L were met at all observations in 31 of the 34 teenagers and adults with data (91%) and in 70 of 75 observations (93%) in this patient subgroup. Likewise, during Stage 1 of the study, target serum IgG levels of ≥6 g/L were met at all observations in 29 of the 34 teenagers and adults (85%) and in 447 of 463 observations (97%) in this patient subgroup (Table 14).

Table 14: Number of Observations of <6 g/L Serum IgG levels and Patients who had at Least 1 Observation <6 g/L Serum IgG Levels Across the Whole Study - Teenagers and Adults (≥12 y)

	No. Observations		No. Patients (n=34)		
	n	IgG <6 g/L	n	IgG <6 g/L	Patient ID Number
Pre-Subgam®	75	5	34	3	17,18,34
Stage 1, months (0 to 6)[a]	463	16	34	5	10, 18, 31, 34, 84
Stage 2, months					
>6 to 12[b]	61	5	30	3	18, 30, 34
>12 to 18	61	5	27	4	11, 29, 30, 34
>18 to 24	64	8	27	4	11, 18, 29, 34
>24 to 30	58	8	28	4	10, 11, 30, 34
>30 to 36	46	2	26	2	11, 34
>36 to 42	32	7	21	4	10, 11, 29, 34
>42 to 48	22	3	16	3	10, 11, 34
>48 to 54	8	1	7	1	34
Total in Stage 2	352	39	30	6	10, 11, 18, 29, 30, 34

a. This interval lasted approximately 6 months, depending on the number of infusions received by the patient during Stage 1, and contains post-Subgam® data only

b. This interval lasted approximately 6 months, from the first infusion in Stage 2 until 12 months after the first infusion of Subgam®

Source: Table 14.2.1.1, Table 14.2.2.1

During Stage 2 of the study, target serum IgG levels of ≥6 g/L were met in 24 of the 30 teenagers and adults (80%) who had serum IgG levels measured during Stage 2, and in 313 of 352 observations (89%) in this patient subgroup.

The reports of serum IgG levels falling below the target level of 6 g/L were in 9 patients, as detailed below (based on data from Listings 16.2.5.1 and 16.2.6.1). (Patient 42, who was excluded from much of the analysis for poor compliance, has been included in this evaluation of IgG levels.)

- **Patient 10**, a female, 37 years old, with CVID, previously on 3-weekly IVIG. The patient remained on the same Subgam® dose (100 mg/kg) up to Infusion 39. The dose was increased at regular intervals from Infusion 40 to Infusion 202 (her last study infusion) with her final dose being 157 mg/kg. The patient's trough IgG levels fell below 6 g/L prior to Infusions 9, 10 and 11 when her serum IgG levels were 5.89, 5.26 and 5.55 g/L respectively. Her trough serum IgG level increased to 6.25 g/L at Infusion 12 but fell below 6 g/L again pre-Infusions 13 and 14 (5.51 and 5.82 g/L respectively), following a chest infection. Pre-Infusion 114, her serum trough IgG fell again to 5.92 g/L and again at Infusion 164 (5.5 g/L) and Infusion 170 (5.83 g/L).

Her IgG level on prior IVIG therapy was 7.22 g/L and at End of Study her IgG level was 7.63 g/L. Compliance was generally good in this patient, although there were occasions where her dosing interval was more than 10 days. The instances where her serum IgG levels were <6 g/L, in the main, were as a result of a dosing interval of more than 7 days between infusions.

- **Patient 11,** a female, 25 years old, with CVID, previously on 3-weekly IVIG. The patient commenced Subgam® treatment on a dose of 97 mg/kg, and remained on approximately the same dose until Infusion 87. The patient's dose then increased to 120 mg/kg at Infusion 88, following which there was considerable variation in dose levels, at times reaching as low as 60 mg/kg, before finally settling around 248 mg/kg at Infusion 196. Her final infusion (Infusion 222) was at a dose of 251 mg/kg. This patient became pregnant shortly after joining the study and was delivered of a healthy child by caesarean section shortly after Infusion 41. The patient's trough serum IgG level at Infusion 1 was 6.5 g/L and her trough IgG level remained >6 g/L throughout her pregnancy until pre-Infusion 73, when it fell to 5.02 g/L. Pre-Infusions 88, 98 and 120 the patient's serum trough IgG levels were 4.87 g/L, 5.17 g/L and 5.43 g/L respectively and her dose was subsequently increased. From Infusion 118, the patient tended to split her weekly dose over two infusions per week. The patient's serum trough IgG levels began to fall below 6 g/L again from Infusions 130 to 150 but her dose was not increased as a result; she simply returned to once weekly rather than twice weekly dosing. However, the patient did suffer a miscarriage during this time, shortly after Infusion 139. After Infusion 191, the patient's serum trough IgG levels fell below 6 g/L again (4.65 g/L) and her dose was increased at Infusion 196 to 248 mg/kg. Her serum trough IgG levels fell below 6 g/L several times further before the end of study assessment (4.4 g/L pre-Infusion 202, 4.0 g/L pre-Infusion 210, 4.17 g/L pre-Infusion 215, 4.31 g/L pre-Infusion 218). The patient's dose was not increased as a result but during this period the patient suffered increasing numbers of infections. This patient was withdrawn from the study, at her request, after 222 infusions because of her continued decrease in serum trough IgG levels and increased infections. Generally compliance was good in this patient.

- **Patient 17,** a male, 75 years old, with IgG Heavy Chain deficiency previously on IVIG. The patient commenced Subgam® treatment at a dose of 101 mg/kg which increased to 102 mg/kg during the study, but returned to 101 mg/kg by the end of the study. The patient completed the study having received 185 infusions of Subgam® during his approximately 3.5 years participation. The patient only showed a drop in serum IgG <6 g/L in the pre-Subgam® stage of the study. As there were a number of different batches of Subgam® used throughout Stages 1 and 2, this may account for the small variation in dose seen in this patient.
- **Patient 18**, a female, 22 years old, with CVID, previously on 3-weekly IVIG. The patient commenced Subgam® treatment on a dose of 101 mg/kg, which increased at intervals throughout the study to 109 mg/kg at her final infusion. The patient received 180 Subgam® infusions. Her pre-study serum trough IgG level was 5.4 g/L, which had increased to 7.29 g/L pre-Infusion 4 (first Subgam® infusion). Pre-Infusion 10 her serum trough IgG level fell to 5.7 g/L; her dose was marginally increased from 102 to 103 mg/kg but her serum trough IgG level remained below 6 g/L until pre-Infusion 13. The patient's serum trough IgG levels fell below 6 g/L again pre-Infusions 18 to 20 (5.78 g/L, 5.7 g/L and 5.59 g/L) and her dose was marginally increased again at Infusion 23 to 105 mg/kg. This patient's serum trough IgG levels fell below 6 g/L on a further five occasions; pre-Infusion 28 (5.8 g/L), pre-Infusion 31 (4.64 g/L), pre-Infusion 38 (5.61 g/L), pre-Infusion 52 (4.99 g/L) and pre-Infusion 90 (5.38 g/L), but otherwise remained above 6 g/L for the remainder of the study (Infusion 183). The patient's compliance was good and she maintained her weekly dosing interval throughout the study, with little fluctuation. Although the patient suffered a number of infections, only one coincided with a fall in her serum trough IgG (post Infusion 38).
- **Patient 29**, a male, 50 years old, with CVID, previously on 4-weekly IVIG. The patient commenced Subgam® treatment at a dose of 103 mg/kg, which was decreased during his first six months on treatment to 77 mg/kg and which he remained on until his last infusion. The patient received 194 Subgam® infusions. The patient's serum trough IgG levels were maintained above 6 g/L for the whole study, with the exception of four instances. These

were pre-Infusion 73 (serum trough IgG 5.3 g/L), pre-Infusion 80 (serum trough IgG 5.4 g/L), pre-Infusion 86 (serum trough IgG 5.2 g/L) and pre-Infusion 154 (serum trough IgG 5.3 g/L). The patient's dose was not increased as a result of any of these serum trough IgG levels nor was the patient's compliance erratic at these times, nor did the patient suffer an increase in the number of infections. In general the patient maintained on weekly dosing throughout the whole study period.

- **Patient 30**, a female, 12 years old, with CVID, previously on 4-weekly IVIG. The patient commenced Subgam® treatment at a dose of 106 mg/kg, which was decreased to 82 mg/kg during her first six months on treatment but was subsequently increased at intervals to 123 mg/kg at the End of Study. The patient received 192 infusions with Subgam®. The patient's serum trough IgG levels were maintained above 6 g/L throughout the study with the exception of six instances. Pre-Infusion 40, 48 and 55 her serum trough IgG level fell to 5.7 g/L, 5.8 g/L and 4.9 g/L respectively. Her dose was subsequently increased from 82 to 103 mg/kg. Pre-Infusion 99 and 114 her serum trough IgG levels fell again to 4.7 g/L and 5.1 g/L respectively and her dose increased again 123 mg/kg. The patient's compliance was generally good, with infusions being maintained more or less at weekly intervals. At the times of serum trough IgG level decreases, infusions had been given at weekly intervals but the patient did have a concurrent infection. At the patient's end of study visit her serum trough IgG level was noted to be 5.6 g/L, although her last recorded study dose of Subgam® was some six months before this visit. There is no information as to what treatment she had after her last recorded Subgam® study dose.

- **Patient 31**, a female, 55 years old, with CVID, previously on IVIG. The patient commenced Subgam® treatment at a dose of 92 mg/kg, which decreased to 60 mg/kg during her six months of treatment with Subgam®. The patient received 28 Subgam® infusions and withdrew after Stage 1 of the study, as she did not have anyone to assist her with home therapy. The patient's serum trough IgG levels were maintained above 6 g/L throughout the study with one exception where it fell to 5.4 g/L pre-Infusion 27. The patient's compliance was good, with seven-day intervals between infusions

being more or less maintained. However, it should be noted that the intervals between the two prior infusions to Infusion 27 were 9 and 8 days.

- **Patient 34**, a male, 43 years old, with CVID, previously on 3-weekly IVIG. The patient commenced Subgam® treatment at a dose of 105 mg/kg, which decreased at intervals to 95 mg/kg at End of Study. The patient received 122 Subgam® infusions. The patient had a low serum trough IgG level of 5.9 g/L pre-Infusion 1, which increased to 10.1 g/L pre-Infusion 4 (the first Subgam® infusion). His serum trough IgG levels were maintained above 6 g/L for almost all of Stage 1 of the study; his levels fell to 5.2 g/L and 5.4 g/L respectively at Infusion 29 and the end of Stage 1 assessment. During Stage 2 of the study, his serum trough IgG levels were again maintained above 6 g/L with the exception of 9 instances; pre-Infusion 39 (5.8 g/L), pre-Infusion 56 (4.3 g/L), pre-Infusion 62 (4.0 g/L), pre-Infusion 71 (5.9 g/L), pre-Infusion 74 (4.8 g/L), pre-Infusion 92 (4.0 g/L), pre-Infusion 103 (4.9 g/L), pre-Infusion 117 (5.8 g/L) and pre-Infusion 123 (5.4 g/L). This patient's compliance was not good, with frequent intervals of greater than 7 days between infusions. Some of the longer intervals have included for instance: 35 days between Infusions 31 and 32; 46 days between Infusions 54 and 55; 47 days between Infusions 61 and 62; 20 days between Infusion 102 and 103 and 23 days between Infusions 123 and 124.
- **Patient 42**, a male, 53 years old, with CVID, previously on 3-weekly IVIG. The patient commenced Subgam® treatment at a dose of 102 mg/kg, which was decreased to 97 mg/kg after six months treatment and then increased at intervals to 103 mg/kg. The patient received 99 Subgam® infusions. However, immediately prior to Infusions 22, 28, 91 and 102 trough serum IgG levels were <4 g/L (3.62, 3.40, 3.36 and 3.68 g/L respectively). The patient also had trough serum IgG levels <6 g/L prior to Infusions 10, 15, 20, 68 and 73 (5.16, 5.49, 4.12, 5.14 and 5.18 g/L respectively). This patient had a low IgG trough level on prior IVIG treatment (trough level at Infusion 1 was 5.85 g/L); in addition, he showed poor compliance while infusing with Subgam®. For example, the patient had not infused for 31, 17, 29, 36, 11, 16, 33 and 13 days prior to Infusions 10,15, 20, 22, 28, 68, 91 and 102 respectively. During the periods when the patient appeared to be infusing

more regularly, his IgG levels remained above 6 g/L (at around 8.53 – 9.21 g/L). This patient was eventually withdrawn from the study because of his non-compliance to study procedures. This patient was excluded from the data analysis presented in Table 14 because of his poor compliance (refer to section 9.7.7.3).

- **Patient 84**, a female, 13 years old, with CVID, previously on 3-weekly IVIG. This patient commenced Subgam® treatment at a dose of 95 mg/kg. The patient received 137 Subgam® infusions. The patient's serum trough IgG levels were maintained above 6 g/L for the duration of the study, with one exception, when it fell to 5.84 g/L pre-Infusion 24. Her dose was subsequently increased from 91 to 121 mg/kg although it was subsequently decreased at intervals to 100 mg/kg at study end. The patient's compliance was good, with seven-day intervals between infusions being more or less maintained throughout the study.

During this long-term study, a proportion of the adult/teenager patients did have serum IgG levels below the target value of 6 g/L. The above case narratives show that there was no systematic cause for these values. For some patients (e.g. numbers, 34 and 42), poor compliance was the most likely explanation. Several others (e.g. Patients 10 and 11) had infections at the time of their unusually low serum IgG levels, and it is feasible that these patients required a dosage increase to manage their clinical situation.

In conclusion, the high proportion of observations above the target minimum serum IgG levels in all age groups, and the low number of patients with serum IgG levels falling below these target levels, indicated that Subgam® was efficacious in the majority of cases.

11.4.1.2 Time Taken to Reach Steady State

The time taken for each patient to reach steady state serum IgG level, defined as three consecutive infusions when the serum IgG levels were within 1 g/L of each other, is summarised by prior therapy in Table 14.2.1.2. The mean time taken to reach steady state was 6.14 infusions (SD, 1.73; 95% CI: 5.37, 6.90; n=22) for patients who had received IVIG previously. There were only 4 patients included in the analysis of those who had received prior treatment with SCIG; this is

because the majority of such patients had progressed rapidly to home therapy and serum IgG levels were therefore not measured on a weekly basis prior to each infusion (see Section 9.7.7.3). The mean time to reach steady state for patients who had received prior treatment with SCIG was 6.50 infusions (SD, 1.29; 95% CI: 4.45, 8.55; n=4). Such a short time to reach steady state was not surprising as all patients on the study had at least 6 months prior treatment with IVIG or SCIG and would be expected to be at steady state before starting Subgam®.

11.4.1.3 Serum IgG Levels

Serum IgG levels (g/L) in Stage 1 of the study are listed by infusion number for all patients (Table 14.2.1.3), by diagnosis of PAD (Table 14.2.1.4), by age group (Table 14.2.1.5) and by prior therapy (Table 14.2.1.6). Mean serum IgG levels (g/L) in Stage 1 of the study are listed by month for all patients (Table 14.2.1.7), by diagnosis of PAD (Table 14.2.1.8), by age group (Table 14.2.1.10) and by prior therapy (Table 14.2.1.12).

The mean of individual mean serum IgG levels displayed in figures in this section for months 7 and 8 in Stage 1, and for the >42-48 months and >48-54 months time intervals in Stage 2, are unlikely to be representative of the whole study population, due to the small number of patients who had IgG values available at these time-points.

The mean of individual mean serum IgG levels are presented for Stage 1 of the study in Figure 14.2.1.11. During Stage 1 of the study, the mean level peaked at 10.4 g/L (SD, 3.0) during the first 4 weeks of treatment, and remained steady between 9.0 and 9.6 g/L for the following 6 months (Table 14.2.1.7). The mean level for all patients during Stage 1 as a whole was 9.75 g/L (SD, 2.69; Table 14.2.2.2).

The mean of individual mean serum IgG levels (g/L) according to diagnosis of PAD is presented graphically for Stage 1 of the study (Figure 3). During Subgam® treatment, the overall mean of means serum IgG level was maintained in the region of 8 to 10 g/L and close to the pre-Subgam® values. The overall mean of individual mean serum IgG level in Stage 1 was slightly lower in patients with CVID/XLA than those with other primary antibody deficiencies (9.24 g/L [SD,

2.10; n=32] and 10.70 g/L [SD, 3.42; n=17], respectively). This difference has not been tested for statistical significance. However, the difference was apparent in the pre-Subgam® phase of the study (see Figure 3) and is probably because the endogenous production of IgG is likely to be lower in patients with XLA and CVID than other diagnoses.

Figure 3: Mean of Means Serum IgG Levels in Stage 1, by Diagnosis of PAD

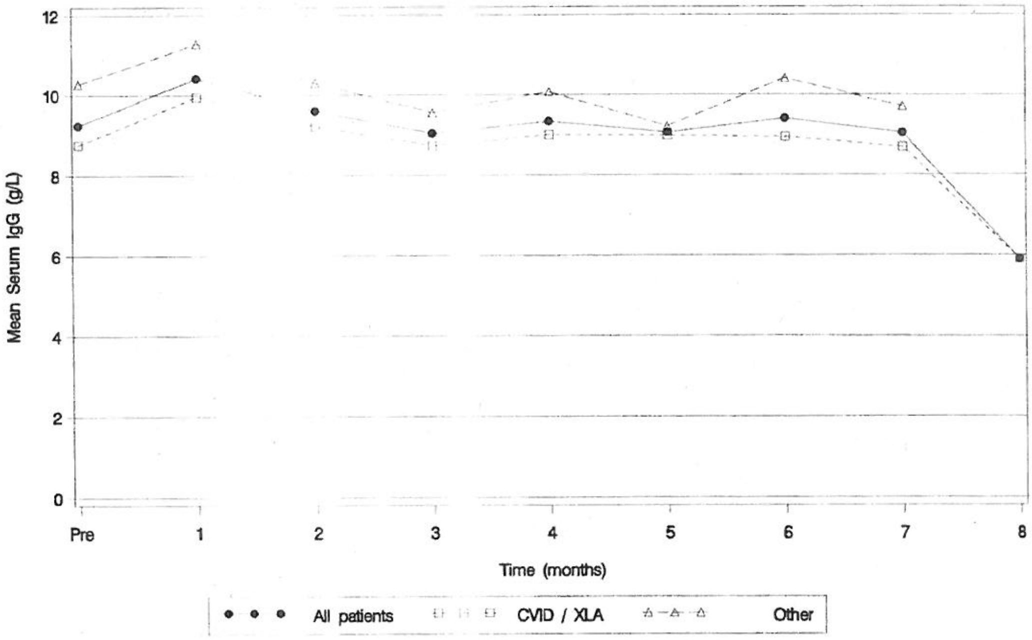

Program: T0339.SAS, Version: 8.2, Datetime: 23NOV2006:08:23

Certain data have been excluded from the efficacy analysis. See filenote 001, Section 16.1.9, for details of data exclusions from this figure

Source data: Figure 14.2.1.9

The mean of individual mean serum IgG levels (g/L) according to age sub-group is presented graphically for Stage 1 of the study (Figure 4). The overall mean of means serum IgG level in Stage 1 was slightly lower in children than those in teenagers and adults (8.48 g/L [SD, 2.05; n=15], 10.10 g/L [SD, 2.24; n=7] and 10.36 g/L [SD, 2.94; n=27], respectively) (Table 14.2.2.7). This difference, which has not been subject to any statistical testing, was expected, given the lower acceptable trough level (≥4 g/L) in children compared to teenagers and adults (≥6 g/L).

Figure 4: Mean of Means Serum IgG Levels in Stage 1, by Age Group

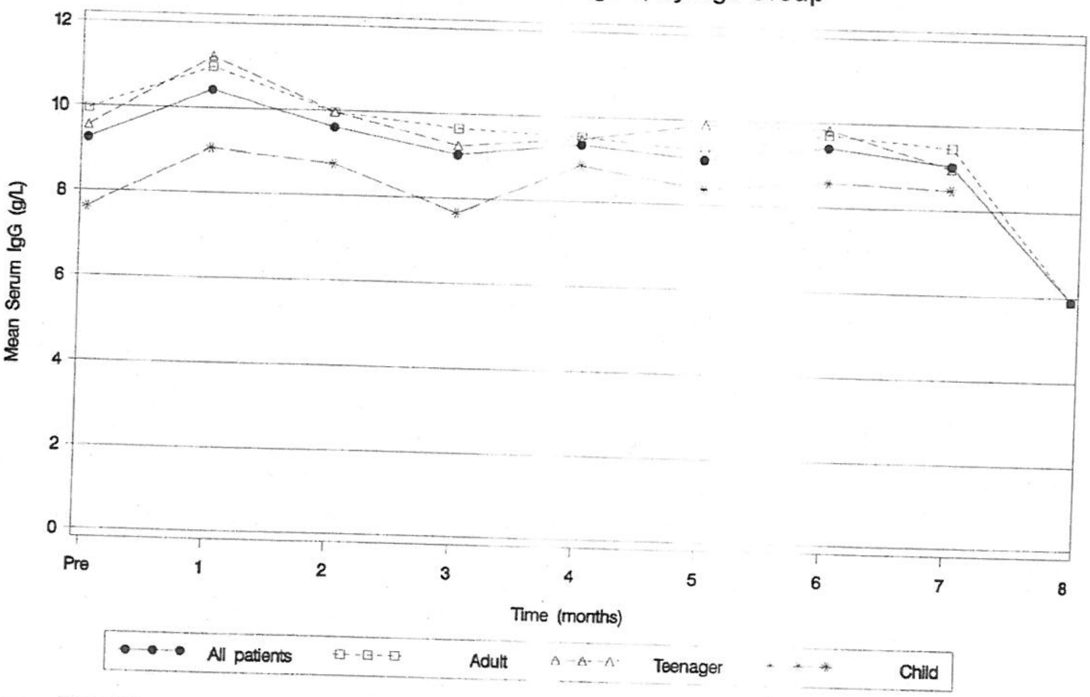

Program: T0340.SAS, Version: 8.2, Datetime: 23NOV2006:08:23

Certain data have been excluded from the efficacy analysis. See filenote 001, Section 16.1.9, for details of data exclusions from this figure

Source data: Figure 14.2.1.11

The mean of individual mean serum IgG levels in Stage 1 was higher in patients who had received IVIG previously than those who had received SCIG (10.17 g/L [SD, 2.81; n=35] and 8.68 g/L [SD, 2.10; n=14], respectively; Table 14.2.2.9). However, this can be attributed to a sizeable difference between serum IgG levels in the first month of Subgam® treatment, during which serum IgG levels were 8.5 g/L (SD, 2.6; n=14) and 11.2 g/L (SD, 2.9; n=35) for patients who had received SCIG previously and patients who had received IVIG previously, respectively (Table 14.2.1.12). From month 2 onwards, the mean of individual mean serum IgG levels in the two groups remained similar (Figure 14.2.1.13).

The mean serum IgG level was maintained above the pre-Subgam® IgG level of 9.2 g/L over the duration of the study (Table 15). Levels remained steady throughout the study, with the lower 95% confidence limit maintained above 6 g/L for all three age groups (children, teenager and adults) for the first 36 months on Subgam®.

Table 15: Mean Serum IgG Levels (g/L) by 6-Monthly Intervals

	Mean Serum IgG Levels (g/L) Treatment Period (Months)[a]						
	0 to 6[b]	>6 to 12[c]	>12 to 18	>18 to 24	>24 to 30	>30 to 36	>36 to 42
**All Patients**							
**n**	49	43	38	37	36	36	25
**Mean[d]**	9.75	9.43	9.30	9.77	9.61	9.52	9.26
**SD**	2.69	2.94	2.62	2.93	3.02	2.85	3.38
**Median**	9.21	9.30	8.66	9.77	9.38	9.15	8.50
**Min**	4.93	5.40	4.90	4.15	4.90	5.00	4.31
**Max**	19.40	18.47	17.10	16.60	17.30	17.35	16.20
**Lower 95%CI**	8.97	8.53	8.44	8.79	8.59	8.55	7.86
**Upper 95%CI**	10.52	10.33	10.16	10.74	10.64	10.48	10.65
**Adults**							
**n**	27	23	20	21	21	19	16
**Mean[d]**	10.36	10.11	9.93	10.01	9.95	10.14	9.62
**SD**	2.94	3.10	2.77	3.45	3.37	3.36	3.65
**Median**	9.54	9.67	10.28	10.30	10.08	10.00	10.45
**Min**	6.21	5.40	4.98	4.15	4.91	5.00	4.31
**Max**	19.40	18.47	17.10	16.60	17.30	17.35	16.20
**Lower 95%CI**	9.20	8.77	8.64	8.43	8.42	8.53	7.67
**Upper 95%CI**	11.52	11.45	11.23	11.58	11.48	11.76	11.57
**Teenagers**							
**n**	7	7	7	6	7	7	5
**Mean[d]**	10.10	9.89	9.66	10.37	10.03	9.88	10.25
**SD**	2.24	3.15	3.27	2.42	3.13	2.11	3.06
**Median**	9.21	9.47	8.90	10.47	11.15	9.83	8.86
**Min**	7.48	5.75	4.90	6.95	4.90	7.40	6.90
**Max**	12.98	13.90	14.08	13.00	13.55	12.30	13.60
**Lower 95%CI**	8.03	6.98	6.63	7.83	7.13	7.92	6.46
**Upper 95%CI**	12.17	12.80	12.69	12.92	12.93	11.83	14.05
**Children**							
**n**	15	13	11	10	8	10	4
**Mean[d]**	8.48	7.99	7.91	8.90	8.36	8.07	6.56
**SD**	2.05	2.09	1.15	1.82	1.62	1.67	0.66
**Median**	8.49	7.40	7.80	9.19	8.36	7.82	6.56
**Min**	4.93	5.47	6.28	6.40	6.35	5.59	5.83
**Max**	11.79	12.20	9.76	12.17	11.30	10.70	7.30
**Lower 95%CI**	7.34	6.73	7.14	7.60	7.01	6.88	5.51
**Upper 95%CI**	9.62	9.25	8.68	10.21	9.72	9.27	7.61

a. Table shows treatment periods with data from at least 25 patients (overall)
b. This interval lasts approx. 6 months, depending on no. infusions received by patient during Stage 1, it contains post-Subgam® data only
c. This interval lasts approximately 6 months, from the first infusion in Stage 2 until 12 months after the first infusion of Subgam® in Stage 1
d. Overall mean of individual mean
e. This table excludes data for Patient 42 due to non-compliance (see Section 9.7.7.3)

Source data: Table 14.2.2.2 and Table 14.2.2.7[e]

In the completers subgroup (see Section 9.7.4 for definition), the mean of individual mean serum IgG levels at >30 to 36 months of treatment (9.51 g/L; SD, 2.79; n=29) was 95.0% of the level at 0 to 6 months of treatment (10.01 g/L; SD, 2.59; n=32; Table 14.2.2.3). The means of individual means serum IgG levels (g/L) are presented graphically for all patients and completers in Figure 14.2.2.4. The IgG levels throughout the study for all patients and completers were very similar, indicating that although the completers subgroup was selected to exclude patients with a low number of datapoints or who deviated significantly from the protocol, the serum IgG values for the completers subgroup closely matched those for all patients. The apparent drop in IgG levels between months 7 to 8

(Figure 3 and Figure 4) represents data from just two patients, whereas for the other time periods the data is represented by between 17 and 32 patients.

As seen in Stage 1, the mean of individual mean serum IgG levels was consistently lower throughout the treatment period in patients with CVID/XLA than those with other primary antibody deficiencies (at >30 to 36 months: 9.09 g/L [SD, 2.66; n=24] and 10.37 g/L [SD, 3.15; n=12], respectively; Table 14.2.2.5). This is shown in Figure 5.

Figure 5: Mean of Means Serum IgG Levels in 6-Monthly Intervals up to 54 months, by Diagnosis of PAD

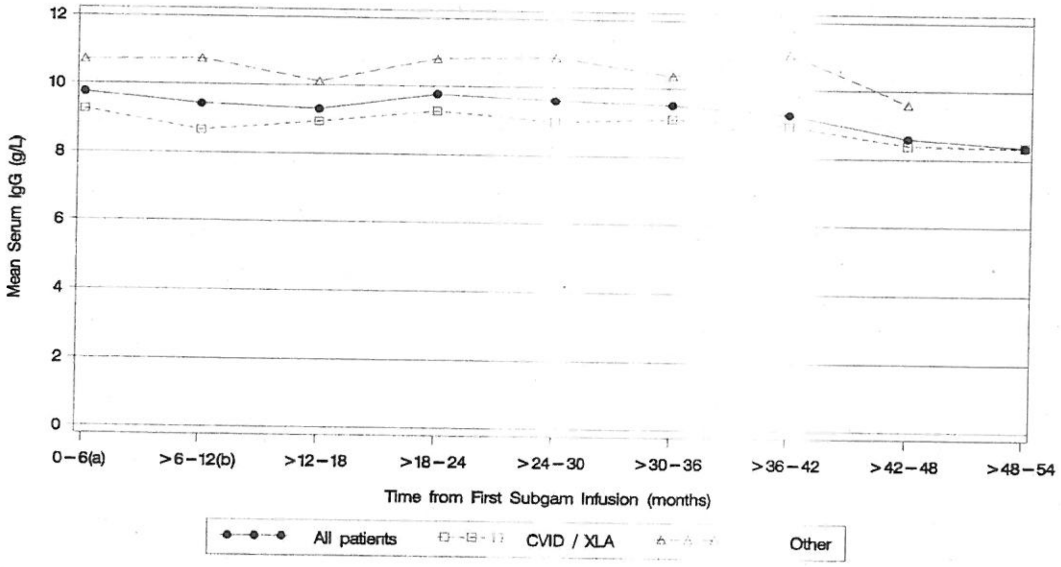

(a) This interval lasts approximately 6 months, depending on the number of infusions received by the patient during Stage 1
(b) This interval lasts approximately 6 months, from the first infusion in Stage 2 until 12 months after the first infusion of Subgam in Stage 1

Certain data have been excluded from the efficacy analysis. See filenote 001, Section 16.1.9, for details of data exclusions from this figure

Source: Figure 14.2.2.6

As seen in Stage 1, the mean of individual mean serum IgG levels was higher in adults and teenagers than in children at all time-points in the study (Table 15 and Figure 6).

Figure 6: Mean of Means Serum IgG Levels in 6-Monthly Intervals up to 54 months, by Age Group

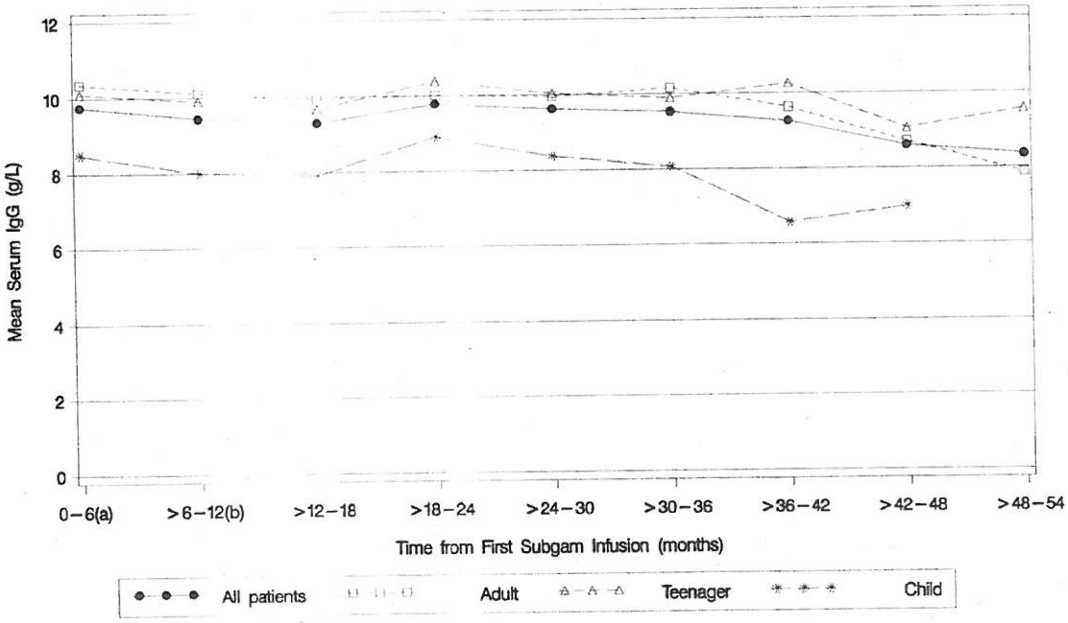

(a) This interval lasts approximately 6 months, depending on the number of infusions received by the patient during Stage 1, and contains post–Subgam data only
(b) This interval lasts approximately 6 months, from the first infusion in Stage 2 until 12 months after the first infusion of Subgam in Stage 1

Source data: Figure 14.2.2.8

Other than the differences in serum IgG in Stage 1 already noted, the mean of individual mean serum IgG levels for patients who had previously received IVIG and those who had previously received SCIG remained similar for the whole treatment period (Figure 14.2.2.10).

11.4.1.4 Inter-Infusion serum IgG Levels

Participation in this part of the study was optional, and most patients chose not to do so because of the inconvenience of visiting the hospital every weekday or because they were not prepared to give so many blood samples.

Eight patients (numbers 17, 21, 22, 23, 24, 29, 77 and 78) – all adults who were on IVIG therapy before starting the study – volunteered to have daily serum IgG measurements taken. Inter-infusion serum IgG levels for individual patients are given in Table 14.2.3.1; summarised data are shown in Table 16.

Table 16: Daily Inter-Infusion Total Serum IgG Values (g/L)

Time Period	Patient Number (a)	Serum IgG Level (g/L)							
		Pre-Infusion	Number of Days Post-Infusion						
			1	2	3	4	5	6	7
1st Subgam® Infusion	17 (1)	7.41	7.92	7.47	7.71	ND	ND	7.14	ND
	21 (1)	13.80	14.50	13.70	13.80	13.50	ND	ND	13.40
	22 (1)	17.80	17.50	16.90	ND	ND	15.70	ND	14.40
	23 (1)	13.70	13.50	ND	10.70	12.40	12.70	12.30	ND
	24 (1)	9.90	10.50	10.90	ND	9.60	ND	ND	9.20
	29 (1)	10.60	10.40	10.20	ND	ND	9.60	10.70	9.20
	77 (1)	16.30	17.00	ND	ND	ND	ND	15.20	15.50
	78 (1)	18.90	18.90	ND	ND	17.70	17.10	15.80	16.40
	n	8	8	5	4	4	4	5	6
	Mean	13.55	13.78	11.83	12.30	13.30	13.78	12.23	13.02
After Approximately 3 Months on Subgam®	17 (13)	7.60	8.90	7.51	8.00	ND	ND	7.97	ND
	21 (15)	9.70	10.50	11.10	11.00	10.50	ND	ND	ND
	22 (16)	3.90	ND	ND	ND	ND	11.10	10.10	10.00
	23 (13)	ND	12.50	ND	ND	12.10	12.90	11.50	ND
	24	ND	ND	ND	ND	ND	ND	ND	ND
	29 (15)	ND	ND	ND	ND	ND	7.50	7.50	6.60
	77 (13)	ND	ND	11.80	11.60	11.90	11.70	ND	ND
	78 (15)	13.60	13.80	ND	ND	14.20	14.50	13.60	ND
	n	4	4	3	3	4	5	5	2
	Mean	10.05	11.43	10.14	10.20	12.18	11.54	10.13	8.30

Source: Table 14.2.3.1
(a) Numbers in parenthesis are the numbers of Subgam® infusions prior to the start of the assessment.
ND = Not done

Serum IgG levels remained fairly stable with little daily fluctuation between infusions. There was no distinct peak in serum IgG levels after dosing, although mean levels on Days 4 and 5 were a little higher. The pattern after approximately 3 months on Subgam® appeared to be similar to that after the first dose of Subgam®, but the number of patients is too small to allow any firm conclusions to be drawn.

11.4.1.5 Subgam® Dose Analysis

Table 14.2.4.1 to Table 14.2.4.4 present summaries of Subgam® doses (mg/kg) received in Stage 1 of the study for all patients, and by PAD diagnosis, age group and prior therapy, respectively.

Table 14.2.5.1, Table 14.2.5.2, Table 14.2.5.4, Table 14.2.5.6 and Table 14.2.5.8 present summaries of Subgam® doses (mg/kg) received across the whole study for all patients and completers, and by PAD diagnosis, age group and prior therapy, respectively. The overall trends in dosage were similar whether considering all patients or completers (see section 9.7.4 for definition). In both

cases, there was a trend for the mean dose to increase gradually during the course of the study.

Overall, the mean of individual mean Subgam® doses increased from 104.6 mg/kg during the first 6 months, with the dose levelling off at around 115 mg/kg by >18 to 24 months (Table 17). The 95% confidence intervals for dosage remained relatively tight for the duration of the study, suggesting that the majority of patients were receiving doses close to the overall mean (Table 17). However, in both the children and adult sub-groups, the maximum mean Subgam® dose rose to over 200 mg/kg at some point, suggesting that in both sub-groups one patient at least was requiring doses that were much higher than the overall means. This is consistent with the fact that in both subgroups, the median of individual mean doses remained relatively stable over the duration of the study and was somewhat lower than the corresponding means. There was no evidence of any patient in the teenager subgroup requiring such dose increases; the highest maximum mean dose in this subgroup was 125.8 mg/kg (at >36 to 42 months) (see Table 17).

A similar slight increase in Subgam® dose over time was observed in the completers subgroup; the mean of individual mean doses during the first 6 months was 104.8 mg/kg (SD, 13.7; n=32), and the mean dose plateaued at around >18 to 24 months (117.0 mg/kg; SD, 29.4; n=32; Table 14.2.5.2). As with the data for all patients (see above), the median of individual mean doses showed very little change over the duration of study, whereas the maximum mean dose rose from 144.8 mg/kg at 0 to 6 months to 250.5 mg/kg at >42 to 48 months. Subgam® doses for all patients and for the completers subgroup are presented for the entire study period in Figure 14.2.5.3.

Table 17: Mean Subgam® Dose (mg/kg) by 6-Monthly Intervals

	Mean Subgam® dose (mg/kg) Treatment Period (Months)[a]						
	0 to 6[b]	>6 to 12[c]	>12 to 18	>18 to 24	>24 to 30	>30 to 36	>36 to 42
**All Patients**							
n	49	46	44	44	43	41	31
Mean[d]	104.6	109.7	114.6	116.1	113.5	117.6	116.0
SD	14.9	20.9	26.8	29.0	30.2	32.3	38.4
Median	101.8	103.3	103.0	103.9	102.2	107.9	106.2
Min	66.7	76.7	76.7	76.7	73.7	69.9	67.4
Max	149.5	159.1	194.2	185.3	213.8	227.6	230.3
Lower 95%CI	100.3	103.5	106.5	107.3	104.2	107.3	102.0
Upper 95%CI	108.8	115.9	122.8	124.9	122.8	127.8	130.1
**Adults**							
n	27	24	22	22	22	22	20
Mean[d]	105.5	113.5	118.0	118.2	112.2	119.1	126.8
SD	18.0	22.3	28.4	27.1	22.4	29.4	42.8
Median	101.8	105.3	105.5	106.7	106.9	108.8	109.1
Min	66.7	76.7	76.7	76.7	76.7	76.7	76.7
Max	149.5	152.7	194.2	182.5	157.6	199.0	230.3
Lower 95%CI	98.4	104.1	105.4	106.2	102.2	106.0	106.8
Upper 95%CI	112.6	122.9	130.5	130.2	122.1	132.1	146.8
**Teenagers**							
n	7	7	7	7	7	7	6
Mean[d]	100.7	101.7	102.8	100.4	99.8	102.9	103.7
SD	8.8	11.1	6.8	5.8	6.2	10.6	13.7
Median	100.6	104.5	100.1	101.1	102.1	101.2	103.3
Min	84.9	82.1	95.6	93.5	90.8	87.6	84.1
Max	114.4	118.0	112.6	108.3	106.8	119.3	125.8
Lower 95%CI	92.5	91.4	96.5	95.0	94.0	93.2	89.3
Upper 95%CI	108.8	111.9	109.0	105.8	105.5	112.7	118.1
**Children**							
n	15	15	15	15	14	12	5
Mean[d]	104.7	107.4	115.3	120.3	122.6	123.3	87.8
SD	10.8	21.7	29.8	36.2	44.0	43.8	17.1
Median	102.1	98.3	101.4	99.7	109.8	116.3	82.5
Min	92.1	88.1	83.0	78.1	73.7	69.9	67.4
Max	121.0	159.1	166.2	185.3	213.8	227.6	110.7
Lower 95%CI	98.7	95.4	98.7	100.3	97.2	95.5	66.7
Upper 95%CI	110.6	119.4	131.8	140.4	148.0	151.1	109.0

a. Table shows treatment periods with data from at least 25 patients (overall)
b. This interval lasts approx. 6 months, depending on no. infusions received by patient during Stage 1. It contains Subgam® data only
c. This interval lasts approximately 6 months, from the first infusion in Stage 2 until 12 months after the first infusion of Subgam® in Stage 1
d. Overall mean of individual mean
e. This table excludes data for Patient 42 due to non-compliance (see Section 9.7.7.3)

Source data: Table 14.2.5.1 and Table 14.2.5.6[e]

As discussed above, the trend in dose increases between the first 6 months and >18 to 24 months was observed in both the adults and children (Table 17 and Figure 7). No notable increase in mean dose with time was observed in teenagers.

Figure 7: Mean of Means of Subgam® Dosages (mg/kg) Received in 6-Monthly Intervals up to 54 months, by Age Group

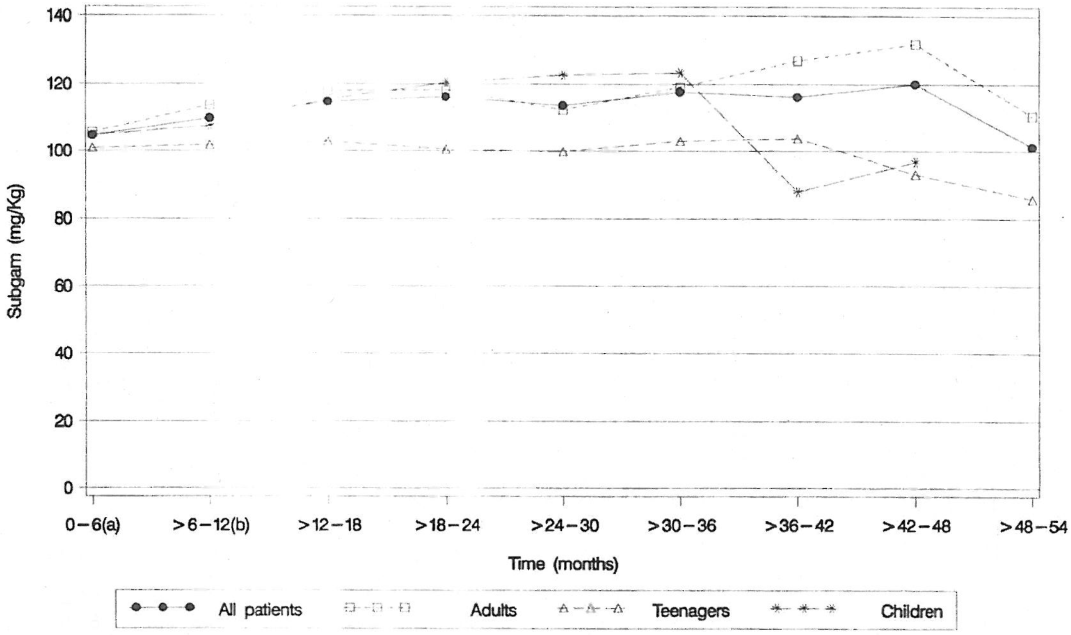

(a) This interval lasts approximately 6 months, depending on the number of infusions received by the patient during Stage 1, and contains post–Subgam data only
(b) This interval lasts approximately 6 months, from the first infusion in Stage 2 until 12 months after the first infusion of Subgam in Stage 1

Certain data have been excluded from the efficacy analysis. See filenote 001, Section 16.1.9, for details of data exclusions from this figure

Source: Figure 14.2.5.7

The trend in dose increases was observed in patients regardless of their diagnosis (Table 14.2.5.4), but interestingly was slightly more marked in patients who had received IVIG as prior therapy than those who were on SCIG prior to study start (Figure 8). Dosage remained relatively stable in the latter subgroup, possibly because patients on SCIG previously had been stabilised. Alternatively, it is feasible that patients previously on IVIG had somewhat more severe disease than those on prior SCIG and consequently required slightly higher doses of Subgam® after switching to this treatment modality.

Figure 8: 'Mean of Means' of Subgam® Dosage (mg/kg) Received in 6-Monthly Intervals up to 54 months, by Prior Therapy

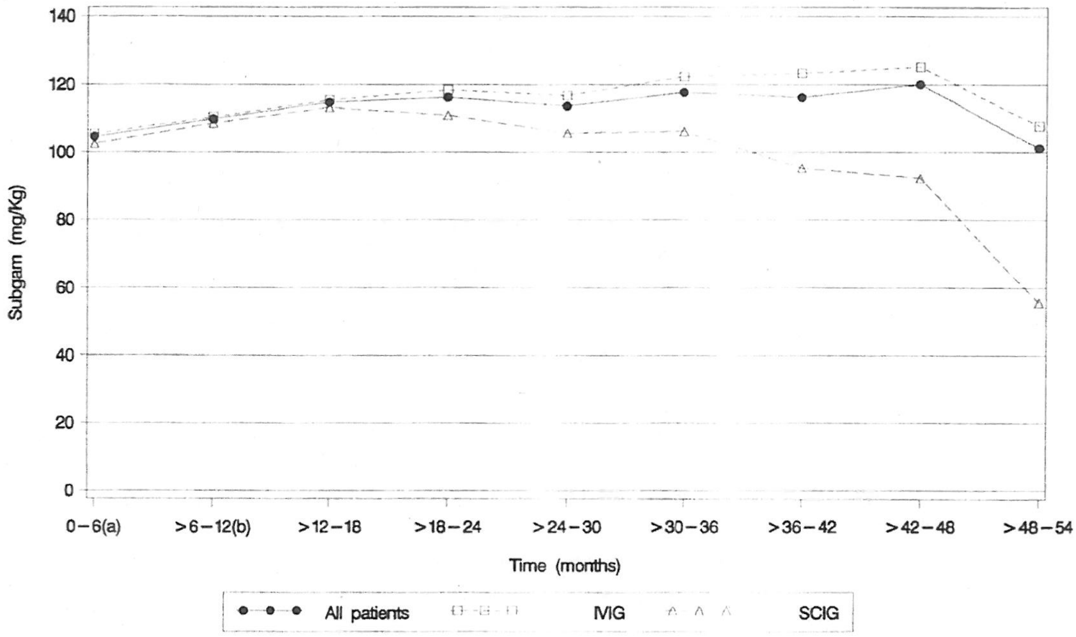

(a) This interval lasts approximately 6 months, depending on the number of infusions received by the patient during Stage 1, and contains post−Subgam data only
(b) This interval lasts approximately 6 months, from the first infusion in Stage 2 until 12 months after the first infusion of Subgam in Stage 1

Certain data have been excluded from the efficacy analysis. See filenote 001, Section 16.1.9, for details of data exclusions from this figure

Source: Figure 14.2.5.9

Changes in dose across the study were analysed in two ways: by presenting the number of dose increases and dose decreases of >1 mL (~160 mg) of Subgam®; and by examining the minimum and maximum Subgam® dose in a given time interval as a percentage of the starting dose.

Table 14.2.6.1 to Table 14.2.6.4 present summaries of the number of increases and decreases in Subgam® dose (mL) for all patients, and by PAD diagnosis, age group, and prior therapy, respectively. These are displayed in Table 18.

Table 18: Number of Increases and Decreases in Subgam® Dose (mL) Across the Whole Study

Patient Group		Number of Infusions Given	Number of Dose Increases > 1 mL	Number of Dose Decreases > 1mL
All Patients	n	48	48	48
	Mean	144.1	2.2	1.1
	Min	22.0	0.0	0.0
	Max	219.0	8.0	7.0
CVID/XLA	n	32	32	32
	Mean	154.2	2.3	1.3
	Min	27.0	0.0	0.0
	Max	219.0	8.0	7.0
Other	n	16	16	16
	Mean	124.1	2.1	0.7
	Min	22.0	0.0	0.0
	Max	185.0	6.0	5.0
Adult	n	26	26	26
	Mean	144.0	1.8	1.0
	Min	22.0	0.0	0.0
	Max	219.0	8.0	4.0
Teenager	n	7	7	7
	Mean	171.3	3.4	2.4
	Min	137.0	0.0	0.0
	Max	202.0	8.0	7.0
Child	n	15	15	15
	Mean	131.7	2.4	0.5
	Min	78.0	0.0	0.0
	Max	181.0	7.0	5.0
Prior IVIG	n	34	34	34
	Mean	147.1	2.3	1.2
	Min	22.0	0.0	0.0
	Max	219.0	8.0	7.0
Prior SCIG	n	14	14	14
	Mean	137.0	2.0	0.9
	Min	27.0	0.0	0.0
	Max	201.0	4.0	3.0

Source: Tables 14.2.6.1, 14.2.6.2, 14.2.6.3 and 14.2.6.4

Each patient experienced a mean of 2.2 dose increases of >1 mL (SD, 2.1, n=45, min=0, max=8) and a mean of 1.1 dose decreases of >1 mL (SD, 1.6, n=18, min=0, max=7) during a mean of 144.1 infusions throughout the study. This corresponds to one dose increase every 66 infusions and one dose decrease every 131 infusions. A slightly higher number of dose adjustments were made in teenagers and children (one dose increase every 50 infusions and every 55 infusions, respectively) than in adults (one dose increase every 80 infusions). This could be explained by the increase in body weight throughout the study in these patient subgroups, and hence the increase in doses required to maintain a stable dose per kg bodyweight.

Tables 14.2.6.5 to 14.2.6.14 present summaries of the minimum and maximum Subgam® dose as a percentage of the starting dose. In Table 14.2.6.5 to Table 14.2.6.9 minimum and maximum doses in Stage 1 and Stage 2 are displayed, showing summaries for all patients and completers subgroup, and by diagnosis of PAD, age group and prior therapy respectively. In Table 14.2.6.10 to Table 14.2.6.14 minimum and maximum doses in each 6-monthly interval are displayed, showing summaries for all patients and completers, and by subgroup diagnosis of PAD, age group and prior therapy respectively.

Summary statistics for the mean minimum and maximum doses for all patients as a percentage of the starting dose are displayed in Table 19. The high standard deviations seen in these data reflect the large variation in doses, particularly in the maximum doses. The mean minimum dose was 92.9% of the starting dose in Stage 1 and 93.3% in Stage 2, indicating very little change. The mean maximum dose was 115.0% of the starting dose in Stage 1 and 132.4% of the starting dose in Stage 2. The median maximum dose showed a less marked shift from Stage 1 (102.9% of starting dose) to Stage 2 (109.3% of starting dose). These data are consistent with the earlier statistics suggesting that a small proportion of patients increased their Subgam® dose during the course of the study.

Table 19: Minimum and Maximum Doses as a Percentage of the Starting Dose (All Patients)

	Minimum Dose as a % of Starting Dose (mg/kg)		Maximum Dose as a % of Starting Dose (mg/kg)	
	Stage 1	Stage 2	Stage 1	Stage 2
n	48	45	48	45
Mean	92.9	93.3	115.0	132.4
Median	97.6	94.2	102.9	109.3
SD	11.5	26.34	19.45	46.28
Min	58.6	37.4	100.0	74.5
Max	100.0	144.4	158.4	278.1
Lower 95% CI	89.52	85.43	109.39	118.53
Upper 95% CI	96.20	101.26	120.69	146.35

Source data: Table 14.2.6.5

A total of fourteen patients had a minimum dose of <50% of the starting dose or a maximum dose of >150% of the starting dose either in Stage 1 or Stage 2. Twelve patients had maximum doses of >150% of the starting dose, either in Stage 1 or in Stage 2. Of these, 4 patients had maximum doses of >200% of the starting dose. In contrast, only two patients had a minimum dose of <50% of the starting dose either in Stage 1 or Stage 2. Minimum and maximum doses of

<50% and >150% of the starting dose are displayed for these 14 patients in Table 20.

Three patients (all from the Oxford centre) started on low doses of Subgam® (see section 9.7.7.3 – Exclusion of Data from Analysis). To prevent the minimum and maximum dose as a percentage of baseline from being artificially high, the data for these patients has been excluded from Table 20. For these 3 patients only, the dose at Infusion 6 was used as the starting dose in Tables 14.2.6.5-14 and in Table 19 and Table 20.

Table 20: Minimum and Maximum Doses for Those Patients with a Minimum Subgam® Dose of <50% of Starting Dose or a Maximum Subgam® Dose of >150% of Starting Dose

	Minimum Dose as a % of Starting Dose (mg/kg)		Maximum Dose as a % of Starting Dose (mg/kg)	
Patient Number	Stage 1	Stage 2	Stage 1	Stage 2
08	100.0	107.2	129.5	192.6
10	99.9	99.9	100.0	156.6
11	93.7	61.6	100.0	258.3
22	100.0	110.3	130.4	233.5
24	100.0	133.6	137.7	151.4
27	96.1	120.0	132.9	159.6
34	58.6	37.4	100.0	96.2
53	100.0	101.8	158.4	152.6
57	99.1	134.0	146.8	169.3
77	100.0	144.4	144.4	172.6
81	100.0	143.8	153.5	278.1
82	98.4	116.9	158.3	227.0
83	93.2	101.9	132.2	179.6
86	58.7	41.8	100.0	120.4

Source data: Table 14.2.6.5

Narratives for the elevated doses highlighted in Table 20 are provided in Section 11.4.1.1. In some of these patients the changes in dose were of no clinical significance, whereas in other patients there were significant changes in dose with clinical explanations, e.g. the pregnancy in Patient 11 necessitated higher than usual Subgam® doses. Details of all infusions for the above patients are provided in Appendix 16.2.5 (Listing 16.2.5.1).

There were no differences between the subgroups in minimum and maximum doses as a percentage of the starting dose, with the exception of the teenagers subgroup, in which the mean minimum and maximum doses in Stage 2 were 73.3% and 119.4%, respectively (see Table 14.2.6.8).

For each patient the "target" number of infusions was calculated on the assumption that a patient had received regular weekly infusions as per the protocol. The actual number of Subgam® infusions as a percentage of this "target" is shown in Table 21 below. The mean percentage of the target number

of infusions was 100.6%, with quite tight 95% confidence intervals, suggesting that the majority of patients adhered quite closely to a 7-day dosing schedule. However, one patient (Patient number 34) received only 61.6% of the infusions that would be achieved with once-weekly dosing, suggesting consistently quite poor compliance (see Table 21). At the other extreme, one patient (Patient number 58) received 199.4% of the "target" number of infusions, suggesting that on average he received 2 doses of Subgam® per week.

Table 21: Number of Subgam® Infusions Compared with Target for Weekly Dosing

Statistic	Total Time on Subgam® (Weeks)	Number of Subgam® Infusions	Percentage of Target[a] Number of Subgam® Infusions
No. of Patients (n)	49	49	49
Mean	147.5	147.7	100.6
Median	154.0	146.0	99.1
SD	49.2	55.1	17.2
Min	24.0	22.0	61.6
Max	208.0	317.0	199.4
Lower 95%CI	133.4	131.9	95.7
Upper 95%CI	161.7	163.5	105.6

[a] Target infusions = no. of infusions if given weekly per protocol
Source: Table 14.2.13.5
NB: See filenote 001, Section 16.1.9, for details of data exclusions from this table

11.4.1.6 Dose-Corrected Serum IgG Levels

Tables 14.2.7.2, 14.2.7.3, 14.2.7.5, 14.2.7.7, and 14.2.7.9 present summaries of the dose-corrected mean serum IgG (g/L/mg/kg) in 6-monthly intervals for all patients, by the completers subgroup, diagnosis of PAD, age group and prior therapy respectively.

The dose-corrected mean serum IgG level was 0.094 g/L/mg/kg during the first 6 months of the study, and fell to 0.086 g/L/mg/kg, remaining at a steady level throughout the study (Table 14.2.7.2). The mean of individual mean dose-corrected serum IgG levels was consistently lower in patients with CVID/XLA, than those with other primary antibody deficiencies (Figure 14.2.7.6). Similarly, the mean of individual mean dose-corrected serum IgG levels was consistently lower in children than in teenagers and adults (Figure 14.2.7.8). This reflects the lower normal serum IgG levels in children.

11.4.1.7 Summary of Infections

Infections (see Table 14.2.10.1 to Table 14.2.10.8) are summarised in Table 22. No specific diagnostic tests were made to validate whether a suspected infection was a proven infection: thus, data on infections represent a worst-case analysis. It was not necessary for an infection to be verified by a clinician.

In the pre-Subgam® treatment phase (Infusions 1-3), there were 18 infections experienced by 17 out of a total of 50 patients across 1932 study days, equating to 3.40 infections per patient per year (source: Table 14.2.10.2b). The most common site for infection was the upper respiratory tract (Table 22). The majority of infections were considered mild or moderate in severity. There was one report of an infection (lower respiratory tract infection) classified as serious, equating to 0.19 serious infections per patient per year (source: Table 14.2.10.4b). There were no apparent differences between any of the subgroups studied, although the data should be interpreted with caution due to the small numbers of infections in each sub-category.

During the Subgam® treatment phase, the 50 patients participating in the study experienced a total of 513 infections (across 51,655 study days); this equates to 3.62 infections per patient per year (source: Table 14.2.10.2f). There were no apparent differences between any of the subgroups. There was a mean of 10.3 infections per patient, the most common sites being the upper respiratory tract (207 infections) and lower respiratory tract (179 infections) (source: Table 14.2.10.2d and Table 14.2.10.2f). The majority were mild or moderate; however, 10 adult patients experienced 19 severe infections and 1 teenage patient experienced 1 severe infection during the Subgam® treatment phase. There were no severe infections in children during the Subgam® treatment phase.

Of these 513 infections, 22 were classified as serious and were experienced by 9 patients (Table 14.2.10.4e). This equates to 0.16 serious infections per patient per year (source: Table 14.2.10.4f). These 22 serious infections comprised 17 serious LRTI infections, 1 serious gastrointestinal infection, 2 serious skin infections and 2 serious infections categorized as "other". Full details are provided in Table 14.2.10.4d and 14.2.10.4e.

The number of infections is displayed by type and month of onset in Table 14.2.10.8. As expected, the number of URTIs peaked in the winter months (December and February), falling to lower levels in the summer months (June and July). The onset of other types of infection was distributed more evenly throughout the year.

Table 22: Number of Infections Documented Across the Study - All Patients

	No. of Infections	No. Infections (No. Patients)											
		Mild			Moderate			Severe			Unknown		
		Adult[a]	Teens[a]	Child[a]	Adult[a]	Teens[a]	Child[a]	Adult[a]	Teens[a]	Child[a]	Adult[a]	Teens[a]	Child[a]
**Pre-Subgam®[b]**													
**Type[c]**													
URTI	9	4 (4)	0 (0)	3 (3)	0 (0)	0 (0)	1 (1)	0 (0)	0 (0)	0 (0)	1 (1)	0 (0)	0 (0)
LRTI	4	0 (0)	0 (0)	1 (1)	3 (3)	0 (0)	0 (0)	0 (0)	0 (0)	0 (0)	0 (0)	0 (0)	0 (0)
GI	1	1 (1)	0 (0)	0 (0)	0 (0)	0 (0)	0 (0)	0 (0)	0 (0)	0 (0)	0 (0)	0 (0)	0 (0)
Skin	0	0 (0)	0 (0)	0 (0)	0 (0)	0 (0)	0 (0)	0 (0)	0 (0)	0 (0)	0 (0)	0 (0)	0 (0)
Oral	1	1 (1)	0 (0)	0 (0)	0 (0)	0 (0)	0 (0)	0 (0)	0 (0)	0 (0)	0 (0)	0 (0)	0 (0)
Other	3	0 (0)	0 (0)	2 (2)	0 (0)	0 (0)	0 (0)	0 (0)	0 (0)	1 (1)	0 (0)	0 (0)	0 (0)
**Total**	**18**	**6 (6)**	**0 (0)**	**6 (6)**	**3 (3)**	**0 (0)**	**1 (1)**	**0 (0)**	**0 (0)**	**1 (1)**	**1 (1)**	**0 (0)**	**0 (0)**
**During Subgam®[d]**													
**Type[c]**													
URTI	207	72 (18)	28 (5)	43 (11)	33 (12)	5 (3)	21 (5)	3 (3)	0 (0)	0 (0)	2 (2)	0 (0)	0 (0)
LRTI	179	66 (21)	8 (3)	11 (4)	63 (16)	6 (2)	13 (2)	12 (7)	0 (0)	0 (0)	0 (0)	0 (0)	0 (0)
GI	7	4 (4)	0 (0)	1 (1)	1 (1)	0 (0)	0 (0)	1 (1)	0 (0)	0 (0)	0 (0)	0 (0)	0 (0)
Skin	28	8 (6)	3 (3)	10 (5)	3 (3)	0 (0)	2 (1)	2 (1)	0 (0)	0 (0)	0 (0)	0 (0)	0 (0)
Oral	10	8 (8)	0 (0)	0 (0)	1 (1)	0 (0)	0 (0)	1 (1)	0 (0)	0 (0)	0 (0)	0 (0)	0 (0)
Other	82	39 (13)	9 (4)	11 (5)	10 (7)	6 (2)	2 (2)	0 (0)	1 (1)	0 (0)	0 (0)	0 (0)	4 (1)
**Total**	**513**	**197 (27)**	**48 (7)**	**76 (13)**	**111 (20)**	**17 (3)**	**38 (6)**	**19 (10)**	**1 (1)**	**0 (0)**	**2 (2)**	**0 (0)**	**4 (1)**

a. Adults = ≥20y, teens = ≥12-<20y, child = <12 y
b. Patients received their usual immunoglobulin infusion at the same dose and frequency for three treatments prior to treatment with Subgam®
c. URTI = upper respiratory tract infection, LRTI = lower respiratory tract infection, GI = gastrointestinal, Oral = oral and dental
d. Infusion 4 (first Subgam® infusion) to last dose of Subgam®
Source: Tables 14.2.10.2a and 14.2.10.2d

11.4.1.8 Potential Serious Acute Bacterial Infections

Potential serious acute bacterial infections (SABI) are displayed by patient number in Table 14.2.10.7 and in Table 23. These were identified as described in Section 9.5.1.1. A total of 44 infections were identified by the criteria used. This number represents a maximum mean of 0.31 infections per patient per year. After scrutiny of the CRFs, 18 infections could not be discounted as potential SABIs. Therefore, 18 possible serious acute bacterial infections were identified in 11 patients during the Subgam® treatment phase (mean of 0.13 infections per patient per year). This compares favourably to expectations described by the US FDA (2005), who stated that patients with PAD could be expected to have about 0.5 (up to 1.0) serious acute bacterial infections per year. Narratives for all 44 potential serious acute bacterial infections are given in Section 14.2.1.

Table 23: Potential Serious Acute Bacterial Infections During Subgam® Treatment

Patient Number	Diagnosis	Elapsed Days from First Subgam® Infusion	Serious?	Severity
18	Dental abscess	987	No	Moderate
18	Pneumonia	673	No	Moderate
23	Severe exacerbation of chest infection	1205	Yes	Severe
27	Chest infection	59	Yes	Moderate
33	Collapse/consolidation (possible pneumonia)	42	No	Severe
57	Chest infection with purulent mucus	225	No	Mild
58	Abscess, left thumb	440	Yes	Moderate
58	Chest Infection, culture positive for *Pseudomonas aeruginosa*	78	Yes	Moderate
58	Chest infection	1022	Yes	Moderate
58	Perianal abscess	341	Yes	Severe
61	Chest infection with purulent sputum	330	No	Mild
73	Possible Pneumonia	223	Yes	Severe
73	Possible Pneumonia	256	Yes	Severe
73	Probable Pneumonia	450	Yes	Severe
77	Chest Infection	81	Yes	Moderate
78	Chest Infection, possible *Pseudomonas aeruginosa*	845	Yes	Severe
78	Chest infection, possible *Pseudomonas aeruginosa*	70	Yes	Moderate
83	Pneumonia	892	No	Mild

Source: Table 14.2.10.7

Of the 18 cases of possible serious acute bacterial infections, 12 were classified by the investigator as "serious" according to ICH criteria. Nine were chest infections, six were pneumonia (or possible pneumonia) and the remaining 3 were abscesses. There were no reports of septicaemia,

meningitis or septic arthritis. The duration of Subgam® dosing prior to onset of infection ranged from 42 days (Patient 33) to 1205 days (Patient 23). There were no serious acute bacterial infections in the teenagers, only in the adults (14 SABIs) and children (4 SABIs – Patients 27, 57, 61 and 83).

11.4.1.9 Antibiotic Use

Antibiotic use is displayed in Table 14.2.11.1 to Table 14.2.11.12d, and has been divided into several categories:

- Long-term prophylaxis – antibiotic given for at least 90 percent of the study days in any study year;
- Intravenous – antibiotic given intravenously;
- Acute – antibiotic given orally for a maximum period of 28 consecutive days;
- Other – any other antibiotics.

During the pre-Subgam® treatment phase, 24 patients received antibiotics for 32.8% of all study days (Table 14.2.11.1). During the much longer Subgam® treatment phase, 49 of the 50 patients received antibiotics during the study for a total of 40.3% of study days (see Table 24). During Subgam® treatment, 15 patients received antibiotics for long-term prophylaxis (26.6% of total study days), 10 received intravenous antibiotics (0.9% of study days), 46 oral antibiotics for acute therapy (10.0% of study days) and 21 other antibiotics (8.5% of study days) (Table 24). The number of study days on "Any antibiotics" is less than the combined sum of study days for each individual antibiotic category, because a day when a patient was on more than one antibiotic category counted as a single day on "Any antibiotics".

There were some differences in the number of antibiotic days as a percentage of total study days, and in the category of antibiotic treatment, between the different subgroups. Use of any antibiotic was higher amongst patients diagnosed with 'other' type of PAD, compared to those diagnosed with CVID/XLA. This was reflected in a higher use of all different categories of antibiotics, with the exception of acute antibiotics, in this patient group. It was particularly noticeable that long-term prophylactic antibiotics were used by 8

out of 17 patients with 'other' type of PAD, compared with only 7 out of 32 patients with CVID/XLA who received antibiotics. The reason for this difference is unknown, and this may just be a chance finding.

Use of any antibiotic was also higher amongst children than amongst teenagers or adults, with children spending 70.4% of study days on antibiotics compared to 40.3% of study days for the whole study population. Much of this difference was accounted for by long-term antibiotics that were used by 9 out of 15 children, compared with 6 out of 27 adults who received antibiotics and none of the teenagers. The reason for these differences is unknown but may reflect an increased willingness amongst clinicians managing immunodeficient children to use various treatment modalities to keep them infection free.

Overall, only 10 out of 50 patients dosed with Subgam® required intravenous antibiotics for the management of infections. Of these, 9 were adult patients and one was a child. This is consistent with the fact that as PAD patients reach adulthood they are more likely to have acute exacerbations of long-standing infections.

Table 24: Days on Antibiotics During Subgam® Treatment

Patient Group (Total Number of Days in Study for Patient Population)	Antibiotic Category	Number of Patients	Total Number of Antibiotic Days	Antibiotic Days as % of Days in Study for Population
**All Patients** (51655 days)	Any antibiotics	49	20838	40.3
	Long-term prophylaxis	15	13763	26.6
	Intravenous	10	482	0.9
	Acute	46	5157	10.0
	Other	21	4406	8.5
**By Diagnosis of PAD**				
CVID/XLA (36067 days)	Any antibiotics	32	11743	32.6
	Long-term prophylaxis	7	6550	18.2
	Intravenous	7	254	0.7
	Acute	32	3674	10.2
	Other	12	2582	6.9
Other (15588 days)	Any antibiotics	17	9095	58.3
	Long-term prophylaxis	8	7213	46.3
	Intravenous	3	228	1.5
	Acute	14	1483	9.5
	Other	9	1924	12.3
**By Age Group**				
Adult (28894 days)	Any antibiotics	27	9973	34.5
	Long-term prophylaxis	6	6031	20.9
	Intravenous	9	479	1.7
	Acute	27	3562	12.3
	Other	12	2259	7.8
Teenager (8601 days)	Any antibiotics	7	896	10.4
	Long-term prophylaxis	0	0	0.0
	Intravenous	0	0	0.0
	Acute	7	461	5.4
	Other	2	435	5.1
Child (14160 days)	Any antibiotics	15	9969	70.4
	Long-term prophylaxis	9	7732	54.6
	Intravenous	1	3	0.0
	Acute	12	1134	8.0
	Other	7	1712	12.1
**By Prior Therapy**				
IVIG (38009 days)	Any antibiotics	35	13099	34.5
	Long-term prophylaxis	9	8083	21.3
	Intravenous	7	408	1.1
	Acute	33	3995	10.5
	Other	13	2744	7.2
SCIG (13646 days)	Any antibiotics	14	7739	56.7
	Long-term prophylaxis	6	5680	41.6
	Intravenous	3	74	0.5
	Acute	13	1162	8.5
	Other	8	1662	12.2

Source: Table 14.2.11.3

11.4.1.10 Effect on Serum IgA and IgM Levels

Summary statistics for serum IgA and serum IgM levels are given in Tables 14.2.9.2 and 14.2.9.3, respectively. There were no marked changes in serum IgA and IgM levels during Subgam® treatment. Mean IgA levels were 0.97 g/L and 1.04 g/L before and during treatment respectively, and mean IgM levels were 0.84 g/L and 0.76 g/L before and during treatment respectively. IgM sometimes increases in the early months of treatment with IVIG. In this study the patients had treatment with IgG for at least 6 months prior to treatment with Subgam®.

11.4.1.11 Effect on Specific Antibodies

Listings of anti-pneumococcus and anti-HIB antibody data are given in Data Listing 16.2.6.4; summary data for anti-HIB are shown in Table 14.2.9.1. It was not possible to summarise the anti-pneumococcus data, as results were reported either in titres, units/mL or mg/mL. There was no marked change in anti-HIB antibody levels during Subgam® treatment in Stage 1. The mean anti-HIB antibody level during Stage 1 was 2.33 mg/mL (95% CI 2.12, 2.54; median = 1.88 mg/mL; n=256). Anti-HIB antibody levels remained at a fairly constant level for all the patients throughout the study. There were no clinically relevant changes.

Although there was variability between patients for anti-pneumococcus antibody levels, for most patients the results remained fairly consistent throughout the study, with some fluctuation. For some patients, the data was difficult to interpret, as results from different times during the study were presented in different units (other patients had results presented with no units, and the laboratories responsible were not able to provide the units). Some patients (Patients 01, 06, 08, 10, 13, 21, 22, 30, 81, and 82) showed a downward trend in anti-pneumococcus antibody levels as the study progressed, whereas others appeared to have an increase in antibody levels (Patients 29, 54, 86, and 57). However, all values were within the applicable reference ranges for each site.

11.4.1.12 Time Off Work/School

Of the 50 patients in the ITT population, 16 patients were excluded from analysis of time off work/school, as described in Section 9.7.7.3, and for another patient, days off work were described as 'not known'.

In total, 28 of the 33 applicable patients took at least one day off work/school, ranging from 1.5 days to 231 days (Table 14.2.12.1). This equated to a mean of 2.9% (median = 1.2%) of days off work/school during Subgam® treatment. Patients who had previously received IVIG treatment took a mean of 1.7% of days (median = 1.1%) off work/school compared with 7.5% of days (median = 1.3%) for patients who had previously received SCIG treatment (Table 25).

Table 25: Summary of Numbers of Days Off Work or School During Subgam® Treatment by Prior Therapy

	All Applicable Patients			By Prior Therapy					
				IVIG			SCIG		
	Total Days Off	Total No. Days Received Subgam	Days Off (%)[a]	Total Days Off	Total No. Days Received Subgam	Days off (%)[a]	Total Days Off	Total No. Days Received Subgam	Days Off (%)[a]
n	33	33	33	26	26	26	7	7	7
Mean	29.8	1049.5	2.9	19.9	1052.1	1.7	66.6	1040.1	7.5
Median	15.0	1071.0	1.2	11.0	1120.5	1.1	15.0	1022.0	1.3
SD	45.9	337.2	5.0	23.1	368.8	1.7	83.7	198.8	9.6
Min	0.0	164.0	0.0	0.0	164.0	0.0	5.0	856.0	0.5
Max	231.0	1450.0	26.2	77.5	1450.0	5.3	231.0	1399.0	26.2
Lower 95%CI	13.5	930.0	1.1	10.5	903.1	1.0	-10.8	856.3	-1.5
Upper 95%CI	46.1	1169.1	4.7	29.2	1201.0	2.4	144.1	1224.0	16.4

[a] Total days off/days receiving Subgam®; CI: Confidence Interval
Source: Tables 14.2.12.2 and 14.2.12.6
NB: See filenote 001, Section 16.1.9, for details of data exclusions from this table

When analysed by age, children took a mean of 3.7% (median = 2.3%) of days off school, teenagers took a mean of 1.8% (median = 1.6%) of days off work/school and adults took a mean of 2.9% (median = 0.5%) of days off work during Subgam® treatment (Table 26).

Table 26: Summary of Numbers of Days Off Work or School by Age During Subgam® Treatment.

	Adults			Teenagers			Children		
	Total Days Off	Total No. Days Received Subgam	Days Off (%)[a]	Total Days Off	Total No. Days Received Subgam	Days Off (%)[a]	Total Days Off	Total No. Days Received Subgam	Days Off (%)[a]
n	16	16	16	7	7	7	10	10	10
Mean	30.3	1030.8	2.9	23.7	1228.7	1.8	33.3	954.1	3.7
Median	4.5	1192.8	0.5	17.0	1274.0	1.6	18.3	885.0	2.3
SD	59.5	430.9	6.5	19.3	201.8	1.3	36.0	178.5	4.2
Min	0.0	164.0	0.0	4.0	960.0	0.4	5.0	752.0	0.5
Max	231.0	1450.0	26.2	59.0	1448.0	4.2	106.5	1282.0	12.2
Lower 95%CI	-1.5	801.2	-0.6	5.9	1042.1	0.6	7.5	826.4	0.7
Upper 95%CI	62.0	1260.4	6.4	41.5	1415.3	3.0	59.1	1081.8	6.7

[a] Total days off/days receiving Subgam®; CI: Confidence Interval
Source: Table 14.2.12.5
NB: See filenote 001, Section 16.1.9, for details of data exclusions from this table

11.4.1.13 Time Spent on Home Therapy

Of the 50 patients who entered the study, 49 started home therapy during Stage 1. The exception was Patient 31, who was an elderly patient who had problems finding a suitable friend or relative to help her with the infusions. Over 50% of patients were infusing at home after 7 weeks training on Subgam® (Table 14.2.13.1). As expected, patients on SCIG prior therapy started home therapy more quickly than patients on IVIG prior therapy, with a mean elapsed time from Infusion 4 to start of home therapy of 30.5 days (SD, 20.8; 95% CI 18.5, 42.5), compared to 57.3 days (SD, 35.0; 95% CI 45.3, 69.3), respectively (Table 14.2.13.4). Children also started home therapy earlier than other age groups, after a mean of 30.7 days (SD, 20.7; 95% CI 19.2, 42.2) compared to 58.1 days (SD 36.1; 95% CI 43.9, 72.4) for adults and 57.4 days (SD, 33.6; 95% CI 26.3, 88.5) for teenagers (Table 14.2.13.3).

During the study, patients received Subgam® for a mean of 147.5 weeks (range: 24.0 weeks to 208.0 weeks) (Table 27). Patients received a mean of 147.7 Subgam® infusions (range: 22 to 317), and patients received a mean of 93.6% of infusions at home (range: 63.6% to 99.4%).

Table 27: Subgam® Infusions Given as Home Therapy

Statistic	Total Time on Subgam® (Weeks)	Number of Subgam® Infusions	Number of Infusions Given at Home	Percentage Infusions Given at Home
No. of Patients (n)	49	49	48	48
Mean	147.5	147.7	142.1	93.6
Median	154.0	146.0	147.5	94.7
SD	49.2	55.1	51.9	5.7
Min	24.0	22.0	14.0	63.6
Max	208.0	317.0	309.0	99.4
Lower 95%CI	133.4	131.9	127.0	91.9
Upper 95%CI	161.7	163.5	157.1	95.2

Source: Table 14.2.13.5
NB: See filenote 001, Section 16.1.9, for details of data exclusions from this table

11.4.1.14 Patient Satisfaction with Subgam®

Patients' Overall Satisfaction with Subgam®

Patient Satisfaction Questionnaire data are provided in Appendix 16.2.6 (Listing 16.2.6.11) and summarised in Table 28 to Table 33 below.

Several patients did not complete all three questionnaires within the correct timeframes. Forty-eight patients completed Questionnaire 1 (pre-Subgam®), 38 patients completed Questionnaire 2 (3 months), and 43 patients completed Questionnaire 3 (6 months). For Table 32 and Table 33, where answers from Questionnaire 2 are compared with those from Questionnaire 1, data are included only from those patients who completed both Questionnaires, a total of 36 patients.

Table 28: Preference of Subgam® Compared to Previous Medication (After 3 Months)

			Preference to Previous Medication				
Patient Group		n	Much More	More	Same	Not as Much	Not at All
All Patients		38	20	14	3	1	0
Age Group	Adult	20	11	6	2	1	0
	Teenager	5	2	3	0	0	0
	Child	13	7	5	1	0	0
Diagnosis	CVID/XLA	26	11	11	3	1	0
	Other	12	9	3	0	0	0
Prior Therapy	SCIG	12	5	5	2	0	0
	IVIG	26	15	9	1	1	0

Source: Table 14.2.14.1
NB: See filenote 001, Section 16.1.9, for details of data exclusions from this table

Study Code: SCIG01
Filenote No.: N/A
Date: 13 September 2007

FILENOTE

Subject: Final Study Report – Correction of Typographical Error

The final study report was found to have a typographical error in the text following sign-off by all parties.

The typographical error was found in Table 29 (p98), where it was found to be inconsistent with the original table (14.2.14.2). The amendment involved correcting the number of patients diagnosed as CVID/XLA (in column 3 of the table), from 19 to 29.

The PI will be informed of this minor typographical amendment once the document is submitted to the Regulatory Authorities, at which time he will receive an amended copy of the report, with an explanation of the change.

Approved by:

Line Manager

K. Gillanders

Signature Kate Gillanders

13-SEP-2007

Date

Clinical Project Leader

[illegible]

Signature Samantha Leach

13 Sep 2007
Date

Data Manager – Not Applicable

Table 29: Preference of Subgam® Compared to Previous Medication (After 6 Months)

			Preference to Previous Medication					
Patient Group		n	Much More	More	Same	Not as Much	Not at All	p-value (a)
All Patients		43	26	7	8	2	0	
Age Group	Adult	22	14	2	5	1	0	0.45
	Teenager	6	3	2	0	1	0	
	Child	15	9	3	3	0	0	
Diagnosis	CVID/XLA	29	14	6	7	2	0	0.13
	Other	14	12	1	1	0	0	
Prior Therapy	SCIG	14	7	1	5	1	0	0.20
	IVIG	29	19	6	3	1	0	

Source: Table 14.2.14.2
(a) Analysed by the exact version of the Pearson chi-squared test
NB: See filenote 001, Section 16.1.9, for details of data exclusions from this table

Most patients stated that in general they liked Subgam® more or much more than their previous medication (89% and 77% after 3 and 6 months on Subgam® respectively).

Only 3 patients (see below) stated that they did not like Subgam® as much as their previous medication (1 patient after 3 months on Subgam® and 2 patients after 6 months on Subgam®). Two of those patients chose to continue taking Subgam®, and entered Stage 2 of the study.

- **Patient 29** (49 years old) stated after 3 months on Subgam® that he did not like Subgam® as much as his previous treatment (4-weekly infusions at the hospital with IVIG). He had rated his previous treatment as 'extremely comfortable' and 'very convenient', but thought that Subgam® was 'not very comfortable' and 'neither convenient nor inconvenient'. He felt that his symptoms were 'about the same'. This patient did not complete Questionnaire 3 at the End of Stage 1. However, he did choose to remain on Subgam® to take part in Stage 2 of the study rather than return to his previous medication after completion of Stage 1.
- **Patient 41** (18 years old) stated after 6 months on Subgam® (she did not complete Questionnaire 2 after 3 months), that she did not like Subgam® as much as her previous medication (3-weekly intravenous infusions at home). However, she rated her prior medication as 'not very comfortable' and 'very inconvenient', but rated Subgam® as 'quite comfortable' and 'quite inconvenient'. She felt that her symptoms were 'about the same'. She may have found Subgam® inconvenient because of the increase in frequency of infusions (i.e. once weekly).

This patient also chose to continue infusing Subgam® at the end of Stage 1, and took part in Stage 2 of the study.

- **Patient 76** (50 years old) stated after 3 months that she liked Subgam® 'much more' than her previous medication. However, after 6 months she stated that she 'did not like it as much'. This patient had also stated that her previous medication (weekly home infusions with SCIG) was 'extremely comfortable' and 'very convenient', whereas at both 3 and 6 months after starting the study she thought that Subgam® was 'very comfortable' and 'very convenient'. On both occasions the patient felt that her symptoms were 'worse'. Patient 76 chose not to take part in Stage 2 of the study.

There were no statistically significant differences between subgroups in terms of their preference of Subgam® to previous medication (see Table 29), indicating its suitability for use in a diverse patient population.

Patients' Symptoms While on Subgam®

Most patients stated that their symptoms were better or much better on Subgam® than they had on their previous medication (50% and 58% after 3 and 6 months on Subgam® respectively).

Table 30: Patients' Perception of Symptoms on Subgam® Compared to Previous Medication (After 3 Months)

Patient Group		n	Much Better	Better	Same	Worse	Much Worse
All Patients		38	11	8	18	1	0
By Age Group	Adult	20	5	4	10	1	0
	Teenager	5	2	2	1	0	0
	Child	13	4	2	7	0	0
By Diagnosis of PAD	CVID/XLA	26	8	5	12	1	0
	Other	12	3	3	6	0	0
By Prior Therapy	SCIG	12	3	2	6	1	0
	IVIG	26	8	6	12	0	0

Source: Table 14.2.14.3

Table 31: Patients' Perception of Symptoms on Subgam® Compared to Previous Medication (After 6 Months)

Patient Group		n	Much Better	Better	Same	Worse	Much Worse	p-value(a)
**All Patients**		43	13	12	16	2	0	
**By Age Group**	Adult	22	7	5	8	2	0	
	Teenager	6	2	1	3	0	0	0.79
	Child	15	4	6	5	0	0	
**By Diagnosis of PAD**	CVID/XLA	29	8	6	13	2	0	0.28
	Other	14	5	6	3	0	0	
**By Prior Therapy**	SCIG	14	4	2	7	1	0	0.46
	IVIG	29	9	10	9	1	0	

Source: Table 14.2.14.4
(a) Analysed by the exact version of the Pearson chi-squared test

Only 2 patients stated that they felt worse on Subgam® than they had on their previous medication, as follows:

- **Patient 22** (53 years old and previously on IVIG) stated after 6 months on Subgam® that her symptoms were 'worse', though after 3 months she had stated that they were 'about the same'. On both occasions the patient stated that she liked Subgam® 'more' or 'much more' than her previous medication. The patient chose to continue into Stage 2 of the study.
- **Patient 76** (50 years old and previously on SCIG) stated at both 3 and 6 months that she felt 'worse'. A detailed discussion of this patient is given above.

Patients' perception of symptoms on Subgam® compared to previous medication (after 6 months) did not show any statistically significant difference when analysed by age group (p=0.79), diagnosis of PAD (p=0.28) or prior therapy (p=0.46) (see Table 31 above).

Convenience and Comfort of Administering Subgam®

Table 14.2.14.5 to Table 14.2.14.8 present summaries of patients' perceptions of convenience and comfort of administering Subgam®, at 3 and 6 months. Each table shows results for all patients as well as by subgroup (diagnosis of PAD, age group and prior treatment).

Responses for comfort and convenience after 6 months of treatment with Subgam® were similar to those given after 3 months treatment. The discussion in this section will therefore focus on comparisons between

Questionnaires 1 and 2 (i.e. prior to starting Subgam® and after 3 months on Subgam®).

Patients' perceptions of the convenience of Subgam® at 3 months are summarised in Table 32. Of the 36 patients who completed Questionnaires 1 and 2, nine considered their prior medication to be 'quite inconvenient' or 'very inconvenient'. All of them were receiving IVIG prior to starting Subgam®. All of those patients who were receiving SCIG before starting Subgam® considered that their prior medication was either 'quite convenient' or 'very convenient'.

Of the 36 patients, 20 (including several patients who were previously receiving SCIG) gave Subgam® a higher rating for convenience than their previous medication, and only 5 of the 36 patients gave Subgam® a lower rating for convenience than their previous medication. Most patients (30 of the 36) considered that Subgam® was either 'quite convenient' or 'very convenient', compared with 22 of the 36 who thought that their previous medication was 'quite convenient' or 'very convenient'.

Table 32: Patients' Perception of Convenience on Subgam® Compared to Previous Medication (after 3 Months)

	After 3 Months Subgam®					
Pre-Subgam®	n	Very Inconvenient	Quite Inconvenient	Neither Convenient nor Inconvenient	Quite Convenient	Very Convenient
No. of Patients	36	0	0	6	7	23
Very Inconvenient	4	0	0	0	1	3
Quite Inconvenient	5	0	0	1	1	3
Neither Convenient nor Inconvenient	5	0	0	2	1	2
Quite Convenient	12	0	0	2	2	8
Very Convenient	10	0	0	1	2	7

Source: Table 14.2.14.5a

Six of the 36 patients considered that their prior medication was either 'not very comfortable' or 'extremely uncomfortable' (see Table 33). Four of them were children, only one of whom (Patient 27) was infusing with SCIG. All but 1 of these 6 patients found Subgam® more comfortable than their prior medication. In fact, 13 of the 36 patients gave Subgam® a higher rating for comfort than they gave their prior medication. Only 7 of the 36 patients gave Subgam® a lower rating for comfort than their previous IgG; 2 of those were infusing with SCIG before they started on the study.

Table 33: Patients' Perception of Comfort on Subgam® Compared to Previous Medication (After 3 Months)

		After Subgam® for 3 Months				
Pre-Subgam®	n	Extremely Uncomfortable	Not very Comfortable	Quite Comfortable	Very Comfortable	Extremely Comfortable
No. of Patients	36	0	2	18	14	2
Extremely Uncomfortable	2	0	0	2	0	0
Not Very Comfortable	4	0	1	3	0	0
Quite Comfortable	15	0	0	9	6	0
Very Comfortable	12	0	0	4	6	2
Extremely Comfortable	3	0	1	0	2	0

Source: Table 14.2.14.7a

11.4.2 Tabulation of Individual Efficacy Response Data

Individual efficacy response data are tabulated in Appendix 16.2.6.

11.4.3 Drug Dose, Drug Concentration and Relationships to Response

Drug dose details for all the patients are listed in Data Listing 16.2.5.1. Overall Subgam® exposure is summarised in Table 14.2.8.3. Patients received a mean of 147.7 infusions (range 22 to 317 infusions). The mean dose per infusion was 111.1 mg/kg (range 66.7 mg/kg rising to 170.8 mg/kg), (calculated from 49 subjects). There was no attempt to investigate any dose-response in this study. Indeed the protocol specified that the dosage should be adjusted to individual needs, as is usual in the long-term management of these patients.

11.4.4 Drug-Dose and Drug-Disease Interactions

IgG is a replacement therapy. No drug-dose or drug-disease interaction was expected, and none was evident.

11.4.5 By-Patient Displays

Data are provided by patient in various listings in Appendix 16.2. Where applicable, individual data are presented and discussed in the appropriate sections of this report. No further individual data is provided in this section.

11.4.6 Efficacy Conclusions

The efficacy conclusions are based on 50 patients (aged 0.8 to 75.2 y) whose period in the study on Subgam® ranged from 24 to 208 weeks (mean 147.5

weeks). During Stage 1 of treatment, 85% of adults/teenagers and 93% of children achieved target trough serum IgG levels at all time points.

The mean time to achieve a steady state serum IgG level was 6.14 Subgam® infusions for patients who had previously received IVIG treatment and 6.50 Subgam® infusions for patients who had previously received SCIG treatment, however only 4 patients included in the analysis had previously received SCIG.

Overall the total mean serum IgG level was 9.2 g/L in the pre-Subgam® phase of the study. This level increased to 9.75 g/L during the first 6 months of Subgam® treatment and was maintained above the pre-Subgam® level for 30 months of treatment. For patients completing the study, the total mean of means IgG level for 30-36 months of treatment was 95.0% of the level achieved for 0-6 months of treatment. However, data from the inter-infusion pharmacokinetics indicated that daily values of serum IgG had only small differences, confirming previous data from Waniewski *et al.* (1994).

The protocol stated that all Subgam® infusions would be taken at home on a weekly basis. The mean total time on Subgam® was 147.5 weeks (median = 154.0; n = 49 patients) and the mean number of Subgam® infusions was 147.7 (median = 146.0; n=49. Overall a mean total of 93.6% of infusions were given at home (median = 94.7; n=48). Patient 31 did not receive any home infusions, explaining the n = 48 value.

The required Subgam® dose level increased with time from 104.6 mg/kg during the first 6 months, reaching a plateau of around 115 mg/kg by 18-24 months of treatment. The trend in increased dose was seen in adults and children, however there was no noticeable increase with time observed in teenagers.

There was no marked increase in the frequency, severity or seriousness of bacterial infections during Subgam® treatment compared with the pre-Subgam® stage. Patients experienced a mean of 3.40 infections per patient per year in the pre-Subgam® treatment period and a mean of 3.62 infections per patient per year during Subgam® treatment. Patients experienced a mean of 0.19 serious infections per patient per year in the pre-Subgam® treatment

phase and a mean of 0.16 serious infections per patient per year during Subgam® treatment. Whilst receiving Subgam® treatment, patients experienced a mean of 0.13 potential serious acute bacterial infections (SABIs) per patient per year. This is appreciably below the expected maximum figure of up to 1.0 serious acute bacterial infection per patient per year, as outlined in guidance documents from the US FDA relating to clinical studies in this disease area (US Food & Drug Administration, 2005)

Patients who had previously received SCIG treatment experienced a higher percentage of days off work or school than patients who had previously received IVIG. In total, all applicable patients took a mean of 2.9% of treatment days off work or school (median = 1.2%), prior IVIG patients took a mean of 1.7% of days off work or school (median = 1.1%) and prior SCIG patients took a mean of 7.5% of days off work or school (median = 1.3%). Adults, teenagers and children took approximately the same amount of time off work or school (2.9%, 1.8% and 3.7% of days off school or work, respectively).

The majority of patients preferred Subgam® treatment to their previous therapy. After 3 months of treatment, 34 of 38 patients preferred Subgam® "more" or "much more" than their previous treatment and after 6 months of treatment patients continued to prefer Subgam®. There were no major differences at 6 months between patient sub-groups in terms of their preference for Subgam® compared with their previous treatment.

12 Safety Evaluation

Full summaries of safety data are provided in Section 14.3.

12.1 Extent of Exposure

12.1.1 Total Subgam® Exposure Over Study Duration

Table 14.2.8.3 to Table 14.2.8.6 present summaries of the number of infusions, mean dose per infusion (mg/kg) and total Subgam® exposure over the whole study (mg/kg) for all patients, and by diagnosis of PAD, age group and prior therapy, respectively.

Forty-nine patients (excluding Patient 42, due to non-compliance) received a mean total exposure of Subgam® of 16581.2 mg/kg during participation in the study, with a mean dose of 111.1 mg/kg per infusion (Table 34).

Table 34: Total Subgam® Exposure – All Patients

	Total Number of Infusions (n=49)	Mean Dose/Infusion (mg/kg) (n=49)	Total Subgam® Exposure (mg/kg) (n=49)
**Mean**	147.7	111.1	16581.2
**Median**	146.0	103.8	17392.3
**SD**	55.1	23.5	6996.4
**Min**	22.0	66.7	1866.6
**Max**	317.0	170.8	32183.8
**Lower 95% CI**	131.9	104.4	14571.6
**Upper 95% CI**	163.5	117.8	18590.8

Source: Table 14.2.8.3

NB: See filenote 001, Section 16.1.9, for details of data exclusions from this table

The statistical analyses presented in Tables 14.2.8.3 to 14.2.8.6 (summarised in the table above) exclude Patient 42 due to non-compliance. However for the purposes of safety data analysis, when the 98 infusions for Patient 42 are included, there were 7334 infusions in 50 patients (mean = 146.7 infusions per patient), a mean dose per infusion of 110.9 mg/kg, and a total Subgam® exposure over the whole study for all patients of 822,185 mg/kg (mean = 16443.7 mg/kg per patient).

There were no marked differences in number of infusions, dose/infusion or total Subgam® exposure between the patient subgroups (Tables 14.2.8.4 to 14.2.8.6).

Measured from the date of the first Subgam® dose to the date of the last Subgam® dose, 50 patients received Subgam® treatment for 51,655 days. This is equivalent to a total of 141.5 patient years experience.

12.1.2 Interval Between Subgam® Infusions

The mean time between infusions for all patients was 7.0 days (median = 7.0 days). There was no marked variation in time between infusions when stratified by age, diagnosis of PAD or prior therapy (Table 35).

The mean interval between infusions (7.0 days) was consistent with the protocol, and the tight 95% confidence limits (6.95 days to 7.06 days) suggest

that most patients complied broadly with the specified weekly dosing schedule. However, this parameter did show a substantial range (1 day to 83 days), suggesting that on some occasions at least a minority of patients were poor compliers.

Table 35: Interval Between Infusions (Days)

		n	Mean	Median	SD	Min	Max	Lower 95% CI	Upper 95% CI
**All Patients**		7193	7.00	7.00	2.45	1	83	6.95	7.06
**By Age Group**[a]	Adults	4035	6.85	7.00	2.66	1	67	6.77	6.93
	Teenagers	1192	7.21	7.00	1.57	2	28	7.12	7.30
	Children	1966	7.19	7.00	2.41	2	83	7.09	7.30
**By Diagnosis of PAD**	CVID/XLA	4898	7.11	7.00	2.29	1	67	7.04	7.17
	Other	2295	6.78	7.00	2.75	1	83	6.67	6.90
**By Prior Therapy**	IVIG	5284	6.95	7.00	2.48	1	67	6.89	7.02
	SCIG	1909	7.14	7.00	2.36	2	83	7.03	7.25

[a] Adults = ≥20 y, teens = ≥12-<20 y, child = <12 y
n = number of infusions
Source: Table 14.2.8.2

12.2 Adverse Events

12.2.1 Summary of Adverse Events

These adverse events exclude infections (see sections 11.4.1.7 and 11.4.1.8), but include SAEs (other than infections). In the period from pre-study to start of Infusion 4 (1,932 patient study days), 28 patients experienced 61 adverse events (59 not related, 2 related), which if extrapolated represents a mean of 11.52 adverse events per patient-study year. From the start of the first Subgam® treatment to End of Study (55,380 study days) all 50 patients experienced 1,490 adverse events (9.82 adverse events per patient-study year). Of these, 1345 (90%) were considered not related and 144 (10%) were considered related. One adverse event (cough) had an unspecified relationship (Table 36).

Table 36: Summary of Adverse Events Excluding Infections

	Mean (range) Duration of Treatment (Days)	Number of Adverse Events (No. of Patients)	Relationship		
			Not Related (No. of Patients)	Related (No. of Patients)	Not Specified (No. of Patients)
Pre-Subgam® a	38.6 (7-65)	61 (28)	59 (27)	2 (2)	0 (0)
During-Subgam® b	1107.6 (217-1563)	1490 (50)	1345 (50)	144 (30)	1 (1)

a (Pre-study to Infusion 3)
b (Infusion 4 to EOS)
Source: Table 14.3.1.1

12.2.2 Display of Adverse Events

Adverse events (excluding infections) are listed by patient in Appendix 16.2.7, and are summarised in Table 14.3.1.1 to Table 14.3.1.7. The most commonly reported adverse events, as expected for this patient population, were in the System Organ Class of Respiratory, thoracic and mediastinal disorders, which accounted for 410 adverse events, 27.5% of all adverse events.

Infusion site reactions (regardless of causality) accounted for 82 adverse events in 25 patients. The incidence of the infusion site reactions during Subgam® generally decreased with time, except between 30-36 months, where there was a slight increase. However the significance of this increase cannot be determined (see Table 37).

Table 37: Infusion Site Reactions (Regardless of Causality) in All Patients During Subgam® (Infusion 4 to End of Study)

Months:-	0-6	>6-12	>12-18	>18-24	>24-30	>30-36	>36-42	>42-48	>42-48
No. of Reactions	59	7	6	2	2	8	0	0	0
No. of Patients with Infusion Site Reactions	20	4	4	2	2	5	0	0	0
No. of Reactions Per Patient with a Reaction	2.95	1.75	1.5	1	1	1.6	0	0	0

Source: Table 14.3.3.1 and 14.3.3.7a to 14.3.3.7h

In total there were 1,408 other adverse events in 50 patients (1339 adverse events in 50 patients considered unrelated, 68 adverse events in 23 patients considered related and one adverse event of unspecified relationship respectively) (Table 38).

Table 38: Summary of Adverse Events by System Organ Class – During Subgam® (Infusion 4 to End of Study)

System Organ Class	Number Events (Number Patients)	Relationship		
		Not Related	Related	Not Specified
Blood and lymphatic system disorders	6 (6)	6 (6)	0	0
Cardiac disorders	3 (3)	3 (3)	0	0
Congenital, familial and genetic disorders	1 (1)	1 (1)	0	0
Ear and labyrinth disorder	21 (11)	21 (11)	0	0
Endocrine disorders	1 (1)	1 (1)	0	0
Eye disorders	9 (7)	9 (7)	0	0
Gastrointestinal disorders	263 (43)	250 (41)	13 (7)	0
General disorders & administration site conditions	255 (44)	166 (39)	89 (25)	0
Immune system disorders	8 (7)	8 (7)	0	0
Injury, poisoning and procedural complications	24 (17)	24 (17)	0	0
Investigations	48 (19)	45 (19)	3 (2)	0
Metabolism and nutrition disorders	5 (4)	5 (4)	0	0
Musculoskeletal and connective tissue disorders	123 (28)	118 (28)	5 (5)	0
Nervous system disorders	188 (32)	173 (29)	15 (12)	0
Pregnancy, puerperium and perinatal conditions	4 (1)	4 (1)	0	0
Psychiatric disorders	16 (7)	15 (7)	1 (1)	0
Renal and urinary disorders	7 (4)	7 (4)	0	0
Reproductive system and breast disorders	1 (1)	1 (1)	0	0
Respiratory, thoracic and mediastinal disorders	410 (45)	407 (45)	2 (2)	1 (1)
Skin and subcutaneous tissue disorders	76 (27)	62 (26)	14 (8)	0
Surgical and medical procedures	7 (5)	7 (5)	0	0
Vascular disorders	14 (9)	12 (9)	2 (1)	0
**Total**	**1490 (50)**	**1345 (50)**	**144 (30)**	**1 (1)**
Infusion Site Reactions	82* (25)	6 (6)	76 (21)	0
**Total (excluding infusion site reactions)**	**1408 (50)**	**1339 (50)**	**68 (23)**	**1 (1)**

Source: Table 14.3.1.3

*Two additional infusion site reactions were reported that were infections, therefore not classified as adverse events: infection to lower abdomen injection site (onset 30 Mar 2002) and infection at infusion site (onset 20Mar2003), both in Patient 22.

The most common adverse events (>5% of total adverse events) were headache (119 events), cough (141 events) and pharyngitis (103 events). However, the vast majority of these were considered to be unrelated to Subgam® (Table 39).

Table 39: Common Adverse Events (>5% of total) by Severity and Relationship

Preferred Term	N	Severity and Relationship						
		Mild		Moderate		Severe		Unknown
		Unrelated	Related	Unrelated	Related	Unrelated	Related	Not Specified
Headache	119	75	7	26	1	10	0	0
Cough	141	102	0	36	0	2	0	1
Pharyngitis	103	66	0	37	0	0	0	0

N = total number of events

Source: Table 14.3.1.5

12.2.3 Product-Related Adverse Events

As stated in Section 12.2.2, there were a total of 76 product-related infusion site reactions in 21 patients that were classified as related to Subgam®. A total of 68 other adverse events (in 23 patients) were also classified as related to Subgam® (Table 38). None of the product-related adverse events was classified as serious.

Table 14.3.1.8 displays all of the product-related adverse events with respect to MedDRA System Organ Class and Preferred Term. Infusion site reactions will be discussed in detail in the next section. Of the remaining 68 product-related adverse events, the most commonly reported were as follows: Headache (8 reports in 7 patients), Pruritus (7 reports in 2 patients), Vomiting NOS (5 reports in 3 patients) and Dizziness (3 reports in 2 patients) (see Table 14.3.1.8).

12.2.4 Infusion Site Reactions

Of the 144 adverse events that were considered related to Subgam®, 76 were local reactions at the site of infusion; these included: rash, swelling, inflammation, erythema, tenderness, pain, itchiness, bruising and bleeding (see Table 14.3.1.8 for details). Some of these reactions were probably due to the insertion of the infusion needle (bruising and haemorrhage) rather than the study medication itself.

The most commonly reported infusion site reactions were: Infusion site inflammation (14 reports in 7 patients), Infusion site swelling (13 reports in 6 patients), Infusion site erythema (11 reports in 5 patients) and Injection site rash (11 reports in 4 patients). These reactions occurred in 14 separate

patients and therefore some of the reactions were repeated in the same patients.

Infusion site reactions were analysed by subgroup and whether the patient was above or below the median BMI for the study or subgroup population. The aim of this analysis was to determine whether the probable presence of increased subcutaneous fat had an effect on the incidence of infusion site reactions. The number of infusion site reactions was highest during Stage 1, as would be expected during this period of intensive observation during infusions. Thereafter the number of infusion site reactions fell and none were reported after 36 months in the study (Table 40), as reported in other studies (Stein *et al.* 2006).

Table 40: Summary of Product-Related Infusion Site Reactions in 6-Monthly Intervals – by Diagnosis of PAD, Age Group and Prior Therapy

	Number of Reported Reactions						
	Stage 1		Stage 2				
	Pre-Subgam	0-6 Months (a)	>6-12 Months (b)	>12-18 Months	>18-24 Months	>24-30 Months	>30-36 Months
**ALL PATIENTS (n = 50)**							
Median BMI = 24.4							
< Median BMI	4	27	1	2	1	0	3
>= Median BMI	0	32	6	4	1	2	5
**BY DIAGNOSIS OF PAD**							
Median BMI = 26.5							
**CVID/XLA (n=33)**							
< Median BMI	1	31	6	2	0	0	1
>= Median BMI	0	19	1	2	1	2	4
Median BMI = 21.9							
**Other (n=17)**							
< Median BMI	3	3	0	2	1	0	0
>= Median BMI	0	6	0	0	0	0	3
**BY AGE GROUP**							
Median BMI = 26.6							
**Adults (n= 28)**							
< Median BMI	0	24	5	2	0	0	1
>= Median BMI	0	20	1	2	0	2	4
Median BMI = 24.4							
**Teenagers (n=7)**							
< Median BMI	0	10	0	0	0	0	0
>= Median BMI	0	1	1	0	1	0	0
Median BMI = 16.0							
**Children (n=15)**							
< Median BMI	3	3	0	2	0	0	0
>= Median BMI	1	1	0	0	1	0	3
**BY PRIOR THERAPY**							
Median BMI = 24.6							
**IVIG (n=36)**							
< Median BMI	0	26	2	0	1	0	3
>= Median BMI	0	19	5	4	1	1	5
Median BMI = 16.0							
**SCIG (n=14)**							
< Median BMI	3	3	0	2	0	0	0
>= Median BMI	1	11	0	0	0	1	0

Source: Table 14.3.3.1

For the study population as a whole, there was no evidence of BMI affecting the incidence of infusion site reactions, with 32 reactions in patients with a higher than median BMI, and 27 in patients with a lower than median BMI.

There were no apparent differences in the numbers of infusion site reactions between subgroups. In the teenagers subgroup, there were 10 infusion site reactions reported in patients with a lower than median BMI compared to just one in patients with a higher than median BMI; in the subgroup of patients who had received SCIG previously, there were 11 infusion site reactions reported in patients with a higher than median BMI compared to 3 in patients with a lower than median BMI (Table 14.3.3.1). However, such data must be viewed with caution due to the small numbers of patients in each subgroup. There were no other apparent differences in the number of infusion site reactions between patients with a lower than median BMI or higher than median BMI.

Regardless of causality, there were 84 infusion site reactions (including two infections) in 7,334 infusions of Subgam®, a mean of 0.01 infusion site reactions per infusion (Table 14.3.3.6). Of these, 74 (88.1%) were of mild intensity and 10 (11.9%) were of moderate intensity. There were no severe infusion site reactions during the study.

12.3 Deaths and Other Serious Adverse Events

12.3.1 Listings of Deaths and Other Serious Adverse Events

There were no deaths during the study. Excluding infections, which are addressed in Section 11.4.1.7, a total of 14 patients experienced 28 serious adverse events during participation in the study (pre- and during Subgam®). Of these, 16 (9 patients) were considered severe, 11 (8 patients) were considered moderate and 1 (1 patient) was considered mild (Table 41). Two of these serious adverse events (haematemesis and phlebothrombosis) occurred in the pre-Subgam® treatment phase. During the Subgam® treatment phase (Infusion 4 to End of Study), 13 patients experienced 26 serious adverse events; of these, none was considered related to Subgam® treatment.

Table 41: Serious Adverse Events by Severity (Infusion 1 to the End of Study)

Preferred Term	Number of Events (Number of Patients)	Severity Number of Events (Number of Patients)		
		Mild	Moderate	Severe
Abdominal pain NOS	1(1)	0	0	1(1)
Abortion spontaneous NOS	1(1)	0	1(1)	0
Appendicitis	1(1)	0	0	1(1)
Asthma NOS	2 (2)	0	0	2 (2)
Atrial fibrillation	1(1)	0	1(1)	0
Back pain	1(1)	0	0	1(1)
Blood potassium decreased	1(1)	0	0	1(1)
Blood pressure increased	1(1)	0	1(1)	0
Caesarean section	1(1)	0	0	1(1)
Chest pain	1(1)	0	0	1(1)
Chest tightness	1(1)	0	1(1)	0
Cough	1(1)	0	0	1(1)
Epistaxis	1(1)	0	1(1)	0
Granuloma NOS	1(1)	0	1(1)	0
Haematemesis[1]	1(1)	0	0	1(1)
Hernia repair NOS	1(1)	0	1(1)	0
Hypertension NOS	1(1)	0	1(1)	0
Neck pain	1(1)	0	0	1(1)
Nephrolithiasis	1(1)	0	0	1(1)
Neurological disorder NOS	1(1)	0	1(1)	0
Open fracture	1(1)	1(1)	0	0
Pain in limb	1(1)	0	0	1(1)
Phlebothrombosis[1]	1(1)	0	1(1)	0
Pyrexia	1(1)	0	0	1(1)
Rigors	1(1)	0	0	1(1)
Sinus operation NOS	1(1)	0	1(1)	0
Vomiting in pregnancy	1(1)	0	0	1(1)
**Total**	**28 (14)**	**1(1)**	**11(8)**	**16 (9)**

[1] SAE occurred in pre-Subgam™ treatment phase

NOS = Not Otherwise Specified

Source: Table 14.3.2

12.3.2 Narratives of Deaths and Other Serious Adverse Events

There were no deaths during the study. Narratives of infections identified as potential serious acute bacterial infections (as described in Section 9.5.1.3) are given in Section 14.2.1.

12.4 Clinical Laboratory Evaluations

There was no notable overall change in haematology or biochemistry parameters before and after participation in the study (Table 42 and Table 43).

12.4.1 Listing of Individual Laboratory Measurements by Patient and Each Abnormal Laboratory Value

Listings of laboratory data can be found in Appendix 16.2.8; Listings 16.2.8.1 to 16.2.8.4, and Listing 16.2.8.7. Data for abnormal laboratory values can be found in Appendix 16.8.8; Listings 16.2.8.5 to 16.2.8.6.

12.4.2 Evaluation of Each Laboratory Parameter

12.4.2.1 Haematology

Haematology results, at Pre-Study and the End of Stage 1, are summarised in Table 14.3.4.1 and Table 42.

Table 42: Haematology Parameters

Parameter	Pre-Study		End of Stage 1	
	Mean (SD)	Range	Mean (SD)	Range
Haemoglobin (g/dL)	13.4 (1.4)	9.7-16.1	13.5 (1.4)	10.8-16.6
Haematocrit (%)	39.6 (4.2)	30.0-48.0	40.3 (4.3)	33.0-49.0
Erythrocytes (10^{12}/L)	4.7 (0.5)	3.6-5.9	4.7 (0.5)	3.3-6.0
Leukocytes (10^{9}/L)	6.9 (3.0)	2.2-17.8	7.2 (3.1)	1.9-15.9
Neutrophils (10^{9}/L)	3.7 (1.5)	1.0-7.4	4.2 (2.3)	0.8-12.8
Lymphocytes (10^{9}/L)	2.4 (1.8)	0.6-9.8	2.1 (1.5)	0.8-8.8
Monocytes (10^{9}/L)	0.49 (0.28)	0.15-1.38	0.50 (0.23)	0.17-1.20
Eosinophils (10^{9}/L)	0.23 (0.21)	0.00-0.96	0.24 (0.31)	0.00-1.85
Basophils (10^{9}/L)	0.04 (0.05)	0.00-0.26	0.05 (0.05)	0.00-0.22
Platelets (10^{9}/L)	286.8 (110.7)	89.0-603.0	276.7 (96.1)	97.0-484.0
Reticulocytes (10^{9}/L)	64.3 (31.7)	23.0-159.6	62.8 (36.3)	18.3-146.2

Source: Table 14.3.4.1

Haematology follow-up after Stage 1 was only to be done if clinically indicated.

Although some of the haematology results were marginally outside the normal ranges, very few of them were considered by the investigators to be of clinical significance. High WBC counts and differentials were noted in a few patients: these were probably a result of the fact that these patients are more susceptible to infections.

Clinically relevant haematology results are provided in Appendix 16.2.8 (Listing 16.2.8.6) and are described as follows:

Patient 33, CVID, 30 years, male

- Low platelets (normal range = 150–360 x 10^9/L): 97 x 10^9/L at End of Stage 1.
- Low platelets (normal range = 150–360 x 10^9/L): 38 x 10^9/L at End of Study.

Patient 33 had consistently low platelet counts throughout the study – 91 x 10^9/L, 76 x 10^9/L, 63 x 10^9/L, 60 x 10^9/L and 67 x 10^9/L at Pre-study, Infusions 8, 10 and 22, and at the End of Stage 1 assessment respectively. The End of Stage 1 assessment was repeated twice resulting in values of 6 9x 10^9/L and 97 x 10^9/L; the latter value has been used in Table 41 and is quoted above. This event had also been reported as an ongoing adverse event (mild intensity, non-serious) prior to Infusion 4, although no treatment was required. The investigator considered the abnormality to be attributable to cytopenia – either secondary to the autoimmune process or due to hypersplenism. At the End of Stage 2 assessment, his platelet count was 38 x 10^9/L. The investigator attributed this to persistence of neutropenia and thrombocytopenia, which did not improve on return to IVIG therapy. Other haematological variables for this patient were unremarkable.

- Low neutrophils (normal range = 1.7-7.5 x 10^9/L): 1.0 x 10^9/L at End of Study.

Patient 33 had a low neutrophil count at the End of Stage 1 assessment (0.8 x 10^9/L) that increased marginally to 1.0 x 10^9/L at the end of study assessment. The investigator considered the abnormality to be due to neutropenia which was evident before the patient commenced Subgam® therapy. The low neutrophil count was reported as an adverse event (moderate severity, non-serious) that was unlikely to be related to Subgam®, and was ongoing at the end of the study. (See also section 12.4.2.2 for clinically relevant biochemistry results in this patient at the end of the study).

Patient 35, CVID, 60 years, female

- High haemoglobin (normal range = 11.5-14.5 g/dL): 17.0 g/dL at End of Study
- High haematocrit (normal range = 36-46%): 51% at End of Study
- High RBC (normal range = 3.8-5.0 x 10^{12}/L): 5.97 x 10^{12}/L at End of Study

On entry to the study, the patient had slightly high haemoglobin, haematocrit and RBC values consistent with their COPD. The high levels at the end of the study (almost 4 years later) are, therefore, not unexpected and represent continuing COPD.

None of these results were considered to be clinically unexpected, because of the underlying pathology.

Patient 49, CVID, 13 years, female

- High white blood cells (normal range = 1.7–7.5 x 10^9/L): 14.4 x 10^9/L at End of Stage 1.
- High neutrophils (normal range = 3.5–10 x 10^9/L): 9.6 x 10^9/L at End of Stage 1.

These results are indicative of an infection: the patient was showing signs of a sinus infection at the time.

Patient 82, CD40 ligand deficiency, 4 years, male

- Low haptoglobin (normal range = 0.45-2.42 g/L): 0.24 g/L at End of Stage 1.

This patient had a low haptoglobin (0.36 g/L) at the pre-study visit, which was repeated four weeks later and was still 0.36 g/L. The investigator commented that the patient had slight eosinophilia and did not consider this to be clinically relevant. Following the low haptoglobin result (0.24 g/L) at End of Stage 1, a repeat test was performed three months later that gave a result of 0.45 g/L.

Patient 84, CVID, 13 years, female

- Low neutrophils (normal range = 1.8-8.0 x 10^9/L): 1.5 x 10^9/L at End of Stage 1.

This low neutrophil count was considered to be mild neutropenia by the investigator, consistent with immunodeficiency. A repeat test 2.5 months later gave a neutrophil result of 2.68 x 10^9/L.

In conclusion there were no clinically relevant changes in haematology parameters that were considered to be related to Subgam®.

12.4.2.2 Biochemistry (including Liver Function Tests)

ALT and AST were measured more frequently in Stage 1 than other biochemistry parameters. High ALT and AST values were noted in a few patients (see Listing 16.2.8.3). Shift tables for liver function tests are shown in Tables 14.3.4.5 and 14.3.4.6 for ALT and AST, respectively.

Although some of the biochemistry results were marginally outside the normal ranges, very few of them were considered by the investigators to be of clinical significance. For the whole study there were no clinically relevant changes that were considered to be related to Subgam®.

Other biochemistry results at Pre-Study and the End of Stage 1 are summarised in Table 43 (source: Table 14.3.4.2, Table 14.3.4.3 and Table 14.3.4.4).

Table 43: Biochemistry Parameters

Parameter	Pre-Study		End of Stage 1	
	Mean (SD)	Range	Mean (SD)	Range
Sodium (mmol/L)	139.9 (2.0)	135.0-144.0	140.3 (2.0)	137.0-145.0
Potassium (mmol/L)	4.2 (0.4)	3.4-6.0	4.3 (0.5)	3.6-5.9
Creatinine (µmol/L)	66.0 (20.1)	25.0-106.0	67.4 (22.0)	27.0-122.0
LDH (U/L)	504.0 (372.5)	117.0-2017.0	508.3 (264.6)	121.0-964.0
GGT (U/L)	29.4 (35.0)	6.0-210.0	28.4 (29.3)	4.0-150.0
Total Bilirubin (µmol/L)	9.1 (4.7)	1.0-26.0	8.3 (4.0)	2.0-19.0
Alkaline Phosphatase (U/L)	172.8 (138.0)	40.0-662.0	157.6 (124.0)	41.0-527.0
Haptoglobin (g/L)	1.4 (0.7)	0.3-3.6	1.2 (0.6)	0.0-2.0

Source: Tables 14.3.4.2

Clinically relevant biochemistry results are provided in Appendix 16.2.8 (Listing 16.2.8.6) and are described as follows:

Patient 13, specific antibody deficiency, 9 years old, female

- High LDH (normal range = 0-582 U/L): 612 U/L at Infusion 3

The patient's LDH returned to within normal range (494 U/L) at Infusion 4 and remained within range for the rest of the study.

Patient 21, CVID, 35 years, male

- High ALT levels (normal range = 0–36 U/L): 52, 49 and 73 U/L at Pre-study and Infusions 5 and 22 respectively.
- High GGT levels (normal range = 0–50 U/L): 56 U/L at Infusion 22.

The high ALT levels at Pre-study and Infusion 5 were considered by the investigator to be the result of an infection. The high ALT and GGT levels at Infusion 22 were considered by the investigator to be the result of an intercurrent sinus infection. Finally, in response to reports by the patient of aching joints, the investigator ran an unscheduled laboratory test (creatine kinase) at an unscheduled visit (Infusion 24). The creatine kinase level was 245 IU/L (normal range 26–174 /L). In the opinion of the investigator, the high ALT and GGT levels were indicative of a viral infection (possibly myositis), which was causing the symptoms of joint pain. This was reported as an adverse event (moderate intensity, non-serious), from which the patient recovered with no residual effects.

Patient 33, CVID, 31 years old, male

- High alkaline phosphatase (normal range = 0-125 U/L): 183 U/L at End of Study
- High ALT (normal range = 0-50 U/L): 61 U/L at End of Study
- High GGT (normal range = 7-40 U/L): 100 U/L at End of Study

This patient's high alkaline phosphatase, ALT and GGT were due to mild hepatic inflammation, likely of autoimmune origin, which had been diagnosed Pre-study.

Patient 34, CVID, 47 years old, male

- High ALT (normal range = 0-50 U/L): 111 U/L at End of Study
- High GGT (normal range = 7-40 U/L): 128 U/L at End of Study

This patient's high ALT and GGT levels were due to a high use of ibuprofen for treatment of a shoulder injury.

Patient 73, CVID, 47 years, female

- High ALT (normal range = 2–53 U/L): 113 U/L at Infusion 28.
- High AST (normal range = 2–53 U/L): 214 U/L at Infusion 28.

The patient had a chest infection and was given a 5-day course of ciprofloxacin that started 3 days before Infusion 28. In the opinion of the investigator, it is likely that the antibiotic caused the increase in liver function tests. Both were reported as adverse events (moderate severity, non-serious), from which the patient recovered with no residual effects.

Patient 81, CVID, 3 years, male

- High GGT (normal range = 5–16 U/L): 25, 20, 21, 21, 19 and 19 U/L at Pre-study and Infusions 3, 4, 8, 11 and End of Stage 1 respectively.

High LDH (normal range = 500–920 U/L): 2017, 899 and 958 U/L at Pre-study, Infusion 3 and End of Stage1 respectively.

GGT levels were just above normal range at Pre-study and remained slightly elevated through to End of Stage 1. LDH levels had returned to just within range by Infusion 3 and were just above range at the End of Stage 1. These abnormal values were present before the start of Subgam® and therefore not considered to be related to study drug.

Patient 83, CD40 ligand deficiency, 9 months, male

- High ALT (normal range = 5–45 U/L): 58 U/L at Infusion 16.
- High GGT (normal range = 5–16 U/L): 17 U/L at Pre-study
- High LDH (normal range = 500–920 U/L): 1223 U/L at Pre-study.

The ALT test was repeated 4 weeks after Infusion 16 and had returned to within normal range. LDH levels were high at Pre-study and remained above

range for the whole of the study, though by the End of Stage 1 they had reduced to 964 U/L (only slightly above normal range). GGT levels also remained high throughout the study, though they were only slightly above normal range. These values were not considered to be related to study drug.

Patient 86, CVID, 8 years, female

- High LDH (normal range = 420–750 U/L): 942 U/L at Pre-study.

This was an isolated event at Pre-study. LDH levels had returned to normal by Infusion 2. This value was not considered to be related to study drug.

12.4.3 Virology

12.4.3.1 HIV PCR Test Results

There were no positive findings to suggest virus transmission (see Listing 16.2.8.4a).

12.4.3.2 HBsAg, HCV and Parvovirus B19 Test Results

HBsAg, HCV and Parvovirus B19 tests were performed in all patients. There was no evidence of transmission of HBV, HCV or Parvovirus B19 infection in the study.

12.5 Vital Signs, Physical Findings, Other Observations related to Safety

12.5.1 Vital Signs

Table 14.3.5.1 to Table 14.3.5.31 present summaries of vital signs: (systolic blood pressure, diastolic blood pressure, pulse rate, respiration rate and body temperature). Summaries are shown for pre-, during and post-infusion observations during each infusion in Stage 1; for pre-, during and post-infusion observations consolidated for Infusions 1-3 (pre-Subgam®); for pre-, during and post-infusion observations for Infusions 4-30 (post-Subgam®); for each visit number during Stage 2; and for observations recorded during the pharmacokinetic sub-study.

There were no marked changes in vital signs during the study (Table 44). Only 49 patients reported observations for the pre-study visit, because vital signs were not measured for Patient 01 at this visit.

Table 44: Pre- and Post-Subgam® Vital Signs and Physical Findings

	SBP (mmHg)		DBP (mmHg)		Pulse (bpm)		Respiration Rate (Breaths per min)	
	**PRE**	**EOS**	**PRE**	**EOS**	**PRE**	**EOS**	**PRE**	**EOS**
**n**	49	47	49	47	49	47	49	46
**Mean**	119.00	122.77	70.92	72.94	80.59	80.00	18.90	18.28
**Median**	116.00	120.00	68.00	72.00	78.00	80.00	18.00	17.50
**SD**	21.02	18.01	14.10	16.42	14.27	14.24	4.02	3.66
**Min**	81.00	91.0	40.00	37.00	58.00	50.00	12.00	14.00
**Max**	170.00	170.00	106.00	130.00	129.00	126.00	28.00	32.00
**Lower 95% CI**	112.96	117.48	66.87	68.12	76.49	75.82	17.74	17.20
**Upper 95% CI**	125.04	128.05	74.97	77.76	84.69	84.18	20.05	19.37

SBP = Systolic Blood Pressure; DBP = Diastolic Blood Pressure

PRE = Pre-Study Visit; EOS = End of Study

Source: Table 14.3.5.1; Table 14.3.5.4; Table 14.3.5.6; Table 14.3.5.9; Table 14.3.5.11; Table 14.3.5.14; Table 14.3.5.16; Table 14.3.5.19

There were also no marked changes in vital signs before, during and after each infusion in Stage 1 or measured at each visit to the study site in Stage 2.

Table 45 displays the number of reports of body temperatures of >37°C pre-infusion and post-infusion during Stage 1, respectively. Only 49 patients reported observations for Infusions 1-3, because Patient 01 took these infusions as home therapy and body temperature was not measured.

Table 45: Pre- and Post-Subgam® Body Temperatures >37°C During Stage 1

	Observations		Patients	
	n	Percentage Observations >37°C	n	Percentage Patients with Temp >37°C
**Pre-Subgam® (Infusions 1-3)**				
Pre-infusion	191	8.9	49	18.4
Post-infusion	493	12.2	49	34.7
**During-Subgam® (Infusions 4-30)**				
Pre-infusion	590	9.5	50	38.0
Post-infusion	945	8.5	50	44.0

Source: Table 14.3.5.27 and Table 14.3.5.28

The percentage of observations of body temperature >37°C was similar, whether measured pre- or post-infusion of Subgam®. The percentage of patients reporting body temperatures of >37°C was lower for pre-infusion observations during Infusions 1-3 than at any other time, but this was probably an artefact of the lower number of observations at this time-point.

Table 14.3.5.29 displays the number of reported body temperature elevations of >37°C during Stage 1 (*i.e.* pre-infusion body temperature of <37°C followed by post-infusion body temperature of >37°C). In the pre-Subgam® treatment phase, such temperature elevations were observed in 10.7% of infusions and in 36.7% of patients. In the Subgam® treatment phase, these were observed in 8.4% of infusions and in 62.0% of patients. The increase in number of patients reporting temperature elevations in the Subgam® phase (Stage 1) is likely to be due to the higher number of infusions in this phase compared to the pre-Subgam® phase and much longer follow-up phase (1535 and 684 infusions, respectively). The incidence of temperature elevation per infusion compared to the pre-Subgam® phase, however, remained steady, indicating there was no trend of body temperature elevations during treatment with Subgam®.

Table 46 shows the number of AE reports with MedDRA preferred term 'Pyrexia' or 'Body Temperature increased'. All adverse events classified as possibly or probably related to study drug by the investigator are presented as related. All events considered to be unlikely to be related or not related to study drug are classed as not related.

Table 46: Adverse Events During Subgam® With Preferred Term of Pyrexia or Body Temperature Increased

Preferred term	n	Relatedness to Subgam®		
		Related	Not related	Unknown
Pyrexia	27	1	26	0
Body Temperature Increased	30	2	28	0

Source: Table 14.3.1.5

Only 3 reports of increased body temperature/pyrexia were considered to be possibly related to Subgam®. These episodes were mild in severity and all recovered with no residual effects:

- **Patient 5** (a 2-year-old male with IgG subclass deficiency, previously on IVIG) reported a "high temperature" that started 2 days after Infusion 12 and resolved 1 day before Infusion 14. This patient also reported a "high temperature" that started 1 day after Infusion 22 and resolved on the day of Infusion 23. No high temperatures (i.e. >37°C) were recorded as part of the vital signs assessment apart from a high temperature before (37.3 °C) and during Infusion 5 (37.2°C).

- **Patient 26** (a 5-year-old male with X-linked agammaglobulinaemia, previously on SCIG) reported a fever that started 1 day after Infusion 5 and lasted for 1 day. No high temperatures (i.e. >37°C) were recorded as part of the vital signs assessment.

In Stage 1 during Subgam® treatment, 13 of the temperature elevations (in 5 patients) had corresponding adverse events coded with the MedDRA Preferred term of Pyrexia (Table 14.3.5.31).

12.5.2 Concomitant Therapy

Concomitant Medications are listed in Appendix 16.2.9 (Listing 16.2.9.3). The majority of concomitant medications were non-steroidal anti-inflammatory drugs, antibiotics and drugs for the treatment of asthma symptoms. The number of days that patients spent on antibiotics is a measure of efficacy in this study, and as such is discussed in more detail in Section 11.4.1.9.

12.6 Safety Conclusions

Fifty patients were dosed with Subgam® at approximately weekly intervals for a total of 51,655 study days, equivalent to approximately 141.5 patient years. Overall patients receiving Subgam® therapy experienced a similar amount of adverse events and SAEs per study day compared with patients in the pre-Subgam® phase. The most common adverse events (regardless of causality) were headache, cough and pharyngitis, and the majority of these were considered unrelated to Subgam®. There were no withdrawals due to adverse events or safety considerations.

The most commonly reported adverse events classified as related to Subgam® were infusion site reactions (76 reports in 21 patients). There were a further 68 product-related adverse events in 23 patients. These were rather non-specific in nature, and included 8 reports of headache, 7 reports of pruritus and 5 reports of vomiting. None of the product-related adverse events was classified as serious.

There were no clinically relevant changes in haematology or biochemistry parameters that were considered by the investigator to be related to Subgam®. Also, there was no evidence of the transmission of HIV, HBV, HCV

or Parvovirus B19. Likewise, there were no clinically important changes in vital signs that were attributed to Subgam® therapy.

13 Discussion and Overall Conclusion

Replacement therapy with IVIG is now accepted practice for patients with primary antibody deficiency (PAD). However this mode of administration is not suitable for all patients due to poor venous access, systemic adverse reactions and the need for frequent hospital visits. S/D Human Normal Immunoglobulin (Subgam®) used in this study has proven to be a suitable replacement therapy and an alternative to IVIG in PAD patients. Maintenance of adequate serum IgG concentrations by using rapid subcutaneous infusions has been well documented in previous studies (Gardulf *et al.*, 1995 & Gaspar *et al.*, 1998). The findings from this study are consistent with those reports.

In this study of Subgam®, 50 patients (28 aged ≥ 20 years, 7 aged 12<20 years and 15 < 12 years) received a mean total exposure of 16,443.7 mg/kg in a mean total of 146.7 infusions (range: 22 to 317). Overall Subgam® treatment was equivalent to 141.5 patient years.

During Stage 1 of treatment, 85% of adults/teenagers and 93% of children achieved target trough serum IgG levels at all observations. The mean time to achieve a steady state serum IgG level was 6.14 Subgam® infusions for patients who had previously received IVIG treatment and 6.50 infusions for patients who had previously received SCIG treatment.

Mean IgG levels increased from 9.2 g/L to 9.75 g/L during Subgam® treatment and were maintained above pre-Subgam® levels for 30 months of treatment. For patients completing the study, the total mean of means IgG level for 30-36 months of treatment was 95.0% of the level achieved for 0-6 months of treatment.

Overall 93.6% of Subgam® infusions were given at home. The dose of Subgam® required to maintain IgG levels increased with time from a mean of 104.6 mg/kg during the first 6 months, reaching a plateau at around 115 mg/kg by 18-24 months of treatment. The trend in increased dose was seen in adults and children; however there was no noticeable increase with

time observed in teenagers. Dosage increases are to be expected in a proportion of patients with PAD as clinicians adjust the dose on an individual basis to meet clinical need.

There was no notable change in serum IgM levels during Subgam® treatment.

There was no marked increase in the frequency, severity or seriousness of bacterial infections during Subgam® treatment. Patients experienced 3.40 infections per patient per year in the pre-Subgam® treatment phase and 3.62 infections per patient per year during Subgam® treatment. Patients experienced 0.19 serious infections per patient per year in the pre-Subgam® phase, 0.16 serious infections per patient per year during Subgam® treatment and 0.13 potential serious acute bacterial infections per year during Subgam® treatment. The latter figure is well below the target of not more than 1.0 serious acute bacterial infections set by the US FDA in their guidelines for clinical studies on intravenous immunoglobulin in primary antibody deficiency (US Food & Drug Administration, 2005).

Applicable patients who had previously received SCIG treatment experienced a higher percentage of days off work or school than patients who had previously received IVIG. In total, all applicable patients took a mean 2.9% (median = 1.2%) of treatment days off work or school, prior IVIG patients took a mean 1.7% (median = 1.1%) of days off work or school and prior SCIG patients took a mean 7.5% (median = 1.3%) of days off work or school. Adults, teenagers and children took approximately the same amount of time off work or school (2.9%, 1.8% and 3.7% of days off school or work respectively).

In general, compliance was very good. Adults and children and even teenagers (who were widely recognized by clinicians to present difficulties in this area) were compliant and seemed to prefer Subgam® to their previous IgG therapy. Only 1 patient was consistently poorly compliant (Patient 42); he was also poorly compliant on his prior IVIG therapy. Patient satisfaction assessed by a questionnaire after 3 to 6 months treatment supports the conclusion that subcutaneous administration of IgG is a well-liked regimen for patients and parents/guardians.

The majority of patients preferred Subgam® treatment to their previous therapy. This is consistent with the findings of Kittner *et al.* (2006). After 3 months of treatment, 34 of 38 patients preferred Subgam® "more" or "much more" than their previous treatment and after 6 months of treatment patients continued to prefer Subgam®. There was no major difference between patients previously treated with SCIG or IVIG.

During the study Subgam® was shown to be safe and well tolerated, with few systemic adverse reactions. Of those adverse events that were considered either possibly or probably related to Subgam® (144 in total), the majority (76 or 53%) were infusion site reactions (predominantly local rash and inflammation), and none of those were severe. There were no deaths, and of the 28 serious adverse events reported by 14 patients, none was considered to be related to Subgam®, nor were there any clinically relevant changes in laboratory tests, blood pressure, pulse or respiratory rate that could be attributed to Subgam®.

Subgam® was safe and well tolerated and was effective in increasing and maintaining serum IgG levels with only moderate dose increases required with time. The majority of patients in the study preferred Subgam® treatment to their previous treatments. The overall incidence of bacterial infections during Subgam® therapy was acceptably low.

In conclusion, the results indicate that the rapid subcutaneous administration of Subgam® is a safe, well-tolerated, convenient and effective alternative replacement therapy for a range of immune deficiencies.

14 Tables, Figures and Graphs

14.1 Demographic Data

14.2 Efficacy Data

14.2.1 Narratives of Potential Serious Acute Bacterial Infections

Table 47: Potential Serious Acute Bacterial Infection by Narrative Number

Narrative Number	Patient Number	Indication	Severity	Serious Y/N	SABI Y/N
1	73	Chest infection	Severe	Y	Y
2	73	Chest infection	Severe	Y	Y
3	73	Pneumonia	Severe	Y	Y
4	75	Chest infection	Severe	Y	N
5	75	Exacerbation of bronchiectasis	Severe	Y	N
6	23	Chest infection	Severe	Y	Y
7	22	Chest infection	Severe	N	N
8	75	Chest infection	Moderate	Y	N
9	75	Chest infection	Severe	Y	N
10	22	Exacerbation of bronchiectasis	Severe	Y	N
11	83	Pneumonia	Mild	N	Y
12	22	Chest infection	Severe	Y	N
13	58	Left thumb infection	Moderate	Y	Y
14	58	Chest infection	Moderate	Y	N
15	58	Chest infection	Severe	Y	N
16	58	Chest infection	Moderate	Y	Y
17	58	Increased production purulent sputum (chest infection)	Moderate	Y	Y
18	77	Chest infection	Moderate	Y	Y
19	27	Chest infection	Moderate	Y	Y
20	78	Pseudomonas (chest infection)	Moderate	Y	Y
21	33	Collapse consolidation (possible pneumonia)	Severe	N	Y
22	78	Chest infection	Severe	Y	Y
23	26	Cough	Moderate	N	N
24	61	Coughed purulent sputum	Mild	N	Y
25	27	Croup-like cough	Severe	Y	N
26	27	Cough (croupy-like)	Moderate	N	N
27	78	Purulent sputum	Mild	N	N
28	57	Purulent mucus	Mild	N	N
29	57	Purulent mucus	Mild	N	Y
30	78	Haemoptysis (Blood in sputum)	Mild	N	N
31	78	Haemoptysis (Blood in sputum)	Mild	N	N
32	42	Loin pain, pyrexia and rigors	Severe	Y	N
33	8	Pyrexia	Severe	N	N

Narrative Number	Patient Number	Indication	Severity	Serious Y/N	SABI Y/N
34	8	Hip pain	Severe	N	N
35	8	Right knee pain	Severe	N	N
36	8	Right ankle pain	Severe	N	N
37	8	Hip pain	Severe	N	N
38	8	Very painful shoulder	Severe	N	N
39	8	Joint pains	Severe	N	N
40	8	Severe pain in leg joints	Severe	Y	N
41	35	Diarrhoea/infection GI	Mild	N	N
42	58	Perianal abscess	Severe	Y	Y
43	18	Abscess	Moderate	N	Y
44	18	Pneumonia (cough)	Moderate	N	Y

Narrative number: 01

Patient number: **73**
Patient initials: [redacted]
Gender: **F**
Date of birth: [redacted] **1954**
Weight: **89.1 kg**
Centre: **Leicester Royal Infirmary, UK**
Diagnosis: **CVID**
Infection: **Chest infection**
Severity: **Severe**
Serious: **Yes (hospitalised)**
Dates: **02 April 2002 – 16 April 2002**

This is the same patient as in Narratives 02 and 03.

This 48-year-old female with CVID had chronic obstructive pulmonary disease (COPD) and emphysema. She had been using domiciliary oxygen on demand for several years (since the 1990s). She had a history of repeated chest infections. In addition, she had been using nebulised salbutamol 2.5 mg x5/day since 1975, supplemented with nebulised ipratropium 500 mg *bd* and nebulised saline 5 mL x5/day, both since the 1980s.

Other inhaled treatment consisted of fluticasone propionate 1.0 mg *bd* and terbutaline sulphate 250 µg *prn qds.*

Oral therapy was co-amilofruse 5/40 *bd* since the 1980s; prednisolone 5 mg *od* since 1980s; Sandocal 400 (calcium lactate gluconate with calcium carbonate), taken since 2000, two daily; and alendronate 70 mg once weekly since May 2001.

Soon after entering Stage 2 of the study, she developed a severe chest infection for which she was hospitalised.

She was given amoxicillin 250 mg *tds* from 2-4 April 2002 when it was changed to ciprofloxacin 750 mg *bd*. After two doses of this antibiotic, treatment was changed on 5 April 2002 to intravenous ceftazidime 2 g, which was continued until 16 April 2002. The infection was resolved. She had received nebulised N-acetyl cysteine 20% *bd* from 10-15 April. Prednisolone was reduced from 10 mg to 5 mg *od* on 18 April 2002 after the presumed infection had resolved.

No imaging details, blood test results or clinical symptoms or signs are available to further define the patient's severe "chest infection".

BPL Comment: This patient clearly has severe, chronically compromised respiratory function, which is complicated by the anatomical abnormalities and expected functional reversibility implied by the inhaled treatments. It is possible that this was an exacerbation of COPD, however, erring on the worst case, it is probably wise to regard this infection as a possible pneumonia (i.e. serious acute bacterial infection).

Narrative number: 02
Patient number: 73
Patient initials: [redacted]
Gender: F
Date of birth: [redacted] 1954
Weight: 89.1 kg
Centre: Leicester Royal Infirmary, UK
Diagnosis: CVID
Infection: Chest infection
Severity: Severe
Serious: Yes (hospitalised)
Dates: 05 May 2002 – 17 May 2002

This is the same patient as in Narratives 01 and 03.

This 48-year-old female with CVID had chronic obstructive pulmonary disease (COPD) and emphysema. She had been using domiciliary oxygen on demand for several years (since the 1990s). She had a history of repeated chest infections.

In addition, she had been using nebulised salbutamol 2.5 mg x 5/day since 1975, supplemented with nebulised ipratropium 500 mg *bd* and nebulised saline 5 mL x 5/day, both since the 1980s.

Other inhaled treatment consisted of fluticasone propionate 1.0 mg *bd* and terbutaline sulphate 250 µg *prn qds*.

Oral therapy was co-amilofruse 5/40 *bd* since the 1980s; prednisolone 5 mg *od* since 1980s; Sandocal 400 (calcium lactate gluconate with calcium carbonate), two daily; and alendronate 70 mg once weekly.

Nineteen days after recovering from the infection mentioned above (see narrative 01), she developed another chest infection. On 5 May 2002, she developed pyrexia which rose to >37°C that evening. On 7 May she was started on intravenous ceftazidime 2 g *bd* and gentamicin 600 mg *od*. Gentamicin was stopped on 12 May and ceftazidime on 16 May. She had been started on nebulised colomycin 1 M units *bd* which was stopped in September 2002.

No imaging details, blood test results or clinical symptoms or signs are available to further define the patient's severe "chest infection".

BPL Comment: This patient clearly has severe, chronically compromised respiratory function, which is complicated by the anatomical abnormalities and expected functional reversibility implied by the inhaled treatments. It is possible that this was an exacerbation of COPD, however, erring on the worst case, it is probably wise to regard this infection as a possible pneumonia (i.e. serious acute bacterial infection).

CONFIDENTIAL

Narrative number: **03**
Patient number: **73**
Patient initials: [redacted]
Gender: **F**
Date of birth: [redacted] **1954**
Weight: **89.1 kg**
Centre: **Leicester Royal Infirmary, UK**
Diagnosis: **CVID**
Infection: **Pneumonia**
Severity: **Severe**
Serious: **Yes (hospitalised)**
Dates: **15 November 2002 – 26 November 2002**

This is the same patient as in Narratives 01 and 02.

This 48-year-old female with CVID had chronic obstructive pulmonary disease (COPD) and emphysema. She had been using domiciliary oxygen on demand for several years (since the 1990s). She had a history of repeated chest infections.

In addition, she had been using nebulised salbutamol 2.5 mg x 5/day since 1975, supplemented with nebulised ipratropium 500 mg *bd* and nebulised saline 5 mL x 5/day, both since the 1980s.

Other inhaled treatment consisted of fluticasone propionate 1.0 mg *bd* and terbutaline sulphate 250 µg *prn qds*.

Oral therapy was co-amilofruse 5/40 *bd* since the 1980s; prednisolone 5 mg *od* since 1980s; Sandocal 400 (calcium lactate gluconate with calcium carbonate), two daily; and alendronate 70 mg once weekly.

From 4 November 2002, she became more dyspnoeic than usual and started oral amoxicillin on 6 November, although her temperature was 36.7°C, and continued until 13 November. She was started on intravenous amoxicillin 500 mg *tds* on 15 November 2002, which was switched to the oral route on 18 November and continued until resolution eight days later (26 November). Oral erythromycin 500 mg *tds* was also given from 15-26 November 2002.

No imaging details, blood test results or clinical symptoms or signs are available to further define the patient's severe "pneumonia".

BPL Comment: This patient clearly has severe, chronically compromised respiratory function, which is complicated by the anatomical abnormalities and expected functional reversibility implied by the inhaled treatments. It is possible that this was an exacerbation of COPD, however, erring on the worst case, it is probably wise to regard this infection as a probable pneumonia (i.e. serious acute bacterial infection).

CONFIDENTIAL

Narrative number: **04**
Patient number: **75**
Patient initials: [redacted]
Gender: **F**
Date of birth: [redacted] **1962**
Weight: **72.2 kg**
Centre: **Leicester Royal Infirmary, UK**
Diagnosis: **Specific pneumococcal antibody deficiency**
Infection: **Chest infection**
Severity: **Severe**
Serious: **Yes (hospitalised)**
Dates: **21 February 2003 – 25 March 2003**

This is the same patient as in Narratives 05, 08 and 09.

This 40-year-old female with specific pneumococcal antibody deficiency, chronic obstructive pulmonary disease (COPD) and bronchiectasis had been on nebulised salbutamol 2.5 mg *bd* and ipratropium 500 µg *bd* since 1996.

She also suffered with depression and sleep disturbance since September 2001 and had been taking paroxetine 20 mg *od* until 29 August 2002, before continuing with the same dose again from 12 December 2002 onwards. She had also been taking paracetamol and codeine phosphate since 11 August 2001 for post-thrombotic pain.

She developed a cough on 10 February 2003 with later wheeze and body temperature recorded as 35.7°C. However, she did not have any time off work. On 21 February 2003 she was diagnosed with a severe chest infection and acute asthma, hospitalised and given intravenous aminophylline 250 mg, nebulised salbutamol and was started on intravenous ceftriaxone 2 g *od*, which was continued until 2 March. She was started on continuous infusion of aminophylline 500 mg/24 hours, which was continued until 12 March. Intravenous imipenem 500 mg *qds* was introduced on 8 March and changed to oral ciprofloxacin 750 mg *bd* on 14 March together with clarithromycin, both continued until 24 March. Oral fluconazole 400 mg *od* was given for 5 days from 12-17 March. On 25 March 2003, symptoms of the chest infection had improved but the asthmatic symptoms continued requiring ongoing therapy.

No imaging details, blood test results or clinical symptoms or signs are available to further define the patient's severe "chest infection with acute asthma".

BPL Comment: This patient with asthma (COPD) and bronchiectasis developed an acute respiratory condition, which needed intensive bronchodilator therapy. Despite a wide range of broad-spectrum antibiotics, ceftriaxone, imipenem, clarithromycin and ciprofloxacin, she made a slow response. This patient's antibody deficiency is specifically described as pneumococcal. Therefore, this event is more likely to be related to the COPD/asthma rather than an acute bacterial infection. This event should not be regarded as a serious acute bacterial infection.

Narrative number: 05
Patient number: 75
Patient initials: [redacted]
Gender: F
Date of birth: [redacted] 1962
Weight: 72.2 kg
Centre: Leicester Royal Infirmary, UK
Diagnosis: Specific pneumococcal antibody deficiency
Infection: Exacerbation of bronchiectasis
Severity: Severe
Serious: Yes (hospitalised)
Dates: 12 May 2003 – 19 May 2003

This is the same patient as in Narratives 04, 08 and 09.

This 40-year-old female with specific pneumococcal antibody deficiency, chronic obstructive pulmonary and bronchiectasis had been on nebulised salbutamol 2.5 mg *bd* and ipratropium 500 µg *bd* since 1996.

She also suffered with depression and sleep disturbance since September 2001 and had been taking paroxetine 20 mg *od* until 29 August 2002, before continuing with the same dose again from 12 December 2002 onwards. She had also been taking paracetamol and codeine phosphate since 11 August 2001 for post-thrombotic pain.

She had a pain in her side (position not specified) and her chest felt sore for about a week in early May 2003. On 12 May she was wheezy and diagnosed with an exacerbation of bronchiectasis, hospitalised and given intravenous aminophylline 500 mg/24 hours by continuous infusion, which was continued until 19 May, and she also was prescribed prednisolone 20 mg *od* (this was continued for about 4 months, until 15 September). Oral co-amoxiclav 625 mg *tds* was started and continued for 2 weeks (until 26 May). She required analgesia for chest pain from 12 – 28 May with different products, including tramadol 100 mg *qds*, dihydrocodeine 30 mg *prn* and benzoxazocine 30 mg *tds*.

No imaging details, blood test results or clinical symptoms or signs are available to further define the patient's severe "exacerbation of bronchiectasis".

BPL Comment: This patient with asthma (COPD), bronchiectasis and specific pneumococcal antibody deficiency developed chest pain and wheeze that was later diagnosed as exacerbation of bronchiectasis. She responded slowly to intravenous aminophylline, prednisolone and co-amoxiclav. Chest pain is not necessarily caused by acute severe bacterial infection especially in a patient with COPD in exacerbation. Therefore, this event is more likely to be related to the COPD/asthma rather than a serious acute bacterial infection. This event should not be regarded as a serious acute bacterial infection.

Narrative number: **06**
Patient number: **23**
Patient initials: [redacted]
Gender: **F**
Date of birth: [redacted] **1944**
Weight: **68.0 kg**
Centre: **Papworth Hospital, UK**
Diagnosis: **CVID**
Infection: **Chest Infection**
Severity: **Severe**
Serious: **Yes (hospitalised)**
Dates: **NK October 2004 – 16 November 2004**

This 60-year-old female with CVID had chronic asthma and bronchiectasis and had a history of repeated chest infections.

She had been on nebulised salbutamol 5 mg *qid* supplemented with nebulised ipratropium 500 µg *qid* and inhaled salmeterol 50 µg *bd* for an unspecified length of time. She had also received nebulised budesonide 1 mg *bd* since 1999, oral montelukast 10 mg daily since 1998, oral theophyline 400 mg *nocte* since 1998 and inhaled beclometasone 50 µg *bid* since 1997.

She had recently suffered a moderate chest infection, lasting from 29 September 2004 until 12 October 2004, which was treated with oral ciprofloxacin 750 mg *bd*. At an unspecified date later in October 2004 (between 20 and 27 October), she developed a severe chest infection. On 08 November 2004 it was decided to admit her to hospital as an elective (i.e. non-emergency) admission for IV antibiotics.

She was given intravenous tobramycin 120 mg *tds* from 08 November until 11 November, and the dose was then changed to 140 mg *bd*, until the event resolved on 16 November 2004. She was also given ceftazidime 2 g *tds* from 08 November to 16 November. Around this time her body temperature ranged from 36.4 (3 November 2004) to 36.8°C (11 Novenver 2004).

Blood tests performed on 11 November gave results for lactate dehydrogenase of 161 U/L, antichymotrypsin of 0.5 g/L and haptoglobin of 1.2 g/L. Tobramycin levels were measured on 13 November and were 1.0 mg/L pre-infusion and 8.1 mg/L post-infusion.

No imaging details or clinical symptoms or signs are available to further define the patient's severe "chest infection".

BPL comment: This patient's severe exacerbation was more likely to be infective in origin on the basis of reasonably prompt response to ceftazidime (from 8-16 November) and tobramycin (8-16 November). She was not given oral or intravenous corticosteroids. This should be regarded as a serious acute bacterial infection.

Narrative number: 07
Patient number: 22
Patient initials: [redacted]
Gender: F
Date of birth: [redacted] 1947
Weight: 73.5 kg
Centre: Papworth Hospital, UK
Diagnosis: CVID
Infection: Chest Infection
Severity: Severe
Serious: No
Dates: 12 October 2002 – 29 October 2002

This is the same patient as in Narratives 10 and 12.

This 54-year-old female with CVID had chronic asthma, emphysema and bronchiectasis, and had suffered prior pulmonary emboli. She had a number of chest infections during the study. She had been using aminophylline 450 mg daily since 1993 and 225 mg *bd* since 2002. She also received inhaled beclometasone 400 µg *tds* for an unspecified length of time, until December 2001 when the dose was changed to 1 mg *bd*. Treatment stopped in May 2002. Formoterol fumate was given at 12 µg *bd* and inhaled salbutamol 2.5 mg / ipratropium bromide given *tds*, both for an unspecified length of time.

This patient had also been taking oral phenindione 275 mg for previous pulmonary emboli for an unspecified length of time before the study and through the study until completion. From 19 July 2001 until the end of the study this patient was receiving oral hyoscine 10 mg *prn* intermittently for pleuritic chest pain.

The patient developed a severe but non-serious chest infection on 12 October 2002. This was treated with oral ciprofloxacin 750 mg *tds* from 12-31 October 2002, and oral prednisolone 25 mg from 17-31 October 2002. The patient recovered on 29 October 2002 with no residual effects. The maximum body temperature during this time was 37.3°C.

No imaging details, blood test results or clinical symptoms or signs are available to further define the patient's severe "chest infection".

BPL comment: This patient with longstanding asthma and bronchiectasis had an exacerbation which was poorly responsive to high dose ciprofloxacin but improved, albeit slowly, after moderate dose oral steroids. The patient also had marginal pyrexia. It is more likely that this event was related to the asthma. It should not be regarded as a serious acute bacterial infection.

Narrative number: 08
Patient number: 75
Patient initials: [redacted]
Gender: F
Date of birth: [redacted] 1962
Weight: 72.2 kg
Centre: Leicester Royal Infirmary, UK
Diagnosis: Specific antibody deficiency
Infection: Chest Infection
Severity: Moderate
Serious: Yes (hospitalised)
Dates: 21 October 2002 – 05 November 2002

This is the same patient as in Narratives 04, 05 and 09.

This 40-year-old female with specific antibody deficiency suffered from chronic obstructive pulmonary disease (COPD), recurrent respiratory tract infections, bronchiectasis and prior left lower lobe collapse. Inhaled treatment consisted of ipratropium 500 µg *bd* and salbutamol 2.5 mg *bd*, both since 1996 and fluticasone propionate 250 µg *bd* since 2001.

On 21 October 2002 she was admitted to hospital with a chest infection, which was treated with iv ceftriaxone 2 g once daily between 24-28 October and oral prednisolone 30 mg once daily between 24 October and 01 November 2002. Her temperature, recorded twice during this time, was 36.7°C. The patient took 9 days off work, returning to work on 04 November 2002. The presumed infection resolved on 05 November 2002.

No imaging details, blood test results or clinical symptoms or signs are available to further define the patient's moderate "chest infection".

BPL comment: This patient with COPD was treated with a broad spectrum antibiotic intravenously and moderately high dose oral steroids, at which time she was able to be discharged. She was apyrexial throughout. Exacerbation of COPD is the most likely reason. The event should not be regarded as a serious acute bacterial infection.

CONFIDENTIAL

Narrative number: **09**
Patient number: **75**
Patient initials: [redacted]
Gender: **F**
Date of birth: [redacted] **1962**
Weight: **72.2 kg**
Centre: **Leicester Royal Infirmary, UK**
Diagnosis: **Specific antibody deficiency**
Infection: **Chest Infection**
Severity: **Severe**
Serious: **Yes (hospitalised)**
Dates: **21 February 2003 – 25 March 2003**

This is the same patient as in Narratives 04, 05 and 08.

This 40-year-old female with specific antibody deficiency suffered from recurrent respiratory tract infections, bronchiectasis and left lower lobe collapse. Inhaled treatment consisted of ipratropium 500 µg *bd* and salbutamol 2.5 mg *bd*, both since 1996 and fluticasone propionate 250 µg *bd* since 2001.

On 21 February 2003 this patient was admitted to hospital with a chest infection and asthma. She was treated with intravenous ceftriaxone 2 g once daily between 21 February – 02 March, intravenous imipenem 500 mg *qds* from 08-13 March, oral ciprofloxacin 750 mg *bd* and clarithromycin 500 mg *bd*, both from 14-24 March. Her body temperature did not rise above 36.9°C.

She also received intravenous aminophylline 250 mg once on 21 February 2003 and a continuous infusion of 500 mg aminophylline/24 hours from 22 February – 12 March 2003 for associated asthmatic symptoms.

She was discharged from hospital on 25 March 2003 after the presumed infection had resolved. However, the patient failed to return to work due to residual effects of this event, and occurrence of further adverse events, until the patient's withdrawal from the study on 09 March 2004.

No imaging details, blood test results or clinical symptoms or signs are available to further define the patient's severe "chest infection".

BPL comment: This patient with COPD on intensive inhaled therapy had a prolonged period of hospitalization. She had several sequential and concomitant broad spectrum antibiotics without dramatic improvement. She was apyrexial throughout. In hospital she had intensive anti-asthma therapy; exacerbation of her COPD is the more likely reason for the event. It should not be regarded as a serious acute bacterial infection.

Narrative number: 10
Patient number: 22
Patient initials: [redacted]
Gender: F
Date of birth: [redacted] 1947
Weight: 73.5 kg
Centre: Papworth Hospital, UK
Diagnosis: CVID
Infection: Exacerbation of Bronchiectasis
Severity: Severe
Serious: Yes (hospitalised)
Dates: 27 July 2002 – 12 August 2002

This is the same patient as in Narratives 07 and 12.

This 54-year-old female with CVID had chronic asthma, emphysema and bronchiectasis, and had suffered prior pulmonary emboli. She had a number of chest infections during the study. She had been using aminophylline 450 mg daily since 1993 and 225 mg *bd* since 2002. She also received inhaled beclometasone 400 µg *tds* for an unspecified length of time, until December 2001 when the dose was changed to 1 mg *bd*. Treatment stopped in May 2002. Formoterol fumate was given at 12 µg *bd* and inhaled salbutamol 2.5 mg / ipratropium bromide given *tds*, both for an unspecified length of time.
This patient had also been taking oral phenindione 275 mg for previous pulmonary emboli for an unspecified length of time before the study and through the study until completion. From 19 July 2001 until the end of the study this patient was receiving oral hyoscine 10 mg *prn* intermittently for pleuritic chest pain.

This patient had recently suffered a moderate chest infection, from 13 June until 11 July 2002, accompanied by chest pain from 04 to11 July 2002. She was then hospitalised with a severe chest infection (see Narrative 12), and was discharged on 26 July 2002 with IV antibiotics for home administration (cefuroxime 750 mg *tds* until 01 August 2002). After feeling some improvement, pleuritic chest pain resumed and the patient was re-admitted to hospital on 27 July 2002. From 26 July to 10 August 2002 she took oral prednisolone for associated asthma (25 mg from 26 to 29 July 2002, 20 mg from 30 July to 03 August 2002 and 15 mg from 04 to 10 August 2002). Treatment with intravenous cefuroxime 750 mg *tds* was recommenced from 07 to 14 August 2002.

The patient was discharged on 12 August 2002 after the patient had recovered from the presumed infection. She returned to hospital at fortnightly intervals until 17 September 2002 for chest reviews, during which time infusions of Subgam® were interrupted and the patient received intravenous human normal immunoglobulin (Vigam® Liquid) to maintain serum IgG levels.

No imaging details, blood test results or clinical symptoms or signs are available to further define the patient's severe "exacerbation of bronchiectasis".

BPL comment: This patient with COPD and bronchiectasis complicated by previous pulmonary emboli had intravenous antibiotics but was readmitted and treated with reducing doses of oral steroids and further doses of the intravenous antibiotics. It is more likely that the event was exacerbation of COPD. It should not be regarded as a serious acute bacterial infection.

Narrative number: 11
Patient number: 83
Patient initials: [redacted]
Gender: M
Date of birth: [redacted] 2001
Weight: 10.1 kg
Centre: Great Ormond Street Hospital, London, UK
Diagnosis: CD40 Ligand Deficiency
Infection: Pneumonia
Severity: Mild
Serious: No
Dates: 30 May 2004 – 31 May 2004

This 3-year-old male with CD40 ligand deficiency had been in general good health, with the exception of teething, coughs, colds, rhinorrhoea and mild gastrointestinal symptoms. In June 2003 he started taking oral ursodeoxycholic acid 200 mg to promote bile flow in prophylactic treatment of CD40 ligand deficiency, and he had recently started on oral co-trimoxazole 600 mg *od* for Pneumocystis carinii pneumonia prophylaxis and oral paramomycin 200 mg *od* for Cryptosporidium prophylaxis.

On 30 May 2004 the patient reported pneumonia and a body temperature of 37.5°C, which was treated with paracetamol *prn* (dose unknown) on 30 May and oral co-amoxiclav *tds* (dose unknown) from 30 May until 04 June 2004. The patient recovered without residual effects on 31 May 2004. The patient's body temperature on 07 and 14 June had returned to normal at 36.5°C.

No imaging details, blood test results or clinical symptoms or signs are available to further define the patient's mild "pneumonia".

BPL comment: This young boy had a low grade pyrexia but nevertheless was diagnosed with pneumonia and treated uneventfully with co-amoxiclav as an outpatient. Although there is no objective data or clinical management data to confirm the report of pneumonia, it would be safer to regard this as a serious acute bacterial infection.

Narrative number: 12
Patient number: 22
Patient initials: [redacted]
Gender: F
Date of birth: [redacted] 1947
Weight: 73.5 kg
Centre: Papworth Hospital, UK
Diagnosis: CVID
Infection: Chest Infection
Severity: Severe
Serious: Yes (hospitalised)
Dates: 15 July 2002 – 26 July 2002

This is the same patient as in Narratives 07 and 10.

This 54-year-old female with CVID had chronic asthma, emphysema and bronchiectasis, and had suffered prior pulmonary emboli. She had a number of chest infections during the study. She had been using aminophylline 450 mg daily since 1993 and 225 mg *bd* since 2002. She also received inhaled beclometasone 400 µg *tds* for an unspecified length of time, until December 2001 when the dose was changed to 1 mg *bd*. Treatment stopped in May 2002. Formoterol fumate was given at 12 µg *bd* and inhaled salbutamol 2.5 mg / ipratropium bromide given *tds*, both for an unspecified length of time. This patient had also been taking oral phenindione 275 mg for previous pulmonary emboli for an unspecified length of time before the study and through the study until completion. From 19 July 2001 until the end of the study this patient was receiving oral hyoscine 10 mg *prn* intermittently for pleuritic chest pain.

She had recently suffered a moderate chest infection, from 13 June until 11 July 2002, accompanied by chest pain from 04 to 11 July 2002. Both resolved with treatment. On 15 July she developed a severe chest infection, for which she was hospitalised on 18 July 2002. From 16 to 18 July she had been treated with oral clarithromycin 500 mg once daily, oral amoxicillin 500 mg *tds* and oral co-codamol 50 mg *tds*. She received oral tramadol 100 mg once (18 July 2002) and started oral prednisolone 30 mg daily from 18 July 2002 that was continued until 25 July 2002. She also started IV cefuroxime 750 mg *tds* until 01 August, plus she received physiotherapy. On 18 July her body temperature was 37.2°C.

The patient was discharged on 26 July 2002 with IV antibiotics for home administration (cefuroxime 750 mg *tds* until 01 August), but was re-hospitalised the following day with infective exacerbation of bronchitis (see Narrative 10). She also had asthmatic symptoms requiring ongoing therapy.

No imaging details, blood test results or clinical symptoms or signs are available to further define the patient's severe "chest infection".

BPL comment: This patient had severe pulmonary disease including asthma. The minimum increase in body temperature and the lack of effect from intensive antibiotic therapy suggests this is unlikely to be a serious acute bacterial infection. It is more likely to be an exacerbation of her longstanding asthma.

Narrative number: 13
Patient number: 58
Patient initials: [redacted]
Gender: M
Date of birth: [redacted] 1971
Weight: 83.3 kg
Centre: University Hospital of Wales, Cardiff, UK
Diagnosis: Specific Antibody Deficiency
Infection: Left Thumb Infection
Severity: Moderate
Serious: Yes (hospitalised)
Dates: 25 November 2002 – 09 December 2002

This is the same patient as in Narratives 14, 15, 16, 17 and 42.

This 30-year-old male with specific antibody deficiency had had bronchiectasis for many years prior to the study. He also suffered from recurrent skin abscesses and severe eczema as part of the Hyper-IgE (Job's) syndrome. He had been on oral flucloxacillin 500 mg *qds* for chest prophylaxis, fluconazole 200 mg *od*, and co-codamol 30/500 *qds prn* for some years. Inhaled treatment consisted of salmeterol 2 puffs *bd*, fluticasone propionate 2 puffs *bd*, both for many years, and salbutamol 2 puffs *prn* since June 2001.

In September 2001 his dose of inhaled fluticasone propionate was increased to 4 puffs *bd*. In January 2002 the dose of inhaled fluticasone propionate was changed to 250 µg *bd* and he was also started on oral flucloxacillin 500 mg *qds* for prophylaxis against chest infections. In April 2002 this was supplemented by nebulised colomycin 2 million units *bd*.

On 25 November 2002 the patient developed a moderate infection of the left thumb, which was reviewed by the chest physician on 29 November. At this time he was given oral ciprofloxacin 750 mg *tds*, which he took until 08 December; he also took morphine sulphate 10 mg *tds* between 28 November and 04 December. However, the condition worsened and he was hospitalised on 03 December in order to receive intravenous antibiotics (fusidic acid 500 mg *tds* and flucloxacillin 1 g *qds* between 04-09 December).

Oral analgesia consisted of morphine sulphate 10 mg *bd* and tramadol hydrochloride 100 mg *prn* from 04 December, and diclofenac 75 mg *bd* on 05 December, all of which were stopped on 09 December on the patient's discharge from hospital. These were supplemented with morphine sulphate 10 mg *prn* from 03 to 09 December. At this point the patient started a course of oral fusidic acid 250 mg *tds* until resolution ten days later (19 December 2002).

No imaging details, blood test results or clinical symptoms or signs are available to further define the patient's moderate "left thumb infection".

BPL comment: This patient developed a presumed bacterial infection of his left thumb. This resolved slowly with anti-staphylococcal antibiotics. This should be regarded as a serious acute bacterial infection (abscess, left thumb).

Narrative number: 14
Patient number: 58
Patient initials: [redacted]
Gender: M
Date of birth: [redacted] 1971
Weight: 83.3 kg
Centre: University Hospital of Wales, Cardiff, UK
Diagnosis: Specific Antibody Deficiency
Infection: Chest Infection
Severity: Moderate
Serious: Yes (hospitalised)
Dates: 03 September 2001 – 28 September 2001

This is the same patient as in Narratives 13, 15, 16, 17 and 42.

This 30-year-old male with specific antibody deficiency had had bronchiectasis for many years prior to the study. He also suffered from recurrent skin abscesses and severe eczema as part of the Hyper-IgE (Job's) syndrome. He had been on oral flucloxacillin 500 mg *qds* for chest prophylaxis, fluconazole 200 mg *od*, and co-codamol 30/500 *qds prn* for some years. Inhaled treatment consisted of salmeterol 2 puffs *bd*, fluticasone propionate 2 puffs *bd*, both for many years, and salbutamol 2 puffs *prn* since June 2001.

On 03 September 2001, shortly after entry to the study, this patient was admitted to hospital with a chest infection that was failing to respond to long-term oral antibiotics. The patient received oral ciprofloxacin 750 mg *bd* from 11 to 14 September, followed by amoxicillin 500 mg *tds* from 14 to 29 September. Intravenous therapy was ceftazidine 2 g *tds* and gentamicin 340 mg *od*, both from 14 to 28 September. Inhaled bronchodilator treatment consisted of nebulised salbutamol 5 mg *qds*, fluticasone propionate 4 puffs *bd*, and salmeterol 2 puffs *bd*, all from 14 September 2001 to 03 January 2002. The patient was discharged from hospital on 17 September 2001.

Treatment with oral flucloxacillin 500 mg *qds* and oral fluconazole 200 mg *od* was interrupted during hospitalization, both of which were resumed from 29 September 2001. Temperatures recorded during the patient's period of hospitalization were in the range 36.6-36.8°C. He had 7 days off work during this time.

No imaging details, blood test results or clinical symptoms or signs are available to further define the patient's moderate "chest infection".

BPL comment: The lack of pyrexia and slow response to intensive antibiotic therapy in this patient also receiving treatment for asthma suggests this is more likely to be an exacerbation of asthma than a serious acute bacterial infection.

Narrative number: 15
Patient number: 58
Patient initials: [redacted]
Gender: M
Date of birth: [redacted] 1971
Weight: 83.3 kg
Centre: University Hospital of Wales, Cardiff, UK
Diagnosis: Specific Antibody Deficiency
Infection: Chest Infection
Severity: Severe
Serious: Yes (hospitalised)
Dates: 14 May 2004 – 14 June 2004

This is the same patient as in Narratives 13, 14, 16, 17 and 42.

This 30-year-old male with specific antibody deficiency had had bronchiectasis for many years prior to the study. He also suffered from recurrent skin abscesses and severe eczema as part of the Hyper-IgE (Job's) syndrome. He had been on oral flucloxacillin 500 mg *qds* for chest prophylaxis, fluconazole 200 mg *od*, and co-codamol 30/500 *qds prn* for some years. Inhaled treatment consisted of salmeterol 2 puffs *bd*, fluticasone propionate 2 puffs *bd*, both for many years, and salbutamol 2 puffs *prn* since June 2001.

In September 2001 his dose of inhaled fluticasone propionate was increased to 4 puffs *bd*. In January 2002 the dose of inhaled fluticasone propionate was changed to 250 µg *bd* and he was also started on oral flucloxacillin 500 mg *qds* for prophylaxis against chest infections. In April 2002 this was supplemented by nebulised colomycin 2 million units *bd*.

On 14 May 2004 the patient developed a severe chest infection, which was treated with oral ciprofloxacin 1 g *bd* from 26 May to 02 June and oral prednisolone 40 mg *od* from 26 May to 01 June. From 19 May he stopped work, unable to return during the course of the presumed infection. His condition worsened and on 08 June 2004 he was admitted to hospital for insertion of a PICC line and administration of intravenous antibiotics (ceftazidime 2 g *tds* and gentamicin 120 mg *tds*). He also received nebulised salbutamol 5 mg *qds*.

The patient was discharged from hospital on 11 June 2004, continuing intravenous antibiotic therapy (gentamicin 120 mg *tds*), nebulised salbutamol (5 mg *qds*) and ceftazidime (2 g *tds*) until eleven days later (25 June 2004).

No imaging details, blood test results or clinical symptoms or signs are available to further define the patient's severe "chest infection".

BPL comment: The pattern of this patient's acute episode suggests it is more likely to be related to asthma than to a serious acute bacterial infection.

CONFIDENTIAL

Narrative number: **16**
Patient number: **58**
Patient initials: [redacted]
Gender: **M**
Date of birth: [redacted] **1971**
Weight: **83.3 kg**
Centre: **University Hospital of Wales, Cardiff, UK**
Diagnosis: **Specific Antibody Deficiency**
Infection: **Chest Infection**
Severity: **Moderate**
Serious: **Yes (hospitalised)**
Dates: **29 June 2004 – 14 September 2004**

This is the same patient as in Narratives 13, 14, 15, 17 and 42.

This 30-year-old male with specific antibody deficiency had had bronchiectasis for many years prior to the study. He also suffered from recurrent skin abscesses and severe eczema as part of the Hyper-IgE (Job's) syndrome. He had been on oral flucloxacillin 500 mg *qds* for chest prophylaxis, fluconazole 200 mg *od*, and co-codamol 30/500 *qds prn* for some years. Inhaled treatment consisted of salmeterol 2 puffs *bd*, fluticasone propionate 2 puffs *bd*, both for many years, and salbutamol 2 puffs *prn* since June 2001.

In September 2001 his dose of inhaled fluticasone propionate was increased to 4 puffs *bd*. In January 2002 the dose of inhaled fluticasone propionate was changed to 250 µg *bd* and he was also started on oral flucloxacillin 500 mg *qds* for prophylaxis against chest infections. In April 2002 this was supplemented by nebulised colomycin 2 million units *bd*.

This patient had recently been admitted to hospital with a severe chest infection (Narrative 15), and was discharged on 11 June 2004 with intravenous antibiotic therapy (gentamicin 120 mg *tds*), nebulised salbutamol (5 mg *qds*) and ceftazidime (2 g *tds*) until eleven days later (25 June 2004).

From 25 June 2004 he started salbutamol 2 puffs *prn* to ease difficulty in breathing. However, he recorded wheezing and phlegm as symptoms of a mild chest infection on 29 June, which gradually progressed to a moderate infection. From 04 July 2004 he stopped work, unable to return during the course of the presumed infection. He was given oral antibiotics ciprofloxacin 750 mg *bd* from 01 to 21 July, followed by oral prednisolone in decreasing doses (30 mg *od* from 07 to 10 August, 20 mg *od* from 11 to 13 August and 10 mg *od* from 14 to 16 August). However, the condition worsened further, resulting in his hospitalization on 10 September.

While in hospital the patient received oral ciprofloxacin 750 mg *bd* from 10 to 01 September 2004, supplemented with intravenous gentamicin 120 mg *tds* and aztreonam 2 g *tds*, which were continuing at the patient's completion of the study on 01 October 2004. He also received nebulised salbutamol 5 mg

qds from 10 to 14 September 2004. He was discharged from hospital on 14 September 2004 on resolution of the presumed infection.

No imaging details, blood test results or clinical symptoms or signs are available to further define the patient's moderate "chest infection".

BPL comment: This patient's acute condition apparently improved with 4 days of intravenous antibiotics such that he was discharged from hospital with continuing treatment. This should be regarded as a serious acute bacterial infection.

Narrative number: 17
Patient number: 58
Patient initials: [redacted]
Gender: M
Date of birth: [redacted] 1971
Weight: 83.3 kg
Centre: University Hospital of Wales, Cardiff, UK
Diagnosis: Specific Antibody Deficiency
Infection: Increased Production Purulent Sputum (Chest Infection)
Severity: Moderate
Serious: Yes (hospitalised)
Dates: 28 November 2001 – 20 December 2001

This is the same patient as in Narratives 13, 14, 15, 16, and 42.

This 30-year-old male with specific antibody deficiency had had bronchiectasis for many years prior to the study. He also suffered from recurrent skin abscesses and severe eczema as part of the Hyper-IgE (Job's) syndrome. He had been on oral flucloxacillin 500 mg *qds* for chest prophylaxis, fluconazole 200 mg *od*, and co-codamol 30/500 *qds prn* for some years. Inhaled treatment consisted of salmeterol 2 puffs *bd*, fluticasone propionate 2 puffs *bd*, both for many years, and salbutamol 2 puffs *prn* since June 2001.

In September 2001 this patient's dose of inhaled fluticasone propionate was increased to 4 puffs *bd*. He had suffered from increased Pseudomonas aeruginosa infected sputum production from 20 October 2001, and visited the hospital on 22 October for insertion of an IV line and commencement of intravenous antibiotics (gentamicin 300 mg *od* and ceftazidine 2 g *tds*) to be self-administered at home. These were continued until 05 November 2001. A sputum specimen on 07 December 2001 indicated there was continued heavy growth of *Pseudomonas aeruginosa*.

On 28 November 2001, this patient was admitted to hospital with a chest infection that was failing to respond to long-term oral antibiotics. He received intravenous therapy consisting of gentamicin 340 mg *od* and ceftazidine 2 g *tds* from 07 to 20 December 2001.

He was discharged from hospital on 10 December 2001 and the event was considered resolved on 20 December 2001. From 28 November until 20 December the patient took 4 days off work. His maximum pre-infusion body temperature during this time was 36.5°C.

No imaging details, blood test results or clinical symptoms or signs are available to further define the patient's moderate "chest infection".

BPL comment: The heavy growth of Pseudomonas aeruginosa with purulent sputum indicates that this probably is a serious acute bacterial infection.

Narrative number: **18**
Patient number: **77**
Patient initials: [redacted]
Gender: **F**
Date of birth: [redacted] **1965**
Weight: **58.0 kg**
Centre: **Papworth Hospital, UK**
Diagnosis: **CVID**
Infection: **Chest Infection**
Severity: **Moderate**
Serious: **Yes (hospitalised)**
Dates: **12 November 2001 – 28 November 2001**

This 36-year-old female with CVID had a history of left lower lobe bronchiectasis. She had no history of medication immediately prior to the study, with the exception of IVIG.

On 09 November 2001, a few months after joining the study, this patient reported fever, chest infection and chest pains, which worsened until she was hospitalised with an exacerbation of her bronchiectasis, on 12 November 2001. She was treated with intravenous cefuroxime 750 mg *tds* until 15 November. She also received paracetamol 1 g *qds* from 12 to 20 November, supplemented with ibuprofen 400 mg *prn* from 13 to 15 November. On 13 November she had a full blood count and serum biochemistry test performed; all results were within the normal range, with the exception of CRP, for which a result of 25 mg/L was obtained (normal range 0-10 mg/L).

On her discharge from hospital on 16 November 2001 she was given oral clarithromycin, which she took until 28 November, when the presumed infection had resolved.

No imaging details, further blood test results or clinical symptoms or signs are available to further define the patient's moderate "chest infection".

BPL comment: This patient's acute condition was associated with fever and a raised CRP, but no leucocytosis. However, rapid response to intravenous cefuroxime suggests this should be regarded as a serious acute bacterial infection.

Narrative number: 19
Patient number: 27
Patient initials: [redacted]
Gender: M
Date of birth: [redacted] 1995
Weight: 16.1 kg
Centre: Birmingham Children's Hospital, UK
Diagnosis: IgG subclass deficiency
Infection: Chest Infection
Severity: Moderate
Serious: Yes (hospitalised)
Dates: 22 October 2001 – 29 October 2001

This is the same patient as in Narratives 25 and 26.

This 5-year-old male with IgG subclass deficiency had a history of asthma, coughs and repeated chest infections. He had been on oral co-trimoxazole 360 mg *bd*, inhaled fluticasone propionate 100 µg *bd* and inhaled salbutamol 200 µg *prn* since February 2001. This was supplemented with inhaled salmeterol 100 µg *bd* and beclometasone dipropionate 2 puffs *bd* since July 2001.

On 19 October 2001 this patient reported a sore throat, cough and temperature, and on 22 October was admitted to hospital with a moderate chest infection. He had a body temperature of 36.9°C on 26 October. The patient received oral co-amoxiclav 250/62 mg *tds* from 19 October until 26 October. During this time he took 6 days off school.

No imaging details, blood test results or clinical symptoms or signs are available to further define the patient's moderate "chest infection".

BPL comment: This patient's 'chest infection' associated with pyrexia normalized by one week after treatment with co-amoxiclav. This should be regarded as a serious acute bacterial infection.

CONFIDENTIAL

Narrative number: **20**
Patient number: **78**
Patient initials: [redacted]
Gender: **F**
Date of birth: [redacted] **1939**
Weight: **46.0 kg**
Centre: **Papworth Hospital, UK**
Diagnosis: **Specific Antibody Deficiency**
Infection: **(Pseudomonas) Chest Infection**
Severity: **Moderate**
Serious: **Yes (hospitalised)**
Dates: **21 February 2002 – 12 March 2002**

This is the same patient as in Narratives 22, 27, 30 and 31.

This 63-year-old female with specific antibody deficiency had a history of bronchiectasis and emphysema and had had a left lobectomy in 1999. She had received inhaled salbutamol/ipratropium bromide 120 µg *bd* and inhaled budesonide 200 µg 2 puffs *bd* since 1997. In April 2000 this was supplemented with nebulised salbutamol 2.5 mg *bd*. In August 2001 she started on nebulised gentamicin 80 mg *bd* as prophylaxis for *Pseudomonas aeruginosa*.

On 21 February 2002 she reported a moderate chest infection, which was treated with oral ciprofloxacin 500 mg *tds* until 28 February. Her condition worsened, however, and she was hospitalised on 28 February for intravenous antibiotic therapy (aztreonam 2 g *tds* and tobramycin 120 mg *tds*) and physiotherapy. She was discharged from hospital on 08 March but completed intravenous antibiotic therapy as an outpatient, until 12 March 2002, when the presumed infection had resolved.

No imaging details, blood test results or clinical symptoms or signs are available to further define the patient's moderate "(pseudomonas) chest infection".

BPL comment: This patient had a lobectomy for bronchiectasis, had emphysema and asthma. She responded within a reasonable period of time to intravenous antibiotics. This should be regarded as a serious acute bacterial infection.

Narrative number: **21**
Patient number: **33**
Patient initials: [redacted]
Gender: **Male**
Date of birth: [redacted] **1970**
Weight: **65.7 kg**
Centre: **St Helier Hospital**
Diagnosis: **CVID**
Infection: **Collapse Consolidation (possible pneumonia)**
Severity: **Severe**
Serious: **No**
Dates: **27 February 2001 – date unspecified**

This 31-year-old male with CVID had a history of recurrent bronchitis. He also suffered from splenomegaly, thrombocytopenia, thalassaemia and neutropenia. He had no history of medication immediately prior to the study, with the exception of IVIG.

The patient suffered from a moderate fever and headache on 25 and 26 February 2001, during which time he took one day off work, followed by a moderate cough from 27 February until 04 March 2001.

On 27 February 2001 the patient visited the hospital for his usual study infusion. During the visit he had a chest x-ray, which indicated that he had a collapse consolidation of the left lung. A CT scan was arranged. The investigator noted that bronchiectasis was likely and chest and sinus infections would continue at intervals regardless of all therapy. On the same day the patient had a full blood count and CRP performed. He had a white cell count of 3.2 x 10^9/L (normal range 3.5-10.0), mean cell volume of 74 fl (normal range 84-98), mean cell Hb of 24.1 pg (normal range 27.5-32.0), platelet count of 63 x 10^9/L (normal range 150-360), lymphocyte count of 0.8 x 10^9/L (normal range 1.0-3.5) and CRP result of 28.6 mg/L. All other laboratory parameters were within the normal range and a blood culture indicated no growth. His pre-infusion body temperature was 37.2°C. The patient was given oral co-amoxiclav 2 tablets *tds* from 27 February until 02 March to treat the fever and headache but was not hospitalised.

On 06 March 2001 his CRP had fallen to 12.8 mg/L. He had taken ½ day off work in the last week.

No imaging details, further blood test results or clinical symptoms or signs are available to further define the patient's severe "collapse consolidation (possible pneumonia)".

BPL comment: As this patient's X-ray following fever showed collapse consolidation of left lung with leucopenia but raised CRP, this should be regarded as a serious acute bacterial infection.

Narrative number: 22
Patient number: 78
Patient initials: [redacted]
Gender: F
Date of birth: [redacted] 1939
Weight: 46.0 kg
Centre: Papworth Hospital, UK
Diagnosis: Specific Antibody Deficiency
Infection: Chest Infection
Severity: Severe
Serious: Yes (hospitalised)
Dates: 06 April 2004 – 21 May 2004

This is the same patient as in Narratives 20, 27, 30 and 31.

This 63-year-old female with specific antibody deficiency had a history of bronchiectasis and emphysema and had had a left lobectomy in 1999. She had received nebulised salbutamol/ipratropium bromide 120 µg *bd* and inhaled budesonide 200 µg 2 puffs *bd* since 1997. In April 2000 this was supplemented with nebulised salbutamol 2.5 mg *bd*. In August 2001 she started on nebulised gentamicin 80 mg *bd* as prophylaxis for *Pseudomonas aeruginosa*.

On 25 March 2004 the patient had experienced exacerbation of bronchiectasis and sputum examination was positive for *Pseudomonas aeruginosa*.

On 06 April 2004 this patient was developed a severe chest infection. Intravenous treatment was aztreonam 2 g *tds* until 12 April, pipericillin/tazobactam 2 g / 250 mg from 13 to 20 April, colomycin 1 million units *tds* on 20 to 21 April, ciprofloxacin 600 mg *tds* from 22 April to 05 May, benzylpenicillin 400 mg *tds* from 20 April to 03 May and tobramycin 80 mg *tds* from 06 to 10 April, which was reduced to 100 mg *bd* from 11 April to 03 May.

On 07 May the patient was admitted to hospital, where she was given intravenous co-trimoxazole 1.4 g *bd* until 21 May and intravenous tobramycin 100 mg *tds* until 21 May. Long-term oral antibiotic therapy (azithromycin 250 mg 3 x per week) was started on 14 May. A single dose of nebulised colomycin 2 million units was given on 19 May. She was discharged from hospital on 21 May 2004, after the presumed infection had resolved.

No imaging details, blood test results or clinical symptoms or signs are available to further define the patient's severe "chest infection".

BPL comment: This patient had very severe pulmonary pathology such that colonization with Pseudomonas aeruginosa was likely. The exacerbation could be due to Pseudomonas aeruginosa but this is uncertain. Although she had a poor response to a wide range of antibiotics, including those effective in vitro against Pseudomonas aeruginosa, it is probably better to assume this was a serious acute bacterial infection.

CONFIDENTIAL

Narrative number: 23
Patient number: 26
Patient initials: [redacted]
Gender: M
Date of birth: [redacted] 1996
Weight: 19.7 kg
Centre: Birmingham Children's Hospital, UK
Diagnosis: X-linked antibody deficiency
Infection: Cough
Severity: Moderate
Serious: No
Dates: 30 October 2002 – ongoing at 26 January 2004

This 6-year-old male with X-linked antibody deficiency suffered from mild asthma but was not receiving any long-term medication, with the exception of eye ointment for conjunctivitis.

On 30 October 2002 the patient reported a moderate cough, which was treated with oral erythromycin 250 mg *bd* until 01 April 2003 and inhaled salbutamol 100 µg *bd* until 06 November 2002. On 04 April 2003 the patient started on long-term oral co-trimoxazole 720 mg *od* for infection prophylaxis, and took oral co-amoxiclav 125/31 mg *bd* from 13 to 20 November 2003 for cough and common cold. The cough was reported as continuing at the patient's completion of the study on 26 January 2004.

No imaging details, blood test results or clinical symptoms or signs are available to further define the patient's moderate "cough".

BPL comment: In view of the prolonged period of this cough and the poor response to antibiotics, it is more likely that the symptom is due to either asthma or to hyper-reactivity of the airways, possibly secondary to a prior infection. This event should not be regarded as a serious acute bacterial infection.

CONFIDENTIAL

Narrative number: 24
Patient number: 61
Patient initials: [redacted]
Gender: M
Date of birth: [redacted] 1990
Weight: 35.0 kg
Centre: Royal Preston Hospital, UK
Diagnosis: Specific antibody deficiency
Infection: Coughed Purulent Sputum
Severity: Mild
Serious: No
Dates: NK August 2002 – 25 August 2002

This 11-year-old male with specific antibody deficiency had a history of bronchiectasis and repeated chest infections. He had been on inhaled fluticasone propionate 250 µg *bd*, inhaled salbutamol 100 µg *prn* and oral montelukast 10 mg *od* since 1995.

The patient had recently (from 19 July to 18 August 2002) been on amoxicillin 250 mg *bd* for prophylaxis against infection.

On 14 August 2002 the patient reported a mild chest infection, and started oral co-amoxiclav 625 mg *bd* on 15 August. On approximately 20 August 2002 he started coughing up green sputum. This ceased on 25 August and co-amoxiclav was discontinued but on 28 August the patient visited the hospital still feeling unwell. A sputum sample was taken for culture and the patient was re-started on oral co-amoxiclav 625 mg (frequency of dosing not recorded). The sputum culture result was negative. The patient finished his course of antibiotics on 16 September and the chest infection was resolved at an unspecified date in September 2002.

The patient didn't report any days off school and the maximum pre-infusion body temperature recorded during this time was 37.2°C.

No imaging details, blood test results or clinical symptoms or signs are available to further define the patient's mild "coughed purulent sputum".

BPL comment: This patient was treated with anti-asthmatic treatment and prophylactic antibiotics. The acute episode was accompanied by purulent sputum so this should, on balance, be regarded as a serious acute bacterial infection.

Narrative number: 25
Patient number: 27
Patient initials: [redacted]
Gender: M
Date of birth: [redacted] 1995
Weight: 16.1 kg
Centre: Birmingham Children's Hospital, UK
Diagnosis: IgG subclass deficiency
Infection: Croup-like cough
Severity: Severe
Serious: Yes (hospitalised)
Dates: 03 December 2002 – 05 December 2002

This is the same patient as in Narratives 19 and 26.

This 6-year-old male with IgG subclass deficiency had a history of asthma, coughs and repeated chest infections. He had been on oral co-trimoxazole 360 mg *bd*, inhaled fluticasone propionate 100 µg *bd* and inhaled salbutamol 200 µg *prn* since February 2001. This was supplemented with inhaled salmeterol 100 µg *bd* and beclometasone dipropionate 2 puffs *bd* since July 2001.

He had also received oral acyclovir 400 mg *qds* for chickenpox prophylaxis from 05 November to 02 December 2002. He had recently experienced a moderate sore throat, fever, diarrhoea, common cold and abdominal pain, and severe cough, all of which resolved on 02 December 2002.

On 03 December 2002 the patient reported severe croup-like cough with associated severe asthma. He was given oral co-amoxiclav 250 mg *tds* from 03 to 12 December 2002. The patient was admitted to hospital on 04 December 2002where he was observed overnight before being discharged the following day. The patient took 3 days off school during this time but his pre-infusion body temperatures were all recorded as 'apyrexial'.

No imaging details, blood test results or clinical symptoms or signs are available to further define the patient's severe "croup-like cough".

BPL comment: This patient's asthma exacerbated and was associated with the croup-like cough. This should not be regarded as a serious acute bacterial infection.

CONFIDENTIAL

Narrative number: **26**
Patient number: **27**
Patient initials: [redacted]
Gender: **M**
Date of birth: [redacted] **1995**
Weight: **16.1 kg**
Centre: **Birmingham Children's Hospital, UK**
Diagnosis: **IgG subclass deficiency**
Infection: **Cough (croup-like)**
Severity: **Moderate**
Serious: **No**
Dates: **23 October 2003 – 28 October 2003**

This is the same patient as in Narratives 19 and 25.

This 6-year-old male with IgG subclass deficiency had a history of asthma, coughs and repeated chest infections. He had been on oral co-trimoxazole 360 mg *bd*, inhaled fluticasone propionate 100 µg *bd* and inhaled salbutamol 200 µg *prn* since February 2001. This was supplemented with inhaled salmeterol 100 µg *bd* and beclometasone dipropionate 2 puffs *bd* since July 2001.

On 23 October 2003 the patient reported a moderate croupy cough, common cold and sore throat. These symptoms were treated with oral co-amoxiclav 250 mg *tds* until 29 October 2003and oral prednisolone 20 mg *od* until 25 October 2003. On 28 October 2003 the patient was considered to have recovered from all three conditions. He took 5 days off school during this time but his pre-infusion body temperatures were all recorded as 'apyrexial'.

No imaging details, blood test results or clinical symptoms or signs are available to further define the patient's moderate "cough (croup-like)".

BPL comment: This patient's rapid response when given oral prednisolone (and oral co-amoxiclav) suggests this was more likely an exacerbation of asthma and not a serious acute bacterial infection.

Narrative number: 27
Patient number: 78
Patient initials: [redacted]
Gender: F
Date of birth: [redacted] 1939
Weight: 46.0 kg
Centre: Papworth Hospital, UK
Diagnosis: Specific Antibody Deficiency
Infection: Green Sputum
Severity: Mild
Serious: No
Dates: 21 January 2003 – 23 January 2003

This is the same patient as in Narratives 20, 22, 30 and 31.

This 63-year-old female with specific antibody deficiency had a history of bronchiectasis and emphysema and had had a left lobectomy in 1999. She had received nebulised salbutamol/ipratropium bromide 120 µg *bd* and inhaled budesonide 200 µg 2 puffs *bd* since 1997. In April 2000 this was supplemented with nebulised salbutamol 2.5 mg *bd*. In August 2001 she started on nebulised gentamicin 80 mg *bd* as prophylaxis for *Pseudomonas aeruginosa*.

On 21 January 2003 this patient reported green sputum accompanied by a mild headache. She took oral diclofenac 50 mg *prn* from 21 to 24 January 2003 to treat the headache. She recorded a body temperature on 23 January 2003 of 35.4°C.

No imaging details, blood test results or clinical symptoms or signs are available to further define the patient's mild "purulent sputum".

BPL comment: The available details of this mild episode, albeit associated with green sputum, suggest this was not a serious acute bacterial infection. No systemic antibiotics were introduced.

CONFIDENTIAL

Narrative number: 28
Patient number: 57
Patient initials: [redacted]
Gender: Male
Date of birth: [redacted] 1998
Weight: 15.5 kg
Centre: University Hospital of Wales, Cardiff, UK
Diagnosis: X-linked antibody deficiency
Infection: Green mucus
Severity: Mild
Serious: No
Dates: 06 April 2002 – 13 April 2002

This is the same patient as in Narrative 29.

This 5-year-old male with X-linked antibody deficiency had had a history of recurrent respiratory infections but had been well recently.

On 06 April 2002 the patient reported a mild cough and green mucus. From 08 to 13 April 2002 he took oral co-amoxiclav 5 mL *tds* for a chest infection.

No imaging details, blood test results or clinical symptoms or signs are available to further define the patient's mild "green mucus".

BPL comment: This mild lower respiratory infection, on the basis of purulent sputum, is unlikely to represent a serious acute bacterial infection. Therefore, this case should not be categorized as a serious acute bacterial infection.

Narrative number: 29
Patient number: 57
Patient initials: [redacted]
Gender: Male
Date of birth: [redacted] 1998
Weight: 15.5 kg
Centre: University Hospital of Wales, Cardiff, UK
Diagnosis: X-linked antibody deficiency
Infection: Purulent mucus
Severity: Mild
Serious: No
Dates: 20 April 2002 – 27 April 2002

This is the same patient as in Narrative 28.

This 5-year-old male with X-linked antibody deficiency had had a history of recurrent respiratory infections but had been well recently.

On 06 April 2002 he reported a mild cough and green mucus (Narrative 28). From 08 to 13 April 2002 he took oral co-amoxiclav 5 mL *tds* for a chest infection. On 20 April 2002 the patient again reported a mild cough and green mucus. The patient re-started on oral co-amoxiclav 5 mL *tds* for a chest infection on 22 April, until 27 April 2002 when the condition resolved.

No imaging details, blood test results or clinical symptoms or signs are available to further define the patient's mild "purulent mucus".

BPL comment: This patient had two episodes, or a single episode without full resolution leading to relapse. It is possible that this represents a serious acute bacterial infection as response to antibiotics was as might be expected.

CONFIDENTIAL

Narrative number: 30
Patient number: 78
Patient initials: [redacted]
Gender: F
Date of birth: [redacted] 1939
Weight: 46.0 kg
Centre: Papworth Hospital, UK
Diagnosis: Specific Antibody Deficiency
Infection: Hemoptysis
Severity: Mild
Serious: No
Dates: 07 April 2002

This is the same patient as in Narratives 20, 22, 27 and 31.

This 63-year-old female with specific antibody deficiency had a history of bronchiectasis and emphysema and had had a left lobectomy in 1999. She had received nebulised salbutamol/ipratropium bromide 120 µg *bd* and inhaled budesonide 200 µg 2 puffs *bd* since 1997. In April 2000 this was supplemented with nebulised salbutamol 2.5 mg *bd*. In August 2001 she started on nebulised gentamicin 80 mg *bd* as prophylaxis for *Pseudomonas aeruginosa*.

This patient had previously reported increased sputum production, starting in December 2001 and continuing until an unspecified date in 2002. Sputum samples were collected on 03 January 2002 and 15 January 2002 and both were positive for *Pseudomonas aeruginosa*. On 07 April 2002 she reported blood in sputum. It resolved the same day with no treatment. Her pre-infusion body temperature between January and April 2002 ranged from 35.4-36.8°C.

No imaging details, blood test results or clinical symptoms or signs are available to further define the patient's mild "haemoptysis".

BPL comment: An isolated episode of hemoptysis is not unexpected in patients with bronchiectasis. Although she had isolates of P. aeruginosa, these might well be commensals/colonizers in this type of patient. This should not be reported as a serious acute bacterial infection.

Narrative number: 31
Patient number: 78
Patient initials: [redacted]
Gender: F
Date of birth: [redacted] 1939
Weight: 46.0 kg
Centre: Papworth Hospital, UK
Diagnosis: Specific Antibody Deficiency
Infection: Blood in sputum
Severity: Mild
Serious: No
Dates: 20 May 2002

This is the same patient as in Narratives 20, 22, 27 and 30.

This 63-year-old female with specific antibody deficiency had a history of bronchiectasis and emphysema and had had a left lobectomy in 1999. She had received nebulised salbutamol/ipratropium bromide 120 µg *bd* and inhaled budesonide 200 µg 2 puffs *bd* since 1997. In April 2000 this was supplemented with nebulised salbutamol 2.5 mg *bd*. In August 2001 she started on nebulised gentamicin 80 mg *bd* as prophylaxis for *Pseudomonas aeruginosa*.

This patient had previously reported increased sputum production, starting in December 2001 and continuing until an unspecified date in 2002. Sputum samples were collected on 03 January 2002 and 15 January 2002 and both were positive for *Pseudomonas aeruginosa*. On 07 April 2002 she reported blood in sputum (Narrative 30). It resolved the same day with no treatment.

On 20 May 2002 this patient reported blood in sputum. It resolved the same day with no treatment. Her pre-infusion body temperature between April and May 2002 ranged from 35.4-36.2°C.

No imaging details, blood test results or clinical symptoms or signs are available to further define the patient's mild "blood in sputum".

BPL comment: As for the previous narrative (number 30) about this patient, hemoptysis is not unexpected and previous isolation of a potential pathogen, which can also be a colonizing bacteria, in the absence of clinical symptoms and signs does not indicate an infection. This should not be regarded as a serious acute bacterial infection.

Narrative number: 32
Patient number: 42
Patient initials: [redacted]
Gender: M
Date of birth: [redacted] 1948
Weight: 78 kg
Centre: Salford Royal Hospital, UK
Diagnosis: CVID
Infection: Loin pain, pyrexia and rigors
Severity: Severe
Serious: Yes (hospitalised)
Dates: 10 June 2004 – 18 June 2004

This 56-year-old male with CVID had suffered recurrent pneumonia in the past but had not been on any regular medication with the exception of SCIG, although he was non-compliant with this treatment. He had recently been in good health with no adverse events since September 2003, when he had been admitted to hospital with a renal calculus and had an intravenous pyelogram.

On 10 June 2004 the patient complained of severe loin pain, pyrexia, rigors and low potassium levels. On 14 June 2004 he was admitted to hospital where he had a kidney, ureter and bladder examination, but no evidence of calculi was found. No bacterial growth was found on examination of a midstream urine sample. He was started on oral cefadroxil 500 mg *bd*, which continued until 24 June 2004. The condition resolved on 18 June 2004 without further treatment.

Due to the patient's non-compliance with diary card completion, no further details of days off work or pre-infusion body temperatures are available.

No imaging details, blood test results or clinical symptoms or signs are available to further define the patient's severe "loin pain, pyrexia and rigors".

BPL comment: The triad of loin pain, pyrexia and rigors suggest pyelonephritis, and possibly a relapse in a patient with a history of renal calculus. However, MSU was negative, therefore there is no reason to suspect this patient had a serious acute bacterial infection.

Narrative number: 33
Patient number: 08
Patient initials: [redacted]
Gender: M
Date of birth: [redacted] 1960
Weight: 79.0 kg
Centre: St Bartholomew's Hospital, London, UK
Diagnosis: CVID
Infection: Pyrexia
Severity: Moderate
Serious: No
Dates: 10 August 2002

This is the same patient as in Narratives 34, 35, 36, 37, 38, 39 and 40.

This 41-year-old male with CVID was suffering from bronchiectasis, recurrent chest infections, rhinitis and joint pains in his wrists, ankles and legs.

At the time of the event, the patient had been taking oral amitriptyline 50 mg *od nocte* since 01 February 2002 for leg and joint pains, and the following oral medication for mycoplasma arthritis: clarithromycin 500 mg *bd* since 14 June 2002; tetracycline 250 mg *qds* since 20 July 2002; ciprofloxacin 500 mg *bd* from 14 June 2002 to 27 July 2002 and 27 July 2002 to 23 August 2002; and diclofenac 400 mg *bd* from 5 August 2002 until 30 August 2002.

Also around the time of this event, the patient was suffering from mild indigestion (from 05 to 11 August 2002), severe sweating (from 06 to 17 August 2002), moderate shakiness of the hands (from 07 to 16 August 2002), a croaky voice (from 07 to 10 August 2002), and severe nausea (on 11 August 2002), all of which resolved without treatment. He was also suffering from severe urticaria (between 29 July 2002 and 29 September 2002), which was being treated with calamine lotion, oral loratidine 10 mg *od* and oral chlorphenamine maleate 4 mg *bd*.

The patient's regular pre-infusion body temperature was stable at around 36.6°C. However, on 10 August 2002 it had increased to 38.0°C, falling to 36.6°C and 36.7°C measured subsequently on the same day. A measurement taken five days later was back to 36.6°C, and remained stable over the subsequent months. From this date, the patient started suffering moderate intermittent headaches that were treated with paracetamol.

No imaging details, blood test results or clinical symptoms or signs are available to further define the patient's moderate "pyrexia".

BPL comment: This patient with chronic respiratory pathology had a spike of temperature (to 38°C) during/around a period of severe sweating (11 days) and "croaky voice" (3 days). The change in voice and some mild indigestion and severe nausea (1 day) are the only possible presenting symptoms to account for the pyrexia. None of this information suggests that the patient was suffering from a serious acute bacterial infection.

Narrative number: 34
Patient number: 08
Patient initials: [redacted]
Gender: M
Date of birth: [redacted] 1960
Weight: 79.0 kg
Centre: St Bartholomew's Hospital, London, UK
Diagnosis: CVID
Infection: Hip pain
Severity: Severe
Serious: No
Dates: 02 September 2002

This is the same patient as in Narratives 33, 35, 36, 37, 38, 39 and 40.

This 41-year-old male with CVID was suffering from bronchiectasis, recurrent chest infections, rhinitis and joint pains in his wrists, ankles and legs.

At the time of the event, the patient had been taking oral amitriptyline 50 mg *od nocte* since 01 February 2002 for leg and joint pains, and the following oral medication for mycoplasma arthritis: clarithromycin 500 mg *bd* since 14 June 2002 and tetracycline 250 mg *qds* since 20 July 2002.

Also around the time of this event, the patient was suffering from severe urticaria (between 29 July and 29 September 2002), which was being treated with calamine lotion, oral loratidine 10 mg *od* and oral chlorphenamine maleate 4 mg *bd*.

The patient complained of severe hip pain on 02 September 2002, which resolved on the same day without further treatment. Less than a week later he suffered a further case of severe hip pain (see Narrative 37). His pre-infusion body temperature was stable at around 36.5°C throughout both episodes of hip pain.

No imaging details, blood test results or clinical symptoms or signs are available to further define the patient's severe "hip pain".

BPL comment: The clinical history and symptoms of this patient do not suggest the hip pain was caused by septic arthritis. Therefore, this patient should not be regarded as a serious acute bacterial infection. This patient had mycoplasma arthritis since before enrolment into the study.

Narrative number: 35
Patient number: 08
Patient initials: [redacted]
Gender: M
Date of birth: [redacted] 1960
Weight: 79.0 kg
Centre: St Bartholomew's Hospital, London, UK
Diagnosis: CVID
Infection: Right knee pain
Severity: Severe
Serious: No
Dates: 21 January 2003 – 24 January 2003

This is the same patient as in Narratives 33, 34, 36, 37, 38, 39 and 40.

This 42-year-old male with CVID was suffering from bronchiectasis, recurrent chest infections, rhinitis and joint pains in his wrists, ankles and legs.

At the time of the event, the patient was on the following oral medications for joint pain: dihydrocodeine tartrate 30 mg *od* (16 December 2002 to 13 February 2003), naproxen 500 mg *bd* (10 January 2003 to 13 Februrary 2003); and prednisolone 30 mg *od* (13 February 2003 to 27 February 2003) He was suffering a concomitant moderate cough and moderate kidney infection.

The patient reported a severe pain in the right knee on 21 January 2003, which resolved on 24 January 2003 without further treatment. However he developed a severe right ankle pain (see Narrative 36) just two days later on 26 January 2003. His body temperature was stable at around 36.5°C throughout both episodes.

No imaging details, blood test results or clinical symptoms or signs are available to further define the patient's severe "right knee pain".

BPL comment: The clinical history and symptoms of this patient do not suggest the knee pain was caused by septic arthritis. Therefore, this event should not be regarded as a serious acute bacterial infection. The patient had mycoplasma arthritis before enrolment into the study.

Narrative number: 36
Patient number: 08
Patient initials: [redacted]
Gender: M
Date of birth: [redacted] 1960
Weight: 79.0 kg
Centre: St Bartholomew's Hospital, London, UK
Diagnosis: CVID
Infection: Right ankle pain
Severity: Severe
Serious: No
Dates: 26 January 2003 – 28 January 2003

This is the same patient as in Narratives 33, 34, 35, 37, 38, 39 and 40.

This 42-year-old male with CVID was suffering from bronchiectasis, recurrent chest infections, rhinitis and joint pains in his wrists, ankles and legs.

At the time of the event, the patient was on the following oral medications for joint pain: dihydrocodeine 30 mg *od*, naproxen 500 mg *bd* and prednisolone 30 mg *od*. He was suffering a concomitant moderate cough and moderate kidney infection.

The patient had recently recovered (24 January 2003) from a severe pain in the right knee (see Narrative 35). However he went on to develop a severe right ankle pain just two days later on 26 January. The pain resolved on 28 January 2003, without further treatment. His body temperature was stable at around 36.5°C throughout both episodes.

No imaging details, blood test results or clinical symptoms or signs are available to further define the patient's severe "right ankle pain".

BPL comment: The clinical history and symptoms of this patient do not suggest the ankle pain was caused by septic arthritis. Therefore, this event should not be regarded as a serious acute bacterial infection. The patient had mycoplasma arthritis before enrolment into the study.

Narrative number: 37
Patient number: 08
Patient initials: [redacted]
Gender: M
Date of birth: [redacted] 1960
Weight: 79.0 kg
Centre: St Bartholomew's Hospital, London, UK
Diagnosis: CVID
Infection: Hip pain
Severity: Severe
Serious: No
Dates: 08 September 2002 – 11 September 2002

This is the same patient as in Narratives 33, 34, 35, 36, 38, 39 and 40.

This 41-year-old male with CVID was suffering from bronchiectasis, recurrent chest infections, rhinitis and joint pains in his wrists, ankles and legs.

At the time of the event, the patient had been taking oral amitriptyline 50 mg *od nocte* since 01 February 2002 for leg and joint pains, and the following oral medication for mycoplasma arthritis: clarithromycin 500 mg *bd* since 14 June 2002 and tetracycline 250 mg *qds* since 20 July 2002, which were both stopped on 13 September 2002.

Also around the time of this event, the patient was suffering from severe urticaria (between 26 July and 29 September 2002), which was being treated with calamine lotion, oral loratidine 10 mg *od* and oral chlorphenamine maleate 4 mg *bd*.

The patient complained of severe hip pain on 02 September 2002, which resolved on the same day without further treatment (see Narrative 34). Less than a week later he suffered a further case of severe hip pain. This was treated with dispersible aspirin 300 mg *od* on 08, 09 and 11 September 2002, and oral dihydrocodeine tartrate 60 mg *od* on 10 September 2002. The event resolved on 11 September 2002. His pre-infusion body temperature was stable at around 36.5°C throughout both episodes of hip pain.

No imaging details, blood test results or clinical symptoms or signs are available to further define the patient's severe "hip pain".

BPL comment: The clinical history and symptoms of this patient do not suggest the hip pain was caused by septic arthritis. Therefore, this event should not be regarded as a serious acute bacterial infection. The patient had mycoplasma arthritis before enrolment into the study.

CONFIDENTIAL

Narrative number: 38
Patient number: 08
Patient initials: [redacted]
Gender: M
Date of birth: [redacted] 1960
Weight: 79.0 kg
Centre: St Bartholomew's Hospital, London, UK
Diagnosis: CVID
Infection: Very painful shoulder
Severity: Severe
Serious: No
Dates: 13 August 2004 – 17 August 2004

This is the same patient as in Narratives 33, 34, 35, 36, 37, 39 and 40.

This 43-year-old male with CVID was suffering from bronchiectasis, recurrent chest infections, rhinitis and joint pains in his wrists, ankles and legs. He had been suffering from moderate depression since July 2003.

At the time of the event, the patient had been taking oral amitriptyline 100 mg *od* for depression and joint pains, supplemented with the use of a "TENS" machine as required, both from July 2003. He was also taking oral prochlorperazine 5 mg *tds* for nausea and vomiting and venlafaxine 37.5 mg *bd* for depression (since 16 June 2004).

The patient complained of a very painful shoulder on 13 August 2004, which resolved on 17 August 2004 without treatment. His body temperature around this time was stable at around 36.2 °C.

No imaging details, blood test results or clinical symptoms or signs are available to further define the patient's severe "very painful shoulder".

BPL comment: The clinical history and symptoms of this patient do not suggest the shoulder pain was caused by septic arthritis. Therefore, this event should not be regarded as a serious acute bacterial infection. The patient had mycoplasma arthritis before enrolment into the study.

Narrative number: 39
Patient number: 08
Patient initials: [redacted]
Gender: M
Date of birth: [redacted] 1960
Weight: 79.0 kg
Centre: St Bartholomew's Hospital, London, UK
Diagnosis: CVID
Infection: Joint pains
Severity: Severe
Serious: No
Dates: 04 May 2001 – 16 May 2001

This is the same patient as in Narratives 33, 34, 35, 36, 37, 38 and 39.

This 40-year-old male with CVID was suffering from bronchiectasis, recurrent chest infections, rhinitis and joint pains in his wrists, ankles and legs.

On 04 May 2001, shortly after joining the study, the patient complained of severe joint pains. The patient visited the hospital on 11 May 2001 as an outpatient, at which point he was put on oral indomethacin 50 mg *tds* and oral omeprazole 20 mg *od*. On 16 May 2001 the joint pains resolved, and the above medication was stopped on 17 May 2001. The patient was also taking oral zopiclone 7.5 mg *nocte prn* for insomnia.

No imaging details, blood test results or clinical symptoms or signs are available to further define the patient's severe "joint pains".

BPL comment: The clinical history and symptoms of this patient do not suggest the joint pains were caused by septic arthritis. Therefore, this event should not be regarded as a serious acute bacterial infection. The patient had mycoplasma arthritis before enrolment into the study.

Narrative number: 40
Patient number: 08
Patient initials: [redacted]
Gender: M
Date of birth: [redacted] 1960
Weight: 79.0 kg
Centre: St Bartholomew's Hospital, London, UK
Diagnosis: CVID
Infection: Severe pain in leg joints
Severity: Severe
Serious: Yes (hospitalised)
Dates: 08 September 2001 – ongoing at 24 September 2004

This is the same patient as in Narratives 33, 34, 35, 36, 37, 38 and 39.

This 40-year-old male with CVID was suffering from bronchiectasis, recurrent chest infections, rhinitis and joint pains in his wrists, ankles and legs. He was on no long-term medication with the exception of the study SCIG and nasal beclometasone dipropionate 1 puff *bd* for rhinitis.

On 09 September 2001 this patient reported severe pain in his leg joints. He was already taking oral gabapentin 300 mg *qds* for leg and joint pain, but the pains worsened and on 17 September he was admitted to hospital for investigations. A lumbar puncture specimen was negative for enterovirus and an arthroscopy of the right knee was positive for mycoplasma. He was put on the following oral medications for a suspected joint infection: ciprofloxacin 500 mg *bd* until 09 October 2001 and tetracycline 500 mg *qds* until 19 July 2002, supplemented with clarythromycin 500 mg *bd* from 04 October 2001 to 01 May 2002. He was also taking paracetamol 1 g *qds* from 21 September to 26 October 2001, oral amitriptyline 25 mg *od nocte* from 29 September 2001 to 31 January 2002 and tramadol 100 mg *qds* from 29 September 2001 to 01 February 2002 for the resultant joint pain.

The severe joint pain was continuing when the patient completed the study on 24 September 2004.

No imaging details, blood test results or clinical symptoms or signs are available to further define the patient's "severe pain in leg joints".

BPL comment: This patient had confirmed mycoplasma infection, at least of the right knee joint. It is possible that other joints were similarly infected. There is no evidence to associate the symptoms with a septic arthritis. Therefore, this should not be regarded as a serious acute bacterial infection.

Narrative number: 41
Patient number: 35
Patient initials: [redacted]
Gender: F
Date of birth: [redacted] 1944
Weight: 105.2 kg
Centre: St Helier Hospital, UK
Diagnosis: CVID
Infection: Diarrhoea/GI infection
Severity: Mild
Serious: No
Dates: 19 August 2002 – 07 September 2002

This 57-year-old female with CVID was suffering from recurrent bronchitis, asthma, bronchiectasis and pneumonia, hiatus hernia, eczema, chronic rhinitis, osteoarthritis, carpal tunnel syndrome, depression, insomnia and hypertension. She had been on inhaled salbutamol 2 puffs *prn* and oral aminophylline 450 mg *bd* for asthma, both since the 1970s. In 1998 this was supplemented with inhaled budesonide 1 puff *prn* and in 2000 with oral montelukast 10 mg *od*. She was also taking oral rofecoxib 12.5 mg for osteoarthritis (since 1998), although this was stopped in February 2001, oral isosorbine mononitrate 10 mg *tds* for angina (since 1992), oral lisinopril 5 mg *od* for hypertension (since 1990), oral cetirizine 10 mg *prn* for itching (since 1998), oral temazepam 20 mg *prn nocte* for insomnia (since 1995), oral oestrogen 625 µg as HRT (since September 2001) and oral desloratidine 5 mg *od* for hayfever (since May 2002).

On 19 August 2002 the patient reported a mild case of viral infection affecting the GI tract, which she treated with the following over-the-counter products: oral Diocalm (morphine/attapulgite) 10 mL *prn* between 21 to 26 August 2002 and oral Diocalm Replenish (morphine/attapulgite) 1 sachet *prn* between 28 to 30 August 2002. She had a body temperature of 37.1°C both pre- and post-infusion on 28 August; 37.2°C pre-infusion on 29 August, falling to 36.7°C post-infusion; and 37.1°C pre-infusion on 03 September, falling to 36.6°C post-infusion. The condition resolved without further treatment on 07 September 2002.

No imaging details, blood test results or clinical symptoms or signs are available to further define the patient's mild "diarrhoea/GI infection".

BPL comment: This patient had a prolonged period (19 days) of gastrointestinal symptoms with occasional low grade pyrexia. There is no clinical reason to consider she had a serious acute bacterial infection.

Narrative number: 42
Patient number: 58
Patient initials: [redacted]
Gender: Male
Date of birth: [redacted] 1971
Weight: 83.3 kg
Centre: University Hospital of Wales, Cardiff, UK
Diagnosis: Specific Antibody Deficiency
Infection: Perianal abscess
Severity: Severe
Serious: Yes (hospitalised)
Dates: 18 August 2002 – 28 August 2002

This is the same patient as in Narratives 13, 14, 15, 16 and 17.

This 31-year-old male with specific antibody deficiency had had bronchiectasis for many years prior to the study. He also suffered from recurrent skin abscesses and severe eczema as part of the Hyper-IgE (Job's) syndrome. He had been on oral flucloxacillin 500 mg *qds* for chest prophylaxis, fluconazole 200 mg *od*, and co-codamol 30/500 *qds prn* for some years. Inhaled treatment consisted of salmeterol 2 puffs *bd*, fluticasone propionate 2 puffs *bd*, both for many years, and salbutamol 2 puffs *prn* since June 2001.

In September 2001 his dose of oral fluticasone propionate was increased to 4 puffs *bd*. In January 2002 the dose of oral fluticasone propionate was changed to 250 µg *bd* and he was also started on oral flucloxacillin 500 mg *qds* for prophylaxis against chest infections. In April 2002 this was supplemented by nebulised colomycin 2 million units *bd*. He was also on oral loratidine 10 mg *od* for hayfever.

On 18 August 2002 the patient developed a severe perianal abscess despite long-term prophylaxis with flucoxacillin. On 20 August he was admitted to hospital and underwent surgery under general anaesthetic. He was discharged from hospital on 21 August 2002. He took oral metronidazole 400 mg *tds* and oral diclofenac 50 mg *tds* from 21 to 26 August 2002. The wound required daily dressings but was considered to have healed by 28 August 2002. His body temperature ranged from 36.3 to 36.6°C during the period of the event.

No imaging details, blood test results or clinical symptoms or signs are available to further define the patient's severe "perianal abscess".

BPL comment: This patient had a propensity to pyogenic skin lesions as a complication of his primary disease. However, the perianal abscess should be regarded as a serious acute bacterial infection.

CONFIDENTIAL

Narrative number: 43
Patient number: 18
Patient initials: [redacted]
Gender: F
Date of birth: [redacted] 1978
Weight: 111.6 kg
Centre: Northern General Hospital, Sheffield, UK
Diagnosis: CVID
Infection: Abscess
Severity: Moderate
Serious: No
Dates: 24 December 2003 – date unknown

This is the same patient as in Narrative 44.

This 25-year-old female with CVID had a pneumothorax in 1996. High-resolution computed tomography indicated some fine reticulonodular changes but these were thought to be due to obesity. In February 2001 at entry to the study her chest was normal on examination and she had no other current or past medical conditions. She was, however, suffering from frequent coughs, colds and rhinorrhoea.

On 24 December 2003 the patient reported swollen gums around her wisdom tooth and a gum abscess of moderate intensity. She started on oral erythromycin 250 mg (frequency unrecorded) on 24 December 2003 for an unknown length of time. She took no days off work due to the event and her recorded pre-infusion body temperatures in the two weeks after the event were 36.1 and 36.0°C. The condition resolved after an unrecorded length of time.

No imaging details, blood test results or clinical symptoms or signs are available to further define the patient's moderate "abscess".

BPL comment: This patient developed a dental abscess. The general state of her dental hygiene is unknown. Nevertheless, this should be regarded as a serious acute bacterial infection.

CONFIDENTIAL

Narrative number: **44**
Patient number: **18**
Patient initials: [redacted]
Gender: **F**
Date of birth: [redacted] **1978**
Weight: **111.6 kg**
Centre: **Northern General Hospital, Sheffield, UK**
Diagnosis: **CVID**
Infection: **Pneumonia (cough)**
Severity: **Moderate**
Serious: **No**
Dates: **13 February 2003 – 28 February 2003**

This is the same patient as in Narrative 43.

This 25-year-old female with CVID had a pneumothorax in 1996. High-resolution computed tomography indicated some fine reticulonodular changes but these were thought to be due to obesity. In February 2001 at entry to the study her chest was normal on examination and she had no other current or past medical conditions. She was, however, suffering from frequent coughs, colds and rhinorrhoea.

On 24 January 2003 the patient reported a cough, cold and rhinorrhoea, all of moderate intensity. These symptoms all resolved at an unrecorded date, without treatment.

On 13 February 2003 the patient reported a bad cough. She visited her GP on 14 February and was given no medication, but attended the hospital as an out-patient on 18 February 2003. At this visit the cough was diagnosed as pneumonia, and she was started on oral clarithromycin 250 mg *bd*. She completed the ten-day course of medication on 28 February 2003, when the presumed infection had resolved. During this time she took 4 days off work.

No imaging details, blood test results or clinical symptoms or signs are available to further define the patient's moderate "pneumonia (cough)".

BPL comment: This patient had symptoms of respiratory tract infection which was subsequently diagnosed as pneumonia. It is noteworthy, however, that she was not hospitalised, despite having CVID. Thus the diagnosis of pneumonia could be regarded as uncertain. Nevertheless this should be regarded as a serious acute bacterial infection.

14.3 Safety Data

14.3.1 Adverse Events (Excluding Infections)

14.3.2 Deaths and Other Serious and Significant Adverse Events (Excluding Infections)

14.3.3 Infusion Site Reactions

14.3.4 Laboratory Data

14.3.5 Vital Signs

15 References

CPMP Guidelines to assess efficacy and safety of normal intravenous immunoglobulin products for marketing authorisations. CPMP/388/95 Final. London, 13 February 1996.

CPMP Note for guidance on the clinical investigation of human normal immunoglobulin for intravenous administration (IVIG). CPMP/BPWG/388/95, revision 1. London, June 2000.

CPMP Note for guidance on the clinical investigation of human normal immunoglobulin for subcutaneous and intramuscular use. CPMP/BPWG/283/00. London, draft version dated 29 March 2001.

CPMP Note for guidance on the clinical investigation of human normal immunoglobulin for subcutaneous and intramuscular use. EMEA/CPMP/BPWG/283/00. London, 25 July 2002.

Gardulf A, Hammarström L, Smith ECI. Home treatment of hypogammaglobulinaemia with subcutaneous gammaglobulin by rapid infusion. Lancet 1991; 338: 162-166.

Gardulf A, Andersen V, Bjorkander J, Ericson D, *et al.* Subcutaneous immunoglobulin replacement in patients with primary antibody deficiencies: safety and costs. Lancet 1995; 345: 365-9.

Gaspar J, Gerritsen B, Jones A. Immunoglobulin replacement treatment by rapid subcutaneous infusion. Archives of Disease in Childhood 1998; 79: 48-51.

Karch FE, Lasagna L. Adverse drug reactions. A critical review. JAMA 1975; 234: 1236-1241.

Kittner JM, Grimbacher B, Wulff W, Jager B, Schmidt RE. Patients' attitude to subcutaneous immunoglobulin substitution as home therapy. Journal of Clinical Immunology 2006; 26, No.4: 400-405.

Morell A. Chapter 1, Pharmacokinetics of intravenous immunoglobulin preparations. In: Intravenous Immunoglobulins in Clinical Practice. Lee ML & Strand V, Marcel Dekker, Inc, 1997, p1-18.

Stein MR, Ochs HD, C, Investigators. Percentage of patients experiencing local infusion-site reactions during subcutaneous immunoglobulin therapy decreases rapidly upon repeated infusions. The Journal of Allergy and Clinical Immunology 2006, Vol 117, Suppl 2, s108.

US Food and Drug Administration, Guidance for Industry (Draft). Safety, efficacy, and pharmacokinetic studies to support marketing of immune globulin intravenous (human) as replacement therapy for primary humoral immunodeficiency. Nov 2005.

Waniewski J, Gardulf A, Hammerström L. Bioavailability of γ-globulin after subcutaneous infusions in patients with common variable immunodeficiency. Journal of Clinical Immunology 1994; 14, No. 2.

WHO Scientific Group. Primary immunodeficiency diseases. Clinical and Experimental Immunology 1997; 109 Suppl. 1: 1–28.

16.1 Study Information

16.1.1 Protocol and Protocol Amendments

16.1.2 Sample Case Report Form and Other Forms Used for Data Collection

16.1.3 Ethics

- 16.1.3.1 List of MREC and LRECS.
- 16.1.3.2 MREC Approved Patient Information Leaflets
- 16.1.3.3 MREC Approved Patient Informed Consent Forms
- 16.1.3.4 LREC Modified Patient Information and Consent Forms

16.1.4 List and Description of Investigators

- 16.1.4.1 Curriculum Vitae of Investigators
- 16.1.4.2 Curriculum Vitae of Other Key Staff
- 16.1.4.3 Curriculum Vitae of BPL Staff

16.1.5 Responsible Persons at BPL Signature Page

16.1.6 List of Batch Numbers Received by All Patients

16.1.7 Randomisation Scheme and Codes

16.1.8 Audit Certificates

16.1.9 Documentation of Statistical Methods (including Statistical Analysis Plan and Filenotes 001, 002, 003, 004 and 19 October 2006)

16.1.10 Documentation of Inter-Laboratory Standardization Methods and Quality Assurance Procedures

16.1.11 Publications Based on the Study

16.1.12 Important Publications Referenced in the Report

16.2 Patient Data Listings

16.2.1 Patients Who Discontinued Therapy

16.2.2 Protocol Deviations

16.2.3 Listings for Patients Excluded from Efficacy and Safety Analysis

16.2.4 Demographic Data

16.2.5 Compliance and Drug Concentration Data

16.2.6 Individual Efficacy Response Data

16.2.7 Adverse Event Listings

16.2.8 Laboratory Measurements

16.2.9 Other Data Listings

16.3 Case Report Forms

16.3.1 CRFs of Deaths, Withdrawals Due to SAEs

16.3.2 Other CRFs
