# Supplementary material for: Experience with Subgam, a Subcutaneously Administered Human Normal Immunoglobulin (ClinicalTrials.gov - NCT02247141)
Source: PLoS One. 2015 Jul 29;10(7):e0131565. doi: 10.1371/journal.pone.0131565 (PMC4519338; doi:10.1371/journal.pone.0131565)
Supplement: S2 File — This is part 2 of 2 of SCIG01 clinical study report. A multi-centre open study to assess the safety and efficacy of Subgam given via the subcutaneous route in primary antibody deficient patients (study code SCIG01) (ZIP) [file pone.0131565.s002.zip › SCIG01 -clinical study report - S2 part 2 of 2.pdf]

Table / Figure Number	Table / Figure Name
**14.1**	**Demographic Data**
Table 14.1.1	Patient disposition (all patients)
Table 14.1.2	Number of patients enrolled at each hospital
Table 14.1.3	Identification of patients enrolled at each hospital, by diagnosis
Table 14.1.4	Mean (range) demographic data by diagnosis of PAD, age group and prior therapy
Table 14.1.5	Mean (range) demographic data by specific diagnosis of PAD
Table 14.1.6	Mean (range) demographic data by prior therapy
Table 14.1.7	Diagnosis of PAD by gender, age group and prior therapy
Table 14.1.8	Time from diagnosis of PAD by diagnosis, gender, age group and prior therapy
Table 14.1.9	Time from onset of PAD by diagnosis, gender, age group and prior therapy
Table 14.1.10	Summary of IgG therapy immediately prior to starting Subgam – by diagnosis of PAD and age group
Table 14.1.11	Blood group serology
Table 14.1.12	Coombs' test results by gender, diagnosis of PAD, age group and prior therapy
Table 14.1.13	Number of patients with previous and present medical conditions
Table 14.1.14	Mean (range) baseline serum IgG levels (g/L) by age group and prior therapy (Infusions 1-4)
Table 14.1.15	Mean (range) baseline serum IgG levels (g/L) by diagnosis of PAD and prior therapy (Infusions 1-4)

Table 14.1.1: Patient disposition (all patients)

n	Adults (>=20y)		Teenagers (>=12y-<20y)		Children (<12y)	
Prior therapy	SCIG	IVIG	SCIG	IVIG	SCIG	IVIG
Withdrawn from study	3	7	0	1	2	2
Completed the study	3	15	1	5	5	6
Total	6	22	1	6	7	8

Program: T0002.SAS, Version: 8.2, Datetime: 07FEB07:09:48

Table 14.1.2: Number of patients enrolled at each hospital

Hospital	Number of Patients	Patient Number(s)
Great Ormond Street	6	81, 82, 83, 84, 85, 86
Hope Hospital	3	41, 42, 43
John Radcliffe Hospital	4	53, 54, 55, 56
Leicester Royal Infirmary	4	73, 74, 75, 76
Northern General Hospital	2	17, 18
Papworth Hospital	6	21, 22, 23, 24, 77, 78
Birmingham Children's Hospital	5	25, 26, 27, 28, 65
Royal Preston Hospital	1	61
St Barts Hospital	4	05, 06, 07, 08
Southampton General Hospital	1	13
St Helier Hospital	4	33, 34, 35, 49
St James University Hospital	1	01
The Guest Hospital	3	29, 30, 31
University Hospital Wales	6	09, 10, 11, 12, 57, 58

Program: T0003.SAS, Version: 8.2, Datetime: 07FEB07:09:48

Table 14.1.3: Identification of patients enrolled at each hospital, by diagnosis

Hospital	CVID	XLA	IgG subclass deficiency	Specific antibody deficiency	C40 ligand deficiency	IgG heavy chain deficiency	Ataxia telangiectasia	Combined immuno-deficiency	All patients
Birmingham Children's Hospital	25,65	26,28	27						25,26,27,28,65
Great Ormond Street	81,84,86				82,83			85	81,82,83,84,85,86
Hope Hospital	41,42			43					41,42,43
John Radcliffe Hospital	54,56		53	55					53,54,55,56
Leicester Royal Infirmary	73,74,76			75					73,74,75,76
Northern General Hospital	18					17			17,18
Papworth Hospital	21,22,23,24,77			78					21,22,23,24,77,78
Royal Preston Hospital				61					61
Southampton General Hospital				13					13
St Barts Hospital	06,08		05,07						05,06,07,08
St Helier Hospital	33,34,35,49								33,34,35,49
St James University Hospital							01		01
The Guest Hospital	29,30,31								29,30,31
University Hospital Wales	10,11,12	57	09	58					09,10,11,12,57,58

Program: T0004.SAS, Version: 8.2, Datetime: 07FEB07:09:48

Table 14.1.4: Mean (range) demographic data by diagnosis of PAD, age group and prior therapy

Patient group	n	Age (years)	Height (cm)	Weight (kg)	BMI
All patients	50	29.5 (0.8 - 75.2)	148.6 (72 - 186)	57.6 (10.1 - 132.7)	23.9 (13.2 - 43.6)
Male	25	22.8 (0.8 - 75.2)	140.9 (72 - 186)	48.2 (10.1 - 90.0)	21.3 (13.3 - 41.5)
Female	25	36.2 (5.7 - 64.7)	156.2 (107 - 180)	67.0 (16.0 - 132.7)	26.6 (13.2 - 43.6)

Table 14.1.4: Mean (range) demographic data by diagnosis of PAD, age group and prior therapy

By Diagnosis of PAD	Patient group	n	Age (years)	Height (cm)	Weight (kg)	BMI
CVID/XLA	All patients	33	31.2 (3.0 - 62.0)	154.7 (88 - 186)	64.3 (12.7 - 132.7)	25.2 (15.5 - 43.6)
	Male	15	25.4 (3.0 - 53.9)	149.3 (88 - 186)	53.1 (12.7 - 79.2)	21.3 (15.5 - 27.3)
	Female	18	35.9 (8.2 - 62.0)	159.1 (133 - 180)	73.6 (29.3 - 132.7)	28.5 (16.5 - 43.6)
Other	All patients	17	26.2 (0.8 - 75.2)	136.8 (72 - 180)	44.6 (10.1 - 90.0)	21.4 (13.2 - 41.5)
	Male	10	18.8 (0.8 - 75.2)	128.3 (72 - 180)	40.8 (10.1 - 90.0)	21.3 (13.3 - 41.5)
	Female	7	36.7 (5.7 - 64.7)	148.9 (107 - 167)	50.0 (16.0 - 72.2)	21.6 (13.2 - 26.6)

Table 14.1.4: Mean (range) demographic data by diagnosis of PAD, age group and prior therapy

By Age Group	Patient group	n	Age (years)	Height (cm)	Weight (kg)	BMI
Adult	All patients	28	45.5 (21.3 - 75.2)	163.9 (131 - 186)	77.1 (46.0 - 132.7)	28.6 (20.4 - 43.6)
	Male	10	44.9 (30.0 - 75.2)	169.4 (131 - 186)	77.3 (65.7 - 90.0)	27.0 (21.9 - 41.5)
	Female	18	45.9 (21.3 - 64.7)	160.9 (152 - 180)	76.9 (46.0 - 132.7)	29.5 (20.4 - 43.6)
Teenager	All patients	7	15.2 (12.1 - 18.0)	161.4 (153 - 173)	53.7 (37.2 - 70.1)	20.5 (16.5 - 27.0)
	Male	3	16.7 (15.9 - 17.7)	167.7 (164 - 173)	56.9 (49.0 - 70.1)	20.4 (17.8 - 24.3)
	Female	4	14.1 (12.1 - 18.0)	156.8 (153 - 159)	51.3 (37.2 - 69.0)	20.5 (16.5 - 27.0)
Child	All patients	15	6.2 (0.8 - 10.6)	113.9 (72 - 148)	23.1 (10.1 - 48.2)	16.9 (13.2 - 21.4)
	Male	12	5.8 (0.8 - 10.6)	110.5 (72 - 148)	21.8 (10.1 - 48.2)	16.9 (13.3 - 21.4)
	Female	3	7.5 (5.7 - 8.5)	127.7 (107 - 143)	28.4 (16.0 - 39.9)	17.0 (13.2 - 20.4)

Table 14.1.4: Mean (range) demographic data by diagnosis of PAD, age group and prior therapy

By Prior Therapy	Patient group	n	Age (years)	Height (cm)	Weight (kg)	BMI
SCIG	All patients	14	25.7 (2.7 - 64.7)	136.7 (94 - 168)	46.4 (12.8 - 89.1)	21.6 (13.2 - 34.8)
	Male	8	11.7 (2.7 - 40.4)	127.1 (94 - 168)	33.0 (12.8 - 79.0)	18.1 (13.3 - 27.3)
	Female	6	44.5 (5.7 - 64.7)	149.5 (107 - 167)	64.3 (16.0 - 89.1)	26.3 (13.2 - 34.8)
IVIG	All patients	36	30.9 (0.8 - 75.2)	153.2 (72 - 186)	61.9 (10.1 - 132.7)	24.9 (14.2 - 43.6)
	Male	17	28.0 (0.8 - 75.2)	147.4 (72 - 186)	55.4 (10.1 - 90.0)	22.9 (14.2 - 41.5)
	Female	19	33.5 (8.2 - 62.7)	158.4 (133 - 180)	67.8 (29.3 - 132.7)	26.6 (16.5 - 43.6)

Table 14.1.5: Mean (range) demographic data by specific diagnosis of PAD

Diagnosis	n	Age (years)	Height (cm)	Weight (kg)	BMI
All patients	50	29.5 (0.8 - 75.2)	148.6 (72 - 186)	57.6 (10.1 - 132.7)	23.9 (13.2 - 43.6)
CVID/XLA	33	31.2 (3.0 - 62.0)	154.7 (88 - 186)	64.3 (12.7 - 132.7)	25.2 (15.5 - 43.6)
Other	17	26.2 (0.8 - 75.2)	136.8 (72 - 180)	44.6 (10.1 - 90.0)	21.4 (13.2 - 41.5)
CVID	30	33.7 (3.3 - 62.0)	159.0 (88 - 186)	68.8 (12.7 - 132.7)	26.2 (15.7 - 43.6)
XLA	3	6.0 (3.0 - 10.0)	111.0 (97 - 122)	19.5 (15.5 - 23.4)	16.0 (15.5 - 16.3)
IgG subclass deficiency	5	33.0 (2.7 - 64.7)	138.8 (94 - 180)	46.5 (12.8 - 90.0)	20.9 (13.3 - 27.8)
Specific antibody deficiency	7	26.9 (8.5 - 62.7)	158.0 (139 - 176)	55.8 (35.0 - 83.3)	21.7 (17.8 - 25.7)
Ataxia Telangiectasia	1	5.7 (5.7 - 5.7)	107.0 (107 - 107)	16.0 (16.0 - 16.0)	13.2 (13.2 - 13.2)
CD40 Ligand Deficiency	2	2.0 (0.8 - 3.1)	86.0 (72 - 100)	14.5 (10.1 - 18.8)	19.7 (18.8 - 20.6)
IgG Heavy Chain Deficiency	1	75.2 (75.2 - 75.2)	131.0 (131 - 131)	70.1 (70.1 - 70.1)	41.5 (41.5 - 41.5)
Combined Immunodeficiency	1	6.6 (6.6 - 6.6)	115.0 (115 - 115)	20.5 (20.5 - 20.5)	14.2 (14.2 - 14.2)

Table 14.1.6: Mean (range) demographic data by prior therapy

Prior Therapy	n	Age (years)	Height (cm)	Weight (kg)	BMI
All patients	50	29.5 (0.8 - 75.2)	148.6 (72 - 186)	57.6 (10.1 - 132.7)	23.9 (13.2 - 43.6)
SCIG - All patients	15	26.6 (2.7 - 64.7)	138.7 (94 - 168)	48.1 (12.8 - 89.1)	21.9 (13.2 - 34.8)
SCIG - Home	10	25.0 (5.0 - 59.2)	140.2 (107 - 167)	48.3 (16.0 - 89.1)	21.8 (13.2 - 34.8)
SCIG - Hospital	5	30.0 (2.7 - 64.7)	135.8 (94 - 168)	47.9 (12.8 - 79.0)	22.0 (15.5 - 27.3)
IVIG - All patients	36	30.9 (0.8 - 75.2)	153.2 (72 - 186)	61.9 (10.1 - 132.7)	24.9 (14.2 - 43.6)
IVIG - Home	7	37.1 (15.9 - 56.0)	163.9 (157 - 173)	72.0 (51.5 - 105.2)	26.4 (17.8 - 41.1)
IVIG - Hospital	29	29.4 (0.8 - 75.2)	150.6 (72 - 186)	59.5 (10.1 - 132.7)	24.5 (14.2 - 43.6)

Table 14.1.7: Diagnosis of PAD by gender, age group and prior therapy

Diagnosis	n	Adults (>=20y)				Teenagers (>=12y-<20y)				Children (<12y)			
		Male	Female	SCIG	IVIG	Male	Female	SCIG	IVIG	Male	Female	SCIG	IVIG
All patients	50	10	18	22	6	3	4	6	1	12	3	8	7
CVID/XLA	33	7	13	16	4	2	4	5	1	6	1	3	4
Other	17	3	5	6	2	1	0	1	0	6	2	5	3
CVID	30	7	13	16	4	2	4	5	1	3	1	3	1
XLA	3	0	0	0	0	0	0	0	0	3	0	0	3
IgG subclass deficiency	5	1	2	2	1	0	0	0	0	2	0	0	2
Specific antibody deficiency	7	1	3	3	1	1	0	1	0	1	1	2	0
Ataxia Telangiectasia	1	0	0	0	0	0	0	0	0	0	1	0	1
CD40 Ligand Deficiency	2	0	0	0	0	0	0	0	0	2	0	2	0
IgG Heavy Chain Deficiency	1	1	0	1	0	0	0	0	0	0	0	0	0
Combined Immunodeficiency	1	0	0	0	0	0	0	0	0	1	0	1	0

Table 14.1.8: Time from diagnosis of PAD by diagnosis, gender, age group and prior therapy

		Time from diagnosis to study start (years)							
Patient group	n	>0.5, <=1.0	>1.0, <=2.0	>2.0, <=3.0	>3.0, <=4.0	>4.0, <=5.0	>5.0, <=10.0	>10.0	Not Known
All patients	50	5	12	4	5	8	9	7	0
Male	25	3	3	3	1	3	7	5	0
Female	25	2	9	1	4	5	2	2	0

Table 14.1.8: Time from diagnosis of PAD by diagnosis, gender, age group and prior therapy

			Time from diagnosis to study start (years)							
By Diagnosis of PAD	Patient group	n	>0.5, <=1.0	>1.0, <=2.0	>2.0, <=3.0	>3.0, <=4.0	>4.0, <=5.0	>5.0, <=10.0	>10.0	Not Known
CVID/XLA	All patients	33	3	8	3	2	4	7	6	0
	Male	15	1	1	2	0	1	6	4	0
	Female	18	2	7	1	2	3	1	2	0
Other	All patients	17	2	4	1	3	4	2	1	0
	Male	10	2	2	1	1	2	1	1	0
	Female	7	0	2	0	2	2	1	0	0

Table 14.1.8: Time from diagnosis of PAD by diagnosis, gender, age group and prior therapy

By Age Group	Patient group	n	Time from diagnosis to study start (years) >0.5, <=1.0	>1.0, <=2.0	>2.0, <=3.0	>3.0, <=4.0	>4.0, <=5.0	>5.0, <=10.0	>10.0	Not Known
Adult	All patients	28	2	7	2	4	4	4	5	0
	Male	10	0	1	1	1	1	2	4	0
	Female	18	2	6	1	3	3	2	1	0
Teenager	All patients	7	0	3	0	0	0	2	2	0
	Male	3	0	0	0	0	0	2	1	0
	Female	4	0	3	0	0	0	0	1	0
Child	All patients	15	3	2	2	1	4	3	0	0
	Male	12	3	2	2	0	2	3	0	0
	Female	3	0	0	0	1	2	0	0	0

Program: T0009.SAS, Version: 8.2, Datetime: 07FEB07:09:48

Table 14.1.8: Time from diagnosis of PAD by diagnosis, gender, age group and prior therapy

By Prior Therapy	Patient group	n	Time from diagnosis to study start (years) >0.5, <=1.0	>1.0, <=2.0	>2.0, <=3.0	>3.0, <=4.0	>4.0, <=5.0	>5.0, <=10.0	>10.0	Not Known
SCIG	All patients	14	2	1	1	1	4	4	1	0
	Male	8	2	0	0	0	2	3	1	0
	Female	6	0	1	1	1	2	1	0	0
IVIG	All patients	36	3	11	3	4	4	5	6	0
	Male	17	1	3	3	1	1	4	4	0
	Female	19	2	8	0	3	3	1	2	0

Program: T0009.SAS, Version: 8.2, Datetime: 07FEB07:09:48

Table 14.1.9: Time from the onset of PAD by diagnosis, gender, age group and prior therapy

		Time from onset to study start (years)							
Patient group	n	>0.5, <=1.0	>1.0, <=2.0	>2.0, <=3.0	>3.0, <=4.0	>4.0, <=5.0	>5.0, <=10.0	>10.0	Not Known
All patients	50	1	0	3	5	4	12	17	8
Male	25	1	0	2	3	2	5	11	1
Female	25	0	0	1	2	2	7	6	7

Program: T0010.SAS, Version: 8.2, Datetime: 07FEB07:09:48

Table 14.1.9: Time from the onset of PAD by diagnosis, gender, age group and prior therapy

By Diagnosis of PAD	Patient group	n	Time from onset to study start (years) >0.5, <=1.0	>1.0, <=2.0	>2.0, <=3.0	>3.0, <=4.0	>4.0, <=5.0	>5.0, <=10.0	>10.0	Not Known
CVID/XLA	All patients	33	0	0	2	4	2	6	12	7
	Male	15	0	0	1	2	1	3	7	1
	Female	18	0	0	1	2	1	3	5	6
Other	All patients	17	1	0	1	1	2	6	5	1
	Male	10	1	0	1	1	1	2	4	0
	Female	7	0	0	0	0	1	4	1	1

Program: T0010.SAS, Version: 8.2, Datetime: 07FEB07:09:48

Table 14.1.9: Time from the onset of PAD by diagnosis, gender, age group and prior therapy

			Time from onset to study start (years)							
By Age Group	Patient group	n	>0.5, <=1.0	>1.0, <=2.0	>2.0, <=3.0	>3.0, <=4.0	>4.0, <=5.0	>5.0, <=10.0	>10.0	Not Known
Adult	All patients	28	0	0	0	2	3	5	12	6
	Male	10	0	0	0	1	1	1	7	0
	Female	18	0	0	0	1	2	4	5	6
Teenager	All patients	7	0	0	1	0	0	1	3	2
	Male	3	0	0	0	0	0	0	2	1
	Female	4	0	0	1	0	0	1	1	1
Child	All patients	15	1	0	2	3	1	6	2	0
	Male	12	1	0	2	2	1	4	2	0
	Female	3	0	0	0	1	0	2	0	0

Program: T0010.SAS, Version: 8.2, Datetime: 07FEB07:09:48

Table 14.1.9: Time from the onset of PAD by diagnosis, gender, age group and prior therapy

By Prior Therapy	Patient group	n	Time from onset to study start (years) >0.5, <=1.0	>1.0, <=2.0	>2.0, <=3.0	>3.0, <=4.0	>4.0, <=5.0	>5.0, <=10.0	>10.0	Not Known
SCIG	All patients	14	0	0	2	0	3	5	3	1
	Male	8	0	0	2	0	1	2	2	1
	Female	6	0	0	0	0	2	3	1	0
IVIG	All patients	36	1	0	1	5	1	7	14	7
	Male	17	1	0	0	3	1	3	9	0
	Female	19	0	0	1	2	0	4	5	7

Program: T0010.SAS, Version: 8.2, Datetime: 07FEB07:09:48

Table 14.1.10: Summary of IgG therapy immediately prior to starting Subgam - by diagnosis of PAD and age group

Patient group	Prior Therapy	n	Patient Number
SCIG	Home	10	01, 06, 25, 26, 27, 28, 73, 74, 75, 76
	Hospital	4	05, 08, 53, 57
	Total	14	01, 05, 06, 08, 25, 26, 27, 28, 53, 57, 73, 74, 75, 76
IVIG	Home	7	07, 12, 34, 35, 41, 55, 56
	Hospital	29	09, 10, 11, 13, 17, 18, 21, 22, 23, 24, 29, 30, 31, 33, 42, 43, 49, 54, 58, 61, 65, 77, 78, 81, 82, 83, 84, 85, 86
	Total	36	07, 09, 10, 11, 12, 13, 17, 18, 21, 22, 23, 24, 29, 30, 31, 33, 34, 35, 41, 42, 43, 49, 54, 55, 56, 58, 61, 65, 77, 78, 81, 82, 83, 84, 85, 86

Table 14.1.10: Summary of IgG therapy immediately prior to starting Subgam - by diagnosis of PAD and age group

By Diagnosis of PAD	Patient group	Prior Therapy	n	Patient Number
CVID/XLA	SCIG	Home	7	06, 25, 26, 28, 73, 74, 76
		Hospital	2	08, 57
		Total	9	06, 08, 25, 26, 28, 57, 73, 74, 76
	IVIG	Home	5	12, 34, 35, 41, 56
		Hospital	19	10, 11, 18, 21, 22, 23, 24, 29, 30, 31, 33, 42, 49, 54, 65, 77, 81, 84, 86
		Total	24	10, 11, 12, 18, 21, 22, 23, 24, 29, 30, 31, 33, 34, 35, 41, 42, 49, 54, 56, 65, 77, 81, 84, 86
Other	SCIG	Home	3	01, 27, 75
		Hospital	2	05, 53
		Total	5	01. 05, 27, 53, 75
	IVIG	Home	2	07, 55
		Hospital	10	09, 13, 17, 43, 58, 61, 78, 82, 83, 85
		Total	12	07, 09, 13, 17, 43, 55, 58, 61, 78, 82, 83, 85

Program: T0011.SAS, Version: 8.2, Datetime: 07FEB07:09:48

Table 14.1.10: Summary of IgG therapy immediately prior to starting Subgam - by diagnosis of PAD and age group

By Age Group	Patient group	Prior Therapy	n	Patient Number
Adults (>=20y)	SCIG	Home	4	73, 74, 75, 76
		Hospital	2	08, 53
		Total	6	08, 53, 73, 74, 75, 76
	IVIG	Home	4	07, 12, 34, 35
		Hospital	18	09, 10, 11, 17, 18, 21, 22, 23, 24, 29, 31, 33, 42, 43, 54, 58, 77, 78
		Total	22	07, 09, 10, 11, 12, 17, 18, 21, 22, 23, 24, 29, 31, 33, 34, 35, 42, 43, 54, 58, 77, 78
Teenagers (>=12y-<20y)	SCIG	Home	1	06
		Total	1	06
	IVIG	Home	3	41, 55, 56
		Hospital	3	30, 49, 84
		Total	6	30, 41, 49, 55, 56, 84
Children (<12y)	SCIG	Home	5	01, 25, 26, 27, 28
		Hospital	2	05, 57
		Total	7	01, 05, 25, 26, 27, 28, 57
	IVIG	Hospital	8	13, 61, 65, 81, 82, 83, 85, 86
		Total	8	13, 61, 65, 81, 82, 83, 85, 86

Table 14.1.11: Blood group serology

ABO Type	Rhesus Type			
	Negative	Positive	Not Known	Total
A	3	19	0	22
AB	1	1	0	2
B	1	4	0	5
O	3	17	0	20
Not Known	0	0	1	1
Total	8	41	1	50

Program: T0012.SAS, Version: 8.2, Datetime: 07FEB07:09:48

Table 14.1.12: Coombs' test results by gender, diagnosis of PAD, age group and prior therapy

		Direct Coombs Test		
Patient group	n	Negative	Positive	Not Done
All patients	50	36	6	8
Male	25	21	1	3
Female	25	15	5	5

Program: T0013.SAS, Version: 8.2, Datetime: 07FEB07:09:48

Table 14.1.12: Coombs' test results by gender, diagnosis of PAD, age group and prior therapy

By Diagnosis of PAD	Patient group	n	Direct Coombs Test		
			Negative	Positive	Not Done
CVID/XLA	All patients	33	21	5	7
	Male	15	11	1	3
	Female	18	10	4	4
Other	All patients	17	15	1	1
	Male	10	10	0	0
	Female	7	5	1	1

Table 14.1.12: Coombs' test results by gender, diagnosis of PAD, age group and prior therapy

By Age Group	Patient group	n	Direct Coombs Test		
			Negative	Positive	Not Done
Adult	All patients	28	17	5	6
	Male	10	8	0	2
	Female	18	9	5	4
Teenager	All patients	7	5	1	1
	Male	3	2	1	0
	Female	4	3	0	1
Child	All patients	15	14	0	1
	Male	12	11	0	1
	Female	3	3	0	0

Table 14.1.12: Coombs' test results by gender, diagnosis of PAD, age group and prior therapy

By Prior Therapy	Patient group	n	Direct Coombs Test		
			Negative	Positive	Not Done
SCIG	All patients	14	9	1	4
	Male	8	7	0	1
	Female	6	2	1	3
IVIG	All patients	36	27	5	4
	Male	17	14	1	2
	Female	19	13	4	2

Program: T0013.SAS, Version: 8.2, Datetime: 07FEB07:09:48

Table 14.1.13: Number of patients with previous and present medical conditions

System	Previous	Patient Number (Previous history)	Present	Patient Number (Condition present)
Allergy	20	05, 06, 13, 17, 22, 24, 25, 29, 30, 35, 41, 43, 53, 54, 56, 58, 65, 74, 78, 81	18	05, 06, 13, 22, 24, 25, 29, 30, 35, 41, 43, 54, 56, 58, 65, 74, 78, 81
Cardiovascular	7	22, 24, 33, 35, 73, 76, 86	3	24, 73, 76
Dermatological	16	01, 12, 17, 25, 26, 27, 28, 34, 35, 41, 53, 55, 58, 65, 74, 81	11	01, 12, 17, 26, 28, 34, 35, 41, 58, 65, 81
Endocrine	2	23, 24	2	23, 24
ENT	29	01, 07, 08, 10, 11, 12, 13, 21, 23, 24, 27, 30, 31, 33, 34, 35, 41, 49, 53, 55, 56, 58, 74, 76, 77, 78, 82, 84, 85	17	07, 08, 12, 21, 23, 24, 27, 33, 34, 41, 49, 53, 56, 58, 74, 76, 82
GI	22	06, 08, 11, 12, 13, 21, 22, 23, 24, 25, 27, 31, 34, 35, 43, 55, 74, 78, 81, 83, 84, 86	10	21, 22, 23, 24, 34, 35, 43, 55, 74, 81
Hepatobiliary	1	78	1	82
Lymph Nodes	3	05, 33, 41	2	33, 41
Musculoskeletal	16	01, 08, 11, 21, 22, 24, 25, 31, 33, 35, 53, 74, 76, 77, 78, 84	14	01, 08, 11, 21, 22, 24, 31, 33, 35, 53, 74, 76, 77, 78
Neurological	8	01, 08, 10, 11, 22, 24, 58, 76	6	01, 08, 11, 22, 24, 58
Psychiatric	3	08, 41, 74	1	74
Renal/Genitourinary	5	05, 22, 41, 75, 76	2	75, 76
Respiratory	46	01, 05, 06, 07, 08, 09, 10, 12, 13, 17, 18, 21, 22, 23, 24, 25, 26, 27, 28, 30, 31, 33, 34, 35, 41, 42, 43, 49, 53, 54, 55, 56, 57, 58, 61, 65, 73, 74, 75, 76, 77, 78, 81, 82, 85, 86	34	01, 07, 08, 09, 10, 12, 21, 22, 23, 24, 26, 27, 28, 30, 33, 34, 35, 43, 49, 53, 55, 56, 58, 61, 65, 73, 74, 75, 76, 77, 78, 81, 85, 86
	20	05, 11, 17, 22, 25, 33, 34, 35, 35, 35, 41, 53, 53, 54, 55, 56, 61, 73, 73, 74	16	11, 17, 22, 25, 33, 34, 35, 35, 35, 41, 53, 54, 61, 73, 73, 74
Other	25	05, 07, 09, 11, 21, 22, 24, 25, 26, 27, 31, 33, 34, 35, 41, 53, 55, 56, 65, 73, 74, 77, 82, 85, 86	14	09, 11, 21, 22, 26, 33, 34, 41, 53, 65, 73, 74, 77, 86

Program: T0088.SAS, Version: 8.2, Datetime: 07FEB07:09:48

Table 14.1.14: Mean (range) baseline serum IgG levels (g/L) by age group and prior therapy (Infusions 1-4)

Group / Subgroup	All Patients		SCIG		IVIG	
	n	mean (range) IgG	n	mean (range) IgG	n	mean (range) IgG
All patients	50	9.87 (2.96-18.68)	14	8.59 (2.96-12.80)	36	10.37 (5.82-18.68)
Adults (>= 20y)	28	10.51 (5.82-18.68)	6	9.85 (9.30-12.43)	22	10.69 (5.82-18.68)
Teenagers (>= 12-<20y)	7	10.52 (7.30-12.97)	1	12.80 (12.80-12.80)	6	10.14 (7.30-12.97)
Children (<12 y)	15	8.39 (2.96-11.70)	7	6.91 (2.96- 9.70)	8	9.68 (7.45-11.70)

Program: T0485.SAS, Version: 8.2, Datetime: 07FEB07:09:48

Table 14.1.15: Mean (range) baseline serum IgG levels (g/L) by diagnosis of PAD and prior therapy (Infusions 1-4)

Group / Subgroup	All Patients n	All Patients mean (range) IgG	SCIG n	SCIG mean (range) IgG	IVIG n	IVIG mean (range) IgG
All patients	50	9.87 (2.96-18.68)	14	8.59 (2.96-12.80)	36	10.37 (5.82-18.68)
CVID/XLA	33	9.41 (2.96-15.85)	9	8.40 (2.96-12.80)	24	9.79 (5.82-15.85)
Other	17	10.77 (6.08-18.68)	5	8.94 (7.00- 9.70)	12	11.54 (6.08-18.68)
CVID	30	9.87 (5.82-15.85)	6	10.19 (7.84-12.80)	24	9.79 (5.82-15.85)
XLA	3	4.83 (2.96- 6.51)	3	4.83 (2.96- 6.51)	0	
IgG subclass deficiency	5	10.74 (9.30-14.63)	3	9.46 (9.30- 9.70)	2	12.67 (10.70-14.63)
Specific antibody deficiency	7	12.79 (7.90-18.68)	1	9.30 (9.30- 9.30)	6	13.38 (7.90-18.68)
Ataxia Telangiectasia	1	7.00 (7.00- 7.00)	1	7.00 (7.00- 7.00)	0	
CD40 Ligand Deficiency	2	7.81 (7.45- 8.17)	0		2	7.81 (7.45- 8.17)
IgG Heavy Chain Deficiency	1	6.08 (6.08- 6.08)	0		1	6.08 (6.08- 6.08)
Combined Immunodeficiency	1	11.20 (11.20-11.20)	0		1	11.20 (11.20-11.20)

Table / Figure Number	Table / Figure Name
**14.2**	**Efficacy Data**
**14.2.1**	**Serum IgG Levels in Stage 1**
Table 14.2.1.1	Number of patients with IgG levels <4g/L and <6g/L in Stage 1
Table 14.2.1.2	Time taken to reach steady state – by prior therapy
Table 14.2.1.3	Serum IgG levels (g/L) in Stage 1 of the study, listed by infusion number – all patients
Table 14.2.1.4	Serum IgG levels (g/L) in Stage 1 of the study, listed by infusion number - by diagnosis of PAD
Table 14.2.1.5	Serum IgG levels (g/L) in Stage 1 of the study, listed by infusion number - by age group
Table 14.2.1.6	Serum IgG levels (g/L) in Stage 1 of the study, listed by infusion number - by prior therapy
Table 14.2.1.7	Mean serum IgG levels in Stage 1 of the study – all patients
Table 14.2.1.8	Mean serum IgG levels in Stage 1 of the study – by diagnosis of PAD
Figure 14.2.1.9	'Mean of means' serum IgG levels in Stage 1 of the study – by diagnosis of PAD
Table 14.2.1.10	Mean serum IgG levels in Stage 1 of the study – by age group
Figure 14.2.1.11	'Mean of means' serum IgG levels in Stage 1 of the study – by age group
Table 14.2.1.12	Mean serum IgG levels in Stage 1 of the study – by prior therapy
Figure 14.2.1.13	'Mean of means' serum IgG levels in Stage 1 of the study – by prior therapy
**14.2.2**	**Serum IgG Levels Across the Whole Study**
Table 14.2.2.1	Number of patients with serum IgG levels <4g/L and <6g/L across the whole study
Table 14.2.2.2	Mean serum IgG levels (g/L) by 6-monthly intervals – all patients
Table 14.2.2.3	Mean serum IgG levels (g/L) by 6-monthly intervals – completers
Figure 14.2.2.4	'Mean of means' serum IgG levels (g/L) in 6-monthly intervals – all patients and completers
Table 14.2.2.5	Mean serum IgG levels (g/L) by 6-monthly intervals – by diagnosis of PAD, all patients
Figure 14.2.2.6	'Mean of means' serum IgG levels (g/L) in 6-monthly intervals – by diagnosis of PAD, all patients
Table 14.2.2.7	Mean serum IgG levels (g/L) by 6-monthly intervals – by age group, all patients
Figure 14.2.2.8	'Mean of means' serum IgG levels (g/L) in 6-monthly intervals – by age group, all patients
Table 14.2.2.9	Mean serum IgG levels (g/L) by 6-monthly intervals – by prior therapy, all patients
Figure 14.2.2.10	'Mean of means' serum IgG levels (g/L) in 6-monthly intervals – by prior therapy, all patients
**14.2.3**	**Pharmacokinetic Data**
Table 14.2.3.1	Daily inter-infusion total serum IgG values (g/L)

**14.2.4**	**Dose of Subgam Received in Stage 1**
Table 14.2.4.1	Mean (range) Subgam dose (mg/kg) received in Stage 1 of the study – all patients
Table 14.2.4.2	Mean Subgam dose (mg/kg) received in Stage 1 of the study – by diagnosis of PAD
Table 14.2.4.3	Mean Subgam dose (mg/kg) received in Stage 1 of the study – by age group
Table 14.2.4.4	Mean Subgam dose (mg/kg) received in Stage 1 of the study – by prior therapy
**14.2.5**	**Dose of Subgam Received Across the Whole Study**
Table 14.2.5.1	Mean (range) Subgam dose (mg/kg) received in 6-monthly intervals – all patients
Table 14.2.5.2	Mean (range) Subgam dose (mg/kg) received in 6-monthly intervals - completers
Figure 14.2.5.3	'Mean of means' of Subgam dosage (mg/kg) received in 6-monthly intervals – all patients and completers
Table 14.2.5.4	Mean Subgam dose (mg/kg) received in 6-monthly intervals – by diagnosis of PAD, all patients
Figure 14.2.5.5	'Mean of means' of Subgam dosage (mg/kg) received in 6-monthly intervals – by diagnosis of PAD, all patients
Table 14.2.5.6	Mean Subgam dose (mg/kg) received in 6-monthly intervals – by age group, all patients
Figure 14.2.5.7	'Mean of means' of Subgam dosage (mg/kg) received in 6-monthly intervals - by age group, all patients
Table 14.2.5.8	Mean Subgam dose (mg/kg) received in 6-monthly intervals – by prior therapy
Figure 14.2.5.9	'Mean of means' of Subgam dosage (mg/kg) received in 6-monthly intervals – by prior therapy, all patients
**14.2.6**	**Changes in Subgam Dose Across the Whole Study**
Table 14.2.6.1	Number of increases and decreases in Subgam dose (ml) across the whole study – all patients
Table 14.2.6.2	Number of increases and decreases in Subgam dose (ml) across the whole study – by diagnosis of PAD
Table 14.2.6.3	Number of increases and decreases in Subgam dose (ml) across the whole study – by age group
Table 14.2.6.4	Number of increases and decreases in Subgam dose (ml) across the whole study – by prior therapy
Table 14.2.6.5	Min and max Subgam dose (as a % of the starting dose) – all patients
Table 14.2.6.6	Min and max Subgam dose (as a % of the starting dose) – completers
Table 14.2.6.7	Min and max Subgam dose (as a % of the starting dose) – by diagnosis of PAD
Table 14.2.6.8	Min and max Subgam dose (as a % of the starting dose) – by age group
Table 14.2.6.9	Min and max Subgam dose (as a % of the starting dose) – by prior therapy
Table 14.2.6.10	Min and max Subgam dose (as a % of the starting dose) in 6-monthly intervals – all patients
Table 14.2.6.11	Min and max Subgam dose (as a % of the starting dose) in 6-monthly

	intervals – completers
Table 14.2.6.12	Min and max Subgam dose (as a % of the starting dose) in 6-monthly intervals – by diagnosis of PAD
Table 14.2.6.13	Min and max Subgam dose (as a % of the starting dose) in 6-monthly intervals – by age group
Table 14.2.6.14	Min and max Subgam dose (as a % of the starting dose) in 6-monthly intervals – by prior therapy
**14.2.7**	**Dose-Corrected Mean Serum IgG Levels**
Table 14.2.7.1	Dose-corrected mean serum IgG (g.kg/L.mg) in Stage 1, all patients
Table 14.2.7.2	Dose-corrected mean serum IgG (g.kg/L.mg) across the whole study, all patients
Table 14.2.7.3	Dose-corrected mean serum IgG (g.kg/L.mg) across the whole study, completers
Figure 14.2.7.4	'Mean of means' of dose-corrected serum IgG across the whole study - all patients and completers
Table 14.2.7.5	Dose-corrected mean serum IgG (g.kg/L.mg) across the whole study - by diagnosis of PAD, all patients
Figure 14.2.7.6	'Mean of means' of dose-corrected serum IgG across the whole study - by diagnosis of PAD, all patients
Table 14.2.7.7	Dose-corrected mean serum IgG (g.kg/L.mg) across the whole study - by age group, all patients
Figure 14.2.7.8	'Mean of means' of dose-corrected serum IgG across the whole study - by age group, all patients
Table 14.2.7.9	Dose-corrected mean serum IgG (g.kg/L.mg) across the whole study - by prior therapy, all patients
Figure 14.2.7.10	'Mean of means' of dose-corrected serum IgG across the whole study - by previous therapy, all patients
**14.2.8**	**Interval Between Infusions and Total Subgam Exposure**
Table 14.2.8.1	Interval between infusions of Subgam (days) – all patients
Table 14.2.8.2	Interval between infusions of Subgam (days) – by diagnosis, age group and prior therapy
Table 14.2.8.3	Total Subgam exposure over the whole study – all patients
Table 14.2.8.4	Total Subgam exposure over the whole study – by diagnosis
Table 14.2.8.5	Total Subgam exposure over the whole study – by age group
Table 14.2.8.6	Total Subgam exposure over the whole study – by prior therapy
**14.2.9**	**Specific Antibodies and Immunology Parameters**
Table 14.2.9.1	Summary statistics for Anti-HIB (mg/L) in Stage 1
Table 14.2.9.2	Summary statistics for serum IgA levels (g/L) in Stage 1 – all patients
Table 14.2.9.3	Summary statistics for serum IgM levels (g/L) in Stage 1 – all patients
**14.2.10**	**Number of Infections by Type**
Table 14.2.10.1	Infections by system organ class and preferred term
Table 14.2.10.2a	Numbers of all infections Pre-Subgam (Pre-Study–Infusion 3)- by type, severity and age group
Table 14.2.10.2b	Numbers of all infections Pre-Subgam (Pre-study–Infusion 3) – all patients
Table 14.2.10.2c	Numbers of all infections Pre-Subgam (Pre-study–Infusion 3) – by diagnosis

	of PAD, age group and prior therapy
Table 14.2.10.2d	Numbers of all infections by type, severity and age group - Post-Subgam (Infusions 4-Last Subgam Infusion) -
Table 14.2.10.2e	Numbers of all infections Post-Subgam (Infusion 4– Last Subgam Infusion) – all patients
Table 14.2.10.2f	Numbers of all infections Post-Subgam (Infusion 4– Last Subgam Infusion) – by diagnosis of PAD, age group and prior therapy
Table 14.2.10.3	Number of non-serious infections (reported as ICH-defined non-serious adverse events)
Table 14.2.10.3	Number of serious infections (reported as ICH-defined non-serious adverse events)
Table 14.2.10.3a	Pre-Subgam (Pre-study–Infusion 3) - by type, severity and age group
Table 14.2.10.3b	Pre-Subgam (Pre-study–Infusion 3) – all patients
Table 14.2.10.3c	Pre-Subgam (Pre-study–Infusion 3) – by diagnosis of PAD, age group and prior therapy
Table 14.2.10.3d	By type, severity and age group - Post-Subgam (Infusions 4- Last Subgam Infusion)
Table 14.2.10.3e	Post-Subgam (Infusion 4– Last Subgam Infusion) – all patients
Table 14.2.10.3f	Post-Subgam (Infusion 4– Last Subgam Infusion) – by diagnosis of PAD, age group and prior therapy
Table 14.2.10.4	Number of serious infections (reported as ICH-defined serious adverse events)
Table 14.2.10.4a	Pre-Subgam (Pre-study–Infusion 3) - by type, severity and age group
Table 14.2.10.4b	Pre-Subgam (Pre-study–Infusion 3) – all patients
Table 14.2.10.4c	Pre-Subgam (Pre-study–Infusion 3) – by diagnosis of PAD, age group and prior therapy
Table 14.2.10.4d	By type, severity and age group - Post-Subgam (Infusions 4- Last Subgam Infusion)
Table 14.2.10.4e	Post-Subgam (Infusion 4– Last Subgam Infusion) – all patients
Table 14.2.10.4f	Post-Subgam (Infusion 4– Last Subgam Infusion) – by diagnosis of PAD, age group and prior therapy
Table 14.2.10.5	Summary of all infections by higher level term, preferred term and seriousness – post-Subgam (Pre-study–Infusion 3)
Table 14.2.10.6	Summary of all infections by higher level term, preferred term and seriousness – post-Subgam (Infusion 4-Last Subgam Infusion)
Table 14.2.10.7	Number of 'serious acute bacterial infections' documented in the study – post-Subgam (Infusion 4-EOS)
Table 14.2.10.8	Number of infections – by type and onset month
**14.2.11**	**Number of Study Days on Antibiotics**
Table 14.2.11.1	Total number and percentage of study days on antibiotics by antibiotic category; in yearly intervals
Table 14.2.11.2	Mean number and percentage of study days on antibiotics and antibiotic-free days; in yearly intervals
Table 14.2.11.3	Number and percentage of study days on antibiotics– by diagnosis of PAD, age group and prior therapy
Table 14.2.11.4	Number and percentage of study days on all antibiotics, pre- and post-

	Subgam – all patients
Table 14.2.11.5	Number and percentage of study days on all antibiotics, pre- and post-Subgam – by diagnosis of PAD
Table 14.2.11.6	Number and percentage of study days on all antibiotics, pre- and post-Subgam – by age group
Table 14.2.11.7	Number and percentage of study days on all antibiotics, pre- and post-Subgam – by prior therapy
Table 14.2.11.8	Number and percentage of study days on all antibiotics in yearly intervals
Table 14.2.11.8a	all patients
Table 14.2.11.8b	by diagnosis of PAD
Table 14.2.11.8c	by age group
Table 14.2.11.8d	by prior therapy
Table 14.2.11.9	Number and percentage of study days on long-term prophylactic antibiotics in yearly intervals
Table 14.2.11.9a	all patients
Table 14.2.11.9b	by diagnosis of PAD
Table 14.2.11.9c	by age group
Table 14.2.11.9d	by prior therapy
Table 14.2.11.10	Number and percentage of study days on intravenous antibiotics in yearly intervals
Table 14.2.11.10a	all patients
Table 14.2.11.10b	by diagnosis of PAD
Table 14.2.11.10c	by age group
Table 14.2.11.10d	by prior therapy
Table 14.2.11.11	Number and percentage of study days on acute antibiotics in yearly intervals
Table 14.2.11.11a	all patients
Table 14.2.11.11b	by diagnosis of PAD
Table 14.2.11.11c	by age group
Table 14.2.11.11d	by prior therapy
Table 14.2.11.12	Number and percentage of study days on other antibiotics in yearly intervals
Table 14.2.11.12a	all patients
Table 14.2.11.12b	by diagnosis of PAD
Table 14.2.11.12c	by age group
Table 14.2.11.12d	by prior therapy

**14.2.12**	**Days off Work or School**
Table 14.2.12.1	Number of days off work or school (applicable patients only)
Table 14.2.12.2	Summary statistics for number of days off work or school (applicable patients only)
Table 14.2.12.3	Number of days off work or school – all patients
Table 14.2.12.4	Summary statistics for number of days off work or school – by diagnosis of PAD
Table 14.2.12.5	Summary statistics for number of days off work or school – by age group
Table 14.2.12.6	Summary statistics for number of days off work or school – by prior therapy
**14.2.13**	**Days Spent in the Study and on Home Therapy**
Table 14.2.13.1	Time in relation to starting Subgam that patients began home therapy, by prior therapy
Table 14.2.13.2	Number of days spent on home therapy for each patient – by diagnosis of PAD
Table 14.2.13.3	Number of days spent on home therapy for each patient – by age group
Table 14.2.13.4	Number of days spent on home therapy for each patient – by prior therapy
Table 14.2.13.5	Number and percentage of Subgam infusions given as home therapy – all patients
Table 14.2.13.6	Number and percentage of Subgam infusions given as home therapy – by diagnosis of PAD
Table 14.2.13.7	Number and percentage of Subgam infusions given as home therapy – by age group
Table 14.2.13.8	Number and percentage of Subgam infusions given as home therapy – by prior therapy
**14.2.14**	**Patient Satisfaction with Subgam**
Table 14.2.14.1	How patients liked Subgam overall in comparison to their previous medication – by diagnosis, age group and prior therapy : After 3 months on Subgam
Table 14.2.14.2	How patients liked Subgam overall in comparison to their previous medication – by diagnosis, age group and prior therapy : After 6 months on Subgam
Table 14.2.14.3	Patients' perception of their symptoms while on Subgam compared with their previous medication – by diagnosis, age group and prior therapy : After 3 months on Subgam
Table 14.2.14.4	Patients' perception of their symptoms while on Subgam compared with their previous medication – by diagnosis, age group and prior therapy : After 6 months on Subgam
Table 14.2.14.5	Patients' perceptions of convenience of administering Subgam compared with that of prior therapy – After 3 months on Subgam
Table 14.2.14.5a	all patients
Table 14.2.14.5b	by diagnosis of PAD
Table 14.2.14.5c	by age group
Table 14.2.14.5d	by prior therapy
Table 14.2.14.6	Patients' perceptions of convenience of administering Subgam compared with that of prior therapy – After 6 months on Subgam

Table 14.2.14.6a	all patients
Table 14.2.14.6b	by diagnosis of PAD
Table 14.2.14.6c	by age group
Table 14.2.14.6d	by prior therapy
Table 14.2.14.7	Patient's perceptions of comfort of administering Subgam compared with that of prior therapy – After 3 months on Subgam
Table 14.2.14.7a	all patients
Table 14.2.14.7b	by diagnosis of PAD
Table 14.2.14.7c	by age group
Table 14.2.14.7d	by prior therapy
Table 14.2.14.8	Patient's perceptions of comfort of administering Subgam compared with that of prior therapy – After 6 months on Subgam
Table 14.2.14.8a	all patients
Table 14.2.14.8b	by diagnosis of PAD
Table 14.2.14.8c	by age group
Table 14.2.14.8d	by prior therapy

Table 14.2.1.1: Number of patients with IgG levels <4g/L and <6g/L in Stage 1

		All patients	Children (<12 years)		Teenagers and adults (>=12y)	
	Infusion	n	n	Number of patients (patient numbers) with IgG < 4g/L	n	Number of patients (patient numbers) with IgG < 6g/L
Pre-Subgam	1	9	2	0	7	1 (34)
	2	41	8	0	33	2 (17,18)
	3	42	11	1 (57)	31	2 (17,18)
	3A	2	0	0	2	0
	3B	2	0	0	2	0
Post-Subgam	4	49	15	1 (57)	34	0
	5	47	13	0	34	0
	6	40	7	0	33	0
	7	35	4	1 (57)	31	0
	8	35	6	0	29	0
	9	34	6	0	28	1 (10)
	10	26	0	0	26	2 (10,18)
	11	25	3	1 (57)	22	2 (10,18)
	12	29	8	0	21	1 (18)
	13	13	2	0	11	1 (10)
	14	9	0	0	9	1 (10)
	15	14	3	0	11	0
	16	27	8	0	19	0
	17	8	1	0	7	0
	18	11	1	0	10	1 (18)
	19	14	2	0	12	1 (18)
	20	24	7	0	17	1 (18)

Certain data have been excluded from the efficacy analysis. See filenote 001, Section 16.1.9, for details of data exclusions from this table

Program: T0344.SAS, Version: 8.2, Datetime: 07FEB07:09:48

Table 14.2.1.1: Number of patients with IgG levels <4g/L and <6g/L in Stage 1

	Infusion	All patients	Children (<12 years)		Teenagers and adults (>=12y)	
		n	n	Number of patients (patient numbers) with IgG < 4g/L	n	Number of patients (patient numbers) with IgG < 6g/L
Post-Subgam	21	9	1	0	8	0
	22	9	2	0	7	0
	23	12	3	0	9	0
	24	23	8	0	15	1 (84)
	25	10	1	0	9	0
	26	7	1	0	6	0
	27	11	1	0	10	1 (31)
	28	19	6	0	13	1 (18)
	29	9	1	0	8	1 (34)
	30	12	4	0	8	0
	31	24	8	0	16	1 (18)
	All infusions post-Subgam	585	122	1 (57)	463	5 (10,18,31,34,84)

Certain data have been excluded from the efficacy analysis. See filenote 001, Section 16.1.9, for details of data exclusions from this table

Program: T0344.SAS, Version: 8.2, Datetime: 07FEB07:09:48

Table 14.2.1.2: Time taken to reach steady state - by prior therapy

IVIG / SCIG	Patient	Infusion number at which steady state was reached(a)
IVIG	07	5
	09	9
	10	5
	11	5
	17	5
	18	5
	21	6
	22	6
	23	8
	24	5
	29	5
	30	12
	31	7
	33	7
	34	5
	35	5
	41	6
	42	6
	49	7
	54	6
	55	5
	56	5
	n	22
	Mean	6.14
	Median	5.50

(a) The first of three consecutive serum IgG levels within 1g/L of one another, from infusion 5

Certain data have been excluded from the efficacy analysis. See filenote 001, Section 16.1.9, for details of data exclusions from this table

Program: T0345.SAS, Version: 8.2, Datetime: 07FEB07:09:48

Table 14.2.1.2: Time taken to reach steady state - by prior therapy

IVIG / SCIG	Patient	Infusion number at which steady state was reached(a)
IVIG	SD	1.73
	Min	5.00
	Max	12.00
	Lower 95% CI	5.37
	Upper 95% CI	6.90

(a) The first of three consecutive serum IgG levels within 1g/L of one another, from infusion 5

Certain data have been excluded from the efficacy analysis. See filenote 001, Section 16.1.9, for details of data exclusions from this table

Program: T0345.SAS, Version: 8.2, Datetime: 07FEB07:09:48

Table 14.2.1.2: Time taken to reach steady state - by prior therapy

IVIG / SCIG	Patient	Infusion number at which steady state was reached(a)
SCIG	06	5
	53	6
	73	8
	75	7
	n	4
	Mean	6.50
	Median	6.50
	SD	1.29
	Min	5.00
	Max	8.00
	Lower 95% CI	4.45
	Upper 95% CI	8.55

(a) The first of three consecutive serum IgG levels within 1g/L of one another, from infusion 5

Certain data have been excluded from the efficacy analysis. See filenote 001, Section 16.1.9, for details of data exclusions from this table

Program: T0345.SAS, Version: 8.2, Datetime: 07FEB07:09:48

Table 14.2.1.3: Serum IgG levels (g/L) in Stage 1 of the study, listed by infusion number - all patients

	Infusion	n	Mean	Median	SD	Min	Max	Lower 95% CI	Upper 95% CI
Pre-Subgam	INF1	9	8.91	7.10	4.13	5.90	19.20	5.73	12.08
	INF2	41	9.64	9.30	2.70	5.23	17.20	8.79	10.49
	INF3	28	16.40	15.95	3.85	10.30	24.00	14.91	17.89
	INF3A	1	11.00	11.00		11.00	11.00		
	INF3B	2	12.20	12.20	2.55	10.40	14.00	-10.67	35.07
Post-Subgam	INF4	49	11.20	10.40	3.65	3.11	21.70	10.15	12.24
	INF5	47	10.82	10.40	3.13	5.25	22.50	9.90	11.73
	INF6	40	10.21	9.94	2.64	5.66	19.20	9.36	11.05
	INF7	35	10.42	10.10	2.58	3.56	16.10	9.53	11.31
	INF8	35	10.06	9.60	2.21	6.59	14.50	9.30	10.82
	INF9	34	10.07	9.45	2.79	5.89	19.70	9.10	11.05
	INF10	26	9.97	9.55	2.93	5.26	17.80	8.79	11.16
	INF11	26	9.01	8.65	2.34	3.46	13.40	8.07	9.96
	INF12	29	8.74	8.10	2.10	5.56	13.60	7.94	9.53
	INF13	12	8.48	7.65	2.50	5.51	14.20	6.89	10.07
	INF14	11	8.84	8.15	2.38	5.82	12.60	7.24	10.43
	INF15	12	7.99	7.64	1.42	6.30	11.00	7.09	8.89
	INF16	27	8.99	9.00	2.29	4.51	14.00	8.09	9.90
	INF17	8	8.66	8.09	1.82	6.40	11.50	7.14	10.18
	INF18	13	9.66	8.65	3.43	5.78	18.10	7.59	11.73
	INF19	13	9.16	8.45	2.69	5.70	14.60	7.54	10.79
	INF20	23	9.14	9.28	2.04	5.47	14.00	8.26	10.02

(a) 3 WD visits took place prior to EOP1 (see Listing 16.2.6.1). Of these, one was after infusion 31, resulting in its inclusion as Stage 2 data and its exclusion from this table.
EOP1 = End of Stage 1
WD = Hospital Visit (other than for Subgam infusion)
INF = Infusion
Certain data have been excluded from the efficacy analysis. See filenote 001, Section 16.1.9, for details of data exclusions from this table

Table 14.2.1.3: Serum IgG levels (g/L) in Stage 1 of the study, listed by infusion number - all patients

	Infusion	n	Mean	Median	SD	Min	Max	Lower 95% CI	Upper 95% CI
Post-Subgam	INF21	10	8.25	7.24	2.31	5.60	12.70	6.59	9.90
	INF22	10	7.77	7.86	1.35	6.00	9.46	6.81	8.74
	INF23	10	9.07	8.90	2.17	6.33	12.00	7.52	10.63
	INF24	22	9.71	9.55	2.25	5.84	14.80	8.71	10.71
	INF25	10	9.11	8.34	2.12	6.60	12.90	7.59	10.62
	INF26	7	7.85	7.20	1.67	6.30	10.50	6.30	9.39
	INF27	10	8.89	9.09	2.53	5.40	12.90	7.09	10.70
	INF28	20	10.03	9.36	2.55	5.80	16.80	8.84	11.23
	INF29	8	7.86	7.40	2.46	5.20	12.70	5.81	9.92
	INF30	7	7.38	7.11	0.98	6.30	9.00	6.48	8.28
	INF31	2	9.95	9.95	1.20	9.10	10.80	-0.85	20.75
	EOP1	27	9.04	9.00	2.04	4.64	12.84	8.24	9.85
	WD (a)	2	9.70	9.70	1.41	8.70	10.70	-3.01	22.41

(a) 3 WD visits took place prior to EOP1 (see Listing 16.2.6.1). Of these, one was after infusion 31, resulting in its inclusion as Stage 2 data and its exclusion from this table.
EOP1 = End of Stage 1
WD = Hospital Visit (other than for Subgam infusion)
INF = Infusion
Certain data have been excluded from the efficacy analysis. See filenote 001, Section 16.1.9, for details of data exclusions from this table

Program: T0346.TEM, Version: 8.2, Datetime: 07FEB07:09:48

Table 14.2.1.4: Serum IgG levels (g/L) in Stage 1 of the study, listed by infusion number - by diagnosis of PAD

	Infusion	Diagnosis of PAD	n	Mean	Median	SD	Min	Max	Lower 95% CI	Upper 95% CI
Pre-Subgam	INF1	CVID/XLA	6	6.82	6.91	0.58	5.90	7.60	6.22	7.42
		Other	3	13.08	10.20	5.31	9.83	19.20	-0.10	26.26
	INF2	CVID/XLA	29	9.03	8.48	2.31	5.23	13.90	8.15	9.91
		Other	12	11.14	10.20	3.06	5.68	17.20	9.19	13.08
	INF3	CVID/XLA	20	16.26	16.15	3.61	10.30	23.40	14.57	17.95
		Other	8	16.75	14.80	4.65	12.70	24.00	12.86	20.64
	INF3A	CVID/XLA	1	11.00	11.00		11.00	11.00		
	INF3B	CVID/XLA	1	10.40	10.40		10.40	10.40		
		Other	1	14.00	14.00		14.00	14.00		
Post-Subgam	INF4	CVID/XLA	32	10.74	10.50	3.22	3.11	17.80	9.58	11.90
		Other	17	12.06	9.70	4.31	7.00	21.70	9.84	14.27
	INF5	CVID/XLA	31	10.29	10.50	2.44	5.25	15.50	9.40	11.19
		Other	16	11.83	10.30	4.06	7.14	22.50	9.66	13.99
	INF6	CVID/XLA	27	9.83	9.58	2.12	5.66	13.50	9.00	10.67
		Other	13	10.99	10.50	3.47	7.39	19.20	8.90	13.08
	INF7	CVID/XLA	25	10.09	9.88	2.54	3.56	14.40	9.05	11.14
		Other	10	11.24	10.90	2.64	7.20	16.10	9.35	13.13
	INF8	CVID/XLA	23	9.76	9.60	1.92	6.59	12.50	8.93	10.59
		Other	12	10.63	10.45	2.69	7.55	14.50	8.93	12.34
	INF9	CVID/XLA	22	9.41	9.20	2.04	5.89	13.00	8.51	10.32
		Other	12	11.29	10.36	3.60	7.30	19.70	9.00	13.57
	INF10	CVID/XLA	19	9.01	9.00	2.05	5.26	13.30	8.02	9.99
		Other	7	12.59	13.10	3.50	7.95	17.80	9.35	15.83

INF = Infusion
EOP1 = End of Stage 1
WD = Hospital Visit (other than for Subgam infusion)

Certain data have been excluded from the efficacy analysis. See filenote 001, Section 16.1.9, for details of data exclusions from this table

Program: T0347.TEM, Version: 8.2, Datetime: 07FEB07:09:48

Table 14.2.1.4: Serum IgG levels (g/L) in Stage 1 of the study, listed by infusion number - by diagnosis of PAD

	Infusion	Diagnosis of PAD	n	Mean	Median	SD	Min	Max	Lower 95% CI	Upper 95% CI
Post-Subgam	INF11	CVID/XLA	20	8.52	8.50	2.21	3.46	12.00	7.49	9.55
		Other	6	10.66	10.35	2.18	7.94	13.40	8.37	12.94
	INF12	CVID/XLA	17	8.38	8.01	1.66	5.56	11.80	7.53	9.23
		Other	12	9.24	8.80	2.59	6.40	13.60	7.59	10.89
	INF13	CVID/XLA	9	7.93	7.60	2.01	5.51	11.73	6.39	9.48
		Other	3	10.10	8.71	3.61	7.40	14.20	1.14	19.07
	INF14	CVID/XLA	7	8.41	7.60	2.24	5.82	11.80	6.34	10.48
		Other	4	9.59	9.63	2.77	6.50	12.60	5.19	13.99
	INF15	CVID/XLA	9	8.02	7.40	1.64	6.30	11.00	6.77	9.28
		Other	3	7.89	7.87	0.60	7.30	8.50	6.40	9.38
	INF16	CVID/XLA	16	8.63	8.70	1.56	6.22	11.20	7.81	9.46
		Other	11	9.52	9.00	3.08	4.51	14.00	7.45	11.59
	INF17	CVID/XLA	7	8.79	8.17	1.92	6.40	11.50	7.02	10.57
		Other	1	7.74	7.74		7.74	7.74		
	INF18	CVID/XLA	9	8.21	7.60	1.99	5.78	11.80	6.68	9.74
		Other	4	12.91	12.45	4.01	8.65	18.10	6.54	19.29
	INF19	CVID/XLA	9	8.47	7.50	2.17	5.70	12.40	6.80	10.14
		Other	4	10.71	10.48	3.42	7.30	14.60	5.27	16.15
	INF20	CVID/XLA	14	8.89	9.21	2.09	5.47	12.40	7.69	10.10
		Other	9	9.53	9.80	2.03	7.19	14.00	7.97	11.09
	INF21	CVID/XLA	6	8.15	7.24	2.36	6.20	12.70	5.67	10.63
		Other	4	8.39	8.29	2.57	5.60	11.40	4.30	12.48
	INF22	CVID/XLA	8	7.87	7.99	1.38	6.00	9.46	6.72	9.03
		Other	2	7.37	7.37	1.65	6.20	8.54	-7.50	22.24

INF = Infusion
EOP1 = End of Stage 1
WD = Hospital Visit (other than for Subgam infusion)

Certain data have been excluded from the efficacy analysis. See filenote 001, Section 16.1.9, for details of data exclusions from this table

Program: T0347.TEM, Version: 8.2, Datetime: 07FEB07:09:48

Table 14.2.1.4: Serum IgG levels (g/L) in Stage 1 of the study, listed by infusion number - by diagnosis of PAD

	Infusion	Diagnosis of PAD	n	Mean	Median	SD	Min	Max	Lower 95% CI	Upper 95% CI
Post-Subgam	INF23	CVID/XLA	4	9.75	10.10	2.41	6.80	12.00	5.92	13.58
		Other	6	8.62	8.33	2.10	6.33	12.00	6.42	10.82
	INF24	CVID/XLA	13	8.95	8.80	2.05	5.84	11.50	7.72	10.19
		Other	9	10.81	10.10	2.17	8.86	14.80	9.14	12.48
	INF25	CVID/XLA	8	9.07	8.34	2.30	6.60	12.90	7.15	11.00
		Other	2	9.24	9.24	1.78	7.98	10.50	-6.77	25.25
	INF26	CVID/XLA	4	8.05	7.40	1.66	6.90	10.50	5.41	10.69
		Other	3	7.57	6.52	2.02	6.30	9.90	2.56	12.59
	INF27	CVID/XLA	6	7.98	7.13	2.65	5.40	12.40	5.20	10.75
		Other	4	10.27	9.60	1.81	8.97	12.90	7.39	13.15
	INF28	CVID/XLA	12	9.44	9.19	1.87	5.80	13.40	8.25	10.63
		Other	8	10.93	9.61	3.27	7.87	16.80	8.19	13.66
	INF29	CVID/XLA	5	6.44	6.20	1.02	5.20	7.90	5.18	7.70
		Other	3	10.24	10.00	2.35	8.01	12.70	4.39	16.08
	INF30	CVID/XLA	4	7.05	6.91	0.55	6.59	7.80	6.18	7.92
		Other	3	7.82	8.15	1.38	6.30	9.00	4.39	11.25
	INF31	CVID/XLA	1	10.80	10.80		10.80	10.80		
		Other	1	9.10	9.10		9.10	9.10		
	EOP1	CVID/XLA	19	8.96	8.90	2.16	4.64	12.84	7.92	10.01
		Other	8	9.24	9.07	1.84	6.31	12.30	7.70	10.78
	WD	CVID/XLA	2	9.70	9.70	1.41	8.70	10.70	-3.01	22.41

INF = Infusion
EOP1 = End of Stage 1
WD = Hospital Visit (other than for Subgam infusion)

Certain data have been excluded from the efficacy analysis. See filenote 001, Section 16.1.9, for details of data exclusions from this table

Program: T0347.TEM, Version: 8.2, Datetime: 07FEB07:09:48

Table 14.2.1.5: Serum IgG levels (g/L) in Stage 1 of the study, listed by infusion number - by age group

	Infusion	Age group	n	Mean	Median	SD	Min	Max	Lower 95% CI	Upper 95% CI
Pre-Subgam	INF1	Adult	6	9.39	7.28	5.03	5.90	19.20	4.11	14.67
		Teenager	1	7.10	7.10		7.10	7.10		
		Child	2	8.35	8.35	2.10	6.86	9.83	-10.52	27.21
	INF2	Adult	27	9.98	9.30	2.96	5.23	17.20	8.81	11.15
		Teenager	6	9.80	9.49	2.24	7.20	12.80	7.45	12.15
		Child	8	8.41	8.91	1.80	5.28	10.28	6.90	9.91
	INF3	Adult	16	17.32	17.60	4.76	10.30	24.00	14.78	19.85
		Teenager	5	16.10	16.00	0.42	15.60	16.70	15.58	16.62
		Child	7	14.52	14.00	1.86	12.70	17.30	12.80	16.24
	INF3A	Adult	1	11.00	11.00		11.00	11.00		
	INF3B	Adult	2	12.20	12.20	2.55	10.40	14.00	-10.67	35.07
Post-Subgam	INF4	Adult	27	12.02	10.60	3.87	6.95	21.70	10.48	13.55
		Teenager	7	12.01	12.30	2.14	8.40	14.20	10.03	13.99
		Child	15	9.34	9.20	3.24	3.11	13.90	7.55	11.13
	INF5	Adult	27	11.19	10.20	3.56	6.59	22.50	9.78	12.59
		Teenager	7	11.57	11.60	1.80	8.20	13.50	9.91	13.24
		Child	13	9.64	9.29	2.52	5.25	14.10	8.12	11.16
	INF6	Adult	27	10.59	10.30	2.78	6.57	19.20	9.49	11.69
		Teenager	6	11.06	11.10	1.57	9.10	12.90	9.42	12.71
		Child	7	8.01	7.89	1.72	5.66	11.15	6.43	9.60
	INF7	Adult	25	10.41	9.51	2.52	6.31	16.10	9.37	11.45
		Teenager	6	11.20	11.60	2.00	8.00	13.50	9.10	13.30

Adults (>=20y), Teenagers (>=12-<20y), Children (<12y)
INF = Infusion
EOP1 = End of Stage 1
WD = Hospital Visit (other than for Subgam infusion)

Certain data have been excluded from the efficacy analysis. See filenote 001, Section 16.1.9, for details of data exclusions from this table

Program: T0348.TEM, Version: 8.2, Datetime: 07FEB07:09:48

Table 14.2.1.5: Serum IgG levels (g/L) in Stage 1 of the study, listed by infusion number - by age group

	Infusion	Age group	n	Mean	Median	SD	Min	Max	Lower 95% CI	Upper 95% CI
Post-Subgam	INF7	Child	4	9.34	10.90	3.90	3.56	12.00	3.14	15.54
	INF8	Adult	23	10.07	9.10	2.36	6.59	14.50	9.04	11.09
		Teenager	6	10.16	9.85	2.11	7.40	12.70	7.94	12.37
		Child	6	9.94	10.15	2.09	7.58	12.40	7.75	12.13
	INF9	Adult	24	10.23	9.35	3.06	5.89	19.70	8.94	11.53
		Teenager	4	10.38	10.70	2.49	7.50	12.60	6.42	14.33
		Child	6	9.24	9.76	1.83	6.72	11.19	7.32	11.16
	INF10	Adult	22	10.06	9.55	3.01	5.26	17.80	8.73	11.40
		Teenager	4	9.48	8.85	2.81	7.10	13.10	5.00	13.95
	INF11	Adult	18	8.83	8.65	1.93	5.45	12.90	7.88	9.79
		Teenager	5	10.30	10.50	2.55	7.10	13.40	7.13	13.47
		Child	3	7.95	8.49	4.25	3.46	11.90	-2.60	18.50
	INF12	Adult	17	8.99	8.19	2.13	5.56	13.10	7.90	10.09
		Teenager	4	10.05	9.31	2.66	8.00	13.60	5.82	14.29
		Child	8	7.53	7.23	1.19	6.40	9.46	6.53	8.53
	INF13	Adult	7	7.86	7.70	1.58	5.51	9.90	6.40	9.32
		Teenager	3	9.18	7.00	4.36	6.35	14.20	-1.64	20.01
		Child	2	9.57	9.57	3.06	7.40	11.73	-17.94	37.07
	INF14	Adult	8	8.81	8.78	2.25	5.82	11.80	6.93	10.69
		Teenager	2	10.10	10.10	3.54	7.60	12.60	-21.67	41.87
		Child	1	6.50	6.50		6.50	6.50		
	INF15	Adult	7	7.91	7.87	1.24	6.61	10.10	6.77	9.06
		Teenager	3	8.23	7.40	2.46	6.30	11.00	2.13	14.34

Adults (>=20y), Teenagers (>=12-<20y), Children (<12y)
INF = Infusion
EOP1 = End of Stage 1
WD = Hospital Visit (other than for Subgam infusion)

Certain data have been excluded from the efficacy analysis. See filenote 001, Section 16.1.9, for details of data exclusions from this table

Program: T0348.TEM, Version: 8.2, Datetime: 07FEB07:09:48

Table 14.2.1.5: Serum IgG levels (g/L) in Stage 1 of the study, listed by infusion number - by age group

	Infusion	Age group	n	Mean	Median	SD	Min	Max	Lower 95% CI	Upper 95% CI
Post-Subgam	INF15	Child	2	7.90	7.90	0.85	7.30	8.50	0.28	15.52
	INF16	Adult	15	9.55	9.60	2.14	7.01	14.00	8.37	10.74
		Teenager	4	8.53	7.66	2.80	6.22	12.60	4.08	12.98
		Child	8	8.18	8.95	2.33	4.51	11.20	6.23	10.13
	INF17	Adult	5	8.22	7.74	1.95	6.40	11.50	5.81	10.64
		Teenager	2	9.60	9.60	2.26	8.00	11.20	-10.73	29.93
		Child	1	8.98	8.98		8.98	8.98		
	INF18	Adult	10	10.38	10.10	3.62	5.78	18.10	7.79	12.97
		Teenager	2	7.30	7.30	0.57	6.90	7.70	2.22	12.38
		Child	1	7.17	7.17		7.17	7.17		
	INF19	Adult	8	8.56	7.98	2.72	5.70	14.60	6.28	10.83
		Teenager	3	10.77	12.40	2.92	7.40	12.50	3.52	18.01
		Child	2	9.18	9.18	2.66	7.30	11.06	-14.71	33.07
	INF20	Adult	14	9.45	9.60	2.06	5.59	14.00	8.26	10.65
		Teenager	2	8.22	8.22	1.30	7.30	9.14	-3.47	19.91
		Child	7	8.78	9.28	2.26	5.47	12.40	6.69	10.88
	INF21	Adult	5	8.55	8.60	2.05	6.20	11.40	6.01	11.09
		Teenager	3	8.95	7.26	3.25	6.90	12.70	0.88	17.03
		Child	2	6.41	6.41	1.15	5.60	7.22	-3.88	16.70
	INF22	Adult	8	7.76	7.86	1.26	6.00	9.10	6.70	8.81
		Child	2	7.83	7.83	2.31	6.20	9.46	-12.88	28.54
	INF23	Adult	6	8.79	8.90	1.75	6.80	11.40	6.95	10.62
		Teenager	2	12.00	12.00	0.00	12.00	12.00	12.00	12.00

Adults (>=20y), Teenagers (>=12-<20y), Children (<12y)
INF = Infusion
EOP1 = End of Stage 1
WD = Hospital Visit (other than for Subgam infusion)

Certain data have been excluded from the efficacy analysis. See filenote 001, Section 16.1.9, for details of data exclusions from this table

Program: T0348.TEM, Version: 8.2, Datetime: 07FEB07:09:48

Table 14.2.1.5: Serum IgG levels (g/L) in Stage 1 of the study, listed by infusion number - by age group

	Infusion	Age group	n	Mean	Median	SD	Min	Max	Lower 95% CI	Upper 95% CI
Post-Subgam	INF23	Child	2	7.00	7.00	0.94	6.33	7.66	-1.45	15.44
	INF24	Adult	13	10.17	10.10	2.45	6.44	14.80	8.68	11.65
		Teenager	1	5.84	5.84		5.84	5.84		
		Child	8	9.46	9.34	1.52	6.56	11.35	8.19	10.73
	INF25	Adult	5	9.38	8.10	1.95	7.98	12.30	6.95	11.80
		Teenager	4	8.82	7.89	2.84	6.60	12.90	4.30	13.34
		Child	1	8.90	8.90		8.90	8.90		
	INF26	Adult	6	8.10	7.40	1.67	6.52	10.50	6.35	9.86
		Child	1	6.30	6.30		6.30	6.30		
	INF27	Adult	7	8.22	8.97	1.74	5.40	10.00	6.61	9.83
		Teenager	2	12.65	12.65	0.35	12.40	12.90	9.47	15.83
		Child	1	6.10	6.10		6.10	6.10		
	INF28	Adult	12	10.49	9.40	3.22	5.80	16.80	8.44	12.54
		Teenager	1	8.23	8.23		8.23	8.23		
		Child	7	9.51	9.42	0.55	8.91	10.30	9.01	10.02
	INF29	Adult	6	8.30	7.96	2.73	5.20	12.70	5.43	11.17
		Teenager	2	6.55	6.55	0.49	6.20	6.90	2.10	11.00
	INF30	Adult	5	7.65	7.80	1.02	6.59	9.00	6.39	8.91
		Child	2	6.71	6.71	0.57	6.30	7.11	1.56	11.85
	INF31	Adult	1	10.80	10.80		10.80	10.80		
		Child	1	9.10	9.10		9.10	9.10		
	EOP1	Adult	14	8.87	8.95	1.87	4.64	11.50	7.79	9.95
		Teenager	4	10.19	10.58	2.58	7.21	12.40	6.08	14.30

Adults (>=20y), Teenagers (>=12-<20y), Children (<12y)
INF = Infusion
EOP1 = End of Stage 1
WD = Hospital Visit (other than for Subgam infusion)

Certain data have been excluded from the efficacy analysis. See filenote 001, Section 16.1.9, for details of data exclusions from this table

Program: T0348.TEM, Version: 8.2, Datetime: 07FEB07:09:48

Table 14.2.1.5: Serum IgG levels (g/L) in Stage 1 of the study, listed by infusion number - by age group

	Infusion	Age group	n	Mean	Median	SD	Min	Max	Lower 95% CI	Upper 95% CI
Post-Subgam	EOP1	Child	9	8.80	9.13	2.14	5.76	12.84	7.16	10.45
	WD	Adult	2	9.70	9.70	1.41	8.70	10.70	-3.01	22.41

Adults (>=20y), Teenagers (>=12-<20y), Children (<12y)
INF = Infusion
EOP1 = End of Stage 1
WD = Hospital Visit (other than for Subgam infusion)

Certain data have been excluded from the efficacy analysis. See filenote 001, Section 16.1.9, for details of data exclusions from this table

Program: T0348.TEM, Version: 8.2, Datetime: 07FEB07:09:48

Table 14.2.1.6: Serum IgG levels (g/L) in Stage 1 of the study, listed by infusion number - by prior therapy

	Infusion	Prior Therapy	n	Mean	Median	SD	Min	Max	Lower 95% CI	Upper 95% CI
Pre-Subgam	INF1	IVIG	7	9.07	7.10	4.67	5.90	19.20	4.74	13.39
		SCIG	2	8.35	8.35	2.10	6.86	9.83	-10.52	27.21
	INF2	IVIG	30	9.81	9.20	2.87	5.23	17.20	8.74	10.88
		SCIG	11	9.19	9.30	2.22	5.28	12.80	7.70	10.68
	INF3	IVIG	28	16.40	15.95	3.85	10.30	24.00	14.91	17.89
	INF3A	IVIG	1	11.00	11.00		11.00	11.00		
	INF3B	IVIG	2	12.20	12.20	2.55	10.40	14.00	-10.67	35.07
Post-Subgam	INF4	IVIG	35	12.26	12.20	3.48	6.95	21.70	11.07	13.46
		SCIG	14	8.53	9.05	2.59	3.11	12.80	7.04	10.03
	INF5	IVIG	35	11.38	11.10	3.23	6.59	22.50	10.28	12.49
		SCIG	12	9.16	9.45	2.17	5.25	13.50	7.78	10.54
	INF6	IVIG	30	10.56	10.35	2.73	6.57	19.20	9.54	11.58
		SCIG	10	9.17	9.05	2.14	5.66	12.60	7.63	10.70
	INF7	IVIG	28	10.56	10.35	2.37	6.31	16.10	9.64	11.48
		SCIG	7	9.87	9.10	3.46	3.56	13.70	6.66	13.07
	INF8	IVIG	30	10.22	10.30	2.30	6.59	14.50	9.36	11.07
		SCIG	5	9.12	8.70	1.43	8.10	11.60	7.34	10.90
	INF9	IVIG	25	10.48	10.40	3.07	5.89	19.70	9.21	11.74
		SCIG	9	8.96	9.30	1.39	6.72	11.30	7.89	10.03
	INF10	IVIG	23	9.95	9.42	3.11	5.26	17.80	8.61	11.30
		SCIG	3	10.13	9.60	1.01	9.50	11.30	7.62	12.65
	INF11	IVIG	20	9.14	8.55	2.25	5.45	13.40	8.08	10.20
		SCIG	6	8.59	8.95	2.81	3.46	12.00	5.64	11.54

INF = Infusion
EOP1 = End of Stage 1
WD = Hospital Visit (other than for Subgam infusion)

Certain data have been excluded from the efficacy analysis. See filenote 001, Section 16.1.9, for details of data exclusions from this table

Program: T0349.TEM, Version: 8.2, Datetime: 07FEB07:09:48

Table 14.2.1.6: Serum IgG levels (g/L) in Stage 1 of the study, listed by infusion number - by prior therapy

	Infusion	Prior Therapy	n	Mean	Median	SD	Min	Max	Lower 95% CI	Upper 95% CI
Post-Subgam	INF12	IVIG	21	8.57	8.01	2.27	5.56	13.60	7.54	9.60
		SCIG	8	9.17	8.80	1.63	7.02	11.80	7.80	10.53
	INF13	IVIG	11	8.57	7.70	2.60	5.51	14.20	6.83	10.32
		SCIG	1	7.40	7.40		7.40	7.40		
	INF14	IVIG	10	9.07	8.78	2.37	5.82	12.60	7.37	10.77
		SCIG	1	6.50	6.50		6.50	6.50		
	INF15	IVIG	10	7.64	7.35	1.14	6.30	10.10	6.82	8.45
		SCIG	2	9.75	9.75	1.77	8.50	11.00	-6.13	25.63
	INF16	IVIG	19	8.89	8.89	2.65	4.51	14.00	7.62	10.17
		SCIG	8	9.24	9.30	1.19	7.02	10.80	8.25	10.23
	INF17	IVIG	7	8.62	8.00	1.96	6.40	11.50	6.81	10.43
		SCIG	1	8.98	8.98		8.98	8.98		
	INF18	IVIG	12	9.87	9.18	3.49	5.78	18.10	7.65	12.09
		SCIG	1	7.17	7.17		7.17	7.17		
	INF19	IVIG	12	8.89	7.98	2.62	5.70	14.60	7.23	10.56
		SCIG	1	12.40	12.40		12.40	12.40		
	INF20	IVIG	15	9.09	9.14	2.23	5.59	14.00	7.86	10.33
		SCIG	8	9.23	9.60	1.77	5.47	11.70	7.76	10.71
	INF21	IVIG	7	8.59	7.26	2.50	6.20	12.70	6.28	10.90
		SCIG	3	7.44	7.22	1.96	5.60	9.50	2.57	12.31
	INF22	IVIG	9	7.59	7.17	1.29	6.00	9.10	6.59	8.58
		SCIG	1	9.46	9.46		9.46	9.46		
	INF23	IVIG	7	8.56	7.66	2.29	6.33	12.00	6.44	10.68
		SCIG	3	10.27	9.80	1.55	9.00	12.00	6.41	14.13

INF = Infusion
EOP1 = End of Stage 1
WD = Hospital Visit (other than for Subgam infusion)

Certain data have been excluded from the efficacy analysis. See filenote 001, Section 16.1.9, for details of data exclusions from this table

Program: T0349.TEM, Version: 8.2, Datetime: 07FEB07:09:48

Table 14.2.1.6: Serum IgG levels (g/L) in Stage 1 of the study, listed by infusion number - by prior therapy

	Infusion	Prior Therapy	n	Mean	Median	SD	Min	Max	Lower 95% CI	Upper 95% CI
Post-Subgam	INF24	IVIG	14	9.77	9.49	2.61	5.84	14.80	8.27	11.27
		SCIG	8	9.61	9.75	1.61	6.56	11.50	8.27	10.95
	INF25	IVIG	8	8.96	8.05	2.33	6.60	12.90	7.01	10.91
		SCIG	2	9.70	9.70	1.13	8.90	10.50	-0.46	19.86
	INF26	IVIG	6	7.50	7.05	1.54	6.30	10.50	5.89	9.12
		SCIG	1	9.90	9.90		9.90	9.90		
	INF27	IVIG	6	8.54	8.39	2.66	5.40	12.90	5.75	11.33
		SCIG	4	9.43	9.60	2.60	6.10	12.40	5.29	13.56
	INF28	IVIG	13	10.25	9.42	3.10	5.80	16.80	8.38	12.12
		SCIG	7	9.63	9.30	1.05	8.50	11.60	8.66	10.60
	INF29	IVIG	7	7.56	6.90	2.48	5.20	12.70	5.26	9.86
		SCIG	1	10.00	10.00		10.00	10.00		
	INF30	IVIG	5	7.11	6.70	0.81	6.30	8.15	6.10	8.12
		SCIG	2	8.06	8.06	1.34	7.11	9.00	-3.95	20.06
	INF31	IVIG	1	10.80	10.80		10.80	10.80		
		SCIG	1	9.10	9.10		9.10	9.10		
	EOP1	IVIG	20	9.01	8.88	2.11	4.64	12.84	8.02	9.99
		SCIG	7	9.16	9.13	2.00	5.76	11.50	7.31	11.00
	WD	SCIG	2	9.70	9.70	1.41	8.70	10.70	-3.01	22.41

INF = Infusion
EOP1 = End of Stage 1
WD = Hospital Visit (other than for Subgam infusion)

Certain data have been excluded from the efficacy analysis. See filenote 001, Section 16.1.9, for details of data exclusions from this table

Program: T0349.TEM, Version: 8.2, Datetime: 07FEB07:09:48

Table 14.2.1.7: Mean serum IgG levels in Stage 1 of study - all patients

Patient	Mean IgG Levels (g/L)										
	Pre-Subgam	Month 1(a)	Month 2	Month 3	Month 4	Month 5	Month 6	Month 7	Month 8	Month 9	Month 10
01		7.0	7.3	6.5		5.6					
05		9.7	9.4	9.0	9.8	9.4	9.8	9.1			
06	12.8	13.1	12.0	11.0	12.4	12.0	12.4				
07	13.3	13.8	13.4	13.8	10.4		14.8	15.3			
08	9.5	8.7	8.4	9.7	9.0	8.7		8.0			
09	10.6	10.8	11.2	11.1	11.3	11.4	11.0				
10	6.5	6.8	5.6	5.9	6.8	6.5	6.5		6.6		
11	6.9	7.5	8.1	8.2	8.2	7.2	8.0				
12	10.4	11.0	9.4	8.8		9.7					
13	6.5	9.4	6.4	7.3	7.3	6.2	6.3	6.3			
17	5.4	7.3	7.8	8.1	8.3	7.9	7.8	8.2			
18	5.1	7.0	5.9	6.3	5.9	6.4	5.8	4.6			
21	13.2	12.6	11.9	9.9	9.7	9.1	10.5	10.8			
22	13.9	13.9	10.4	10.3	9.3	10.0		9.0			
23	11.5	12.5	11.9	11.8	11.7	11.4	12.3	11.1			
24	8.0	9.1	8.0	8.0		8.6		8.0			
25	8.0	7.7	8.5		9.0	9.5	8.9	9.1			
26	5.0	5.3				7.2					
27	9.5	8.1	10.0	8.0		11.0	8.9				
28	6.6	6.3	6.7	7.0	7.2		6.6	7.1			
29	6.3	9.0	7.7	7.4	6.9	6.9		7.2			
30	7.0	11.5	8.2	7.4	7.6		6.6	6.2			
31	8.0	12.1	9.7	8.4	7.4	6.3	6.9	6.4			
33	7.5	9.6	8.0	7.0	7.5	8.8	8.0	8.4			
34	6.8	9.4	8.4	9.8	9.0	9.1	6.9		5.2		
35	8.4	9.1	8.5	9.0	9.1	9.7	8.5				

(a) 1 month = 28 days as defined in Section 6; Month 1 starts at 1st infusion of Subgam (Infusion 4)

Certain data have been excluded from the efficacy analysis. See filenote 001, Section 16.1.9, for details of data exclusions from this table

Program: T0350.TEM, Version: 8.2, Datetime: 07FEB07:09:48

Table 14.2.1.7: Mean serum IgG levels in Stage 1 of study - all patients

	Mean IgG Levels (g/L)										
Patient	Pre-Subgam	Month 1(a)	Month 2	Month 3	Month 4	Month 5	Month 6	Month 7	Month 8	Month 9	Month 10
41	8.4	10.6	8.0		6.2	9.1	8.6	7.2			
42	6.7	9.1		5.2	5.5		4.1		3.6		3.4
43	12.7	14.6		13.1	14.0	14.0	13.9	12.7			
49	6.9	8.2	7.3	6.3	6.9	6.9	7.2	6.9			
53	9.4	9.3	9.0	8.4	9.0	9.3	9.9	9.3			
54	8.6	10.4	10.4	9.9	10.1	11.0	9.5	9.9			
55	12.4	13.2	13.2	13.1	12.5	12.0	12.9	12.3			
56	10.9	12.0	11.0	10.6	11.2	12.7	12.9	12.4			
57	2.8	3.3		3.5	7.0	5.5	6.1	5.8			
58	17.7	20.2	18.1	16.8							
61	9.2	11.9	10.1	8.9	9.9	8.9	10.3	10.1			
65	10.1	13.0	11.2	11.7	11.1	11.4	10.1	12.8			
73	12.5	12.1	11.5	10.8	11.7	11.5	11.2	11.5			
74	9.4	10.0	9.9	10.4	9.8	10.8	10.3	10.5			
75	9.3	9.5	8.9	10.0	9.6	10.0	9.8	10.0			
76	9.4	9.3	8.8	8.4	9.4	8.8	9.3				
77	13.3	14.4	12.7		10.5		13.4				
78	15.3	16.2	15.3		14.1						
81	10.0	12.3	11.9	11.2	12.4	10.4	9.1	8.3			
82	7.1	8.7	6.6	4.5	7.2	6.3		6.3			
83	6.0	8.2	6.5	5.4	7.5	7.7		7.7			
84	8.5	9.9		7.0		6.6	8.2	8.9			
85	9.8	14.0	11.7	9.0		9.3	9.4	9.1			
86	8.5	10.8	7.4		9.2	8.8	9.0	10.0			

(a) 1 month = 28 days as defined in Section 6; Month 1 starts at 1st infusion of Subgam (Infusion 4)

Certain data have been excluded from the efficacy analysis. See filenote 001, Section 16.1.9, for details of data exclusions from this table

Program: T0350.TEM, Version: 8.2, Datetime: 07FEB07:09:48

Table 14.2.1.7: Mean serum IgG levels in Stage 1 of study - all patients

	Mean IgG Levels (g/L)								
	Pre-Subgam	Month 1(a)	Month 2	Month 3	Month 4	Month 5	Month 6	Month 7	Month 8
n	47	49	45	43	41	43	38	35	2
Total Mean of Means	9.2	10.4	9.6	9.0	9.3	9.1	9.4	9.0	5.9
Median	9.2	9.9	9.0	8.9	9.2	9.1	9.2	9.0	5.9
SD	3.0	3.0	2.6	2.6	2.1	2.1	2.3	2.4	1.0
Min	2.8	3.3	5.6	3.5	5.9	5.5	5.8	4.6	5.2
Max	17.7	20.2	18.1	16.8	14.1	14.0	14.8	15.3	6.6
Lower 95% CI	8.4	9.6	8.8	8.2	8.7	8.4	8.6	8.2	-2.9
Upper 95% CI	10.1	11.3	10.4	9.8	10.0	9.7	10.2	9.9	14.7

(a) 1 month = 28 days as defined in Section 6; Month 1 starts at 1st infusion of Subgam (Infusion 4)

Certain data have been excluded from the efficacy analysis. See filenote 001, Section 16.1.9, for details of data exclusions from this table

Program: T0350.TEM, Version: 8.2, Datetime: 07FEB07:09:48

Table 14.2.1.8: Mean serum IgG levels in Stage 1 of study - by diagnosis of PAD

By Diagnosis of PAD		Mean IgG Levels (g/L)								
		Pre-Subgam	Month 1(a)	Month 2	Month 3	Month 4	Month 5	Month 6	Month 7	Month 8
CVID/XLA	n	32	32	29	27	28	29	26	23	2
	Total Mean of Means	8.8	10.0	9.2	8.7	9.0	9.0	8.9	8.7	5.9
	Median	8.5	10.0	8.5	8.8	9.1	9.1	8.7	8.4	5.9
	SD	2.6	2.6	1.9	2.0	1.9	1.9	2.2	2.2	1.0
	Min	2.8	3.3	5.6	3.5	5.9	5.5	5.8	4.6	5.2
	Max	13.9	14.4	12.7	11.8	12.4	12.7	13.4	12.8	6.6
	Lower 95% CI	7.8	9.0	8.5	7.9	8.3	8.2	8.1	7.8	-2.9
	Upper 95% CI	9.7	10.9	10.0	9.5	9.7	9.7	9.8	9.6	14.7
Other	n	15	17	16	16	13	14	12	12	
	Total Mean of Means	10.3	11.3	10.3	9.6	10.1	9.2	10.4	9.7	
	Median	9.5	9.7	9.7	8.9	9.8	9.3	9.8	9.2	
	SD	3.5	3.6	3.4	3.3	2.4	2.4	2.4	2.7	
	Min	5.4	7.0	6.4	4.5	7.2	5.6	6.3	6.3	
	Max	17.7	20.2	18.1	16.8	14.1	14.0	14.8	15.3	
	Lower 95% CI	8.3	9.4	8.5	7.8	8.6	7.8	8.8	8.0	
	Upper 95% CI	12.2	13.1	12.1	11.3	11.5	10.6	12.0	11.4	

(a) 1 month = 28 days as defined in Section 6; Month 1 starts at 1st infusion of Subgam (Infusion 4)

Certain data have been excluded from the efficacy analysis. See filenote 001, Section 16.1.9, for details of data exclusions from this table

Program: T0351.TEM, Version: 8.2, Datetime: 07FEB07:09:48

Figure 14.2.1.9 'Mean of means' serum IgG levels in Stage 1 of study – by diagnosis of PAD

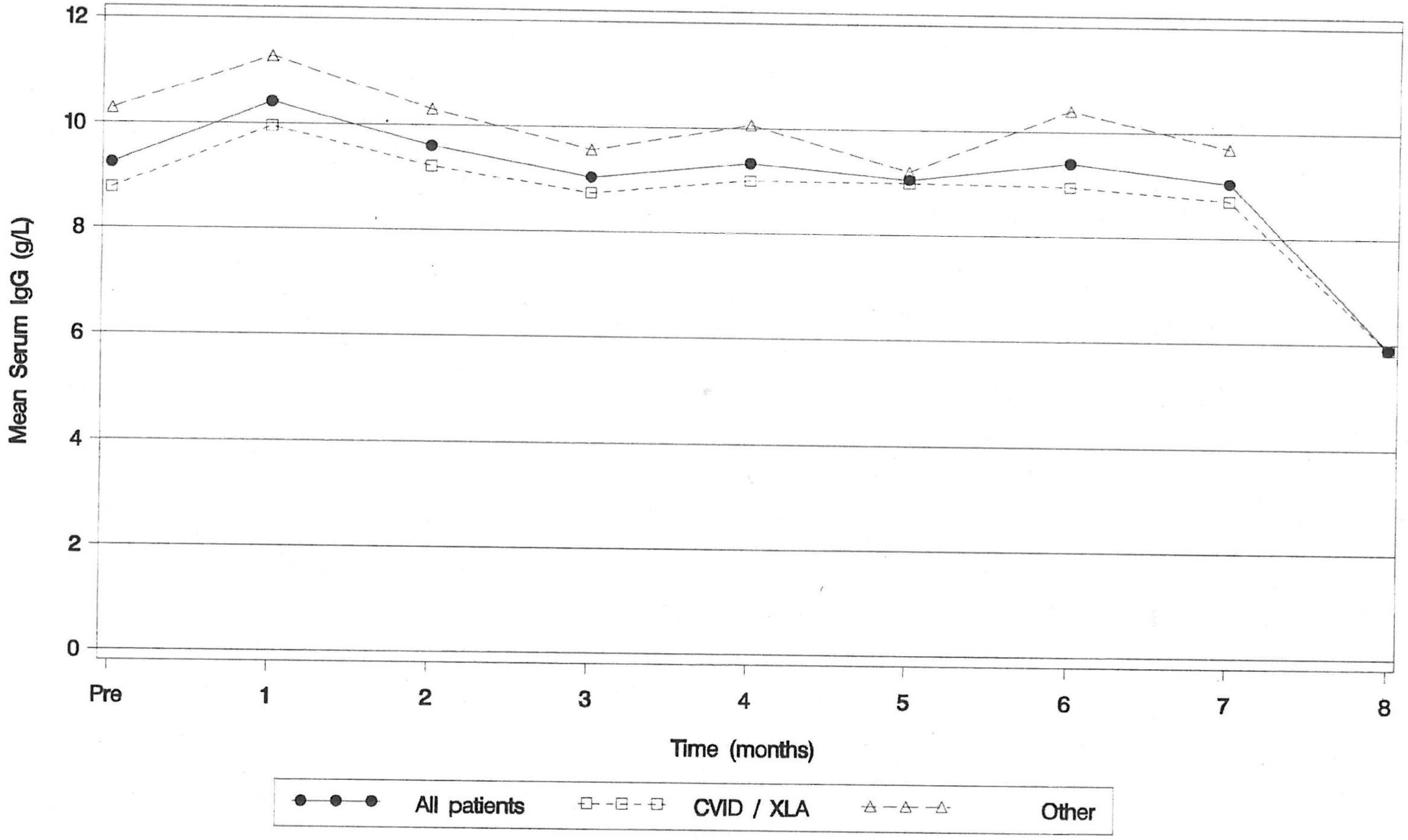

Program: T0339.SAS, Version: 8.2, Datetime: 07FEB2007:09:47

Certain data have been excluded from the efficacy analysis. See filenote 001, Section 16.1.9, for details of data exclusions from this figure

Table 14.2.1.10: Mean serum IgG levels in Stage 1 of study - by age group

By Age group		Mean IgG Levels (g/L)								
		Pre-Subgam	Month 1(a)	Month 2	Month 3	Month 4	Month 5	Month 6	Month 7	Month 8
Adult	n	27	27	26	25	24	23	20	17	2
	Total Mean of Means	9.9	11.0	10.0	9.6	9.5	9.3	9.7	9.5	5.9
	Median	9.4	10.0	9.2	9.7	9.4	9.1	9.7	9.3	5.9
	SD	3.2	3.1	2.8	2.4	2.0	1.9	2.5	2.5	1.0
	Min	5.1	6.8	5.6	5.9	5.9	6.3	5.8	4.6	5.2
	Max	17.7	20.2	18.1	16.8	14.1	14.0	14.8	15.3	6.6
	Lower 95% CI	8.7	9.7	8.8	8.7	8.7	8.4	8.5	8.2	-2.9
	Upper 95% CI	11.2	12.2	11.1	10.6	10.4	10.1	10.9	10.7	14.7
Teenager	n	7	7	6	6	6	6	7	6	
	Total Mean of Means	9.5	11.2	10.0	9.2	9.5	9.9	9.8	9.0	
	Median	8.5	11.5	9.6	9.0	9.4	10.6	8.6	8.0	
	SD	2.4	1.8	2.4	2.7	2.9	2.7	2.8	2.8	
	Min	6.9	8.2	7.3	6.3	6.2	6.6	6.6	6.2	
	Max	12.8	13.2	13.2	13.1	12.5	12.7	12.9	12.4	
	Lower 95% CI	7.3	9.6	7.4	6.4	6.4	7.0	7.2	6.1	
	Upper 95% CI	11.8	12.9	12.5	12.1	12.5	12.8	12.4	11.9	
Child	n	13	15	13	12	11	14	11	12	
	Total Mean of Means	7.6	9.0	8.7	7.7	8.9	8.4	8.6	8.5	
	Median	8.0	8.7	8.5	7.6	9.0	8.8	9.0	8.7	
	SD	2.2	3.0	2.1	2.5	1.8	2.0	1.5	2.0	
	Min	2.8	3.3	6.4	3.5	7.0	5.5	6.1	5.8	
	Max	10.1	14.0	11.9	11.7	12.4	11.4	10.3	12.8	
	Lower 95% CI	6.3	7.4	7.5	6.1	7.6	7.2	7.6	7.2	
	Upper 95% CI	9.0	10.7	10.0	9.2	10.1	9.5	9.6	9.8	

(a) 1 month = 28 days as defined in Section 6; Month 1 starts at 1st infusion of Subgam (Infusion 4)

Certain data have been excluded from the efficacy analysis. See filenote 001, Section 16.1.9, for details of data exclusions from this table

Program: T0352.TEM, Version: 8.2, Datetime: 07FEB07:09:48

Figure 14.2.1.11 'Mean of means' serum IgG levels in Stage 1 of study – by age group

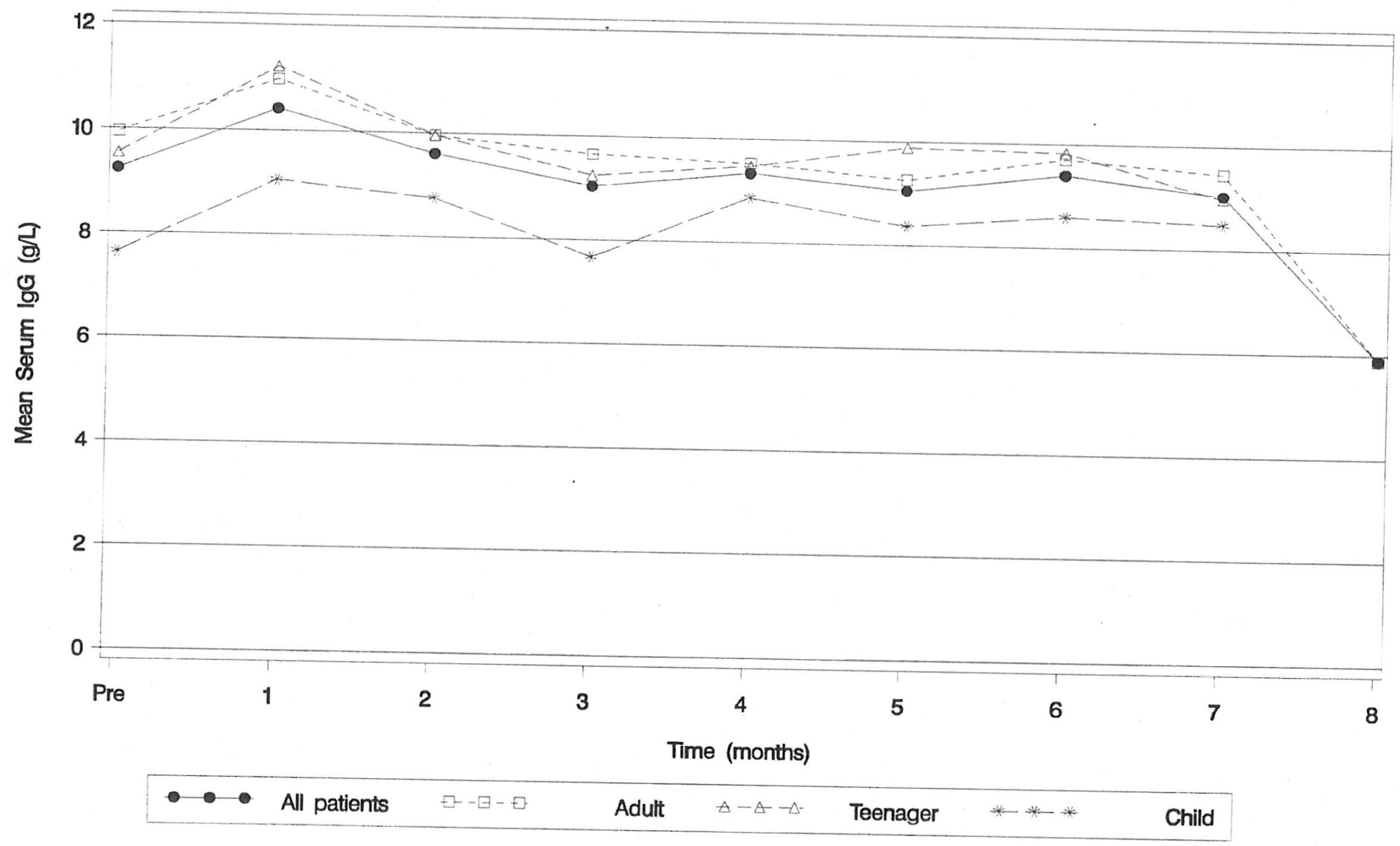

Program: T0340.SAS, Version: 8.2, Datetime: 07FEB2007:09:47

Certain data have been excluded from the efficacy analysis. See filenote 001, Section 16.1.9, for details of data exclusions from this figure

Table 14.2.1.12: Mean serum IgG levels in Stage 1 of study - by prior therapy

By Prior Therapy		Mean IgG Levels (g/L) Pre-Subgam	Month 1(a)	Month 2	Month 3	Month 4	Month 5	Month 6	Month 7	Month 8
IVIG	n	35	35	33	31	30	30	27	26	2
	Total Mean of Means	9.4	11.2	9.8	9.2	9.3	9.0	9.4	9.1	5.9
	Median	8.5	10.8	9.4	8.9	9.1	9.0	9.0	8.6	5.9
	SD	3.0	2.9	2.8	2.7	2.3	2.1	2.6	2.6	1.0
	Min	5.1	6.8	5.6	4.5	5.9	6.2	5.8	4.6	5.2
	Max	17.7	20.2	18.1	16.8	14.1	14.0	14.8	15.3	6.6
	Lower 95% CI	8.4	10.2	8.7	8.2	8.5	8.2	8.4	8.0	-2.9
	Upper 95% CI	10.5	12.2	10.8	10.2	10.1	9.8	10.4	10.1	14.7
SCIG	n	12	14	12	12	11	13	11	9	
	Total Mean of Means	8.7	8.5	9.2	8.6	9.4	9.2	9.4	8.9	
	Median	9.4	9.0	8.9	8.7	9.4	9.4	9.8	9.1	
	SD	2.8	2.6	1.5	2.2	1.6	2.1	1.8	1.8	
	Min	2.8	3.3	6.7	3.5	7.0	5.5	6.1	5.8	
	Max	12.8	13.1	12.0	11.0	12.4	12.0	12.4	11.5	
	Lower 95% CI	6.9	7.1	8.2	7.2	8.4	7.9	8.2	7.6	
	Upper 95% CI	10.5	10.0	10.2	9.9	10.5	10.4	10.6	10.3	

(a) 1 month = 28 days as defined in Section 6; Month 1 starts at 1st infusion of Subgam (Infusion 4)

Certain data have been excluded from the efficacy analysis. See filenote 001, Section 16.1.9, for details of data exclusions from this table

Program: T0353.TEM, Version: 8.2, Datetime: 07FEB07:09:48

Figure 14.2.1.13 'Mean of means' serum IgG levels in Stage 1 of study – by prior therapy

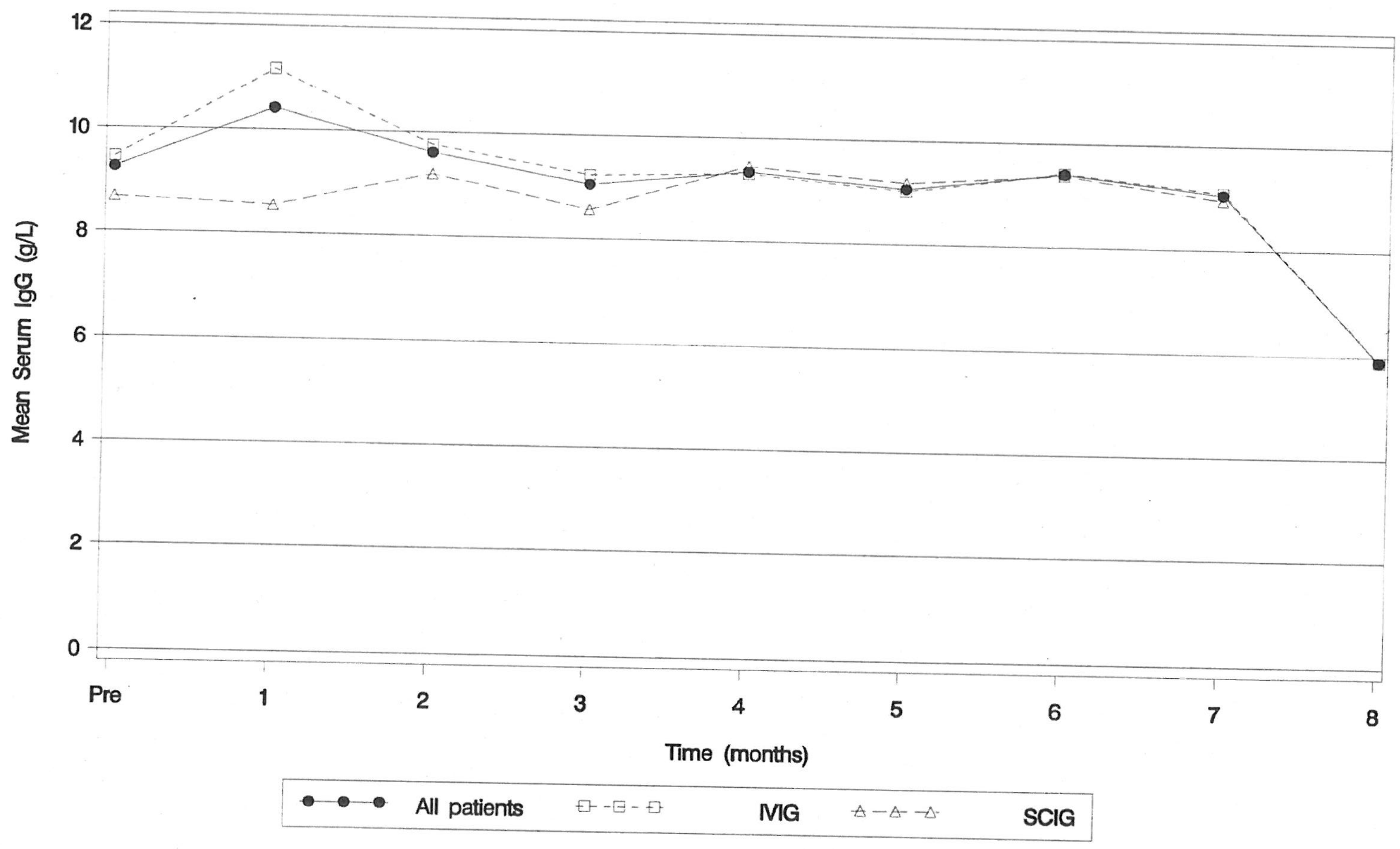

Program: T0102.SAS, Version: 8.2, Datetime: 07FEB2007:09:47

Certain data have been excluded from the efficacy analysis. See filenote 001, Section 16.1.9, for details of data exclusions from this figure

Table 14.2.2.1: Number of patients with serum IgG levels <4g/L and <6g/L across the whole study
Number of patients (patient numbers)

	Stage 1	Stage 2							
	0 to 6 months(a)	>6 to 12 months(b)	>12 to 18 months	>18 to 24 months	>24 to 30 months	>30 to 36 months	>36 to 42 months	>42 to 48 months	>48 to 54 months
**Children (<12y)**									
Number of observations	122	18	16	16	14	16	5	2	0
Number of observations with IgG <4g/L	3	0	0	0	0	0	0	0	0
Percentage of Observations with IgG <4g/L	2.46	0	0	0	0	0	0	0	0
Number of Patients	15	13	11	10	8	10	4	2	0
Number of Patients with IgG <4g/L	1	0	0	0	0	0	0	0	0
Patient ID numbers	57								
Percentage of Patients with IgG <4g/L	6.67	0	0	0	0	0	0	0	0
**Teenagers and adults (>=12y)**									
Number of observations	463	61	61	64	58	46	32	22	8
Number of observations with IgG <6g/L	16	5	5	8	8	2	7	3	1
Percentage of Observations with IgG <6g/L	3.46	8.20	8.20	12.50	13.79	4.35	21.88	13.64	12.50
Number of Patients	34	30	27	27	28	26	21	16	7
Number of Patients with IgG <6g/L	5	3	4	4	4	2	4	3	1
Patient ID numbers	10,18,31, 34,84	18,30,34	11,29,30, 34	11,18,29, 34	10,11,30, 34	11,34	10,11,29, 34	10,11,34	34
Percentage of Patients with IgG <6g/L	14.71	10.00	14.81	14.81	14.29	7.69	19.05	18.75	14.29

(a) This interval lasts approx. 6 months, depending on no. infusions received by patient during Stage 1.
(b) This interval lasts approx. 6 months, from the first infusion in Stage 2 until 12 months after the first infusion of Subgam in Stage 1.

Certain data have been excluded from the efficacy analysis. See filenote 001, Section 16.1.9, for details of data exclusions from this table

Program: T0354.SAS, Version: 8.2, Datetime: 07FEB07:09:48

Table 14.2.2.2: Mean serum IgG levels (g/L) by 6-monthly intervals - all patients

Patient	Stage 1	Stage 2							
	0 to 6 months(a)	>6 to12 months(b)	>12 to18 months	>18 to 24 months	>24 to 30 months	>30 to 36 months	>36 to 42 months	>42 to 48 months	>48 to 54 months
01	6.60	7.20	7.80	.	8.80	8.20	.	.	.
05	9.47	10.00	.	.	.	.	.	.	.
06	12.47	12.60	11.10	11.60	13.55	12.25	13.40	13.85	11.50
07	13.60	14.20	12.60	11.90	13.47	.	.	14.10	.
08	8.63	9.53	11.95	10.30	7.65	.	.	8.00	.
09	11.08	.	.	.	.	.	.	.	.
10	6.34	6.82	7.14	6.96	6.46	6.69	5.97	6.67	8.04
11	7.76	7.06	4.98	5.40	4.91	5.00	4.31	4.31	.
12	10.25	9.03	.	.	.	.	.	.	.
13	7.87	6.10	7.40	8.60	8.25	8.05	7.30	7.00	.
17	7.90	8.25	8.64	8.34	8.07	7.46	8.18	7.70	.
18	6.21	5.68	.	5.38	6.08	.	6.06	6.06	.
21	11.34	10.37	11.70	11.65	11.40	13.00	11.75	.	12.80
22	11.53	9.67	10.20	14.40	11.10	12.50	10.80	10.70	.
23	11.99	12.60	12.45	13.90	12.55	13.10	.	12.70	.
24	8.45	8.00	8.40	8.90	9.60	9.45	.	11.40	.
25	8.49	.	8.46	9.77	.	.	6.90	6.95	.
26	5.80	5.83	.	.	.	.	.	.	.
27	8.78	10.69	.	12.17	.	10.03	.	.	.
28	6.75	7.85	6.93	.	.	6.22	5.83	.	.
29	7.82	.	6.70	5.30	.	6.55	5.30	8.00	7.10
30	8.58	5.75	4.90	.	4.90	7.40	6.90	6.70	.
31	8.42	.	.	.	.	.	.	.	.

(a) This interval lasts approx. 6 months, depending on no. infusions received by patient during Stage 1.
(b) This interval lasts approx. 6 months, from the first infusion in Stage 2 until 12 months after the first infusion of Subgam in Stage 1.

Certain data have been excluded from the efficacy analysis. See filenote 001, Section 16.1.9, for details of data exclusions from this table

Program: T0355.TEM, Version: 8.2, Datetime: 07FEB07:09:48

Table 14.2.2.2: Mean serum IgG levels (g/L) by 6-monthly intervals - all patients

Patient	Stage 1	Stage 2							
	0 to 6 months(a)	>6 to12 months(b)	>12 to18 months	>18 to 24 months	>24 to 30 months	>30 to 36 months	>36 to 42 months	>42 to 48 months	>48 to 54 months
33	8.54	7.40	8.20	8.60	7.90	7.80	11.90	.	.
34	8.62	5.40	6.35	4.15	5.35	5.05	4.90	5.80	5.40
35	8.88	8.55	7.80	7.90	7.40	8.85	10.10	9.00	6.00
41	9.21	7.68	8.50	8.68	8.45	9.83	8.86	7.46	7.62
42	6.72	3.23	7.01	5.76	5.18	.	3.36	3.68	.
43	14.09	12.80	.	.	.	.	.	.	.
49	7.48	7.30	7.10	6.95	7.55	7.60	8.50	8.20	.
53	9.29	9.30	10.48	10.88	10.80	11.38	.	.	.
54	10.29	10.73	10.15	9.54	9.17	8.30	7.30	.	.
55	12.98	13.90	14.08	12.68	12.20	11.30	.	.	.
56	11.76	12.55	13.07	13.00	12.40	12.30	13.60	.	.
57	4.93	5.47	6.28	7.46	6.61	5.59	6.23	.	.
58	19.40	18.47	17.10	16.60	17.15	17.35	16.20	.	.
61	10.39	12.20	9.76	9.92	.	.	.	.	.
65	11.79	.	.	.	.	.	.	.	.
73	11.63	11.15	10.68	12.58	11.53	11.63	13.55	.	.
74	10.13	.	10.35	10.90	10.80	10.00	11.60	.	.
75	9.54	10.23	10.60	.	10.08	11.20	.	.	.
76	9.06	9.80	.	.	.	.	.	.	.
77	13.40	12.83	12.20	11.65	10.20	12.55	13.50	.	.
78	15.51	14.60	.	14.90	17.30	14.90	12.50	.	.
81	11.33	6.93	7.91	6.93	7.50	7.39	.	.	.
82	7.28	7.40	6.50	6.40	6.35	7.59	.	.	.

(a) This interval lasts approx. 6 months, depending on no. infusions received by patient during Stage 1.
(b) This interval lasts approx. 6 months, from the first infusion in Stage 2 until 12 months after the first infusion of Subgam in Stage 1.

Certain data have been excluded from the efficacy analysis. See filenote 001, Section 16.1.9, for details of data exclusions from this table

Program: T0355.TEM, Version: 8.2, Datetime: 07FEB07:09:48

Table 14.2.2.2: Mean serum IgG levels (g/L) by 6-monthly intervals - all patients

Patient	Stage 1	Stage 2							
	0 to 6 months(a)	>6 to12 months(b)	>12 to18 months	>18 to 24 months	>24 to 30 months	>30 to 36 months	>36 to 42 months	>42 to 48 months	>48 to 54 months
83	7.49	6.60	7.66	7.53	9.64	7.11	.	.	.
84	8.22	9.47	8.90	9.34	11.15	8.48	.	.	.
85	10.68	10.10	8.68	9.86	8.46	9.84	.	.	.
86	9.57	7.49	9.63	10.41	11.30	10.70	.	.	.

(a) This interval lasts approx. 6 months, depending on no. infusions received by patient during Stage 1.
(b) This interval lasts approx. 6 months, from the first infusion in Stage 2 until 12 months after the first infusion of Subgam in Stage 1.

Certain data have been excluded from the efficacy analysis. See filenote 001, Section 16.1.9, for details of data exclusions from this table

Program: T0355.TEM, Version: 8.2, Datetime: 07FEB07:09:48

Table 14.2.2.2: Mean serum IgG levels (g/L) by 6-monthly intervals - all patients

	Stage 1	Stage 2							
	0 to 6 months(a)	>6 to12 months(b)	>12 to18 months	>18 to 24 months	>24 to 30 months	>30 to 36 months	>36 to 42 months	>42 to 48 months	>48 to 54 months
n	49	43	38	37	36	36	25	18	7
Total Mean of Means	9.75	9.43	9.30	9.77	9.61	9.52	9.26	8.59	8.35
Median	9.21	9.30	8.66	9.77	9.38	9.15	8.50	7.85	7.62
SD	2.69	2.94	2.62	2.93	3.02	2.85	3.38	2.82	2.77
Min	4.93	5.40	4.90	4.15	4.90	5.00	4.31	4.31	5.40
Max	19.40	18.47	17.10	16.60	17.30	17.35	16.20	14.10	12.80
Lower 95% CI	8.97	8.53	8.44	8.79	8.59	8.55	7.86	7.18	5.79
Upper 95% CI	10.52	10.33	10.16	10.74	10.64	10.48	10.65	9.99	10.92

(a) This interval lasts approx. 6 months, depending on no. infusions received by patient during Stage 1.
(b) This interval lasts approx. 6 months, from the first infusion in Stage 2 until 12 months after the first infusion of Subgam in Stage 1.

Certain data have been excluded from the efficacy analysis. See filenote 001, Section 16.1.9, for details of data exclusions from this table

Program: T0355.TEM, Version: 8.2, Datetime: 07FEB07:09:48

Table 14.2.2.3: Mean serum IgG levels (g/L) by 6-monthly intervals - completers(c)

	Stage 1	Stage 2								
	0 to 6 months(a)	>6 to12 months(b)	>12 to18 months	>18 to 24 months	>24 to 30 months	>30 to 36 months	>36 to 42 months	>42 to 48 months	>48 to 54 months	% change in mean serum IgG(d)
n	32	30	32	30	30	29	19	14	7	29
Total Mean of Means	10.01	9.63	9.54	9.61	9.55	9.51	9.64	8.53	8.35	95.93
Median	9.41	9.39	9.26	9.70	9.40	8.85	8.86	7.85	7.62	98.68
SD	2.59	3.04	2.74	2.81	2.85	2.79	3.52	2.96	2.77	15.17
Min	6.34	5.40	4.90	4.15	4.90	5.00	4.31	4.31	5.40	58.56
Max	19.40	18.47	17.10	16.60	17.15	17.35	16.20	14.10	12.80	122.50
Lower 95% CI	9.08	8.50	8.55	8.57	8.49	8.45	7.94	6.82	5.79	90.16
Upper 95% CI	10.95	10.77	10.53	10.66	10.62	10.57	11.33	10.24	10.92	101.69

(a) This interval lasts approx. 6 months, depending on no. infusions received by patient during Stage 1.
(b) This interval lasts approx. 6 months, from the first infusion in Stage 2 until 12 months after the first infusion of Subgam in Stage 1.
(c) Completers are defined in Section 5 of the statistical analysis plan.
(d) Mean IgG at >30-36 months interval as a % of mean IgG in Stage 1

Certain data have been excluded from the efficacy analysis. See filenote 001, Section 16.1.9, for details of data exclusions from this table

Program: T0356.TEM, Version: 8.2, Datetime: 07FEB07:09:48

Figure 14.2.2.4 'Mean of means' serum IgG levels (g/L) in 6–monthly intervals – all patients and completers(c)

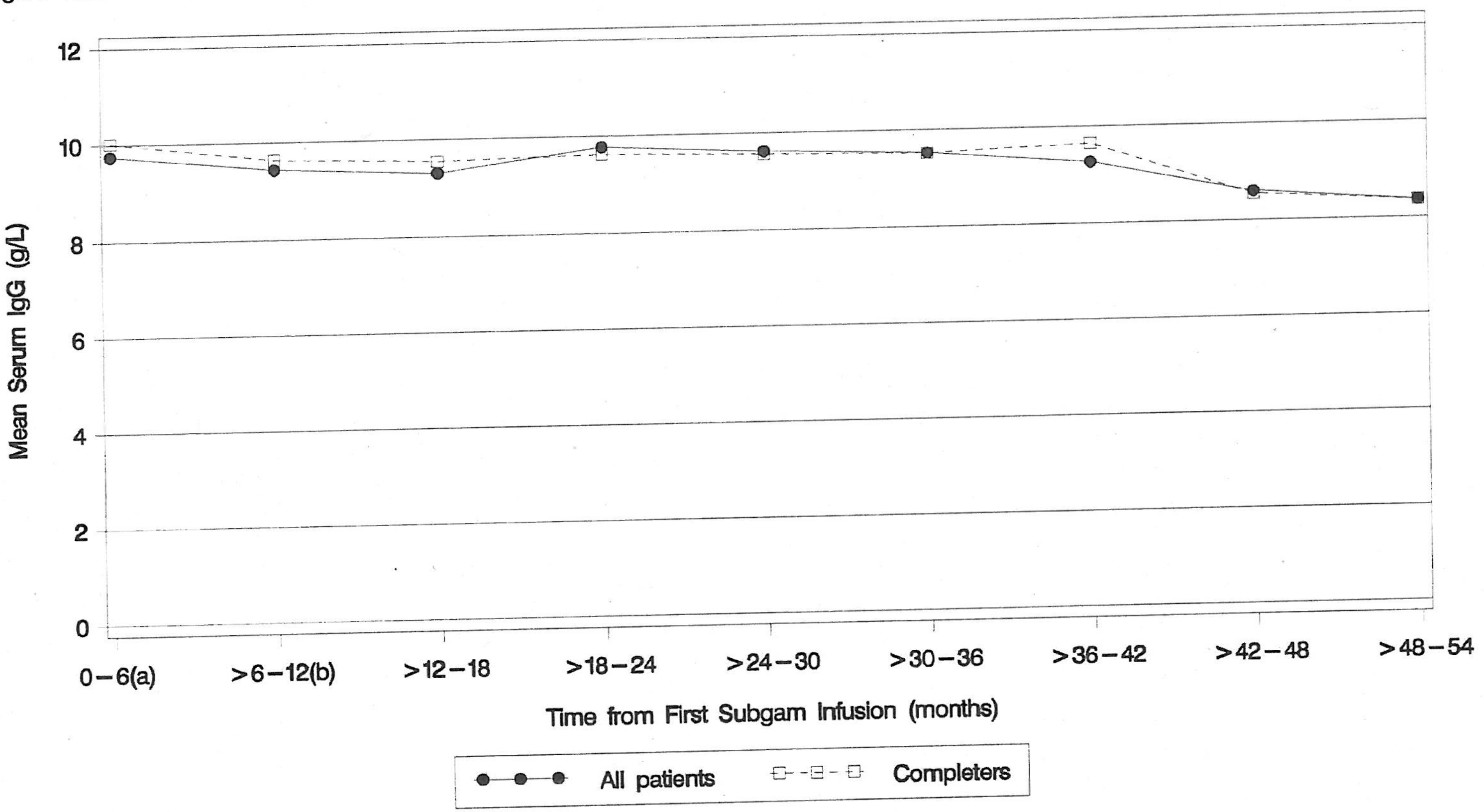

(a) This interval lasts approximately 6 months, depending on the number of infusions received by the patient during Stage 1
(b) This interval lasts approximately 6 months, from the first infusion in Stage 2 until 12 months after the first infusion of Subgam in Stage 1
(c) Completers are defined in Section 5

Certain data have been excluded from the efficacy analysis. See filenote 001, Section 16.1.9, for details of data exclusions from this figure

Program: T0341.SAS, Version: 8.2, Datetime: 07FEB2007:09:47

Table 14.2.2.5: Mean serum IgG levels (g/L) by 6-monthly intervals - by diagnosis of PAD, all patients

Diagnosis of PAD		Stage 1	Stage 2							
		0 to 6 months(a)	>6 to12 months(b)	>12 to18 months	>18 to 24 months	>24 to 30 months	>30 to 36 months	>36 to 42 months	>42 to 48 months	>48 to 54 months
CVID/XLA	n	32	27	26	25	24	24	21	15	7
	Total Mean of Means	9.24	8.65	8.92	9.27	8.98	9.09	8.92	8.39	8.35
	Median	8.76	8.00	8.48	9.34	8.81	8.66	8.50	8.00	7.62
	SD	2.10	2.34	2.32	2.81	2.59	2.66	3.23	2.69	2.77
	Min	4.93	5.40	4.90	4.15	4.90	5.00	4.31	4.31	5.40
	Max	13.40	12.83	13.07	14.40	13.55	13.10	13.60	13.85	12.80
	Lower 95% CI	8.48	7.72	7.99	8.11	7.89	7.97	7.45	6.90	5.79
	Upper 95% CI	10.00	9.58	9.86	10.42	10.07	10.21	10.39	9.87	10.92
Other	n	17	16	12	12	12	12	4	3	0
	Total Mean of Means	10.70	10.75	10.11	10.81	10.88	10.37	11.05	9.60	
	Median	9.54	10.17	9.22	10.40	9.86	9.94	10.34	7.70	
	SD	3.42	3.41	3.12	3.01	3.53	3.15	4.12	3.91	
	Min	6.60	6.10	6.50	6.40	6.35	7.11	7.30	7.00	
	Max	19.40	18.47	17.10	16.60	17.30	17.35	16.20	14.10	
	Lower 95% CI	8.94	8.93	8.12	8.90	8.64	8.37	4.49	-0.12	
	Upper 95% CI	12.46	12.57	12.09	12.73	13.12	12.37	17.60	19.32	

(a) This interval lasts approx. 6 months, depending on no. infusions received by patient during Stage 1.
(b) This interval lasts approx. 6 months, from the first infusion in Stage 2 until 12 months after the first infusion of Subgam in Stage 1.

Certain data have been excluded from the efficacy analysis. See filenote 001, Section 16.1.9, for details of data exclusions from this table

Program: T0357.TEM, Version: 8.2, Datetime: 07FEB07:09:48

Figure 14.2.2.6 'Mean of means' serum IgG levels (g/L) in 6−monthly intervals − by diagnosis of PAD, all patients

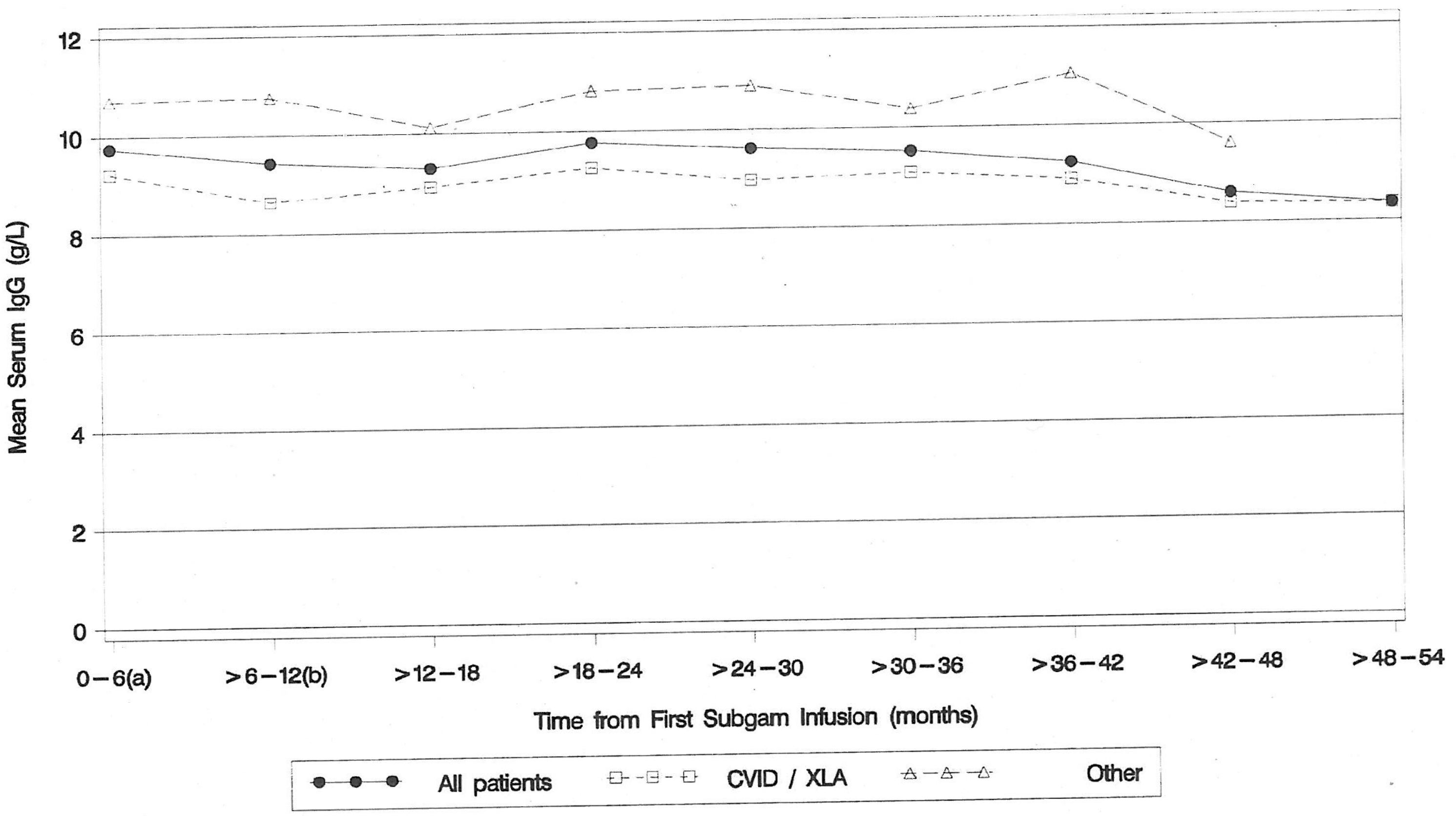

(a) This interval lasts approximately 6 months, depending on the number of infusions received by the patient during Stage 1
(b) This interval lasts approximately 6 months, from the first infusion in Stage 2 until 12 months after the first infusion of Subgam in Stage 1

Certain data have been excluded from the efficacy analysis. See filenote 001, Section 16.1.9, for details of data exclusions from this figure

Program: T0342.SAS, Version: 8.2, Datetime: 07FEB2007:09:47

Table 14.2.2.7: Mean serum IgG levels (g/L) by 6-monthly intervals - by age group, all patients

Age group		Stage 1	Stage 2							
		0 to 6 months(a)	>6 to12 months(b)	>12 to18 months	>18 to 24 months	>24 to 30 months	>30 to 36 months	>36 to 42 months	>42 to 48 months	>48 to 54 months
Adult	n	27	23	20	21	21	19	16	12	5
	Total Mean of Means	10.36	10.11	9.93	10.01	9.95	10.14	9.62	8.70	7.87
	Median	9.54	9.67	10.28	10.30	10.08	10.00	10.45	8.00	7.10
	SD	2.94	3.10	2.77	3.45	3.37	3.36	3.65	2.97	2.94
	Min	6.21	5.40	4.98	4.15	4.91	5.00	4.31	4.31	5.40
	Max	19.40	18.47	17.10	16.60	17.30	17.35	16.20	14.10	12.80
	Lower 95% CI	9.20	8.77	8.64	8.43	8.42	8.53	7.67	6.81	4.22
	Upper 95% CI	11.52	11.45	11.23	11.58	11.48	11.76	11.57	10.59	11.52
Teenager	n	7	7	7	6	7	7	5	4	2
	Total Mean of Means	10.10	9.89	9.66	10.37	10.03	9.88	10.25	9.05	9.56
	Median	9.21	9.47	8.90	10.47	11.15	9.83	8.86	7.83	9.56
	SD	2.24	3.15	3.27	2.42	3.13	2.11	3.06	3.26	2.74
	Min	7.48	5.75	4.90	6.95	4.90	7.40	6.90	6.70	7.62
	Max	12.98	13.90	14.08	13.00	13.55	12.30	13.60	13.85	11.50
	Lower 95% CI	8.03	6.98	6.63	7.83	7.13	7.92	6.46	3.87	-15.09
	Upper 95% CI	12.17	12.80	12.69	12.92	12.93	11.83	14.05	14.23	34.21
Child	n	15	13	11	10	8	10	4	2	0
	Total Mean of Means	8.48	7.99	7.91	8.90	8.36	8.07	6.56	6.98	
	Median	8.49	7.40	7.80	9.19	8.36	7.82	6.56	6.98	
	SD	2.05	2.09	1.15	1.82	1.62	1.67	0.66	0.04	
	Min	4.93	5.47	6.28	6.40	6.35	5.59	5.83	6.95	

(a) This interval lasts approx. 6 months, depending on no. infusions received by patient during Stage 1.
(b) This interval lasts approx. 6 months, from the first infusion in Stage 2 until 12 months after the first infusion of Subgam in Stage 1.

Certain data have been excluded from the efficacy analysis. See filenote 001, Section 16.1.9, for details of data exclusions from this table

Program: T0358.TEM, Version: 8.2, Datetime: 07FEB07:09:48

Table 14.2.2.7: Mean serum IgG levels (g/L) by 6-monthly intervals - by age group, all patients

Age group		Stage 1	Stage 2							
		0 to 6 months(a)	>6 to12 months(b)	>12 to18 months	>18 to 24 months	>24 to 30 months	>30 to 36 months	>36 to 42 months	>42 to 48 months	>48 to 54 months
Child	Max	11.79	12.20	9.76	12.17	11.30	10.70	7.30	7.00	
	Lower 95% CI	7.34	6.73	7.14	7.60	7.01	6.88	5.51	6.66	
	Upper 95% CI	9.62	9.25	8.68	10.21	9.72	9.27	7.61	7.29	

(a) This interval lasts approx. 6 months, depending on no. infusions received by patient during Stage 1.
(b) This interval lasts approx. 6 months, from the first infusion in Stage 2 until 12 months after the first infusion of Subgam in Stage 1.

Certain data have been excluded from the efficacy analysis. See filenote 001, Section 16.1.9, for details of data exclusions from this table

Program: T0358.TEM, Version: 8.2, Datetime: 07FEB07:09:48

Figure 14.2.2.8 'Mean of means' serum IgG levels (g/L) in 6–monthly intervals – by age group, all patients

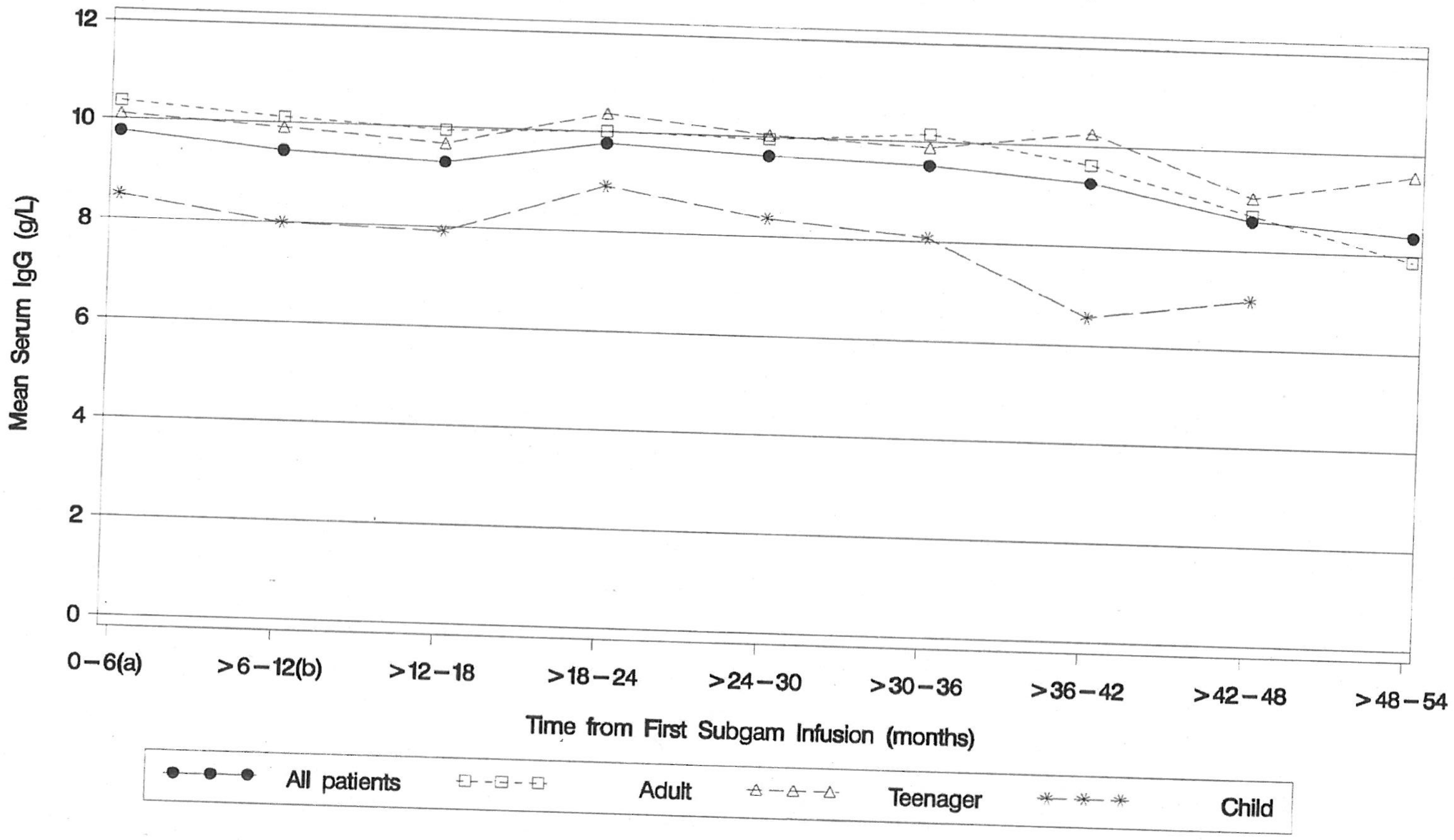

(a) This interval lasts approximately 6 months, depending on the number of infusions received by the patient during Stage 1
(b) This interval lasts approximately 6 months, from the first infusion in Stage 2 until 12 months after the first infusion of Subgam in Stage 1

Certain data have been excluded from the efficacy analysis. See filenote 001, Section 16.1.9, for details of data exclusions from this figure

Program: T0359.SAS, Version: 8.2, Datetime: 07FEB2007:09:47

Table 14.2.2.9: Mean serum IgG levels (g/L) by 6-monthly intervals - by prior therapy, all patients

Prior therapy		Stage 1	Stage 2							
		0 to 6 months(a)	>6 to12 months(b)	>12 to18 months	>18 to 24 months	>24 to 30 months	>30 to 36 months	>36 to 42 months	>42 to 48 months	>48 to 54 months
IVIG	n	35	31	28	29	28	27	19	15	6
	Total Mean of Means	10.17	9.55	9.24	9.51	9.51	9.49	9.15	8.39	7.83
	Median	9.57	8.55	8.57	8.90	8.81	8.48	8.50	7.70	7.36
	SD	2.81	3.21	2.85	3.17	3.24	3.02	3.39	2.73	2.63
	Min	6.21	5.40	4.90	4.15	4.90	5.00	4.31	4.31	5.40
	Max	19.40	18.47	17.10	16.60	17.30	17.35	16.20	14.10	12.80
	Lower 95% CI	9.21	8.37	8.13	8.30	8.25	8.29	7.52	6.88	5.07
	Upper 95% CI	11.14	10.72	10.34	10.71	10.77	10.68	10.79	9.90	10.59
SCIG	n	14	12	10	8	8	9	6	3	1
	Total Mean of Means	8.68	9.14	9.46	10.71	9.98	9.61	9.58	9.60	11.50
	Median	8.92	9.66	10.41	10.89	10.44	10.03	9.25	8.00	11.50
	SD	2.10	2.15	1.94	1.61	2.23	2.41	3.66	3.72	
	Min	4.93	5.47	6.28	7.46	6.61	5.59	5.83	6.95	11.50
	Max	12.47	12.60	11.95	12.58	13.55	12.25	13.55	13.85	11.50
	Lower 95% CI	7.47	7.77	8.08	9.36	8.12	7.75	5.74	0.36	
	Upper 95% CI	9.90	10.50	10.85	12.05	11.84	11.47	13.42	18.84	

(a) This interval lasts approx. 6 months, depending on no. infusions received by patient during Stage 1.
(b) This interval lasts approx. 6 months, from the first infusion in Stage 2 until 12 months after the first infusion of Subgam in Stage 1.

Certain data have been excluded from the efficacy analysis. See filenote 001, Section 16.1.9, for details of data exclusions from this table

Program: T0360.TEM, Version: 8.2, Datetime: 07FEB07:09:48

Figure 14.2.2.10 'Mean of means' serum IgG levels (g/L) in 6-monthly intervals – by prior therapy, all patients

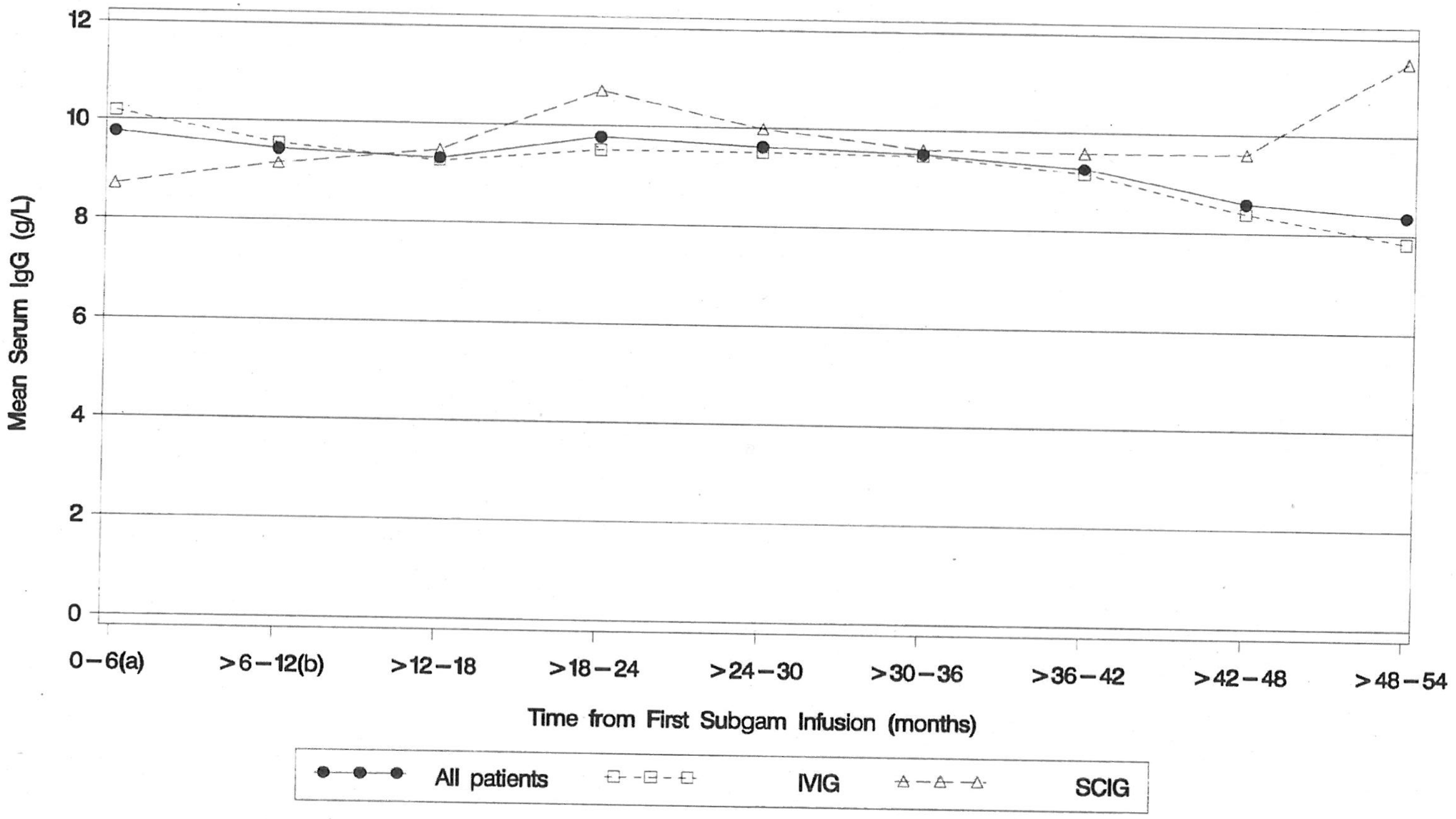

(a) This interval lasts approximately 6 months, depending on the number of infusions received by the patient during Stage 1
(b) This interval lasts approximately 6 months, from the first infusion in Stage 2 until 12 months after the first infusion of Subgam in Stage 1

Certain data have been excluded from the efficacy analysis. See filenote 001, Section 16.1.9, for details of data exclusions from this figure

Program: T0361.SAS, Version: 8.2, Datetime: 07FEB2007:09:47

Table 14.2.3.1: Daily inter-infusion total serum IgG values (g/L)

	Patient(a)	Pre-infusion	Number of days post-infusion 1	2	3	4	5	6	7
1st Subgam Infusion	17 (1)	7.41	7.92	7.47	7.71			7.14	
	21 (1)	13.80	14.50	13.70	13.80	13.50			13.40
	22 (1)	17.80	17.50	16.90	17.00		15.70		14.40
	23 (1)	13.70	13.50			12.40	12.70	12.30	
	24 (1)	9.90	10.50	10.90	10.70	9.60			9.20
	29 (1)	10.60	10.40	10.20			9.60	10.70	9.20
	77 (1)	16.30	17.00					15.20	15.50
	78 (1)	18.90	18.90			17.70	17.10	15.80	16.40
	n	8	8	5	4	4	4	5	6
	Mean	13.55	13.78	11.83	12.30	13.30	13.78	12.23	13.02
	Median	13.75	14.00	10.90	12.25	12.95	14.20	12.30	13.90
	SD	4.04	3.92	3.60	4.00	3.36	3.33	3.53	3.12
	Min	7.41	7.92	7.47	7.71	9.60	9.60	7.14	9.20
	Max	18.90	18.90	16.90	17.00	17.70	17.10	15.80	16.40
	Lower 95% CI	10.18	10.50	7.37	5.94	7.95	8.47	7.85	9.74
	Upper 95% CI	16.93	17.05	16.30	18.67	18.65	19.08	16.61	16.30

(a) Numbers in parenthesis are the numbers of Subgam infusions prior to the start of the assessment.

Program: T0362.SAS, Version: 8.2, Datetime: 07FEB07:09:48

Table 14.2.3.1: Daily inter-infusion total serum IgG values (g/L)

	Patient(a)	Pre-infusion	Number of days post-infusion						
			1	2	3	4	5	6	7
After approximately 3 months on Subgam	17 (13)	7.60	8.90	7.51	8.00			7.97	
	21 (15)	9.70	10.50	11.10	11.00	10.50			
	22 (16)	9.30					11.10	10.10	10.00
	23 (13)		12.50			12.10	12.90	11.50	
	29 (15)						7.50	7.50	6.60
	77 (13)			11.80	11.60	11.90	11.70		
	78 (15)	13.60	13.80			14.20	14.50	13.60	
	n	4	4	3	3	4	5	5	2
	Mean	10.05	11.43	10.14	10.20	12.18	11.54	10.13	8.30
	Median	9.50	11.50	11.10	11.00	12.00	11.70	10.10	8.30
	SD	2.54	2.16	2.30	1.93	1.53	2.61	2.52	2.40
	Min	7.60	8.90	7.51	8.00	10.50	7.50	7.50	6.60
	Max	13.60	13.80	11.80	11.60	14.20	14.50	13.60	10.00
	Lower 95% CI	6.02	7.98	4.42	5.41	9.75	8.30	7.00	-13.30
	Upper 95% CI	14.08	14.87	15.85	14.99	14.60	14.78	13.27	29.90

(a) Numbers in parenthesis are the numbers of Subgam infusions prior to the start of the assessment.

Program: T0362.SAS, Version: 8.2, Datetime: 07FEB07:09:48

Table 14.2.4.1: Mean (range) Subgam dose (mg/kg) received in Stage 1 of the study - all patients

	Stage 1							
Patient	Month 1(a)	Month 2	Month 3	Month 4	Month 5	Month 6	Month 7	Month 8
01	95.3<br>(94.9 - 95.6)	94.4<br>(94.2 - 94.7)	93.7<br>(93.5 - 94.0)	93.0<br>(92.8 - 93.3)	92.3<br>(92.1 - 92.6)	91.6<br>(91.4 - 91.9)	91.1<br>(90.9 - 91.2)	
05	97.6<br>(92.9 - 100.8)	99.0<br>(98.7 - 99.4)	101.8<br>(101.5 - 102.1)	102.4<br>(100.8 - 106.5)	105.9<br>(105.6 - 106.3)	105.0<br>(104.7 - 105.3)	104.4<br>(104.2 - 104.5)	
06	99.7<br>(99.4 - 100.0)	99.1<br>(98.9 - 99.3)	98.6<br>(98.3 - 98.8)	98.0<br>(97.8 - 98.2)	97.5<br>(97.3 - 97.7)	97.0<br>(96.8 - 97.2)	96.5<br>(96.4 - 96.7)	
07	99.7<br>(99.6 - 99.8)	99.4<br>(99.4 - 99.5)	99.2<br>(99.2 - 99.3)	99.0<br>(98.9 - 99.1)	98.8<br>(98.8 - 98.9)	98.7<br>(98.6 - 98.7)	98.4<br>(98.4 - 98.5)	
08	101.5<br>(101.4 - 101.5)	101.7<br>(101.6 - 101.7)	101.8<br>(101.8 - 101.9)	102.0<br>(101.9 - 102.1)	102.2<br>(102.1 - 102.2)	102.3<br>(102.3 - 102.4)	116.9<br>(102.5 - 131.3)	
09	99.9<br>(99.8 - 100.0)	100.1<br>(100.0 - 100.2)	100.3<br>(100.2 - 100.3)	100.4<br>(100.4 - 100.5)	100.6<br>(100.5 - 100.6)	100.8<br>(100.7 - 100.8)		
10	100.1<br>(100.1 - 100.1)	100.0<br>(100.0 - 100.1)	100.0<br>(100.0 - 100.0)	100.0<br>(100.0 - 100.0)	100.0<br>(100.0 - 100.0)	100.0<br>(100.0 - 100.0)	100.0<br>(100.0 - 100.0)	100.0<br>(100.0 - 100.0)
11	96.6<br>(96.0 - 97.2)	95.4<br>(95.2 - 95.7)	94.3<br>(93.8 - 94.7)	93.1<br>(92.7 - 93.5)	91.8<br>(91.3 - 92.4)	93.9<br>(91.0 - 94.8)	94.1<br>(94.1 - 94.1)	
12	100.3<br>(100.3 - 100.3)	93.2<br>(93.2 - 93.2)	92.7<br>(91.1 - 93.2)	93.2<br>(93.2 - 93.2)	93.2<br>(93.2 - 93.2)	93.2<br>(93.2 - 93.2)		

(a) 1 month = 28 days as defined in Section 6 of the statistical analysis plan; Month 1 starts at 1st infusion of Subgam (Infusion 4)

Certain data have been excluded from the efficacy analysis. See filenote 001, Section 16.1.9, for details of data exclusions from this table

Program: T0127.TEM, Version: 8.2, Datetime: 07FEB07:09:48

Table 14.2.4.1: Mean (range) Subgam dose (mg/kg) received in Stage 1 of the study - all patients

Patient	Stage 1							
	Month 1(a)	Month 2	Month 3	Month 4	Month 5	Month 6	Month 7	Month 8
13	98.6<br>(97.6 - 99.7)	96.3<br>(95.5 - 97.1)	94.6<br>(94.1 - 95.0)	92.6<br>(91.7 - 93.5)	90.8<br>(90.3 - 91.3)	89.0<br>(88.2 - 89.8)	87.4<br>(86.9 - 87.8)	
17	101.0<br>(100.9 - 101.1)	101.2<br>(101.2 - 101.3)	101.4<br>(101.4 - 101.5)	101.7<br>(101.6 - 101.7)	101.9<br>(101.8 - 102.0)	102.1<br>(102.0 - 102.1)	102.2<br>(102.2 - 102.3)	
18	101.8<br>(101.5 - 102.1)	102.4<br>(102.3 - 102.6)	103.1<br>(102.8 - 103.4)	103.9<br>(103.6 - 104.1)	104.5<br>(104.3 - 104.7)	105.2<br>(104.9 - 105.4)	105.8<br>(105.6 - 106.0)	
21	100.8<br>(100.6 - 101.0)	101.3<br>(101.1 - 101.4)	101.7<br>(101.5 - 101.8)	102.1<br>(101.9 - 102.2)	102.5<br>(102.3 - 102.6)	126.4<br>(102.7 - 134.4)	134.7<br>(134.6 - 134.8)	
22	124.3<br>(100.0 - 130.4)	130.2<br>(130.1 - 130.2)	130.0<br>(129.9 - 130.1)	129.9<br>(129.8 - 129.9)	129.7<br>(129.6 - 129.8)	129.5<br>(129.5 - 129.6)	129.4<br>(129.4 - 129.4)	
23	135.4<br>(104.5 - 143.6)	144.2<br>(143.8 - 144.6)	145.3<br>(144.9 - 145.7)	146.4<br>(146.0 - 146.8)	147.6<br>(147.1 - 148.0)	148.7<br>(148.3 - 149.1)	149.7<br>(149.4 - 150.0)	
24	104.8<br>(84.0 - 110.4)	110.9<br>(110.6 - 111.3)	111.8<br>(111.5 - 112.0)	112.5<br>(112.2 - 112.9)	113.5<br>(113.1 - 113.8)	114.4<br>(114.0 - 114.8)	115.3<br>(115.0 - 115.7)	
25	98.0<br>(97.2 - 99.2)	96.5<br>(96.2 - 96.9)	95.3<br>(94.7 - 95.9)	94.0<br>(93.7 - 94.3)	93.0<br>(92.5 - 93.4)	91.8<br>(91.4 - 92.2)	90.5<br>(90.2 - 90.8)	90.1<br>(90.1 - 90.1)
26	100.1<br>(99.3 - 100.8)	98.4<br>(97.9 - 99.0)	97.0<br>(96.5 - 97.6)	96.2<br>(96.2 - 96.2)	94.0<br>(93.9 - 94.2)	93.0<br>(92.6 - 93.5)	91.9<br>(91.6 - 92.3)	

(a) 1 month = 28 days as defined in Section 6 of the statistical analysis plan; Month 1 starts at 1st infusion of Subgam (Infusion 4)

Certain data have been excluded from the efficacy analysis. See filenote 001, Section 16.1.9, for details of data exclusions from this table

Program: T0127.TEM, Version: 8.2, Datetime: 07FEB07:09:48

Table 14.2.4.1: Mean (range) Subgam dose (mg/kg) received in Stage 1 of the study - all patients

	Stage 1							
Patient	Month 1(a)	Month 2	Month 3	Month 4	Month 5	Month 6	Month 7	Month 8
27	103.5<br>(102.8 - 105.3)	103.6<br>(101.6 - 104.8)	100.3<br>(99.7 - 101.0)	127.3<br>(99.2 - 137.2)	134.8<br>(134.2 - 135.4)	132.8<br>(132.0 - 133.6)	130.6<br>(129.8 - 131.4)	
28	102.1<br>(102.1 - 102.2)	101.9<br>(101.8 - 102.0)	101.7<br>(101.6 - 101.8)	101.5<br>(101.4 - 101.6)	101.3<br>(101.2 - 101.4)	101.1<br>(101.0 - 101.1)	100.8<br>(100.6 - 100.9)	
29	90.2<br>(82.8 - 103.0)	81.0<br>(72.9 - 83.9)	77.3<br>(73.7 - 84.1)	74.5<br>(74.1 - 74.8)	75.2<br>(75.0 - 75.5)	75.9<br>(75.7 - 76.2)	76.5<br>(76.4 - 76.7)	
30	93.2<br>(63.3 - 106.1)	84.2<br>(84.0 - 84.4)	83.7<br>(83.5 - 83.9)	83.3<br>(83.1 - 83.4)	82.8<br>(82.6 - 83.0)	82.3<br>(82.2 - 82.5)	82.1<br>(82.1 - 82.1)	
31	92.0<br>(91.6 - 92.3)	73.4<br>(58.1 - 92.6)	58.4<br>(58.2 - 58.6)	58.9<br>(58.8 - 59.0)	59.4<br>(59.2 - 59.6)	59.9<br>(59.8 - 60.1)	60.4<br>(60.2 - 60.5)	
33	103.1<br>(98.2 - 110.3)	98.6<br>(98.4 - 98.7)	98.9<br>(98.8 - 99.0)	105.4<br>(99.1 - 124.2)	124.4<br>(124.3 - 124.6)	124.9<br>(124.7 - 125.1)	125.3<br>(125.2 - 125.4)	
34	104.8<br>(104.6 - 105.1)	104.3<br>(104.1 - 104.4)	103.8<br>(103.6 - 103.9)	103.3<br>(103.2 - 103.5)	92.6<br>(61.6 - 103.1)	102.4<br>(102.2 - 102.5)	101.8<br>(101.7 - 101.9)	101.5<br>(101.3 - 101.6)
35	90.9<br>(90.8 - 91.1)	90.7<br>(90.6 - 90.8)	90.4<br>(90.3 - 90.5)	90.2<br>(90.1 - 90.3)	90.0<br>(89.9 - 90.0)	89.7<br>(89.6 - 89.8)	89.5<br>(89.4 - 89.5)	
41	104.7<br>(104.7 - 104.8)	104.9<br>(104.8 - 104.9)	105.0<br>(105.0 - 105.1)	105.2<br>(105.1 - 105.2)	105.3<br>(105.3 - 105.4)	105.5<br>(105.5 - 105.5)	105.7<br>(105.6 - 105.7)	

(a) 1 month = 28 days as defined in Section 6 of the statistical analysis plan; Month 1 starts at 1st infusion of Subgam (Infusion 4)

Certain data have been excluded from the efficacy analysis. See filenote 001, Section 16.1.9, for details of data exclusions from this table

Program: T0127.TEM, Version: 8.2, Datetime: 07FEB07:09:48

Table 14.2.4.1: Mean (range) Subgam dose (mg/kg) received in Stage 1 of the study - all patients

Patient	Stage 1 Month 1(a)	Month 2	Month 3	Month 4	Month 5	Month 6	Month 7	Month 8
43	101.6 (101.3 - 101.8)	101.1 (100.9 - 101.2)	100.7 (100.5 - 100.8)	100.2 (100.0 - 100.4)	99.7 (99.6 - 99.9)	99.3 (99.2 - 99.4)	98.9 (98.7 - 99.1)	
49	95.1 (94.3 - 95.8)	93.4 (92.9 - 93.9)	95.8 (91.8 - 106.9)	105.9 (105.3 - 106.5)	104.3 (103.7 - 104.9)	102.8 (102.4 - 103.4)	101.6 (101.3 - 101.9)	
53	104.2 (98.3 - 106.4)	105.6 (105.4 - 105.9)	105.0 (104.8 - 105.2)	104.4 (104.2 - 104.6)	129.6 (103.5 - 155.7)	103.1 (102.9 - 103.4)	111.2 (102.6 - 128.2)	
54	80.1 (32.3 - 101.1)	101.2 (101.2 - 101.3)	101.5 (101.4 - 101.5)	101.7 (101.6 - 101.7)	101.8 (101.8 - 101.9)	102.0 (101.9 - 102.1)	102.2 (102.2 - 102.3)	
55	91.7 (41.5 - 116.6)	115.9 (115.7 - 116.1)	115.2 (115.0 - 115.5)	133.7 (114.7 - 171.7)	128.2 (113.7 - 171.2)	113.3 (113.1 - 113.5)	112.7 (112.6 - 112.9)	
56	82.8 (32.0 - 106.2)	106.3 (105.5 - 107.6)	107.2 (107.0 - 107.4)	106.6 (106.4 - 106.8)	106.1 (105.9 - 106.2)	105.5 (105.3 - 105.7)	105.0 (104.8 - 105.2)	104.8 (104.8 - 104.8)
57	81.1 (80.7 - 81.5)	100.2 (99.9 - 100.6)	119.2 (118.7 - 119.6)	118.0 (117.6 - 118.5)	116.9 (116.5 - 117.4)	115.8 (115.4 - 116.2)	114.7 (114.2 - 115.1)	113.9 (113.9 - 113.9)
58	102.4 (96.1 - 105.8)	102.0 (96.2 - 107.8)	102.1 (96.3 - 107.9)	100.2 (96.4 - 107.9)				
61	109.9 (109.2 - 110.7)	108.2 (107.7 - 108.8)	102.6 (102.0 - 103.1)	101.2 (100.7 - 101.7)	102.0 (100.1 - 103.9)	102.7 (102.2 - 103.2)	101.6 (101.3 - 101.9)	

(a) 1 month = 28 days as defined in Section 6 of the statistical analysis plan; Month 1 starts at 1st infusion of Subgam (Infusion 4)

Certain data have been excluded from the efficacy analysis. See filenote 001, Section 16.1.9, for details of data exclusions from this table

Program: T0127.TEM, Version: 8.2, Datetime: 07FEB07:09:48

Table 14.2.4.1: Mean (range) Subgam dose (mg/kg) received in Stage 1 of the study - all patients

Patient	Stage 1							
	Month 1(a)	Month 2	Month 3	Month 4	Month 5	Month 6	Month 7	Month 8
65	98.4<br>(97.7 - 99.1)	96.8<br>(96.3 - 97.3)	95.4<br>(94.9 - 95.9)	94.1<br>(93.6 - 94.6)	92.8<br>(92.3 - 93.3)	91.5<br>(91.0 - 92.0)	90.5<br>(90.3 - 90.7)	
73	100.3<br>(100.2 - 100.4)	97.9<br>(91.1 - 100.2)	101.7<br>(101.6 - 101.8)	101.5<br>(101.4 - 101.6)	101.3<br>(101.2 - 101.4)	102.4<br>(101.1 - 106.4)	97.4<br>(79.7 - 106.2)	
74	99.5<br>(99.4 - 99.7)	99.1<br>(99.0 - 99.3)	100.7<br>(100.6 - 100.8)	100.3<br>(100.2 - 100.5)	100.0<br>(99.8 - 100.1)	101.5<br>(99.5 - 107.1)	105.0<br>(104.9 - 105.1)	
75	99.5<br>(99.4 - 99.6)	99.2<br>(99.2 - 99.3)	101.2<br>(101.1 - 101.3)	105.4<br>(105.3 - 105.5)	105.2<br>(105.1 - 105.3)	104.9<br>(104.8 - 105.0)	108.0<br>(104.8 - 109.1)	108.9<br>(108.9 - 108.9)
76	100.6<br>(100.3 - 100.9)	101.3<br>(101.1 - 101.4)	104.2<br>(104.0 - 104.5)	104.9<br>(104.6 - 105.1)	105.6<br>(105.3 - 105.9)	106.3<br>(106.1 - 106.5)	104.3<br>(99.5 - 106.8)	
77	134.6<br>(99.3 - 143.4)	143.4<br>(143.4 - 143.4)	143.4<br>(143.4 - 143.4)	143.4<br>(143.4 - 143.4)	143.4<br>(143.4 - 143.4)	143.4<br>(143.4 - 143.4)	143.4<br>(143.4 - 143.4)	
78	142.6<br>(104.0 - 152.4)	151.9<br>(151.7 - 152.0)	151.5<br>(151.3 - 151.6)	151.1<br>(151.0 - 151.3)	150.7<br>(150.6 - 150.9)	150.3<br>(150.2 - 150.5)	150.0<br>(149.9 - 150.1)	
81	115.9<br>(81.3 - 124.8)	123.7<br>(123.4 - 124.0)	122.8<br>(122.5 - 123.1)	122.0<br>(121.7 - 122.3)	121.1<br>(120.8 - 121.4)	120.3<br>(120.0 - 120.6)	119.6<br>(119.4 - 119.8)	
82	100.7<br>(100.3 - 101.1)	99.8<br>(99.5 - 100.1)	123.8<br>(123.5 - 124.2)	122.9<br>(122.5 - 123.2)	121.9<br>(121.6 - 122.3)	121.0<br>(120.7 - 121.4)	133.6<br>(120.2 - 160.0)	

(a) 1 month = 28 days as defined in Section 6 of the statistical analysis plan; Month 1 starts at 1st infusion of Subgam (Infusion 4)

Certain data have been excluded from the efficacy analysis. See filenote 001, Section 16.1.9, for details of data exclusions from this table

Program: T0127.TEM, Version: 8.2, Datetime: 07FEB07:09:48

Table 14.2.4.1: Mean (range) Subgam dose (mg/kg) received in Stage 1 of the study - all patients

Patient	Stage 1							
	Month 1(a)	Month 2	Month 3	Month 4	Month 5	Month 6	Month 7	Month 8
83	103.8<br>(102.1 - 105.7)	99.8<br>(98.5 - 101.1)	137.9<br>(136.2 - 139.7)	133.4<br>(131.9 - 135.1)	129.3<br>(127.8 - 130.8)	125.3<br>(123.9 - 126.8)	122.1<br>(121.2 - 123.0)	
84	94.4<br>(94.0 - 94.8)	93.5<br>(93.2 - 93.8)	92.6<br>(92.3 - 92.9)	91.8<br>(91.5 - 92.1)	98.6<br>(90.9 - 121.0)	120.3<br>(119.9 - 120.7)	119.4<br>(119.2 - 119.7)	
85	114.4<br>(114.0 - 114.9)	113.2<br>(112.8 - 113.7)	112.0<br>(111.6 - 112.6)	110.9<br>(110.6 - 111.1)	110.0<br>(109.4 - 110.4)	108.8<br>(108.4 - 109.1)	119.7<br>(107.8 - 143.2)	
86	95.5<br>(61.5 - 104.8)	84.8<br>(75.3 - 91.3)	97.7<br>(93.9 - 99.8)	92.4<br>(73.7 - 97.8)	90.4<br>(76.8 - 95.4)	92.2<br>(89.4 - 93.6)	91.8<br>(91.4 - 92.2)	

(a) 1 month = 28 days as defined in Section 6 of the statistical analysis plan; Month 1 starts at 1st infusion of Subgam (Infusion 4)

Certain data have been excluded from the efficacy analysis. See filenote 001, Section 16.1.9, for details of data exclusions from this table

Program: T0127.TEM, Version: 8.2, Datetime: 07FEB07:09:48

Table 14.2.4.1: Mean (range) Subgam dose (mg/kg) received in Stage 1 of the study - all patients

	Stage 1							
	Month 1(a)	Month 2	Month 3	Month 4	Month 5	Month 6	Month 7	Month 8
n	49	49	49	49	48	48	46	6
Total Mean of Means	101.8	103.0	104.4	105.2	105.9	106.0	107.3	103.2
Median	100.3	100.1	101.5	101.7	101.9	102.6	104.3	103.1
SD	12.0	14.6	16.5	17.2	18.0	17.4	18.4	8.2
Min	80.1	73.4	58.4	58.9	59.4	59.9	60.4	90.1
Max	142.6	151.9	151.5	151.1	150.7	150.3	150.0	113.9
Lower 95% CI	98.4	98.8	99.7	100.3	100.7	101.0	101.8	94.6
Upper 95% CI	105.3	107.1	109.1	110.2	111.1	111.1	112.7	111.8

(a) 1 month = 28 days as defined in Section 6 of the statistical analysis plan; Month 1 starts at 1st infusion of Subgam (Infusion 4)

Certain data have been excluded from the efficacy analysis. See filenote 001, Section 16.1.9, for details of data exclusions from this table

Program: T0127.TEM, Version: 8.2, Datetime: 07FEB07:09:48

Table 14.2.4.2: Mean Subgam dose (mg/kg) received in Stage 1 of the study - by diagnosis of PAD

By Diagnosis of PAD		Stage 1							
		Month 1(a)	Month 2	Month 3	Month 4	Month 5	Month 6	Month 7	Month 8
CVID/XLA	n	32	32	32	32	32	32	31	5
	Total Mean of Means	100.7	101.7	102.3	102.4	102.6	104.4	105.2	102.0
	Median	100.1	99.6	101.1	101.6	101.3	102.4	102.2	101.5
	SD	12.4	15.3	16.7	17.0	17.6	18.1	19.0	8.6
	Min	80.1	73.4	58.4	58.9	59.4	59.9	60.4	90.1
	Max	135.4	144.2	145.3	146.4	147.6	148.7	149.7	113.9
	Lower 95% CI	96.2	96.2	96.3	96.3	96.3	97.9	98.3	91.4
	Upper 95% CI	105.2	107.2	108.3	108.5	108.9	111.0	112.2	112.7
Other	n	17	17	17	17	16	16	15	1
	Total Mean of Means	103.9	105.3	108.4	110.6	112.6	109.3	111.5	108.9
	Median	101.0	101.1	101.8	102.4	105.5	104.0	108.0	108.9
	SD	11.2	13.2	15.7	16.8	17.4	16.0	17.1	
	Min	91.7	94.4	93.7	92.6	90.8	89.0	87.4	108.9
	Max	142.6	151.9	151.5	151.1	150.7	150.3	150.0	108.9
	Lower 95% CI	98.1	98.5	100.4	101.9	103.3	100.8	102.0	.
	Upper 95% CI	109.7	112.1	116.5	119.2	121.9	117.8	120.9	.

(a) 1 month = 28 days as defined in Section 6 of the statistical analysis plan; Month 1 starts at 1st infusion of Subgam (Infusion 4)

Certain data have been excluded from the efficacy analysis. See filenote 001, Section 16.1.9, for details of data exclusions from this table

Program: T0133.TEM, Version: 8.2, Datetime: 07FEB07:09:48

Table 14.2.4.3: Mean Subgam dose (mg/kg) received in Stage 1 of the study - by age group

By Age group		Stage 1 Month 1(a)	Month 2	Month 3	Month 4	Month 5	Month 6	Month 7	Month 8
Adult	n	27	27	27	27	26	26	24	3
	Total Mean of Means	104.2	104.8	104.5	104.8	106.4	107.0	109.2	103.5
	Median	100.8	101.2	101.5	101.7	101.9	102.4	104.7	101.5
	SD	14.0	17.8	19.2	19.4	20.8	20.4	21.5	4.8
	Min	80.1	73.4	58.4	58.9	59.4	59.9	60.4	100.0
	Max	142.6	151.9	151.5	151.1	150.7	150.3	150.0	108.9
	Lower 95% CI	98.6	97.8	96.9	97.1	97.9	98.7	100.1	91.6
	Upper 95% CI	109.7	111.9	112.1	112.5	114.8	115.2	118.3	115.3
Teenager	n	7	7	7	7	7	7	7	1
	Total Mean of Means	94.5	99.6	99.7	103.5	103.3	103.8	103.3	104.8
	Median	94.4	99.1	98.6	105.2	104.3	105.5	105.0	104.8
	SD	6.8	10.4	10.4	15.8	13.6	12.1	12.0	
	Min	82.8	84.2	83.7	83.3	82.8	82.3	82.1	104.8
	Max	104.7	115.9	115.2	133.7	128.2	120.3	119.4	104.8
	Lower 95% CI	88.2	90.0	90.1	88.8	90.7	92.6	92.2	.
	Upper 95% CI	100.8	109.2	109.3	118.2	115.8	115.0	114.3	.
Child	n	15	15	15	15	15	15	15	2
	Total Mean of Means	101.0	101.1	106.4	106.8	106.4	105.5	106.0	102.0
	Median	100.1	99.8	101.7	101.5	102.0	102.7	101.6	102.0
	SD	8.4	8.9	13.5	14.3	15.1	14.4	16.0	16.8
	Min	81.1	84.8	93.7	92.4	90.4	89.0	87.4	90.1
	Max	115.9	123.7	137.9	133.4	134.8	132.8	133.6	113.9
	Lower 95% CI	96.3	96.2	98.9	98.9	98.1	97.5	97.2	-49.3
	Upper 95% CI	105.7	106.0	113.9	114.7	114.8	113.5	114.9	253.2

(a) 1 month = 28 days as defined in Section 6 of the statistical analysis plan; Month 1 starts at 1st infusion of Subgam (Infusion 4)

Certain data have been excluded from the efficacy analysis. See filenote 001, Section 16.1.9, for details of data exclusions from this table

Program: T0134.TEM, Version: 8.2, Datetime: 07FEB07:09:48

Table 14.2.4.4: Mean Subgam dose (mg/kg) received in Stage 1 of the study - by prior therapy

By Prior Therapy		Stage 1							
		Month 1(a)	Month 2	Month 3	Month 4	Month 5	Month 6	Month 7	Month 8
IVIG	n	35	35	35	35	34	34	32	3
	Total Mean of Means	103.0	104.2	105.5	105.9	106.0	107.1	108.5	102.1
	Median	100.7	101.1	101.5	101.7	101.9	102.8	103.6	101.5
	SD	13.7	17.1	19.1	19.6	19.9	19.6	20.8	2.5
	Min	80.1	73.4	58.4	58.9	59.4	59.9	60.4	100.0
	Max	142.6	151.9	151.5	151.1	150.7	150.3	150.0	104.8
	Lower 95% CI	98.3	98.3	99.0	99.2	99.1	100.3	100.9	96.0
	Upper 95% CI	107.7	110.1	112.1	112.6	113.0	113.9	116.0	108.2
SCIG	n	14	14	14	14	14	14	14	3
	Total Mean of Means	98.8	99.9	101.6	103.5	105.7	103.5	104.5	104.3
	Median	99.9	99.2	101.5	101.8	101.7	102.4	104.3	108.9
	SD	5.6	2.9	6.0	9.1	12.9	10.6	11.3	12.6
	Min	81.1	94.4	93.7	93.0	92.3	91.6	90.5	90.1
	Max	104.2	105.6	119.2	127.3	134.8	132.8	130.6	113.9
	Lower 95% CI	95.6	98.2	98.1	98.2	98.2	97.4	98.0	73.1
	Upper 95% CI	102.0	101.5	105.0	108.8	113.2	109.6	111.0	135.5

(a) 1 month = 28 days as defined in Section 6 of the statistical analysis plan; Month 1 starts at 1st infusion of Subgam (Infusion 4)

Certain data have been excluded from the efficacy analysis. See filenote 001, Section 16.1.9, for details of data exclusions from this table

Program: T0140.TEM, Version: 8.2, Datetime: 07FEB07:09:48

Table 14.2.5.1: Mean (range) Subgam dose (mg/kg) received in 6-monthly intervals - all patients

	Stage 1	Stage 2							
Patient	0 to 6 months(a)	>6 to 12 months(b)	>12 to 18 months	>18 to 24 months	>24 to 30 months	>30 to 36 months	>36 to 42 months	>42 to 48 months	>48 to 54 months
01	93.2 (90.9-95.6)	88.2 (85.8-90.6)	83.0 (80.6-85.6)	78.1 (75.9-80.4)	73.7 (71.8-75.7)	69.9 (68.1-71.7)	67.4 (66.7-68.0)		
05	102.1 (92.9-106.5)	100.6 (96.9-103.9)	97.1 (93.5-102.3)	99.7 (99.1-100.3)					
06	98.2 (96.4-100.0)	96.1 (95.9-96.3)	95.6 (95.4-95.9)	95.1 (94.8-95.3)	101.6 (94.8-110.7)	110.3 (110.0-110.6)	106.2 (97.3-110.0)	72.5 (55.7-109.4)	55.6 (55.6-55.6)
07	99.1 (98.4-99.8)	98.3 (98.3-98.3)	98.3 (98.3-98.3)	98.3 (98.3-98.3)	98.3 (98.3-98.3)	98.3 (98.3-98.3)	98.3 (98.3-98.3)	98.3 (98.3-98.3)	
08	104.0 (101.4-131.3)	152.7 (130.6-195.3)	194.2 (193.3-195.1)	182.5 (112.5-193.2)	111.9 (111.4-112.4)	110.9 (110.4-111.4)	109.9 (109.4-110.3)	109.0 (108.7-109.3)	
09	100.3 (99.8-100.8)								
10	100.0 (100.0-100.1)	100.6 (100.0-101.4)	102.6 (101.5-103.6)	104.8 (103.7-106.0)	107.2 (106.1-108.4)	109.8 (108.5-111.0)	112.4 (111.1-113.7)	134.0 (113.8-154.5)	155.8 (154.8-156.7)
11	94.1 (91.0-97.2)	94.0 (93.7-94.6)	95.1 (94.6-95.6)	121.7 (59.9-161.1)	87.2 (64.5-161.5)	119.2 (65.2-164.8)	228.8 (82.5-250.0)	250.5 (250.1-250.9)	

(a) This interval lasts approx. 6 months, depending on the number of infusions received by the patient during Stage 1.
(b) This interval lasts approx. 6 months, from the first infusion in Stage 2 until 12 months after the first infusion of Subgam in Stage 1.

Certain data have been excluded from the efficacy analysis. See filenote 001, Section 16.1.9, for details of data exclusions from this table

Program: T0135.TEM, Version: 8.2, Datetime: 07FEB07:09:48

Table 14.2.5.1: Mean (range) Subgam dose (mg/kg) received in 6-monthly intervals - all patients

	Stage 1	Stage 2							
Patient	0 to 6 months(a)	>6 to 12 months(b)	>12 to 18 months	>18 to 24 months	>24 to 30 months	>30 to 36 months	>36 to 42 months	>42 to 48 months	>48 to 54 months
12	94.1 (91.1-100.3)	92.6 (90.0-93.2)							
13	93.0 (86.9-99.7)	91.7 (83.6-100.0)	101.1 (96.2-105.5)	93.5 (90.3-96.9)	99.8 (89.1-106.3)	98.0 (94.8-101.3)	99.1 (92.7-104.0)	98.2 (96.3-99.9)	
17	101.6 (100.9-102.3)	102.3 (102.2-102.3)	102.1 (102.0-102.2)	101.6 (92.7-102.0)	101.8 (101.7-101.9)	101.6 (101.6-101.7)	101.5 (101.4-101.6)	101.4 (101.3-101.4)	
18	103.7 (101.5-106.0)	106.2 (106.0-106.3)	106.6 (106.4-106.8)	107.0 (106.8-107.2)	107.4 (107.2-107.7)	107.9 (107.7-108.1)	108.3 (108.1-108.5)	108.7 (108.6-108.7)	
21	108.7 (100.6-134.8)	135.6 (134.9-135.8)	135.7 (135.7-135.7)	135.6 (135.6-135.7)	135.5 (135.5-135.6)	133.7 (90.3-135.5)	135.4 (135.3-135.4)	135.3 (135.3-135.3)	135.2 (135.2-135.3)
22	128.8 (100.0-130.4)	147.5 (129.5-174.2)	166.4 (110.2-175.6)	168.2 (110.3-223.0)	112.2 (111.7-112.8)	199.0 (113.0-228.8)	230.3 (228.9-231.7)	232.8 (231.8-233.5)	
23	144.8 (104.5-150.0)	150.3 (150.0-150.7)	151.1 (150.7-151.4)	151.8 (151.5-152.2)	152.6 (152.2-153.0)	153.4 (153.0-153.8)	154.2 (153.8-154.6)	154.7 (154.6-154.8)	
24	111.6 (84.0-115.7)	115.4 (115.1-115.6)	114.7 (114.4-115.1)	114.1 (113.7-114.4)	113.4 (113.1-113.7)	112.7 (112.4-113.1)	122.1 (112.2-127.1)	126.6 (126.5-126.7)	

(a) This interval lasts approx. 6 months, depending on the number of infusions received by the patient during Stage 1.
(b) This interval lasts approx. 6 months, from the first infusion in Stage 2 until 12 months after the first infusion of Subgam in Stage 1.

Certain data have been excluded from the efficacy analysis. See filenote 001, Section 16.1.9, for details of data exclusions from this table

Program: T0135.TEM, Version: 8.2, Datetime: 07FEB07:09:48

Table 14.2.5.1: Mean (range) Subgam dose (mg/kg) received in 6-monthly intervals - all patients

	Stage 1	Stage 2							
Patient	0 to 6 months(a)	>6 to 12 months(b)	>12 to 18 months	>18 to 24 months	>24 to 30 months	>30 to 36 months	>36 to 42 months	>42 to 48 months	>48 to 54 months
25	94.2 (90.1-99.2)	88.1 (83.1-89.8)	85.1 (83.4-86.8)	91.9 (82.9-96.6)	88.9 (87.2-90.6)	85.5 (84.0-87.1)	82.5 (81.2-83.9)	95.4 (95.4-95.4)	
26	97.2 (91.6-101.5)	89.1 (87.3-91.3)	87.2 (83.4-99.9)	97.0 (94.8-99.3)	96.0 (91.7-103.7)	102.1 (102.0-102.3)			
27	117.9 (99.2-137.2)	130.3 (123.9-164.8)	159.7 (155.5-164.0)	151.4 (147.8-155.1)	140.8 (140.3-141.2)	139.5 (138.9-140.0)			
28	101.5 (100.6-102.2)	98.3 (96.2-100.4)	93.8 (91.7-96.0)	89.3 (87.3-91.4)	85.2 (83.3-87.1)	81.5 (79.8-83.3)	79.6 (79.5-79.7)		
29	79.1 (72.9-103.0)	76.7 (76.7-76.7)	76.7 (76.7-76.7)	76.7 (76.7-76.7)	76.7 (76.7-76.7)	76.7 (76.7-76.7)	76.7 (76.7-76.7)	76.7 (76.7-76.7)	76.7 (76.7-76.7)
30	84.9 (63.3-106.1)	82.1 (82.1-82.1)	98.8 (82.1-102.6)	101.1 (73.8-102.6)	103.5 (102.6-123.1)	119.3 (61.5-123.1)	125.8 (123.1-143.6)	123.1 (123.1-123.1)	123.1 (123.1-123.1)
31	66.7 (58.1-92.6)								
33	111.6 (98.2-125.4)	125.3 (125.3-125.4)	125.2 (125.2-125.3)	125.1 (125.1-125.2)	125.0 (124.9-125.1)	134.8 (62.5-149.9)	149.8 (149.8-149.8)		

(a) This interval lasts approx. 6 months, depending on the number of infusions received by the patient during Stage 1.
(b) This interval lasts approx. 6 months, from the first infusion in Stage 2 until 12 months after the first infusion of Subgam in Stage 1.

Certain data have been excluded from the efficacy analysis. See filenote 001, Section 16.1.9, for details of data exclusions from this table

Program: T0135.TEM, Version: 8.2, Datetime: 07FEB07:09:48

Table 14.2.5.1: Mean (range) Subgam dose (mg/kg) received in 6-monthly intervals - all patients

	Stage 1	Stage 2							
Patient	0 to 6 months(a)	>6 to 12 months(b)	>12 to 18 months	>18 to 24 months	>24 to 30 months	>30 to 36 months	>36 to 42 months	>42 to 48 months	>48 to 54 months
34	101.8 (61.6-105.1)	95.1 (60.6-101.1)	100.2 (99.7-100.5)	99.1 (98.7-99.4)	94.5 (39.3-98.6)	97.3 (96.8-97.7)	96.4 (95.9-96.8)	95.4 (95.0-95.9)	94.9 (94.9-94.9)
35	90.2 (89.4-91.1)	89.4 (89.4-89.4)	89.5 (89.4-89.5)	89.5 (89.5-89.5)	89.5 (89.5-89.6)	89.6 (89.6-89.6)	89.6 (89.6-89.7)	89.7 (89.7-89.7)	89.7 (89.7-89.7)
41	105.2 (104.7-105.7)	104.5 (102.6-105.9)	100.1 (97.9-102.4)	95.6 (93.5-97.7)	91.4 (89.5-93.3)	87.6 (86.0-89.3)	84.1 (82.6-85.7)	80.9 (79.4-82.4)	78.4 (77.6-79.3)
43	100.2 (98.7-101.8)	99.8 (98.5-101.4)							
49	99.6 (91.8-106.9)	99.3 (98.0-100.9)	96.4 (95.0-97.9)	93.5 (92.2-94.9)	90.8 (89.5-92.1)	93.6 (88.1-100.5)	98.0 (96.7-99.4)	95.9 (95.2-96.6)	
53	109.0 (98.3-155.7)	127.7 (127.3-128.1)	126.8 (126.3-127.2)	125.7 (125.2-126.2)	143.4 (100.0-150.0)	148.6 (148.2-149.1)			
54	97.9 (32.3-102.3)	101.7 (101.2-102.3)	100.5 (99.9-101.1)	99.3 (98.7-99.9)	98.1 (97.5-98.7)	96.9 (96.3-97.5)	96.2 (96.2-96.2)		
55	114.4 (41.5-171.7)	106.8 (94.3-112.5)	110.2 (103.5-158.0)	102.9 (101.1-104.5)	102.1 (101.6-102.5)	101.0 (100.6-101.5)	100.4 (100.2-100.5)		

(a) This interval lasts approx. 6 months, depending on the number of infusions received by the patient during Stage 1.
(b) This interval lasts approx. 6 months, from the first infusion in Stage 2 until 12 months after the first infusion of Subgam in Stage 1.

Certain data have been excluded from the efficacy analysis. See filenote 001, Section 16.1.9, for details of data exclusions from this table

Program: T0135.TEM, Version: 8.2, Datetime: 07FEB07:09:48

Table 14.2.5.1: Mean (range) Subgam dose (mg/kg) received in 6-monthly intervals - all patients

	Stage 1	Stage 2							
Patient	0 to 6 months(a)	>6 to 12 months(b)	>12 to 18 months	>18 to 24 months	>24 to 30 months	>30 to 36 months	>36 to 42 months	>42 to 48 months	>48 to 54 months
56	101.9 (32.0-107.6)	105.0 (104.8-105.3)	105.6 (105.3-105.9)	106.2 (105.9-106.5)	106.8 (106.5-107.1)	107.4 (107.1-107.7)	107.8 (107.8-107.9)		
57	109.2 (80.7-119.6)	120.0 (110.5-137.9)	132.8 (129.7-136.0)	126.2 (123.2-129.4)	120.3 (117.6-123.0)	114.9 (112.4-117.4)	110.7 (109.1-112.3)		
58	102.0 (96.1-107.9)	101.2 (96.1-108.0)	100.9 (95.9-105.7)	100.6 (95.7-105.5)	100.4 (95.5-105.3)	100.1 (95.4-105.1)	99.7 (85.8-104.9)		
61	104.3 (100.1-110.7)	96.3 (92.0-100.8)	102.2 (90.3-107.9)	95.4 (91.2-99.8)	89.1 (87.4-90.9)				
65	94.5 (90.3-99.1)	90.3 (90.3-90.3)	90.3 (90.3-90.3)	90.3 (90.3-90.3)	90.3 (90.3-90.3)				
73	100.5 (79.7-106.4)	106.3 (106.2-106.3)	106.4 (106.3-106.4)	106.5 (106.4-106.6)	106.6 (106.6-106.7)	106.8 (106.7-106.8)	106.9 (106.8-106.9)		
74	100.7 (99.0-107.1)	104.4 (104.0-104.8)	103.4 (102.9-103.9)	102.4 (101.9-102.9)	101.4 (100.9-101.9)	100.4 (100.0-100.9)	99.6 (99.2-99.9)		
75	103.5 (99.2-109.1)	108.2 (107.7-108.8)	104.6 (95.5-107.6)	94.8 (94.2-95.5)	97.3 (83.7-103.7)	103.1 (102.9-103.2)			

(a) This interval lasts approx. 6 months, depending on the number of infusions received by the patient during Stage 1.
(b) This interval lasts approx. 6 months, from the first infusion in Stage 2 until 12 months after the first infusion of Subgam in Stage 1.

Certain data have been excluded from the efficacy analysis. See filenote 001, Section 16.1.9, for details of data exclusions from this table

Program: T0135.TEM, Version: 8.2, Datetime: 07FEB07:09:48

Table 14.2.5.1: Mean (range) Subgam dose (mg/kg) received in 6-monthly intervals - all patients

	Stage 1	Stage 2							
Patient	0 to 6 months(a)	>6 to 12 months(b)	>12 to 18 months	>18 to 24 months	>24 to 30 months	>30 to 36 months	>36 to 42 months	>42 to 48 months	>48 to 54 months
76	103.8 (99.5-106.8)								
77	141.9 (99.3-143.4)	143.8 (143.4-144.2)	144.6 (144.2-145.0)	145.5 (145.1-145.9)	157.6 (145.9-169.3)	169.9 (169.4-170.4)	170.9 (170.4-171.4)		
78	149.5 (104.0-152.4)	149.7 (149.6-149.8)	149.5 (149.4-149.6)	149.4 (149.3-149.4)	149.2 (149.1-149.3)	149.0 (148.9-149.1)	148.9 (148.8-148.9)		
81	120.6 (81.3-124.8)	118.3 (117.4-119.3)	166.2 (117.0-175.4)	178.2 (169.7-226.2)	213.8 (166.1-225.6)	164.6 (163.4-165.8)			
82	116.5 (99.5-160.0)	159.1 (158.3-159.9)	155.6 (118.2-158.2)	185.3 (155.9-194.8)	200.2 (191.3-229.4)	227.6 (226.5-228.7)			
83	121.0 (98.5-139.7)	114.4 (108.8-120.6)	149.4 (107.7-160.7)	173.8 (142.9-189.8)	168.3 (161.1-175.9)	155.0 (149.7-160.5)			
84	100.6 (90.9-121.0)	118.0 (115.0-138.7)	112.6 (110.5-114.8)	108.3 (106.4-110.3)	102.2 (51.3-106.2)	101.2 (100.0-102.4)			
85	112.4 (107.8-143.2)	136.7 (131.0-142.6)	124.2 (118.9-130.2)	133.6 (115.9-144.1)	130.2 (125.5-135.3)	123.6 (122.0-125.1)			

(a) This interval lasts approx. 6 months, depending on the number of infusions received by the patient during Stage 1.
(b) This interval lasts approx. 6 months, from the first infusion in Stage 2 until 12 months after the first infusion of Subgam in Stage 1.

Certain data have been excluded from the efficacy analysis. See filenote 001, Section 16.1.9, for details of data exclusions from this table

Program: T0135.TEM, Version: 8.2, Datetime: 07FEB07:09:48

Table 14.2.5.1: Mean (range) Subgam dose (mg/kg) received in 6-monthly intervals - all patients

Patient	Stage 1	Stage 2							
	0 to 6 months(a)	>6 to 12 months(b)	>12 to 18 months	>18 to 24 months	>24 to 30 months	>30 to 36 months	>36 to 42 months	>42 to 48 months	>48 to 54 months
86	92.1 (61.5-104.8)	89.7 (88.3-91.2)	101.4 (43.8-109.2)	121.1 (105.3-126.1)	119.9 (117.8-122.2)	117.6 (117.5-117.7)			

(a) This interval lasts approx. 6 months, depending on the number of infusions received by the patient during Stage 1.
(b) This interval lasts approx. 6 months, from the first infusion in Stage 2 until 12 months after the first infusion of Subgam in Stage 1.

Certain data have been excluded from the efficacy analysis. See filenote 001, Section 16.1.9, for details of data exclusions from this table

Program: T0135.TEM, Version: 8.2, Datetime: 07FEB07:09:48

Table 14.2.5.1: Mean (range) Subgam dose (mg/kg) received in 6-monthly intervals - all patients

	Stage 1	Stage 2							
	0 to 6 months(a)	>6 to 12 months(b)	>12 to 18 months	>18 to 24 months	>24 to 30 months	>30 to 36 months	>36 to 42 months	>42 to 48 months	>48 to 54 months
n	49	46	44	44	43	41	31	19	8
Total Mean of Means.	104.6	109.7	114.6	116.1	113.5	117.6	116.0	119.9	101.2
Median	101.8	103.3	103.0	103.9	102.2	107.9	106.2	101.4	92.3
SD	14.9	20.9	26.8	29.0	30.2	32.3	38.4	47.9	33.8
Min	66.7	76.7	76.7	76.7	73.7	69.9	67.4	72.5	55.6
Max	149.5	159.1	194.2	185.3	213.8	227.6	230.3	250.5	155.8
Lower 95% CI	100.3	103.5	106.5	107.3	104.2	107.3	102.0	96.8	72.9
Upper 95% CI	108.8	115.9	122.8	124.9	122.8	127.8	130.1	143.0	129.4

(a) This interval lasts approx. 6 months, depending on the number of infusions received by the patient during Stage 1.
(b) This interval lasts approx. 6 months, from the first infusion in Stage 2 until 12 months after the first infusion of Subgam in Stage 1.

Certain data have been excluded from the efficacy analysis. See filenote 001, Section 16.1.9, for details of data exclusions from this table

Program: T0135.TEM, Version: 8.2, Datetime: 07FEB07:09:48

Table 14.2.5.2: Mean (range) Subgam dose (mg/kg) received in 6-monthly intervals - completers(c)

	Stage 1	Stage 2							
	0 to 6 months(a)	>6 to 12 months(b)	>12 to 18 months	>18 to 24 months	>24 to 30 months	>30 to 36 months	>36 to 42 months	>42 to 48 months	>48 to 54 months
n	32	32	32	32	32	31	23	15	8
Total Mean of Means	104.8	110.4	114.9	117.0	115.8	118.0	115.1	114.4	101.2
Median	101.9	104.4	103.0	103.9	102.1	107.4	101.5	98.3	92.3
SD	13.7	21.0	25.8	29.4	32.3	31.0	33.7	44.2	33.8
Min	79.1	76.7	76.7	76.7	76.7	76.7	76.7	72.5	55.6
Max	144.8	159.1	194.2	185.3	213.8	227.6	228.8	250.5	155.8
Lower 95% CI	99.9	102.8	105.6	106.5	104.1	106.6	100.5	89.9	72.9
Upper 95% CI	109.8	117.9	124.2	127.6	127.4	129.4	129.7	138.9	129.4

(a) This interval lasts approx. 6 months, depending on the number of infusions received by the patient during Stage 1.
(b) This interval lasts approx. 6 months, from the first infusion in Stage 2 until 12 months after the first infusion of Subgam in Stage 1.
(c) Completers are defined in Section 5 of the statistical analysis plan.

Certain data have been excluded from the efficacy analysis. See filenote 001, Section 16.1.9, for details of data exclusions from this table

Program: T0136.TEM, Version: 8.2, Datetime: 07FEB07:09:48

Figure 14.2.5.3 'Mean of means' of Subgam dosage (mg/kg) received in 6−monthly intervals − all patients and completers(c)

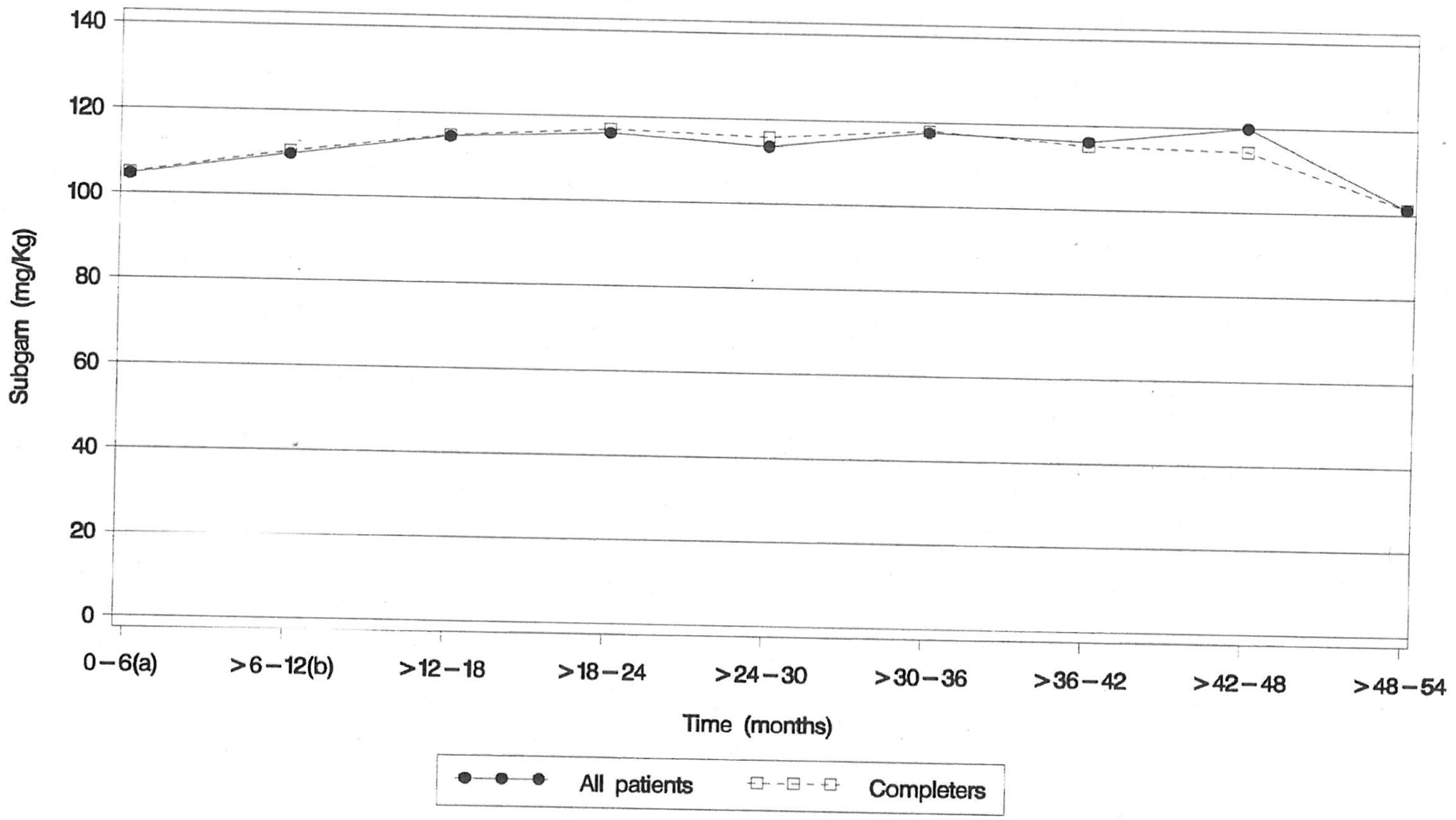

(a) This interval lasts approximately 6 months, depending on the number of infusions received by the patient during Stage 1, and contains post−Subgam data only
(b) This interval lasts approximately 6 months, from the first infusion in Stage 2 until 12 months after the first infusion of Subgam in Stage 1
(c) Completers are defined in Section 5 of the statistical analysis plan

Certain data have been excluded from the efficacy analysis. See filenote 001, Section 16.1.9, for details of data exclusions from this figure

Program: T0139.SAS, Version: 8.2, Datetime: 07FEB2007:09:47

Table 14.2.5.4: Mean Subgam dose (mg/kg) received in 6-monthly intervals - by diagnosis of PAD, all patients

By Diagnosis of PAD		Stage 1	Stage 2							
		0 to 6 months(a)	>6 to 12 months(b)	>12 to 18 months	>18 to 24 months	>24 to 30 months	>30 to 36 months	>36 to 42 months	>42 to 48 months	>48 to 54 months
CVID/XLA	n	32	30	29	29	29	28	24	16	8
	Total Mean of Means	102.6	107.9	113.1	114.6	109.9	114.5	120.1	123.8	101.2
	Median	100.7	103.1	102.6	106.2	103.5	108.8	108.1	108.8	92.3
	SD	15.4	20.8	28.1	27.4	27.4	28.2	41.2	51.5	33.8
	Min	66.7	76.7	76.7	76.7	76.7	76.7	76.7	72.5	55.6
	Max	144.8	152.7	194.2	182.5	213.8	199.0	230.3	250.5	155.8
	Lower 95% CI	97.1	100.1	102.4	104.2	99.5	103.5	102.7	96.4	72.9
	Upper 95% CI	108.2	115.6	123.7	125.0	120.3	125.4	137.5	151.3	129.4
Other	n	17	16	15	15	14	13	7	3	
	Total Mean of Means	108.2	113.2	117.6	119.0	121.0	124.3	102.2	99.3	
	Median	103.5	104.5	104.6	101.6	101.9	103.1	99.7	98.3	
	SD	13.5	21.2	24.8	32.5	35.3	40.3	23.9	1.8	
	Min	93.0	88.2	83.0	78.1	73.7	69.9	67.4	98.2	
	Max	149.5	159.1	159.7	185.3	200.2	227.6	148.9	101.4	
	Lower 95% CI	101.3	101.9	103.9	100.9	100.7	99.9	80.1	94.8	
	Upper 95% CI	115.2	124.5	131.4	137.0	141.4	148.6	124.3	103.8	

(a) This interval lasts approx. 6 months, depending on the number of infusions received by the patient during Stage 1.
(b) This interval lasts approx. 6 months, from the first infusion in Stage 2 until 12 months after the first infusion of Subgam in Stage 1.

Certain data have been excluded from the efficacy analysis. See filenote 001, Section 16.1.9, for details of data exclusions from this table

Program: T0363.TEM, Version: 8.2, Datetime: 07FEB07:09:48

Figure 14.2.5.5 'Mean of means' of Subgam dosage (mg/kg) received in 6-monthly intervals - by Diagnosis of PAD, all patients

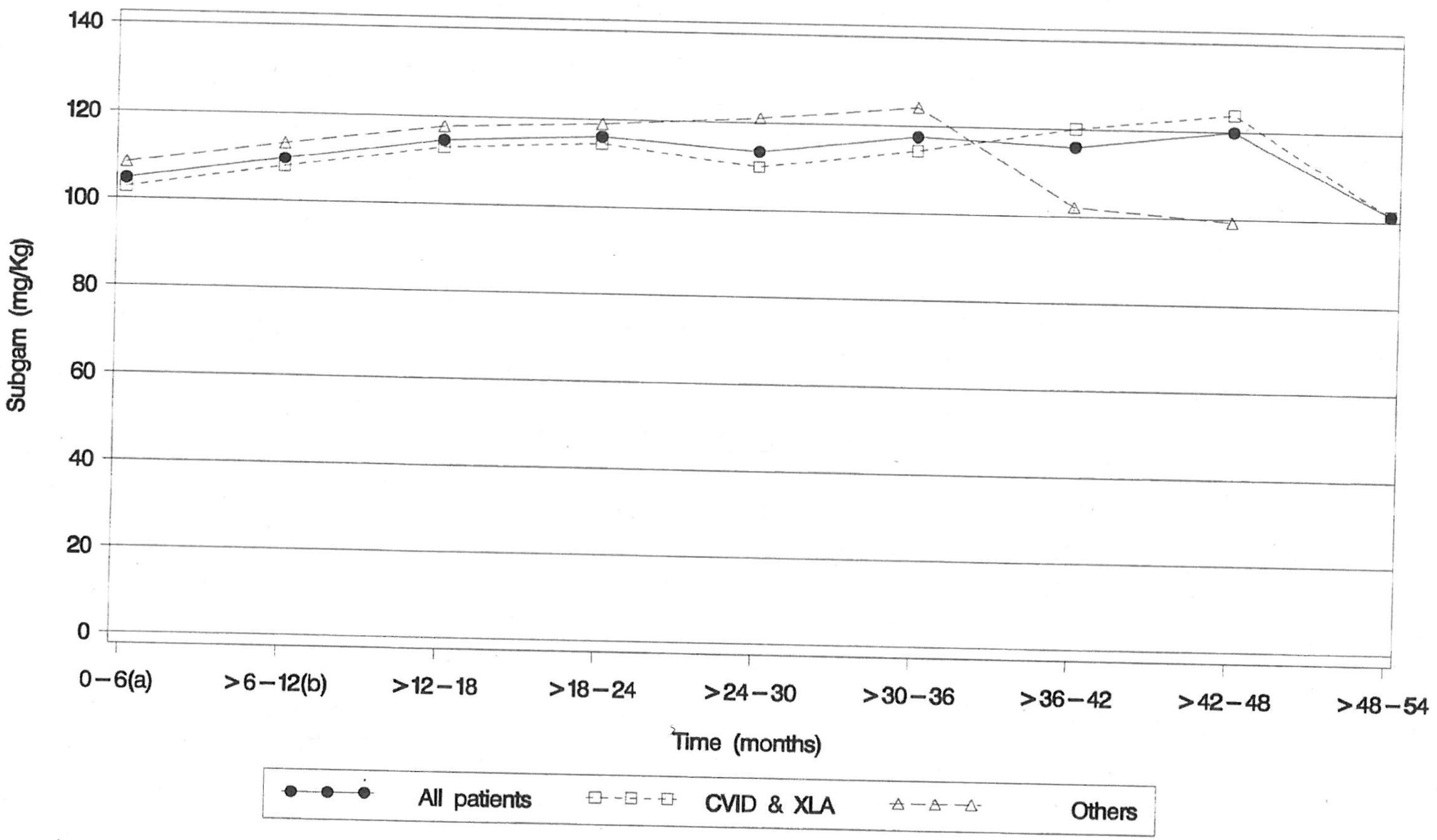

(a) This interval lasts approximately 6 months, depending on the number of infusions received by the patient during Stage 1, and contains post-Subgam data only
(b) This interval lasts approximately 6 months, from the first infusion in Stage 2 until 12 months after the first infusion of Subgam in Stage 1

Certain data have been excluded from the efficacy analysis. See filenote 001, Section 16.1.9, for details of data exclusions from this figure

Program: T0330.SAS, Version: 8.2, Datetime: 07FEB2007:09:47

Table 14.2.5.6: Mean Subgam dose (mg/kg) received in 6-monthly intervals - by age group

By Age group		Stage 1	Stage 2							
		0 to 6 months(a)	>6 to 12 months(b)	>12 to 18 months	>18 to 24 months	>24 to 30 months	>30 to 36 months	>36 to 42 months	>42 to 48 months	>48 to 54 months
Adult	n	27	24	22	22	22	22	20	13	5
	Total Mean of Means	105.5	113.5	118.0	118.2	112.2	119.1	126.8	131.8	110.5
	Median	101.8	105.3	105.5	106.7	106.9	108.8	109.1	109.0	94.9
	SD	18.0	22.3	28.4	27.1	22.4	29.4	42.8	53.3	33.5
	Min	66.7	76.7	76.7	76.7	76.7	76.7	76.7	76.7	76.7
	Max	149.5	152.7	194.2	182.5	157.6	199.0	230.3	250.5	155.8
	Lower 95% CI	98.4	104.1	105.4	106.2	102.2	106.0	106.8	99.6	68.9
	Upper 95% CI	112.6	122.9	130.5	130.2	122.1	132.1	146.8	164.0	152.0
Teenager	n	7	7	7	7	7	7	6	4	3
	Total Mean of Means	100.7	101.7	102.8	100.4	99.8	102.9	103.7	93.1	85.7
	Median	100.6	104.5	100.1	101.1	102.1	101.2	103.3	88.4	78.4
	SD	8.8	11.1	6.8	5.8	6.2	10.6	13.7	22.2	34.3
	Min	84.9	82.1	95.6	93.5	90.8	87.6	84.1	72.5	55.6
	Max	114.4	118.0	112.6	108.3	106.8	119.3	125.8	123.1	123.1
	Lower 95% CI	92.5	91.4	96.5	95.0	94.0	93.2	89.3	57.7	0.4
	Upper 95% CI	108.8	111.9	109.0	105.8	105.5	112.7	118.1	128.4	171.0
Child	n	15	15	15	15	14	12	5	2	
	Total Mean of Means	104.7	107.4	115.3	120.3	122.6	123.3	87.8	96.8	
	Median	102.1	98.3	101.4	99.7	109.8	116.3	82.5	96.8	
	SD	10.8	21.7	29.8	36.2	44.0	43.8	17.1	2.0	
	Min	92.1	88.1	83.0	78.1	73.7	69.9	67.4	95.4	
	Max	121.0	159.1	166.2	185.3	213.8	227.6	110.7	98.2	

(a) This interval lasts approx. 6 months, depending on the number of infusions received by the patient during Stage 1.
(b) This interval lasts approx. 6 months, from the first infusion in Stage 2 until 12 months after the first infusion of Subgam in Stage 1.

Certain data have been excluded from the efficacy analysis. See filenote 001, Section 16.1.9, for details of data exclusions from this table

Program: T0364.TEM, Version: 8.2, Datetime: 07FEB07:09:48

Table 14.2.5.6: Mean Subgam dose (mg/kg) received in 6-monthly intervals - by age group

By Age group		Stage 1	Stage 2							
		0 to 6 months(a)	>6 to 12 months(b)	>12 to 18 months	>18 to 24 months	>24 to 30 months	>30 to 36 months	>36 to 42 months	>42 to 48 months	>48 to 54 months
Child	Lower 95% CI	98.7	95.4	98.7	100.3	97.2	95.5	66.7	78.9	
	Upper 95% CI	110.6	119.4	131.8	140.4	148.0	151.1	109.0	114.6	

(a) This interval lasts approx. 6 months, depending on the number of infusions received by the patient during Stage 1.
(b) This interval lasts approx. 6 months, from the first infusion in Stage 2 until 12 months after the first infusion of Subgam in Stage 1.

Certain data have been excluded from the efficacy analysis. See filenote 001, Section 16.1.9, for details of data exclusions from this table

Program: T0364.TEM, Version: 8.2, Datetime: 07FEB07:09:48

Figure 14.2.5.7 'Mean of means' of Subgam dosage (mg/kg) received in 6-monthly intervals – by age group, all patients

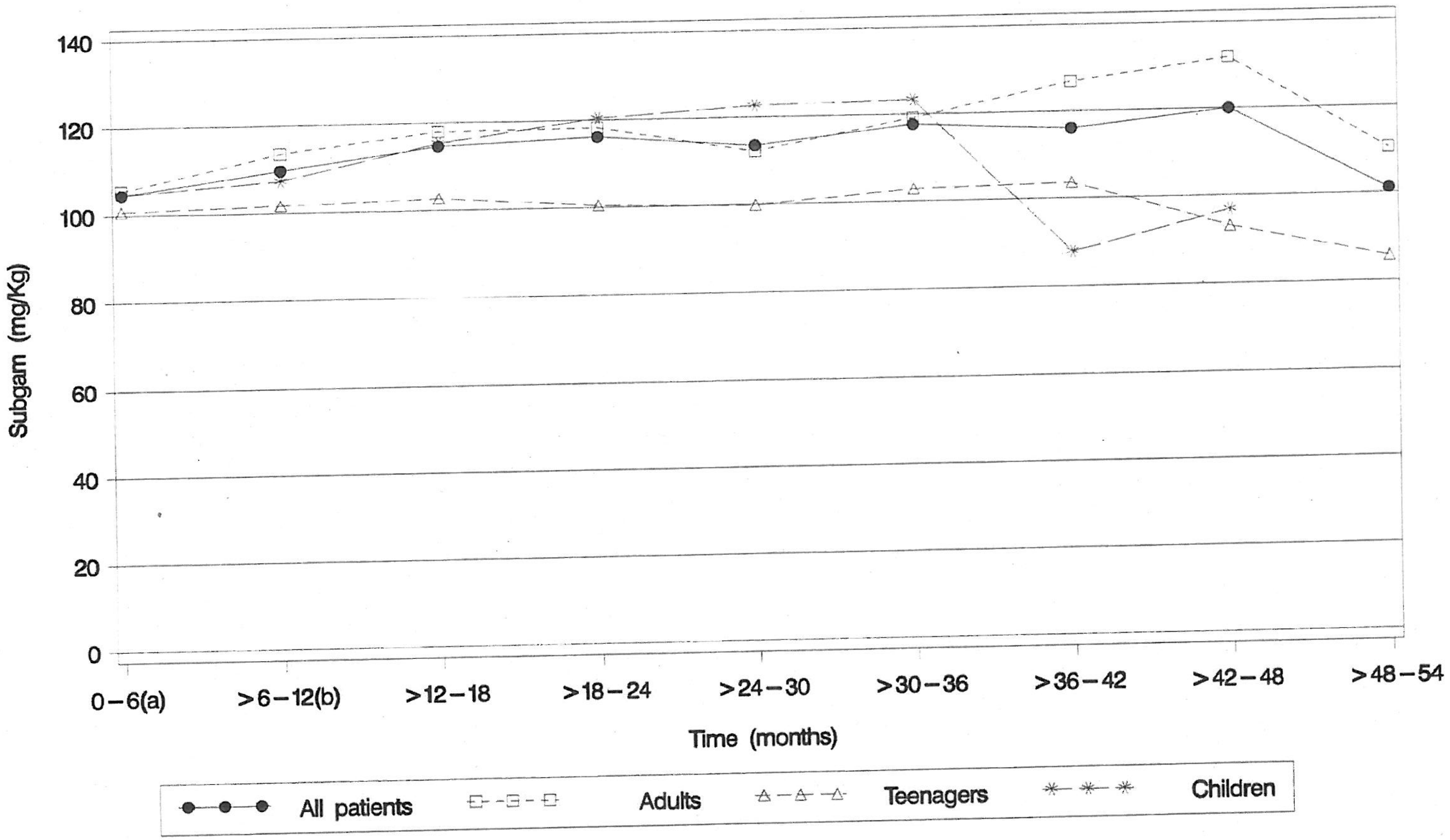

(a) This interval lasts approximately 6 months, depending on the number of infusions received by the patient during Stage 1, and contains post-Subgam data only
(b) This interval lasts approximately 6 months, from the first infusion in Stage 2 until 12 months after the first infusion of Subgam in Stage 1

Certain data have been excluded from the efficacy analysis. See filenote 001, Section 16.1.9, for details of data exclusions from this figure

Program: T0331.SAS, Version: 8.2, Datetime: 07FEB2007:09:47

Table 14.2.5.8: Mean Subgam dose (mg/kg) received in 6-monthly intervals - by prior therapy

By Prior Therapy		Stage 1	Stage 2							
		0 to 6 months(a)	>6 to 12 months(b)	>12 to 18 months	>18 to 24 months	>24 to 30 months	>30 to 36 months	>36 to 42 months	>42 to 48 months	>48 to 54 months
IVIG	n	35	33	31	31	31	29	23	16	7
	Total Mean of Means	105.4	110.2	115.3	118.3	116.6	122.3	123.2	125.1	107.7
	Median	101.8	102.3	102.6	106.2	103.5	109.8	107.8	105.0	94.9
	SD	17.2	21.7	24.5	29.2	32.9	34.9	41.3	50.3	30.6
	Min	66.7	76.7	76.7	76.7	76.7	76.7	76.7	76.7	76.7
	Max	149.5	159.1	166.4	185.3	213.8	227.6	230.3	250.5	155.8
	Lower 95% CI	99.5	102.5	106.3	107.6	104.5	109.0	105.4	98.3	79.4
	Upper 95% CI	111.3	117.9	124.3	129.0	128.7	135.6	141.1	151.9	136.0
SCIG	n	14	13	13	13	12	12	8	3	1
	Total Mean of Means	102.5	108.5	113.1	110.8	105.6	106.1	95.3	92.3	55.6
	Median	101.8	104.4	103.4	99.7	101.5	104.9	102.9	95.4	55.6
	SD	6.4	19.3	32.7	28.9	20.9	22.2	16.5	18.5	
	Min	93.2	88.1	83.0	78.1	73.7	69.9	67.4	72.5	55.6
	Max	117.9	152.7	194.2	182.5	143.4	148.6	110.7	109.0	55.6
	Lower 95% CI	98.8	96.8	93.3	93.3	92.3	92.0	81.5	46.4	.
	Upper 95% CI	106.2	120.1	132.8	128.3	118.9	120.3	109.1	138.2	.

(a) This interval lasts approx. 6 months, depending on the number of infusions received by the patient during Stage 1.
(b) This interval lasts approx. 6 months, from the first infusion in Stage 2 until 12 months after the first infusion of Subgam in Stage 1.

Certain data have been excluded from the efficacy analysis. See filenote 001, Section 16.1.9, for details of data exclusions from this table

Program: T0365.TEM, Version: 8.2, Datetime: 07FEB07:09:48

Figure 14.2.5.9 'Mean of means' of Subgam dosage (mg/kg) received in 6-monthly intervals – by prior therapy, all patients

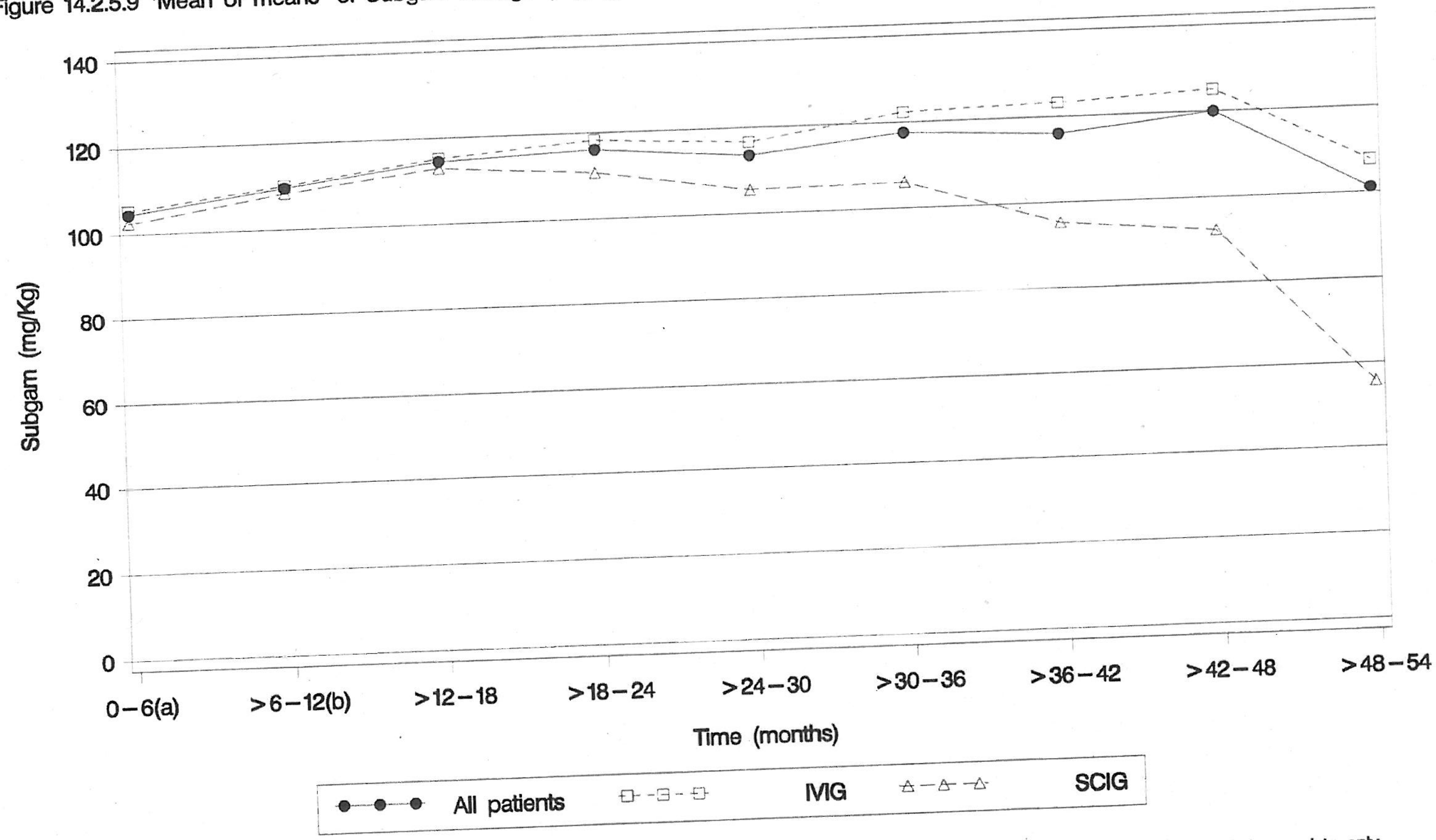

(a) This interval lasts approximately 6 months, depending on the number of infusions received by the patient during Stage 1, and contains post-Subgam data only
(b) This interval lasts approximately 6 months, from the first infusion in Stage 2 until 12 months after the first infusion of Subgam in Stage 1

Certain data have been excluded from the efficacy analysis. See filenote 001, Section 16.1.9, for details of data exclusions from this figure

Program: T0332.SAS, Version: 8.2, Datetime: 07FEB2007:09:47

Table 14.2.6.1: Number of increases and decreases in Subgam dose (ml) across the whole study - all patients

Patient	Number of infusions given	Number of dose increases(a)	Number of dose decreases(b)
01	155	0	0
05	78	0	0
06	201	3	3
07	181	0	0
08	181	3	1
09	22	0	0
10	199	1	0
11	219	8	3
12	43	0	1
13	181	5	0
17	185	1	1
18	179	0	0
21	208	2	1
22	196	4	2
23	177	1	0
24	174	2	0
25	167	3	1
26	122	2	0
27	104	2	0
28	145	0	0
29	194	1	4
30	192	8	7
31	28	0	2
33	144	3	2
34	122	3	3
35	194	0	0

(a) increase > 1ml
(b) decrease > 1ml

Certain data have been excluded from the efficacy analysis. See filenote 001, Section 16.1.9, for details of data exclusions from this table

Program: T0366.TEM, Version: 8.2, Datetime: 07FEB07:09:48

Table 14.2.6.1: Number of increases and decreases in Subgam dose (ml) across the whole study - all patients

Patient	Number of infusions given	Number of dose increases(a)	Number of dose decreases(b)
41	202	0	0
42	98	0	0
43	42	0	0
49	183	2	0
53	139	4	2
54	146	2	0
55	138	6	5
56	146	2	0
57	159	3	0
58	317	154	155
61	107	1	0
65	107	0	0
73	159	3	2
74	165	1	0
75	116	4	2
76	27	0	1
77	162	2	0
78	142	1	0
81	136	3	1
82	138	5	1
83	138	3	0
84	137	3	2
85	119	2	0
86	120	7	5

(a) increase > 1ml
(b) decrease > 1ml

Certain data have been excluded from the efficacy analysis. See filenote 001, Section 16.1.9, for details of data exclusions from this table

Table 14.2.6.1: Number of increases and decreases in Subgam dose (ml) across the whole study - all patients

	Number of infusions given	Number of dose increases(a)	Number of dose decreases(b)
n	48	48	48
Mean	144.1	2.2	1.1
Median	146.0	2.0	0.0
SD	49.7	2.1	1.6
Min	22.0	0.0	0.0
Max	219.0	8.0	7.0
Lower 95% CI	129.7	1.6	0.6
Upper 95% CI	158.6	2.8	1.6

(a) increase > 1ml
(b) decrease > 1ml

Certain data have been excluded from the efficacy analysis. See filenote 001, Section 16.1.9, for details of data exclusions from this table

Program: T0366.TEM, Version: 8.2, Datetime: 07FEB07:09:48

Table 14.2.6.2: Number of increases and decreases in Subgam dose (ml) across the whole study - by diagnosis of PAD

Diagnosis of PAD		Number of infusions given	Number of dose increases(a)	Number of dose decreases(b)
CVID/XLA	n	32	32	32
	Mean	154.2	2.3	1.3
	Median	163.5	2.0	1.0
	SD	48.9	2.2	1.7
	Min	27.0	0.0	0.0
	Max	219.0	8.0	7.0
	Lower 95% CI	136.5	1.5	0.7
	Upper 95% CI	171.8	3.0	1.9
Other	n	16	16	16
	Mean	124.1	2.1	0.7
	Median	138.0	1.5	0.0
	SD	46.4	2.1	1.4
	Min	22.0	0.0	0.0
	Max	185.0	6.0	5.0
	Lower 95% CI	99.3	1.0	-0.0
	Upper 95% CI	148.8	3.2	1.4

(a) increase > 1ml
(b) decrease > 1ml

Certain data have been excluded from the efficacy analysis. See filenote 001, Section 16.1.9, for details of data exclusions from this table

Program: T0367.TEM, Version: 8.2, Datetime: 07FEB07:09:48

Table 14.2.6.3: Number of increases and decreases in Subgam dose (ml) across the whole study - by age group

Age group		Number of infusions given	Number of dose increases(a)	Number of dose decreases(b)
Adult	n	26	26	26
	Mean	144.0	1.8	1.0
	Median	163.5	1.0	1.0
	SD	61.0	1.9	1.2
	Min	22.0	0.0	0.0
	Max	219.0	8.0	4.0
	Lower 95% CI	119.4	1.0	0.6
	Upper 95% CI	168.6	2.5	1.5
Teenager	n	7	7	7
	Mean	171.3	3.4	2.4
	Median	183.0	3.0	2.0
	SD	29.8	2.7	2.8
	Min	137.0	0.0	0.0
	Max	202.0	8.0	7.0
	Lower 95% CI	143.8	0.9	-0.1
	Upper 95% CI	198.8	5.9	5.0
Child	n	15	15	15
	Mean	131.7	2.4	0.5
	Median	136.0	2.0	0.0
	SD	27.4	2.1	1.3
	Min	78.0	0.0	0.0
	Max	181.0	7.0	5.0
	Lower 95% CI	116.6	1.2	-0.2
	Upper 95% CI	146.9	3.6	1.3

(a) increase > 1ml
(b) decrease > 1ml

Certain data have been excluded from the efficacy analysis. See filenote 001, Section 16.1.9, for details of data exclusions from this table

Program: T0368.TEM, Version: 8.2, Datetime: 07FEB07:09:48

Table 14.2.6.4: Number of increases and decreases in Subgam dose (ml) across the whole study - by prior therapy

Prior therapy		Number of infusions given	Number of dose increases(a)	Number of dose decreases(b)
IVIG	n	34	34	34
	Mean	147.1	2.3	1.2
	Median	146.0	2.0	0.0
	SD	51.9	2.3	1.8
	Min	22.0	0.0	0.0
	Max	219.0	8.0	7.0
	Lower 95% CI	129.0	1.5	0.5
	Upper 95% CI	165.2	3.1	1.8
SCIG	n	14	14	14
	Mean	137.0	2.0	0.9
	Median	150.0	2.5	0.5
	SD	45.1	1.5	1.0
	Min	27.0	0.0	0.0
	Max	201.0	4.0	3.0
	Lower 95% CI	111.0	1.1	0.3
	Upper 95% CI	163.0	2.9	1.5

(a) increase > 1ml
(b) decrease > 1ml

Certain data have been excluded from the efficacy analysis. See filenote 001, Section 16.1.9, for details of data exclusions from this table

Program: T0369.TEM, Version: 8.2, Datetime: 07FEB07:09:48

Table 14.2.6.5: Min and max Subgam dose (as a % of the starting dose) - all patients

Patient	Min dose as a % of starting dose (mg/kg)		Max dose as a % of starting dose (mg/kg)	
	Inf 4 - EOP1	EOP1 - EOP2	Inf 4 - EOP1	EOP1 - EOP2
01	95.0	69.8	100.0	94.8
05	93.5	94.2	107.3	104.6
06	96.4	55.6	100.0	110.7
07	98.6	98.5	100.0	98.5
08	100.0	107.2	129.5	192.6
09	100.0	.	101.0	.
10	99.9	99.9	100.0	156.6
11	93.7	61.6	100.0	258.3
12	90.9	89.8	100.0	92.9
13	87.2	83.9	100.0	106.6
17	100.0	91.8	101.4	101.4
18	100.0	104.4	104.4	107.2
21	100.0	89.7	134.0	134.9
22	100.0	110.3	130.4	233.5
23	100.0	143.6	143.6	148.2
24	100.0	133.6	137.7	151.4
25	90.8	81.8	100.0	97.4
26	90.9	82.7	100.7	102.9
27	96.1	120.0	132.9	159.6
28	98.4	77.8	100.0	98.2
29	70.7	74.5	100.0	74.5
30	59.7	58.0	100.0	135.3
31	63.4	.	101.0	.
33	89.0	56.6	113.7	135.9
34	58.6	37.4	100.0	96.2

EOP1 = End of Stage 1; EOP2 = End of Stage 2

Certain data have been excluded from the efficacy analysis. See filenote 001, Section 16.1.9, for details of data exclusions from this table
Certain data have been excluded from the efficacy analysis. See filenote 002, Section 16.1.9, for details of data exclusions from this table

Program: T0143.TEM, Version: 8.2, Datetime: 07FEB07:09:48

Table 14.2.6.5: Min and max Subgam dose (as a % of the starting dose) - all patients

Patient	Min dose as a % of starting dose (mg/kg)		Max dose as a % of starting dose (mg/kg)	
	Inf 4 - EOP1	EOP1 - EOP2	Inf 4 - EOP1	EOP1 - EOP2
35	98.2	98.2	100.0	98.5
41	100.0	74.1	101.0	101.1
42	95.4	94.9	100.0	101.5
43	96.9	96.7	100.0	99.6
49	95.9	91.9	111.5	105.3
53	100.0	101.8	158.4	152.6
54	100.0	95.3	101.3	101.2
55	96.6	80.9	147.3	135.5
56	98.6	98.7	101.3	101.6
57	99.1	134.0	146.8	169.3
58	90.9	81.1	102.1	102.1
61	90.4	79.0	100.0	97.5
65	91.1	91.1	100.0	91.1
73	79.4	105.8	105.9	106.5
74	99.3	99.5	107.4	105.1
75	99.6	84.0	109.6	109.3
76	99.2	.	106.5	.
77	100.0	144.4	144.4	172.6
78	100.0	143.1	146.6	144.0
81	100.0	143.8	153.5	278.1
82	98.4	116.9	158.3	227.0
83	93.2	101.9	132.2	179.6
84	95.9	54.1	127.6	146.2
85	93.8	100.9	124.7	125.5
86	58.7	41.8	100.0	120.4

EOP1 = End of Stage 1; EOP2 = End of Stage 2

Certain data have been excluded from the efficacy analysis. See filenote 001, Section 16.1.9, for details of data exclusions from this table
Certain data have been excluded from the efficacy analysis. See filenote 002, Section 16.1.9, for details of data exclusions from this table

Program: T0143.TEM, Version: 8.2, Datetime: 07FEB07:09:48

Table 14.2.6.5: Min and max Subgam dose (as a % of the starting dose) - all patients

	Min dose as a % of starting dose (mg/kg)		Max dose as a % of starting dose (mg/kg)	
	Inf 4 - EOP1	EOP1 - EOP2	Inf 4 - EOP1	EOP1 - EOP2
n	48	45	48	45
Mean	92.9	93.3	115.0	132.4
Median	97.6	94.2	102.9	109.3
SD	11.50	26.34	19.45	46.28
Min	58.6	37.4	100.0	74.5
Max	100.0	144.4	158.4	278.1
Lower 95% CI	89.52	85.43	109.39	118.53
Upper 95% CI	96.20	101.26	120.69	146.35

EOP1 = End of Stage 1; EOP2 = End of Stage 2

Certain data have been excluded from the efficacy analysis. See filenote 001, Section 16.1.9, for details of data exclusions from this table
Certain data have been excluded from the efficacy analysis. See filenote 002, Section 16.1.9, for details of data exclusions from this table

Program: T0143.TEM, Version: 8.2, Datetime: 07FEB07:09:48

Table 14.2.6.6: Min and max Subgam dose (as a % of the starting dose) - completers(a)

	Min dose as a % of starting dose (mg/kg)		Max dose as a % of starting dose (mg/kg)	
	Inf 4 - EOP1	EOP1 - EOP2	Inf 4 - EOP1	EOP1 - EOP2
n	31	31	31	31
Mean	92.0	89.4	116.3	135.9
Median	98.2	91.9	105.9	120.4
SD	12.79	27.15	20.37	48.52
Min	58.6	37.4	100.0	74.5
Max	100.0	144.4	158.4	278.1
Lower 95% CI	87.30	79.44	108.87	118.12
Upper 95% CI	96.69	99.36	123.81	153.71

EOP1 = End of Stage 1; EOP2 = End of Stage 2
(a) Completers are defined in Section 5 of the statistical analysis plan

Certain data have been excluded from the efficacy analysis. See filenote 001, Section 16.1.9, for details of data exclusions from this table
Certain data have been excluded from the efficacy analysis. See filenote 002, Section 16.1.9, for details of data exclusions from this table

Program: T0144.TEM, Version: 8.2, Datetime: 07FEB07:09:48

Table 14.2.6.7: Min and max Subgam dose (as a % of the starting dose) - by diagnosis of PAD

Diagnosis of PAD		Min dose as a % of starting dose (mg/kg)		Max dose as a % of starting dose (mg/kg)	
		Inf 4 - EOP1	EOP1 - EOP2	Inf 4 - EOP1	EOP1 - EOP2
CVID/XLA	n	32	30	32	30
	Mean	91.2	91.2	112.6	134.1
	Median	98.3	91.5	101.3	108.9
	SD	13.59	29.55	17.49	50.47
	Min	58.6	37.4	100.0	74.5
	Max	100.0	144.4	153.5	278.1
	Lower 95% CI	86.28	80.21	106.27	115.28
	Upper 95% CI	96.08	102.28	118.88	152.97
Other	n	16	15	16	15
	Mean	96.2	97.6	120.0	129.1
	Median	96.8	96.7	108.4	109.3
	SD	3.82	18.54	22.67	37.92
	Min	87.2	69.8	100.0	94.8
	Max	100.0	143.1	158.4	227.0
	Lower 95% CI	94.18	87.30	107.89	108.08
	Upper 95% CI	98.25	107.83	132.05	150.07

EOP1 = End of Stage 1; EOP2 = End of Stage 2

Certain data have been excluded from the efficacy analysis. See filenote 001, Section 16.1.9, for details of data exclusions from this table
Certain data have been excluded from the efficacy analysis. See filenote 002, Section 16.1.9, for details of data exclusions from this table

Program: T0145.TEM, Version: 8.2, Datetime: 07FEB07:09:48

Table 14.2.6.8: Min and max Subgam dose (as a % of the starting dose) - by age group

Age group		Min dose as a % of starting dose (mg/kg)		Max dose as a % of starting dose (mg/kg)	
		Inf 4 - EOP1	EOP1 - EOP2	Inf 4 - EOP1	EOP1 - EOP2
Adult	n	26	23	26	23
	Mean	93.7	98.6	114.5	133.5
	Median	99.8	98.5	105.2	109.3
	SD	11.97	26.57	18.77	46.29
	Min	58.6	37.4	100.0	74.5
	Max	100.0	144.4	158.4	258.3
	Lower 95% CI	88.91	87.12	106.91	113.53
	Upper 95% CI	98.58	110.09	122.07	153.56
Teenager	n	7	7	7	7
	Mean	91.9	73.3	112.7	119.4
	Median	96.4	74.1	101.3	110.7
	SD	14.29	18.11	18.30	18.96
	Min	59.7	54.1	100.0	101.1
	Max	100.0	98.7	147.3	146.2
	Lower 95% CI	78.65	56.58	95.75	101.85
	Upper 95% CI	105.08	90.07	129.59	136.92
Child	n	15	15	15	15
	Mean	91.8	94.6	117.1	136.8
	Median	93.5	91.1	100.7	106.6
	SD	9.87	26.15	22.13	55.71
	Min	58.7	41.8	100.0	91.1
	Max	100.0	143.8	158.3	278.1
	Lower 95% CI	86.31	80.15	104.84	105.98

EOP1 = End of Stage 1; EOP2 = End of Stage 2

Certain data have been excluded from the efficacy analysis. See filenote 001, Section 16.1.9, for details of data exclusions from this table
Certain data have been excluded from the efficacy analysis. See filenote 002, Section 16.1.9, for details of data exclusions from this table

Program: T0146.TEM, Version: 8.2, Datetime: 07FEB07:09:48

Table 14.2.6.8: Min and max Subgam dose (as a % of the starting dose) - by age group

Age group		Min dose as a % of starting dose (mg/kg)		Max dose as a % of starting dose (mg/kg)	
		Inf 4 - EOP1	EOP1 - EOP2	Inf 4 - EOP1	EOP1 - EOP2
Child	Upper 95% CI	97.25	109.12	129.34	167.68

EOP1 = End of Stage 1; EOP2 = End of Stage 2

Certain data have been excluded from the efficacy analysis. See filenote 001, Section 16.1.9, for details of data exclusions from this table
Certain data have been excluded from the efficacy analysis. See filenote 002, Section 16.1.9, for details of data exclusions from this table

Program: T0146.TEM, Version: 8.2, Datetime: 07FEB07:09:48

Table 14.2.6.9: Min and max Subgam dose (as a % of the starting dose) - by prior therapy

Prior therapy		Min dose as a % of starting dose (mg/kg)		Max dose as a % of starting dose (mg/kg)	
		Inf 4 - EOP1	EOP1 - EOP2	Inf 4 - EOP1	EOP1 - EOP2
IVIG	n	34	32	34	32
	Mean	91.7	93.3	115.2	136.1
	Median	97.6	93.6	101.3	123.0
	SD	13.09	28.49	19.80	50.74
	Min	58.6	37.4	100.0	74.5
	Max	100.0	144.4	158.3	278.1
	Lower 95% CI	87.18	83.06	108.29	117.84
	Upper 95% CI	96.32	103.60	122.11	154.43
SCIG	n	14	13	14	13
	Mean	95.6	93.4	114.6	123.3
	Median	97.4	94.2	106.9	106.5
	SD	5.65	21.16	19.29	32.85
	Min	79.4	55.6	100.0	94.8
	Max	100.0	134.0	158.4	192.6
	Lower 95% CI	92.29	80.61	103.51	103.50
	Upper 95% CI	98.81	106.18	125.78	143.20

EOP1 = End of Stage 1; EOP2 = End of Stage 2

Certain data have been excluded from the efficacy analysis. See filenote 001, Section 16.1.9, for details of data exclusions from this table
Certain data have been excluded from the efficacy analysis. See filenote 002, Section 16.1.9, for details of data exclusions from this table

Program: T0147.TEM, Version: 8.2, Datetime: 07FEB07:09:48

Table / Figure Number	Table / Figure Name
**14.3**	**Safety Data**
**14.3.1**	**Adverse Events (Excluding Infections)**
Table 14.3.1.1	Summary of adverse events (excluding infections)
Table 14.3.1.2	Adverse events (excluding infections) by system organ class and relationship to study treatment – Pre-Subgam (Pre-study–Infusion 3)
Table 14.3.1.3	Adverse events (excluding infections) by system organ class and relationship to study treatment– Post-Subgam (Infusion 4-Last Subgam Infusion)
Table 14.3.1.4	Adverse events (excluding infections) by system organ class, preferred term, severity and relationship to treatment - Pre-Subgam (Pre-study–Infusion 3)
Table 14.3.1.5	Adverse events (excluding infections) by system organ class, preferred term, severity and relationship to treatment - Post-Subgam (Infusion 4-EOS)
Table 14.3.1.6	Serious adverse events by system organ class, preferred term, severity and relationship to treatment (excluding infections) – Pre-Subgam (Pre-study–Infusion 3)
Table 14.3.1.7	Serious adverse events by system organ class, preferred term, severity and relationship to treatment (excluding infections) – Post-Subgam (Infusion 4-EOS)
Table 14.3.1.8	Adverse events (excluding infections) related to Subgam, by system organ class and preferred term – Post-Subgam (Infusion 4-EOS)
**14.3.2**	**Deaths and Other Serious and Significant Adverse Events (Excluding Infections)**
**14.3.3**	**Infusion Site Reactions**
Table 14.3.3.1	Summary of infusion site reactions in 6-monthly intervals – by diagnosis of PAD, age group and prior therapy
Figure 14.3.3.2	Summary of infusion site reactions in 6-monthly intervals – by diagnosis of PAD, all patients
Figure 14.3.3.3	Summary of infusion site reactions in 6-monthly intervals – by age group , all patients
Figure 14.3.3.4	Summary of infusion site reactions in 6-monthly intervals – by prior therapy, all patients
Figure 14.3.3.5	Summary of infusion site reactions by preferred term and severity – Pre-Subgam (Pre-study – Infusion 3) - all patients
Figure 14.3.3.6	Summary of infusion site reactions by preferred term and severity – Post-Subgam (Infusion 4-Last Subgam Infusion) - all patients
Table 14.3.3.7	Summary of infusion site reactions by preferred term and severity – all patients
Table 14.3.3.7a	0 to 6 months Post-Subgam
Table 14.3.3.7b	>6 to12 months Post-Subgam
Table 14.3.3.7c	>12 to 18 months Post-Subgam
Table 14.3.3.7d	>18 to 24 months Post-Subgam
Table 14.3.3.7e	>24 to 30 months Post-Subgam
Table 14.3.3.7f	>30 to 36 months Post-Subgam

Table 14.3.3.7g	>36 to 42 months Post-Subgam
Table 14.3.3.7h	>42 to 48 months Post-Subgam
Table 14.3.3.7i	>48 to 54 months Post-Subgam
Table 14.3.3.8	Summary of infusion site reactions by preferred term and severity – by diagnosis of PAD
Table 14.3.3.8a	0 to 6 months Post-Subgam
Table 14.3.3.8b	>6 to12 months Post-Subgam
Table 14.3.3.8c	>12 to 18 months Post-Subgam
Table 14.3.3.8d	>18 to 24 months Post-Subgam
Table 14.3.3.8e	>24 to 30 months Post-Subgam
Table 14.3.3.8f	>30 to 36 months Post-Subgam
Table 14.3.3.8g	>36 to 42 months Post-Subgam
Table 14.3.3.8h	>42 to 48 months Post-Subgam
Table 14.3.3.8i	>48 to 54 months Post-Subgam
Table 14.3.3.9	Summary of infusion site reactions by preferred term and severity – by age group
Table 14.3.3.9a	0 to 6 months Post-Subgam
Table 14.3.3.9b	>6 to12 months Post-Subgam
Table 14.3.3.9c	>12 to 18 months Post-Subgam
Table 14.3.3.9d	>18 to 24 months Post-Subgam
Table 14.3.3.9e	>24 to 30 months Post-Subgam
Table 14.3.3.9f	>30 to 36 months Post-Subgam
Table 14.3.3.9g	>36 to 42 months Post-Subgam
Table 14.3.3.9h	>42 to 48 months Post-Subgam
Table 14.3.3.9i	>48 to 54 months Post-Subgam
Table 14.3.3.9	Summary of infusion site reactions by preferred term and severity – by prior therapy
Table 14.3.3.10a	0 to 6 months Post-Subgam
Table 14.3.3.10b	>6 to12 months Post-Subgam
Table 14.3.3.10c	>12 to 18 months Post-Subgam
Table 14.3.3.10d	>18 to 24 months Post-Subgam
Table 14.3.3.10e	>24 to 30 months Post-Subgam
Table 14.3.3.10f	>30 to 36 months Post-Subgam
Table 14.3.3.10g	>36 to 42 months Post-Subgam
Table 14.3.3.10h	>42 to 48 months Post-Subgam
Table 14.3.3.10i	>48 to 54 months Post-Subgam
**14.3.4**	**Laboratory Data**
Table 14.3.4.1	Summary statistics for haematology parameters
Table 14.3.4.2	Summary statistics for biochemistry parameters
Table 14.3.4.3	Summary statistics for ALT (U/L)
Table 14.3.4.4	Summary statistics for AST (U/L)
Table 14.3.4.5	Pre-Subgam value versus end of Stage 1 value for ALT (U/L)
Table 14.3.4.6	Pre-Subgam value versus end of Stage 1 value for AST (U/L)
Table 14.3.4.7	Pre-Subgam value versus end of study / withdrawal value for HbsAg
Table 14.3.4.8	Pre-Subgam value versus end of study / withdrawal value for HCV
Table 14.3.4.9	Pre-Subgam value versus end of study value for Parvovirus B19 (PCR)

**14.3.5**	**Vital Signs**
Table 14.3.5.1	Systolic blood pressure – summary statistics for Stage 1 - All infusions, mmHg
Table 14.3.5.2	Systolic blood pressure – summary statistics for Stage 1 - Infusions 1-3 (pre-Subgam), mmHg
Table 14.3.5.3	Systolic blood pressure – summary statistics for Stage 1 - Infusions 4–30 (post-Subgam), mmHg
Table 14.3.5.4	Systolic blood pressure – summary statistics for Stage 2 (post-Subgam), mmHg
Table 14.3.5.5	Systolic blood pressure – summary statistics for pharmacokinetic assessments, mmHg
Table 14.3.5.6	Diastolic blood pressure – summary statistics for Stage 1 - All infusions, mmHg
Table 14.3.5.7	Diastolic blood pressure – summary statistics for Stage 1 - Infusions 1-3 (pre-Subgam), mmHg
Table 14.3.5.8	Diastolic blood pressure – summary statistics for Stage 1 - Infusions 4–30 (post-Subgam), mmHg
Table 14.3.5.9	Diastolic blood pressure – summary statistics for Stage 2 (post-Subgam), mmHg
Table 14.3.5.10	Diastolic blood pressure – summary statistics for pharmacokinetic assessments, mmHg
Table 14.3.5.11	Pulse – summary statistics for Stage 1 - All infusions, beats per minute
Table 14.3.5.12	Pulse – summary statistics for Stage 1 - Infusions 1-3 (pre-Subgam), beats per minute
Table 14.3.5.13	Pulse – summary statistics for Stage 1 - Infusions 4–30 (post-Subgam), beats per minute
Table 14.3.5.14	Pulse – summary statistics for Stage 2 (post-Subgam), beats per minute
Table 14.3.5.15	Pulse – summary statistics for pharmacokinetic assessments, beats per minute
Table 14.3.5.16	Respiration rate – summary statistics for Stage 1 - All infusions, breaths per minute
Table 14.3.5.17	Respiration rate – summary statistics for Stage 1 - Infusions 1-3 (pre-Subgam), breaths per minute
Table 14.3.5.18	Respiration rate – summary statistics for Stage 1 - Infusions 4–30 (post-Subgam), breaths per minute
Table 14.3.5.19	Respiration rate – summary statistics for Stage 2 (post-Subgam), breaths per minute
Table 14.3.5.20	Respiration rate – summary statistics for pharmacokinetic assessments, breaths per minute
Table 14.3.5.21	Temperature – summary statistics for Stage 1 – All infusions, °C
Table 14.3.5.22	Temperature – summary statistics for Stage 1 - Infusions 1–3 (pre-Subgam), °C
Table 14.3.5.23	Temperature – summary statistics for Stage 1 - Infusions 4–30 (post-Subgam), °C
Table 14.3.5.26	Temperature – summary statistics for pharmacokinetic assessments, °C
Table 14.3.5.27	Summary of pre-infusion body temperature >37°C in Stage 1

Table 14.3.5.28	Summary of post-infusion body temperature >37°C in Stage 1
Table 14.3.5.29	Number of reported body temperature elevations of >37°C in Stage 1
Table 14.3.5.30	Number of adverse events reported with preferred term of 'pyrexia' by relatedness to study drug, diagnosis, age group and prior therapy
Table 14.3.5.31	Number of reported temperature elevations of >37°C with and without corresponding adverse events with preferred term of 'pyrexia' by relatedness to study drug, diagnosis, age group and prior therapy

Table 14.3.1.1: Summary of adverse events (excluding infections)

	Mean (range) duration of treatment (days)	Number of Adverse Events	Relationship to SCIG/IVIG		
			NR	R	NS
Pre-Subgam (Pre-study - Infusion 3)	38.6 (7- 65)	61 (28)	59 (27)	2 (2)	0
Post-Subgam (Infusion 4 - EOS)	1107.6 (217-1563)	1490 (50)	1345 (50)	144 (30)	1 (1)

NR = not related; R = related; NS = not specified.

Program: T0045.SAS, Version: 8.2, Datetime: 07FEB07:09:48

Table 14.3.1.2: Adverse events (excluding Infections) by system organ class and relationship to study treatment - Pre-Subgam (Pre-study-Infusion 3)
Number of events (number of patients)

System Organ Class	n	Relationship to SCIG/IVIG NR	R	NS
Ear and labyrinth disorders	3 (3)	3 (3)	0	
Gastrointestinal disorders	10 (8)	9 (7)	1 (1)	
General disorders and administration site conditions	7 (5)	7 (5)	0	
Injury, poisoning and procedural complications	2 (2)	2 (2)	0	
Investigations	6 (5)	6 (5)	0	
Metabolism and nutrition disorders	1 (1)	1 (1)	0	
Musculoskeletal and connective tissue disorders	4 (3)	4 (3)	0	
Nervous system disorders	7 (5)	7 (5)	0	
Psychiatric disorders	3 (3)	3 (3)	0	
Respiratory, thoracic and mediastinal disorders	13 (12)	13 (12)	0	
Skin and subcutaneous tissue disorders	3 (3)	2 (2)	1 (1)	
Vascular disorders	2 (2)	2 (2)	0	
All Disorders	61 (28)	59 (27)	2 (2)	
Infusion Site Reactions	4 (2)	4 (2)	0	
All Other Events	57 (27)	55 (26)	2 (2)	

NR = not related; R = related; NS = not specified.

Program: T0046.SAS, Version: 8.2, Datetime: 07FEB07:09:48

Table 14.3.1.3: Adverse events (excluding Infections) by system organ class and relationship to study treatment- Post-Subgam (Infusion 4-Last Subg
Number of events (number of patients)

System Organ Class	n	Relationship to SCIG/IVIG NR	R	NS
Blood and lymphatic system disorders	6 (6)	6 (6)	0	0
Cardiac disorders	3 (3)	3 (3)	0	0
Congenital, familial and genetic disorders	1 (1)	1 (1)	0	0
Ear and labyrinth disorders	21 (11)	21 (11)	0	0
Endocrine disorders	1 (1)	1 (1)	0	0
Eye disorders	9 (7)	9 (7)	0	0
Gastrointestinal disorders	263 (43)	250 (41)	13 (7)	0
General disorders and administration site conditions	255 (44)	166 (39)	89 (25)	0
Immune system disorders	8 (7)	8 (7)	0	0
Injury, poisoning and procedural complications	24 (17)	24 (17)	0	0
Investigations	48 (19)	45 (19)	3 (2)	0
Metabolism and nutrition disorders	5 (4)	5 (4)	0	0
Musculoskeletal and connective tissue disorders	123 (28)	118 (28)	5 (5)	0
Nervous system disorders	188 (32)	173 (29)	15 (12)	0
Pregnancy, puerperium and perinatal conditions	4 (1)	4 (1)	0	0
Psychiatric disorders	16 (7)	15 (7)	1 (1)	0
Renal and urinary disorders	7 (4)	7 (4)	0	0
Reproductive system and breast disorders	1 (1)	1 (1)	0	0
Respiratory, thoracic and mediastinal disorders	410 (45)	407 (45)	2 (2)	1 (1)
Skin and subcutaneous tissue disorders	76 (27)	62 (26)	14 (8)	0
Surgical and medical procedures	7 (5)	7 (5)	0	0
Vascular disorders	14 (9)	12 (9)	2 (1)	0
All Disorders	1490 (50)	1345 (50)	144 (30)	1 (1)
Infusion Site Reactions	82 (25)	6 (6)	76 (21)	0
All Other Events	1408 (50)	1339 (50)	68 (23)	1 (1)

NR = not related; R = related; NS = not specified.

Program: T0047.SAS, Version: 8.2, Datetime: 07FEB07:09:48

Table 14.3.1.4: Adverse events (excluding infections) by system organ class, preferred term, severity and relationship to treatment
Pre-Subgam (Pre-study-Infusion 3)

			Severity and relationship to treatment															
			Mild				Moderate				Severe				Not known			
System Organ Class	Preferred term	n	NR	R	NS	Tot	NR	R	NS	Tot	NR	R	NS	Tot	NR	R	NS	Tot
Ear and labyrinth disorders	Ear pain	1	1	0	0	1	0	0	0	0	0	0	0	0	0	0	0	0
	Sensation of block in ear	1	1	0	0	1	0	0	0	0	0	0	0	0	0	0	0	0
	Vertigo positional	1	1	0	0	1	0	0	0	0	0	0	0	0	0	0	0	0
	Total reports	3	3	0	0	3	0	0	0	0	0	0	0	0	0	0	0	0
	Total patients	3	3	0	0	3	0	0	0	0	0	0	0	0	0	0	0	0
Gastrointestinal disorders	Diarrhoea NOS	3	3	0	0	3	0	0	0	0	0	0	0	0	0	0	0	0
	Haematemesis	1	0	0	0	0	0	0	0	0	1	0	0	1	0	0	0	0
	Nausea	1	0	1	0	1	0	0	0	0	0	0	0	0	0	0	0	0
	Teething	1	1	0	0	1	0	0	0	0	0	0	0	0	0	0	0	0
	Vomiting NOS	4	3	0	0	3	1	0	0	1	0	0	0	0	0	0	0	0
	Total reports	10	7	1	0	8	1	0	0	1	1	0	0	1	0	0	0	0
	Total patients	8	5	1	0	6	1	0	0	1	1	0	0	1	0	0	0	0

NR = not related; R = related; NS = not specified; Tot = total.

Program: T0230.TEM, Version: 8.2, Datetime: 07FEB07:09:48

Table 14.3.1.4: Adverse events (excluding infections) by system organ class, preferred term, severity and relationship to treatment
Pre-Subgam (Pre-study-Infusion 3)

			Severity and relationship to treatment															
			Mild				Moderate				Severe				Not known			
System Organ Class	Preferred term	n	NR	R	NS	Tot	NR	R	NS	Tot	NR	R	NS	Tot	NR	R	NS	Tot
General disorders and administration site conditions	Cannula site reaction	1	0	0	0	0	1	0	0	1	0	0	0	0	0	0	0	0
	Infusion site pain	4	1	0	0	1	3	0	0	3	0	0	0	0	0	0	0	0
	Lethargy	1	1	0	0	1	0	0	0	0	0	0	0	0	0	0	0	0
	Rigors	1	1	0	0	1	0	0	0	0	0	0	0	0	0	0	0	0
	Total reports	7	3	0	0	3	4	0	0	4	0	0	0	0	0	0	0	0
	Total patients	5	3	0	0	3	2	0	0	2	0	0	0	0	0	0	0	0
Injury, poisoning and procedural complications	Back injury NOS	1	0	0	0	0	0	0	0	0	1	0	0	1	0	0	0	0
	Limb injury NOS	1	1	0	0	1	0	0	0	0	0	0	0	0	0	0	0	0
	Total reports	2	1	0	0	1	0	0	0	0	1	0	0	1	0	0	0	0
	Total patients	2	1	0	0	1	0	0	0	0	1	0	0	1	0	0	0	0
Investigations	Body temperature increased	4	3	0	0	3	1	0	0	1	0	0	0	0	0	0	0	0
	Platelet count decreased	1	1	0	0	1	0	0	0	0	0	0	0	0	0	0	0	0
	Sputum increased	1	1	0	0	1	0	0	0	0	0	0	0	0	0	0	0	0
	Total reports	6	5	0	0	5	1	0	0	1	0	0	0	0	0	0	0	0
	Total patients	5	4	0	0	4	1	0	0	1	0	0	0	0	0	0	0	0

NR = not related; R = related; NS = not specified; Tot = total.

Program: T0230.TEM, Version: 8.2, Datetime: 07FEB07:09:48

Table 14.3.1.4: Adverse events (excluding infections) by system organ class, preferred term, severity and relationship to treatment
Pre-Subgam (Pre-study-Infusion 3)

System Organ Class	Preferred term	n	Mild				Moderate				Severe				Not known			
			NR	R	NS	Tot	NR	R	NS	Tot	NR	R	NS	Tot	NR	R	NS	Tot
Metabolism and nutrition disorders	Anorexia	1	1	0	0	1	0	0	0	0	0	0	0	0	0	0	0	0
	Total reports	1	1	0	0	1	0	0	0	0	0	0	0	0	0	0	0	0
	Total patients	1	1	0	0	1	0	0	0	0	0	0	0	0	0	0	0	0
Musculoskeletal and connective tissue disorders	Arthralgia	1	0	0	0	0	0	0	0	0	1	0	0	1	0	0	0	0
	Back pain	1	0	0	0	0	1	0	0	1	0	0	0	0	0	0	0	0
	Pain in limb	1	0	0	0	0	0	0	0	0	1	0	0	1	0	0	0	0
	Tendonitis	1	1	0	0	1	0	0	0	0	0	0	0	0	0	0	0	0
	Total reports	4	1	0	0	1	1	0	0	1	2	0	0	2	0	0	0	0
	Total patients	3	1	0	0	1	1	0	0	1	1	0	0	1	0	0	0	0
Nervous system disorders	Dizziness	2	2	0	0	2	0	0	0	0	0	0	0	0	0	0	0	0
	Headache	5	3	0	0	3	2	0	0	2	0	0	0	0	0	0	0	0
	Total reports	7	5	0	0	5	2	0	0	2	0	0	0	0	0	0	0	0
	Total patients	5	4	0	0	4	1	0	0	1	0	0	0	0	0	0	0	0

NR = not related; R = related; NS = not specified; Tot = total.

Program: T0230.TEM, Version: 8.2, Datetime: 07FEB07:09:48

Table 14.3.1.4: Adverse events (excluding infections) by system organ class, preferred term, severity and relationship to treatment
Pre-Subgam (Pre-study-Infusion 3)

			Severity and relationship to treatment															
			Mild				Moderate				Severe				Not known			
System Organ Class	Preferred term	n	NR	R	NS	Tot	NR	R	NS	Tot	NR	R	NS	Tot	NR	R	NS	Tot
Psychiatric disorders	Anxiety	1	0	0	0	0	1	0	0	1	0	0	0	0	0	0	0	0
	Depression	1	0	0	0	0	0	0	0	0	1	0	0	1	0	0	0	0
	Listless	1	1	0	0	1	0	0	0	0	0	0	0	0	0	0	0	0
	Total reports	3	1	0	0	1	1	0	0	1	1	0	0	1	0	0	0	0
	Total patients	3	1	0	0	1	1	0	0	1	1	0	0	1	0	0	0	0
Respiratory, thoracic and mediastinal disorders	Asthma NOS	1	1	0	0	1	0	0	0	0	0	0	0	0	0	0	0	0
	Cough	5	3	0	0	3	2	0	0	2	0	0	0	0	0	0	0	0
	Nasal congestion	2	2	0	0	2	0	0	0	0	0	0	0	0	0	0	0	0
	Pharyngitis	1	1	0	0	1	0	0	0	0	0	0	0	0	0	0	0	0
	Rhinitis NOS	1	1	0	0	1	0	0	0	0	0	0	0	0	0	0	0	0
	Sinus pain	2	1	0	0	1	1	0	0	1	0	0	0	0	0	0	0	0
	Wheezing	1	1	0	0	1	0	0	0	0	0	0	0	0	0	0	0	0
	Total reports	13	10	0	0	10	3	0	0	3	0	0	0	0	0	0	0	0
	Total patients	12	10	0	0	10	3	0	0	3	0	0	0	0	0	0	0	0

NR = not related; R = related; NS = not specified; Tot = total.

Program: T0230.TEM, Version: 8.2, Datetime: 07FEB07:09:48

Table 14.3.1.4: Adverse events (excluding infections) by system organ class, preferred term, severity and relationship to treatment
Pre-Subgam (Pre-study-Infusion 3)

			Severity and relationship to treatment															
			Mild				Moderate				Severe				Not known			
System Organ Class	Preferred term	n	NR	R	NS	Tot	NR	R	NS	Tot	NR	R	NS	Tot	NR	R	NS	Tot
Skin and subcutaneous tissue disorders	Rosacea	1	1	0	0	1	0	0	0	0	0	0	0	0	0	0	0	0
	Urticaria NOS	2	0	1	0	1	1	0	0	1	0	0	0	0	0	0	0	0
	Total reports	3	1	1	0	2	1	0	0	1	0	0	0	0	0	0	0	0
	Total patients	3	1	1	0	2	1	0	0	1	0	0	0	0	0	0	0	0
Vascular disorders	Hypotension NOS	1	1	0	0	1	0	0	0	0	0	0	0	0	0	0	0	0
	Phlebothrombosis	1	0	0	0	0	1	0	0	1	0	0	0	0	0	0	0	0
	Total reports	2	1	0	0	1	1	0	0	1	0	0	0	0	0	0	0	0
	Total patients	2	1	0	0	1	1	0	0	1	0	0	0	0	0	0	0	0
All disorders	Total reports	61	39	2	0	41	15	0	0	15	5	0	0	5	0	0	0	0
	Total patients	28	21	2	0	22	10	0	0	10	4	0	0	4	0	0	0	0

NR = not related; R = related; NS = not specified; Tot = total.

Program: T0230.TEM, Version: 8.2, Datetime: 07FEB07:09:48

Table 14.3.1.5: Adverse events (excluding infections) by system organ class, preferred term, severity and relationship to treatment
Post-Subgam (Infusion 4-EOS)

			Severity and relationship to treatment															
			Mild				Moderate				Severe				Not known			
System Organ Class	Preferred term	n	NR	R	NS	Tot	NR	R	NS	Tot	NR	R	NS	Tot	NR	R	NS	Tot
Blood and lymphatic system disorders	Lymphadenopathy	3	1	0	0	1	2	0	0	2	0	0	0	0	0	0	0	0
	Neutropenia	1	1	0	0	1	0	0	0	0	0	0	0	0	0	0	0	0
	Pernicious anaemia	1	0	0	0	0	1	0	0	1	0	0	0	0	0	0	0	0
	Splenomegaly	1	1	0	0	1	0	0	0	0	0	0	0	0	0	0	0	0
	Total reports	6	3	0	0	3	3	0	0	3	0	0	0	0	0	0	0	0
	Total patients	6	3	0	0	3	3	0	0	3	0	0	0	0	0	0	0	0
Cardiac disorders	Atrial fibrillation	1	0	0	0	0	1	0	0	1	0	0	0	0	0	0	0	0
	Bradycardia NOS	1	1	0	0	1	0	0	0	0	0	0	0	0	0	0	0	0
	Palpitations	1	1	0	0	1	0	0	0	0	0	0	0	0	0	0	0	0
	Total reports	3	2	0	0	2	1	0	0	1	0	0	0	0	0	0	0	0
	Total patients	3	2	0	0	2	1	0	0	1	0	0	0	0	0	0	0	0

NR = not related; R = related; NS = not specified; Tot = total.

Program: T0231.TEM, Version: 8.2, Datetime: 07FEB07:09:48

Table 14.3.1.5: Adverse events (excluding infections) by system organ class, preferred term, severity and relationship to treatment
Post-Subgam (Infusion 4-EOS)

			Severity and relationship to treatment															
			Mild				Moderate				Severe				Not known			
System Organ Class	Preferred term	n	NR	R	NS	Tot	NR	R	NS	Tot	NR	R	NS	Tot	NR	R	NS	Tot
Congenital, familial and genetic disorders	Pigmented naevus	1	1	0	0	1	0	0	0	0	0	0	0	0	0	0	0	0
	Total reports	1	1	0	0	1	0	0	0	0	0	0	0	0	0	0	0	0
	Total patients	1	1	0	0	1	0	0	0	0	0	0	0	0	0	0	0	0
Ear and labyrinth disorders	Cerumen impaction	2	2	0	0	2	0	0	0	0	0	0	0	0	0	0	0	0
	Ear congestion	1	0	0	0	0	1	0	0	1	0	0	0	0	0	0	0	0
	Ear pain	11	10	0	0	10	1	0	0	1	0	0	0	0	0	0	0	0
	Hypoacusis	2	0	0	0	0	1	0	0	1	1	0	0	1	0	0	0	0
	Otorrhoea	2	1	0	0	1	1	0	0	1	0	0	0	0	0	0	0	0
	Sensation of block in ear	2	0	0	0	0	2	0	0	2	0	0	0	0	0	0	0	0
	Vertigo	1	0	0	0	0	1	0	0	1	0	0	0	0	0	0	0	0
	Total reports	21	13	0	0	13	7	0	0	7	1	0	0	1	0	0	0	0
	Total patients	11	8	0	0	8	6	0	0	6	1	0	0	1	0	0	0	0
Endocrine disorders	Cushingoid	1	1	0	0	1	0	0	0	0	0	0	0	0	0	0	0	0
	Total reports	1	1	0	0	1	0	0	0	0	0	0	0	0	0	0	0	0
	Total patients	1	1	0	0	1	0	0	0	0	0	0	0	0	0	0	0	0

NR = not related; R = related; NS = not specified; Tot = total.

Program: T0231.TEM, Version: 8.2, Datetime: 07FEB07:09:48

Table 14.3.1.5: Adverse events (excluding infections) by system organ class, preferred term, severity and relationship to treatment
Post-Subgam (Infusion 4-EOS)

			Severity and relationship to treatment															
			Mild				Moderate				Severe				Not known			
System Organ Class	Preferred term	n	NR	R	NS	Tot	NR	R	NS	Tot	NR	R	NS	Tot	NR	R	NS	Tot
Eye disorders	Eye pain	3	3	0	0	3	0	0	0	0	0	0	0	0	0	0	0	0
	Eye pruritus	1	1	0	0	1	0	0	0	0	0	0	0	0	0	0	0	0
	Eye swelling	1	1	0	0	1	0	0	0	0	0	0	0	0	0	0	0	0
	Photophobia	2	1	0	0	1	0	0	0	0	1	0	0	1	0	0	0	0
	Vision blurred	1	1	0	0	1	0	0	0	0	0	0	0	0	0	0	0	0
	Visual disturbance NOS	1	1	0	0	1	0	0	0	0	0	0	0	0	0	0	0	0
	Total reports	9	8	0	0	8	0	0	0	0	1	0	0	1	0	0	0	0
	Total patients	7	7	0	0	7	0	0	0	0	1	0	0	1	0	0	0	0

NR = not related; R = related; NS = not specified; Tot = total.

Program: T0231.TEM, Version: 8.2, Datetime: 07FEB07:09:48

Table 14.3.1.5: Adverse events (excluding infections) by system organ class, preferred term, severity and relationship to treatment
Post-Subgam (Infusion 4-EOS)

			Severity and relationship to treatment															
			Mild				Moderate				Severe				Not known			
System Organ Class	Preferred term	n	NR	R	NS	Tot	NR	R	NS	Tot	NR	R	NS	Tot	NR	R	NS	Tot
Gastrointestinal disorders	Abdominal pain NOS	18	10	0	0	10	6	0	0	6	2	0	0	2	0	0	0	0
	Abdominal pain upper	14	8	1	0	9	5	0	0	5	0	0	0	0	0	0	0	0
	Aphthous stomatitis	1	0	1	0	1	0	0	0	0	0	0	0	0	0	0	0	0
	Appendicitis	1	0	0	0	0	0	0	0	0	1	0	0	1	0	0	0	0
	Chapped lips	1	1	0	0	1	0	0	0	0	0	0	0	0	0	0	0	0
	Colitis microscopic	1	0	0	0	0	1	0	0	1	0	0	0	0	0	0	0	0
	Constipation	2	2	0	0	2	0	0	0	0	0	0	0	0	0	0	0	0
	Diarrhoea NOS	57	41	2	0	43	12	0	0	12	2	0	0	2	0	0	0	0
	Dyspepsia	12	11	0	0	11	1	0	0	1	0	0	0	0	0	0	0	0
	Dysphagia	1	0	0	0	0	1	0	0	1	0	0	0	0	0	0	0	0
	Epigastric discomfort	1	0	0	0	0	1	0	0	1	0	0	0	0	0	0	0	0
	Gastrointestinal upset	1	1	0	0	1	0	0	0	0	0	0	0	0	0	0	0	0
	Gastrooesophageal reflux disease	1	1	0	0	1	0	0	0	0	0	0	0	0	0	0	0	0
	Gingival swelling	1	0	0	0	0	1	0	0	1	0	0	0	0	0	0	0	0
	Glossodynia	1	1	0	0	1	0	0	0	0	0	0	0	0	0	0	0	0
	Haemorrhoidal haemorrhage	1	1	0	0	1	0	0	0	0	0	0	0	0	0	0	0	0
	Lip pain	1	1	0	0	1	0	0	0	0	0	0	0	0	0	0	0	0
	Lip ulceration	1	1	0	0	1	0	0	0	0	0	0	0	0	0	0	0	0
	Mouth ulceration	28	19	0	0	19	8	1	0	9	0	0	0	0	0	0	0	0

NR = not related; R = related; NS = not specified; Tot = total.

Program: T0231.TEM, Version: 8.2, Datetime: 07FEB07:09:48

Table 14.3.1.5: Adverse events (excluding infections) by system organ class, preferred term, severity and relationship to treatment
Post-Subgam (Infusion 4-EOS)

			Severity and relationship to treatment															
			Mild				Moderate				Severe				Not known			
System Organ Class	Preferred term	n	NR	R	NS	Tot	NR	R	NS	Tot	NR	R	NS	Tot	NR	R	NS	Tot
Gastrointestinal disorders	Nausea	56	37	3	0	40	12	0	0	12	4	0	0	4	0	0	0	0
	Oral pain	2	0	0	0	0	1	0	0	1	1	0	0	1	0	0	0	0
	Stomach discomfort	1	0	0	0	0	1	0	0	1	0	0	0	0	0	0	0	0
	Teething	1	1	0	0	1	0	0	0	0	0	0	0	0	0	0	0	0
	Toothache	2	2	0	0	2	0	0	0	0	0	0	0	0	0	0	0	0
	Vomiting NOS	57	43	5	0	48	8	0	0	8	1	0	0	1	0	0	0	0
	Total reports	263	181	12	0	193	58	1	0	59	11	0	0	11	0	0	0	0
	Total patients	43	38	7	0	40	21	1	0	21	7	0	0	7	0	0	0	0

NR = not related; R = related; NS = not specified; Tot = total.

Program: T0231.TEM, Version: 8.2, Datetime: 07FEB07:09:48

Table 14.3.1.5: Adverse events (excluding infections) by system organ class, preferred term, severity and relationship to treatment
Post-Subgam (Infusion 4-EOS)

System Organ Class	Preferred term	n	Severity and relationship to treatment															
			Mild				Moderate				Severe				Not known			
			NR	R	NS	Tot	NR	R	NS	Tot	NR	R	NS	Tot	NR	R	NS	Tot
General disorders and administration site conditions	Application site rash	1	1	0	0	1	0	0	0	0	0	0	0	0	0	0	0	0
	Asthenia	5	1	0	0	1	2	1	0	3	1	0	0	1	0	0	0	0
	Chest pain	15	7	0	0	7	5	1	0	6	2	0	0	2	0	0	0	0
	Chest tightness	20	16	1	0	17	3	0	0	3	0	0	0	0	0	0	0	0
	Fall	2	2	0	0	2	0	0	0	0	0	0	0	0	0	0	0	0
	Fatigue	20	15	1	0	16	3	0	0	3	1	0	0	1	0	0	0	0
	Feeling abnormal	1	1	0	0	1	0	0	0	0	0	0	0	0	0	0	0	0
	Feeling cold	2	1	1	0	2	0	0	0	0	0	0	0	0	0	0	0	0
	Gait abnormal	1	1	0	0	1	0	0	0	0	0	0	0	0	0	0	0	0
	Granuloma NOS	1	0	0	0	0	1	0	0	1	0	0	0	0	0	0	0	0
	Hernia NOS	1	0	0	0	0	1	0	0	1	0	0	0	0	0	0	0	0
	Ill-defined disorder NOS	18	13	1	0	14	3	1	0	4	0	0	0	0	0	0	0	0
	Influenza like illness	12	10	0	0	10	2	0	0	2	0	0	0	0	0	0	0	0
	Infusion site burning	1	0	1	0	1	0	0	0	0	0	0	0	0	0	0	0	0
	Infusion site erythema	12	1	10	0	11	0	1	0	1	0	0	0	0	0	0	0	0
	Infusion site induration	2	0	2	0	2	0	0	0	0	0	0	0	0	0	0	0	0
	Infusion site inflammation	16	2	13	0	15	0	1	0	1	0	0	0	0	0	0	0	0
	Infusion site pain	5	0	5	0	5	0	0	0	0	0	0	0	0	0	0	0	0
	Infusion site pruritus	4	0	4	0	4	0	0	0	0	0	0	0	0	0	0	0	0

NR = not related; R = related; NS = not specified; Tot = total.

Program: T0231.TEM, Version: 8.2, Datetime: 07FEB07:09:48

Table 14.3.1.5: Adverse events (excluding infections) by system organ class, preferred term, severity and relationship to treatment
Post-Subgam (Infusion 4-EOS)

System Organ Class	Preferred term	n	Mild NR	Mild R	Mild NS	Mild Tot	Moderate NR	Moderate R	Moderate NS	Moderate Tot	Severe NR	Severe R	Severe NS	Severe Tot	Not known NR	Not known R	Not known NS	Not known Tot
General disorders and administration site conditions	Infusion site swelling	14	1	10	0	11	0	3	0	3	0	0	0	0	0	0	0	0
	Infusion site tenderness	3	0	1	0	1	0	2	0	2	0	0	0	0	0	0	0	0
	Injection site bruising	5	1	4	0	5	0	0	0	0	0	0	0	0	0	0	0	0
	Injection site discomfort	1	0	1	0	1	0	0	0	0	0	0	0	0	0	0	0	0
	Injection site haemorrhage	1	0	0	0	0	0	1	0	1	0	0	0	0	0	0	0	0
	Injection site paraesthesia	2	0	2	0	2	0	0	0	0	0	0	0	0	0	0	0	0
	Injection site rash	12	1	11	0	12	0	0	0	0	0	0	0	0	0	0	0	0
	Injection site stinging	2	0	2	0	2	0	0	0	0	0	0	0	0	0	0	0	0
	Injection site urticaria	2	0	0	0	0	0	2	0	2	0	0	0	0	0	0	0	0
	Lethargy	15	11	1	0	12	2	0	0	2	1	0	0	1	0	0	0	0
	Malaise	9	6	1	0	7	2	0	0	2	0	0	0	0	0	0	0	0
	Oedema peripheral	1	1	0	0	1	0	0	0	0	0	0	0	0	0	0	0	0
	Pain NOS	6	4	2	0	6	0	0	0	0	0	0	0	0	0	0	0	0
	Pyrexia	27	14	1	0	15	10	0	0	10	2	0	0	2	0	0	0	0
	Rigors	15	12	0	0	12	1	1	0	2	1	0	0	1	0	0	0	0
	Sensation of foreign body NOS	1	1	0	0	1	0	0	0	0	0	0	0	0	0	0	0	0
	Total reports	255	123	75	0	198	35	14	0	49	8	0	0	8	0	0	0	0
	Total patients	44	30	22	0	38	22	8	0	24	4	0	0	4	0	0	0	0

NR = not related; R = related; NS = not specified; Tot = total.

Program: T0231.TEM, Version: 8.2, Datetime: 07FEB07:09:48

Table 14.3.1.5: Adverse events (excluding infections) by system organ class, preferred term, severity and relationship to treatment
Post-Subgam (Infusion 4-EOS)

			Severity and relationship to treatment															
			Mild				Moderate				Severe				Not known			
System Organ Class	Preferred term	n	NR	R	NS	Tot	NR	R	NS	Tot	NR	R	NS	Tot	NR	R	NS	Tot
Immune system disorders	Hypersensitivity NOS	1	0	0	0	0	0	0	0	0	1	0	0	1	0	0	0	0
	Seasonal allergy	7	6	0	0	6	1	0	0	1	0	0	0	0	0	0	0	0
	Total reports	8	6	0	0	6	1	0	0	1	1	0	0	1	0	0	0	0
	Total patients	7	6	0	0	6	1	0	0	1	1	0	0	1	0	0	0	0

NR = not related; R = related; NS = not specified; Tot = total.

Program: T0231.TEM, Version: 8.2, Datetime: 07FEB07:09:48

Table 14.3.1.5: Adverse events (excluding infections) by system organ class, preferred term, severity and relationship to treatment
Post-Subgam (Infusion 4-EOS)

			Severity and relationship to treatment															
			Mild				Moderate				Severe				Not known			
System Organ Class	Preferred term	n	NR	R	NS	Tot	NR	R	NS	Tot	NR	R	NS	Tot	NR	R	NS	Tot
Injury, poisoning and procedural complications	Animal bite	2	2	0	0	2	0	0	0	0	0	0	0	0	0	0	0	0
	Arthropod bite	1	1	0	0	1	0	0	0	0	0	0	0	0	0	0	0	0
	Arthropod sting	1	1	0	0	1	0	0	0	0	0	0	0	0	0	0	0	0
	Foot fracture	2	2	0	0	2	0	0	0	0	0	0	0	0	0	0	0	0
	Head injury	1	1	0	0	1	0	0	0	0	0	0	0	0	0	0	0	0
	Joint dislocation	3	1	0	0	1	2	0	0	2	0	0	0	0	0	0	0	0
	Joint sprain	2	0	0	0	0	2	0	0	2	0	0	0	0	0	0	0	0
	Ligament injury NOS	1	1	0	0	1	0	0	0	0	0	0	0	0	0	0	0	0
	Lumbar puncture headache	2	0	0	0	0	1	0	0	1	1	0	0	1	0	0	0	0
	Open fracture	1	1	0	0	1	0	0	0	0	0	0	0	0	0	0	0	0
	Post procedural pain	1	0	0	0	0	1	0	0	1	0	0	0	0	0	0	0	0
	Rib fracture	2	0	0	0	0	2	0	0	2	0	0	0	0	0	0	0	0
	Skin laceration	2	2	0	0	2	0	0	0	0	0	0	0	0	0	0	0	0
	Thermal burn	1	1	0	0	1	0	0	0	0	0	0	0	0	0	0	0	0
	Tooth injury	2	2	0	0	2	0	0	0	0	0	0	0	0	0	0	0	0
	Total reports	24	15	0	0	15	8	0	0	8	1	0	0	1	0	0	0	0
	Total patients	17	11	0	0	11	6	0	0	6	1	0	0	1	0	0	0	0

NR = not related; R = related; NS = not specified; Tot = total.

Table 14.3.1.5: Adverse events (excluding infections) by system organ class, preferred term, severity and relationship to treatment
Post-Subgam (Infusion 4-EOS)

System Organ Class	Preferred term	n	Mild				Moderate				Severe				Not known			
			NR	R	NS	Tot	NR	R	NS	Tot	NR	R	NS	Tot	NR	R	NS	Tot
Investigations	Alanine aminotransferase increased	1	0	0	0	0	1	0	0	1	0	0	0	0	0	0	0	0
	Aspartate aminotransferase increased	1	0	0	0	0	1	0	0	1	0	0	0	0	0	0	0	0
	Biopsy skin	1	1	0	0	1	0	0	0	0	0	0	0	0	0	0	0	0
	Blood creatine phosphokinase increased	1	0	0	0	0	1	0	0	1	0	0	0	0	0	0	0	0
	Blood in stool	1	1	0	0	1	0	0	0	0	0	0	0	0	0	0	0	0
	Blood potassium decreased	1	0	0	0	0	0	0	0	0	1	0	0	1	0	0	0	0
	Blood pressure increased	2	0	1	0	1	1	0	0	1	0	0	0	0	0	0	0	0
	Blood urine present	2	2	0	0	2	0	0	0	0	0	0	0	0	0	0	0	0
	Body temperature increased	30	22	2	0	24	6	0	0	6	0	0	0	0	0	0	0	0
	Neutrophil count decreased	1	0	0	0	0	1	0	0	1	0	0	0	0	0	0	0	0
	Sputum	2	1	0	0	1	1	0	0	1	0	0	0	0	0	0	0	0
	Sputum increased	4	3	0	0	3	1	0	0	1	0	0	0	0	0	0	0	0
	Weight decreased	1	0	0	0	0	1	0	0	1	0	0	0	0	0	0	0	0
	Total reports	48	30	3	0	33	14	0	0	14	1	0	0	1	0	0	0	0
	Total patients	19	13	2	0	13	8	0	0	8	1	0	0	1	0	0	0	0

NR = not related; R = related; NS = not specified; Tot = total.

Program: T0231.TEM, Version: 8.2, Datetime: 07FEB07:09:48

Table 14.3.1.5: Adverse events (excluding infections) by system organ class, preferred term, severity and relationship to treatment
Post-Subgam (Infusion 4-EOS)

			Severity and relationship to treatment															
			Mild				Moderate				Severe				Not known			
System Organ Class	Preferred term	n	NR	R	NS	Tot	NR	R	NS	Tot	NR	R	NS	Tot	NR	R	NS	Tot
Metabolism and nutrition disorders	Anorexia	2	2	0	0	2	0	0	0	0	0	0	0	0	0	0	0	0
	Diabetes mellitus inadequate control	1	0	0	0	0	1	0	0	1	0	0	0	0	0	0	0	0
	Iron deficiency	2	2	0	0	2	.0	0	0	0	0	0	0	0	0	0	0	0
	Total reports	5	4	0	0	4	1	0	0	1	0	0	0	0	0	0	0	0
	Total patients	4	4	0	0	4	1	0	0	1	0	0	0	0	0	0	0	0

NR = not related; R = related; NS = not specified; Tot = total.

Program: T0231.TEM, Version: 8.2, Datetime: 07FEB07:09:48

Table 14.3.1.5: Adverse events (excluding infections) by system organ class, preferred term, severity and relationship to treatment
Post-Subgam (Infusion 4-EOS)

			Severity and relationship to treatment															
			Mild				Moderate				Severe				Not known			
System Organ Class	Preferred term	n	NR	R	NS	Tot	NR	R	NS	Tot	NR	R	NS	Tot	NR	R	NS	Tot
Musculoskeletal and connective tissue disorders	Arthralgia	48	21	2	0	23	19	0	0	19	6	0	0	6	0	0	0	0
	Arthritis NOS	4	2	0	0	2	2	0	0	2	0	0	0	0	0	0	0	0
	Back pain	17	4	1	0	5	8	0	0	8	4	0	0	4	0	0	0	0
	Bone pain	2	2	0	0	2	0	0	0	0	0	0	0	0	0	0	0	0
	Chest wall pain	2	2	0	0	2	0	0	0	0	0	0	0	0	0	0	0	0
	Chondromalacia patellae	1	1	0	0	1	0	0	0	0	0	0	0	0	0	0	0	0
	Facial pain	1	1	0	0	1	0	0	0	0	0	0	0	0	0	0	0	0
	Flank pain	1	0	0	0	0	1	0	0	1	0	0	0	0	0	0	0	0
	Groin pain	1	0	0	0	0	1	0	0	1	0	0	0	0	0	0	0	0
	Joint disorder NOS	1	0	0	0	0	1	0	0	1	0	0	0	0	0	0	0	0
	Joint stiffness	1	0	0	0	0	1	0	0	1	0	0	0	0	0	0	0	0
	Joint swelling	3	3	0	0	3	0	0	0	0	0	0	0	0	0	0	0	0
	Local swelling	2	2	0	0	2	0	0	0	0	0	0	0	0	0	0	0	0
	Muscle spasms	3	3	0	0	3	0	0	0	0	0	0	0	0	0	0	0	0
	Musculoskeletal discomfort	2	1	0	0	1	1	0	0	1	0	0	0	0	0	0	0	0
	Musculoskeletal stiffness	3	2	1	0	3	0	0	0	0	0	0	0	0	0	0	0	0
	Myalgia	2	1	0	0	1	1	0	0	1	0	0	0	0	0	0	0	0
	Neck pain	5	2	0	0	2	2	0	0	2	1	0	0	1	0	0	0	0
	Osteoporosis NOS	2	1	0	0	1	1	0	0	1	0	0	0	0	0	0	0	0

NR = not related; R = related; NS = not specified; Tot = total.

Program: T0231.TEM, Version: 8.2, Datetime: 07FEB07:09:48

Table 14.3.1.5: Adverse events (excluding infections) by system organ class, preferred term, severity and relationship to treatment
Post-Subgam (Infusion 4-EOS)

			Severity and relationship to treatment															
			Mild				Moderate				Severe				Not known			
System Organ Class	Preferred term	n	NR	R	NS	Tot	NR	R	NS	Tot	NR	R	NS	Tot	NR	R	NS	Tot
Musculoskeletal and connective tissue disorders	Pain in foot	2	1	0	0	1	1	0	0	1	0	0	0	0	0	0	0	0
	Pain in jaw	1	1	0	0	1	0	0	0	0	0	0	0	0	0	0	0	0
	Pain in limb	14	6	1	0	7	3	0	0	3	4	0	0	4	0	0	0	0
	Peripheral swelling	1	1	0	0	1	0	0	0	0	0	0	0	0	0	0	0	0
	Polymyalgia	1	1	0	0	1	0	0	0	0	0	0	0	0	0	0	0	0
	Swelling NOS	3	3	0	0	3	0	0	0	0	0	0	0	0	0	0	0	0
	Total reports	123	61	5	0	66	42	0	0	42	15	0	0	15	0	0	0	0
	Total patients	28	24	5	0	24	16	0	0	16	4	0	0	4	0	0	0	0

NR = not related; R = related; NS = not specified; Tot = total.

Program: T0231.TEM, Version: 8.2, Datetime: 07FEB07:09:48

Table 14.3.1.5: Adverse events (excluding infections) by system organ class, preferred term, severity and relationship to treatment
Post-Subgam (Infusion 4-EOS)

System Organ Class	Preferred term	n	Mild				Moderate				Severe				Not known			
			NR	R	NS	Tot	NR	R	NS	Tot	NR	R	NS	Tot	NR	R	NS	Tot
Nervous system disorders	Aphonia	1	0	0	0	0	1	0	0	1	0	0	0	0	0	0	0	0
	Dizziness	31	16	1	0	17	12	1	0	13	0	0	0	0	0	1	0	1
	Entrapment neuropathy	1	0	0	0	0	1	0	0	1	0	0	0	0	0	0	0	0
	Headache	119	75	7	0	82	26	1	0	27	10	0	0	10	0	0	0	0
	Hyperaesthesia	1	0	0	0	0	1	0	0	1	0	0	0	0	0	0	0	0
	Hypoaesthesia	3	3	0	0	3	0	0	0	0	0	0	0	0	0	0	0	0
	Loss of consciousness	1	0	0	0	0	1	0	0	1	0	0	0	0	0	0	0	0
	Migraine NOS	13	10	0	0	10	2	1	0	3	0	0	0	0	0	0	0	0
	Neurological disorder NOS	1	0	0	0	0	1	0	0	1	0	0	0	0	0	0	0	0
	Paraesthesia	3	1	1	0	2	1	0	0	1	0	0	0	0	0	0	0	0
	Paraesthesia oral	1	0	0	0	0	1	0	0	1	0	0	0	0	0	0	0	0
	Restless legs syndrome	2	0	0	0	0	0	0	0	0	2	0	0	2	0	0	0	0
	Sinus headache	8	6	0	0	6	1	1	0	2	0	0	0	0	0	0	0	0
	Syncope	1	1	0	0	1	0	0	0	0	0	0	0	0	0	0	0	0
	Tremor	2	0	1	0	1	1	0	0	1	0	0	0	0	0	0	0	0
	Total reports	188	112	10	0	122	49	4	0	53	12	0	0	12	0	1	0	1
	Total patients	32	24	8	0	27	12	4	0	14	5	0	0	5	0	1	0	1

NR = not related; R = related; NS = not specified; Tot = total.

Program: T0231.TEM, Version: 8.2, Datetime: 07FEB07:09:48

Table 14.3.1.5: Adverse events (excluding infections) by system organ class, preferred term, severity and relationship to treatment
Post-Subgam (Infusion 4-EOS)

			Severity and relationship to treatment															
			Mild				Moderate				Severe				Not known			
System Organ Class	Preferred term	n	NR	R	NS	Tot	NR	R	NS	Tot	NR	R	NS	Tot	NR	R	NS	Tot
Pregnancy, puerperium and perinatal conditions	Abortion spontaneous NOS	1	0	0	0	0	1	0	0	1	0	0	0	0	0	0	0	0
	Vomiting in pregnancy	3	2	0	0	2	0	0	0	0	1	0	0	1	0	0	0	0
	Total reports	4	2	0	0	2	1	0	0	1	1	0	0	1	0	0	0	0
	Total patients	1	1	0	0	1	1	0	0	1	1	0	0	1	0	0	0	0
Psychiatric disorders	Anxiety	1	0	0	0	0	0	1	0	1	0	0	0	0	0	0	0	0
	Crying	1	1	0	0	1	0	0	0	0	0	0	0	0	0	0	0	0
	Depressed mood	1	1	0	0	1	0	0	0	0	0	0	0	0	0	0	0	0
	Depression	4	2	0	0	2	2	0	0	2	0	0	0	0	0	0	0	0
	Insomnia	4	2	0	0	2	2	0	0	2	0	0	0	0	0	0	0	0
	Panic attack	2	0	0	0	0	2	0	0	2	0	0	0	0	0	0	0	0
	Restlessness	1	0	0	0	0	1	0	0	1	0	0	0	0	0	0	0	0
	Stress symptoms	2	2	0	0	2	0	0	0	0	0	0	0	0	0	0	0	0
	Total reports	16	8	0	0	8	7	1	0	8	0	0	0	0	0	0	0	0
	Total patients	7	6	0	0	6	4	1	0	5	0	0	0	0	0	0	0	0

NR = not related; R = related; NS = not specified; Tot = total.

Program: T0231.TEM, Version: 8.2, Datetime: 07FEB07:09:48

Table 14.3.1.5: Adverse events (excluding infections) by system organ class, preferred term, severity and relationship to treatment
Post-Subgam (Infusion 4-EOS)

			Severity and relationship to treatment															
			Mild				Moderate				Severe				Not known			
System Organ Class	Preferred term	n	NR	R	NS	Tot	NR	R	NS	Tot	NR	R	NS	Tot	NR	R	NS	Tot
Renal and urinary disorders	Dysuria	2	2	0	0	2	0	0	0	0	0	0	0	0	0	0	0	0
	Glycosuria during pregnancy	1	1	0	0	1	0	0	0	0	0	0	0	0	0	0	0	0
	Nephrolithiasis	3	0	0	0	0	0	0	0	0	3	0	0	3	0	0	0	0
	Renal pain	1	0	0	0	0	1	0	0	1	0	0	0	0	0	0	0	0
	Total reports	7	3	0	0	3	1	0	0	1	3	0	0	3	0	0	0	0
	Total patients	4	2	0	0	2	1	0	0	1	2	0	0	2	0	0	0	0
Reproductive system and breast disorders	Vaginal haemorrhage	1	1	0	0	1	0	0	0	0	0	0	0	0	0	0	0	0
	Total reports	1	1	0	0	1	0	0	0	0	0	0	0	0	0	0	0	0
	Total patients	1	1	0	0	1	0	0	0	0	0	0	0	0	0	0	0	0

NR = not related; R = related; NS = not specified; Tot = total.

Program: T0231.TEM, Version: 8.2, Datetime: 07FEB07:09:48

Table 14.3.1.5: Adverse events (excluding infections) by system organ class, preferred term, severity and relationship to treatment
Post-Subgam (Infusion 4-EOS)

			Severity and relationship to treatment															
			Mild				Moderate				Severe				Not known			
System Organ Class	Preferred term	n	NR	R	NS	Tot	NR	R	NS	Tot	NR	R	NS	Tot	NR	R	NS	Tot
Respiratory, thoracic and mediastinal disorders	Asthma NOS	10	3	0	0	3	4	0	0	4	2	1	0	3	0	0	0	0
	Asthma aggravated	3	2	0	0	2	1	0	0	1	0	0	0	0	0	0	0	0
	Atelectasis	1	0	0	0	0	1	0	0	1	0	0	0	0	0	0	0	0
	Bronchitis NOS	1	0	0	0	0	1	0	0	1	0	0	0	0	0	0	0	0
	Bronchospasm NOS	1	1	0	0	1	0	0	0	0	0	0	0	0	0	0	0	0
	Catarrh	3	1	0	0	1	2	0	0	2	0	0	0	0	0	0	0	0
	Cough	141	102	0	0	102	36	0	0	36	2	0	0	2	0	0	1	1
	Cough aggravated	3	0	0	0	0	2	0	0	2	1	0	0	1	0	0	0	0
	Dyspnoea NOS	9	2	0	0	2	7	0	0	7	0	0	0	0	0	0	0	0
	Epistaxis	12	10	0	0	10	2	0	0	2	0	0	0	0	0	0	0	0
	Haemoptysis	2	2	0	0	2	0	0	0	0	0	0	0	0	0	0	0	0
	Hoarseness	1	0	0	0	0	1	0	0	1	0	0	0	0	0	0	0	0
	Laryngitis NOS	1	1	0	0	1	0	0	0	0	0	0	0	0	0	0	0	0
	Nasal congestion	5	4	0	0	4	1	0	0	1	0	0	0	0	0	0	0	0
	Nasal passage irritation	2	2	0	0	2	0	0	0	0	0	0	0	0	0	0	0	0
	Paranasal sinus hypersecretion	2	2	0	0	2	0	0	0	0	0	0	0	0	0	0	0	0
	Pharyngeal ulceration	3	1	0	0	1	2	0	0	2	0	0	0	0	0	0	0	0
	Pharyngitis	103	66	0	0	66	37	0	0	37	0	0	0	0	0	0	0	0
	Pleuritic pain	1	1	0	0	1	0	0	0	0	0	0	0	0	0	0	0	0

NR = not related; R = related; NS = not specified; Tot = total.

Table 14.3.1.5: Adverse events (excluding infections) by system organ class, preferred term, severity and relationship to treatment
Post-Subgam (Infusion 4-EOS)

System Organ Class	Preferred term	n	Mild NR	Mild R	Mild NS	Mild Tot	Moderate NR	Moderate R	Moderate NS	Moderate Tot	Severe NR	Severe R	Severe NS	Severe Tot	Not known NR	Not known R	Not known NS	Not known Tot
Respiratory, thoracic and mediastinal disorders	Postnasal drip	1	1	0	0	1	0	0	0	0	0	0	0	0	0	0	0	0
	Productive cough	2	1	0	0	1	1	0	0	1	0	0	0	0	0	0	0	0
	Respiratory disorder NOS	1	1	0	0	1	0	0	0	0	0	0	0	0	0	0	0	0
	Respiratory tract irritation	1	0	0	0	0	0	0	0	0	1	0	0	1	0	0	0	0
	Rhinitis NOS	7	3	0	0	3	4	0	0	4	0	0	0	0	0	0	0	0
	Rhinitis NOS exacerbated	1	0	0	0	0	1	0	0	1	0	0	0	0	0	0	0	0
	Rhinitis perennial	1	1	0	0	1	0	0	0	0	0	0	0	0	0	0	0	0
	Rhinorrhoea	21	17	0	0	17	4	0	0	4	0	0	0	0	0	0	0	0
	Sinus congestion	6	6	0	0	6	0	0	0	0	0	0	0	0	0	0	0	0
	Sinus pain	24	16	0	0	16	7	0	0	7	1	0	0	1	0	0	0	0
	Sinus polyp	1	1	0	0	1	0	0	0	0	0	0	0	0	0	0	0	0
	Sneezing	3	3	0	0	3	0	0	0	0	0	0	0	0	0	0	0	0
	Sputum discoloured	4	4	0	0	4	0	0	0	0	0	0	0	0	0	0	0	0
	Throat tightness	1	1	0	0	1	0	0	0	0	0	0	0	0	0	0	0	0
	Wheezing	32	28	1	0	29	3	0	0	3	0	0	0	0	0	0	0	0
	Total reports	410	283	1	0	284	117	0	0	117	7	1	0	8	0	0	1	1
	Total patients	45	43	1	0	43	26	0	0	26	5	1	0	6	0	0	1	1

NR = not related; R = related; NS = not specified; Tot = total.

Program: T0231.TEM, Version: 8.2, Datetime: 07FEB07:09:48

Table 14.3.1.5: Adverse events (excluding infections) by system organ class, preferred term, severity and relationship to treatment
Post-Subgam (Infusion 4-EOS)

System Organ Class	Preferred term	n	Severity and relationship to treatment															
			Mild				Moderate				Severe				Not known			
			NR	R	NS	Tot	NR	R	NS	Tot	NR	R	NS	Tot	NR	R	NS	Tot
Skin and subcutaneous tissue disorders	Acne NOS	5	5	0	0	5	0	0	0	0	0	0	0	0	0	0	0	0
	Cold sweat	2	2	0	0	2	0	0	0	0	0	0	0	0	0	0	0	0
	Contusion	9	6	2	0	8	1	0	0	1	0	0	0	0	0	0	0	0
	Dermatitis NOS	1	1	0	0	1	0	0	0	0	0	0	0	0	0	0	0	0
	Dermatitis NOS aggravated	1	0	0	0	0	1	0	0	1	0	0	0	0	0	0	0	0
	Eczema	4	4	0	0	4	0	0	0	0	0	0	0	0	0	0	0	0
	Eczema exacerbated	2	2	0	0	2	0	0	0	0	0	0	0	0	0	0	0	0
	Erythema	1	1	0	0	1	0	0	0	0	0	0	0	0	0	0	0	0
	Heat rash	3	3	0	0	3	0	0	0	0	0	0	0	0	0	0	0	0
	Lichen planus	2	1	0	0	1	1	0	0	1	0	0	0	0	0	0	0	0
	Livedo reticularis	1	1	0	0	1	0	0	0	0	0	0	0	0	0	0	0	0
	Night sweats	2	2	0	0	2	0	0	0	0	0	0	0	0	0	0	0	0
	Pruritus	9	2	5	0	7	0	2	0	2	0	0	0	0	0	0	0	0
	Pruritus generalised	1	1	0	0	1	0	0	0	0	0	0	0	0	0	0	0	0
	Rash NOS	7	5	1	0	6	1	0	0	1	0	0	0	0	0	0	0	0
	Rash pruritic	1	1	0	0	1	0	0	0	0	0	0	0	0	0	0	0	0
	Rosacea	2	1	1	0	2	0	0	0	0	0	0	0	0	0	0	0	0
	Sebaceous gland disorder NOS	1	1	0	0	1	0	0	0	0	0	0	0	0	0	0	0	0
	Skin inflammation NOS	1	1	0	0	1	0	0	0	0	0	0	0	0	0	0	0	0

NR = not related; R = related; NS = not specified; Tot = total.

Program: T0231.TEM, Version: 8.2, Datetime: 07FEB07:09:48

Table 14.3.1.5: Adverse events (excluding infections) by system organ class, preferred term, severity and relationship to treatment
Post-Subgam (Infusion 4-EOS)

			Severity and relationship to treatment															
			Mild				Moderate				Severe				Not known			
System Organ Class	Preferred term	n	NR	R	NS	Tot	NR	R	NS	Tot	NR	R	NS	Tot	NR	R	NS	Tot
Skin and subcutaneous tissue disorders	Skin lesion NOS	2	2	0	0	2	0	0	0	0	0	0	0	0	0	0	0	0
	Skin ulcer	2	2	0	0	2	0	0	0	0	0	0	0	0	0	0	0	0
	Sweating increased	6	3	2	0	5	0	0	0	0	1	0	0	1	0	0	0	0
	Swelling face	3	3	0	0	3	0	0	0	0	0	0	0	0	0	0	0	0
	Urticaria NOS	8	4	0	0	4	3	1	0	4	0	0	0	0	0	0	0	0
	Total reports	76	54	11	0	65	7	3	0	10	1	0	0	1	0	0	0	0
	Total patients	27	24	7	0	25	5	2	0	7	1	0	0	1	0	0	0	0
Surgical and medical procedures	Caesarean section	1	0	0	0	0	0	0	0	0	1	0	0	1	0	0	0	0
	Dental treatment NOS	1	1	0	0	1	0	0	0	0	0	0	0	0	0	0	0	0
	Hernia repair NOS	1	0	0	0	0	1	0	0	1	0	0	0	0	0	0	0	0
	Sinus operation NOS	1	0	0	0	0	1	0	0	1	0	0	0	0	0	0	0	0
	Tooth extraction NOS	1	1	0	0	1	0	0	0	0	0	0	0	0	0	0	0	0
	Tooth repair	1	1	0	0	1	0	0	0	0	0	0	0	0	0	0	0	0
	Vasectomy NOS	1	1	0	0	1	0	0	0	0	0	0	0	0	0	0	0	0
	Total reports	7	4	0	0	4	2	0	0	2	1	0	0	1	0	0	0	0
	Total patients	5	2	0	0	2	2	0	0	2	1	0	0	1	0	0	0	0

NR = not related; R = related; NS = not specified; Tot = total.

Program: T0231.TEM, Version: 8.2, Datetime: 07FEB07:09:48

Table 14.3.1.5: Adverse events (excluding infections) by system organ class, preferred term, severity and relationship to treatment
Post-Subgam (Infusion 4-EOS)

			Severity and relationship to treatment															
			Mild				Moderate				Severe				Not known			
System Organ Class	Preferred term	n	NR	R	NS	Tot	NR	R	NS	Tot	NR	R	NS	Tot	NR	R	NS	Tot
Vascular disorders	Flushing	2	0	1	0	1	0	1	0	1	0	0	0	0	0	0	0	0
	Heat stroke	1	1	0	0	1	0	0	0	0	0	0	0	0	0	0	0	0
	Hot flushes NOS	2	2	0	0	2	0	0	0	0	0	0	0	0	0	0	0	0
	Hypertension NOS	4	2	0	0	2	2	0	0	2	0	0	0	0	0	0	0	0
	Pallor	1	1	0	0	1	0	0	0	0	0	0	0	0	0	0	0	0
	Petechiae	1	1	0	0	1	0	0	0	0	0	0	0	0	0	0	0	0
	Phlebitis NOS	2	0	0	0	0	1	0	0	1	1	0	0	1	0	0	0	0
	Raynaud's phenomenon	1	1	0	0	1	0	0	0	0	0	0	0	0	0	0	0	0
	Total reports	14	8	1	0	9	3	1	0	4	1	0	0	1	0	0	0	0
	Total patients	9	7	1	0	7	3	1	0	4	1	0	0	1	0	0	0	0
All disorders	Total reports	1490	923	118	0	1041	357	24	0	381	65	1	0	66	0	1	1	2
	Total patients	50	49	27	0	49	38	12	0	38	16	1	0	16	0	1	1	2

NR = not related; R = related; NS = not specified; Tot = total.

Program: T0231.TEM, Version: 8.2, Datetime: 07FEB07:09:48

Table 14.3.1.6: Serious adverse events by system organ class, preferred term, severity and relationship to study treatment (excluding Infections)
Pre-Subgam (Pre-study-Infusion 3)

			Severity and relationship to treatment															
			Mild				Moderate				Severe				Not known			
System Organ Class	Preferred term	n	NR	R	NS	Tot	NR	R	NS	Tot	NR	R	NS	Tot	NR	R	NS	Tot
Gastrointestinal disorders	Haematemesis	1	0	0	0	0	0	0	0	0	1	0	0	1	0	0	0	0
	Total reports	1	0	0	0	0	0	0	0	0	1	0	0	1	0	0	0	0
	Total patients	1	0	0	0	0	0	0	0	0	1	0	0	1	0	0	0	0
Vascular disorders	Phlebothrombosis	1	0	0	0	0	1	0	0	1	0	0	0	0	0	0	0	0
	Total reports	1	0	0	0	0	1	0	0	1	0	0	0	0	0	0	0	0
	Total patients	1	0	0	0	0	1	0	0	1	0	0	0	0	0	0	0	0
All disorders	Total reports	2	0	0	0	0	1	0	0	1	1	0	0	1	0	0	0	0
	Total patients	2	0	0	0	0	1	0	0	1	1	0	0	1	0	0	0	0

NR = not related; R = related; NS = not specified; Tot = total.

Program: T0322.TEM, Version: 8.2, Datetime: 07FEB07:09:48

Table 14.3.1.7: Serious adverse events by system organ class, preferred term, severity and relationship to study treatment (excluding Infections)
Post-Subgam (Infusion 4-Last Subgam Infusion)

			Severity and relationship to treatment															
			Mild				Moderate				Severe				Not known			
System Organ Class	Preferred term	n	NR	R	NS	Tot	NR	R	NS	Tot	NR	R	NS	Tot	NR	R	NS	Tot
Cardiac disorders	Atrial fibrillation	1	0	0	0	0	1	0	0	1	0	0	0	0	0	0	0	0
	Total reports	1	0	0	0	0	1	0	0	1	0	0	0	0	0	0	0	0
	Total patients	1	0	0	0	0	1	0	0	1	0	0	0	0	0	0	0	0
Gastrointestinal disorders	Abdominal pain NOS	1	0	0	0	0	0	0	0	0	1	0	0	1	0	0	0	0
	Appendicitis	1	0	0	0	0	0	0	0	0	1	0	0	1	0	0	0	0
	Total reports	2	0	0	0	0	0	0	0	0	2	0	0	2	0	0	0	0
	Total patients	2	0	0	0	0	0	0	0	0	2	0	0	2	0	0	0	0
General disorders and administration site conditions	Chest pain	1	0	0	0	0	0	0	0	0	1	0	0	1	0	0	0	0
	Chest tightness	1	0	0	0	0	1	0	0	1	0	0	0	0	0	0	0	0
	Granuloma NOS	1	0	0	0	0	1	0	0	1	0	0	0	0	0	0	0	0
	Pyrexia	1	0	0	0	0	0	0	0	0	1	0	0	1	0	0	0	0
	Rigors	1	0	0	0	0	0	0	0	0	1	0	0	1	0	0	0	0
	Total reports	5	0	0	0	0	2	0	0	2	3	0	0	3	0	0	0	0
	Total patients	4	0	0	0	0	2	0	0	2	2	0	0	2	0	0	0	0

NR = not related; R = related; NS = not specified; Tot = total.

Program: T0323.TEM, Version: 8.2, Datetime: 07FEB07:09:48

Table 14.3.1.7: Serious adverse events by system organ class, preferred term, severity and relationship to study treatment (excluding Infections)
Post-Subgam (Infusion 4-Last Subgam Infusion)

			Severity and relationship to treatment															
			Mild				Moderate				Severe				Not known			
System Organ Class	Preferred term	n	NR	R	NS	Tot	NR	R	NS	Tot	NR	R	NS	Tot	NR	R	NS	Tot
Injury, poisoning and procedural complications	Open fracture	1	1	0	0	1	0	0	0	0	0	0	0	0	0	0	0	0
	Total reports	1	1	0	0	1	0	0	0	0	0	0	0	0	0	0	0	0
	Total patients	1	1	0	0	1	0	0	0	0	0	0	0	0	0	0	0	0
Investigations	Blood potassium decreased	1	0	0	0	0	0	0	0	0	1	0	0	1	0	0	0	0
	Blood pressure increased	1	0	0	0	0	1	0	0	1	0	0	0	0	0	0	0	0
	Total reports	2	0	0	0	0	1	0	0	1	1	0	0	1	0	0	0	0
	Total patients	2	0	0	0	0	1	0	0	1	1	0	0	1	0	0	0	0
Musculoskeletal and connective tissue disorders	Back pain	1	0	0	0	0	0	0	0	0	1	0	0	1	0	0	0	0
	Neck pain	1	0	0	0	0	0	0	0	0	1	0	0	1	0	0	0	0
	Pain in limb	1	0	0	0	0	0	0	0	0	1	0	0	1	0	0	0	0
	Total reports	3	0	0	0	0	0	0	0	0	3	0	0	3	0	0	0	0
	Total patients	3	0	0	0	0	0	0	0	0	3	0	0	3	0	0	0	0

NR = not related; R = related; NS = not specified; Tot = total.

Program: T0323.TEM, Version: 8.2, Datetime: 07FEB07:09:48

Table 14.3.1.7: Serious adverse events by system organ class, preferred term, severity and relationship to study treatment (excluding Infections)
Post-Subgam (Infusion 4-Last Subgam Infusion)

			Severity and relationship to treatment															
			Mild				Moderate				Severe				Not known			
System Organ Class	Preferred term	n	NR	R	NS	Tot	NR	R	NS	Tot	NR	R	NS	Tot	NR	R	NS	Tot
Nervous system disorders	Neurological disorder NOS	1	0	0	0	0	1	0	0	1	0	0	0	0	0	0	0	0
	Total reports	1	0	0	0	0	1	0	0	1	0	0	0	0	0	0	0	0
	Total patients	1	0	0	0	0	1	0	0	1	0	0	0	0	0	0	0	0
Pregnancy, puerperium and perinatal conditions	Abortion spontaneous NOS	1	0	0	0	0	1	0	0	1	0	0	0	0	0	0	0	0
	Vomiting in pregnancy	1	0	0	0	0	0	0	0	0	1	0	0	1	0	0	0	0
	Total reports	2	0	0	0	0	1	0	0	1	1	0	0	1	0	0	0	0
	Total patients	1	0	0	0	0	1	0	0	1	1	0	0	1	0	0	0	0
Renal and urinary disorders	Nephrolithiasis	1	0	0	0	0	0	0	0	0	1	0	0	1	0	0	0	0
	Total reports	1	0	0	0	0	0	0	0	0	1	0	0	1	0	0	0	0
	Total patients	1	0	0	0	0	0	0	0	0	1	0	0	1	0	0	0	0
Respiratory, thoracic and mediastinal disorders	Asthma NOS	2	0	0	0	0	0	0	0	0	2	0	0	2	0	0	0	0
	Cough	1	0	0	0	0	0	0	0	0	1	0	0	1	0	0	0	0
	Epistaxis	1	0	0	0	0	1	0	0	1	0	0	0	0	0	0	0	0
	Total reports	4	0	0	0	0	1	0	0	1	3	0	0	3	0	0	0	0
	Total patients	3	0	0	0	0	1	0	0	1	2	0	0	2	0	0	0	0

NR = not related; R = related; NS = not specified; Tot = total.

Program: T0323.TEM, Version: 8.2, Datetime: 07FEB07:09:48

Table 14.3.1.7: Serious adverse events by system organ class, preferred term, severity and relationship to study treatment (excluding Infections)
Post-Subgam (Infusion 4-Last Subgam Infusion)

			Severity and relationship to treatment															
			Mild				Moderate				Severe				Not known			
System Organ Class	Preferred term	n	NR	R	NS	Tot	NR	R	NS	Tot	NR	R	NS	Tot	NR	R	NS	Tot
Surgical and medical procedures	Caesarean section	1	0	0	0	0	0	0	0	0	1	0	0	1	0	0	0	0
	Hernia repair NOS	1	0	0	0	0	1	0	0	1	0	0	0	0	0	0	0	0
	Sinus operation NOS	1	0	0	0	0	1	0	0	1	0	0	0	0	0	0	0	0
	Total reports	3	0	0	0	0	2	0	0	2	1	0	0	1	0	0	0	0
	Total patients	3	0	0	0	0	2	0	0	2	1	0	0	1	0	0	0	0
Vascular disorders	Hypertension NOS	1	0	0	0	0	1	0	0	1	0	0	0	0	0	0	0	0
	Total reports	1	0	0	0	0	1	0	0	1	0	0	0	0	0	0	0	0
	Total patients	1	0	0	0	0	1	0	0	1	0	0	0	0	0	0	0	0
All disorders	Total reports	26	1	0	0	1	10	0	0	10	15	0	0	15	0	0	0	0
	Total patients	13	1	0	0	1	7	0	0	7	8	0	0	8	0	0	0	0

NR = not related; R = related; NS = not specified; Tot = total.

Program: T0323.TEM, Version: 8.2, Datetime: 07FEB07:09:48

Table 14.3.1.8: Adverse events (excluding Infections) related to Subgam, by system organ class and preferred term
Post-Subgam (Infusion 4-EOS)

System Organ Class	Preferred Term	Number of Reports	Number of Patients
Gastrointestinal disorders	Abdominal pain upper	1	1
	Aphthous stomatitis	1	1
	Diarrhoea NOS	2	2
	Mouth ulceration	1	1
	Nausea	3	3
	Vomiting NOS	5	3
	Total	13	7
General disorders and administration site conditions	Asthenia	1	1
	Chest pain	1	1
	Chest tightness	1	1
	Fatigue	1	1
	Feeling cold	1	1
	Ill-defined disorder NOS	2	1
	Infusion site burning	1	1
	Infusion site erythema	11	5
	Infusion site induration	2	1
	Infusion site inflammation	14	7
	Infusion site pain	5	5
	Infusion site pruritus	4	3
	Infusion site swelling	13	6
	Infusion site tenderness	3	3
	Injection site bruising	4	1
	Injection site discomfort	1	1
	Injection site haemorrhage	1	1
	Injection site paraesthesia	2	2
	Injection site rash	11	4

Table 14.3.1.8: Adverse events (excluding Infections) related to Subgam, by system organ class and preferred term
Post-Subgam (Infusion 4-EOS)

System Organ Class	Preferred Term	Number of Reports	Number of Patients
General disorders and administration site conditions	Injection site stinging	2	1
	Injection site urticaria	2	1
	Lethargy	1	1
	Malaise	1	1
	Pain NOS	2	1
	Pyrexia	1	1
	Rigors	1	1
	Total	89	25
Investigations	Blood pressure increased	1	1
	Body temperature increased	2	1
	Total	3	2
Musculoskeletal and connective tissue disorders	Arthralgia	2	2
	Back pain	1	1
	Musculoskeletal stiffness	1	1
	Pain in limb	1	1
	Total	5	5
Nervous system disorders	Dizziness	3	2
	Headache	8	7
	Migraine NOS	1	1
	Paraesthesia	1	1

Program: T0497.SAS, Version: 8.2, Datetime: 07FEB07:09:48

Table 14.3.1.8: Adverse events (excluding Infections) related to Subgam, by system organ class and preferred term
Post-Subgam (Infusion 4-EOS)

System Organ Class	Preferred Term	Number of Reports	Number of Patients
Nervous system disorders	Sinus headache	1	1
	Tremor	1	1
	Total	15	12
Psychiatric disorders	Anxiety	1	1
	Total	1	1
Respiratory, thoracic and mediastinal disorders	Asthma NOS	1	1
	Wheezing	1	1
	Total	2	2
Skin and subcutaneous tissue disorders	Contusion	2	1
	Pruritus	7	2
	Rash NOS	1	1
	Rosacea	1	1
	Sweating increased	2	2
	Urticaria NOS	1	1
	Total	14	8
Vascular disorders	Flushing	2	1

Table 14.3.1.8: Adverse events (excluding Infections) related to Subgam, by system organ class and preferred term
Post-Subgam (Infusion 4-EOS)

System Organ Class	Preferred Term	Number of Reports	Number of Patients
Vascular disorders	Total	2	1

Table 14.3.2: Deaths and other serious and significant adverse events (excluding infections)

Preferred term	n	Severity		
		Mild	Moderate	Severe
Abdominal pain NOS	1 (1)	0	0	1 (1)
Abortion spontaneous NOS	1 (1)	0	1 (1)	0
Appendicitis	1 (1)	0	0	1 (1)
Asthma NOS	2 (2)	0	0	2 (2)
Atrial fibrillation	1 (1)	0	1 (1)	0
Back pain	1 (1)	0	0	1 (1)
Blood potassium decreased	1 (1)	0	0	1 (1)
Blood pressure increased	1 (1)	0	1 (1)	0
Caesarean section	1 (1)	0	0	1 (1)
Chest pain	1 (1)	0	0	1 (1)
Chest tightness	1 (1)	0	1 (1)	0
Cough	1 (1)	0	0	1 (1)
Epistaxis	1 (1)	0	1 (1)	0
Granuloma NOS	1 (1)	0	1 (1)	0
Haematemesis	1 (1)	0	0	1 (1)
Hernia repair NOS	1 (1)	0	1 (1)	0
Hypertension NOS	1 (1)	0	1 (1)	0
Neck pain	1 (1)	0	0	1 (1)
Nephrolithiasis	1 (1)	0	0	1 (1)
Neurological disorder NOS	1 (1)	0	1 (1)	0
Open fracture	1 (1)	1 (1)	0	0
Pain in limb	1 (1)	0	0	1 (1)
Phlebothrombosis	1 (1)	0	1 (1)	0
Pyrexia	1 (1)	0	0	1 (1)
Rigors	1 (1)	0	0	1 (1)
Sinus operation NOS	1 (1)	0	1 (1)	0
Vomiting in pregnancy	1 (1)	0	0	1 (1)

NR = not related; R = related; NS = not specified.

Program: T0324.SAS, Version: 8.2, Datetime: 07FEB07:09:48

Table 14.3.2: Deaths and other serious and significant adverse events (excluding infections)

Preferred term	n	Severity		
		Mild	Moderate	Severe
Total	28 (14)	1 (1)	11 (8)	16 (9)

NR = not related; R = related; NS = not specified.

Program: T0324.SAS, Version: 8.2, Datetime: 07FEB07:09:48

Table 14.3.3.1: Summary of infusion site reactions in 6-monthly intervals - by diagnosis of PAD, age group and prior therapy

	Stage 1			Stage 2						
	Pre-Subgam	0-6 months (a)	>6-12 months (b)	>12-18 months	>18-24 months	>24-30 months	>30-36 months	>36-42 months	>42-48 months	>48-54 months
**All patients**										
median BMI = 24.4										
< median BMI	4	27	1	2	1	0	3	0	0	0
>= median BMI	0	32	6	4	1	2	5	0	0	0
**By diagnosis of PAD**										
CVID/XLA (median BMI = 26.5)										
< median BMI	1	31	6	2	0	0	1	0	0	0
>= median BMI	0	19	1	2	1	2	4	0	0	0
Other (median BMI = 21.9)										
< median BMI	3	3	0	2	1	0	0	0	0	0
>= median BMI	0	6	0	0	0	0	3	0	0	0
**By age group**										
Adults (median BMI = 26.6)										
< median BMI	0	24	5	2	0	0	1	0	0	0
>= median BMI	0	20	1	2	0	2	4	0	0	0
Teenagers (median BMI = 24.4)										
< median BMI	0	10	0	0	0	0	0	0	0	0
>= median BMI	0	1	1	0	1	0	0	0	0	0

(a) This interval lasts approximately 6 months, depending on the number of infusions received by the patient during Stage 1.
(b) This interval lasts approximately 6 months, from the first infusion in Stage 2 until 12 months after the first infusion of Subgam in Stage 1.
The calculation of median BMI includes all patients participating in the study, whether or not they reported an infusion site reaction.
For those patients who had an infusion site reaction, the mean weighted BMI for each site reaction is calculated for each patient.
Otherwise, for those patients who did not have an infusion site reaction, the BMI for each patient is the mean of all values recorded for that patient during the study.

Program: T0326.SAS, Version: 8.2, Datetime: 07FEB07:09:48

Table 14.3.3.1: Summary of infusion site reactions in 6-monthly intervals - by diagnosis of PAD, age group and prior therapy

	Stage 1		Stage 2							
	Pre-Subgam	0-6 months (a)	>6-12 months (b)	>12-18 months	>18-24 months	>24-30 months	>30-36 months	>36-42 months	>42-48 months	>48-54 months
Children				median BMI = 16.0						
< median BMI	3	3	0	2	0	0	0	0	0	0
>= median BMI	1	1	0	0	1	0	3	0	0	0

(a) This interval lasts approximately 6 months, depending on the number of infusions received by the patient during Stage 1.
(b) This interval lasts approximately 6 months, from the first infusion in Stage 2 until 12 months after the first infusion of Subgam in Stage 1.
The calculation of median BMI includes all patients participating in the study, whether or not they reported an infusion site reaction.
For those patients who had an infusion site reaction, the mean weighted BMI for each site reaction is calculated for each patient.
Otherwise, for those patients who did not have an infusion site reaction, the BMI for each patient is the mean of all values recorded for that patient during the study.

Program: T0326.SAS, Version: 8.2, Datetime: 07FEB07:09:48

Table 14.3.3.1: Summary of infusion site reactions in 6-monthly intervals - by diagnosis of PAD, age group and prior therapy

	Stage 1		Stage 2							
	Pre-Subgam	0-6 months (a)	>6-12 months (b)	>12-18 months	>18-24 months	>24-30 months	>30-36 months	>36-42 months	>42-48 months	>48-54 months
By prior therapy										
median BMI = 24.6										
IVIG										
< median BMI	0	26	2	0	1	0	3	0	0	0
>= median BMI	0	19	5	4	1	1	5	0	0	0
median BMI = 16.0										
SCIG										
< median BMI	3	3	0	2	0	0	0	0	0	0
>= median BMI	1	11	0	0	0	1	0	0	0	0

(a) This interval lasts approximately 6 months, depending on the number of infusions received by the patient during Stage 1.
(b) This interval lasts approximately 6 months, from the first infusion in Stage 2 until 12 months after the first infusion of Subgam in Stage 1.
The calculation of median BMI includes all patients participating in the study, whether or not they reported an infusion site reaction.
For those patients who had an infusion site reaction, the mean weighted BMI for each site reaction is calculated for each patient.
Otherwise, for those patients who did not have an infusion site reaction, the BMI for each patient is the mean of all values recorded for that patient during the study.

Figure 14.3.3.2 Summary of infusion site reactions in 6-monthly intervals – by Diagnosis of PAD, all patients

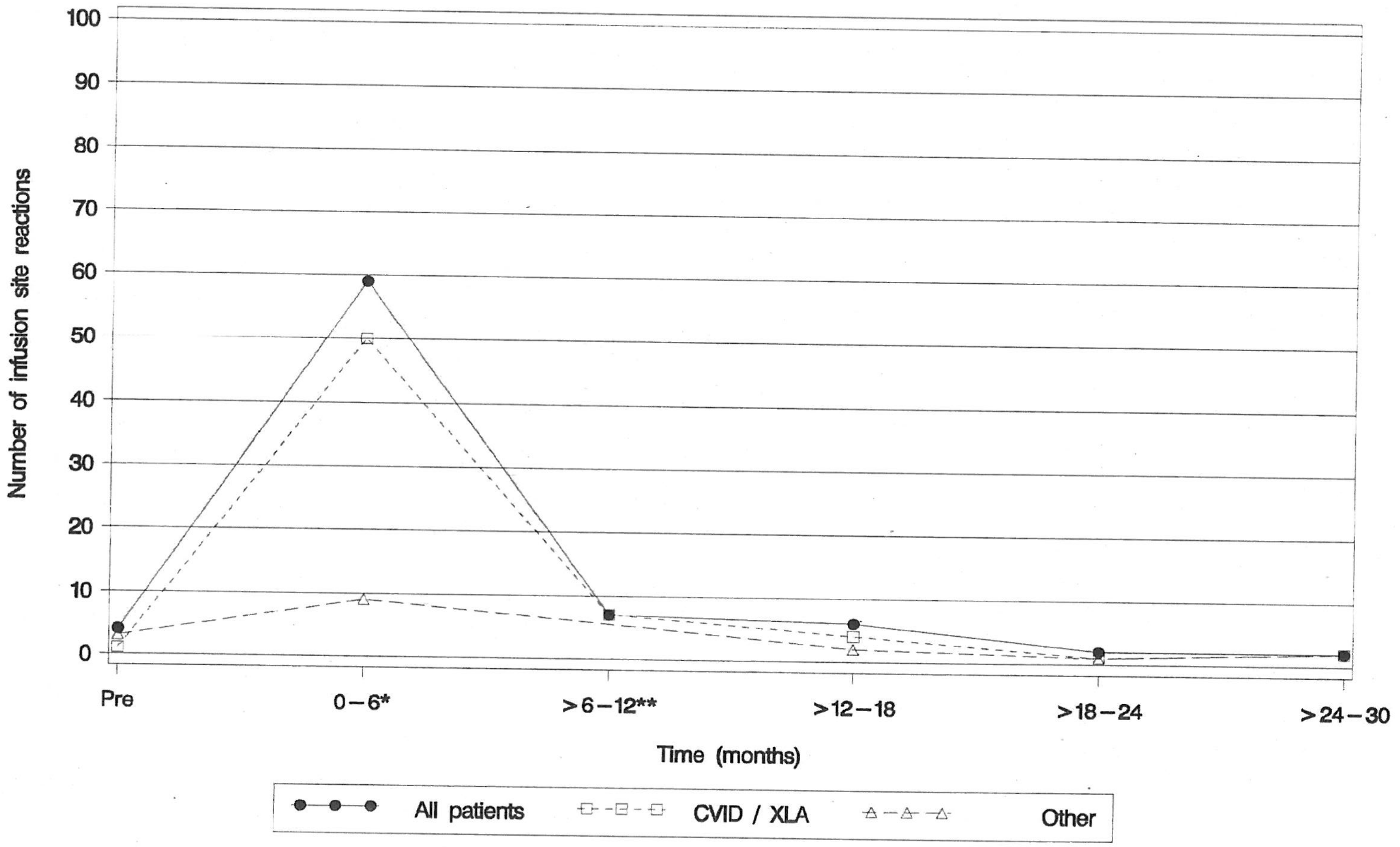

* This interval lasts approximately 6 months, depending on the number of infusions received by the patient during Stage 1, and contains post-Subgam data only

** This interval lasts approximately 6 months, from the first infusion in Stage 2 until 12 months after the first infusion of Subgam in Stage 1

Program: T0327.SAS, Version: 8.2, Datetime: 07FEB2007:09:47

Figure 14.3.3.3 Summary of infusion site reactions in 6-monthly intervals – by age group, all patients

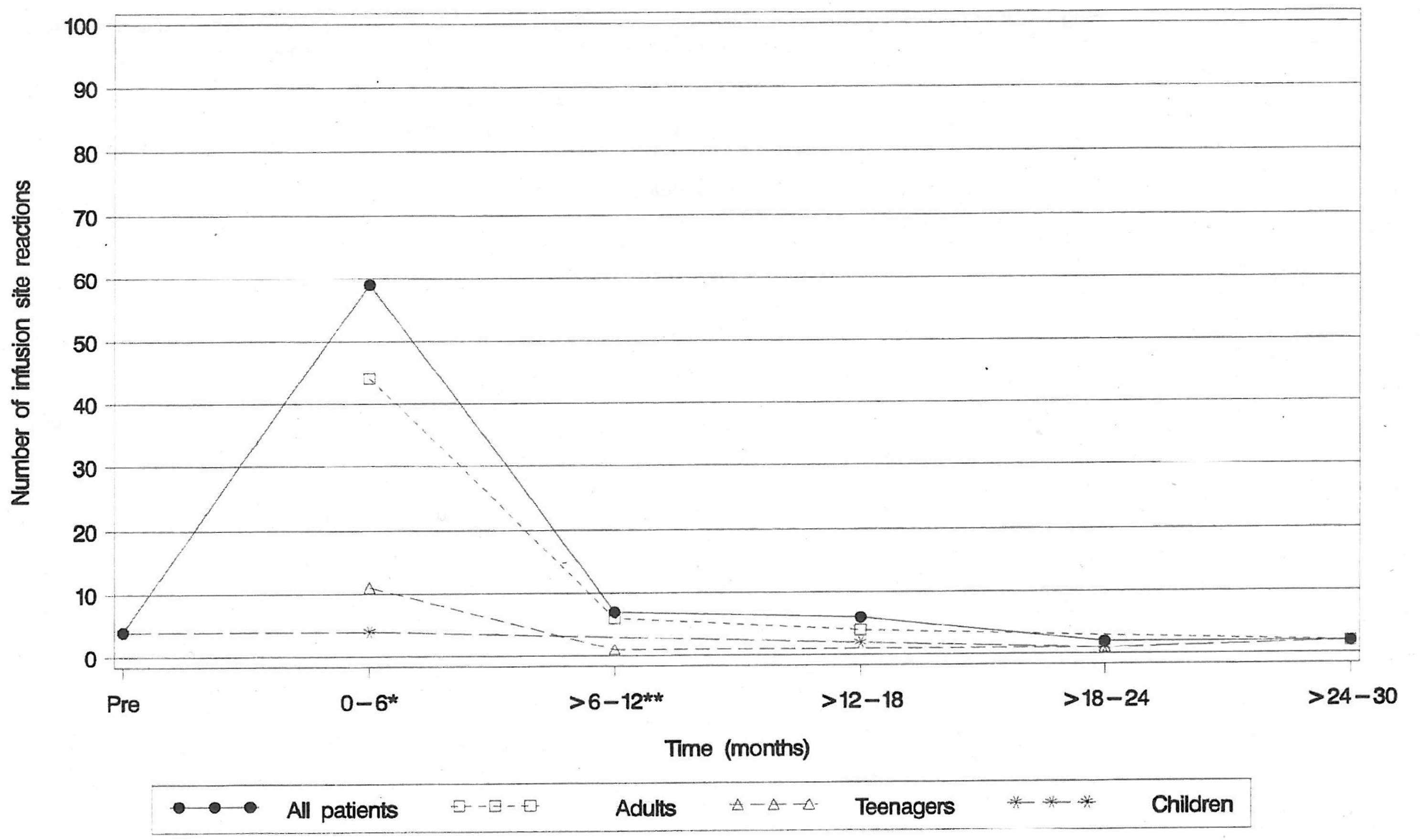

* This interval lasts approximately 6 months, depending on the number of infusions received by the patient during Stage 1, and contains post-Subgam data only

** This interval lasts approximately 6 months, from the first infusion in Stage 2 until 12 months after the first infusion of Subgam in Stage 1

Program: T0328.SAS, Version: 8.2, Datetime: 07FEB2007:09:47

Figure 14.3.3.4 Summary of infusion site reactions in 6–monthly intervals – by prior therapy, all patients

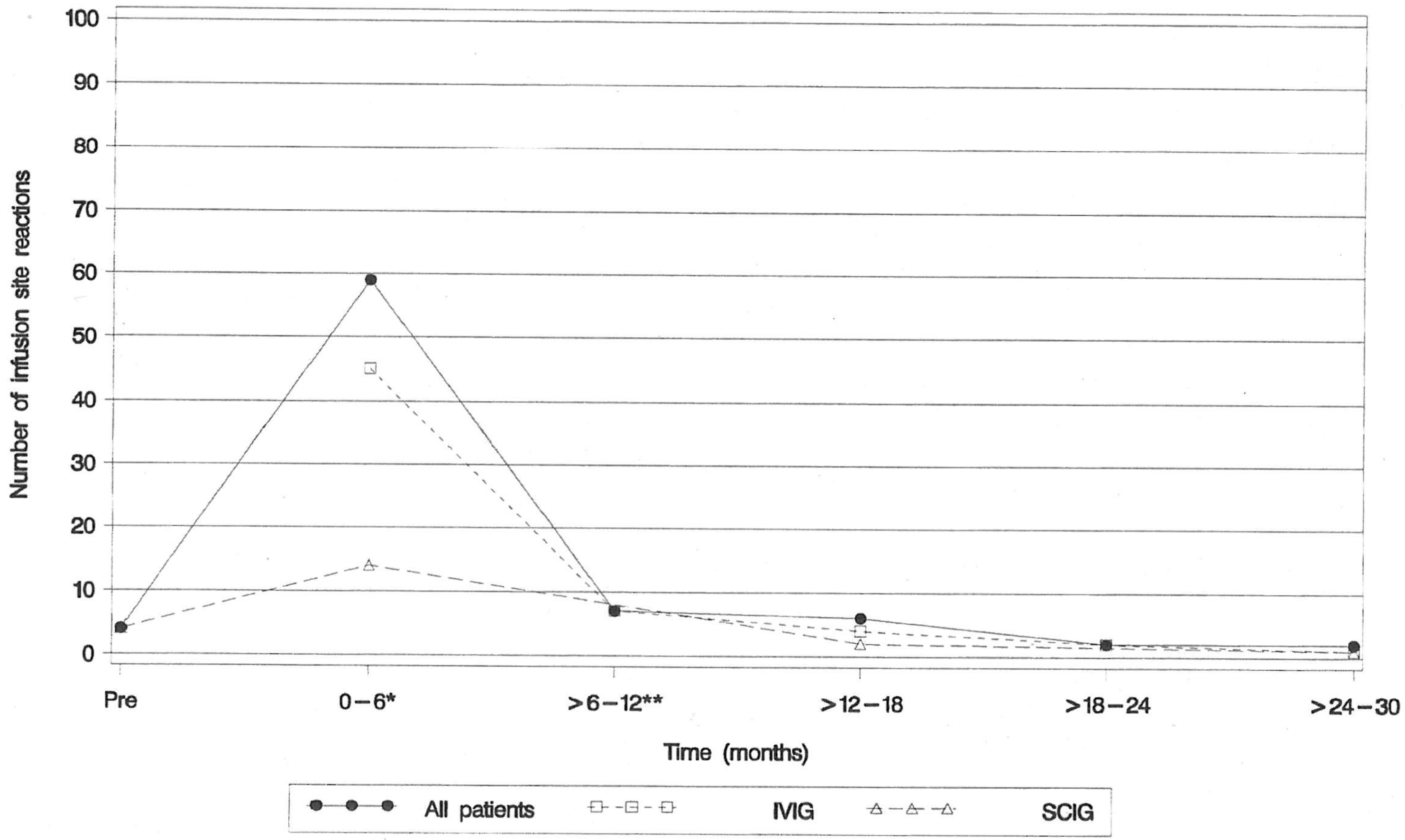

* This interval lasts approximately 6 months, depending on the number of infusions received by the patient during Stage 1, and contains post–Subgam data only
** This interval lasts approximately 6 months, from the first infusion in Stage 2 until 12 months after the first infusion of Subgam in Stage 1

Program: T0329.SAS, Version: 8.2, Datetime: 07FEB2007:09:47

Table 14.3.3.5: Summary of infusion site reactions by preferred term and severity - Pre-Subgam (Pre-study - Infusion 3) - all patients
Number of events (number of patients)

Preferred term	Median BMI = 23.4	n	Severity		
			Mild	Moderate	Severe
Total number of infusions for study		147			
Infusion site pain	>= median	0 (0)	0 (0)	0 (0)	0 (0)
	< median	4 (2)	1 (1)	3 (1)	0 (0)
	Total	4 (2)	1 (1)	3 (1)	0 (0)
Total	Total >= median	0 (0)	0 (0)	0 (0)	0 (0)
	Total < median	4 (2)	1 (1)	3 (1)	0 (0)
	Grand total	4 (2)	1 (1)	3 (1)	0 (0)

The calculation of median BMI includes all patients participating in the study, whether or not they reported an infusion site reaction.
For those patients who had an infusion site reaction, the mean weighted BMI for each site reaction is calculated for each patient.
Otherwise, for those patients who did not have an infusion site reaction, the BMI for each patient is the mean of all values recorded for that patient during the study.

Program: T0337.TEM, Version: 8.2, Datetime: 07FEB07:09:48

Table 14.3.3.6: Summary of infusion site reactions by preferred term and severity - Post-Subgam (Infusion 4-Last Subgam Infusion) - all patients
Number of events (number of patients)

Preferred term	Median BMI = 24.0	n	Severity		
			Mild	Moderate	Severe
Total number of infusions for study		7,346			
Infusion site burning	>= median	0 (0)	0 (0)	0 (0)	0 (0)
	< median	1 (1)	1 (1)	0 (0)	0 (0)
	Total	1 (1)	1 (1)	0 (0)	0 (0)
Infusion site erythema	>= median	2 (2)	2 (2)	0 (0)	0 (0)
	< median	10 (4)	9 (4)	1 (1)	0 (0)
	Total	12 (6)	11 (6)	1 (1)	0 (0)
Infusion site induration	>= median	2 (1)	2 (1)	0 (0)	0 (0)
	< median	0 (0)	0 (0)	0 (0)	0 (0)
	Total	2 (1)	2 (1)	0 (0)	0 (0)
Infusion site infection	>= median	2 (1)	2 (1)	0 (0)	0 (0)
	< median	0 (0)	0 (0)	0 (0)	0 (0)
	Total	2 (1)	2 (1)	0 (0)	0 (0)

The calculation of median BMI includes all patients participating in the study, whether or not they reported an infusion site reaction.
For those patients who had an infusion site reaction, the mean weighted BMI for each site reaction is calculated for each patient.
Otherwise, for those patients who did not have an infusion site reaction, the BMI for each patient is the mean of all values recorded for that patient during the study.

Program: T0338.TEM, Version: 8.2, Datetime: 07FEB07:09:48

Table 14.3.3.6: Summary of infusion site reactions by preferred term and severity - Post-Subgam (Infusion 4-Last Subgam Infusion) - all patients
Number of events (number of patients)

			Severity		
Preferred term	Median BMI = 24.0	n	Mild	Moderate	Severe
Infusion site inflammation	>= median	10 (7)	10 (7)	0 (0)	0 (0)
	< median	6 (2)	5 (1)	1 (1)	0 (0)
	Total	16 (9)	15 (8)	1 (1)	0 (0)
Infusion site pain	>= median	3 (3)	3 (3)	0 (0)	0 (0)
	< median	2 (2)	2 (2)	0 (0)	0 (0)
	Total	5 (5)	5 (5)	0 (0)	0 (0)
Infusion site pruritus	>= median	4 (3)	4 (3)	0 (0)	0 (0)
	< median	0 (0)	0 (0)	0 (0)	0 (0)
	Total	4 (3)	4 (3)	0 (0)	0 (0)
Infusion site swelling	>= median	6 (3)	5 (3)	1 (1)	0 (0)
	< median	8 (4)	6 (4)	2 (2)	0 (0)
	Total	14 (7)	11 (7)	3 (3)	0 (0)
Infusion site tenderness	>= median	3 (3)	1 (1)	2 (2)	0 (0)
	< median	0 (0)	0 (0)	0 (0)	0 (0)
	Total	3 (3)	1 (1)	2 (2)	0 (0)

The calculation of median BMI includes all patients participating in the study, whether or not they reported an infusion site reaction.
For those patients who had an infusion site reaction, the mean weighted BMI for each site reaction is calculated for each patient.
Otherwise, for those patients who did not have an infusion site reaction, the BMI for each patient is the mean of all values recorded for that patient during the study.

Program: T0338.TEM, Version: 8.2, Datetime: 07FEB07:09:48

Table 14.3.3.6: Summary of infusion site reactions by preferred term and severity - Post-Subgam (Infusion 4-Last Subgam Infusion) - all patients
Number of events (number of patients)

			Severity		
Preferred term	Median BMI = 24.0	n	Mild	Moderate	Severe
Injection site bruising	>= median	5 (2)	5 (2)	0 (0)	0 (0)
	< median	0 (0)	0 (0)	0 (0)	0 (0)
	Total	5 (2)	5 (2)	0 (0)	0 (0)
Injection site discomfort	>= median	0 (0)	0 (0)	0 (0)	0 (0)
	< median	1 (1)	1 (1)	0 (0)	0 (0)
	Total	1 (1)	1 (1)	0 (0)	0 (0)
Injection site haemorrhage	>= median	0 (0)	0 (0)	0 (0)	0 (0)
	< median	1 (1)	0 (0)	1 (1)	0 (0)
	Total	1 (1)	0 (0)	1 (1)	0 (0)
Injection site paraesthesia	>= median	1 (1)	1 (1)	0 (0)	0 (0)
	< median	1 (1)	1 (1)	0 (0)	0 (0)
	Total	2 (2)	2 (2)	0 (0)	0 (0)
Injection site rash	>= median	11 (3)	11 (3)	0 (0)	0 (0)
	< median	1 (1)	1 (1)	0 (0)	0 (0)
	Total	12 (4)	12 (4)	0 (0)	0 (0)

The calculation of median BMI includes all patients participating in the study, whether or not they reported an infusion site reaction.
For those patients who had an infusion site reaction, the mean weighted BMI for each site reaction is calculated for each patient.
Otherwise, for those patients who did not have an infusion site reaction, the BMI for each patient is the mean of all values recorded for that patient during the study.

Table 14.3.3.6: Summary of infusion site reactions by preferred term and severity - Post-Subgam (Infusion 4-Last Subgam Infusion) - all patients
Number of events (number of patients)

Preferred term	Median BMI = 24.0	n	Severity		
			Mild	Moderate	Severe
Injection site stinging	>= median	2 (1)	2 (1)	0 (0)	0 (0)
	< median	0 (0)	0 (0)	0 (0)	0 (0)
	Total	2 (1)	2 (1)	0 (0)	0 (0)
Injection site urticaria	>= median	0 (0)	0 (0)	0 (0)	0 (0)
	< median	2 (1)	0 (0)	2 (1)	0 (0)
	Total	2 (1)	0 (0)	2 (1)	0 (0)
Total	Total >= median	51 (15)	48 (14)	3 (2)	0 (0)
	Total < median	33 (10)	26 (9)	7 (4)	0 (0)
	Grand total	84 (25)	74 (23)	10 (6)	0 (0)

The calculation of median BMI includes all patients participating in the study, whether or not they reported an infusion site reaction.
For those patients who had an infusion site reaction, the mean weighted BMI for each site reaction is calculated for each patient.
Otherwise, for those patients who did not have an infusion site reaction, the BMI for each patient is the mean of all values recorded for that patient during the study.

Table 14.3.3.7a: Summary of infusion site reactions by preferred term and severity - all patients
0 - 6 months Post-Subgam
Number of events (number of patients)

Preferred term	Median BMI = 23.4	n	Severity: Mild	Severity: Moderate	Severity: Severe
Total number of infusions for period		1,391			
Infusion site burning	>= median	0 (0)	0 (0)	0 (0)	0 (0)
	< median	1 (1)	1 (1)	0 (0)	0 (0)
	Total	1 (1)	1 (1)	0 (0)	0 (0)
Infusion site erythema	>= median	2 (2)	2 (2)	0 (0)	0 (0)
	< median	7 (2)	6 (2)	1 (1)	0 (0)
	Total	9 (4)	8 (4)	1 (1)	0 (0)
Infusion site induration	>= median	2 (1)	2 (1)	0 (0)	0 (0)
	< median	0 (0)	0 (0)	0 (0)	0 (0)
	Total	2 (1)	2 (1)	0 (0)	0 (0)
Infusion site inflammation	>= median	5 (5)	5 (5)	0 (0)	0 (0)
	< median	5 (1)	5 (1)	0 (0)	0 (0)
	Total	10 (6)	10 (6)	0 (0)	0 (0)

The calculation of median BMI includes all patients participating in the study within the time period, whether or not they reported an infusion site reaction. For those patients who had an infusion site reaction within the time period, the mean weighted BMI for each site reaction is calculated for each patient. Otherwise, for those patients who did not have an infusion site reaction within the time period, the BMI for each patient is the mean of most relevant calculated BMIs for that patient.

Program: T0049.TEM, Version: 8.2, Datetime: 07FEB07:09:48

Table 14.3.3.7a: Summary of infusion site reactions by preferred term and severity - all patients
0 - 6 months Post-Subgam
Number of events (number of patients)

Preferred term	Median BMI = 23.4	n	Severity: Mild	Severity: Moderate	Severity: Severe
Infusion site pain	>= median	0 (0)	0 (0)	0 (0)	0 (0)
	< median	1 (1)	1 (1)	0 (0)	0 (0)
	Total	1 (1)	1 (1)	0 (0)	0 (0)
Infusion site pruritus	>= median	3 (2)	3 (2)	0 (0)	0 (0)
	< median	0 (0)	0 (0)	0 (0)	0 (0)
	Total	3 (2)	3 (2)	0 (0)	0 (0)
Infusion site swelling	>= median	4 (2)	3 (2)	1 (1)	0 (0)
	< median	7 (3)	5 (3)	2 (2)	0 (0)
	Total	11 (5)	8 (5)	3 (3)	0 (0)
Infusion site tenderness	>= median	2 (2)	1 (1)	1 (1)	0 (0)
	< median	0 (0)	0 (0)	0 (0)	0 (0)
	Total	2 (2)	1 (1)	1 (1)	0 (0)
Injection site bruising	>= median	3 (2)	3 (2)	0 (0)	0 (0)
	< median	0 (0)	0 (0)	0 (0)	0 (0)
	Total	3 (2)	3 (2)	0 (0)	0 (0)

The calculation of median BMI includes all patients participating in the study within the time period, whether or not they reported an infusion site reaction. For those patients who had an infusion site reaction within the time period, the mean weighted BMI for each site reaction is calculated for each patient. Otherwise, for those patients who did not have an infusion site reaction within the time period, the BMI for each patient is the mean of most relevant calculated BMIs for that patient.

Program: T0049.TEM, Version: 8.2, Datetime: 07FEB07:09:48

Table 14.3.3.7a: Summary of infusion site reactions by preferred term and severity - all patients
0 - 6 months Post-Subgam
Number of events (number of patients)

Preferred term	Median BMI = 23.4	n	Severity: Mild	Severity: Moderate	Severity: Severe
Injection site haemorrhage	>= median	0 (0)	0 (0)	0 (0)	0 (0)
	< median	1 (1)	0 (0)	1 (1)	0 (0)
	Total	1 (1)	0 (0)	1 (1)	0 (0)
Injection site paraesthesia	>= median	1 (1)	1 (1)	0 (0)	0 (0)
	< median	1 (1)	1 (1)	0 (0)	0 (0)
	Total	2 (2)	2 (2)	0 (0)	0 (0)
Injection site rash	>= median	11 (3)	11 (3)	0 (0)	0 (0)
	< median	1 (1)	1 (1)	0 (0)	0 (0)
	Total	12 (4)	12 (4)	0 (0)	0 (0)
Injection site urticaria	>= median	0 (0)	0 (0)	0 (0)	0 (0)
	< median	2 (1)	0 (0)	2 (1)	0 (0)
	Total	2 (1)	0 (0)	2 (1)	0 (0)
Total	Total >= median	33 (12)	31 (12)	2 (1)	0 (0)
	Total < median	26 (8)	20 (7)	6 (3)	0 (0)
	Grand total	59 (20)	51 (19)	8 (4)	0 (0)

The calculation of median BMI includes all patients participating in the study within the time period, whether or not they reported an infusion site reaction. For those patients who had an infusion site reaction within the time period, the mean weighted BMI for each site reaction is calculated for each patient. Otherwise, for those patients who did not have an infusion site reaction within the time period, the BMI for each patient is the mean of most relevant calculated BMIs for that patient.

Program: T0049.TEM, Version: 8.2, Datetime: 07FEB07:09:48

Table 14.3.3.7b: Summary of infusion site reactions by preferred term and severity - all patients
>6 - 12 months Post-Subgam
Number of events (number of patients)

Preferred term	Median BMI = 23.4	n	Severity		
			Mild	Moderate	Severe
Total number of infusions for period		960			
Infusion site inflammation	>= median	1 (1)	1 (1)	0 (0)	0 (0)
	< median	0 (0)	0 (0)	0 (0)	0 (0)
	Total	1 (1)	1 (1)	0 (0)	0 (0)
Infusion site pain	>= median	1 (1)	1 (1)	0 (0)	0 (0)
	< median	1 (1)	1 (1)	0 (0)	0 (0)
	Total	2 (2)	2 (2)	0 (0)	0 (0)
Infusion site swelling	>= median	2 (2)	2 (2)	0 (0)	0 (0)
	< median	0 (0)	0 (0)	0 (0)	0 (0)
	Total	2 (2)	2 (2)	0 (0)	0 (0)
Injection site bruising	>= median	2 (1)	2 (1)	0 (0)	0 (0)
	< median	0 (0)	0 (0)	0 (0)	0 (0)
	Total	2 (1)	2 (1)	0 (0)	0 (0)

The calculation of median BMI includes all patients participating in the study within the time period, whether or not they reported an infusion site reaction. For those patients who had an infusion site reaction within the time period, the mean weighted BMI for each site reaction is calculated for each patient. Otherwise, for those patients who did not have an infusion site reaction within the time period, the BMI for each patient is the mean of most relevant calculated BMIs for that patient.

Program: T0050.TEM, Version: 8.2, Datetime: 07FEB07:09:48

Table 14.3.3.7b: Summary of infusion site reactions by preferred term and severity - all patients
>6 - 12 months Post-Subgam
Number of events (number of patients)

Preferred term	Median BMI = 23.4	n	Severity		
			Mild	Moderate	Severe
Total	Total >= median	6 (3)	6 (3)	0 (0)	0 (0)
	Total < median	1 (1)	1 (1)	0 (0)	0 (0)
	Grand total	7 (4)	7 (4)	0 (0)	0 (0)

The calculation of median BMI includes all patients participating in the study within the time period, whether or not they reported an infusion site reaction. For those patients who had an infusion site reaction within the time period, the mean weighted BMI for each site reaction is calculated for each patient. Otherwise, for those patients who did not have an infusion site reaction within the time period, the BMI for each patient is the mean of most relevant calculated BMIs for that patient.

Program: T0050.TEM, Version: 8.2, Datetime: 07FEB07:09:48

Table 14.3.3.7c: Summary of infusion site reactions by preferred term and severity - all patients
>12 - 18 months Post-Subgam
Number of events (number of patients)

Preferred term	Median BMI = 23.0	n	Severity		
			Mild	Moderate	Severe
Total number of infusions for period		1,065			
Infusion site erythema	>= median	0 (0)	0 (0)	0 (0)	0 (0)
	< median	2 (1)	2 (1)	0 (0)	0 (0)
	Total	2 (1)	2 (1)	0 (0)	0 (0)
Infusion site infection	>= median	1 (1)	1 (1)	0 (0)	0 (0)
	< median	0 (0)	0 (0)	0 (0)	0 (0)
	Total	1 (1)	1 (1)	0 (0)	0 (0)
Infusion site inflammation	>= median	2 (2)	2 (2)	0 (0)	0 (0)
	< median	0 (0)	0 (0)	0 (0)	0 (0)
	Total	2 (2)	2 (2)	0 (0)	0 (0)
Infusion site pain	>= median	1 (1)	1 (1)	0 (0)	0 (0)
	< median	0 (0)	0 (0)	0 (0)	0 (0)
	Total	1 (1)	1 (1)	0 (0)	0 (0)

The calculation of median BMI includes all patients participating in the study within the time period, whether or not they reported an infusion site reaction. For those patients who had an infusion site reaction within the time period, the mean weighted BMI for each site reaction is calculated for each patient. Otherwise, for those patients who did not have an infusion site reaction within the time period, the BMI for each patient is the mean of most relevant calculated BMIs for that patient.

Program: T0051.TEM, Version: 8.2, Datetime: 07FEB07:09:48

Table 14.3.3.7c: Summary of infusion site reactions by preferred term and severity - all patients
>12 - 18 months Post-Subgam
Number of events (number of patients)

			Severity		
Preferred term	Median BMI = 23.0	n	Mild	Moderate	Severe
Total	Total >= median	4 (3)	4 (3)	0 (0)	0 (0)
	Total < median	2 (1)	2 (1)	0 (0)	0 (0)
	Grand total	6 (4)	6 (4)	0 (0)	0 (0)

The calculation of median BMI includes all patients participating in the study within the time period, whether or not they reported an infusion site reaction. For those patients who had an infusion site reaction within the time period, the mean weighted BMI for each site reaction is calculated for each patient. Otherwise, for those patients who did not have an infusion site reaction within the time period, the BMI for each patient is the mean of most relevant calculated BMIs for that patient.

Program: T0051.TEM, Version: 8.2, Datetime: 07FEB07:09:48

Table 14.3.3.7d: Summary of infusion site reactions by preferred term and severity - all patients
>18 - 24 months Post-Subgam
Number of events (number of patients)

Preferred term	Median BMI = 23.0	n	Severity: Mild	Severity: Moderate	Severity: Severe
Total number of infusions for period		1,047			
Infusion site erythema	>= median	0 (0)	0 (0)	0 (0)	0 (0)
	< median	1 (1)	1 (1)	0 (0)	0 (0)
	Total	1 (1)	1 (1)	0 (0)	0 (0)
Infusion site inflammation	>= median	1 (1)	1 (1)	0 (0)	0 (0)
	< median	0 (0)	0 (0)	0 (0)	0 (0)
	Total	1 (1)	1 (1)	0 (0)	0 (0)
Total	Total >= median	1 (1)	1 (1)	0 (0)	0 (0)
	Total < median	1 (1)	1 (1)	0 (0)	0 (0)
	Grand total	2 (2)	2 (2)	0 (0)	0 (0)

The calculation of median BMI includes all patients participating in the study within the time period, whether or not they reported an infusion site reaction. For those patients who had an infusion site reaction within the time period, the mean weighted BMI for each site reaction is calculated for each patient. Otherwise, for those patients who did not have an infusion site reaction within the time period, the BMI for each patient is the mean of most relevant calculated BMIs for that patient.

Program: T0052.TEM, Version: 8.2, Datetime: 07FEB07:09:48

Table 14.3.3.7e: Summary of infusion site reactions by preferred term and severity - all patients
>24 - 30 months Post-Subgam
Number of events (number of patients)

Preferred term	Median BMI = 23.2	n	Severity: Mild	Severity: Moderate	Severity: Severe
Total number of infusions for period		1,039			
Infusion site tenderness	>= median	1 (1)	0 (0)	1 (1)	0 (0)
	< median	0 (0)	0 (0)	0 (0)	0 (0)
	Total	1 (1)	0 (0)	1 (1)	0 (0)
Injection site stinging	>= median	1 (1)	1 (1)	0 (0)	0 (0)
	< median	0 (0)	0 (0)	0 (0)	0 (0)
	Total	1 (1)	1 (1)	0 (0)	0 (0)
Total	Total >= median	2 (2)	1 (1)	1 (1)	0 (0)
	Total < median	0 (0)	0 (0)	0 (0)	0 (0)
	Grand total	2 (2)	1 (1)	1 (1)	0 (0)

The calculation of median BMI includes all patients participating in the study within the time period, whether or not they reported an infusion site reaction. For those patients who had an infusion site reaction within the time period, the mean weighted BMI for each site reaction is calculated for each patient. Otherwise, for those patients who did not have an infusion site reaction within the time period, the BMI for each patient is the mean of most relevant calculated BMIs for that patient.

Program: T0053.TEM, Version: 8.2, Datetime: 07FEB07:09:48

Table 14.3.3.7f: Summary of infusion site reactions by preferred term and severity - all patients
>30 - 36 months Post-Subgam
Number of events (number of patients)

Preferred term	Median BMI = 23.8	n	Severity: Mild	Severity: Moderate	Severity: Severe
Total number of infusions for period		879			
Infusion site infection	>= median	1 (1)	1 (1)	0 (0)	0 (0)
	< median	0 (0)	0 (0)	0 (0)	0 (0)
	Total	1 (1)	1 (1)	0 (0)	0 (0)
Infusion site inflammation	>= median	1 (1)	1 (1)	0 (0)	0 (0)
	< median	1 (1)	0 (0)	1 (1)	0 (0)
	Total	2 (2)	1 (1)	1 (1)	0 (0)
Infusion site pain	>= median	1 (1)	1 (1)	0 (0)	0 (0)
	< median	0 (0)	0 (0)	0 (0)	0 (0)
	Total	1 (1)	1 (1)	0 (0)	0 (0)
Infusion site pruritus	>= median	1 (1)	1 (1)	0 (0)	0 (0)
	< median	0 (0)	0 (0)	0 (0)	0 (0)
	Total	1 (1)	1 (1)	0 (0)	0 (0)

The calculation of median BMI includes all patients participating in the study within the time period, whether or not they reported an infusion site reaction. For those patients who had an infusion site reaction within the time period, the mean weighted BMI for each site reaction is calculated for each patient. Otherwise, for those patients who did not have an infusion site reaction within the time period, the BMI for each patient is the mean of most relevant calculated BMIs for that patient.

Program: T0054.TEM, Version: 8.2, Datetime: 07FEB07:09:48

Table 14.3.3.7f: Summary of infusion site reactions by preferred term and severity - all patients
>30 - 36 months Post-Subgam
Number of events (number of patients)

Preferred term	Median BMI = 23.8	n	Severity: Mild	Severity: Moderate	Severity: Severe
Infusion site swelling	>= median	0 (0)	0 (0)	0 (0)	0 (0)
	< median	1 (1)	1 (1)	0 (0)	0 (0)
	Total	1 (1)	1 (1)	0 (0)	0 (0)
Injection site discomfort	>= median	0 (0)	0 (0)	0 (0)	0 (0)
	< median	1 (1)	1 (1)	0 (0)	0 (0)
	Total	1 (1)	1 (1)	0 (0)	0 (0)
Injection site stinging	>= median	1 (1)	1 (1)	0 (0)	0 (0)
	< median	0 (0)	0 (0)	0 (0)	0 (0)
	Total	1 (1)	1 (1)	0 (0)	0 (0)
Total	Total >= median	5 (4)	5 (4)	0 (0)	0 (0)
	Total < median	3 (1)	2 (1)	1 (1)	0 (0)
	Grand total	8 (5)	7 (5)	1 (1)	0 (0)

The calculation of median BMI includes all patients participating in the study within the time period, whether or not they reported an infusion site reaction. For those patients who had an infusion site reaction within the time period, the mean weighted BMI for each site reaction is calculated for each patient. Otherwise, for those patients who did not have an infusion site reaction within the time period, the BMI for each patient is the mean of most relevant calculated BMIs for that patient.

Program: T0054.TEM, Version: 8.2, Datetime: 07FEB07:09:48

Table 14.3.3.7g: Summary of infusion site reactions by preferred term and severity - all patients
>36 - 42 months Post-Subgam
Number of events (number of patients)

```
/*************************************************************************************/
/*                                                                                  */
/*                                                                                  */
/*  No observations met the criteria of the current report.                         */
/*                                                                                  */
/*                                                                                  */
/*************************************************************************************/
```

The calculation of median BMI includes all patients participating in the study within the time period, whether or not they reported an infusion site reaction. For those patients who had an infusion site reaction within the time period, the mean weighted BMI for each site reaction is calculated for each patient. Otherwise, for those patients who did not have an infusion site reaction within the time period, the BMI for each patient is the mean of most relevant calculated BMIs for that patient.

Program: T0055.TEM, Version: 8.2, Datetime: 07FEB07:09:48

Table 14.3.3.7h: Summary of infusion site reactions by preferred term and severity - all patients
>42 - 48 months Post-Subgam
Number of events (number of patients)

```
/*****************************************************************************************/
/*                                                                                       */
/*                                                                                       */
/*   No observations met the criteria of the current report.                             */
/*                                                                                       */
/*                                                                                       */
/*                                                                                       */
/*****************************************************************************************/
```

The calculation of median BMI includes all patients participating in the study within the time period, whether or not they reported an infusion site reaction. For those patients who had an infusion site reaction within the time period, the mean weighted BMI for each site reaction is calculated for each patient. Otherwise, for those patients who did not have an infusion site reaction within the time period, the BMI for each patient is the mean of most relevant calculated BMIs for that patient.

Program: T0056.TEM, Version: 8.2, Datetime: 07FEB07:09:48

Table 14.3.3.7i: Summary of infusion site reactions by preferred term and severity - all patients
>48 - 54 months Post-Subgam
Number of events (number of patients)

```
/**************************************************************************************/
/*                                                                                    */
/*                                                                                    */
/*  No observations met the criteria of the current report.                           */
/*                                                                                    */
/*                                                                                    */
/*                                                                                    */
/**************************************************************************************/
```

The calculation of median BMI includes all patients participating in the study within the time period, whether or not they reported an infusion site reaction. For those patients who had an infusion site reaction within the time period, the mean weighted BMI for each site reaction is calculated for each patient. Otherwise, for those patients who did not have an infusion site reaction within the time period, the BMI for each patient is the mean of most relevant calculated BMIs for that patient.

Program: T0057.TEM, Version: 8.2, Datetime: 07FEB07:09:48

Table 14.3.3.8a: Summary of infusion site reactions by preferred term and severity - by diagnosis of PAD
0 - 6 months Post-Subgam
Number of events (number of patients)

Preferred term	Diagnosis of PAD	Median BMI = 23.4	n	Severity: Mild	Moderate	Severe
Total number of infusions for period	.		1,391			
Infusion site burning	CVID/XLA	>= median	0 (0)	0 (0)	0 (0)	0 (0)
		< median	1 (1)	1 (1)	0 (0)	0 (0)
	Other	>= median	0 (0)	0 (0)	0 (0)	0 (0)
		< median	0 (0)	0 (0)	0 (0)	0 (0)
Infusion site erythema	CVID/XLA	>= median	2 (2)	2 (2)	0 (0)	0 (0)
		< median	7 (2)	6 (2)	1 (1)	0 (0)
	Other	>= median	0 (0)	0 (0)	0 (0)	0 (0)
		< median	0 (0)	0 (0)	0 (0)	0 (0)
Infusion site induration	CVID/XLA	>= median	2 (1)	2 (1)	0 (0)	0 (0)
		< median	0 (0)	0 (0)	0 (0)	0 (0)
	Other	>= median	0 (0)	0 (0)	0 (0)	0 (0)
		< median	0 (0)	0 (0)	0 (0)	0 (0)
Infusion site inflammation	CVID/XLA	>= median	5 (5)	5 (5)	0 (0)	0 (0)
		< median	5 (1)	5 (1)	0 (0)	0 (0)
	Other	>= median	0 (0)	0 (0)	0 (0)	0 (0)
		< median	0 (0)	0 (0)	0 (0)	0 (0)

The calculation of median BMI includes all patients participating in the study within the time period, whether or not they reported an infusion site reaction. For those patients who had an infusion site reaction within the time period, the mean weighted BMI for each site reaction is calculated for each patient. Otherwise, for those patients who did not have an infusion site reaction within the time period, the BMI for each patient is the mean of most relevant calculated BMIs for that patient.

Program: T0058.TEM, Version: 8.2, Datetime: 07FEB07:09:48

Table 14.3.3.8a: Summary of infusion site reactions by preferred term and severity - by diagnosis of PAD
0 - 6 months Post-Subgam
Number of events (number of patients)

				Severity		
Preferred term	Diagnosis of PAD	Median BMI = 23.4	n	Mild	Moderate	Severe
Infusion site pain	CVID/XLA	>= median	0 (0)	0 (0)	0 (0)	0 (0)
		< median	1 (1)	1 (1)	0 (0)	0 (0)
	Other	>= median	0 (0)	0 (0)	0 (0)	0 (0)
		< median	0 (0)	0 (0)	0 (0)	0 (0)
Infusion site pruritus	CVID/XLA	>= median	2 (1)	2 (1)	0 (0)	0 (0)
		< median	0 (0)	0 (0)	0 (0)	0 (0)
	Other	>= median	1 (1)	1 (1)	0 (0)	0 (0)
		< median	0 (0)	0 (0)	0 (0)	0 (0)
Infusion site swelling	CVID/XLA	>= median	4 (2)	3 (2)	1 (1)	0 (0)
		< median	6 (2)	4 (2)	2 (2)	0 (0)
	Other	>= median	0 (0)	0 (0)	0 (0)	0 (0)
		< median	1 (1)	1 (1)	0 (0)	0 (0)
Infusion site tenderness	CVID/XLA	>= median	2 (2)	1 (1)	1 (1)	0 (0)
		< median	0 (0)	0 (0)	0 (0)	0 (0)
	Other	>= median	0 (0)	0 (0)	0 (0)	0 (0)
		< median	0 (0)	0 (0)	0 (0)	0 (0)

The calculation of median BMI includes all patients participating in the study within the time period, whether or not they reported an infusion site reaction. For those patients who had an infusion site reaction within the time period, the mean weighted BMI for each site reaction is calculated for each patient. Otherwise, for those patients who did not have an infusion site reaction within the time period, the BMI for each patient is the mean of most relevant calculated BMIs for that patient.

Program: T0058.TEM, Version: 8.2, Datetime: 07FEB07:09:48

Table 14.3.3.8a: Summary of infusion site reactions by preferred term and severity - by diagnosis of PAD
0 - 6 months Post-Subgam
Number of events (number of patients)

Preferred term	Diagnosis of PAD	Median BMI = 23.4	n	Severity: Mild	Severity: Moderate	Severity: Severe
Injection site bruising	CVID/XLA	>= median	2 (1)	2 (1)	0 (0)	0 (0)
		< median	0 (0)	0 (0)	0 (0)	0 (0)
	Other	>= median	1 (1)	1 (1)	0 (0)	0 (0)
		< median	0 (0)	0 (0)	0 (0)	0 (0)
Injection site haemorrhage	CVID/XLA	>= median	0 (0)	0 (0)	0 (0)	0 (0)
		< median	1 (1)	0 (0)	1 (1)	0 (0)
	Other	>= median	0 (0)	0 (0)	0 (0)	0 (0)
		< median	0 (0)	0 (0)	0 (0)	0 (0)
Injection site paraesthesia	CVID/XLA	>= median	1 (1)	1 (1)	0 (0)	0 (0)
		< median	1 (1)	1 (1)	0 (0)	0 (0)
	Other	>= median	0 (0)	0 (0)	0 (0)	0 (0)
		< median	0 (0)	0 (0)	0 (0)	0 (0)
Injection site rash	CVID/XLA	>= median	8 (1)	8 (1)	0 (0)	0 (0)
		< median	0 (0)	0 (0)	0 (0)	0 (0)
	Other	>= median	3 (2)	3 (2)	0 (0)	0 (0)
		< median	1 (1)	1 (1)	0 (0)	0 (0)

The calculation of median BMI includes all patients participating in the study within the time period, whether or not they reported an infusion site reaction. For those patients who had an infusion site reaction within the time period, the mean weighted BMI for each site reaction is calculated for each patient. Otherwise, for those patients who did not have an infusion site reaction within the time period, the BMI for each patient is the mean of most relevant calculated BMIs for that patient.

Program: T0058.TEM, Version: 8.2, Datetime: 07FEB07:09:48

Table 14.3.3.8a: Summary of infusion site reactions by preferred term and severity - by diagnosis of PAD
0 - 6 months Post-Subgam
Number of events (number of patients)

Preferred term	Diagnosis of PAD	Median BMI = 23.4	n	Severity: Mild	Severity: Moderate	Severity: Severe
Injection site urticaria	CVID/XLA	>= median	0 (0)	0 (0)	0 (0)	0 (0)
		< median	0 (0)	0 (0)	0 (0)	0 (0)
	Other	>= median	0 (0)	0 (0)	0 (0)	0 (0)
		< median	2 (1)	0 (0)	2 (1)	0 (0)
Total	CVID/XLA	>= median	28 (9)	26 (9)	2 (1)	0 (0)
		< median	22 (5)	18 (5)	4 (2)	0 (0)
	Other	>= median	5 (3)	5 (3)	0 (0)	0 (0)
		< median	4 (3)	2 (2)	2 (1)	0 (0)

The calculation of median BMI includes all patients participating in the study within the time period, whether or not they reported an infusion site reaction. For those patients who had an infusion site reaction within the time period, the mean weighted BMI for each site reaction is calculated for each patient. Otherwise, for those patients who did not have an infusion site reaction within the time period, the BMI for each patient is the mean of most relevant calculated BMIs for that patient.

Program: T0058.TEM, Version: 8.2, Datetime: 07FEB07:09:48

Table 14.3.3.8b: Summary of infusion site reactions by preferred term and severity - by diagnosis of PAD
>6 - 12 months Post-Subgam
Number of events (number of patients)

Preferred term	Diagnosis of PAD	Median BMI = 23.4	n	Severity: Mild	Moderate	Severe
Total number of infusions for period	.		960			
Infusion site inflammation	CVID/XLA	>= median	1 (1)	1 (1)	0 (0)	0 (0)
		< median	0 (0)	0 (0)	0 (0)	0 (0)
	Other	>= median	0 (0)	0 (0)	0 (0)	0 (0)
		< median	0 (0)	0 (0)	0 (0)	0 (0)
Infusion site pain	CVID/XLA	>= median	1 (1)	1 (1)	0 (0)	0 (0)
		< median	1 (1)	1 (1)	0 (0)	0 (0)
	Other	>= median	0 (0)	0 (0)	0 (0)	0 (0)
		< median	0 (0)	0 (0)	0 (0)	0 (0)
Infusion site swelling	CVID/XLA	>= median	2 (2)	2 (2)	0 (0)	0 (0)
		< median	0 (0)	0 (0)	0 (0)	0 (0)
	Other	>= median	0 (0)	0 (0)	0 (0)	0 (0)
		< median	0 (0)	0 (0)	0 (0)	0 (0)
Injection site bruising	CVID/XLA	>= median	2 (1)	2 (1)	0 (0)	0 (0)
		< median	0 (0)	0 (0)	0 (0)	0 (0)
	Other	>= median	0 (0)	0 (0)	0 (0)	0 (0)
		< median	0 (0)	0 (0)	0 (0)	0 (0)

The calculation of median BMI includes all patients participating in the study within the time period, whether or not they reported an infusion site reaction. For those patients who had an infusion site reaction within the time period, the mean weighted BMI for each site reaction is calculated for each patient. Otherwise, for those patients who did not have an infusion site reaction within the time period, the BMI for each patient is the mean of most relevant calculated BMIs for that patient.

Program: T0059.TEM, Version: 8.2, Datetime: 07FEB07:09:48

Table 14.3.3.8b: Summary of infusion site reactions by preferred term and severity - by diagnosis of PAD
>6 - 12 months Post-Subgam
Number of events (number of patients)

Preferred term	Diagnosis of PAD	Median BMI = 23.4	n	Severity		
				Mild	Moderate	Severe
Total	CVID/XLA	>= median	6 (3)	6 (3)	0 (0)	0 (0)
		< median	1 (1)	1 (1)	0 (0)	0 (0)
	Other	>= median	0 (0)	0 (0)	0 (0)	0 (0)
		< median	0 (0)	0 (0)	0 (0)	0 (0)

The calculation of median BMI includes all patients participating in the study within the time period, whether or not they reported an infusion site reaction. For those patients who had an infusion site reaction within the time period, the mean weighted BMI for each site reaction is calculated for each patient. Otherwise, for those patients who did not have an infusion site reaction within the time period, the BMI for each patient is the mean of most relevant calculated BMIs for that patient.

Program: T0059.TEM, Version: 8.2, Datetime: 07FEB07:09:48

Table 14.3.3.8c: Summary of infusion site reactions by preferred term and severity - by diagnosis of PAD
>12 - 18 months Post-Subgam
Number of events (number of patients)

Preferred term	Diagnosis of PAD	Median BMI = 23.0	n	Severity: Mild	Severity: Moderate	Severity: Severe
Total number of infusions for period	.		1,065			
Infusion site erythema	CVID/XLA	>= median	0 (0)	0 (0)	0 (0)	0 (0)
		< median	0 (0)	0 (0)	0 (0)	0 (0)
	Other	>= median	0 (0)	0 (0)	0 (0)	0 (0)
		< median	2 (1)	2 (1)	0 (0)	0 (0)
Infusion site infection	CVID/XLA	>= median	1 (1)	1 (1)	0 (0)	0 (0)
		< median	0 (0)	0 (0)	0 (0)	0 (0)
	Other	>= median	0 (0)	0 (0)	0 (0)	0 (0)
		< median	0 (0)	0 (0)	0 (0)	0 (0)
Infusion site inflammation	CVID/XLA	>= median	2 (2)	2 (2)	0 (0)	0 (0)
		< median	0 (0)	0 (0)	0 (0)	0 (0)
	Other	>= median	0 (0)	0 (0)	0 (0)	0 (0)
		< median	0 (0)	0 (0)	0 (0)	0 (0)
Infusion site pain	CVID/XLA	>= median	1 (1)	1 (1)	0 (0)	0 (0)
		< median	0 (0)	0 (0)	0 (0)	0 (0)
	Other	>= median	0 (0)	0 (0)	0 (0)	0 (0)
		< median	0 (0)	0 (0)	0 (0)	0 (0)

The calculation of median BMI includes all patients participating in the study within the time period, whether or not they reported an infusion site reaction. For those patients who had an infusion site reaction within the time period, the mean weighted BMI for each site reaction is calculated for each patient. Otherwise, for those patients who did not have an infusion site reaction within the time period, the BMI for each patient is the mean of most relevant calculated BMIs for that patient.

Program: T0060.TEM, Version: 8.2, Datetime: 07FEB07:09:48

Table 14.3.3.8c: Summary of infusion site reactions by preferred term and severity - by diagnosis of PAD
>12 - 18 months Post-Subgam
Number of events (number of patients)

				Severity		
Preferred term	Diagnosis of PAD	Median BMI = 23.0	n	Mild	Moderate	Severe
Total	CVID/XLA	>= median	4 (3)	4 (3)	0 (0)	0 (0)
		< median	0 (0)	0 (0)	0 (0)	0 (0)
	Other	>= median	0 (0)	0 (0)	0 (0)	0 (0)
		< median	2 (1)	2 (1)	0 (0)	0 (0)

The calculation of median BMI includes all patients participating in the study within the time period, whether or not they reported an infusion site reaction. For those patients who had an infusion site reaction within the time period, the mean weighted BMI for each site reaction is calculated for each patient. Otherwise, for those patients who did not have an infusion site reaction within the time period, the BMI for each patient is the mean of most relevant calculated BMIs for that patient.

Program: T0060.TEM, Version: 8.2, Datetime: 07FEB07:09:48

Table 14.3.3.8d: Summary of infusion site reactions by preferred term and severity - by diagnosis of PAD
>18 - 24 months Post-Subgam
Number of events (number of patients)

Preferred term	Diagnosis of PAD	Median BMI = 23.0	n	Severity: Mild	Moderate	Severe
Total number of infusions for period	.		1,047			
Infusion site erythema	CVID/XLA	>= median	0 (0)	0 (0)	0 (0)	0 (0)
		< median	0 (0)	0 (0)	0 (0)	0 (0)
	Other	>= median	0 (0)	0 (0)	0 (0)	0 (0)
		< median	1 (1)	1 (1)	0 (0)	0 (0)
Infusion site inflammation	CVID/XLA	>= median	1 (1)	1 (1)	0 (0)	0 (0)
		< median	0 (0)	0 (0)	0 (0)	0 (0)
	Other	>= median	0 (0)	0 (0)	0 (0)	0 (0)
		< median	0 (0)	0 (0)	0 (0)	0 (0)
Total	CVID/XLA	>= median	1 (1)	1 (1)	0 (0)	0 (0)
		< median	0 (0)	0 (0)	0 (0)	0 (0)
	Other	>= median	0 (0)	0 (0)	0 (0)	0 (0)
		< median	1 (1)	1 (1)	0 (0)	0 (0)

The calculation of median BMI includes all patients participating in the study within the time period, whether or not they reported an infusion site reaction. For those patients who had an infusion site reaction within the time period, the mean weighted BMI for each site reaction is calculated for each patient. Otherwise, for those patients who did not have an infusion site reaction within the time period, the BMI for each patient is the mean of most relevant calculated BMIs for that patient.

Program: T0061.TEM, Version: 8.2, Datetime: 07FEB07:09:48

Table 14.3.3.8e: Summary of infusion site reactions by preferred term and severity - by diagnosis of PAD
>24 - 30 months Post-Subgam
Number of events (number of patients)

Preferred term	Diagnosis of PAD	Median BMI = 23.2	n	Severity: Mild	Severity: Moderate	Severity: Severe
Total number of infusions for period	.		1,039			
Infusion site tenderness	CVID/XLA	>= median	1 (1)	0 (0)	1 (1)	0 (0)
		< median	0 (0)	0 (0)	0 (0)	0 (0)
	Other	>= median	0 (0)	0 (0)	0 (0)	0 (0)
		< median	0 (0)	0 (0)	0 (0)	0 (0)
Injection site stinging	CVID/XLA	>= median	1 (1)	1 (1)	0 (0)	0 (0)
		< median	0 (0)	0 (0)	0 (0)	0 (0)
	Other	>= median	0 (0)	0 (0)	0 (0)	0 (0)
		< median	0 (0)	0 (0)	0 (0)	0 (0)
Total	CVID/XLA	>= median	2 (2)	1 (1)	1 (1)	0 (0)
		< median	0 (0)	0 (0)	0 (0)	0 (0)
	Other	>= median	0 (0)	0 (0)	0 (0)	0 (0)
		< median	0 (0)	0 (0)	0 (0)	0 (0)

The calculation of median BMI includes all patients participating in the study within the time period, whether or not they reported an infusion site reaction. For those patients who had an infusion site reaction within the time period, the mean weighted BMI for each site reaction is calculated for each patient. Otherwise, for those patients who did not have an infusion site reaction within the time period, the BMI for each patient is the mean of most relevant calculated BMIs for that patient.

Program: T0062.TEM, Version: 8.2, Datetime: 07FEB07:09:48

Table 14.3.3.8f: Summary of infusion site reactions by preferred term and severity - by diagnosis of PAD
>30 - 36 months Post-Subgam
Number of events (number of patients)

Preferred term	Diagnosis of PAD	Median BMI = 23.8	n	Severity		
				Mild	Moderate	Severe
Total number of infusions for period	.		879			
Infusion site infection	CVID/XLA	>= median	1 (1)	1 (1)	0 (0)	0 (0)
		< median	0 (0)	0 (0)	0 (0)	0 (0)
	Other	>= median	0 (0)	0 (0)	0 (0)	0 (0)
		< median	0 (0)	0 (0)	0 (0)	0 (0)
Infusion site inflammation	CVID/XLA	>= median	1 (1)	1 (1)	0 (0)	0 (0)
		< median	0 (0)	0 (0)	0 (0)	0 (0)
	Other	>= median	0 (0)	0 (0)	0 (0)	0 (0)
		< median	1 (1)	0 (0)	1 (1)	0 (0)
Infusion site pain	CVID/XLA	>= median	1 (1)	1 (1)	0 (0)	0 (0)
		< median	0 (0)	0 (0)	0 (0)	0 (0)
	Other	>= median	0 (0)	0 (0)	0 (0)	0 (0)
		< median	0 (0)	0 (0)	0 (0)	0 (0)
Infusion site pruritus	CVID/XLA	>= median	1 (1)	1 (1)	0 (0)	0 (0)
		< median	0 (0)	0 (0)	0 (0)	0 (0)
	Other	>= median	0 (0)	0 (0)	0 (0)	0 (0)
		< median	0 (0)	0 (0)	0 (0)	0 (0)

The calculation of median BMI includes all patients participating in the study within the time period, whether or not they reported an infusion site reaction. For those patients who had an infusion site reaction within the time period, the mean weighted BMI for each site reaction is calculated for each patient. Otherwise, for those patients who did not have an infusion site reaction within the time period, the BMI for each patient is the mean of most relevant calculated BMIs for that patient.

Program: T0063.TEM, Version: 8.2, Datetime: 07FEB07:09:48

Table 14.3.3.8f: Summary of infusion site reactions by preferred term and severity - by diagnosis of PAD
>30 - 36 months Post-Subgam
Number of events (number of patients)

				Severity		
Preferred term	Diagnosis of PAD	Median BMI = 23.8	n	Mild	Moderate	Severe
Infusion site swelling	CVID/XLA	>= median	0 (0)	0 (0)	0 (0)	0 (0)
		< median	0 (0)	0 (0)	0 (0)	0 (0)
	Other	>= median	0 (0)	0 (0)	0 (0)	0 (0)
		< median	1 (1)	1 (1)	0 (0)	0 (0)
Injection site discomfort	CVID/XLA	>= median	0 (0)	0 (0)	0 (0)	0 (0)
		< median	0 (0)	0 (0)	0 (0)	0 (0)
	Other	>= median	0 (0)	0 (0)	0 (0)	0 (0)
		< median	1 (1)	1 (1)	0 (0)	0 (0)
Injection site stinging	CVID/XLA	>= median	1 (1)	1 (1)	0 (0)	0 (0)
		< median	0 (0)	0 (0)	0 (0)	0 (0)
	Other	>= median	0 (0)	0 (0)	0 (0)	0 (0)
		< median	0 (0)	0 (0)	0 (0)	0 (0)
Total	CVID/XLA	>= median	5 (4)	5 (4)	0 (0)	0 (0)
		< median	0 (0)	0 (0)	0 (0)	0 (0)
	Other	>= median	0 (0)	0 (0)	0 (0)	0 (0)
		< median	3 (1)	2 (1)	1 (1)	0 (0)

The calculation of median BMI includes all patients participating in the study within the time period, whether or not they reported an infusion site reaction. For those patients who had an infusion site reaction within the time period, the mean weighted BMI for each site reaction is calculated for each patient. Otherwise, for those patients who did not have an infusion site reaction within the time period, the BMI for each patient is the mean of most relevant calculated BMIs for that patient.

Program: T0063.TEM, Version: 8.2, Datetime: 07FEB07:09:48

Table 14.3.3.8g: Summary of infusion site reactions by preferred term and severity - by diagnosis of PAD
>36 - 42 months Post-Subgam
Number of events (number of patients)

```
/******************************************************************************************/
/*                                                                                        */
/*                                                                                        */
/*  No observations met the criteria of the current report.                               */
/*                                                                                        */
/*                                                                                        */
/*                                                                                        */
/******************************************************************************************/
```

The calculation of median BMI includes all patients participating in the study within the time period, whether or not they reported an infusion site reaction. For those patients who had an infusion site reaction within the time period, the mean weighted BMI for each site reaction is calculated for each patient. Otherwise, for those patients who did not have an infusion site reaction within the time period, the BMI for each patient is the mean of most relevant calculated BMIs for that patient.

Program: T0064.TEM, Version: 8.2, Datetime: 07FEB07:09:48

Table 14.3.3.8h: Summary of infusion site reactions by preferred term and severity - by diagnosis of PAD
>42 - 48 months Post-Subgam
Number of events (number of patients)

```
/*****************************************************************************************/
/*                                                                                       */
/*                                                                                       */
/*  No observations met the criteria of the current report.                              */
/*                                                                                       */
/*                                                                                       */
/*                                                                                       */
/*****************************************************************************************/
```

The calculation of median BMI includes all patients participating in the study within the time period, whether or not they reported an infusion site reaction. For those patients who had an infusion site reaction within the time period, the mean weighted BMI for each site reaction is calculated for each patient. Otherwise, for those patients who did not have an infusion site reaction within the time period, the BMI for each patient is the mean of most relevant calculated BMIs for that patient.

Table 14.3.3.8i: Summary of infusion site reactions by preferred term and severity - by diagnosis of PAD
>48 - 54 months Post-Subgam
Number of events (number of patients)

```
/*********************************************************************************/
/*                                                                               */
/*                                                                               */
/*  No observations met the criteria of the current report.                      */
/*                                                                               */
/*                                                                               */
/*                                                                               */
/*********************************************************************************/
```

The calculation of median BMI includes all patients participating in the study within the time period, whether or not they reported an infusion site reaction. For those patients who had an infusion site reaction within the time period, the mean weighted BMI for each site reaction is calculated for each patient. Otherwise, for those patients who did not have an infusion site reaction within the time period, the BMI for each patient is the mean of most relevant calculated BMIs for that patient.

Table 14.3.3.9a: Summary of infusion site reactions by preferred term and severity - by age group
0 - 6 months Post-Subgam
Number of events (number of patients)

Preferred term	Age Group	Median BMI = 26.7 for adults 19.7 for teenagers 16.5 for children	n	Severity		
				Mild	Moderate	Severe
Total number of infusions for period	.		1,391			
Infusion site burning	Adult	>= median	0 (0)	0 (0)	0 (0)	0 (0)
		< median	0 (0)	0 (0)	0 (0)	0 (0)
	Child	>= median	0 (0)	0 (0)	0 (0)	0 (0)
		< median	1 (1)	1 (1)	0 (0)	0 (0)
	Teenager	>= median	0 (0)	0 (0)	0 (0)	0 (0)
		< median	0 (0)	0 (0)	0 (0)	0 (0)
Infusion site erythema	Adult	>= median	1 (1)	1 (1)	0 (0)	0 (0)
		< median	4 (2)	3 (2)	1 (1)	0 (0)
	Child	>= median	0 (0)	0 (0)	0 (0)	0 (0)
		< median	0 (0)	0 (0)	0 (0)	0 (0)
	Teenager	>= median	4 (1)	4 (1)	0 (0)	0 (0)
		< median	0 (0)	0 (0)	0 (0)	0 (0)
Infusion site induration	Adult	>= median	0 (0)	0 (0)	0 (0)	0 (0)
		< median	0 (0)	0 (0)	0 (0)	0 (0)
	Child	>= median	0 (0)	0 (0)	0 (0)	0 (0)
		< median	0 (0)	0 (0)	0 (0)	0 (0)
	Teenager	>= median	2 (1)	2 (1)	0 (0)	0 (0)
		< median	0 (0)	0 (0)	0 (0)	0 (0)

The calculation of median BMI includes all patients participating in the study within the time period, whether or not they reported an infusion site reaction. For those patients who had an infusion site reaction within the time period, the mean weighted BMI for each site reaction is calculated for each patient. Otherwise, for those patients who did not have an infusion site reaction within the time period, the BMI for each patient is the mean of most relevant calculated BMIs for that patient.

Table 14.3.3.9a: Summary of infusion site reactions by preferred term and severity - by age group
0 - 6 months Post-Subgam
Number of events (number of patients)

Preferred term	Age Group	Median BMI = 26.7 for adults 19.7 for teenagers 16.5 for children	n	Severity		
				Mild	Moderate	Severe
Infusion site inflammation	Adult	>= median	1 (1)	1 (1)	0 (0)	0 (0)
		< median	9 (5)	9 (5)	0 (0)	0 (0)
	Child	>= median	0 (0)	0 (0)	0 (0)	0 (0)
		< median	0 (0)	0 (0)	0 (0)	0 (0)
	Teenager	>= median	0 (0)	0 (0)	0 (0)	0 (0)
		< median	0 (0)	0 (0)	0 (0)	0 (0)
Infusion site pain	Adult	>= median	0 (0)	0 (0)	0 (0)	0 (0)
		< median	0 (0)	0 (0)	0 (0)	0 (0)
	Child	>= median	1 (1)	1 (1)	0 (0)	0 (0)
		< median	0 (0)	0 (0)	0 (0)	0 (0)
	Teenager	>= median	0 (0)	0 (0)	0 (0)	0 (0)
		< median	0 (0)	0 (0)	0 (0)	0 (0)
Infusion site pruritus	Adult	>= median	2 (1)	2 (1)	0 (0)	0 (0)
		< median	1 (1)	1 (1)	0 (0)	0 (0)
	Child	>= median	0 (0)	0 (0)	0 (0)	0 (0)
		< median	0 (0)	0 (0)	0 (0)	0 (0)
	Teenager	>= median	0 (0)	0 (0)	0 (0)	0 (0)
		< median	0 (0)	0 (0)	0 (0)	0 (0)

The calculation of median BMI includes all patients participating in the study within the time period, whether or not they reported an infusion site reaction. For those patients who had an infusion site reaction within the time period, the mean weighted BMI for each site reaction is calculated for each patient. Otherwise, for those patients who did not have an infusion site reaction within the time period, the BMI for each patient is the mean of most relevant calculated BMIs for that patient.

Table 14.3.3.9a: Summary of infusion site reactions by preferred term and severity - by age group
0 - 6 months Post-Subgam
Number of events (number of patients)

Preferred term	Age Group	Median BMI = 26.7 for adults 19.7 for teenagers 16.5 for children	n	Severity: Mild	Severity: Moderate	Severity: Severe
Infusion site swelling	Adult	>= median	4 (2)	3 (2)	1 (1)	0 (0)
		< median	2 (1)	1 (1)	1 (1)	0 (0)
	Child	>= median	0 (0)	0 (0)	0 (0)	0 (0)
		< median	1 (1)	1 (1)	0 (0)	0 (0)
	Teenager	>= median	4 (1)	3 (1)	1 (1)	0 (0)
		< median	0 (0)	0 (0)	0 (0)	0 (0)
Infusion site tenderness	Adult	>= median	1 (1)	0 (0)	1 (1)	0 (0)
		< median	1 (1)	1 (1)	0 (0)	0 (0)
	Child	>= median	0 (0)	0 (0)	0 (0)	0 (0)
		< median	0 (0)	0 (0)	0 (0)	0 (0)
	Teenager	>= median	0 (0)	0 (0)	0 (0)	0 (0)
		< median	0 (0)	0 (0)	0 (0)	0 (0)
Injection site bruising	Adult	>= median	1 (1)	1 (1)	0 (0)	0 (0)
		< median	2 (1)	2 (1)	0 (0)	0 (0)
	Child	>= median	0 (0)	0 (0)	0 (0)	0 (0)
		< median	0 (0)	0 (0)	0 (0)	0 (0)
	Teenager	>= median	0 (0)	0 (0)	0 (0)	0 (0)
		< median	0 (0)	0 (0)	0 (0)	0 (0)

The calculation of median BMI includes all patients participating in the study within the time period, whether or not they reported an infusion site reaction. For those patients who had an infusion site reaction within the time period, the mean weighted BMI for each site reaction is calculated for each patient. Otherwise, for those patients who did not have an infusion site reaction within the time period, the BMI for each patient is the mean of most relevant calculated BMIs for that patient.

Table 14.3.3.9a: Summary of infusion site reactions by preferred term and severity - by age group
0 - 6 months Post-Subgam
Number of events (number of patients)

Preferred term	Age Group	Median BMI = 26.7 for adults 19.7 for teenagers 16.5 for children	n	Severity		
				Mild	Moderate	Severe
Injection site haemorrhage	Adult	>= median	0 (0)	0 (0)	0 (0)	0 (0)
		< median	1 (1)	0 (0)	1 (1)	0 (0)
	Child	>= median	0 (0)	0 (0)	0 (0)	0 (0)
		< median	0 (0)	0 (0)	0 (0)	0 (0)
	Teenager	>= median	0 (0)	0 (0)	0 (0)	0 (0)
		< median	0 (0)	0 (0)	0 (0)	0 (0)
Injection site paraesthesia	Adult	>= median	1 (1)	1 (1)	0 (0)	0 (0)
		< median	0 (0)	0 (0)	0 (0)	0 (0)
	Child	>= median	0 (0)	0 (0)	0 (0)	0 (0)
		< median	0 (0)	0 (0)	0 (0)	0 (0)
	Teenager	>= median	1 (1)	1 (1)	0 (0)	0 (0)
		< median	0 (0)	0 (0)	0 (0)	0 (0)
Injection site rash	Adult	>= median	7 (1)	7 (1)	0 (0)	0 (0)
		< median	4 (3)	4 (3)	0 (0)	0 (0)
	Child	>= median	0 (0)	0 (0)	0 (0)	0 (0)
		< median	1 (1)	1 (1)	0 (0)	0 (0)
	Teenager	>= median	0 (0)	0 (0)	0 (0)	0 (0)
		< median	0 (0)	0 (0)	0 (0)	0 (0)

The calculation of median BMI includes all patients participating in the study within the time period, whether or not they reported an infusion site reaction. For those patients who had an infusion site reaction within the time period, the mean weighted BMI for each site reaction is calculated for each patient. Otherwise, for those patients who did not have an infusion site reaction within the time period, the BMI for each patient is the mean of most relevant calculated BMIs for that patient.

Program: T0067.TEM, Version: 8.2, Datetime: 07FEB07:09:48

Table 14.3.3.9a: Summary of infusion site reactions by preferred term and severity - by age group
0 - 6 months Post-Subgam
Number of events (number of patients)

Preferred term	Age Group	Median BMI = 26.7 for adults 19.7 for teenagers 16.5 for children	n	Severity		
				Mild	Moderate	Severe
Injection site urticaria	Adult	>= median	0 (0)	0 (0)	0 (0)	0 (0)
		< median	2 (1)	0 (0)	2 (1)	0 (0)
	Child	>= median	0 (0)	0 (0)	0 (0)	0 (0)
		< median	0 (0)	0 (0)	0 (0)	0 (0)
	Teenager	>= median	0 (0)	0 (0)	0 (0)	0 (0)
		< median	0 (0)	0 (0)	0 (0)	0 (0)
Total	Adult	>= median	18 (5)	16 (5)	2 (1)	0 (0)
		< median	26 (10)	21 (9)	5 (2)	0 (0)
	Child	>= median	1 (1)	1 (1)	0 (0)	0 (0)
		< median	3 (3)	3 (3)	0 (0)	0 (0)
	Teenager	>= median	11 (2)	10 (2)	1 (1)	0 (0)
		< median	0 (0)	0 (0)	0 (0)	0 (0)

The calculation of median BMI includes all patients participating in the study within the time period, whether or not they reported an infusion site reaction. For those patients who had an infusion site reaction within the time period, the mean weighted BMI for each site reaction is calculated for each patient. Otherwise, for those patients who did not have an infusion site reaction within the time period, the BMI for each patient is the mean of most relevant calculated BMIs for that patient.

Table 14.3.3.9b: Summary of infusion site reactions by preferred term and severity - by age group
>6 - 12 months Post-Subgam
Number of events (number of patients)

Preferred term	Age Group	Median BMI = 27.1 for adults 20.2 for teenagers 16.6 for children	n	Severity: Mild	Severity: Moderate	Severity: Severe
Total number of infusions for period	.		960			
Infusion site inflammation	Adult	>= median	0 (0)	0 (0)	0 (0)	0 (0)
		< median	1 (1)	1 (1)	0 (0)	0 (0)
	Child	>= median	0 (0)	0 (0)	0 (0)	0 (0)
		< median	0 (0)	0 (0)	0 (0)	0 (0)
	Teenager	>= median	0 (0)	0 (0)	0 (0)	0 (0)
		< median	0 (0)	0 (0)	0 (0)	0 (0)
Infusion site pain	Adult	>= median	0 (0)	0 (0)	0 (0)	0 (0)
		< median	2 (2)	2 (2)	0 (0)	0 (0)
	Child	>= median	0 (0)	0 (0)	0 (0)	0 (0)
		< median	0 (0)	0 (0)	0 (0)	0 (0)
	Teenager	>= median	0 (0)	0 (0)	0 (0)	0 (0)
		< median	0 (0)	0 (0)	0 (0)	0 (0)
Infusion site swelling	Adult	>= median	1 (1)	1 (1)	0 (0)	0 (0)
		< median	0 (0)	0 (0)	0 (0)	0 (0)
	Child	>= median	0 (0)	0 (0)	0 (0)	0 (0)
		< median	0 (0)	0 (0)	0 (0)	0 (0)
	Teenager	>= median	1 (1)	1 (1)	0 (0)	0 (0)
		< median	0 (0)	0 (0)	0 (0)	0 (0)

The calculation of median BMI includes all patients participating in the study within the time period, whether or not they reported an infusion site reaction. For those patients who had an infusion site reaction within the time period, the mean weighted BMI for each site reaction is calculated for each patient. Otherwise, for those patients who did not have an infusion site reaction within the time period, the BMI for each patient is the mean of most relevant calculated BMIs for that patient.

Program: T0068.TEM, Version: 8.2, Datetime: 07FEB07:09:48

Table 14.3.3.9b: Summary of infusion site reactions by preferred term and severity - by age group
>6 - 12 months Post-Subgam
Number of events (number of patients)

Preferred term	Age Group	Median BMI = 27.1 for adults 20.2 for teenagers 16.6 for children	n	Severity: Mild	Severity: Moderate	Severity: Severe
Injection site bruising	Adult	>= median	0 (0)	0 (0)	0 (0)	0 (0)
		< median	2 (1)	2 (1)	0 (0)	0 (0)
	Child	>= median	0 (0)	0 (0)	0 (0)	0 (0)
		< median	0 (0)	0 (0)	0 (0)	0 (0)
	Teenager	>= median	0 (0)	0 (0)	0 (0)	0 (0)
		< median	0 (0)	0 (0)	0 (0)	0 (0)
Total	Adult	>= median	1 (1)	1 (1)	0 (0)	0 (0)
		< median	5 (2)	5 (2)	0 (0)	0 (0)
	Child	>= median	0 (0)	0 (0)	0 (0)	0 (0)
		< median	0 (0)	0 (0)	0 (0)	0 (0)
	Teenager	>= median	1 (1)	1 (1)	0 (0)	0 (0)
		< median	0 (0)	0 (0)	0 (0)	0 (0)

The calculation of median BMI includes all patients participating in the study within the time period, whether or not they reported an infusion site reaction. For those patients who had an infusion site reaction within the time period, the mean weighted BMI for each site reaction is calculated for each patient. Otherwise, for those patients who did not have an infusion site reaction within the time period, the BMI for each patient is the mean of most relevant calculated BMIs for that patient.

Table 14.3.3.9c: Summary of infusion site reactions by preferred term and severity - by age group
>12 - 18 months Post-Subgam
Number of events (number of patients)

Preferred term	Age Group	Median BMI = 27.1 for adults 21.2 for teenagers 16.6 for children	n	Severity: Mild	Severity: Moderate	Severity: Severe
Total number of infusions for period	.		1,065			
Infusion site erythema	Adult	>= median	0 (0)	0 (0)	0 (0)	0 (0)
		< median	0 (0)	0 (0)	0 (0)	0 (0)
	Child	>= median	0 (0)	0 (0)	0 (0)	0 (0)
		< median	2 (1)	2 (1)	0 (0)	0 (0)
	Teenager	>= median	0 (0)	0 (0)	0 (0)	0 (0)
		< median	0 (0)	0 (0)	0 (0)	0 (0)
Infusion site infection	Adult	>= median	0 (0)	0 (0)	0 (0)	0 (0)
		< median	1 (1)	1 (1)	0 (0)	0 (0)
	Child	>= median	0 (0)	0 (0)	0 (0)	0 (0)
		< median	0 (0)	0 (0)	0 (0)	0 (0)
	Teenager	>= median	0 (0)	0 (0)	0 (0)	0 (0)
		< median	0 (0)	0 (0)	0 (0)	0 (0)
Infusion site inflammation	Adult	>= median	1 (1)	1 (1)	0 (0)	0 (0)
		< median	1 (1)	1 (1)	0 (0)	0 (0)
	Child	>= median	0 (0)	0 (0)	0 (0)	0 (0)
		< median	0 (0)	0 (0)	0 (0)	0 (0)
	Teenager	>= median	0 (0)	0 (0)	0 (0)	0 (0)
		< median	0 (0)	0 (0)	0 (0)	0 (0)

The calculation of median BMI includes all patients participating in the study within the time period, whether or not they reported an infusion site reaction. For those patients who had an infusion site reaction within the time period, the mean weighted BMI for each site reaction is calculated for each patient. Otherwise, for those patients who did not have an infusion site reaction within the time period, the BMI for each patient is the mean of most relevant calculated BMIs for that patient.

Table 14.3.3.9c: Summary of infusion site reactions by preferred term and severity - by age group
>12 - 18 months Post-Subgam
Number of events (number of patients)

Preferred term	Age Group	Median BMI = 27.1 for adults 21.2 for teenagers 16.6 for children	n	Severity		
				Mild	Moderate	Severe
Infusion site pain	Adult	>= median	1 (1)	1 (1)	0 (0)	0 (0)
		< median	0 (0)	0 (0)	0 (0)	0 (0)
	Child	>= median	0 (0)	0 (0)	0 (0)	0 (0)
		< median	0 (0)	0 (0)	0 (0)	0 (0)
	Teenager	>= median	0 (0)	0 (0)	0 (0)	0 (0)
		< median	0 (0)	0 (0)	0 (0)	0 (0)
Total	Adult	>= median	2 (2)	2 (2)	0 (0)	0 (0)
		< median	2 (1)	2 (1)	0 (0)	0 (0)
	Child	>= median	0 (0)	0 (0)	0 (0)	0 (0)
		< median	2 (1)	2 (1)	0 (0)	0 (0)
	Teenager	>= median	0 (0)	0 (0)	0 (0)	0 (0)
		< median	0 (0)	0 (0)	0 (0)	0 (0)

The calculation of median BMI includes all patients participating in the study within the time period, whether or not they reported an infusion site reaction. For those patients who had an infusion site reaction within the time period, the mean weighted BMI for each site reaction is calculated for each patient. Otherwise, for those patients who did not have an infusion site reaction within the time period, the BMI for each patient is the mean of most relevant calculated BMIs for that patient.

Table 14.3.3.9d: Summary of infusion site reactions by preferred term and severity - by age group
>18 - 24 months Post-Subgam
Number of events (number of patients)

Preferred term	Age Group	Median BMI = 27.1 for adults 21.2 for teenagers 16.6 for children	n	Severity		
				Mild	Moderate	Severe
Total number of infusions for period			1,047			
Infusion site erythema	Adult	>= median	0 (0)	0 (0)	0 (0)	0 (0)
		< median	0 (0)	0 (0)	0 (0)	0 (0)
	Child	>= median	1 (1)	1 (1)	0 (0)	0 (0)
		< median	0 (0)	0 (0)	0 (0)	0 (0)
	Teenager	>= median	0 (0)	0 (0)	0 (0)	0 (0)
		< median	0 (0)	0 (0)	0 (0)	0 (0)
Infusion site inflammation	Adult	>= median	0 (0)	0 (0)	0 (0)	0 (0)
		< median	0 (0)	0 (0)	0 (0)	0 (0)
	Child	>= median	0 (0)	0 (0)	0 (0)	0 (0)
		< median	0 (0)	0 (0)	0 (0)	0 (0)
	Teenager	>= median	1 (1)	1 (1)	0 (0)	0 (0)
		< median	0 (0)	0 (0)	0 (0)	0 (0)
Total	Adult	>= median	0 (0)	0 (0)	0 (0)	0 (0)
		< median	0 (0)	0 (0)	0 (0)	0 (0)
	Child	>= median	1 (1)	1 (1)	0 (0)	0 (0)
		< median	0 (0)	0 (0)	0 (0)	0 (0)
	Teenager	>= median	1 (1)	1 (1)	0 (0)	0 (0)
		< median	0 (0)	0 (0)	0 (0)	0 (0)

The calculation of median BMI includes all patients participating in the study within the time period, whether or not they reported an infusion site reaction. For those patients who had an infusion site reaction within the time period, the mean weighted BMI for each site reaction is calculated for each patient. Otherwise, for those patients who did not have an infusion site reaction within the time period, the BMI for each patient is the mean of most relevant calculated BMIs for that patient.

Table 14.3.3.9e: Summary of infusion site reactions by preferred term and severity - by age group
>24 - 30 months Post-Subgam
Number of events (number of patients)

Preferred term	Age Group	Median BMI = 27.1 for adults 21.2 for teenagers 17.0 for children	n	Severity		
				Mild	Moderate	Severe
Total number of infusions for period			1,039			
Infusion site tenderness	Adult	>= median	1 (1)	0 (0)	1 (1)	0 (0)
		< median	0 (0)	0 (0)	0 (0)	0 (0)
	Child	>= median	0 (0)	0 (0)	0 (0)	0 (0)
		< median	0 (0)	0 (0)	0 (0)	0 (0)
	Teenager	>= median	0 (0)	0 (0)	0 (0)	0 (0)
		< median	0 (0)	0 (0)	0 (0)	0 (0)
Injection site stinging	Adult	>= median	1 (1)	1 (1)	0 (0)	0 (0)
		< median	0 (0)	0 (0)	0 (0)	0 (0)
	Child	>= median	0 (0)	0 (0)	0 (0)	0 (0)
		< median	0 (0)	0 (0)	0 (0)	0 (0)
	Teenager	>= median	0 (0)	0 (0)	0 (0)	0 (0)
		< median	0 (0)	0 (0)	0 (0)	0 (0)
Total	Adult	>= median	2 (2)	1 (1)	1 (1)	0 (0)
		< median	0 (0)	0 (0)	0 (0)	0 (0)
	Child	>= median	0 (0)	0 (0)	0 (0)	0 (0)
		< median	0 (0)	0 (0)	0 (0)	0 (0)
	Teenager	>= median	0 (0)	0 (0)	0 (0)	0 (0)
		< median	0 (0)	0 (0)	0 (0)	0 (0)

The calculation of median BMI includes all patients participating in the study within the time period, whether or not they reported an infusion site reaction. For those patients who had an infusion site reaction within the time period, the mean weighted BMI for each site reaction is calculated for each patient. Otherwise, for those patients who did not have an infusion site reaction within the time period, the BMI for each patient is the mean of most relevant calculated BMIs for that patient.

Program: T0071.TEM, Version: 8.2, Datetime: 07FEB07:09:48

Table 14.3.3.9f: Summary of infusion site reactions by preferred term and severity - by age group
>30 - 36 months Post-Subgam
Number of events (number of patients)

Preferred term	Age Group	Median BMI = 27.1 for adults 21.2 for teenagers 16.3 for children	n	Severity		
				Mild	Moderate	Severe
Total number of infusions for period	.		879			
Infusion site infection	Adult	>= median	0 (0)	0 (0)	0 (0)	0 (0)
		< median	1 (1)	1 (1)	0 (0)	0 (0)
	Child	>= median	0 (0)	0 (0)	0 (0)	0 (0)
		< median	0 (0)	0 (0)	0 (0)	0 (0)
	Teenager	>= median	0 (0)	0 (0)	0 (0)	0 (0)
		< median	0 (0)	0 (0)	0 (0)	0 (0)
Infusion site inflammation	Adult	>= median	1 (1)	1 (1)	0 (0)	0 (0)
		< median	0 (0)	0 (0)	0 (0)	0 (0)
	Child	>= median	1 (1)	0 (0)	1 (1)	0 (0)
		< median	0 (0)	0 (0)	0 (0)	0 (0)
	Teenager	>= median	0 (0)	0 (0)	0 (0)	0 (0)
		< median	0 (0)	0 (0)	0 (0)	0 (0)
Infusion site pain	Adult	>= median	1 (1)	1 (1)	0 (0)	0 (0)
		< median	0 (0)	0 (0)	0 (0)	0 (0)
	Child	>= median	0 (0)	0 (0)	0 (0)	0 (0)
		< median	0 (0)	0 (0)	0 (0)	0 (0)
	Teenager	>= median	0 (0)	0 (0)	0 (0)	0 (0)
		< median	0 (0)	0 (0)	0 (0)	0 (0)

The calculation of median BMI includes all patients participating in the study within the time period, whether or not they reported an infusion site reaction. For those patients who had an infusion site reaction within the time period, the mean weighted BMI for each site reaction is calculated for each patient. Otherwise, for those patients who did not have an infusion site reaction within the time period, the BMI for each patient is the mean of most relevant calculated BMIs for that patient.

Table 14.3.3.9f: Summary of infusion site reactions by preferred term and severity - by age group
>30 - 36 months Post-Subgam
Number of events (number of patients)

Preferred term	Age Group	Median BMI = 27.1 for adults 21.2 for teenagers 16.3 for children	n	Severity Mild	Moderate	Severe
Infusion site pruritus	Adult	>= median	1 (1)	1 (1)	0 (0)	0 (0)
		< median	0 (0)	0 (0)	0 (0)	0 (0)
	Child	>= median	0 (0)	0 (0)	0 (0)	0 (0)
		< median	0 (0)	0 (0)	0 (0)	0 (0)
	Teenager	>= median	0 (0)	0 (0)	0 (0)	0 (0)
		< median	0 (0)	0 (0)	0 (0)	0 (0)
Infusion site swelling	Adult	>= median	0 (0)	0 (0)	0 (0)	0 (0)
		< median	0 (0)	0 (0)	0 (0)	0 (0)
	Child	>= median	1 (1)	1 (1)	0 (0)	0 (0)
		< median	0 (0)	0 (0)	0 (0)	0 (0)
	Teenager	>= median	0 (0)	0 (0)	0 (0)	0 (0)
		< median	0 (0)	0 (0)	0 (0)	0 (0)
Injection site discomfort	Adult	>= median	0 (0)	0 (0)	0 (0)	0 (0)
		< median	0 (0)	0 (0)	0 (0)	0 (0)
	Child	>= median	1 (1)	1 (1)	0 (0)	0 (0)
		< median	0 (0)	0 (0)	0 (0)	0 (0)
	Teenager	>= median	0 (0)	0 (0)	0 (0)	0 (0)
		< median	0 (0)	0 (0)	0 (0)	0 (0)

The calculation of median BMI includes all patients participating in the study within the time period, whether or not they reported an infusion site reaction. For those patients who had an infusion site reaction within the time period, the mean weighted BMI for each site reaction is calculated for each patient. Otherwise, for those patients who did not have an infusion site reaction within the time period, the BMI for each patient is the mean of most relevant calculated BMIs for that patient.

Program: T0072.TEM, Version: 8.2, Datetime: 07FEB07:09:48

Table 14.3.3.9f: Summary of infusion site reactions by preferred term and severity - by age group
>30 - 36 months Post-Subgam
Number of events (number of patients)

Preferred term	Age Group	Median BMI = 27.1 for adults 21.2 for teenagers 16.3 for children	n	Severity		
				Mild	Moderate	Severe
Injection site stinging	Adult	>= median	1 (1)	1 (1)	0 (0)	0 (0)
		< median	0 (0)	0 (0)	0 (0)	0 (0)
	Child	>= median	0 (0)	0 (0)	0 (0)	0 (0)
		< median	0 (0)	0 (0)	0 (0)	0 (0)
	Teenager	>= median	0 (0)	0 (0)	0 (0)	0 (0)
		< median	0 (0)	0 (0)	0 (0)	0 (0)
Total	Adult	>= median	4 (3)	4 (3)	0 (0)	0 (0)
		< median	1 (1)	1 (1)	0 (0)	0 (0)
	Child	>= median	3 (1)	2 (1)	1 (1)	0 (0)
		< median	0 (0)	0 (0)	0 (0)	0 (0)
	Teenager	>= median	0 (0)	0 (0)	0 (0)	0 (0)
		< median	0 (0)	0 (0)	0 (0)	0 (0)

The calculation of median BMI includes all patients participating in the study within the time period, whether or not they reported an infusion site reaction. For those patients who had an infusion site reaction within the time period, the mean weighted BMI for each site reaction is calculated for each patient. Otherwise, for those patients who did not have an infusion site reaction within the time period, the BMI for each patient is the mean of most relevant calculated BMIs for that patient.

Program: T0072.TEM, Version: 8.2, Datetime: 07FEB07:09:48

Table 14.3.3.9g: Summary of infusion site reactions by preferred term and severity - by age group
>36 - 42 months Post-Subgam
Number of events (number of patients)

```
/*****************************************************************************************/
/*                                                                                       */
/*                                                                                       */
/*  No observations met the criteria of the current report.                              */
/*                                                                                       */
/*                                                                                       */
/*                                                                                       */
/*****************************************************************************************/
```

The calculation of median BMI includes all patients participating in the study within the time period, whether or not they reported an infusion site reaction. For those patients who had an infusion site reaction within the time period, the mean weighted BMI for each site reaction is calculated for each patient. Otherwise, for those patients who did not have an infusion site reaction within the time period, the BMI for each patient is the mean of most relevant calculated BMIs for that patient.

Program: T0073.TEM, Version: 8.2, Datetime: 07FEB07:09:48

Table 14.3.3.9h: Summary of infusion site reactions by preferred term and severity - by age group
>42 - 48 months Post-Subgam
Number of events (number of patients)

```
/**************************************************************************************/
/*                                                                                   */
/*                                                                                   */
/*   No observations met the criteria of the current report.                         */
/*                                                                                   */
/*                                                                                   */
/*                                                                                   */
/**************************************************************************************/
```

The calculation of median BMI includes all patients participating in the study within the time period, whether or not they reported an infusion site reaction. For those patients who had an infusion site reaction within the time period, the mean weighted BMI for each site reaction is calculated for each patient. Otherwise, for those patients who did not have an infusion site reaction within the time period, the BMI for each patient is the mean of most relevant calculated BMIs for that patient.

Program: T0074.TEM, Version: 8.2, Datetime: 07FEB07:09:48

Table 14.3.3.9i: Summary of infusion site reactions by preferred term and severity - by age group
>48 - 54 months Post-Subgam
Number of events (number of patients)

```
/*****************************************************************************************/
/*                                                                                       */
/*                                                                                       */
/*  No observations met the criteria of the current report.                              */
/*                                                                                       */
/*                                                                                       */
/*                                                                                       */
/*****************************************************************************************/
```

The calculation of median BMI includes all patients participating in the study within the time period, whether or not they reported an infusion site reaction. For those patients who had an infusion site reaction within the time period, the mean weighted BMI for each site reaction is calculated for each patient. Otherwise, for those patients who did not have an infusion site reaction within the time period, the BMI for each patient is the mean of most relevant calculated BMIs for that patient.

Program: T0075.TEM, Version: 8.2, Datetime: 07FEB07:09:48

Table 14.3.3.10a: Summary of infusion site reactions by preferred term and severity - by prior therapy
0 - 6 months Post-Subgam
Number of events (number of patients)

Preferred term	Prior Therapy	Median BMI = 23.4	n	Severity: Mild	Severity: Moderate	Severity: Severe
Total number of infusions for period	.		1,391			
Infusion site burning	IVIG	>= median	0 (0)	0 (0)	0 (0)	0 (0)
		< median	0 (0)	0 (0)	0 (0)	0 (0)
	SCIG	>= median	0 (0)	0 (0)	0 (0)	0 (0)
		< median	1 (1)	1 (1)	0 (0)	0 (0)
Infusion site erythema	IVIG	>= median	2 (2)	2 (2)	0 (0)	0 (0)
		< median	7 (2)	6 (2)	1 (1)	0 (0)
	SCIG	>= median	0 (0)	0 (0)	0 (0)	0 (0)
		< median	0 (0)	0 (0)	0 (0)	0 (0)
Infusion site induration	IVIG	>= median	2 (1)	2 (1)	0 (0)	0 (0)
		< median	0 (0)	0 (0)	0 (0)	0 (0)
	SCIG	>= median	0 (0)	0 (0)	0 (0)	0 (0)
		< median	0 (0)	0 (0)	0 (0)	0 (0)
Infusion site inflammation	IVIG	>= median	5 (5)	5 (5)	0 (0)	0 (0)
		< median	5 (1)	5 (1)	0 (0)	0 (0)
	SCIG	>= median	0 (0)	0 (0)	0 (0)	0 (0)
		< median	0 (0)	0 (0)	0 (0)	0 (0)

The calculation of median BMI includes all patients participating in the study within the time period, whether or not they reported an infusion site reaction. For those patients who had an infusion site reaction within the time period, the mean weighted BMI for each site reaction is calculated for each patient. Otherwise, for those patients who did not have an infusion site reaction within the time period, the BMI for each patient is the mean of most relevant calculated BMIs for that patient.

Program: T0076.TEM, Version: 8.2, Datetime: 07FEB07:09:48

Table 14.3.3.10a: Summary of infusion site reactions by preferred term and severity - by prior therapy
0 - 6 months Post-Subgam
Number of events (number of patients)

Preferred term	Prior Therapy	Median BMI = 23.4	n	Severity: Mild	Severity: Moderate	Severity: Severe
Infusion site pain	IVIG	>= median	0 (0)	0 (0)	0 (0)	0 (0)
		< median	1 (1)	1 (1)	0 (0)	0 (0)
	SCIG	>= median	0 (0)	0 (0)	0 (0)	0 (0)
		< median	0 (0)	0 (0)	0 (0)	0 (0)
Infusion site pruritus	IVIG	>= median	2 (1)	2 (1)	0 (0)	0 (0)
		< median	0 (0)	0 (0)	0 (0)	0 (0)
	SCIG	>= median	1 (1)	1 (1)	0 (0)	0 (0)
		< median	0 (0)	0 (0)	0 (0)	0 (0)
Infusion site swelling	IVIG	>= median	4 (2)	3 (2)	1 (1)	0 (0)
		< median	6 (2)	4 (2)	2 (2)	0 (0)
	SCIG	>= median	0 (0)	0 (0)	0 (0)	0 (0)
		< median	1 (1)	1 (1)	0 (0)	0 (0)
Infusion site tenderness	IVIG	>= median	2 (2)	1 (1)	1 (1)	0 (0)
		< median	0 (0)	0 (0)	0 (0)	0 (0)
	SCIG	>= median	0 (0)	0 (0)	0 (0)	0 (0)
		< median	0 (0)	0 (0)	0 (0)	0 (0)

The calculation of median BMI includes all patients participating in the study within the time period, whether or not they reported an infusion site reaction. For those patients who had an infusion site reaction within the time period, the mean weighted BMI for each site reaction is calculated for each patient. Otherwise, for those patients who did not have an infusion site reaction within the time period, the BMI for each patient is the mean of most relevant calculated BMIs for that patient.

Program: T0076.TEM, Version: 8.2, Datetime: 07FEB07:09:48

Table 14.3.3.10a: Summary of infusion site reactions by preferred term and severity - by prior therapy
0 - 6 months Post-Subgam
Number of events (number of patients)

Preferred term	Prior Therapy	Median BMI = 23.4	n	Severity: Mild	Severity: Moderate	Severity: Severe
Injection site bruising	IVIG	>= median	3 (2)	3 (2)	0 (0)	0 (0)
		< median	0 (0)	0 (0)	0 (0)	0 (0)
	SCIG	>= median	0 (0)	0 (0)	0 (0)	0 (0)
		< median	0 (0)	0 (0)	0 (0)	0 (0)
Injection site haemorrhage	IVIG	>= median	0 (0)	0 (0)	0 (0)	0 (0)
		< median	1 (1)	0 (0)	1 (1)	0 (0)
	SCIG	>= median	0 (0)	0 (0)	0 (0)	0 (0)
		< median	0 (0)	0 (0)	0 (0)	0 (0)
Injection site paraesthesia	IVIG	>= median	1 (1)	1 (1)	0 (0)	0 (0)
		< median	1 (1)	1 (1)	0 (0)	0 (0)
	SCIG	>= median	0 (0)	0 (0)	0 (0)	0 (0)
		< median	0 (0)	0 (0)	0 (0)	0 (0)
Injection site rash	IVIG	>= median	1 (1)	1 (1)	0 (0)	0 (0)
		< median	0 (0)	0 (0)	0 (0)	0 (0)
	SCIG	>= median	10 (2)	10 (2)	0 (0)	0 (0)
		< median	1 (1)	1 (1)	0 (0)	0 (0)

The calculation of median BMI includes all patients participating in the study within the time period, whether or not they reported an infusion site reaction. For those patients who had an infusion site reaction within the time period, the mean weighted BMI for each site reaction is calculated for each patient. Otherwise, for those patients who did not have an infusion site reaction within the time period, the BMI for each patient is the mean of most relevant calculated BMIs for that patient.

Program: T0076.TEM, Version: 8.2, Datetime: 07FEB07:09:48

Table 14.3.3.10a: Summary of infusion site reactions by preferred term and severity - by prior therapy
0 - 6 months Post-Subgam
Number of events (number of patients)

Preferred term	Prior Therapy	Median BMI = 23.4	n	Severity: Mild	Severity: Moderate	Severity: Severe
Injection site urticaria	IVIG	>= median	0 (0)	0 (0)	0 (0)	0 (0)
		< median	2 (1)	0 (0)	2 (1)	0 (0)
	SCIG	>= median	0 (0)	0 (0)	0 (0)	0 (0)
		< median	0 (0)	0 (0)	0 (0)	0 (0)
Total	IVIG	>= median	22 (10)	20 (10)	2 (1)	0 (0)
		< median	23 (5)	17 (4)	6 (3)	0 (0)
	SCIG	>= median	11 (2)	11 (2)	0 (0)	0 (0)
		< median	3 (3)	3 (3)	0 (0)	0 (0)

The calculation of median BMI includes all patients participating in the study within the time period, whether or not they reported an infusion site reaction. For those patients who had an infusion site reaction within the time period, the mean weighted BMI for each site reaction is calculated for each patient. Otherwise, for those patients who did not have an infusion site reaction within the time period, the BMI for each patient is the mean of most relevant calculated BMIs for that patient.

Program: T0076.TEM, Version: 8.2, Datetime: 07FEB07:09:48

Table 14.3.3.10b: Summary of infusion site reactions by preferred term and severity - by prior therapy
>6 - 12 months Post-Subgam
Number of events (number of patients)

Preferred term	Prior Therapy	Median BMI = 23.4	n	Severity		
				Mild	Moderate	Severe
Total number of infusions for period	.		960			
Infusion site inflammation	IVIG	>= median	1 (1)	1 (1)	0 (0)	0 (0)
		< median	0 (0)	0 (0)	0 (0)	0 (0)
	SCIG	>= median	0 (0)	0 (0)	0 (0)	0 (0)
		< median	0 (0)	0 (0)	0 (0)	0 (0)
Infusion site pain	IVIG	>= median	1 (1)	1 (1)	0 (0)	0 (0)
		< median	1 (1)	1 (1)	0 (0)	0 (0)
	SCIG	>= median	0 (0)	0 (0)	0 (0)	0 (0)
		< median	0 (0)	0 (0)	0 (0)	0 (0)
Infusion site swelling	IVIG	>= median	2 (2)	2 (2)	0 (0)	0 (0)
		< median	0 (0)	0 (0)	0 (0)	0 (0)
	SCIG	>= median	0 (0)	0 (0)	0 (0)	0 (0)
		< median	0 (0)	0 (0)	0 (0)	0 (0)
Injection site bruising	IVIG	>= median	2 (1)	2 (1)	0 (0)	0 (0)
		< median	0 (0)	0 (0)	0 (0)	0 (0)
	SCIG	>= median	0 (0)	0 (0)	0 (0)	0 (0)
		< median	0 (0)	0 (0)	0 (0)	0 (0)

The calculation of median BMI includes all patients participating in the study within the time period, whether or not they reported an infusion site reaction. For those patients who had an infusion site reaction within the time period, the mean weighted BMI for each site reaction is calculated for each patient. Otherwise, for those patients who did not have an infusion site reaction within the time period, the BMI for each patient is the mean of most relevant calculated BMIs for that patient.

Program: T0077.TEM, Version: 8.2, Datetime: 07FEB07:09:48

Table 14.3.3.10b: Summary of infusion site reactions by preferred term and severity - by prior therapy
>6 - 12 months Post-Subgam
Number of events (number of patients)

				Severity		
Preferred term	Prior Therapy	Median BMI = 23.4	n	Mild	Moderate	Severe
Total	IVIG	>= median	6 (3)	6 (3)	0 (0)	0 (0)
		< median	1 (1)	1 (1)	0 (0)	0 (0)
	SCIG	>= median	0 (0)	0 (0)	0 (0)	0 (0)
		< median	0 (0)	0 (0)	0 (0)	0 (0)

The calculation of median BMI includes all patients participating in the study within the time period, whether or not they reported an infusion site reaction. For those patients who had an infusion site reaction within the time period, the mean weighted BMI for each site reaction is calculated for each patient. Otherwise, for those patients who did not have an infusion site reaction within the time period, the BMI for each patient is the mean of most relevant calculated BMIs for that patient.

Program: T0077.TEM, Version: 8.2, Datetime: 07FEB07:09:48

Table 14.3.3.10c: Summary of infusion site reactions by preferred term and severity - by prior therapy
>12 - 18 months Post-Subgam
Number of events (number of patients)

Preferred term	Prior Therapy	Median BMI = 23.0	n	Severity: Mild	Severity: Moderate	Severity: Severe
Total number of infusions for period			1,065			
Infusion site erythema	IVIG	>= median	0 (0)	0 (0)	0 (0)	0 (0)
		< median	0 (0)	0 (0)	0 (0)	0 (0)
	SCIG	>= median	0 (0)	0 (0)	0 (0)	0 (0)
		< median	2 (1)	2 (1)	0 (0)	0 (0)
Infusion site infection	IVIG	>= median	1 (1)	1 (1)	0 (0)	0 (0)
		< median	0 (0)	0 (0)	0 (0)	0 (0)
	SCIG	>= median	0 (0)	0 (0)	0 (0)	0 (0)
		< median	0 (0)	0 (0)	0 (0)	0 (0)
Infusion site inflammation	IVIG	>= median	2 (2)	2 (2)	0 (0)	0 (0)
		< median	0 (0)	0 (0)	0 (0)	0 (0)
	SCIG	>= median	0 (0)	0 (0)	0 (0)	0 (0)
		< median	0 (0)	0 (0)	0 (0)	0 (0)
Infusion site pain	IVIG	>= median	1 (1)	1 (1)	0 (0)	0 (0)
		< median	0 (0)	0 (0)	0 (0)	0 (0)
	SCIG	>= median	0 (0)	0 (0)	0 (0)	0 (0)
		< median	0 (0)	0 (0)	0 (0)	0 (0)

The calculation of median BMI includes all patients participating in the study within the time period, whether or not they reported an infusion site reaction. For those patients who had an infusion site reaction within the time period, the mean weighted BMI for each site reaction is calculated for each patient. Otherwise, for those patients who did not have an infusion site reaction within the time period, the BMI for each patient is the mean of most relevant calculated BMIs for that patient.

Program: T0078.TEM, Version: 8.2, Datetime: 07FEB07:09:48

Table 14.3.3.10c: Summary of infusion site reactions by preferred term and severity - by prior therapy
>12 - 18 months Post-Subgam
Number of events (number of patients)

				Severity		
Preferred term	Prior Therapy	Median BMI = 23.0	n	Mild	Moderate	Severe
Total	IVIG	>= median	4 (3)	4 (3)	0 (0)	0 (0)
		< median	0 (0)	0 (0)	0 (0)	0 (0)
	SCIG	>= median	0 (0)	0 (0)	0 (0)	0 (0)
		< median	2 (1)	2 (1)	0 (0)	0 (0)

The calculation of median BMI includes all patients participating in the study within the time period, whether or not they reported an infusion site reaction. For those patients who had an infusion site reaction within the time period, the mean weighted BMI for each site reaction is calculated for each patient. Otherwise, for those patients who did not have an infusion site reaction within the time period, the BMI for each patient is the mean of most relevant calculated BMIs for that patient.

Program: T0078.TEM, Version: 8.2, Datetime: 07FEB07:09:48

Table 14.3.3.10d: Summary of infusion site reactions by preferred term and severity - by prior therapy
>18 - 24 months Post-Subgam
Number of events (number of patients)

Preferred term	Prior Therapy	Median BMI = 23.0	n	Severity		
				Mild	Moderate	Severe
Total number of infusions for period	.		1,047			
Infusion site erythema	IVIG	>= median	0 (0)	0 (0)	0 (0)	0 (0)
		< median	1 (1)	1 (1)	0 (0)	0 (0)
	SCIG	>= median	0 (0)	0 (0)	0 (0)	0 (0)
		< median	0 (0)	0 (0)	0 (0)	0 (0)
Infusion site inflammation	IVIG	>= median	1 (1)	1 (1)	0 (0)	0 (0)
		< median	0 (0)	0 (0)	0 (0)	0 (0)
	SCIG	>= median	0 (0)	0 (0)	0 (0)	0 (0)
		< median	0 (0)	0 (0)	0 (0)	0 (0)
Total	IVIG	>= median	1 (1)	1 (1)	0 (0)	0 (0)
		< median	1 (1)	1 (1)	0 (0)	0 (0)
	SCIG	>= median	0 (0)	0 (0)	0 (0)	0 (0)
		< median	0 (0)	0 (0)	0 (0)	0 (0)

The calculation of median BMI includes all patients participating in the study within the time period, whether or not they reported an infusion site reaction. For those patients who had an infusion site reaction within the time period, the mean weighted BMI for each site reaction is calculated for each patient. Otherwise, for those patients who did not have an infusion site reaction within the time period, the BMI for each patient is the mean of most relevant calculated BMIs for that patient.

Program: T0079.TEM, Version: 8.2, Datetime: 07FEB07:09:48

Table 14.3.3.10e: Summary of infusion site reactions by preferred term and severity - by prior therapy
>24 - 30 months Post-Subgam
Number of events (number of patients)

Preferred term	Prior Therapy	Median BMI = 23.2	n	Severity		
				Mild	Moderate	Severe
Total number of infusions for period	.		1,039			
Infusion site tenderness	IVIG	>= median	0 (0)	0 (0)	0 (0)	0 (0)
		< median	0 (0)	0 (0)	0 (0)	0 (0)
	SCIG	>= median	1 (1)	0 (0)	1 (1)	0 (0)
		< median	0 (0)	0 (0)	0 (0)	0 (0)
Injection site stinging	IVIG	>= median	1 (1)	1 (1)	0 (0)	0 (0)
		< median	0 (0)	0 (0)	0 (0)	0 (0)
	SCIG	>= median	0 (0)	0 (0)	0 (0)	0 (0)
		< median	0 (0)	0 (0)	0 (0)	0 (0)
Total	IVIG	>= median	1 (1)	1 (1)	0 (0)	0 (0)
		< median	0 (0)	0 (0)	0 (0)	0 (0)
	SCIG	>= median	1 (1)	0 (0)	1 (1)	0 (0)
		< median	0 (0)	0 (0)	0 (0)	0 (0)

The calculation of median BMI includes all patients participating in the study within the time period, whether or not they reported an infusion site reaction. For those patients who had an infusion site reaction within the time period, the mean weighted BMI for each site reaction is calculated for each patient. Otherwise, for those patients who did not have an infusion site reaction within the time period, the BMI for each patient is the mean of most relevant calculated BMIs for that patient.

Program: T0080.TEM, Version: 8.2, Datetime: 07FEB07:09:48

Table 14.3.3.10f: Summary of infusion site reactions by preferred term and severity - by prior therapy
>30 - 36 months Post-Subgam
Number of events (number of patients)

Preferred term	Prior Therapy	Median BMI = 23.8	n	Severity: Mild	Moderate	Severe
Total number of infusions for period	.		879			
Infusion site infection	IVIG	>= median	1 (1)	1 (1)	0 (0)	0 (0)
		< median	0 (0)	0 (0)	0 (0)	0 (0)
	SCIG	>= median	0 (0)	0 (0)	0 (0)	0 (0)
		< median	0 (0)	0 (0)	0 (0)	0 (0)
Infusion site inflammation	IVIG	>= median	1 (1)	1 (1)	0 (0)	0 (0)
		< median	1 (1)	0 (0)	1 (1)	0 (0)
	SCIG	>= median	0 (0)	0 (0)	0 (0)	0 (0)
		< median	0 (0)	0 (0)	0 (0)	0 (0)
Infusion site pain	IVIG	>= median	1 (1)	1 (1)	0 (0)	0 (0)
		< median	0 (0)	0 (0)	0 (0)	0 (0)
	SCIG	>= median	0 (0)	0 (0)	0 (0)	0 (0)
		< median	0 (0)	0 (0)	0 (0)	0 (0)
Infusion site pruritus	IVIG	>= median	1 (1)	1 (1)	0 (0)	0 (0)
		< median	0 (0)	0 (0)	0 (0)	0 (0)
	SCIG	>= median	0 (0)	0 (0)	0 (0)	0 (0)
		< median	0 (0)	0 (0)	0 (0)	0 (0)

The calculation of median BMI includes all patients participating in the study within the time period, whether or not they reported an infusion site reaction. For those patients who had an infusion site reaction within the time period, the mean weighted BMI for each site reaction is calculated for each patient. Otherwise, for those patients who did not have an infusion site reaction within the time period, the BMI for each patient is the mean of most relevant calculated BMIs for that patient.

Program: T0081.TEM, Version: 8.2, Datetime: 07FEB07:09:48

Table 14.3.3.10f: Summary of infusion site reactions by preferred term and severity - by prior therapy
>30 - 36 months Post-Subgam
Number of events (number of patients)

Preferred term	Prior Therapy	Median BMI = 23.8	n	Severity: Mild	Severity: Moderate	Severity: Severe
Infusion site swelling	IVIG	>= median	0 (0)	0 (0)	0 (0)	0 (0)
		< median	1 (1)	1 (1)	0 (0)	0 (0)
	SCIG	>= median	0 (0)	0 (0)	0 (0)	0 (0)
		< median	0 (0)	0 (0)	0 (0)	0 (0)
Injection site discomfort	IVIG	>= median	0 (0)	0 (0)	0 (0)	0 (0)
		< median	1 (1)	1 (1)	0 (0)	0 (0)
	SCIG	>= median	0 (0)	0 (0)	0 (0)	0 (0)
		< median	0 (0)	0 (0)	0 (0)	0 (0)
Injection site stinging	IVIG	>= median	1 (1)	1 (1)	0 (0)	0 (0)
		< median	0 (0)	0 (0)	0 (0)	0 (0)
	SCIG	>= median	0 (0)	0 (0)	0 (0)	0 (0)
		< median	0 (0)	0 (0)	0 (0)	0 (0)
Total	IVIG	>= median	5 (4)	5 (4)	0 (0)	0 (0)
		< median	3 (1)	2 (1)	1 (1)	0 (0)
	SCIG	>= median	0 (0)	0 (0)	0 (0)	0 (0)
		< median	0 (0)	0 (0)	0 (0)	0 (0)

The calculation of median BMI includes all patients participating in the study within the time period, whether or not they reported an infusion site reaction. For those patients who had an infusion site reaction within the time period, the mean weighted BMI for each site reaction is calculated for each patient. Otherwise, for those patients who did not have an infusion site reaction within the time period, the BMI for each patient is the mean of most relevant calculated BMIs for that patient.

Program: T0081.TEM, Version: 8.2, Datetime: 07FEB07:09:48

Table 14.3.3.10g: Summary of infusion site reactions by preferred term and severity - by prior therapy
>36 - 42 months Post-Subgam
Number of events (number of patients)

```
/******************************************************************************/
/*                                                                            */
/*                                                                            */
/*  No observations met the criteria of the current report.                   */
/*                                                                            */
/*                                                                            */
/*                                                                            */
/******************************************************************************/
```

The calculation of median BMI includes all patients participating in the study within the time period, whether or not they reported an infusion site reaction. For those patients who had an infusion site reaction within the time period, the mean weighted BMI for each site reaction is calculated for each patient. Otherwise, for those patients who did not have an infusion site reaction within the time period, the BMI for each patient is the mean of most relevant calculated BMIs for that patient.

Program: T0082.TEM, Version: 8.2, Datetime: 07FEB07:09:48

Table 14.3.3.10h: Summary of infusion site reactions by preferred term and severity - by prior therapy
>42 - 48 months Post-Subgam
Number of events (number of patients)

```
/*************************************************************************************/
/*                                                                                  */
/*                                                                                  */
/*   No observations met the criteria of the current report.                        */
/*                                                                                  */
/*                                                                                  */
/*                                                                                  */
/*************************************************************************************/
```

The calculation of median BMI includes all patients participating in the study within the time period, whether or not they reported an infusion site reaction. For those patients who had an infusion site reaction within the time period, the mean weighted BMI for each site reaction is calculated for each patient. Otherwise, for those patients who did not have an infusion site reaction within the time period, the BMI for each patient is the mean of most relevant calculated BMIs for that patient.

Program: T0083.TEM, Version: 8.2, Datetime: 07FEB07:09:48

Table 14.3.3.10i: Summary of infusion site reactions by preferred term and severity - by prior therapy
>48 - 54 months Post-Subgam
Number of events (number of patients)

```
/******************************************************************************************/
/*                                                                                        */
/*                                                                                        */
/*   No observations met the criteria of the current report.                              */
/*                                                                                        */
/*                                                                                        */
/*                                                                                        */
/******************************************************************************************/
```

The calculation of median BMI includes all patients participating in the study within the time period, whether or not they reported an infusion site reaction. For those patients who had an infusion site reaction within the time period, the mean weighted BMI for each site reaction is calculated for each patient. Otherwise, for those patients who did not have an infusion site reaction within the time period, the BMI for each patient is the mean of most relevant calculated BMIs for that patient.

Program: T0084.TEM, Version: 8.2, Datetime: 07FEB07:09:48

Table 14.3.4.1: Summary statistics for haematology parameters

Parameter	Visit	n	Mean	Median	SD	Min	Max	Lower 95% CI	Upper 95% CI
Haemoglobin (g/dL)	Pre-study	50	13.4	13.5	1.4	9.7	16.1	13.0	13.8
	EOP1	43	13.5	13.7	1.4	10.8	16.6	13.1	14.0
Haematocrit (%)	Pre-study	47	39.6	40.0	4.2	30.0	48.0	38.3	40.8
	EOP1	43	40.3	40.0	4.3	33.0	49.0	39.0	41.7
Erythrocytes (x10*12/L)	Pre-study	50	4.7	4.6	0.5	3.6	5.9	4.6	4.8
	EOP1	43	4.7	4.7	0.5	3.3	6.0	4.6	4.9
Leukocytes (x10*9/L)	Pre-study	50	6.9	6.3	3.0	2.2	17.8	6.1	7.7
	EOP1	43	7.2	6.2	3.1	1.9	15.9	6.2	8.1
Neutrophils (x10*9/L)	Pre-study	50	3.7	3.6	1.5	1.0	7.4	3.3	4.1
	EOP1	43	4.2	3.7	2.3	0.8	12.8	3.5	4.9
Lymphocytes (x10*9/L)	Pre-study	50	2.4	1.9	1.8	0.6	9.8	1.9	2.9
	EOP1	43	2.1	1.8	1.5	0.8	8.8	1.7	2.6
Monocytes (x10*9/L)	Pre-study	50	0.49	0.42	0.28	0.15	1.38	0.41	0.57
	EOP1	43	0.50	0.47	0.23	0.17	1.20	0.43	0.57
Eosinophils (x10*9/L)	Pre-study	50	0.23	0.17	0.21	0.00	0.96	0.17	0.29
	EOP1	42	0.24	0.13	0.31	0.00	1.85	0.14	0.33
Basophils (x10*9/L)	Pre-study	43	0.04	0.04	0.05	0.00	0.26	0.03	0.05
	EOP1	39	0.05	0.03	0.05	0.00	0.22	0.03	0.06
Platelets (x10*9/L)	Pre-study	49	286.8	267.0	110.7	89.0	603.0	255.0	318.6
	EOP1	43	276.7	266.0	96.1	97.0	484.0	247.1	306.3

EOP1 = End of Stage 1

Program: T0444.SAS, Version: 8.2, Datetime: 07FEB07:09:48

Table 14.3.4.1: Summary statistics for haematology parameters

Parameter	Visit	n	Mean	Median	SD	Min	Max	Lower 95% CI	Upper 95% CI
Reticulocytes (x10*9/L)	Pre-study	33	64.3	54.1	31.7	23.0	159.6	53.0	75.5
	EOP1	20	62.8	51.0	36.3	18.3	146.2	45.8	79.7

EOP1 = End of Stage 1

Program: T0444.SAS, Version: 8.2, Datetime: 07FEB07:09:48

Table 14.3.4.2: Summary statistics for biochemistry parameters

Parameter	Visit	n	Mean	Median	SD	Min	Max	Lower 95% CI	Upper 95% CI
Sodium (mmol/L)	Pre-study	48	139.9	140.0	2.0	135.0	144.0	139.3	140.4
	EOP1	36	140.3	140.0	2.0	137.0	145.0	139.6	141.0
Potassium (mmol/L)	Pre-study	47	4.2	4.1	0.4	3.4	6.0	4.1	4.4
	EOP1	35	4.3	4.2	0.5	3.6	5.9	4.1	4.4
Creatinine (umol/L)	Pre-study	48	66.0	71.5	20.1	25.0	106.0	60.2	71.8
	EOP1	36	67.4	67.0	22.0	27.0	122.0	60.0	74.9
LDH (U/L)	Pre-study	36	504.0	436.0	372.5	117.0	2017	378.0	630.0
	EOP1	22	508.3	523.5	264.6	121.0	964.0	391.0	625.6
GGT (U/L)	Pre-study	41	29.4	19.0	35.0	6.0	210.0	18.3	40.4
	EOP1	32	28.4	17.0	29.3	4.0	150.0	17.8	38.9
Total bilirubin (umol/L)	Pre-study	47	9.1	9.0	4.7	1.0	26.0	7.7	10.5
	EOP1	41	8.3	7.0	4.0	2.0	19.0	7.1	9.6
Alkaline phosphatase (U/L)	Pre-study	48	172.8	117.5	138.0	40.0	662.0	132.7	212.8
	EOP1	42	157.6	109.0	124.0	41.0	527.0	119.0	196.2
Haptoglobin (g/L)	Pre-study	36	1.4	1.2	0.7	0.3	3.6	1.1	1.6
	EOP1	18	1.2	1.2	0.6	0.0	2.0	0.9	1.5

EOP1 = End of Stage 1

Program: T0445.SAS, Version: 8.2, Datetime: 07FEB07:09:48

Table 14.3.4.3: Summary statistics for ALT (U/L)

Infusion No	n	Mean	Median	SD	Min	Max	Lower 95% CI	Upper 95% CI
Pre-study	43	21.1	20.0	11.0	5.0	52.0	17.7	24.5
1	1	18.0	18.0	.	18.0	18.0	.	.
2	5	19.8	22.0	7.5	8.0	28.0	10.5	29.1
3	6	21.2	22.0	12.5	5.0	39.0	8.1	34.3
4	40	23.9	23.0	9.7	9.0	55.0	20.8	27.0
5	6	23.5	20.5	13.0	13.0	49.0	9.9	37.1
6	1	40.0	40.0	.	40.0	40.0	.	.
7	4	18.0	18.0	7.4	9.0	27.0	6.2	29.8
8	23	20.8	17.0	11.6	5.0	53.0	15.8	25.8
9	10	26.3	21.0	16.6	10.0	68.0	14.4	38.2
10	3	30.0	26.0	8.7	24.0	40.0	8.3	51.7
11	4	13.0	13.0	4.1	8.0	18.0	6.5	19.5
12	23	19.4	18.0	9.2	5.0	40.0	15.4	23.4
13	4	28.3	29.0	12.7	12.0	43.0	8.0	48.5
14	2	21.0	21.0	2.8	19.0	23.0	-4.4	46.4
15	8	29.0	34.5	13.9	8.0	42.0	17.4	40.6
16	22	22.1	20.5	11.5	8.0	58.0	17.0	27.2
17	3	15.7	15.0	1.2	15.0	17.0	12.8	18.5
18	5	32.2	34.0	15.9	9.0	48.0	12.5	51.9
19	5	24.8	22.0	11.0	17.0	44.0	11.1	38.5
20	20	21.6	22.5	10.1	6.0	43.0	16.9	26.3
21	4	24.5	25.0	12.4	9.0	39.0	4.8	44.2
22	7	34.7	31.0	18.8	11.0	73.0	17.3	52.1
23	5	18.6	21.0	11.9	5.0	35.0	3.8	33.4
24	19	22.7	22.0	10.7	7.0	43.0	17.6	27.9
25	3	17.7	18.0	3.5	14.0	21.0	8.9	26.4
26	4	23.8	24.0	8.6	13.0	34.0	10.1	37.4
27	5	22.6	20.0	12.5	9.0	40.0	7.1	38.1
28	20	25.7	20.5	22.4	5.0	113.0	15.2	36.1
29	3	18.0	13.0	9.5	12.0	29.0	-5.7	41.7

EOP1 = End of Stage 1

Program: T0446.TEM, Version: 8.2, Datetime: 07FEB07:09:48

Table 14.3.4.3: Summary statistics for ALT (U/L)

Infusion No	n	Mean	Median	SD	Min	Max	Lower 95% CI	Upper 95% CI
30	3	31.0	28.0	7.0	26.0	39.0	13.6	48.4
EOP1	42	24.6	22.0	13.4	6.0	77.0	20.4	28.8
All infusions	353	23.1	22.0	12.4	5.0	113.0	21.8	24.4

EOP1 = End of Stage 1

Program: T0446.TEM, Version: 8.2, Datetime: 07FEB07:09:48

Table 14.3.4.4: Summary statistics for AST (U/L)

Infusion No	n	Mean	Median	SD	Min	Max	Lower 95% CI	Upper 95% CI
Pre-study	27	26.7	25.0	7.7	17.0	47.0	23.7	29.8
1	1	37.0	37.0	.	37.0	37.0	.	.
2	5	28.6	25.0	8.4	21.0	40.0	18.1	39.1
3	6	25.7	25.0	3.6	22.0	32.0	21.9	29.4
4	29	26.8	25.0	7.4	18.0	50.0	24.0	29.6
5	2	33.5	33.5	2.1	32.0	35.0	14.4	52.6
6	1	21.0	21.0	.	21.0	21.0	.	.
7	4	25.8	25.5	6.7	18.0	34.0	15.2	36.3
8	16	25.5	23.0	6.8	17.0	39.0	21.9	29.1
9	5	31.0	29.0	7.7	24.0	44.0	21.4	40.6
10	2	32.5	32.5	10.6	25.0	40.0	-62.8	127.8
11	4	24.0	25.0	2.7	20.0	26.0	19.7	28.3
12	17	24.9	27.0	5.9	16.0	36.0	21.9	27.9
13	3	33.3	35.0	4.7	28.0	37.0	21.6	45.1
14	2	26.5	26.5	12.0	18.0	35.0	-81.5	134.5
15	5	32.0	37.0	10.2	17.0	41.0	19.3	44.7
16	13	22.9	21.0	5.9	16.0	33.0	19.4	26.5
17	2	22.0	22.0	1.4	21.0	23.0	9.3	34.7
18	4	37.3	41.5	13.2	18.0	48.0	16.2	58.3
19	3	28.0	29.0	3.6	24.0	31.0	19.0	37.0
20	13	27.8	29.0	5.3	21.0	38.0	24.6	31.0
21	2	31.5	31.5	13.4	22.0	41.0	-89.2	152.2
22	5	26.4	25.0	8.7	16.0	40.0	15.6	37.2
23	2	22.5	22.5	2.1	21.0	24.0	3.4	41.6
24	14	30.1	28.0	8.5	19.0	44.0	25.2	35.1
25	3	28.0	26.0	5.3	24.0	34.0	14.9	41.1
26	3	23.3	25.0	5.7	17.0	28.0	9.2	37.5
27	3	22.0	23.0	5.6	16.0	27.0	8.2	35.8
28	14	42.1	28.5	50.3	18.0	214.0	13.0	71.1
29	3	22.7	21.0	7.6	16.0	31.0	3.7	41.6

EOP1 = End of Stage 1

Program: T0447.TEM, Version: 8.2, Datetime: 07FEB07:09:48

Table 14.3.4.4: Summary statistics for AST (U/L)

Infusion No	n	Mean	Median	SD	Min	Max	Lower 95% CI	Upper 95% CI
30	4	27.3	28.0	7.0	18.0	35.0	16.0	38.5
EOP1	29	28.7	27.0	11.6	16.0	78.0	24.3	33.1
All infusions	246	28.0	26.0	14.4	16.0	214.0	26.2	29.9

EOP1 = End of Stage 1

Program: T0447.TEM, Version: 8.2, Datetime: 07FEB07:09:48

Table 14.3.4.5: Pre-Subgam value versus end of Stage 1 value for ALT (U/L)

	End of Stage 1 value Number of events (patient number*)			
Pre-Subgam value	Above Normal Range	Within Normal Range	Below Normal Range	Not Done
Above Normal Range	0	1 (21)	0	0
Within Normal Range	5 (34, 58, 73, 81, 84)	33	0	2 (31, 53)
Below Normal Range	0	1 (86)	0	1 (13)
Not Done	0	2 (12, 54)	0	5 (29, 30, 55, 61, 77)

*Where either pre-Subgam or end of Stage 1 result is outside the normal range or not done

Program: T0448.TEM, Version: 8.2, Datetime: 07FEB07:09:48

Table 14.3.4.6: Pre-Subgam value versus end of Stage 1 value for AST (U/L)

	End of Stage 1 value<br>Number of events (patient number*)			
Pre-Subgam value	Above Normal Range	Within Normal Range	Below Normal Range	Not Done
Above Normal Range	0	0	0	1<br>(21)
Within Normal Range	1<br>(73)	23	0	16<br>(01, 22, 23, 24, 31,<br>33, 34, 35, 41, 49, 78,<br>81, 82, 83, 84, 85)
Below Normal Range	0	0	0	2<br>(13, 86)
Not Done	0	5<br>(12, 29, 54, 55, 61)	0	2<br>(30, 77)

*Where either pre-Subgam or end of Stage 1 result is outside the normal range or not done

Program: T0449.TEM, Version: 8.2, Datetime: 07FEB07:09:48

Table 14.3.4.7: Pre-Subgam value versus end of study / withdrawal value for HbsAg

Pre-Subgam Virology Result	End of Study / Withdrawal Virology Result Number of events (patient number*)		
	Negative	Positive	Not done / Missing
Negative	7	0	0
Positive	0	0	0
Not Done / Missing	43 (01, 05, 06, 07, 08, 11, 12, 13, 17, 18, 21, 22, 23, 24, 26, 27, 28, 29, 30, 31, 33, 34, 35, 49, 53, 54, 55, 56, 57, 58, 61, 65, 73, 74, 75, 76, 78, 81, 82, 83, 84, 85, 86)	0	0

*Where end of study / withdrawal result is positive or not done / missing

Program: T0450.TEM, Version: 8.2, Datetime: 07FEB07:09:48

Table 14.3.4.8: Pre-Subgam value versus end of study / withdrawal value for HCV

Pre-Subgam Virology Result	End of Study / Withdrawal Virology Result Number of events (patient number*)		
	Not detected	Detected	Not done / Missing
Not detected	7	0	0
Detected	0	0	0
Not Done / Missing	43 (01, 05, 06, 07, 08, 11, 12, 13, 17, 18, 21, 22, 23, 24, 26, 27, 28, 29, 30, 31, 33, 34, 35, 49, 53, 54, 55, 56, 57, 58, 61, 65, 73, 74, 75, 76, 78, 81, 82, 83, 84, 85, 86)	0	0

*where either pre-Subgam or end of study result is detected or not done

Program: T0452.TEM, Version: 8.2, Datetime: 07FEB07:09:48

Table 14.3.4.9: Pre-Subgam value versus end of study value for Parvovirus B19 (PCR)

Pre-Subgam Virology Result	Post-Subgam Virology Result Number of events (patient number*)		
	Negative	Positive	Not done / Missing
Negative	46	0	1 (23)
Positive	0	0	0
Not Done / Missing	2 (57, 58)	0	1 (24)

*where either pre-Subgam or post-Subgam result is positive or not done / missing

Program: T0453.TEM, Version: 8.2, Datetime: 07FEB07:09:48

Table 14.3.5.1: Systolic blood pressure - summary statistics for Stage 1 - all infusions, mmHg

Infusion	Observation	n	Mean	Median	SD	Min	Max	Lower 95% CI	Upper 95% CI
PRE		49	119.00	116.00	21.02	81.00	170.00	112.96	125.04
1	Pre-infusion	48	118.65	114.50	20.81	81.00	170.00	112.60	124.69
	1	47	121.17	120.00	20.72	85.00	169.00	115.09	127.25
	2	46	120.33	118.50	17.87	90.00	168.00	115.02	125.63
	3	37	122.89	120.00	17.22	91.00	159.00	117.15	128.63
	4	20	123.50	122.50	15.87	95.00	151.00	116.07	130.93
	5	6	118.67	119.50	11.94	99.00	131.00	106.13	131.20
	6	4	116.50	115.50	8.89	107.00	128.00	102.36	130.64
	7	2	115.50	115.50	3.54	113.00	118.00	83.73	147.27
	8	1	102.00	102.00		102.00	102.00		
2	Pre-infusion	45	122.00	118.00	19.80	86.00	162.00	116.05	127.95
	1	44	120.89	116.50	19.72	90.00	162.00	114.89	126.88
	2	44	120.02	112.00	20.42	90.00	180.00	113.82	126.23
	3	36	120.28	114.50	21.45	68.00	165.00	113.02	127.53
	4	19	122.89	124.00	18.96	89.00	155.00	113.75	132.04
	5	8	110.88	109.50	10.48	95.00	130.00	102.11	119.64
	6	3	106.00	110.00	8.72	96.00	112.00	84.34	127.66
	7	1	95.00	95.00		95.00	95.00		
3	Pre-infusion	47	116.30	115.00	17.22	88.00	177.00	111.24	121.35
	1	46	118.15	117.50	17.26	91.00	183.00	113.03	123.28
	2	45	117.62	116.00	16.81	87.00	175.00	112.57	122.67
	3	35	121.57	117.00	19.01	95.00	180.00	115.04	128.10
	4	16	116.31	122.00	13.76	92.00	135.00	108.98	123.64
	5	7	121.71	118.00	16.90	101.00	148.00	106.09	137.34
	6	3	114.33	101.00	23.97	100.00	142.00	54.80	173.87

PRE = Pre-study Visit
EOP1 = End of Stage 1

Program: T0454.TEM, Version: 8.2, Datetime: 07FEB07:09:48

Table 14.3.5.1: Systolic blood pressure - summary statistics for Stage 1 - all infusions, mmHg

Infusion	Observation	n	Mean	Median	SD	Min	Max	Lower 95% CI	Upper 95% CI
3	7	3	113.67	104.00	19.40	101.00	136.00	65.48	161.86

PRE = Pre-study Visit
EOP1 = End of Stage 1

Program: T0454.TEM, Version: 8.2, Datetime: 07FEB07:09:48

Table 14.3.5.1: Systolic blood pressure - summary statistics for Stage 1 - all infusions, mmHg

Infusion	Observation	n	Mean	Median	SD	Min	Max	Lower 95% CI	Upper 95% CI
3A	Pre-infusion	2	152.50	152.50	7.78	147.00	158.00	82.62	222.38
	1	2	151.00	151.00	18.38	138.00	164.00	-14.18	316.18
	2	2	139.50	139.50	12.02	131.00	148.00	31.50	247.50
	3	2	148.50	148.50	0.71	148.00	149.00	142.15	154.85
3B	Pre-infusion	2	136.50	136.50	16.26	125.00	148.00	-9.62	282.62
	1	2	136.50	136.50	16.26	125.00	148.00	-9.62	282.62
	2	2	140.50	140.50	2.12	139.00	142.00	121.44	159.56
	3	2	150.00	150.00	11.31	142.00	158.00	48.35	251.65
	4	2	130.00	130.00	12.73	121.00	139.00	15.64	244.36
	5	1	138.00	138.00		138.00	138.00		
4	Pre-infusion	50	119.82	115.50	17.33	93.00	169.00	114.89	124.75
	1	50	120.18	116.00	17.28	93.00	169.00	115.27	125.09
	2	49	119.65	115.00	16.13	96.00	157.00	115.02	124.29
	3	43	121.60	119.00	16.52	96.00	168.00	116.52	126.69
	4	29	121.52	116.00	18.36	95.00	166.00	114.53	128.50
	5	8	135.13	131.50	24.93	109.00	188.00	114.29	155.96
	6	2	131.50	131.50	12.02	123.00	140.00	23.50	239.50
5	Pre-infusion	48	120.00	119.50	19.12	80.00	199.00	114.45	125.55
	1	45	118.07	115.00	19.70	82.00	188.00	112.15	123.98
	2	39	119.64	115.00	18.82	94.00	192.00	113.54	125.74
	3	17	122.12	119.00	23.93	92.00	187.00	109.81	134.42
	4	3	128.33	121.00	19.09	114.00	150.00	80.92	175.75
	5	1	130.00	130.00		130.00	130.00		

PRE = Pre-study Visit
EOP1 = End of Stage 1

Program: T0454.TEM, Version: 8.2, Datetime: 07FEB07:09:48

Table 14.3.5.1: Systolic blood pressure - summary statistics for Stage 1 - all infusions, mmHg

Infusion	Observation	n	Mean	Median	SD	Min	Max	Lower 95% CI	Upper 95% CI
6	Pre-infusion	46	119.30	114.50	21.57	75.00	183.00	112.90	125.71
	1	43	118.44	114.00	17.54	86.00	161.00	113.04	123.84
	2	31	121.23	120.00	18.55	94.00	175.00	114.42	128.03
	3	13	124.46	120.00	25.64	83.00	186.00	108.97	139.96
	4	1	140.00	140.00		140.00	140.00		
7	Pre-infusion	40	120.93	120.00	20.45	81.00	174.00	114.39	127.46
	1	40	119.15	118.50	19.90	81.00	167.00	112.79	125.51
	2	32	121.09	121.00	18.53	96.00	160.00	114.41	127.77
	3	10	127.10	121.00	23.32	105.00	181.00	110.42	143.78
	4	2	117.50	117.50	3.54	115.00	120.00	85.73	149.27
8	Pre-infusion	37	120.81	117.00	19.13	83.00	172.00	114.43	127.19
	1	36	119.47	117.00	17.48	94.00	167.00	113.56	125.39
	2	25	127.12	125.00	21.42	79.00	177.00	118.28	135.96
	3	7	132.14	128.00	23.22	104.00	174.00	110.67	153.62
	4	2	157.00	157.00	9.90	150.00	164.00	68.06	245.94
9	Pre-infusion	27	124.37	119.00	22.04	87.00	182.00	115.65	133.09
	1	25	123.24	115.00	22.65	92.00	171.00	113.89	132.59
	2	23	123.04	121.00	19.00	91.00	166.00	114.83	131.26
	3	7	121.43	120.00	28.25	90.00	175.00	95.30	147.56
	4	2	150.00	150.00	42.43	120.00	180.00	-231.19	531.19
10	Pre-infusion	26	123.42	122.00	18.66	90.00	178.00	115.89	130.96
	1	24	127.83	123.50	20.94	94.00	174.00	118.99	136.68
	2	19	128.84	125.00	21.40	100.00	173.00	118.53	139.16
	3	5	137.40	140.00	12.40	120.00	150.00	122.00	152.80

PRE = Pre-study Visit
EOP1 = End of Stage 1

Table 14.3.5.1: Systolic blood pressure - summary statistics for Stage 1 - all infusions, mmHg

Infusion	Observation	n	Mean	Median	SD	Min	Max	Lower 95% CI	Upper 95% CI
10	4	1	130.00	130.00		130.00	130.00		

PRE = Pre-study Visit
EOP1 = End of Stage 1

Program: T0454.TEM, Version: 8.2, Datetime: 07FEB07:09:48

Table 14.3.5.1: Systolic blood pressure - summary statistics for Stage 1 - all infusions, mmHg

Infusion	Observation	n	Mean	Median	SD	Min	Max	Lower 95% CI	Upper 95% CI
11	Pre-infusion	24	122.00	121.50	23.26	70.00	176.00	112.18	131.82
	1	21	123.38	119.00	21.17	88.00	173.00	113.74	133.02
	2	20	123.75	120.00	23.00	94.00	183.00	112.99	134.51
	3	6	133.33	133.50	22.76	99.00	169.00	109.45	157.22
	4	1	135.00	135.00		135.00	135.00		
12	Pre-infusion	19	116.89	111.00	23.62	90.00	174.00	105.51	128.28
	1	18	119.94	112.00	21.06	95.00	169.00	109.47	130.42
	2	13	125.69	120.00	21.49	99.00	174.00	112.70	138.68
	3	3	140.00	131.00	35.37	110.00	179.00	52.14	227.86
	4	1	120.00	120.00		120.00	120.00		
13	Pre-infusion	9	126.89	122.00	21.27	91.00	165.00	110.54	143.24
	1	9	128.33	125.00	20.74	110.00	173.00	112.39	144.27
	2	7	132.43	128.00	20.06	116.00	175.00	113.88	150.98
	3	3	144.00	131.00	32.51	120.00	181.00	63.24	224.76
	4	1	120.00	120.00		120.00	120.00		
14	Pre-infusion	8	129.38	124.50	18.07	104.00	156.00	114.27	144.48
	1	8	124.75	115.50	23.48	104.00	175.00	105.12	144.38
	2	6	129.50	124.00	19.41	106.00	161.00	109.13	149.87
	3	4	138.75	136.00	22.11	115.00	168.00	103.57	173.93
	4	1	120.00	120.00		120.00	120.00		
15	Pre-infusion	11	120.82	114.00	22.59	92.00	175.00	105.64	135.99
	1	10	119.70	115.50	17.98	100.00	163.00	106.84	132.56
	2	8	122.75	114.00	23.44	98.00	171.00	103.15	142.35
	3	2	155.00	155.00	49.50	120.00	190.00	-289.72	599.72

PRE = Pre-study Visit
EOP1 = End of Stage 1

Program: T0454.TEM, Version: 8.2, Datetime: 07FEB07:09:48

Table 14.3.5.1: Systolic blood pressure - summary statistics for Stage 1 - all infusions, mmHg

Infusion	Observation	n	Mean	Median	SD	Min	Max	Lower 95% CI	Upper 95% CI
15	4	1	130.00	130.00		130.00	130.00		

PRE = Pre-study visit
EOP1 = End of Stage 1

Program: T0454.TEM, Version: 8.2, Datetime: 07FEB07:09:48

Table 14.3.5.1: Systolic blood pressure - summary statistics for Stage 1 - all infusions, mmHg

Infusion	Observation	n	Mean	Median	SD	Min	Max	Lower 95% CI	Upper 95% CI
16	Pre-infusion	16	118.69	112.00	22.27	91.00	175.00	106.82	130.55
	1	14	115.93	114.00	18.68	90.00	150.00	105.14	126.71
	2	6	129.00	123.50	28.93	96.00	177.00	98.64	159.36
	3	2	154.00	154.00	55.15	115.00	193.00	-341.54	649.54
	4	1	120.00	120.00		120.00	120.00		
17	Pre-infusion	5	133.20	124.00	32.40	97.00	180.00	92.97	173.43
	1	4	130.00	117.00	30.23	111.00	175.00	81.89	178.11
	2	2	145.50	145.50	21.92	130.00	161.00	-51.45	342.45
	3	2	135.00	135.00	35.36	110.00	160.00	-182.66	452.66
	4	1	120.00	120.00		120.00	120.00		
18	Pre-infusion	4	136.50	138.50	34.80	92.00	177.00	81.13	191.87
	1	4	125.50	113.50	26.39	110.00	165.00	83.51	167.49
	2	2	127.00	127.00	24.04	110.00	144.00	-89.01	343.01
	3	2	140.00	140.00	42.43	110.00	170.00	-241.19	521.19
	4	1	115.00	115.00		115.00	115.00		
19	Pre-infusion	6	123.50	115.50	29.37	99.00	179.00	92.68	154.32
	1	7	130.29	131.00	27.16	94.00	178.00	105.17	155.40
	2	4	127.75	121.00	30.14	100.00	169.00	79.80	175.70
	3	3	133.00	120.00	28.79	113.00	166.00	61.48	204.52
	4	1	130.00	130.00		130.00	130.00		
20	Pre-infusion	12	115.17	114.00	23.59	80.00	160.00	100.18	130.16
	1	10	113.50	101.50	28.86	88.00	169.00	92.85	134.15
	2	3	141.33	156.00	40.07	96.00	172.00	41.80	240.86
	3	1	180.00	180.00		180.00	180.00		

PRE = Pre-study Visit
EOP1 = End of Stage 1

Program: T0454.TEM, Version: 8.2, Datetime: 07FEB07:09:48

Table 14.3.5.1: Systolic blood pressure - summary statistics for Stage 1 - all infusions, mmHg

Infusion	Observation	n	Mean	Median	SD	Min	Max	Lower 95% CI	Upper 95% CI
21	Pre-infusion	4	137.75	140.50	33.90	101.00	169.00	83.81	191.69
	1	4	138.00	135.50	32.51	101.00	180.00	86.28	189.72
	2	2	155.00	155.00	14.14	145.00	165.00	27.94	282.06
	3	1	174.00	174.00		174.00	174.00		
22	Pre-infusion	4	136.75	132.00	37.64	97.00	186.00	76.85	196.65
	1	3	126.00	117.00	34.39	97.00	164.00	40.56	211.44
	2	2	131.00	131.00	48.08	97.00	165.00	-301.01	563.01
	3	2	117.50	117.50	36.06	92.00	143.00	-206.51	441.51
23	Pre-infusion	5	125.80	122.00	31.98	94.00	167.00	86.09	165.51
	1	4	146.25	154.50	27.42	107.00	169.00	102.63	189.87
	2	2	156.50	156.50	3.54	154.00	159.00	124.73	188.27
	3	2	163.00	163.00	5.66	159.00	167.00	112.18	213.82
24	Pre-infusion	9	114.56	113.00	25.38	87.00	166.00	95.05	134.06
	1	8	120.50	110.00	30.75	91.00	183.00	94.79	146.21
	2	3	142.33	162.00	37.58	99.00	166.00	48.98	235.69
	3	1	176.00	176.00		176.00	176.00		
25	Pre-infusion	4	123.25	119.50	21.82	103.00	151.00	88.52	157.98
	1	4	138.25	136.50	9.95	130.00	150.00	122.42	154.08
	2	2	163.00	163.00	18.38	150.00	176.00	-2.18	328.18
	3	1	160.00	160.00		160.00	160.00		
26	Pre-infusion	4	130.25	140.50	27.45	90.00	150.00	86.57	173.93
	1	4	124.75	126.00	25.24	96.00	151.00	84.59	164.91
	2	3	132.33	150.00	35.02	92.00	155.00	45.34	219.33

PRE = Pre-study Visit
EOP1 = End of Stage 1

Program: T0454.TEM, Version: 8.2, Datetime: 07FEB07:09:48

Table 14.3.5.1: Systolic blood pressure - summary statistics for Stage 1 - all infusions, mmHg

Infusion	Observation	n	Mean	Median	SD	Min	Max	Lower 95% CI	Upper 95% CI
26	3	1	140.00	140.00		140.00	140.00		

PRE = Pre-study Visit
EOP1 = End of Stage 1

Program: T0454.TEM, Version: 8.2, Datetime: 07FEB07:09:48

Table 14.3.5.1: Systolic blood pressure - summary statistics for Stage 1 - all infusions, mmHg

Infusion	Observation	n	Mean	Median	SD	Min	Max	Lower 95% CI	Upper 95% CI
27	Pre-infusion	5	136.60	132.00	28.39	106.00	172.00	101.35	171.85
	1	4	139.50	140.00	28.49	108.00	170.00	94.17	184.83
	2	3	126.67	140.00	27.54	95.00	145.00	58.26	195.07
	3	2	136.00	136.00	48.08	102.00	170.00	-296.01	568.01
28	Pre-infusion	11	113.00	108.00	23.60	82.00	161.00	97.14	128.86
	1	10	114.90	107.50	24.77	88.00	164.00	97.18	132.62
	2	2	153.50	153.50	19.09	140.00	167.00	-18.03	325.03
	3	2	154.50	154.50	20.51	140.00	169.00	-29.74	338.74
29	Pre-infusion	4	142.00	140.50	23.05	120.00	167.00	105.32	178.68
	1	4	133.75	135.50	23.20	109.00	155.00	96.83	170.67
	2	2	163.50	163.50	17.68	151.00	176.00	4.67	322.33
	3	1	168.00	168.00		168.00	168.00		
30	Pre-infusion	3	115.67	130.00	26.58	85.00	132.00	49.65	181.69
	1	4	135.50	139.50	25.23	103.00	160.00	95.36	175.64
	2	3	126.67	140.00	39.72	82.00	158.00	28.01	225.33
	3	1	130.00	130.00		130.00	130.00		
EOP1		50	121.38	120.00	17.19	85.00	180.00	116.49	126.27

PRE = Pre-study Visit
EOP1 = End of Stage 1

Program: T0454.TEM, Version: 8.2, Datetime: 07FEB07:09:48

Table 14.3.5.2: Systolic blood pressure - summary statistics for Stage 1 - Infusions 1-3 (pre-Subgam), mmHg

Observation	n	Mean	Median	SD	Min	Max	Lower 95% CI	Upper 95% CI
Pre-infusion	144	119.65	116.50	19.63	81.00	177.00	116.41	122.88
1	141	120.74	118.00	19.48	85.00	183.00	117.49	123.98
2	139	119.92	117.00	18.40	87.00	180.00	116.83	123.01
3	112	122.58	119.50	19.51	68.00	180.00	118.93	126.23
4	57	121.51	123.00	16.34	89.00	155.00	117.17	125.84
5	22	117.68	115.50	13.97	95.00	148.00	111.49	123.88
6	10	112.70	111.00	13.90	96.00	142.00	102.76	122.64
7	6	111.17	108.50	14.72	95.00	136.00	95.72	126.61
8	1	102.00	102.00		102.00	102.00		

Table 14.3.5.3: Systolic blood pressure - summary statistics for Stage 1 - Infusions 4-30 (post-Subgam), mmHg

Observation	n	Mean	Median	SD	Min	Max	Lower 95% CI	Upper 95% CI
Pre-infusion	441	121.58	119.00	21.64	70.00	199.00	119.55	123.60
1	417	121.67	116.00	20.93	81.00	188.00	119.66	123.69
2	313	124.91	121.00	21.28	79.00	192.00	122.54	127.27
3	144	129.86	122.50	24.68	83.00	193.00	125.80	133.93
4	49	125.27	120.00	18.54	95.00	180.00	119.94	130.59
5	9	134.56	130.00	23.38	109.00	188.00	116.59	152.53
6	2	131.50	131.50	12.02	123.00	140.00	23.50	239.50

Table 14.3.5.4: Systolic blood pressure - summary statistics for Stage 2 (post-Subgam), mmHg

Visit number	n	Mean	Median	SD	Min	Max	Lower 95% CI	Upper 95% CI
1	36	122.28	125.50	18.69	84.00	170.00	115.96	128.60
2	38	118.21	118.50	18.31	86.00	180.00	112.19	124.23
3	38	117.95	117.00	19.02	90.00	165.00	111.70	124.20
4	41	117.10	114.00	16.84	78.00	156.00	111.78	122.41
5	36	118.92	114.50	16.53	86.00	160.00	113.32	124.51
6	34	117.41	112.00	20.76	80.00	165.00	110.17	124.66
7	30	114.47	114.00	15.67	80.00	155.00	108.61	120.32
8	21	120.24	120.00	19.73	74.00	163.00	111.26	129.22
9	17	121.65	120.00	15.08	105.00	159.00	113.89	129.40
10	12	123.25	125.00	13.51	105.00	150.00	114.67	131.83
11	10	120.10	121.00	16.03	90.00	150.00	108.63	131.57
12	5	130.60	128.00	15.58	112.00	147.00	111.25	149.95
13	6	123.00	124.00	12.63	108.00	140.00	109.74	136.26
14	6	127.33	120.00	14.64	114.00	147.00	111.97	142.69
15	2	129.00	129.00	1.41	128.00	130.00	116.29	141.71
16	3	140.33	134.00	14.57	130.00	157.00	104.14	176.53
17	2	131.50	131.50	16.26	120.00	143.00	-14.62	277.62
18	2	134.50	134.50	7.78	129.00	140.00	64.62	204.38
19	3	126.67	140.00	23.09	100.00	140.00	69.30	184.04
20	2	131.00	131.00	1.41	130.00	132.00	118.29	143.71
21	2	137.50	137.50	2.12	136.00	139.00	118.44	156.56
22	2	138.00	138.00	7.07	133.00	143.00	74.47	201.53
23	2	140.00	140.00	0.00	140.00	140.00	140.00	140.00
24	2	138.50	138.50	4.95	135.00	142.00	94.03	182.97
25	1	133.00	133.00		133.00	133.00		
26	1	140.00	140.00		140.00	140.00		
27	1	150.00	150.00		150.00	150.00		
28	1	144.00	144.00		144.00	144.00		
29	1	140.00	140.00		140.00	140.00		

EOP1 = End of Stage 1

Program: T0457.TEM, Version: 8.2, Datetime: 07FEB07:09:48

Table 14.3.5.4: Systolic blood pressure - summary statistics for Stage 2 (post-Subgam), mmHg

Visit number	n	Mean	Median	SD	Min	Max	Lower 95% CI	Upper 95% CI
EOP2	47	122.77	120.00	18.01	91.00	170.00	117.48	128.05

EOP1 = End of Stage 1

Program: T0457.TEM, Version: 8.2, Datetime: 07FEB07:09:48

Table 14.3.5.5: Systolic blood pressure - summary statistics for pharmacokinetic assessments, mmHg

Visit	Days post-infusion	n	Mean	Median	SD	Min	Max	Lower 95% CI	Upper 95% CI
1st Subgam infusion	Pre-infusion	4	130.75	121.00	25.76	113.00	168.00	89.76	171.74
	1	2	167.50	167.50	28.99	147.00	188.00	-92.98	427.98
	2	2	146.50	146.50	33.23	123.00	170.00	-152.10	445.10
	3	1	153.00	153.00		153.00	153.00		
	5	1	130.00	130.00		130.00	130.00		
	6	2	148.00	148.00	72.12	97.00	199.00	-500.02	796.02
	7	1	116.00	116.00		116.00	116.00		
After approximately 3 months on Subgam	Pre-infusion	1	125.00	125.00		125.00	125.00		
	2	1	170.00	170.00		170.00	170.00		
	3	1	150.00	150.00		150.00	150.00		
	5	1	99.00	99.00		99.00	99.00		
	6	2	140.50	140.50	55.86	101.00	180.00	-361.40	642.40
	7	1	100.00	100.00		100.00	100.00		
	8	1	95.00	95.00		95.00	95.00		
	9	1	104.00	104.00		104.00	104.00		

Table 14.3.5.6: Diastolic blood pressure - summary statistics for Stage 1 - all infusions, mmHg

Infusion	Observation	n	Mean	Median	SD	Min	Max	Lower 95% CI	Upper 95% CI
PRE		49	70.92	68.00	14.10	40.00	106.00	66.87	74.97
1	Pre-infusion	48	71.46	68.50	14.53	40.00	106.00	67.24	75.68
	1	47	70.51	68.00	14.73	40.00	128.00	66.19	74.84
	2	46	70.43	70.00	14.28	46.00	127.00	66.19	74.68
	3	37	72.70	71.00	16.48	40.00	127.00	67.21	78.20
	4	20	71.90	70.00	13.18	49.00	101.00	65.73	78.07
	5	6	69.67	73.00	9.05	55.00	80.00	60.17	79.16
	6	4	71.75	72.50	11.90	60.00	82.00	52.82	90.68
	7	2	68.50	68.50	19.09	55.00	82.00	-103.03	240.03
	8	1	62.00	62.00		62.00	62.00		
2	Pre-infusion	45	71.69	70.00	11.91	41.00	96.00	68.11	75.27
	1	44	70.93	66.50	12.01	51.00	99.00	67.28	74.58
	2	44	70.05	67.50	13.07	36.00	97.00	66.07	74.02
	3	36	70.72	68.00	14.53	35.00	113.00	65.81	75.64
	4	19	71.95	73.00	11.55	52.00	97.00	66.38	77.52
	5	8	65.38	68.00	7.69	50.00	73.00	58.95	71.80
	6	3	64.00	70.00	12.17	50.00	72.00	33.78	94.22
	7	1	59.00	59.00		59.00	59.00		
3	Pre-infusion	47	68.66	68.00	12.89	37.00	91.00	64.87	72.45
	1	46	69.39	69.00	12.18	38.00	90.00	65.78	73.01
	2	45	69.42	70.00	11.52	48.00	95.00	65.96	72.88
	3	35	72.26	75.00	13.60	41.00	102.00	67.58	76.93
	4	16	69.38	69.00	14.46	44.00	96.00	61.67	77.08
	5	7	76.71	71.00	12.23	63.00	93.00	65.40	88.03
	6	3	64.67	59.00	13.43	55.00	80.00	31.31	98.03

PRE = Pre-study Visit
EOP1 = End of Stage 1

Program: T0459.TEM, Version: 8.2, Datetime: 07FEB07:09:48

Table 14.3.5.6: Diastolic blood pressure - summary statistics for Stage 1 - all infusions, mmHg

Infusion	Observation	n	Mean	Median	SD	Min	Max	Lower 95% CI	Upper 95% CI
3	7	3	70.67	65.00	15.31	59.00	88.00	32.64	108.69

PRE = Pre-study visit
EOP1 = End of Stage 1

Program: T0459.TEM, Version: 8.2, Datetime: 07FEB07:09:48

Table 14.3.5.6: Diastolic blood pressure - summary statistics for Stage 1 - all infusions, mmHg

Infusion	Observation	n	Mean	Median	SD	Min	Max	Lower 95% CI	Upper 95% CI
3A	Pre-infusion	2	89.00	89.00	4.24	86.00	92.00	50.88	127.12
	1	2	81.50	81.50	10.61	74.00	89.00	-13.80	176.80
	2	2	92.50	92.50	4.95	89.00	96.00	48.03	136.97
	3	2	74.00	74.00	2.83	72.00	76.00	48.59	99.41
3B	Pre-infusion	2	78.50	78.50	4.95	75.00	82.00	34.03	122.97
	1	2	78.50	78.50	4.95	75.00	82.00	34.03	122.97
	2	2	69.00	69.00	1.41	68.00	70.00	56.29	81.71
	3	2	83.00	83.00	1.41	82.00	84.00	70.29	95.71
	4	2	83.00	83.00	12.73	74.00	92.00	-31.36	197.36
	5	1	79.00	79.00		79.00	79.00		
4	Pre-infusion	50	71.60	70.00	12.86	53.00	122.00	67.95	75.25
	1	50	71.58	70.00	12.93	53.00	122.00	67.90	75.26
	2	49	71.37	70.00	10.28	57.00	95.00	68.41	74.32
	3	43	71.70	69.00	11.10	53.00	92.00	68.28	75.12
	4	29	71.24	70.00	10.33	51.00	90.00	67.31	75.17
	5	8	71.38	69.50	9.36	59.00	89.00	63.55	79.20
	6	2	72.50	72.50	10.61	65.00	80.00	-22.80	167.80
5	Pre-infusion	48	70.38	68.50	11.98	45.00	100.00	66.90	73.85
	1	45	69.02	68.00	11.48	48.00	93.00	65.57	72.47
	2	39	70.62	70.00	11.65	44.00	91.00	66.84	74.39
	3	17	69.76	69.00	14.02	44.00	100.00	62.56	76.97
	4	3	76.67	72.00	21.39	58.00	100.00	23.54	129.79
	5	1	85.00	85.00		85.00	85.00		

PRE = Pre-study Visit
EOP1 = End of Stage 1

Program: T0459.TEM, Version: 8.2, Datetime: 07FEB07:09:48

Table 14.3.5.6: Diastolic blood pressure - summary statistics for Stage 1 - all infusions, mmHg

Infusion	Observation	n	Mean	Median	SD	Min	Max	Lower 95% CI	Upper 95% CI
6	Pre-infusion	46	70.80	68.50	12.79	45.00	96.00	67.01	74.60
	1	43	69.23	65.00	12.59	44.00	95.00	65.36	73.11
	2	31	71.74	70.00	13.18	48.00	94.00	66.91	76.58
	3	13	72.54	70.00	14.67	51.00	100.00	63.68	81.40
	4	1	90.00	90.00		90.00	90.00		
7	Pre-infusion	40	72.48	70.00	12.76	50.00	96.00	68.39	76.56
	1	40	69.25	68.50	12.97	50.00	100.00	65.10	73.40
	2	32	72.16	70.50	12.74	54.00	98.00	67.56	76.75
	3	10	67.00	63.50	10.99	55.00	88.00	59.13	74.87
	4	2	70.00	70.00	14.14	60.00	80.00	-57.06	197.06
8	Pre-infusion	37	70.14	69.00	12.25	47.00	103.00	66.05	74.22
	1	36	70.11	70.00	11.63	52.00	93.00	66.18	74.05
	2	25	74.76	71.00	11.25	54.00	90.00	70.12	79.40
	3	7	68.71	71.00	13.01	55.00	90.00	56.68	80.75
	4	2	81.00	81.00	5.66	77.00	85.00	30.18	131.82
9	Pre-infusion	27	70.33	68.00	13.31	48.00	96.00	65.07	75.60
	1	25	69.24	69.00	11.26	40.00	94.00	64.59	73.89
	2	23	72.52	71.00	13.81	50.00	92.00	66.55	78.49
	3	7	64.57	60.00	16.05	48.00	93.00	49.73	79.42
	4	2	87.50	87.50	3.54	85.00	90.00	55.73	119.27
10	Pre-infusion	26	71.81	70.00	11.03	56.00	92.00	67.35	76.26
	1	24	73.13	70.50	11.89	54.00	96.00	68.10	78.15
	2	19	74.37	76.00	11.45	59.00	95.00	68.85	79.89
	3	5	70.00	69.00	7.97	61.00	80.00	60.11	79.89

PRE = Pre-study Visit
EOP1 = End of Stage 1

Program: T0459.TEM, Version: 8.2, Datetime: 07FEB07:09:48

Table 14.3.5.6: Diastolic blood pressure - summary statistics for Stage 1 - all infusions, mmHg

Infusion	Observation	n	Mean	Median	SD	Min	Max	Lower 95% CI	Upper 95% CI
10	4	1	75.00	75.00		75.00	75.00		

PRE = Pre-study Visit
EOP1 = End of Stage 1

Program: T0459.TEM, Version: 8.2, Datetime: 07FEB07:09:48

Table 14.3.5.6: Diastolic blood pressure - summary statistics for Stage 1 - all infusions, mmHg

Infusion	Observation	n	Mean	Median	SD	Min	Max	Lower 95% CI	Upper 95% CI
11	Pre-infusion	24	70.33	72.00	13.63	47.00	89.00	64.58	76.09
	1	21	72.57	72.00	10.23	57.00	90.00	67.91	77.23
	2	20	73.00	74.00	10.26	56.00	93.00	68.20	77.80
	3	6	74.83	78.00	8.26	60.00	82.00	66.17	83.50
	4	1	80.00	80.00		80.00	80.00		
12	Pre-infusion	19	65.68	64.00	12.96	47.00	94.00	59.44	71.93
	1	18	68.11	68.50	11.39	52.00	86.00	62.45	73.77
	2	13	74.08	76.00	10.13	60.00	92.00	67.96	80.20
	3	3	84.00	83.00	4.58	80.00	89.00	72.62	95.38
	4	1	80.00	80.00		80.00	80.00		
13	Pre-infusion	9	69.22	70.00	13.20	54.00	90.00	59.08	79.37
	1	9	70.22	72.00	10.07	58.00	87.00	62.48	77.96
	2	7	71.29	75.00	9.27	55.00	81.00	62.71	79.86
	3	3	81.33	83.00	5.69	75.00	86.00	67.21	95.46
	4	1	80.00	80.00		80.00	80.00		
14	Pre-infusion	8	71.88	70.00	4.97	65.00	80.00	67.72	76.03
	1	8	72.75	78.00	10.21	53.00	81.00	64.22	81.28
	2	6	71.00	72.00	14.48	52.00	93.00	55.81	86.19
	3	4	78.25	82.50	11.15	62.00	86.00	60.51	95.99
	4	1	80.00	80.00		80.00	80.00		
15	Pre-infusion	11	69.91	70.00	13.70	49.00	87.00	60.71	79.11
	1	10	68.10	69.00	12.92	50.00	92.00	58.86	77.34
	2	8	68.38	62.50	12.27	56.00	87.00	58.12	78.63
	3	2	86.50	86.50	2.12	85.00	88.00	67.44	105.56

PRE = Pre-study Visit
EOP1 = End of Stage 1

Program: T0459.TEM, Version: 8.2, Datetime: 07FEB07:09:48

Table 14.3.5.6: Diastolic blood pressure - summary statistics for Stage 1 - all infusions, mmHg

Infusion	Observation	n	Mean	Median	SD	Min	Max	Lower 95% CI	Upper 95% CI
15	4	1	80.00	80.00		80.00	80.00		

PRE = Pre-study Visit
EOP1 = End of Stage 1

Program: T0459.TEM, Version: 8.2, Datetime: 07FEB07:09:48

Table 14.3.5.6: Diastolic blood pressure - summary statistics for Stage 1 - all infusions, mmHg

Infusion	Observation	n	Mean	Median	SD	Min	Max	Lower 95% CI	Upper 95% CI
16	Pre-infusion	16	66.88	68.50	11.81	47.00	83.00	60.58	73.17
	1	14	66.79	67.50	11.24	53.00	80.00	60.30	73.28
	2	6	74.33	73.50	6.35	68.00	86.00	67.67	80.99
	3	2	89.00	89.00	12.73	80.00	98.00	-25.36	203.36
	4	1	70.00	70.00		70.00	70.00		
17	Pre-infusion	5	81.40	85.00	11.95	66.00	94.00	66.56	96.24
	1	4	75.75	75.50	6.13	70.00	82.00	65.99	85.51
	2	2	76.00	76.00	1.41	75.00	77.00	63.29	88.71
	3	2	75.50	75.50	7.78	70.00	81.00	5.62	145.38
	4	1	70.00	70.00		70.00	70.00		
18	Pre-infusion	4	73.25	72.00	7.27	66.00	83.00	61.67	84.83
	1	4	74.75	74.00	7.72	67.00	84.00	62.47	87.03
	2	2	66.00	66.00	8.49	60.00	72.00	-10.24	142.24
	3	2	73.50	73.50	4.95	70.00	77.00	29.03	117.97
	4	1	70.00	70.00		70.00	70.00		
19	Pre-infusion	6	63.67	63.50	15.65	40.00	81.00	47.24	80.10
	1	7	79.43	80.00	19.30	58.00	112.00	61.58	97.28
	2	4	69.25	68.50	20.29	48.00	92.00	36.97	101.53
	3	3	62.67	68.00	11.02	50.00	70.00	35.30	90.03
	4	1	70.00	70.00		70.00	70.00		
20	Pre-infusion	12	68.00	69.00	9.94	53.00	81.00	61.69	74.31
	1	10	63.50	61.50	11.55	49.00	83.00	55.24	71.76
	2	3	70.33	74.00	9.07	60.00	77.00	47.79	92.87
	3	1	90.00	90.00		90.00	90.00		

PRE = Pre-study Visit
EOP1 = End of Stage 1

Program: T0459.TEM, Version: 8.2, Datetime: 07FEB07:09:48

Table 14.3.5.6: Diastolic blood pressure - summary statistics for Stage 1 - all infusions, mmHg

Infusion	Observation	n	Mean	Median	SD	Min	Max	Lower 95% CI	Upper 95% CI
21	Pre-infusion	4	71.75	72.50	7.93	62.00	80.00	59.13	84.37
	1	4	77.75	80.00	11.79	62.00	89.00	59.00	96.50
	2	2	81.50	81.50	9.19	75.00	88.00	-1.09	164.09
	3	1	77.00	77.00		77.00	77.00		
22	Pre-infusion	4	75.50	80.50	11.03	59.00	82.00	57.95	93.05
	1	3	74.33	79.00	15.53	57.00	87.00	35.74	112.92
	2	2	72.00	72.00	15.56	61.00	83.00	-67.77	211.77
	3	2	70.00	70.00	9.90	63.00	77.00	-18.94	158.94
23	Pre-infusion	5	69.60	66.00	9.56	61.00	83.00	57.74	81.46
	1	4	77.00	78.50	7.16	67.00	84.00	65.60	88.40
	2	2	76.50	76.50	2.12	75.00	78.00	57.44	95.56
	3	2	77.00	77.00	1.41	76.00	78.00	64.29	89.71
24	Pre-infusion	9	67.00	62.00	12.48	53.00	89.00	57.41	76.59
	1	8	65.25	66.50	12.75	47.00	87.00	54.59	75.91
	2	3	74.00	76.00	6.24	67.00	79.00	58.49	89.51
	3	1	79.00	79.00		79.00	79.00		
25	Pre-infusion	4	68.00	66.50	8.45	60.00	79.00	54.56	81.44
	1	4	72.75	71.50	3.59	70.00	78.00	67.03	78.47
	2	2	68.50	68.50	12.02	60.00	77.00	-39.50	176.50
	3	1	80.00	80.00		80.00	80.00		
26	Pre-infusion	4	68.50	71.00	11.90	52.00	80.00	49.56	87.44
	1	4	72.00	73.50	12.36	57.00	84.00	52.34	91.66
	2	3	70.67	70.00	9.02	62.00	80.00	48.26	93.07

PRE = Pre-study Visit
EOP1 = End of Stage 1

Program: T0459.TEM, Version: 8.2, Datetime: 07FEB07:09:48

Table 14.3.5.6: Diastolic blood pressure - summary statistics for Stage 1 - all infusions, mmHg

Infusion	Observation	n	Mean	Median	SD	Min	Max	Lower 95% CI	Upper 95% CI
26	3	1	80.00	80.00		80.00	80.00		

PRE = Pre-study Visit
EOP1 = End of Stage 1

Program: T0459.TEM, Version: 8.2, Datetime: 07FEB07:09:48

Table 14.3.5.6: Diastolic blood pressure - summary statistics for Stage 1 - all infusions, mmHg

Infusion	Observation	n	Mean	Median	SD	Min	Max	Lower 95% CI	Upper 95% CI
27	Pre-infusion	5	78.80	84.00	10.13	61.00	85.00	66.22	91.38
	1	4	74.25	76.00	11.21	59.00	86.00	56.42	92.08
	2	3	66.67	77.00	20.55	43.00	80.00	15.62	117.72
	3	2	77.50	77.50	3.54	75.00	80.00	45.73	109.27
28	Pre-infusion	11	64.09	60.00	16.99	49.00	111.00	52.68	75.51
	1	10	66.50	67.50	12.01	51.00	89.00	57.91	75.09
	2	2	75.00	75.00	7.07	70.00	80.00	11.47	138.53
	3	2	71.00	71.00	1.41	70.00	72.00	58.29	83.71
29	Pre-infusion	4	74.00	71.00	11.17	65.00	89.00	56.23	91.77
	1	4	68.00	67.50	4.97	63.00	74.00	60.10	75.90
	2	2	78.00	78.00	7.07	73.00	83.00	14.47	141.53
	3	1	74.00	74.00		74.00	74.00		
30	Pre-infusion	3	68.00	69.00	12.53	55.00	80.00	36.87	99.13
	1	4	70.75	71.50	5.91	63.00	77.00	61.35	80.15
	2	3	75.67	74.00	3.79	73.00	80.00	66.26	85.07
	3	1	70.00	70.00		70.00	70.00		
EOP1		50	71.06	70.00	13.66	40.00	100.00	67.18	74.94

PRE = Pre-study Visit
EOP1 = End of Stage 1

Program: T0459.TEM, Version: 8.2, Datetime: 07FEB07:09:48

Table 14.3.5.7: Diastolic blood pressure - summary statistics for Stage 1 - Infusions 1-3 (pre-Subgam), mmHg

Observation	n	Mean	Median	SD	Min	Max	Lower 95% CI	Upper 95% CI
Pre-infusion	144	70.96	69.50	13.20	37.00	106.00	68.78	73.13
1	141	70.55	68.00	12.94	38.00	128.00	68.39	72.70
2	139	70.28	69.00	13.03	36.00	127.00	68.10	72.47
3	112	72.13	71.00	14.64	35.00	127.00	69.39	74.88
4	57	71.60	70.00	12.92	44.00	101.00	68.17	75.02
5	22	70.77	70.50	10.41	50.00	93.00	66.16	75.39
6	10	67.30	66.50	11.61	50.00	82.00	58.99	75.61
7	6	68.00	62.00	13.68	55.00	88.00	53.64	82.36
8	1	62.00	62.00		62.00	62.00		

Table 14.3.5.8: Diastolic blood pressure - summary statistics for Stage 1 - Infusions 4-30 (post-Subgam), mmHg

Observation	n	Mean	Median	SD	Min	Max	Lower 95% CI	Upper 95% CI
Pre-infusion	441	70.37	70.00	12.37	40.00	122.00	69.21	71.52
1	417	70.20	70.00	11.87	40.00	122.00	69.06	71.35
2	313	72.24	71.00	11.39	43.00	98.00	70.97	73.51
3	144	72.23	71.00	11.79	44.00	100.00	70.29	74.17
4	49	73.84	73.00	10.59	51.00	100.00	70.79	76.88
5	9	72.89	70.00	9.87	59.00	89.00	65.30	80.47
6	2	72.50	72.50	10.61	65.00	80.00	-22.80	167.80

Table 14.3.5.9: Diastolic blood pressure - summary statistics for Stage 2 (post-Subgam), mmHg

Visit number	n	Mean	Median	SD	Min	Max	Lower 95% CI	Upper 95% CI
1	36	72.64	73.50	11.81	46.00	92.00	68.64	76.64
2	38	70.71	72.00	12.19	50.00	100.00	66.70	74.72
3	38	70.95	67.50	14.86	44.00	98.00	66.06	75.83
4	41	71.71	70.00	12.95	52.00	101.00	67.62	75.79
5	36	71.47	70.50	13.40	46.00	95.00	66.94	76.01
6	34	71.94	70.00	11.80	53.00	99.00	67.83	76.06
7	30	72.27	72.00	12.45	52.00	98.00	67.62	76.91
8	21	73.90	72.00	12.36	42.00	95.00	68.28	79.53
9	17	76.35	75.00	11.44	65.00	112.00	70.47	82.23
10	12	80.75	79.00	8.74	70.00	100.00	75.20	86.30
11	10	78.30	80.00	12.99	50.00	95.00	69.01	87.59
12	5	79.80	75.00	12.19	68.00	100.00	64.66	94.94
13	6	82.00	80.00	6.93	72.00	90.00	74.73	89.27
14	6	80.00	78.50	16.78	59.00	107.00	62.39	97.61
15	2	73.00	73.00	4.24	70.00	76.00	34.88	111.12
16	3	88.00	86.00	3.46	86.00	92.00	79.39	96.61
17	2	87.50	87.50	10.61	80.00	95.00	-7.80	182.80
18	2	85.50	85.50	13.44	76.00	95.00	-35.21	206.21
19	3	83.67	90.00	21.22	60.00	101.00	30.95	136.38
20	2	88.00	88.00	2.83	86.00	90.00	62.59	113.41
21	2	86.50	86.50	4.95	83.00	90.00	42.03	130.97
22	2	74.50	74.50	3.54	72.00	77.00	42.73	106.27
23	2	90.00	90.00	0.00	90.00	90.00	90.00	90.00
24	2	74.50	74.50	14.85	64.00	85.00	-58.92	207.92
25	1	76.00	76.00		76.00	76.00		
26	1	80.00	80.00		80.00	80.00		
27	1	100.00	100.00		100.00	100.00		
28	1	74.00	74.00		74.00	74.00		
29	1	70.00	70.00		70.00	70.00		

EOP1 = End of Stage 1

Program: T0462.TEM, Version: 8.2, Datetime: 07FEB07:09:48

Table 14.3.5.9: Diastolic blood pressure - summary statistics for Stage 2 (post-Subgam), mmHg

Visit number	n	Mean	Median	SD	Min	Max	Lower 95% CI	Upper 95% CI
EOP2	47	72.94	72.00	16.42	37.00	130.00	68.12	77.76

EOP1 = End of Stage 1

Program: T0462.TEM, Version: 8.2, Datetime: 07FEB07:09:48

Table 14.3.5.10: Diastolic blood pressure - summary statistics for pharmacokinetic assessments, mmHg

Visit	Days post-infusion	n	Mean	Median	SD	Min	Max	Lower 95% CI	Upper 95% CI
1st Subgam infusion	Pre-infusion	4	82.00	70.50	26.94	65.00	122.00	39.13	124.87
	1	2	76.50	76.50	0.71	76.00	77.00	70.15	82.85
	2	2	92.00	92.00	48.08	58.00	126.00	-340.01	524.01
	3	1	77.00	77.00		77.00	77.00		
	5	1	75.00	75.00		75.00	75.00		
	6	2	71.50	71.50	14.85	61.00	82.00	-61.92	204.92
	7	1	68.00	68.00		68.00	68.00		
After approximately 3 months on Subgam	Pre-infusion	1	69.00	69.00		69.00	69.00		
	2	1	80.00	80.00		80.00	80.00		
	3	1	80.00	80.00		80.00	80.00		
	5	1	56.00	56.00		56.00	56.00		
	6	2	74.00	74.00	22.63	58.00	90.00	-129.30	277.30
	7	1	53.00	53.00		53.00	53.00		
	8	1	57.00	57.00		57.00	57.00		
	9	1	57.00	57.00		57.00	57.00		

Table 14.3.5.11: Pulse - summary statistics for Stage 1 - all infusions, beats per minute

Infusion	Observation	n	Mean	Median	SD	Min	Max	Lower 95% CI	Upper 95% CI
PRE		49	80.59	78.00	14.27	58.00	129.00	76.49	84.69
1	Pre-infusion	48	80.31	78.00	13.62	57.00	129.00	76.36	84.27
	1	48	80.75	76.00	14.14	60.00	120.00	76.64	84.86
	2	46	81.00	78.00	15.64	53.00	123.00	76.35	85.65
	3	38	80.11	76.50	15.47	59.00	121.00	75.02	85.19
	4	19	78.68	80.00	10.18	60.00	92.00	73.78	83.59
	5	6	82.50	82.00	5.24	77.00	91.00	77.00	88.00
	6	4	76.25	79.50	9.95	62.00	84.00	60.42	92.08
	7	2	69.00	69.00	9.90	62.00	76.00	-19.94	157.94
	8	1	88.00	88.00		88.00	88.00		
2	Pre-infusion	45	82.51	80.00	13.10	51.00	125.00	78.58	86.45
	1	45	81.87	80.00	13.06	51.00	118.00	77.94	85.79
	2	45	78.42	76.00	14.22	52.00	112.00	74.15	82.69
	3	37	79.81	80.00	13.03	56.00	116.00	75.47	84.16
	4	18	81.06	80.50	10.64	56.00	96.00	75.77	86.34
	5	8	83.38	84.00	9.49	71.00	96.00	75.44	91.31
	6	3	77.33	70.00	12.70	70.00	92.00	45.78	108.89
	7	1	89.00	89.00		89.00	89.00		
3	Pre-infusion	47	82.60	81.00	15.10	56.00	125.00	78.16	87.03
	1	46	80.98	79.50	15.89	56.00	126.00	76.26	85.70
	2	46	81.41	77.00	16.43	56.00	128.00	76.53	86.29
	3	36	80.36	79.00	15.44	56.00	117.00	75.14	85.59
	4	16	81.94	79.00	20.11	60.00	145.00	71.22	92.65
	5	7	77.86	76.00	11.31	65.00	98.00	67.40	88.31
	6	3	76.33	74.00	4.93	73.00	82.00	64.08	88.59

PRE = Pre-study Visit
EOP1 = End of Stage 1

Program: T0464.TEM, Version: 8.2, Datetime: 07FEB07:09:48

Table 14.3.5.11: Pulse - summary statistics for Stage 1 - all infusions, beats per minute

Infusion	Observation	n	Mean	Median	SD	Min	Max	Lower 95% CI	Upper 95% CI
3	7	3	81.67	78.00	10.02	74.00	93.00	56.78	106.55

PRE = Pre-study Visit
EOP1 = End of Stage 1

Program: T0464.TEM, Version: 8.2, Datetime: 07FEB07:09:48

Table 14.3.5.11: Pulse - summary statistics for Stage 1 - all infusions, beats per minute

Infusion	Observation	n	Mean	Median	SD	Min	Max	Lower 95% CI	Upper 95% CI
3A	Pre-infusion	2	76.50	76.50	10.61	69.00	84.00	-18.80	171.80
	1	2	71.00	71.00	1.41	70.00	72.00	58.29	83.71
	2	2	80.00	80.00	0.00	80.00	80.00	80.00	80.00
	3	2	85.00	85.00	1.41	84.00	86.00	72.29	97.71
3B	Pre-infusion	2	78.50	78.50	4.95	75.00	82.00	34.03	122.97
	1	2	78.50	78.50	4.95	75.00	82.00	34.03	122.97
	2	2	73.50	73.50	3.54	71.00	76.00	41.73	105.27
	3	2	69.00	69.00	1.41	68.00	70.00	56.29	81.71
	4	2	82.00	82.00	0.00	82.00	82.00	82.00	82.00
	5	1	74.00	74.00		74.00	74.00		
4	Pre-infusion	49	82.76	81.00	15.16	60.00	137.00	78.40	87.11
	1	49	82.53	81.00	15.36	60.00	137.00	78.12	86.94
	2	49	82.82	78.00	18.23	54.00	140.00	77.58	88.05
	3	43	78.56	76.00	13.06	61.00	118.00	74.54	82.58
	4	29	75.28	72.00	11.54	59.00	106.00	70.89	79.67
	5	8	78.88	78.00	17.71	54.00	114.00	64.07	93.68
	6	2	64.50	64.50	4.95	61.00	68.00	20.03	108.97
5	Pre-infusion	48	82.85	80.00	13.88	58.00	126.00	78.82	86.88
	1	46	83.83	80.00	17.41	58.00	148.00	78.66	89.00
	2	39	77.62	76.00	11.23	57.00	113.00	73.97	81.26
	3	17	74.35	74.00	10.91	54.00	91.00	68.74	79.96
	4	3	73.00	72.00	6.56	67.00	80.00	56.71	89.29
6	Pre-infusion	46	83.07	80.00	15.05	59.00	137.00	78.60	87.53
	1	44	82.23	79.50	16.40	54.00	126.00	77.24	87.21

PRE = Pre-study Visit
EOP1 = End of Stage 1

Program: T0464.TEM, Version: 8.2, Datetime: 07FEB07:09:48

Table 14.3.5.11: Pulse - summary statistics for Stage 1 - all infusions, beats per minute

Infusion	Observation	n	Mean	Median	SD	Min	Max	Lower 95% CI	Upper 95% CI
6	2	32	80.31	80.00	11.37	58.00	116.00	76.21	84.41
	3	13	73.15	71.00	6.97	62.00	85.00	68.94	77.37
	4	1	68.00	68.00		68.00	68.00		

PRE = Pre-study Visit
EOP1 = End of Stage 1

Program: T0464.TEM, Version: 8.2, Datetime: 07FEB07:09:48

Table 14.3.5.11: Pulse - summary statistics for Stage 1 - all infusions, beats per minute

Infusion	Observation	n	Mean	Median	SD	Min	Max	Lower 95% CI	Upper 95% CI
7	Pre-infusion	41	81.12	80.00	15.80	38.00	115.00	76.14	86.11
	1	40	80.73	80.00	14.64	56.00	129.00	76.04	85.41
	2	33	76.79	75.00	12.58	54.00	111.00	72.33	81.25
	3	10	76.10	73.50	15.37	60.00	114.00	65.10	87.10
	4	2	80.00	80.00	0.00	80.00	80.00	80.00	80.00
8	Pre-infusion	37	84.14	83.00	17.66	48.00	137.00	78.25	90.02
	1	36	83.08	78.50	17.00	60.00	137.00	77.33	88.83
	2	26	79.27	74.00	14.31	62.00	118.00	73.49	85.05
	3	7	74.57	71.00	11.07	64.00	92.00	64.33	84.81
	4	2	68.00	68.00	11.31	60.00	76.00	-33.65	169.65
9	Pre-infusion	27	81.19	79.00	12.55	64.00	106.00	76.22	86.15
	1	26	77.85	75.00	10.99	60.00	110.00	73.41	82.29
	2	24	77.83	73.50	12.80	60.00	110.00	72.43	83.24
	3	7	78.86	78.00	8.93	67.00	95.00	70.59	87.12
	4	2	78.00	78.00	2.83	76.00	80.00	52.59	103.41
10	Pre-infusion	26	85.31	83.50	14.12	64.00	122.00	79.60	91.01
	1	24	75.46	74.00	9.08	58.00	92.00	71.62	79.29
	2	19	76.95	75.00	7.52	68.00	91.00	73.32	80.57
	3	5	72.20	73.00	4.09	68.00	77.00	67.13	77.27
	4	1	76.00	76.00		76.00	76.00		
11	Pre-infusion	24	80.58	80.00	11.79	60.00	115.00	75.61	85.56
	1	22	81.23	78.00	15.19	56.00	118.00	74.49	87.96
	2	20	76.90	76.00	13.31	59.00	106.00	70.67	83.13
	3	6	77.50	77.50	12.21	61.00	98.00	64.69	90.31

PRE = Pre-study Visit
EOP1 = End of Stage 1

Program: T0464.TEM, Version: 8.2, Datetime: 07FEB07:09:48

Table 14.3.5.11: Pulse - summary statistics for Stage 1 - all infusions, beats per minute

Infusion	Observation	n	Mean	Median	SD	Min	Max	Lower 95% CI	Upper 95% CI
11	4	1	76.00	76.00		76.00	76.00		

PRE = Pre-study Visit
EOP1 = End of Stage 1

Program: T0464.TEM, Version: 8.2, Datetime: 07FEB07:09:48

Table 14.3.5.11: Pulse - summary statistics for Stage 1 - all infusions, beats per minute

Infusion	Observation	n	Mean	Median	SD	Min	Max	Lower 95% CI	Upper 95% CI
12	Pre-infusion	19	83.63	86.00	16.29	58.00	131.00	75.78	91.48
	1	19	85.42	81.00	17.25	64.00	125.00	77.10	93.74
	2	14	80.64	80.00	10.58	61.00	101.00	74.53	86.75
	3	3	70.00	68.00	12.12	59.00	83.00	39.88	100.12
	4	1	72.00	72.00		72.00	72.00		
13	Pre-infusion	9	80.00	82.00	14.15	57.00	99.00	69.12	90.88
	1	9	80.11	81.00	12.72	60.00	103.00	70.33	89.89
	2	7	79.57	83.00	11.90	55.00	91.00	68.57	90.58
	3	3	73.67	72.00	15.57	59.00	90.00	35.00	112.34
	4	1	72.00	72.00		72.00	72.00		
14	Pre-infusion	8	78.25	76.00	7.48	68.00	93.00	72.00	84.50
	1	8	75.75	75.50	11.66	60.00	93.00	66.00	85.50
	2	6	73.17	74.00	7.28	60.00	80.00	65.53	80.80
	3	4	73.75	72.00	4.19	71.00	80.00	67.08	80.42
	4	1	72.00	72.00		72.00	72.00		
15	Pre-infusion	11	80.27	81.00	8.83	68.00	98.00	74.34	86.21
	1	10	78.50	79.00	8.50	61.00	93.00	72.42	84.58
	2	8	74.50	79.50	10.09	56.00	86.00	66.07	82.93
	3	2	66.50	66.50	7.78	61.00	72.00	-3.38	136.38
	4	1	72.00	72.00		72.00	72.00		
16	Pre-infusion	16	90.38	93.00	14.78	66.00	125.00	82.50	98.25
	1	15	90.40	87.00	20.39	64.00	137.00	79.11	101.69
	2	6	76.00	69.50	19.83	64.00	116.00	55.19	96.81
	3	2	68.00	68.00	0.00	68.00	68.00	68.00	68.00

PRE = Pre-study Visit
EOP1 = End of Stage 1

Program: T0464.TEM, Version: 8.2, Datetime: 07FEB07:09:48

Table 14.3.5.11: Pulse - summary statistics for Stage 1 - all infusions, beats per minute

Infusion	Observation	n	Mean	Median	SD	Min	Max	Lower 95% CI	Upper 95% CI
16	4	1	68.00	68.00		68.00	68.00		

PRE = Pre-study Visit
EOP1 = End of Stage 1

Program: T0464.TEM, Version: 8.2, Datetime: 07FEB07:09:48

Table 14.3.5.11: Pulse - summary statistics for Stage 1 - all infusions, beats per minute

Infusion	Observation	n	Mean	Median	SD	Min	Max	Lower 95% CI	Upper 95% CI
17	Pre-infusion	5	77.20	74.00	9.63	68.00	91.00	65.25	89.15
	1	4	84.00	79.50	11.52	76.00	101.00	65.67	102.33
	2	2	63.00	63.00	12.73	54.00	72.00	-51.36	177.36
	3	2	78.50	78.50	14.85	68.00	89.00	-54.92	211.92
	4	1	72.00	72.00		72.00	72.00		
18	Pre-infusion	4	84.00	88.50	15.98	61.00	98.00	58.57	109.43
	1	4	78.75	74.00	19.24	61.00	106.00	48.13	109.37
	2	2	69.50	69.50	3.54	67.00	72.00	37.73	101.27
	3	2	73.00	73.00	1.41	72.00	74.00	60.29	85.71
	4	1	72.00	72.00		72.00	72.00		
19	Pre-infusion	6	75.83	76.50	7.36	63.00	85.00	68.11	83.56
	1	7	84.71	84.00	22.76	58.00	128.00	63.67	105.76
	2	4	78.75	74.00	14.64	67.00	100.00	55.46	102.04
	3	3	65.00	65.00	7.00	58.00	72.00	47.61	82.39
	4	1	76.00	76.00		76.00	76.00		
20	Pre-infusion	12	86.17	83.00	20.78	60.00	133.00	72.97	99.37
	1	10	95.70	104.00	27.33	60.00	145.00	76.15	115.25
	2	3	65.67	62.00	8.14	60.00	75.00	45.43	85.90
	3	1	59.00	59.00		59.00	59.00		
21	Pre-infusion	4	80.00	79.50	7.53	72.00	89.00	68.02	91.98
	1	4	80.00	77.00	9.45	73.00	93.00	64.96	95.04
	2	2	66.50	66.50	4.95	63.00	70.00	22.03	110.97
	3	1	63.00	63.00		63.00	63.00		

PRE = Pre-study Visit
EOP1 = End of Stage 1

Program: T0464.TEM, Version: 8.2, Datetime: 07FEB07:09:48

Table 14.3.5.11: Pulse - summary statistics for Stage 1 - all infusions, beats per minute

Infusion	Observation	n	Mean	Median	SD	Min	Max	Lower 95% CI	Upper 95% CI
22	Pre-infusion	4	89.50	79.50	23.81	74.00	125.00	51.61	127.39
	1	3	77.33	76.00	11.06	67.00	89.00	49.86	104.81
	2	2	83.00	83.00	11.31	75.00	91.00	-18.65	184.65
	3	2	80.50	80.50	6.36	76.00	85.00	23.32	137.68
23	Pre-infusion	5	91.40	85.00	20.44	70.00	114.00	66.02	116.78
	1	5	90.60	81.00	22.81	71.00	124.00	62.28	118.92
	2	2	75.50	75.50	4.95	72.00	79.00	31.03	119.97
	3	2	70.50	70.50	2.12	69.00	72.00	51.44	89.56
24	Pre-infusion	9	80.78	82.00	15.22	55.00	106.00	69.08	92.48
	1	8	85.25	85.50	21.96	60.00	125.00	66.89	103.61
	2	3	72.33	72.00	4.51	68.00	77.00	61.13	83.53
	3	1	57.00	57.00		57.00	57.00		
25	Pre-infusion	4	81.00	81.50	8.98	71.00	90.00	66.71	95.29
	1	4	83.75	81.50	22.75	60.00	112.00	47.55	119.95
	2	2	65.00	65.00	1.41	64.00	66.00	52.29	77.71
	3	1	76.00	76.00		76.00	76.00		
26	Pre-infusion	4	76.25	71.50	11.95	68.00	94.00	57.23	95.27
	1	4	78.00	77.50	8.68	68.00	89.00	64.19	91.81
	2	3	76.67	72.00	9.87	70.00	88.00	52.16	101.17
	3	1	68.00	68.00		68.00	68.00		
27	Pre-infusion	5	81.40	80.00	13.43	64.00	101.00	64.73	98.07
	1	4	80.00	76.50	20.74	59.00	108.00	47.00	113.00
	2	3	67.00	69.00	4.36	62.00	70.00	56.17	77.83

PRE = Pre-study Visit
EOP1 = End of Stage 1

Program: T0464.TEM, Version: 8.2, Datetime: 07FEB07:09:48

Table 14.3.5.11: Pulse - summary statistics for Stage 1 - all infusions, beats per minute

Infusion	Observation	n	Mean	Median	SD	Min	Max	Lower 95% CI	Upper 95% CI
27	3	2	69.50	69.50	6.36	65.00	74.00	12.32	126.68

PRE = Pre-study Visit
EOP1 = End of Stage 1

Program: T0464.TEM, Version: 8.2, Datetime: 07FEB07:09:48

Table 14.3.5.11: Pulse - summary statistics for Stage 1 - all infusions, beats per minute

Infusion	Observation	n	Mean	Median	SD	Min	Max	Lower 95% CI	Upper 95% CI
28	Pre-infusion	11	93.45	95.00	17.70	66.00	121.00	81.56	105.35
	1	10	90.80	88.50	19.42	66.00	123.00	76.91	104.69
	2	2	75.50	75.50	4.95	72.00	79.00	31.03	119.97
	3	2	75.00	75.00	9.90	68.00	82.00	-13.94	163.94
29	Pre-infusion	4	84.25	84.00	4.43	80.00	89.00	77.21	91.29
	1	4	78.25	82.00	13.25	60.00	89.00	57.17	99.33
	2	2	68.50	68.50	12.02	60.00	77.00	-39.50	176.50
	3	1	57.00	57.00		57.00	57.00		
30	Pre-infusion	4	76.50	72.50	8.35	72.00	89.00	63.22	89.78
	1	4	78.50	74.00	11.93	70.00	96.00	59.52	97.48
	2	3	82.33	75.00	15.37	72.00	100.00	44.14	120.52
	3	1	64.00	64.00		64.00	64.00		
EOP1		49	83.61	82.00	16.59	20.00	126.00	78.85	88.38

PRE = Pre-study Visit
EOP1 = End of Stage 1

Program: T0464.TEM, Version: 8.2, Datetime: 07FEB07:09:48

Table 14.3.5.12: Pulse - summary statistics for Stage 1 - Infusions 1-3 (pre-Subgam), beats per minute

Observation	n	Mean	Median	SD	Min	Max	Lower 95% CI	Upper 95% CI
Pre-infusion	144	81.67	80.00	13.77	51.00	129.00	79.40	83.94
1	143	81.01	79.00	14.18	51.00	126.00	78.66	83.35
2	141	80.19	78.00	15.21	52.00	128.00	77.66	82.72
3	115	79.98	78.00	14.39	56.00	121.00	77.32	82.64
4	55	80.53	80.00	13.58	56.00	145.00	76.86	84.20
5	22	80.95	78.50	9.03	65.00	98.00	76.95	84.96
6	10	76.60	75.50	8.63	62.00	92.00	70.43	82.77
7	6	78.67	77.00	11.13	62.00	93.00	66.99	90.35
8	1	88.00	88.00		88.00	88.00		

Table 14.3.5.13: Pulse - summary statistics for Stage 1 - Infusions 4-30 (post-Subgam), beats per minute

Observation	n	Mean	Median	SD	Min	Max	Lower 95% CI	Upper 95% CI
Pre-infusion	442	82.99	81.00	14.72	38.00	137.00	81.61	84.36
1	423	82.37	80.00	16.18	54.00	148.00	80.82	83.91
2	318	78.10	76.00	13.13	54.00	140.00	76.65	79.55
3	144	74.88	72.00	11.23	54.00	118.00	73.03	76.72
4	49	74.49	72.00	9.44	59.00	106.00	71.78	77.20
5	8	78.88	78.00	17.71	54.00	114.00	64.07	93.68
6	2	64.50	64.50	4.95	61.00	68.00	20.03	108.97

Table 14.3.5.14: Pulse - summary statistics for Stage 2 (post-Subgam), beats per minute

Visit number	n	Mean	Median	SD	Min	Max	Lower 95% CI	Upper 95% CI
1	36	83.19	79.00	15.46	61.00	133.00	77.96	88.43
2	38	80.61	80.50	11.42	60.00	110.00	76.85	84.36
3	38	80.76	79.00	13.64	58.00	117.00	76.28	85.25
4	40	80.45	80.00	14.26	48.00	112.00	75.89	85.01
5	36	80.42	77.00	14.94	60.00	117.00	75.36	85.47
6	33	80.33	80.00	10.40	60.00	98.00	76.65	84.02
7	29	78.24	80.00	11.82	48.00	111.00	73.74	82.74
8	21	80.48	80.00	11.04	52.00	100.00	75.45	85.50
9	14	75.36	75.50	9.04	60.00	90.00	70.14	80.57
10	11	77.36	78.00	12.80	48.00	97.00	68.76	85.96
11	9	82.33	82.00	8.38	70.00	91.00	75.89	88.78
12	5	79.20	75.00	9.73	70.00	92.00	67.12	91.28
13	6	76.83	77.00	2.99	72.00	81.00	73.69	79.98
14	6	79.50	78.00	9.38	70.00	97.00	69.66	89.34
15	3	74.67	76.00	7.09	67.00	81.00	57.04	92.29
16	3	86.33	84.00	7.77	80.00	95.00	67.04	105.63
17	2	88.50	88.50	12.02	80.00	97.00	-19.50	196.50
18	3	72.67	70.00	4.62	70.00	78.00	61.19	84.14
19	3	73.67	75.00	5.13	68.00	78.00	60.92	86.41
20	2	84.50	84.50	0.71	84.00	85.00	78.15	90.85
21	3	81.33	80.00	16.04	66.00	98.00	41.48	121.18
22	2	72.00	72.00	2.83	70.00	74.00	46.59	97.41
23	2	87.50	87.50	10.61	80.00	95.00	-7.80	182.80
24	2	85.50	85.50	0.71	85.00	86.00	79.15	91.85
25	1	70.00	70.00		70.00	70.00		
26	1	85.00	85.00		85.00	85.00		
28	1	64.00	64.00		64.00	64.00		
EOP2	47	80.00	80.00	14.24	50.00	126.00	75.82	84.18

EOP1 = End of Stage 1

Program: T0467.TEM, Version: 8.2, Datetime: 07FEB07:09:48

Table 14.3.5.15: Pulse - summary statistics for pharmacokinetic assessments, beats per minute

Visit	Days post-infusion	n	Mean	Median	SD	Min	Max	Lower 95% CI	Upper 95% CI
1st Subgam infusion	Pre-infusion	4	76.00	80.50	10.92	60.00	83.00	58.62	93.38
	1	2	76.00	76.00	12.73	67.00	85.00	-38.36	190.36
	2	2	77.00	77.00	19.80	63.00	91.00	-100.89	254.89
	3	1	63.00	63.00		63.00	63.00		
	5	1	85.00	85.00		85.00	85.00		
	6	2	72.50	72.50	14.85	62.00	83.00	-60.92	205.92
	7	1	89.00	89.00		89.00	89.00		
After approximately 3 months on Subgam	Pre-infusion	2	73.00	73.00	12.73	64.00	82.00	-41.36	187.36
	2	1	65.00	65.00		65.00	65.00		
	3	1	60.00	60.00		60.00	60.00		
	5	1	75.00	75.00		75.00	75.00		
	6	2	75.00	75.00	7.07	70.00	80.00	11.47	138.53
	7	1	75.00	75.00		75.00	75.00		
	8	1	76.00	76.00		76.00	76.00		
	9	1	81.00	81.00		81.00	81.00		

Table 14.3.5.16: Respiration rate - summary statistics for Stage 1 - all infusions, breaths per minute

Infusion	Observation	n	Mean	Median	SD	Min	Max	Lower 95% CI	Upper 95% CI
PRE		49	18.90	18.00	4.02	12.00	28.00	17.74	20.05
1	Pre-infusion	48	18.81	18.00	3.85	12.00	28.00	17.70	19.93
	1	48	20.13	20.00	4.08	12.00	28.00	18.94	21.31
	2	45	19.44	19.00	4.30	12.00	30.00	18.15	20.74
	3	38	19.82	18.00	4.20	14.00	30.00	18.43	21.20
	4	19	20.16	20.00	3.78	16.00	30.00	18.34	21.98
	5	5	22.60	20.00	5.18	18.00	30.00	16.17	29.03
	6	3	24.00	22.00	5.29	20.00	30.00	10.86	37.14
	7	2	22.00	22.00	2.83	20.00	24.00	-3.41	47.41
	8	1	20.00	20.00		20.00	20.00		
2	Pre-infusion	44	19.55	20.00	3.74	14.00	32.00	18.41	20.68
	1	43	19.74	20.00	4.14	12.00	32.00	18.47	21.02
	2	43	19.60	20.00	4.08	12.00	32.00	18.35	20.86
	3	35	19.00	19.00	3.83	12.00	30.00	17.69	20.31
	4	17	18.76	19.00	2.36	14.00	24.00	17.55	19.98
	5	5	19.40	20.00	2.41	16.00	22.00	16.41	22.39
	6	2	20.00	20.00	0.00	20.00	20.00	20.00	20.00
	7	1	20.00	20.00		20.00	20.00		
3	Pre-infusion	47	20.19	20.00	5.28	12.00	40.00	18.64	21.74
	1	45	20.09	19.00	5.32	12.00	40.00	18.49	21.69
	2	45	19.51	19.00	4.94	12.00	34.00	18.03	21.00
	3	34	18.79	18.00	4.21	12.00	32.00	17.33	20.26
	4	14	19.14	19.00	2.68	16.00	24.00	17.59	20.69
	5	6	20.00	19.50	3.52	15.00	24.00	16.30	23.70
	6	3	20.00	20.00	4.00	16.00	24.00	10.06	29.94

PRE = Pre-study Visit
EOP1 = End of Stage 1

Program: T0469.TEM, Version: 8.2, Datetime: 07FEB07:09:48

Table 14.3.5.16: Respiration rate - summary statistics for Stage 1 - all infusions, breaths per minute

Infusion	Observation	n	Mean	Median	SD	Min	Max	Lower 95% CI	Upper 95% CI
3	7	3	22.00	22.00	2.00	20.00	24.00	17.03	26.97

PRE = Pre-study Visit
EOP1 = End of Stage 1

Program: T0469.TEM, Version: 8.2, Datetime: 07FEB07:09:48

Table 14.3.5.16: Respiration rate - summary statistics for Stage 1 - all infusions, breaths per minute

Infusion	Observation	n	Mean	Median	SD	Min	Max	Lower 95% CI	Upper 95% CI
3A	Pre-infusion	2	18.50	18.50	0.71	18.00	19.00	12.15	24.85
	1	2	18.00	18.00	0.00	18.00	18.00	18.00	18.00
	2	2	18.00	18.00	0.00	18.00	18.00	18.00	18.00
	3	2	18.50	18.50	0.71	18.00	19.00	12.15	24.85
3B	Pre-infusion	2	18.50	18.50	0.71	18.00	19.00	12.15	24.85
	1	2	18.50	18.50	0.71	18.00	19.00	12.15	24.85
	2	2	18.50	18.50	0.71	18.00	19.00	12.15	24.85
	3	2	18.50	18.50	0.71	18.00	19.00	12.15	24.85
	4	2	18.50	18.50	0.71	18.00	19.00	12.15	24.85
	5	1	18.00	18.00		18.00	18.00		
4	Pre-infusion	48	20.00	20.00	5.15	12.00	44.00	18.50	21.50
	1	48	20.02	20.00	5.09	12.00	44.00	18.54	21.50
	2	48	19.90	20.00	4.74	12.00	38.00	18.52	21.27
	3	42	18.55	19.50	3.37	12.00	28.00	17.50	19.60
	4	29	17.66	18.00	3.19	12.00	24.00	16.44	18.87
	5	8	16.38	16.00	2.50	14.00	21.00	14.28	18.47
	6	2	14.00	14.00	0.00	14.00	14.00	14.00	14.00
5	Pre-infusion	47	20.13	20.00	4.42	14.00	36.00	18.83	21.42
	1	44	19.55	19.50	4.68	12.00	36.00	18.12	20.97
	2	36	18.53	18.00	3.86	12.00	32.00	17.22	19.83
	3	17	16.94	16.00	3.67	12.00	26.00	15.06	18.83
	4	3	14.67	14.00	3.06	12.00	18.00	7.08	22.26
6	Pre-infusion	45	19.84	18.00	4.65	12.00	32.00	18.45	21.24
	1	43	19.77	19.00	4.58	12.00	32.00	18.36	21.18

PRE = Pre-study Visit
EOP1 = End of Stage 1

Program: T0469.TEM, Version: 8.2, Datetime: 07FEB07:09:48

Table 14.3.5.16: Respiration rate - summary statistics for Stage 1 - all infusions, breaths per minute

Infusion	Observation	n	Mean	Median	SD	Min	Max	Lower 95% CI	Upper 95% CI
6	2	32	17.91	18.00	3.51	10.00	28.00	16.64	19.17
	3	13	18.08	18.00	3.86	12.00	25.00	15.74	20.41
	4	1	14.00	14.00		14.00	14.00		

PRE = Pre-study Visit
EOP1 = End of Stage 1

Program: T0469.TEM, Version: 8.2, Datetime: 07FEB07:09:48

Table 14.3.5.16: Respiration rate - summary statistics for Stage 1 - all infusions, breaths per minute

Infusion	Observation	n	Mean	Median	SD	Min	Max	Lower 95% CI	Upper 95% CI
7	Pre-infusion	37	20.24	20.00	4.48	12.00	30.00	18.75	21.74
	1	37	19.41	20.00	4.30	12.00	28.00	17.97	20.84
	2	32	18.88	19.50	4.36	8.00	28.00	17.30	20.45
	3	10	18.10	18.00	3.78	12.00	24.00	15.39	20.81
	4	2	20.00	20.00	8.49	14.00	26.00	-56.24	96.24
8	Pre-infusion	35	20.29	20.00	4.28	12.00	28.00	18.82	21.76
	1	35	20.49	20.00	4.81	12.00	32.00	18.83	22.14
	2	25	18.72	19.00	4.20	12.00	31.00	16.99	20.45
	3	7	17.00	16.00	2.77	14.00	21.00	14.44	19.56
	4	2	15.00	15.00	1.41	14.00	16.00	2.29	27.71
9	Pre-infusion	27	18.70	18.00	3.64	14.00	28.00	17.27	20.14
	1	26	18.96	20.00	4.20	12.00	30.00	17.27	20.66
	2	23	18.00	18.00	3.69	12.00	26.00	16.40	19.60
	3	7	18.43	18.00	2.44	16.00	23.00	16.17	20.68
	4	2	14.00	14.00	0.00	14.00	14.00	14.00	14.00
10	Pre-infusion	24	18.79	19.00	4.40	12.00	31.00	16.93	20.65
	1	22	17.73	19.00	3.17	12.00	22.00	16.32	19.13
	2	18	17.61	18.50	3.16	12.00	21.00	16.04	19.18
	3	5	17.80	16.00	4.49	14.00	24.00	12.22	23.38
	4	1	14.00	14.00		14.00	14.00		
11	Pre-infusion	22	19.86	20.00	4.06	12.00	28.00	18.06	21.66
	1	21	19.62	20.00	4.01	12.00	28.00	17.80	21.44
	2	19	19.21	20.00	4.16	12.00	28.00	17.21	21.21
	3	6	18.33	18.00	4.27	14.00	24.00	13.85	22.82

PRE = Pre-study Visit
EOP1 = End of Stage 1

Program: T0469.TEM, Version: 8.2, Datetime: 07FEB07:09:48

Table 14.3.5.16: Respiration rate - summary statistics for Stage 1 - all infusions, breaths per minute

Infusion	Observation	n	Mean	Median	SD	Min	Max	Lower 95% CI	Upper 95% CI
11	4	1	14.00	14.00		14.00	14.00		

PRE = Pre-study Visit
EOP1 = End of Stage 1

Program: T0469.TEM, Version: 8.2, Datetime: 07FEB07:09:48

Table 14.3.5.16: Respiration rate - summary statistics for Stage 1 - all infusions, breaths per minute

Infusion	Observation	n	Mean	Median	SD	Min	Max	Lower 95% CI	Upper 95% CI
12	Pre-infusion	18	19.61	20.00	5.79	10.00	32.00	16.73	22.49
	1	18	20.39	20.00	7.12	10.00	43.00	16.85	23.93
	2	13	18.38	19.00	4.70	10.00	30.00	15.54	21.22
	3	3	16.33	16.00	2.52	14.00	19.00	10.08	22.58
	4	1	14.00	14.00		14.00	14.00		
13	Pre-infusion	8	18.38	19.50	2.26	14.00	20.00	16.48	20.27
	1	9	18.56	20.00	2.70	14.00	21.00	16.48	20.63
	2	7	18.00	18.00	2.31	14.00	20.00	15.86	20.14
	3	3	17.33	16.00	4.16	14.00	22.00	6.99	27.68
	4	1	14.00	14.00		14.00	14.00		
14	Pre-infusion	8	18.25	19.50	2.66	14.00	20.00	16.03	20.47
	1	8	18.75	20.00	2.60	14.00	22.00	16.57	20.93
	2	6	18.67	19.50	2.16	16.00	21.00	16.40	20.93
	3	4	16.25	16.00	2.63	14.00	19.00	12.07	20.43
	4	1	14.00	14.00		14.00	14.00		
15	Pre-infusion	11	17.82	19.00	2.40	14.00	20.00	16.21	19.43
	1	10	17.30	17.50	3.20	14.00	22.00	15.01	19.59
	2	7	18.00	16.00	3.27	14.00	22.00	14.98	21.02
	3	2	14.00	14.00	0.00	14.00	14.00	14.00	14.00
	4	1	14.00	14.00		14.00	14.00		
16	Pre-infusion	16	20.75	20.00	5.51	12.00	32.00	17.82	23.68
	1	15	21.47	20.00	7.23	14.00	40.00	17.46	25.47
	2	6	19.33	16.00	6.89	14.00	30.00	12.10	26.56
	3	2	16.00	16.00	0.00	16.00	16.00	16.00	16.00

PRE = Pre-study Visit
EOP1 = End of Stage 1

Program: T0469.TEM, Version: 8.2, Datetime: 07FEB07:09:48

Table 14.3.5.16: Respiration rate - summary statistics for Stage 1 - all infusions, breaths per minute

Infusion	Observation	n	Mean	Median	SD	Min	Max	Lower 95% CI	Upper 95% CI
16	4	1	14.00	14.00		14.00	14.00		

PRE = Pre-study Visit
EOP1 = End of Stage 1

Program: T0469.TEM, Version: 8.2, Datetime: 07FEB07:09:48

Table 14.3.5.16: Respiration rate - summary statistics for Stage 1 - all infusions, breaths per minute

Infusion	Observation	n	Mean	Median	SD	Min	Max	Lower 95% CI	Upper 95% CI
17	Pre-infusion	5	18.40	20.00	2.19	16.00	20.00	15.68	21.12
	1	4	17.00	17.00	3.46	14.00	20.00	11.49	22.51
	2	2	14.00	14.00	0.00	14.00	14.00	14.00	14.00
	3	2	15.00	15.00	1.41	14.00	16.00	2.29	27.71
	4	1	14.00	14.00		14.00	14.00		
18	Pre-infusion	4	17.50	18.00	3.00	14.00	20.00	12.73	22.27
	1	4	17.50	18.00	3.00	14.00	20.00	12.73	22.27
	2	2	14.00	14.00	0.00	14.00	14.00	14.00	14.00
	3	2	14.00	14.00	0.00	14.00	14.00	14.00	14.00
	4	1	14.00	14.00		14.00	14.00		
19	Pre-infusion	6	18.00	20.00	3.10	14.00	20.00	14.75	21.25
	1	7	18.29	18.00	2.69	14.00	22.00	15.80	20.77
	2	4	17.00	17.00	2.58	14.00	20.00	12.89	21.11
	3	3	15.33	14.00	2.31	14.00	18.00	9.60	21.07
	4	1	14.00	14.00		14.00	14.00		
20	Pre-infusion	12	20.17	20.00	5.15	12.00	28.00	16.89	23.44
	1	10	24.40	20.00	9.47	16.00	48.00	17.63	31.17
	2	3	18.00	18.00	4.00	14.00	22.00	8.06	27.94
	3	1	16.00	16.00		16.00	16.00		
21	Pre-infusion	4	19.00	18.00	5.29	14.00	26.00	10.58	27.42
	1	4	18.50	18.00	4.43	14.00	24.00	11.44	25.56
	2	2	19.00	19.00	7.07	14.00	24.00	-44.53	82.53
	3	1	16.00	16.00		16.00	16.00		

PRE = Pre-study Visit
EOP1 = End of Stage 1

Program: T0469.TEM, Version: 8.2, Datetime: 07FEB07:09:48

Table 14.3.5.16: Respiration rate - summary statistics for Stage 1 - all infusions, breaths per minute

Infusion	Observation	n	Mean	Median	SD	Min	Max	Lower 95% CI	Upper 95% CI
22	Pre-infusion	4	17.50	18.00	2.52	14.00	20.00	13.50	21.50
	1	3	16.67	16.00	3.06	14.00	20.00	9.08	24.26
	2	2	17.00	17.00	1.41	16.00	18.00	4.29	29.71
	3	2	17.00	17.00	4.24	14.00	20.00	-21.12	55.12
23	Pre-infusion	5	22.00	20.00	6.00	14.00	28.00	14.55	29.45
	1	5	20.80	20.00	4.60	14.00	26.00	15.08	26.52
	2	2	20.00	20.00	5.66	16.00	24.00	-30.82	70.82
	3	2	19.00	19.00	7.07	14.00	24.00	-44.53	82.53
24	Pre-infusion	8	18.50	20.00	3.96	14.00	24.00	15.19	21.81
	1	8	19.75	20.00	4.20	14.00	26.00	16.24	23.26
	2	3	18.00	20.00	3.46	14.00	20.00	9.39	26.61
	3	1	14.00	14.00		14.00	14.00		
25	Pre-infusion	4	20.50	20.00	5.74	14.00	28.00	11.36	29.64
	1	4	20.00	20.00	4.90	14.00	26.00	12.20	27.80
	2	2	20.00	20.00	5.66	16.00	24.00	-30.82	70.82
	3	1	14.00	14.00		14.00	14.00		
26	Pre-infusion	4	18.25	17.50	6.45	12.00	26.00	7.99	28.51
	1	4	19.75	20.50	4.19	14.00	24.00	13.08	26.42
	2	3	19.33	18.00	4.16	16.00	24.00	8.99	29.68
	3	1	14.00	14.00		14.00	14.00		
27	Pre-infusion	5	17.40	18.00	2.41	14.00	20.00	14.41	20.39
	1	4	17.50	18.00	3.00	14.00	20.00	12.73	22.27
	2	3	18.00	18.00	2.00	16.00	20.00	13.03	22.97

PRE = Pre-study Visit
EOP1 = End of Stage 1

Program: T0469.TEM, Version: 8.2, Datetime: 07FEB07:09:48

Table 14.3.5.16: Respiration rate - summary statistics for Stage 1 - all infusions, breaths per minute

Infusion	Observation	n	Mean	Median	SD	Min	Max	Lower 95% CI	Upper 95% CI
27	3	2	15.00	15.00	1.41	14.00	16.00	2.29	27.71

PRE = Pre-study Visit
EOP1 = End of Stage 1

Program: T0469.TEM, Version: 8.2, Datetime: 07FEB07:09:48

Table 14.3.5.16: Respiration rate - summary statistics for Stage 1 - all infusions, breaths per minute

Infusion	Observation	n	Mean	Median	SD	Min	Max	Lower 95% CI	Upper 95% CI
28	Pre-infusion	10	21.60	21.00	5.40	14.00	28.00	17.74	25.46
	1	9	21.78	22.00	4.74	14.00	28.00	18.14	25.42
	2	1	14.00	14.00		14.00	14.00		
	3	1	14.00	14.00		14.00	14.00		
29	Pre-infusion	4	18.50	20.00	3.00	14.00	20.00	13.73	23.27
	1	4	18.00	19.00	2.83	14.00	20.00	13.50	22.50
	2	2	18.00	18.00	2.83	16.00	20.00	-7.41	43.41
	3	1	14.00	14.00		14.00	14.00		
30	Pre-infusion	4	17.50	18.00	2.52	14.00	20.00	13.50	21.50
	1	4	17.00	17.00	2.58	14.00	20.00	12.89	21.11
	2	3	15.33	14.00	2.31	14.00	18.00	9.60	21.07
	3	1	14.00	14.00		14.00	14.00		
EOP1		49	19.71	20.00	3.55	12.00	28.00	18.70	20.73

PRE = Pre-study Visit
EOP1 = End of Stage 1

Program: T0469.TEM, Version: 8.2, Datetime: 07FEB07:09:48

Table 14.3.5.17: Respiration rate - summary statistics for Stage 1 - Infusions 1-3 (pre-Subgam), breaths per minute

Observation	n	Mean	Median	SD	Min	Max	Lower 95% CI	Upper 95% CI
Pre-infusion	143	19.48	19.00	4.31	12.00	40.00	18.77	20.19
1	140	19.94	20.00	4.46	12.00	40.00	19.20	20.69
2	137	19.48	19.00	4.37	12.00	34.00	18.74	20.22
3	111	19.20	18.00	4.00	12.00	32.00	18.45	19.95
4	52	19.37	19.00	3.00	14.00	30.00	18.53	20.20
5	17	20.47	20.00	3.78	15.00	30.00	18.53	22.41
6	8	21.50	20.00	4.11	16.00	30.00	18.07	24.93
7	6	21.67	21.00	1.97	20.00	24.00	19.60	23.73
8	1	20.00	20.00		20.00	20.00		

Table 14.3.5.18: Respiration rate - summary statistics for Stage 1 - Infusions 4-30 (post-Subgam), breaths per minute

Observation	n	Mean	Median	SD	Min	Max	Lower 95% CI	Upper 95% CI
Pre-infusion	425	19.63	20.00	4.46	10.00	44.00	19.21	20.06
1	410	19.61	20.00	4.82	10.00	48.00	19.14	20.08
2	306	18.54	18.00	4.02	8.00	38.00	18.09	19.00
3	142	17.45	17.50	3.42	12.00	28.00	16.88	18.02
4	49	16.49	16.00	3.34	12.00	26.00	15.53	17.45
5	8	16.38	16.00	2.50	14.00	21.00	14.28	18.47
6	2	14.00	14.00	0.00	14.00	14.00	14.00	14.00

Table 14.3.5.19: Respiration rate - summary statistics for Stage 2 (post-Subgam), breaths per minute

Visit number	n	Mean	Median	SD	Min	Max	Lower 95% CI	Upper 95% CI
1	36	20.22	19.50	4.62	10.00	32.00	18.66	21.79
2	37	19.78	20.00	4.31	12.00	30.00	18.35	21.22
3	36	18.89	18.00	4.13	12.00	29.00	17.49	20.29
4	38	19.66	19.50	4.15	11.00	28.00	18.29	21.02
5	33	19.21	20.00	4.20	12.00	28.00	17.72	20.70
6	30	19.23	20.00	4.12	12.00	28.00	17.69	20.77
7	26	19.19	20.00	3.59	12.00	26.00	17.74	20.64
8	21	19.05	18.00	4.31	10.00	28.00	17.09	21.01
9	12	19.33	20.00	2.87	16.00	26.00	17.51	21.16
10	12	19.25	18.50	2.45	16.00	26.00	17.69	20.81
11	6	18.50	19.00	2.17	16.00	21.00	16.22	20.78
12	5	18.40	18.00	1.67	16.00	20.00	16.32	20.48
13	6	19.00	18.00	2.76	16.00	24.00	16.11	21.89
14	4	18.50	17.00	3.79	16.00	24.00	12.48	24.52
15	2	17.50	17.50	0.71	17.00	18.00	11.15	23.85
16	2	17.00	17.00	1.41	16.00	18.00	4.29	29.71
17	1	17.00	17.00		17.00	17.00		
18	1	16.00	16.00		16.00	16.00		
20	2	17.00	17.00	1.41	16.00	18.00	4.29	29.71
21	2	18.50	18.50	3.54	16.00	21.00	-13.27	50.27
22	2	17.50	17.50	2.12	16.00	19.00	-1.56	36.56
23	1	20.00	20.00		20.00	20.00		
24	1	16.00	16.00		16.00	16.00		
25	1	16.00	16.00		16.00	16.00		
28	1	14.00	14.00		14.00	14.00		
EOP2	46	18.28	17.50	3.66	14.00	32.00	17.20	19.37

EOP1 = End of Stage 1

Program: T0472.TEM, Version: 8.2, Datetime: 07FEB07:09:48

Table 14.3.5.20: Respiration rate - summary statistics for pharmacokinetic assessments, breaths per minute

Visit	Days post-infusion	n	Mean	Median	SD	Min	Max	Lower 95% CI	Upper 95% CI
1st Subgam infusion	Pre-infusion	4	19.50	19.00	4.12	16.00	24.00	12.94	26.06
	1	2	17.00	17.00	1.41	16.00	18.00	4.29	29.71
	2	2	15.50	15.50	0.71	15.00	16.00	9.15	21.85
	3	1	16.00	16.00		16.00	16.00		
	5	1	15.00	15.00		15.00	15.00		
	6	2	14.00	14.00	0.00	14.00	14.00	14.00	14.00
	7	1	15.00	15.00		15.00	15.00		
After approximately 3 months on Subgam	Pre-infusion	2	19.00	19.00	7.07	14.00	24.00	-44.53	82.53
	2	1	16.00	16.00		16.00	16.00		
	3	1	14.00	14.00		14.00	14.00		
	5	1	20.00	20.00		20.00	20.00		
	6	2	18.00	18.00	2.83	16.00	20.00	-7.41	43.41
	7	1	20.00	20.00		20.00	20.00		
	8	1	20.00	20.00		20.00	20.00		
	9	1	20.00	20.00		20.00	20.00		

Table 14.3.5.21: Temperature - summary statistics for Stage 1 - all infusions, ~C

Infusion	Observation	n	Mean	Median	SD	Min	Max	Lower 95% CI	Upper 95% CI
PRE		48	36.57	36.60	0.42	35.60	37.70	36.45	36.69
1	Pre-infusion	48	36.56	36.55	0.43	35.60	37.70	36.44	36.68
	1	48	36.56	36.50	0.42	35.60	37.80	36.44	36.68
	2	46	36.52	36.50	0.42	35.30	37.80	36.39	36.64
	3	38	36.54	36.55	0.41	35.60	37.40	36.40	36.67
	4	19	36.45	36.50	0.43	35.50	37.10	36.24	36.66
	5	6	36.65	36.60	0.43	36.20	37.40	36.20	37.10
	6	4	36.75	36.55	0.44	36.50	37.40	36.06	37.44
	7	2	36.75	36.75	0.35	36.50	37.00	33.57	39.93
	8	1	37.10	37.10		37.10	37.10		
2	Pre-infusion	44	36.46	36.45	0.46	35.20	37.30	36.32	36.60
	1	45	36.52	36.50	0.38	35.80	37.30	36.40	36.63
	2	45	36.53	36.60	0.43	35.50	37.30	36.40	36.66
	3	37	36.64	36.60	0.38	35.90	37.40	36.51	36.76
	4	19	36.68	36.70	0.43	36.00	37.40	36.48	36.89
	5	8	36.80	36.65	0.53	36.00	37.60	36.36	37.24
	6	3	36.90	36.70	0.44	36.60	37.40	35.82	37.98
	7	1	36.90	36.90		36.90	36.90		
3	Pre-infusion	47	36.53	36.60	0.41	35.30	37.40	36.40	36.65
	1	46	36.55	36.60	0.44	35.30	37.40	36.42	36.69
	2	46	36.58	36.60	0.35	35.80	37.40	36.47	36.68
	3	35	36.64	36.60	0.59	35.80	38.60	36.44	36.85
	4	16	36.66	36.60	0.46	35.90	37.90	36.42	36.91
	5	7	36.66	36.40	0.47	36.20	37.40	36.22	37.09
	6	3	36.83	37.00	0.38	36.40	37.10	35.89	37.77

PRE = Pre-study visit
EOP1 = End of Stage 1

Program: T0474.TEM, Version: 8.2, Datetime: 07FEB07:09:48

Table 14.3.5.21: Temperature - summary statistics for Stage 1 - all infusions, -C

Infusion	Observation	n	Mean	Median	SD	Min	Max	Lower 95% CI	Upper 95% CI
3	7	3	36.40	36.40	0.10	36.30	36.50	36.15	36.65

PRE = Pre-study Visit
EOP1 = End of Stage 1

Program: T0474.TEM, Version: 8.2, Datetime: 07FEB07:09:48

Table 14.3.5.21: Temperature - summary statistics for Stage 1 - all infusions, ~C

Infusion	Observation	n	Mean	Median	SD	Min	Max	Lower 95% CI	Upper 95% CI
3A	Pre-infusion	2	36.30	36.30	0.14	36.20	36.40	35.03	37.57
	1	2	36.40	36.40	0.00	36.40	36.40	36.40	36.40
	2	2	36.40	36.40	0.00	36.40	36.40	36.40	36.40
	3	2	36.35	36.35	0.21	36.20	36.50	34.44	38.26
3B	Pre-infusion	2	36.90	36.90	0.14	36.80	37.00	35.63	38.17
	1	2	36.90	36.90	0.14	36.80	37.00	35.63	38.17
	2	2	36.90	36.90	0.14	36.80	37.00	35.63	38.17
	3	2	36.90	36.90	0.14	36.80	37.00	35.63	38.17
	4	2	36.95	36.95	0.07	36.90	37.00	36.31	37.59
	5	1	36.80	36.80		36.80	36.80		
4	Pre-infusion	50	36.56	36.55	0.42	35.30	37.50	36.44	36.68
	1	50	36.56	36.60	0.41	35.30	37.40	36.45	36.68
	2	49	36.58	36.60	0.39	35.30	37.40	36.47	36.69
	3	43	36.60	36.60	0.33	35.80	37.30	36.50	36.70
	4	27	36.47	36.50	0.32	35.50	37.00	36.35	36.60
	5	8	36.46	36.50	0.37	36.00	37.00	36.16	36.77
	6	2	36.35	36.35	0.07	36.30	36.40	35.71	36.99
5	Pre-infusion	48	36.51	36.50	0.38	35.50	37.20	36.40	36.63
	1	46	36.64	36.60	0.41	36.00	37.40	36.52	36.76
	2	38	36.56	36.60	0.48	35.50	37.50	36.40	36.71
	3	17	36.45	36.30	0.37	35.60	36.90	36.26	36.64
	4	3	36.23	36.20	0.25	36.00	36.50	35.61	36.86
6	Pre-infusion	46	36.56	36.60	0.36	35.90	37.50	36.45	36.66
	1	44	36.63	36.65	0.38	35.90	37.40	36.52	36.75

PRE = Pre-study Visit
EOP1 = End of Stage 1

Program: T0474.TEM, Version: 8.2, Datetime: 07FEB07:09:48

Table 14.3.5.21: Temperature - summary statistics for Stage 1 - all. infusions, ~C

Infusion	Observation	n	Mean	Median	SD	Min	Max	Lower 95% CI	Upper 95% CI
6	2	32	36.63	36.70	0.34	35.90	37.30	36.51	36.75
	3	13	36.58	36.60	0.39	36.00	37.50	36.35	36.82
	4	1	37.00	37.00		37.00	37.00		

PRE = Pre-study Visit
EOP1 = End of Stage 1

Program: T0474.TEM, Version: 8.2, Datetime: 07FEB07:09:48

Table 14.3.5.21: Temperature - summary statistics for Stage 1 - all infusions, ~C

Infusion	Observation	n	Mean	Median	SD	Min	Max	Lower 95% CI	Upper 95% CI
7	Pre-infusion	41	36.64	36.70	0.34	36.10	37.30	36.53	36.74
	1	40	36.58	36.60	0.37	35.60	37.60	36.46	36.70
	2	32	36.56	36.50	0.34	35.80	37.20	36.43	36.68
	3	10	36.55	36.55	0.22	36.10	36.80	36.39	36.71
	4	2	36.80	36.80	0.57	36.40	37.20	31.72	41.88
8	Pre-infusion	38	36.58	36.70	0.50	35.00	37.40	36.42	36.75
	1	37	36.55	36.60	0.43	35.60	37.40	36.41	36.69
	2	26	36.55	36.55	0.40	35.70	37.20	36.39	36.71
	3	7	36.49	36.40	0.35	36.00	36.90	36.16	36.81
	4	2	36.25	36.25	0.07	36.20	36.30	35.61	36.89
9	Pre-infusion	33	36.58	36.50	0.51	35.80	38.60	36.40	36.76
	1	27	36.60	36.50	0.50	36.00	38.40	36.41	36.80
	2	24	36.44	36.45	0.40	35.70	37.40	36.27	36.61
	3	7	36.49	36.40	0.31	36.10	36.90	36.20	36.78
	4	2	36.30	36.30	0.14	36.20	36.40	35.03	37.57
10	Pre-infusion	32	36.44	36.55	0.54	35.00	37.20	36.24	36.63
	1	25	36.52	36.50	0.38	35.80	37.10	36.37	36.68
	2	21	36.50	36.60	0.50	35.20	37.20	36.27	36.73
	3	5	36.22	36.40	0.30	35.70	36.40	35.84	36.60
	4	1	37.10	37.10		37.10	37.10		
11	Pre-infusion	28	36.46	36.50	0.50	34.90	37.30	36.27	36.66
	1	22	36.57	36.65	0.40	35.60	37.20	36.39	36.75
	2	19	36.58	36.70	0.33	35.80	37.10	36.43	36.74
	3	6	36.78	36.75	0.23	36.50	37.20	36.54	37.03

PRE = Pre-study Visit
EOP1 = End of Stage 1

Program: T0474.TEM, Version: 8.2, Datetime: 07FEB07:09:48

Table 14.3.5.21: Temperature - summary statistics for Stage 1 - all infusions, ~C

Infusion	Observation	n	Mean	Median	SD	Min	Max	Lower 95% CI	Upper 95% CI
11	4	1	36.90	36.90		36.90	36.90		

PRE = Pre-study Visit
EOP1 = End of Stage 1

Program: T0474.TEM, Version: 8.2, Datetime: 07FEB07:09:48

Table 14.3.5.21: Temperature - summary statistics for Stage 1 - all infusions, ~C

Infusion	Observation	n	Mean	Median	SD	Min	Max	Lower 95% CI	Upper 95% CI
12	Pre-infusion	25	36.69	36.70	0.45	35.70	38.00	36.51	36.88
	1	20	36.66	36.60	0.32	36.10	37.30	36.51	36.81
	2	14	36.44	36.50	0.48	35.50	37.20	36.17	36.72
	3	3	37.07	37.00	0.31	36.80	37.40	36.31	37.83
	4	1	36.70	36.70		36.70	36.70		
13	Pre-infusion	16	36.29	36.50	0.76	35.00	38.00	35.88	36.69
	1	7	36.43	36.30	0.48	35.90	37.20	35.98	36.87
	2	7	36.33	36.40	0.35	35.80	36.80	36.01	36.65
	3	3	36.43	36.40	0.25	36.20	36.70	35.81	37.06
	4	1	36.70	36.70		36.70	36.70		
14	Pre-infusion	15	36.43	36.70	0.69	35.00	37.60	36.05	36.81
	1	8	36.26	36.20	0.44	35.60	37.00	35.89	36.63
	2	6	36.47	36.45	0.34	36.10	36.90	36.11	36.82
	3	4	36.53	36.50	0.15	36.40	36.70	36.29	36.76
	4	1	37.00	37.00		37.00	37.00		
15	Pre-infusion	16	36.59	36.65	0.35	35.80	37.10	36.40	36.77
	1	11	36.45	36.40	0.36	36.00	37.20	36.20	36.69
	2	8	36.53	36.55	0.51	35.80	37.40	36.10	36.95
	3	2	36.50	36.50	0.42	36.20	36.80	32.69	40.31
	4	1	36.20	36.20		36.20	36.20		
16	Pre-infusion	23	36.50	36.40	0.49	35.70	37.90	36.28	36.71
	1	15	36.47	36.40	0.44	35.90	37.40	36.22	36.71
	2	6	36.15	36.40	0.42	35.40	36.40	35.71	36.59
	3	2	36.90	36.90	0.28	36.70	37.10	34.36	39.44

PRE = Pre-study Visit
EOP1 = End of Stage 1

Program: T0474.TEM, Version: 8.2, Datetime: 07FEB07:09:48

Table 14.3.5.21: Temperature - summary statistics for Stage 1 - all infusions, ~C

Infusion	Observation	n	Mean	Median	SD	Min	Max	Lower 95% CI	Upper 95% CI
16	4	1	36.60	36.60		36.60	36.60		

PRE = Pre-study Visit
EOP1 = End of Stage 1

Program: T0474.TEM, Version: 8.2, Datetime: 07FEB07:09:48

Table 14.3.5.21: Temperature - summary statistics for Stage 1 - all infusions, ~C

Infusion	Observation	n	Mean	Median	SD	Min	Max	Lower 95% CI	Upper 95% CI
17	Pre-infusion	13	36.41	36.20	0.72	35.30	38.00	35.97	36.84
	1	4	36.53	36.50	0.22	36.30	36.80	36.17	36.88
	2	2	36.60	36.60	0.14	36.50	36.70	35.33	37.87
	3	2	36.65	36.65	0.21	36.50	36.80	34.74	38.56
	4	1	36.60	36.60		36.60	36.60		
18	Pre-infusion	14	36.49	36.65	0.73	35.40	37.90	36.07	36.92
	1	4	36.45	36.40	0.34	36.10	36.90	35.91	36.99
	2	2	35.90	35.90	0.00	35.90	35.90	35.90	35.90
	3	2	36.05	36.05	0.35	35.80	36.30	32.87	39.23
	4	1	36.20	36.20		36.20	36.20		
19	Pre-infusion	15	36.57	36.50	0.63	35.10	38.00	36.22	36.92
	1	7	36.66	36.50	0.62	36.10	37.90	36.08	37.23
	2	4	36.58	36.55	0.33	36.20	37.00	36.05	37.10
	3	3	36.37	36.50	0.32	36.00	36.60	35.57	37.17
	4	1	36.60	36.60		36.60	36.60		
20	Pre-infusion	18	36.48	36.45	0.41	35.80	37.20	36.28	36.69
	1	10	36.39	36.35	0.69	34.80	37.40	35.89	36.89
	2	3	36.40	36.10	0.70	35.90	37.20	34.66	38.14
	3	1	36.30	36.30		36.30	36.30		
21	Pre-infusion	12	36.41	36.60	0.55	35.30	37.00	36.06	36.76
	1	4	36.65	36.75	0.40	36.10	37.00	36.01	37.29
	2	2	36.60	36.60	1.13	35.80	37.40	26.44	46.76
	3	1	36.30	36.30		36.30	36.30		

PRE = Pre-study Visit
EOP1 = End of Stage 1

Program: T0474.TEM, Version: 8.2, Datetime: 07FEB07:09:48

Table 14.3.5.21: Temperature - summary statistics for Stage 1 - all infusions, ~C

Infusion	Observation	n	Mean	Median	SD	Min	Max	Lower 95% CI	Upper 95% CI
22	Pre-infusion	11	36.45	36.70	0.57	35.40	37.10	36.06	36.83
	1	3	36.57	36.60	0.06	36.50	36.60	36.42	36.71
	2	2	36.40	36.40	0.28	36.20	36.60	33.86	38.94
	3	2	36.50	36.50	0.14	36.40	36.60	35.23	37.77
23	Pre-infusion	13	36.45	36.40	0.31	35.90	36.80	36.26	36.64
	1	5	36.34	36.20	0.24	36.10	36.60	36.04	36.64
	2	2	36.50	36.50	0.42	36.20	36.80	32.69	40.31
	3	2	36.10	36.10	0.42	35.80	36.40	32.29	39.91
24	Pre-infusion	16	36.43	36.30	0.42	35.70	37.30	36.21	36.66
	1	8	36.54	36.60	0.54	35.90	37.40	36.09	36.99
	2	3	36.53	36.50	0.55	36.00	37.10	35.17	37.90
	3	1	36.30	36.30		36.30	36.30		
25	Pre-infusion	10	36.31	36.20	0.54	35.60	37.10	35.92	36.70
	1	4	36.03	36.25	1.01	34.60	37.00	34.41	37.64
	2	2	35.95	35.95	0.78	35.40	36.50	28.96	42.94
	3	1	36.00	36.00		36.00	36.00		
26	Pre-infusion	10	36.43	36.60	0.49	35.60	36.90	36.08	36.78
	1	4	36.18	36.25	0.29	35.80	36.40	35.72	36.63
	2	3	36.27	36.40	0.42	35.80	36.60	35.23	37.30
	3	1	36.60	36.60		36.60	36.60		
27	Pre-infusion	12	36.36	36.40	0.28	35.80	36.80	36.18	36.54
	1	4	36.35	36.35	0.29	36.00	36.70	35.89	36.81
	2	3	36.13	36.20	0.80	35.30	36.90	34.14	38.13

PRE = Pre-study Visit
EOP1 = End of Stage 1

Program: T0474.TEM, Version: 8.2, Datetime: 07FEB07:09:48

Table 14.3.5.21: Temperature - summary statistics for Stage 1 - all infusions, ~C

Infusion	Observation	n	Mean	Median	SD	Min	Max	Lower 95% CI	Upper 95% CI
27	3	2	36.10	36.10	0.14	36.00	36.20	34.83	37.37

PRE = Pre-study Visit
EOP1 = End of Stage 1

Program: T0474.TEM, Version: 8.2, Datetime: 07FEB07:09:48

Table 14.3.5.21: Temperature - summary statistics for Stage 1 - all infusions, ~C

Infusion	Observation	n	Mean	Median	SD	Min	Max	Lower 95% CI	Upper 95% CI
28	Pre-infusion	18	36.49	36.50	0.50	35.50	37.70	36.24	36.74
	1	10	36.47	36.55	0.56	35.50	37.20	36.07	36.87
	2	2	36.25	36.25	0.64	35.80	36.70	30.53	41.97
	3	2	36.75	36.75	0.64	36.30	37.20	31.03	42.47
29	Pre-infusion	9	36.59	36.60	0.50	35.90	37.60	36.21	36.97
	1	4	36.23	36.35	0.43	35.60	36.60	35.53	36.92
	2	2	36.40	36.40	0.42	36.10	36.70	32.59	40.21
	3	1	36.00	36.00		36.00	36.00		
30	Pre-infusion	8	36.53	36.55	0.44	35.80	37.20	36.16	36.89
	1	4	36.35	36.40	0.26	36.00	36.60	35.93	36.77
	2	3	36.03	36.00	0.25	35.80	36.30	35.41	36.66
	3	1	36.30	36.30		36.30	36.30		
EOP1		49	36.49	36.50	0.41	35.00	37.30	36.37	36.61

PRE = Pre-study Visit
EOP1 = End of Stage 1

Program: T0474.TEM, Version: 8.2, Datetime: 07FEB07:09:48

Table 14.3.5.22: Temperature - summary statistics for Stage 1 - Infusions 1-3 (pre-Subgam), ~C

Observation	n	Mean	Median	SD	Min	Max	Lower 95% CI	Upper 95% CI
Pre-infusion	143	36.52	36.50	0.43	35.20	37.70	36.45	36.59
1	143	36.55	36.50	0.41	35.30	37.80	36.48	36.62
2	141	36.54	36.60	0.40	35.30	37.80	36.48	36.61
3	114	36.60	36.60	0.46	35.60	38.60	36.52	36.69
4	56	36.61	36.60	0.44	35.50	37.90	36.49	36.73
5	22	36.71	36.65	0.45	36.00	37.60	36.51	36.91
6	10	36.82	36.65	0.38	36.40	37.40	36.55	37.09
7	6	36.60	36.50	0.28	36.30	37.00	36.30	36.90
8	1	37.10	37.10		37.10	37.10	.	

Table 14.3.5.23: Temperature - summary statistics for Stage 1 - Infusions 4-30 (post-Subgam), °C

Observation	n	Mean	Median	SD	Min	Max	Lower 95% CI	Upper 95% CI
Pre-infusion	590	36.51	36.50	0.48	34.90	38.60	36.48	36.55
1	427	36.55	36.60	0.43	34.60	38.40	36.51	36.59
2	317	36.51	36.50	0.42	35.20	37.50	36.47	36.56
3	144	36.53	36.50	0.35	35.60	37.50	36.47	36.58
4	47	36.51	36.50	0.33	35.50	37.20	36.41	36.60
5	8	36.46	36.50	0.37	36.00	37.00	36.16	36.77
6	2	36.35	36.35	0.07	36.30	36.40	35.71	36.99

Program: T0476.TEM, Version: 8.2, Datetime: 07FEB07:09:48

Table 14.3.5.26: Temperature - summary statistics for pharmacokinetic assessments, ~C

Visit	Days.post-infusion	n	Mean	Median	SD	Min	Max	Lower 95% CI	Upper 95% CI
1st Subgam infusion	Pre-infusion	4	36.78	36.70	0.30	36.50	37.20	36.30	37.25
	1	3	36.27	36.50	0.40	35.80	36.50	35.26	37.27
	2	3	36.17	36.30	0.51	35.60	36.60	34.89	37.44
	3	2	36.15	36.15	0.78	35.60	36.70	29.16	43.14
	4	1	36.50	36.50		36.50	36.50		
	5	1	36.20	36.20		36.20	36.20		
	6	2	35.85	35.85	0.21	35.70	36.00	33.94	37.76
	7	1	36.00	36.00		36.00	36.00		
After approximately 3 months on Subgam	Pre-infusion	2	36.50	36.50	0.14	36.40	36.60	35.23	37.77
	2	1	36.50	36.50		36.50	36.50		
	3	1	36.40	36.40		36.40	36.40		
	5	1	35.80	35.80		35.80	35.80		
	6	2	36.10	36.10	0.14	36.00	36.20	34.83	37.37
	7	1	35.00	35.00		35.00	35.00		
	8	1	35.60	35.60		35.60	35.60		
	9	1	36.00	36.00		36.00	36.00		

Table 14.3.5.27: Summary of pre-infusion body temperature >37°C in Stage 1

Pre / Post Subgam	Infusion	No. observations (patients)	No. observations (No. patients) with temperature >37°C	% of observations with temperature >37°C	% of patients with temperature >37°C
Pre-Subgam	PRE	48 (48)	4 (4)	8.3	8.3
	1	48 (48)	5 (5)	10.4	10.4
	2	44 (44)	4 (4)	9.1	9.1
	3	47 (47)	4 (4)	8.5	8.5
	3A	2 (2)	0 (0)	0	0
	3B	2 (2)	0 (0)	0	0
	Total	191 (49)	17 (9)	8.9	18.4
Post-Subgam	4	50 (50)	6 (6)	12.0	12.0
	5	48 (48)	3 (3)	6.3	6.3
	6	46 (46)	2 (2)	4.3	4.3
	7	41 (41)	5 (5)	12.2	12.2
	8	38 (38)	5 (5)	13.2	13.2
	9	33 (33)	3 (3)	9.1	9.1
	10	32 (32)	2 (2)	6.3	6.3
	11	28 (28)	2 (2)	7.1	7.1
	12	25 (25)	3 (3)	12.0	12.0
	13	16 (16)	2 (2)	12.5	12.5
	14	15 (15)	2 (2)	13.3	13.3
	15	16 (16)	1 (1)	6.3	6.3
	16	23 (23)	2 (2)	8.7	8.7
	17	13 (13)	2 (2)	15.4	15.4
	18	14 (14)	3 (3)	21.4	21.4
	19	15 (15)	3 (3)	20.0	20.0
	20	18 (18)	2 (2)	11.1	11.1
	21	12 (12)	0 (0)	0	0
	22	11 (11)	1 (1)	9.1	9.1
	23	13 (13)	0 (0)	0	0
	24	16 (16)	2 (2)	12.5	12.5

PRE = Pre-study Visit

Program: T0480.TEM, Version: 8.2, Datetime: 07FEB07:09:48

Table 14.3.5.27: Summary of pre-infusion body temperature >37°C in Stage 1

Pre / Post Subgam	Infusion	No. observations (patients)	No. observations (No. patients) with temperature >37°C	% of observations with temperature >37°C	% of patients with temperature >37°C
Post-Subgam	25	10 (10)	1 (1)	10.0	10.0
	26	10 (10)	0 (0)	0	0
	27	12 (12)	0 (0)	0	0
	28	18 (18)	2 (2)	11.1	11.1
	29	9 (9)	1 (1)	11.1	11.1
	30	8 (8)	1 (1)	12.5	12.5
	Total	590 (50)	56 (19)	9.5	38.0
All infusions	Total	781 (50)	73 (22)	9.3	44.0

PRE = Pre-study Visit

Program: T0480.TEM, Version: 8.2, Datetime: 07FEB07:09:48

Table 14.3.5.28: Summary of post-infusion body temperature >37°C in Stage 1

Pre / Post Subgam	Infusion	No. observations (patients)	No. observations (No. patients) with temperature >37°C	% of observations with temperature >37°C	% of patients with temperature >37°C
Pre-Subgam	1	164 (48)	16 (8)	9.8	16.7
	2	158 (45)	23 (11)	14.6	24.4
	3	156 (46)	21 (11)	13.5	23.9
	3A	6 (2)	0 (0)	0	0
	3B	9 (2)	0 (0)	0	0
	Total	493 (49)	60 (17)	12.2	34.7
Post-Subgam	4	179 (50)	15 (10)	8.4	20.0
	5	104 (46)	14 (9)	13.5	19.6
	6	90 (44)	7 (6)	7.8	13.6
	7	84 (40)	6 (5)	7.1	12.5
	8	72 (37)	8 (6)	11.1	16.2
	9	60 (27)	3 (2)	5.0	7.4
	10	52 (25)	5 (5)	9.6	20.0
	11	48 (22)	4 (2)	8.3	9.1
	12	38 (20)	4 (3)	10.5	15.0
	13	18 (8)	1 (1)	5.6	12.5
	14	19 (8)	0 (0)	0	0
	15	22 (11)	2 (2)	9.1	18.2
	16	24 (15)	2 (2)	8.3	13.3
	17	9 (4)	0 (0)	0	0
	18	9 (4)	0 (0)	0	0
	19	15 (7)	1 (1)	6.7	14.3
	20	14 (10)	3 (3)	21.4	30.0
	21	7 (4)	1 (1)	14.3	25.0
	22	7 (3)	0 (0)	0	0
	23	9 (5)	0 (0)	0	0
	24	12 (8)	2 (2)	16.7	25.0
	25	7 (4)	0 (0)	0	0

Table 14.3.5.28: Summary of post-infusion body temperature >37°C in Stage 1

Pre / Post Subgam	Infusion	No. observations (patients)	No. observations (No. patients) with temperature >37°C	% of observations with temperature >37°C	% of patients with temperature >37°C
Post-Subgam	26	8 (4)	0 (0)	0	0
	27	9 (4)	0 (0)	0	0
	28	14 (10)	2 (2)	14.3	20.0
	29	7 (4)	0 (0)	0	0
	30	8 (4)	0 (0)	0	0
	Total	945 (50)	80 (22)	8.5	44.0
All infusions	Total	1438 (50)	140 (29)	9.7	58.0

Table 14.3.5.29: Number of reported body temperature elevations of >37°C in Stage 1

Pre / Post Subgam	Infusion	No. observations (patients)	Pre-infusion temperature of <37°C followed by post-infusion temperature of >37°C		
			No. observations (No. patients) with temperature >37°C	% of observations with temperature >37°C	% of patients with temperature >37°C
Pre-Subgam	PRE	48 (48)	0 (0)	0	0
	1	212 (48)	21 (9)	9.9	18.8
	2	202 (45)	27 (12)	13.4	26.7
	3	203 (47)	25 (11)	12.3	23.4
	3A	8 (2)	0 (0)	0	0
	3B	11 (2)	0 (0)	0	0
	Total	684 (49)	73 (18)	10.7	36.7
Post-Subgam	4	229 (50)	21 (12)	9.2	24.0
	5	152 (48)	17 (11)	11.2	22.9
	6	136 (46)	9 (6)	6.6	13.0
	7	125 (41)	11 (8)	8.8	19.5
	8	110 (38)	13 (8)	11.8	21.1
	9	93 (33)	6 (3)	6.5	9.1
	10	84 (32)	7 (6)	8.3	18.8
	11	76 (28)	6 (3)	7.9	10.7
	12	63 (25)	6 (4)	9.5	16.0
	13	34 (16)	1 (1)	2.9	6.3
	14	34 (15)	0 (0)	0	0
	15	38 (16)	3 (2)	7.9	12.5
	16	47 (23)	2 (2)	4.3	8.7
	17	22 (13)	0 (0)	0	0
	18	23 (14)	0 (0)	0	0
	19	30 (15)	1 (1)	3.3	6.7
	20	32 (18)	3 (3)	9.4	16.7
	21	19 (12)	1 (1)	5.3	8.3

PRE = Pre-study visit

Program: T0482.TEM, Version: 8.2, Datetime: 07FEB07:09:48

Table 14.3.5.29: Number of reported body temperature elevations of >37°C in Stage 1

Pre / Post Subgam	Infusion	No. observations (patients)	Pre-infusion temperature of <37°C followed by post-infusion temperature of >37°C		
			No. observations (No. patients) with temperature >37°C	% of observations with temperature >37°C	% of patients with temperature >37°C
Post-Subgam	22	18 (11)	0 (0)	0	0
	23	22 (13)	0 (0)	0	0
	24	28 (16)	4 (2)	14.3	12.5
	25	17 (10)	0 (0)	0	0
	26	18 (10)	0 (0)	0	0
	27	21 (12)	0 (0)	0	0
	28	32 (18)	3 (3)	9.4	16.7
	29	16 (9)	0 (0)	0	0
	30	16 (8)	0 (0)	0	0
	Total	1535 (50)	114 (25)	7.4	50.0
All infusions	Total	2219 (50)	187 (31)	8.4	62.0

PRE = Pre-study Visit

Program: T0482.TEM, Version: 8.2, Datetime: 07FEB07:09:48

Table 14.3.5.30: Number of adverse events reported with Preferred Term of 'Pyrexia' by relatedness to study drug, diagnosis, age group and prior therapy

Number of events (number of patients)

Pre-Subgam	Patient Subgroups	n	Relatedness		
			Related	Not Related	Unknown
	All patients	0 (0)	0 (0)	0 (0)	0 (0)

Adults (>=20y), Teenagers (>=12-<20y), Children (<12y)

Program: T0483.SAS, Version: 8.2, Datetime: 07FEB07:09:48

Table 14.3.5.30: Number of adverse events reported with Preferred Term of 'Pyrexia' by relatedness to study drug, diagnosis, age group and prior therapy

Number of events (number of patients)

Post-Subgam	Patient Subgroups	n	Relatedness		
			Related	Not Related	Unknown
	All patients	27 (10)	1 (1)	26 (9)	0 (0)
By Age Group	Adult	17 (5)	0 (0)	17 (5)	0 (0)
	Teenager	1 (1)	0 (0)	1 (1)	0 (0)
	Child	9 (4)	1 (1)	8 (3)	0 (0)
By Diagnosis of PAD	CVID/XLA	21 (8)	1 (1)	20 (7)	0 (0)
	Other	6 (2)	0 (0)	6 (2)	0 (0)
By Prior Therapy	SCIG	10 (3)	1 (1)	9 (2)	0 (0)
	IVIG	17 (7)	0 (0)	17 (7)	0 (0)

Adults (>=20y), Teenagers (>=12-<20y), Children (<12y)

Program: T0483.SAS, Version: 8.2, Datetime: 07FEB07:09:48

Table 14.3.5.30: Number of adverse events reported with Preferred Term of 'Pyrexia' by relatedness to study drug, diagnosis, age group and prior therapy

Number of events (number of patients)

All Infusions	Patient Subgroups	n	Relatedness		
			Related	Not Related	Unknown
	All patients	27 (10)	1 (1)	26 (9)	0 (0)
By Age Group	Adult	17 (5)	0 (0)	17 (5)	0 (0)
	Teenager	1 (1)	0 (0)	1 (1)	0 (0)
	Child	9 (4)	1 (1)	8 (3)	0 (0)
By Diagnosis of PAD	CVID/XLA	21 (8)	1 (1)	20 (7)	0 (0)
	Other	6 (2)	0 (0)	6 (2)	0 (0)
By Prior Therapy	SCIG	10 (3)	1 (1)	9 (2)	0 (0)
	IVIG	17 (7)	0 (0)	17 (7)	0 (0)

Adults (>=20y), Teenagers (>=12-<20y), Children (<12y)

Program: T0483.SAS, Version: 8.2, Datetime: 07FEB07:09:48

Table 14.3.5.31: Number of reported temperature elevations of >37°C with and without corresponding(a) adverse events with preferred term of 'pyrexia' by relatedness to study drug, diagnosis, age group and prior therapy
Number of events (number of patients)

Pre-Subgam	Patient Subgroups	n	Temperature of >37°C WITHOUT corresponding(a) AE of preferred term of pyrexia	Temperature of >37°C WITH corresponding(a) AE of preferred term of pyrexia
All patients		77 (18)	77 (18)	0 (0)
By Age Group	Adult	37 (10)	37 (10)	0 (0)
	Teenager	20 (2)	20 (2)	0 (0)
	Child	20 (6)	20 (6)	0 (0)
By Diagnosis of PAD	CVID/XLA	63 (11)	63 (11)	0 (0)
	Other	14 (7)	14 (7)	0 (0)
By Prior Therapy	SCIG	7 (2)	7 (2)	0 (0)
	IVIG	70 (16)	70 (16)	0 (0)

Adults (>=20y), Teenagers (>=12-<20y), Children (<12y)
(a) For definition of 'corresponding' see Section 6.5.1 of statistical analysis plan

Program: T0484.SAS, Version: 8.2, Datetime: 07FEB07:09:48

Table 14.3.5.31: Number of reported temperature elevations of >37°C with and without corresponding(a) adverse events with preferred term of 'pyrexia' by relatedness to study drug, diagnosis, age group and prior therapy
Number of events (number of patients)

Post-Subgam	Patient Subgroups	n	Temperature of >37°C WITHOUT corresponding(a) AE of preferred term of pyrexia	Temperature of >37°C WITH corresponding(a) AE of preferred term of pyrexia
All patients		141 (27)	128 (27)	13 (5)
By Age Group	Adult	102 (18)	90 (18)	12 (4)
	Teenager	11 (2)	11 (2)	0 (0)
	Child	28 (7)	27 (7)	1 (1)
By Diagnosis of PAD	CVID/XLA	91 (17)	78 (17)	13 (5)
	Other	50 (10)	50 (10)	0 (0)
By Prior Therapy	SCIG	5 (4)	5 (4)	0 (0)
	IVIG	136 (23)	123 (23)	13 (5)

Adults (>=20y), Teenagers (>=12-<20y), Children (<12y)
(a) For definition of 'corresponding' see Section 6.5.1 of statistical analysis plan

Program: T0484.SAS, Version: 8.2, Datetime: 07FEB07:09:48

Table 14.3.5.31: Number of reported temperature elevations of >37°C with and without corresponding(a) adverse events with preferred term of 'pyrexia' by relatedness to study drug, diagnosis, age group and prior therapy
Number of events (number of patients)

All Infusions	Patient Subgroups	n	Temperature of >37°C WITHOUT corresponding(a) AE of preferred term of pyrexia	Temperature of >37°C WITH corresponding(a) AE of preferred term of pyrexia
All patients		218 (31)	205 (31)	13 (5)
By Age Group	Adult	139 (20)	127 (20)	12 (4)
	Teenager	31 (3)	31 (3)	0 (0)
	Child	48 (8)	47 (8)	1 (1)
By Diagnosis of PAD	CVID/XLA	154 (20)	141 (20)	13 (5)
	Other	64 (11)	64 (11)	0 (0)
By Prior Therapy	SCIG	12 (5)	12 (5)	0 (0)
	IVIG	206 (26)	193 (26)	13 (5)

Adults (>=20y), Teenagers (>=12-<20y), Children (<12y)
(a) For definition of 'corresponding' see Section 6.5.1 of statistical analysis plan

Program: T0484.SAS, Version: 8.2, Datetime: 07FEB07:09:48
