# Supplementary material for: Experience with Subgam, a Subcutaneously Administered Human Normal Immunoglobulin (ClinicalTrials.gov - NCT02247141)
Source: PLoS One. 2015 Jul 29;10(7):e0131565. doi: 10.1371/journal.pone.0131565 (PMC4519338; doi:10.1371/journal.pone.0131565)
Supplement: S1 Protocol — A multi-centre, open study to assess the safety and efficacy of 16% immunoglobulin product given via the subcutaneous route in primary antibody deficient patients. (PDF) [file pone.0131565.s003.pdf]

Protocol and Protocol Amendments

- Final Protocol, April 1999
- Final Protocol Version 2, September 1999
- Final Protocol Version 3, April 2000
- Final Protocol Version 3 - Amendment 1, 31st March 2000
- Final Protocol Version 3 - Amendment 2, 25th July 2001
- Final Protocol Version 3 - Amendment 3, 29th January 2004

A MULTI-CENTRE, OPEN STUDY TO ASSESS THE SAFETY AND EFFICACY OF A 16% IMMUNOGLOBULIN PRODUCT GIVEN VIA THE SUBCUTANEOUS ROUTE IN PRIMARY ANTIBODY DEFICIENT PATIENTS

SCIG01

FINAL VERSION

APRIL 1999

BIO PRODUCTS LABORATORY
DAGGER LANE
ELSTREE
HERTS WD6 3BX

Tel: 0181 258 2200
Fax: 0181 258 2611

A MULTI-CENTRE, OPEN STUDY TO ASSESS THE SAFETY AND EFFICACY OF A 16% IMMUNOGLOBULIN PRODUCT GIVEN VIA THE SUBCUTANEOUS ROUTE IN PRIMARY ANTIBODY DEFICIENT PATIENTS

SCIG01

FINAL VERSION

APRIL 1999

MEDICAL DIRECTOR:	Dr Clive H Dash<br>Bio Products Laboratory<br>Dagger Lane<br>Elstree<br>Herts WD6 3BX<br>Tel: 0181 258 2565<br>Fax: 0181 258 2611
CRA:	Pauline Jackson<br>Senior Clinical Research Associate<br>Tel: 0181 258 2248<br>Fax: 0181 258 2611

A MULTI-CENTRE, OPEN STUDY TO ASSESS THE SAFETY AND EFFICACY OF A 16% IMMUNOGLOBULIN PRODUCT GIVEN VIA THE SUBCUTANEOUS ROUTE IN PRIMARY ANTIBODY DEFICIENT PATIENTS

SCIG01

INVESTIGATOR DETAILS

NAME: ________________ TITLE : ________________

QUALIFICATIONS: ________________

ADDRESS: ________________

____

____

CO-INVESTIGATORS/CO-WORKERS DETAILS

NAME: ________________ TITLE : ________________

NAME: ________________ TITLE : ________________

NAME: ________________ TITLE : ________________

NAME: ________________ TITLE : ________________

NAME: ________________ TITLE : ________________

TABLE OF CONTENTS

1.0 LIST OF ABBREVIATIONS 1
2.0 SYNOPSIS 2
3.0 INTRODUCTION 4
4.0 STUDY OBJECTIVES 5
5.0 STUDY DESIGN 6
6.0 STUDY POPULATION 6
6.1 Source of Subjects 6
6.2 Inclusion Criteria 6
6.3 Exclusion Criteria 6
7.0 STUDY DRUGS 7
7.1 Presentation 7
7.2 Dose and route of administration 8
7.3 Storage 9
7.4 Concurrent Medication 10
7.5 Drug Accountability 11
8.0 STUDY SCHEDULE - PHASE 1 11
8.1 Pre-study Procedures 11
8.2 Study Procedures 12
8.2.1 Clinical Monitoring During Infusion 12
8.2.2 Laboratory Measurements 13
8.2.3 Laboratory Sampling in Children 15
8.3 End of Study Procedures 15
8.4 Blood Sample Collection, Handling and Labelling 16
9.0 HOME THERAPY PROCEDURES 17
9.1 Criteria for Home Therapy 17
9.2 Home Therapy Training 17
9.3 Clinical Monitoring during Home Therapy 18
9.4 Blood Sampling during Home Therapy 18
10.0 STUDY SCHEDULE - PHASE 2 19
11.0 STUDY SCHEDULE - PHARMACOKINETICS 20

11.1 Pharmacokinetic Procedures 20
12.0 EFFICACY MEASURES 20
12.1 Primary Efficacy Measurement 20
12.2 Secondary Efficacy Measurements 20
13.0 SAFETY MEASUREMENTS 21
14.0 WITHDRAWAL OF SUBJECTS 21
15.0 ADVERSE EVENTS 22
15.1 Definition of Serious Adverse Events 22
15.2 Detecting Adverse Events 22
15.3 Completing Adverse Event Forms 23
15.4 Assessment of adverse event intensity 23
15.5 Attribution to study drugs 23
15.6 Reporting Adverse Events 24
15.7 Adverse Event Follow-up 26
16.0 DATA ANALYSIS 26
16.1 Statistical Analysis 26
16.2 Definition of Evaluability 27
16.3 Drop-outs 27
16.5 Interim Analysis 27
17.0 DOCUMENTATION 27
17.1 Required Pre-Study Documentation 27
17.2 Recording data in Case Record Forms 28
17.3 Error Correction 29
17.4 Signing Off and Return of Case Record Forms 29
17.5 Maintenance and Archiving of Study Records 29
18. STUDY CONDUCT 31
18.1 Adherence to the Protocol 31
18.2 Protocol amendments 32
18.3 Early Cessation of the Study 32
18.4 Monitoring Visits and Audit 33
18.5 Report and Publication 34
18.6 Obligations of the Investigator 34
18.7 Confidentiality 34

19.0 ETHICAL CONSIDERATIONS 34
19.1 Informed Consent 35
19.2 Compensation/Indemnity 36
20.0 INVESTIGATOR'S STATEMENT 37
23.0 REFERENCES 38
APPENDIX 1:ADMINISTRATION OF SUBCUTANEOUS INFUSION 39
APPENDIX 2: ADULT PATIENT INFORMATION SHEET 40
APPENDIX 3: CONSENT FORM 43
APPENDIX 2(i): PAEDIATRIC PATIENT INFORMATION SHEET 44
APPENDIX 3(i): PAEDIATRIC CONSENT FORM 47
APPENDIX 4: EXAMPLE GP LETTER 48
APPENDIX 5: HOME THERAPY MANUAL 49
APPENDIX 6: HOME THERAPY CONSENT FORM 51
APPENDIX 7: INFUSION RECORD 52
APPENDIX 8: LOCAL INVESTIGATOR'S RESPONSIBILITIES BASED ON THE ICH GUIDELINES FOR GOOD CLINICAL PRACTICE 53
APPENDIX 9: DECLARATION OF HELSINKI 65
APPENDIX 10: STUDY FLOW CHART 70
APPENDIX 11: STUDY PROCEDURES 72

1.0 LIST OF ABBREVIATIONS

AE	Adverse Event
ADR	Adverse Reaction
ALT	Alanine aminotransferase
AST	Aspartate aminotransferase
BPL	Bio Products Laboratory
CPMP	Committee on Proprietary Medicinal Products
CRF	Case Record Form
CRA	Clinical Research Associate
CTX	Clinical Trial Exemption
CV	Curriculum Vitae
EC	Ethics Committee
GCP	Good Clinical Practice
GGT	Gamma glutamyl transferase
GP	General Practitioner
HbsAg	Hepatitis B surface antigen
HCV	Hepatitis C virus
HIB	Haemophilus Influenzae type B
HIV	Human Immunodeficiency Virus
IgA	Immunoglobulin A
IgG	Immunoglobulin G
IgM	Immunoglobulin M
iu	International Units
ICH	International Conference on Harmonisation
IVIG	Intravenous immunoglobulin
LDH	Lactate dehydrogenase
LREC	Local Research Ethics Committee
MCA	Medicines Control Agency
MREC	Multicentre Research Ethics Committee
PCR	Polymerase Chain Reaction
RBC	Red Blood Cell
SAE	Serious Adverse Event
SCIG	Subcutaneous Immunoglobulin
SOP	Standard Operating Procedure
WBC	White Blood Cell

2.0 SYNOPSIS

A multi-centre, open study to assess the safety and efficacy of a 16% immunoglobulin product given via the subcutaneous route in primary antibody deficient patients.

Up to 40 adult and paediatric patients, aged 0-75 years, stable on intravenous immunoglobulin therapy (IVIG) or subcutaneous immunoglobulin therapy (SCIG), will be enrolled.

Patients will be eligible to enter the study if, in the investigator's opinion, their disease is stable. In order to collect baseline data, patients will continue to receive their usual immunoglobulin, whether IVIG or SCIG, for 3 infusions before commencing the study subcutaneous immunoglobulin. Starting one week after their final treatment they will receive the 16% immunoglobulin product subcutaneously at a dose of 100mg/kg bodyweight. Weekly infusions will then continue to be administered for a period of 6 months. The dose and frequency of the 16% immunoglobulin product given, in order to maintain an adequate serum IgG level, will be tailored to suit the patient. This will be decided by the investigator, but a minimum target IgG trough level of 6g/L will be aimed for.

There will be provision for patients to administer SCIG at home, if appropriate, after a period of training. These patients will be required to complete diary cards during each home infusion and to attend the clinic at 1-4 weekly intervals for blood sampling.

After 6 months of SCIG treatment (Phase 1), patients will then either return to their usual immunoglobulin treatment or continue with the 16% immunoglobulin in Phase 2 of the study which is a safety follow-on phase. During Phase 2, patients will be asked to complete regular diary cards and return for clinic visits at 3 monthly intervals.

Patients will attend the clinic at the following time-points:-

STUDY PHASE	VISIT/INFUSION NUMBER	VISIT DETAILS
**Phase 1**	Prestudy	Up to 28 days prior to study entry
	Infusions 1-3	3 infusions of patient's routine immunoglobulin at usual frequency and dose prior to study SCIG to collect baseline data
	Infusions 4 -30	Starting 1 week after their final routine treatment patients will receive weekly study SCIG infusions for 6 months.
**Phase 2**	Infusions 31 etc (until product is marketed)	Patients will be invited to take part in the safety follow-on phase. They will receive weekly SCIG infusions, complete regular diary cards and attend the clinic at 3 monthly intervals.
**Pharmacokinetics**	PK 1 a-g (optional)<br>PK 2 a-g (optional)	Selected patients will be asked to attend daily clinic visits after first study SCIG infusion for 1 week and repeated after 3 months SCIG

Primary Efficacy Measurement:

- The proportion of trough levels where IgG $\geq$ 4g/L

Secondary Efficacy Measurements:

- The change in SCIG dose to maintain adequate trough levels
- The proportion of trough levels where IgG $\geq$ 6g/L
- Time taken to reach steady state IgG trough level
- The mean change in IgG trough level from baseline
- Number of infections and days off work/school

Safety Measurements:

- Number, type, severity and duration of adverse events occurring throughout the study.
- Laboratory monitoring of haematology, biochemistry and immunological and viral markers
- Monitoring of vital signs

Patient/parent/guardian satisfaction of the subcutaneous route will be assessed by way of a short questionnaire at the beginning, middle and end of treatment.

3.0 INTRODUCTION

Patients who are diagnosed with primary antibody deficiency usually require regular immunoglobulin replacement therapy in order to avoid serious infection. The efficacy of life-long intravenous immunoglobulin (IVIG) therapy is well established and is a major contributor to improved health and quality of life for these patients[1]. However, IVIG treatment for most patients requires regular visits to hospital where the infusion can take many hours to administer depending on the dose and tolerance to the treatment.

Intramuscular and slow subcutaneous infusions of immunoglobulins are alternatives to IVIG but are not without limitations. Intramuscular injections are often painful, the dose that can be safely delivered is limited and the side-effects can be severe. Slow subcutaneous infusions, on the other hand, while benefiting from a better safety profile, are time-consuming and cumbersome usually requiring overnight infusions[2].

Rapid subcutaneous infusions of immunoglobulin preparations intended for intramuscular use were first described in 1991 by Gardulf et al[3]. Patients were given simultaneous infusions totaling 34-40ml/hr via dual portable pumps. In addition to there being very few (0.93%) mild systemic reactions experienced

with this method of administration, patients were also able to infuse the immunoglobulin at home after a period of training.

BPL's Human Normal Immunoglobulin is mainly IgG and is indicated for prophylaxis against hepatitis A infection and replacement therapy in primary antibody deficiency. It is manufactured from venous plasma and is currently licensed for intramuscular use. This study will use an almost identical product, obtained from the plasma of screened donors in the United States. However, the composition of this 16% immunoglobulin product is such that it would also be suitable for subcutaneous use since it is virtually identical to the product used routinely in Scandinavia[3,4], Kabiglobulin. Both products are approximately 16% immunoglobulin, mercury-free and have a very low IgA content which contributes to their subcutaneous tolerance. In addition, they have a solvent/detergent step included in the manufacturing process which inactivates lipid-enveloped viruses such as HIV, hepatitis C (HCV) and hepatitis B (HBV). To date there have been no reports of these viruses being transmitted by BPL's intramuscular product.

The following study has therefore been designed to assess the efficacy and safety of giving a 16% immunoglobulin product via rapid subcutaneous infusion to patients with primary antibody deficiency.

4.0 STUDY OBJECTIVES

1. To determine the efficacy of giving patients with primary antibody deficiency, weekly subcutaneous infusions of a 16% immunoglobulin product at a dose tailored to achieve a minimum trough serum IgG level of 6g/L.

2. To determine the safety of giving patients with primary antibody deficiency, weekly subcutaneous infusions of a 16% immunoglobulin

product at a dose tailored to achieve a minimum trough serum IgG level of 6g/L.

5.0 STUDY DESIGN

A multi-centre, open, safety and efficacy study of weekly subcutaneous 16% immunoglobulin.

6.0 STUDY POPULATION

6.1 Source of Subjects

The trial will be conducted as a multi-centre study. Up to a total of 40 adult and paediatric patients with a diagnosis of primary antibody deficiency will be enrolled. This number will allow for drop-outs and withdrawals. Patients can either be receiving IVIG or SCIG as their normal replacement therapy before entry into the study and a minimum number of 10 of each type will be aimed for in order for an adequate number to be analysed.

6.2 Inclusion Criteria

a) Diagnosis of primary antibody deficiency syndrome
b) Aged 0-75 years
c) Stable disease and has been receiving immunoglobulin (IVIG or SCIG) for the past 6 months
d) Written informed consent (patient/parent/guardian).

6.3 Exclusion Criteria

a) Known to be intolerant to IgA
b) Pregnant women or women who are breast feeding
c) History of clinically significant renal or hepatic disease or known renal or hepatic abnormalities

d) Unsuitable for the purposes of the study (in the opinion of the investigator)

e) Participation in another clinical trial within the last 30 days

f) History of allergic reactions to intravenous blood products

g) History of infection within the last 2 months, requiring IV antibiotics

h) At the beginning of the study, is known to require treatment with another blood product during the course of the study.

7.0 STUDY DRUGS

7.1 Presentation

The immunoglobulin used in this study is a liquid preparation containing 16% immunoglobulin, mainly immunoglobulin G (IgG, gammaglobulin).

The product is presented in a 5ml vial containing 750mg of protein. If available, 10ml vials containing 1500mg of protein will also be used. 1ml of solution contains the following approximate amounts:

Human protein (at least 95% IgG)	140-180mg
Sodium Chloride	0.009g
Glycine	0.006g
Sodium Acetate	0.002g

The product is stored in containers which are drawn vials of neutral borosilicate glass with a 13mm neck. The closure is a 13mm diameter overseal, consisting of a snap-off polypropylene cap, a clear lacquered aluminium skirt and a halobutyl rubber wad.

7.2 Dose and route of administration

Patients enrolled will continue to receive their usual immunoglobulin infusion at the same dose and frequency for 3 treatments prior to receiving study SCIG in order for baseline data to be collected. However, where patients are currently receiving IVIG and are needing to change rapidly to SCIG due to poor venous access, a minimum of 1 baseline IVIG treatment would be acceptable. If patients are currently receiving SCIG they will not be required to undergo any IVIG treatments and can commence SCIG with the study product once they have completed three subcutaneous infusions of their usual immunoglobulin.

Once study product is commenced, the 16% immunoglobulin will be given by subcutaneous infusion via a portable syringe driver (Graseby, MS16A) at an initial starting dose of 100mg/kg bodyweight. Subsequent SCIG infusions will continue to be administered at weekly intervals for a period of 6 months and the dose will be tailored to suit the patient's trough levels of IgG. The dose and frequency of the 16% immunoglobulin product given, in order to maintain an adequate serum IgG level, will be decided by the investigator but a minimum target IgG trough level of 6g/L will be aimed for.

The 16% immunoglobulin product should be given by separate infusion and not mixed with other infusion material.

For an adult, the first SCIG infusion should be given at a slow rate of 10mls/hr increasing over the next 4-8 infusions by 1-2mls/hr until the recommended maximum rate of 20mls/hr is reached. Two sites may be simultaneously used at this rate, via 2 syringe drivers, giving a combined maximum rate of 40mls/hr.

For a child, the first infusion should be given at a slow rate of 5mls/hr increasing by 0.5-1mls/hr over the next 4-8 infusions until the

recommended maximum rate of 10mls/hr is reached. Two sites may be simultaneously used at this rate, via 2 syringe drivers, giving a combined maximum rate of 20mls/hr.

Either the abdomen or the thigh can be used as the site of infusion for both adults and children. Most patients seem to prefer abdominal siting of the infusions although very young children tolerate the infusions better in the thigh than in the abdomen.

Instructions for administration of subcutaneous immunoglobulin can be found in Appendix 1.

7.3 Storage

The 16% immunoglobulin product should be stored in its carton in the dark and has a shelf life of 2 years if stored between 2-8^{0}C or 1 week if stored at 25^{0}C. DO NOT FREEZE.

The 16% immunoglobulin product is for single use only; any used materials and unused solution should be discarded by approved means.

The condition of date-expired or incorrectly stored product cannot be guaranteed. Such product may be unsafe and should not be used. Solutions which are cloudy or have deposits should not be used.

The dose volume for each vial size is specified on the label.

The 16% immunoglobulin product for clinical trial use should be stored separately from routine product, as product reconciliation has to be done in accordance with the ICH guidelines for GCP.

7.4 Concurrent Medication

The 16% immunoglobulin product is believed not to affect the immune response to bacterial vaccines but could reduce the response to some virus vaccines and toxoids. However it should be noted that attempts to vaccinate patients with primary antibody deficiency may not be completely effective even if immunoglobulin has not been administered.

With administration of the 16% immunoglobulin product, a broad spectrum of antibodies is passively administered. These antibodies will interfere with the response to live vaccines, especially the MMR (measles, mumps, rubella) vaccine and varicella vaccine. Such vaccines should therefore be given at least 3 weeks before or 3 months after administration of immunoglobulin. This does not apply to yellow fever vaccine however since the immunoglobulin product obtained from US plasma is unlikely to contain antibody to this virus.

All medications taken by the patient or administered to the patient during the study will be recorded as concurrent medication. In addition, any drugs prescribed by the investigator or the patient's G.P. throughout the trial will also be regarded as concurrent medication.

The following information on all concurrent medication must be recorded in the CRF:-

- the name of the drug and its pharmaceutical form
- the reason for treatment
- the dose
- the duration of treatment

7.5 Drug Accountability

It is essential that accurate drug accountability records are maintained to ensure that the total number of 16% immunoglobulin product vials dispatched, received and returned by a study site can be established.

This is facilitated by completion of dispatch notes (by BPL), receipt forms, stock control logs, dispensing/returns logs and returns forms (by Pharmacy or equivalent) and the recording at each infusion of the batch number and number of grams and vials given in the patient's notes and CRF (investigator or appointed personnel).

The 16% immunoglobulin product should be used solely as indicated in the protocol and must not be used on patients not in the trial without the prior agreement of BPL.

8.0 STUDY SCHEDULE - PHASE 1

8.1 Pre-study Procedures

Prior to enrolling a patient, the investigator should ensure that the patient fulfills the inclusion/exclusion criteria.

Potential subjects for the study, or their parent/guardian, will be given a detailed, oral presentation of the nature, purpose, risks and requirements of the study, in addition to receiving detailed information (Appendix 2). They will be given adequate time to consider participating in the study and the opportunity to ask the study physician about any aspect of the study. Once satisfied, the patient/parent/guardian will be asked to sign a Consent Form (Appendix 3). A letter will be sent to the patient's G.P. (Appendix 4).

Subsequently, the patient will be allocated a Subject Number, provided by BPL, for identification purposes and then undergo screening, which

will take place no more than 28 days prior to Infusion 1. The pre-study assessment will consist of:

- Medical history
- Physical examination including height, weight, sitting blood pressure, pulse, temperature and respiration
- Patient Satisfaction Questionnaire
- Blood samples as detailed in the following table:

**Haematology**	Haemoglobin, Haematocrit, RBC, WBC, Neutrophils, Lymphocytes, Monocytes, Eosinophils, Basophils, Platelet Count, Reticulocytes
**Blood Group Serology**	ABO (D),<br>Direct Coombs' Test
**Biochemistry**	Sodium, Potassium, Creatinine, LDH, ALT (and/or AST), GGT, Total Bilirubin, Alkaline Phosphatase, Haptoglobin
**Immunology**	IgG, IgA, IgM
**Specific Antibodies**	Pneumococcus, HIB
**Repeat Test - Stored**	Separate serum aliquot to be stored at -20^{0}C in case of repeat testing (5ml clotted)

8.2 Study Procedures

8.2.1 Clinical Monitoring During Infusion

Vital signs (body temperature, respiration, pulse and sitting blood pressure) and adverse events since the last infusion, will be recorded prior to each infusion, at hourly intervals during the infusion and again at the end. The infusion site(s) will be inspected during and at the end of the infusion for signs of irritation. Any untoward irritation and/or swelling will be recorded as an adverse event in the CRF. The

patient/parent/guardian will also be asked to complete a Patient Satisfaction Questionnaire at approximately week 12.

8.2.2 Laboratory Measurements

The following immunology samples will be taken immediately prior to each infusion throughout Phase 1. In addition, for patients on IVIG, a post-infusion sample will be taken immediately after the last routine treatment:

**Immunology**	IgG, IgA, IgM

Once the patient has completed all routine treatments, the following baseline samples will be taken before any study 16% subcutaneous immunoglobulin product is administered:

**Parvovirus**<br>Parvovirus B19 (PCR)	5ml clotted
**Virology**<br>Baseline samples of HBsAg, HIV (PCR) for adults and HCV (PCR) will be stored at -70^{0}C and tested at the end of the study if the patient is found to be positive	5ml clotted
**Archive**<br>Sample to be stored at -70^{0}C for 15 years	5ml clotted

One week after the first study SCIG infusion has been given, the following sample for parvovirus will be taken:

**Parvovirus**<br>Parvovirus B19 (PCR)	5ml clotted

The following laboratory measurement will be taken at 4 weekly intervals throughout Phase 1:

**Liver Function**	ALT (and/or AST)
**Specific Antibodies**	Pneumococcus, HIB

The following laboratory measurements will only be assessed if clinically indicated throughout Phase 1:

**Haematology**	Haemoglobin, Haematocrit, RBC, WBC, Neutrophils, Lymphocytes, Monocytes, Eosinophils, Basophils, Platelet Count, Reticulocytes
**Biochemistry**	Sodium, Potassium, Creatinine, LDH, ALT (and/or AST), GGT, Total Bilirubin, Alkaline Phosphatase, Haptoglobin

At each timepoint, a repeat sample will be taken in case of laboratory error or loss of sample. In the case of clinically indicated samples, only when a sample is required to be taken will a repeat test sample also be taken at the same time:

**Repeat Test - Stored**<br>Separate serum aliquot to be stored at $-20^{0}C$ in case of repeat testing	5ml clotted

During the study, any abnormal laboratory values that are considered to be clinically significant by the investigator must be repeated, using the stored sample, as soon as possible to rule out laboratory error.

Values outside the normal range for the laboratory, even those which are not considered to be clinically significant by the investigator must be commented on, on the laboratory report.

All adverse events will be recorded in the CRF.

Patients considered suitable for home therapy will follow the schedule laid out in section 9.

8.2.3 Laboratory Sampling in Children

Laboratory sampling procedures defined by the protocol must be adhered to in the case of all adult patients over the age of 18 years. Where children are involved, all baseline samples should be obtained wherever possible, in particular, immunology and virology (except HIV testing which is not mandatory in children). Thereafter, as many subsequent blood samples as the individual is prepared to undergo or if sampling is clinically indicated should be collected in order to obtain adequate safety and efficacy data. End of study virology samples (except HIV) must also be collected to exclude viral transmission.

8.3 End of Study Procedures

The following assessments will be performed on all patients at the end of Phase 1 one week following the final study SCIG:

- Physical examination including weight, sitting blood pressure, pulse, temperature and respiration
- Patient Satisfaction Questionnaire
- Blood samples as follows:

**Haematology**	Haemoglobin, Haematocrit, RBC, WBC, Neutrophils, Lymphocytes, Monocytes, Eosinophils, Basophils, Platelet Count, Reticulocytes
**Biochemistry**	Sodium, Potassium, Creatinine, LDH, ALT (and/or AST), GGT, Total Bilirubin, Alkaline Phosphatase, Haptoglobin
**Immunology**	IgG, IgA, IgM
**Virology** (if not entering Phase 2)	HBsAg, HIV (PCR), HCV (PCR) (5ml clotted)

**Archive - Stored** (if not entering Phase 2) Plasma sample to be stored at -70^{0}C for 15 years	5ml clotted
**Repeat Test - Stored** Separate serum aliquot to be stored at -20^{0}C in case of repeat testing	5ml clotted

8.4 Blood Sample Collection, Handling and Labelling

Blood samples for haematology and biochemistry will be tested locally. The labelling, handling and testing of samples will be performed according to local procedures. Each blood sample must have the following minimum information:

- Study Number (SCIG01)
- Subject Number
- Date and time of collection

Virology and Parvovirus PCR will be performed by Central Public Health Laboratories, Colindale, London. Samples will be sent by courier to arrive by 10:00am on the day following collection. For Parvovirus samples, the serum will be separated from clot within 2 hours of clot formation.

A 5ml aliquot of serum will be stored at -70^{0}C as a baseline sample immediately prior to the first SCIG infusion at infusion 4 and again at the end of Phase 1. These must be clearly identified as above and kept at the centre for a minimum of 15 years from the time that the study is completed.

A separate 5ml aliquot of serum will be stored at the centre at -20^{0}C or below, each time a study sample is obtained, in case of repeat sampling.

9.0 HOME THERAPY PROCEDURES

9.1 Criteria for Home Therapy

Self-infusion of SCIG will only be offered to suitable patients. The following criteria must be satisfied before a patient is to be considered eligible for home therapy:-

1. In the opinion of the investigator, the patient/parent/guardian must be motivated to perform the infusion.
2. After a period of training by the study staff, the patient/parent/guardian's ability to manage the infusion techniques must be satisfactory.
3. The patient's G.P. must agree to the patient performing home therapy.
4. A relative/friend who has also been trained in the techniques must be available to assist with each infusion performed at home.
5. The patient/parent/guardian must be contactable by telephone.

9.2 Home Therapy Training

A period of training will take place over 8-12 weekly infusions. The patient/parent/guardian will be trained to draw up the 16% immunoglobulin into a syringe, prime the tubing and insert the needle correctly, maintaining an aseptic technique throughout the procedure. The patient/parent/guardian will be instructed on how to use a syringe driver and also how to recognise adverse reactions and the appropriate action to take. Once the patient/parent/guardian is confident and proficient in all aspects of home therapy and when the investigator is satisfied that the patient/parent/guardian has reached the required standard to safely administer SCIG, they will be provided with a Home Therapy Manual (Appendix 5) and asked to sign a Home Therapy Consent Form (Appendix 6).

The criteria in section 9.1 must be satisfied before patient's can receive SCIG at home. In addition, the patient's G.P. having already agreed in principle, must also be informed when the patient commences home therapy. This must be documented in the patient's notes.

9.3 Clinical Monitoring during Home Therapy

Prior to each infusion, the patient/parent/guardian will record the temperature and detail the result on the diary card provided. If the patient's temperature is above the level pre-specified by the investigator and/or the patient is feverish, the SCIG should be delayed until the patient/parent/guardian has sought advice from the investigator using the contact number provided.

The following details will be routinely recorded on the Infusion Diary Card (Appendix 7):

- Infusion date
- Temperature (^{0}C)
- Site(s) used (A=abdomen, T= thigh)
- Dose per site (mg)
- Total dose (mg)
- Rate (mm/hr)
- Batch number
- Comments/Adverse reactions

9.4 Blood Sampling during Home Therapy

If practical, the patient should return to the clinic on a weekly basis for the same blood samples as patients receiving their infusions in hospital. However, since home therapy is intended to provide the patient with a more flexible regimen, blood samples taken every 4 weeks should be scheduled as a minimum. If it is clinically indicated or if local

procedures require however, the patient will be asked to return for more frequent blood sampling. This will be discussed and agreed with the patient on an individual basis.

Therefore, for the purposes of the study, the following minimum blood samples should be obtained from adult patients on home therapy:

Timepoints	Blood Tests
Pre-study	All tests detailed in section 8.1
Infusions 1-3<br>IVIG (if applicable)	All tests detailed in section 8.2.2
Infusion 4<br>SCIG	All tests detailed in section 8.2.2
Infusions 8, 12, 16, 20, 24, 28<br>(i.e. every 4 weeks)	All tests detailed in section 8.2.2
End of Study	All tests detailed in section 8.3

If the patient attends the clinic for an unscheduled visit, additional blood samples for immunology, haematology and biochemistry should also be taken as clinically indicated as well as a repeat test sample.

10.0 STUDY SCHEDULE - PHASE 2

At the end of Phase 1, patients will be invited to enter the safety follow-on study (Phase 2) and continue receiving subcutaneous 16% immunoglobulin either in hospital or at home until such time that they wish to discontinue treatment or the product is available on the market. Patients will be asked to attend study visits at the clinic at 3 monthly intervals throughout Phase 2 when they will be asked whether they have experienced any adverse events since the last visit. If clinically indicated, blood sample for haematology, biochemistry and/or immunology will also be taken. If the patient decides to withdraw from Phase 2 before the product is marketed, a sample for virology and an archive blood sample will be taken to exclude viral transmission during Phase 2.

11.0 STUDY SCHEDULE - PHARMACOKINETICS

Patients who can conveniently attend the clinic for daily blood sampling will be invited to participate in the pharmacokinetic part of the study. Patients who agree to take part will be asked to return to the clinic every week day for 1 week after the first study SCIG has been infused. This will be repeated for a further week, 3 months later.

11.1 Pharmacokinetic Procedures

Vital signs (body temperature, respiration, pulse and sitting blood pressure) and adverse events will be recorded at each visit. The following laboratory samples will be collected on each day that the patient attends for the pharmacokinetic part of the study:

**Immunology**	IgG, IgA, IgM
**Repeat Test - Stored**<br>Separate serum aliquot to be stored at -20^0C in case of repeat testing	5ml clotted

If clinically indicated, blood samples for haematology and biochemistry will also be taken.

12.0 EFFICACY MEASURES

12.1 Primary Efficacy Measurement

- The proportion of trough levels where IgG $\geq$ 4g/L

12.2 Secondary Efficacy Measurements

- The change in SCIG dose to maintain adequate trough levels
- The proportion of trough levels where IgG $\geq$ 6g/L
- Time taken to reach steady state IgG trough level
- The mean change in IgG trough level from baseline
- Number of infections and days off work/school

13.0 SAFETY MEASUREMENTS

- Number, type, severity and duration of adverse events occurring throughout the study.
- Laboratory monitoring of haematology, biochemistry and immunological and viral markers
- Monitoring of vital signs

Patient/parent/guardian's satisfaction of the subcutaneous route will be assessed by way of a short questionnaire at the beginning, middle and end of treatment.

14.0 WITHDRAWAL OF SUBJECTS

Patients will be informed that they are free to withdraw from the study at any time should they so wish without prejudicing their subsequent medical care.

The clinical investigator may remove a patient if, in his/her opinion, it is in the best interest of the patient. A patient may be withdrawn from the study for any of the following reasons:

- Withdrawal of consent - any patient may withdraw from the study at any time
- Deviation from the protocol
- Incidental illness
- An adverse experience

If there is a medical reason for withdrawal, the patient will remain under the supervision of the investigator until in satisfactory health. After receiving the patient's consent, his/her general practitioner will be informed. Every effort will be made to contact patients who fail to attend an appointment to ensure that they are in good health. In any eventuality, the investigator must inform the Medical Department at BPL and record the withdrawal on the CRF.

15.0 ADVERSE EVENTS

An adverse event is defined as any untoward sign, symptom, illness or clinically significant abnormal laboratory value which appears or worsens during the course of the trial and is **temporally** associated with the administration of the test drug. **All adverse events whether or not considered by the Investigator to be related to the study drug(s) must be described and recorded on the appropriate ADVERSE EVENT FORMS in the CRF**. Where possible, a diagnosis should be made.

15.1 Definition of Serious Adverse Events

A **Serious Adverse Event** is any untoward medical occurrence which at any dose:

- results in death
- is life threatening (patient at risk of death at time of event; not hypothetically life-threatening)
- results in persistent or significant disability
- requires or prolongs hospitalisation
- is a congenital anomaly or birth defect

15.2 Detecting Adverse Events

Adverse events should be elicited by careful questioning of the subject at each visit. The Investigator and others responsible for care of the subjects should institute any supplementary investigations of significant adverse events based on the clinical judgment of the likely causative factor. This may include seeking a further opinion from a specialist in the field of the adverse event. The company may suggest special tests based on expert advice. If a serum sample is collected for assay of the test medication or for additional laboratory tests, the Investigator must ensure that the plasma sample is properly labelled and stored.

15.3 Completing Adverse Event Forms

Adverse Event Forms must be completed in a timely manner and contain the following information:

- subject number, initials and date of birth
- description of event (where possible a diagnosis should be made rather than just listing symptoms)
- **relevant** medical history
- study drug, dose and batch number and expiry date
- **all** concurrent medication (comment if suspected cause of event)
- intensity and causality must be assigned following the instructions below:
- action taken (e.g. drug discontinuation) and treatment given
- outcome
- signature of the study physician and date

15.4 Assessment of adverse event intensity

Intensity of adverse events will be assessed by a **nurse or physician**. The following guidelines should be used to assess intensity:

Mild:	Awareness of signs or symptoms that are easily tolerated.
Moderate:	Discomfort enough to cause interference with usual activity.
Severe:	Incapacitating with inability to do usual work.

15.5 Attribution to study drugs

Attribution of adverse effects to study drugs will be assessed by a **physician** according to the following criteria (based on Karch FE, Lasagna L. JAMA 1975; 234: 1236-1241) and recorded on the CRF.

Probable

A reaction that follows a reasonable temporal sequence from administration of the drug, and follows a known response pattern to the suspected drug.

The reaction cannot reasonably be explained by the known characteristics of the subject's clinical state or other modes of therapy administered to the subject.

Possible

Plausible temporal sequence

Follows a known response pattern to the suspected drug

The adverse event might have been produced by the subject's clinical state or other modes of therapy administered to the subject.

Unlikely

The current state of knowledge indicates that a relationship is unlikely

Unknown

It is not possible to assign adverse event to any of the above categories

Not related

In the opinion of the physician the event is unrelated to the study drug. Study drugs are defined as those investigational compounds or their controls used in a study.

In this study, the study drug is: 16% immunoglobulin

15.6 Reporting Adverse Events

The Investigator is responsible for prompt and complete reporting of all adverse events. This facilitates:

- a greater understanding of drug toxicity
- appropriate modification of study protocols

- adherence to regulatory requirements, thus protecting study subjects and prescribing physicians

The Investigator is responsible for complying with his Local Research Ethics Committee's (LREC) policy on adverse event reporting. BPL will inform the MCA in line with regulatory guidelines on adverse events occurring during the trial. The investigator retains the right to inform the MCA if he/she so desires, but must inform BPL so that duplicate reports to the MCA can be highlighted.

WHEN A ADVERSE EVENT OCCURS WHICH FULFILLS THE DEFINITION OF SERIOUS (above) THE INVESTIGATOR MUST IMMEDIATELY:

1. **Telephone or fax available details to the designated CRA or Medical Affairs Manager at BPL**
2. **Complete and sign the adverse event form and send it to the appropriate contacts (see below)**
3. **Inform the LREC of the adverse event where appropriate (i.e. if believed to be study drug related and is unexpected, or if it is a requirement of the adverse event reporting policy of the LREC).**

Mrs Pauline Jackson
Bio Products Laboratory
Dagger Lane
Elstree
Herts WD6 3BX
Telephone: 0181 258 2248
Fax: 0181 258 2611

or

Dr E W Gascoigne
Medical Affairs Manager

Bio Products Laboratory
Elstree
Herts WD6 3BX
Telephone: 0181 258 2622
Fax: 0181 258 2611

Out of hours, BPL Security will contact the Medical Affairs Manager

BPL will inform all Investigators of adverse reactions occurring during the study which would materially affect the safety of study subjects.

15.7 Adverse Event Follow-up

All adverse events will be followed up:

- to resolution

  or
- until an underlying condition has been diagnosed

  or
- until the patient's condition has stabilised

  or
- for a period of 28 days following administration of the study drug

16.0 DATA ANALYSIS

16.1 Statistical Analysis

This is a non-comparative study therefore descriptive statistical techniques will be employed in the analysis of the data. The sample size of 40 evaluable patients has been chosen so that an adequate number of patients receiving both IVIG and SCIG prior to study SCIG can be assessed. This number will also allow for drop-outs and withdrawals. While it would be preferable to have equal numbers in both groups, the study is not reliant upon this. However a minimum number of 10 patients receiving IVIG at baseline and 10 receiving

SCIG will be aimed for when recruiting patients. These subgroups of patients will be analysed separately.

The number, type, severity and duration of adverse events, together with monitoring of haematology and biochemistry screens and viral markers will also be analysed and described.

16.2 Definition of Evaluability

Patients will be considered evaluable and included in the analysis if they satisfy the entry criteria and receive one or more study SCIG infusions.

16.3 Drop-outs

All patients who drop-out or who are withdrawn from the study prior to the first study SCIG infusion will not be considered evaluable for efficacy. The patient's data will however be included in the safety analysis.

All drop-outs and withdrawals must be fully documented and their case record forms submitted to BPL.

16.5 Interim Analysis

If considered necessary by BPL, an interim analysis, using the above statistical tools, will be performed either after at least 10 patients have completed Phase 1 of the study or 20 patients have completed 3 months of SCIG treatment.

17.0 DOCUMENTATION

17.1 Required Pre-Study Documentation

Before the start of the study, BPL will require the following documentation:

- A signed copy of the protocol
- An information leaflet and/or consent form if different from that of the sponsor
- A signed financial agreement
- A signed curriculum vitae for each Investigator and principal co-Investigator
- A copy of laboratory normal ranges for tests required in the protocol
- Evidence of laboratory accreditation and/or performance in relevant National External Quality Assessment Schemes (NEQAS)
- LREC submission, constitution, written approval and composition.
- Signed confidentiality agreement

17.2 Recording data in Case Record Forms

All study data will be recorded on CRFs provided by BPL. These must be completed by the Investigator or a duly authorised assistant. CRFs must be completed in a legible manner using a black ball point pen. Entries made in pencil, colour ink or with a felt tip pen are not acceptable. Entries should be made directly and promptly.

Where data is absent, appropriate abbreviations should be entered instead of leaving blank fields, e.g:

- ND = Not done — should be entered where the required test was not performed. The reason should be stated, e.g. instrument failure and then signed and dated.
- NA = Not Applicable — should be entered where the CRF requires information which is not

appropriate

Where problems arise with blood samples the following abbreviations may be entered:

- H = Haemolysed
- SH = Slightly haemolysed
- GH = Grossly haemolysed
- SS = Short sample. The sample is less than that outlined in the protocol
- BC = Broken container

17.3 Error Correction

- Errors should be corrected by drawing a single black line through the entry without obscuring the original data.
- Corrections should be recorded beside it and should be signed, dated and an explanation given (if necessary).
- Tippex should never be used

17.4 Signing Off and Return of Case Record Forms

The Investigator at each site must sign the completed CRFs to confirm the validity of the data. The Investigator is obliged to return the completed CRFs to BPL at the end of the study. A copy will be retained in the Investigator file.

17.5 Maintenance and Archiving of Study Records

The Investigator must maintain adequate records for the duration of the study.

Before the study begins the following documentation must be present in the Investigator file:

- CRF
- Letter of indemnity (if

- Investigator Brochure
- Signed final protocol and amendments
- Information sheet and consent form for study patients
- CVs for Investigators and co-Investigators
- Financial agreements between
  - Sponsor and Investigator
  - Investigator and other departments for conducting the study, e.g. laboratory/nursing staff/organisation (if applicable)

requested)

- Confidentiality statements
- LREC composition, constitution, submission and letter of approval
- Relevant MREC documentation including submission correspondence and letter of approval
- Normal laboratory values
- Laboratory certification/ accreditation and/or NEQAS performance or validation of analytical methods used in the study.
- Details of transport of test medication and study supplies

During the study the following documents must be added to the file:

- Updates on Investigators brochure (if applicable)
- Amendments (as applicable)
  - to protocols
  - to CRFs
  - information sheets
  - consent forms
- Dated approvals or opinions for any amendments
- Reports to LREC on study progress
- CVs for new Investigators and
- Signed and dated completed CRFs
- Documentation of CRF corrections
- All correspondence concerning adverse event reports between
  - Sponsor and Investigator
  - Investigator and LREC
  - Investigator and MCA (if reported directly)
- Interim or annual reports
- Patient screening log
- Patient ID log
- Sample log
- Site signature log

co-Investigators
- Updates on normal laboratory values
- Updates on technical procedures/tests
- Drug accountability records
- Initiation report
- Final study report (if applicable)
- Correspondence including
  - letters
  - telephone calls
  - meeting notes and minutes
- Signed informed consent forms
- Relevant MREC correspondence

These records must be available for inspection upon reasonable request by the sponsor, members of the regulatory authorities or other authorised individuals.

The Investigator must make proper provision for archiving study documentation at the centre. Patient ID codes must be retained for a period of 15 years after the issue of the final study report. Patient consent forms and other study related documentation must be retained for the maximum period of time permitted by the hospital.

18. STUDY CONDUCT

18.1 Adherence to the Protocol

Deviations from the protocol must not be made without the prior written approval of BPL and the MREC, except where there are logistical or administrative changes, or where they are implemented to eliminate an immediate threat or hazard to the health or safety of the patient. Where a deviation has been made to eliminate an immediate hazard, the Investigator must submit the implemented deviation and the reasons for it to the MREC and must notify relevant members of the Medical Department of BPL. All deviations must be adequately documented in the CRF.

18.2 Protocol amendments

When an amendment is necessary, the principal researcher must comply with the MREC policy on notification of amendments. If the amendment substantially alters the study design or increases the risk to the patients, the principal researcher must do the following:

1. Submit the amendment to the MREC for review and favourable opinion.
2. Notify BPL in writing of the MREC's opinion.
3. Where appropriate revise the consent form and patient information leaflet and have it approved by the MREC.
4. Where the amendment affects the risk/benefit ratio of continued participation for patients already enrolled in the study, informed consent should be obtained using the new information leaflet/consent form.
5. Use the updated version of the information leaflet/consent form for new patients.

Similarly, each Investigator is obliged to inform their LREC of any protocol amendments.

18.3 Early Cessation of the Study

BPL reserves the right to stop the study if:

- Evidence is gained that patients are being exposed to an unacceptable risk
- For any reason, it is not possible to continue to supply the study material
- An advancement in knowledge makes the treatment redundant
- Recruitment is too slow to allow accrual of an adequate number of patients in a reasonable length of time

18.4 Monitoring Visits and Audit

The BPL Clinical Research Associate will monitor the study by telephone, correspondence, and regular visits to the investigative sites. He/she will ensure that:

- the facilities remain adequate
- the Investigator adheres to the protocol and ethical responsibilities
- source documents are legible and agree with entries in the CRF
- adverse events are adequately documented and reported
- study medication is properly stored and drug inventories are being maintained
- samples are identified, handled and stored appropriately

Data entered on the CRFs must be subject to validation. According to Good Clinical Practice, this requires that the study CRA should compare data on the CRF with the raw data, e.g. patient notes and laboratory data.

The study CRA will require access to all patient records to allow verification of the entries in the CRF.

The Investigator will agree to make himself available to correct or discuss any discrepancies.

An audit may also be carried out at the centre to ensure that the study has complied with Good Clinical Practice (GCP).

Access to documentation and facilities used during the study may be required by BPL appointed auditors or by regulatory authorities. In the event that an audit is scheduled by the regulatory authorities, the Investigator must notify BPL immediately.

18.5 Report and Publication

The key authors will be from those centres who have entered patients into the study during the recruitment period. Each centre will be responsible for determining the author from that centre. No more than two authors per centre. The centre that recruits the greatest number of patients will be cited first.

The Investigator is obliged to provide BPL with complete test results and all data and reports within 6 weeks of completion or termination of the study.

Should the Investigator wish to publish the results of the study, a copy of the manuscript will be provided to BPL at least 30 days prior to the expected date of submission to the intended publisher.

18.6 Obligations of the Investigator

The Investigator must conduct the study as outlined in the protocol and in accordance with the ICH guidelines for GCP. Please refer to Appendix I on Investigators Responsibilities at the back of the protocol.

18.7 Confidentiality

Information received pursuant to this study will be regarded as confidential at all times. The Investigator and co-Investigators will be required to sign a confidentiality statement.

19.0 ETHICAL CONSIDERATIONS

The study will be performed in accordance with the Guidelines of the Declaration of Helsinki on biomedical research involving human patients

(South Africa revision 1996) and in accordance with ICH GCP guidelines. Before the study can begin the principal researcher must have received documentary evidence of approval of the study protocol from the MREC. Subsequently, to gain LREC approval, each Investigator must supply the following documentation for review:

- MREC application form
- MREC letter of approval
- Signed MREC response form
- Protocol
- Patient information leaflet and consent form
- Any advertisement for subject recruitment
- The Investigator Brochure
- Investigator's Curriculum Vitae

Once approval has been granted the Investigator is responsible for ensuring that he/she complies with the terms of the approval namely with respect to adverse event reporting, notification of amendments, interim and final reports on the progress of the study.

19.1 Informed Consent

No study-related procedures will be performed prior to the signed consent of the patient/parent/guardian to participate in the study being obtained. Before the decision to participate is made by the patient/parent/guardian, the Investigator or a duly authorised deputy, will provide both an oral and written full explanation of the study. If the Investigator intends to use his own information leaflet, he must ensure that it contains all the information outlined in Appendix 8 (section 1.8.10). After the patient/parent/guardian has been supplied with information and has had sufficient time to review it and ask questions, he/she will be included in the study only if the Investigator is sure that the patient/parent/guardian understands the implications of taking part. His/her agreement to participate will be documented by

the patient/parent/guardian signing and dating a consent form. The Consent Form will be stored at the Investigator's site and a signed copy given to the patient/parent/guardian.

The subject's GP must be informed of his/her participation in the study.

19.2 Compensation/Indemnity

Compensation will be paid by BPL according to the Guidelines drawn up by the Association of the British Pharmaceutical Industry if a patient is injured as a result of being in this study.

Compensation will not be provided for injury or medical conditions that are unrelated to this study.

BPL will indemnify the Institute and the Investigator with respect to any claim for personal injury or death brought against it resulting from the administration to volunteer subject of source materials supplied by BPL, provided the protocol and Investigator agreement have been adhered to, and the event has not been occasioned by malpractice or negligence.

20.0 INVESTIGATOR'S STATEMENT

A multi-centre, open study to assess the safety and efficacy of a 16% immunoglobulin product given via the subcutaneous route in primary antibody deficient patients

I have carefully read this protocol and the Clinical Investigator's Brochure and I confirm that they contain all the information necessary to perform the study. I agree to carry out the study as outlined in this protocol.

Signature: ______________________________

Date: ______________

Name : ______________________________

Centre : ______________________________

23.0 REFERENCES

1. Williams PE, Todd AAM, Yap PL. IVIG Therapy in Primary and Secondary Immunodeficiency. Clinical Applications of Intravenous Immunoglobulin Therapy. Churchill Livingstone 1992: 63-92
2. Berger M, Cupps TR and Fauci AS. Immunoglobulin Replacement Therapy by Slow Subcutaneous Infusion. Ann Intern Med 1980; 93: 55-56
3. Gardulf A, Hammarstrom L, Smith CIE. Home Treatment of Hypogammaglobulinaemia by Rapid Infusion. The Lancet 1992; 338: 162-166
4. Waniewski J, Gardulf A, Hammarstrom L. Bioavailability of γ - Globulin After Subcutaneous Infusions in Patients with Common Variable Immunodeficiency. Journal of Clinical Immunology 1994; vol 14, No 2: 90-97
5. Brennan VM. Subcutaneous Immunoglobulin Therapy: A manual for administration of immunoglobulin by rapid subcutaneous infusion

APPENDIX 1:ADMINISTRATION OF SUBCUTANEOUS INFUSION

Materials Required

- Sterile paper towel
- Medi-swabs
- Needles for drawing up (19G)
- Syringes (10ml)
- Infusion set
- Butterfly needle (28G)
- Tape
- Cotton wool
- Sharps bin
- Syringe drivers (Graseby MS16A)
- Subcutaneous immunoglobulin
- Adrenaline

Assessment of the Patient

- Vital signs should be taken prior to each infusion and an infusion should not be administered if the patient has a fever or is unwell

Method

1. Check syringe drivers and set rate as prescribed
2. Wash hands
3. Clean surface and lay paper towel
4. Check immunoglobulin for correct dose and expiry date
5. Remove vial tops and draw up immunoglobulin with syringe and needle
6. Attach infusion needle to syringe and prime tubing
7. Prepare tape and wipe site (abdomen/thigh) to be used with medi-swab
8. Insert the needle into the abdomen/thigh as instructed and tape firmly in place
9. Check for any blood return by withdrawing the syringe plunger and by removing the syringe from the tubing
10. If blood returns re-site the needle and repeat 9
11. Once position is satisfactory with no blood return, place the syringe on the driver and secure in place
12. Switch on the driver and administer infusion
13. When the alarm sounds remove the needle and dispose of in the sharps bin
14. Repeat process if further dose is needed
15. At the end of the infusion dispose of all used materials, wash hands and record details

APPENDIX 2: ADULT PATIENT INFORMATION SHEET

A MULTI-CENTRE, OPEN STUDY TO ASSESS THE SAFETY AND EFFICACY OF A 16% IMMUNOGLOBULIN PRODUCT GIVEN VIA THE SUBCUTANEOUS ROUTE IN PRIMARY ANTIBODY DEFICIENT PATIENTS

This purpose of this leaflet is to inform you about a research project in which you are invited to take part. If there is anything which you do not understand or if you require further information, please do not hesitate to ask the doctor or the nurse. Please read these notes carefully, before starting the study, and keep them safe so that you may look at them again during the study.

What is the purpose of the study?

You have been asked to take part in a research study to test a specially treated blood product. It is an intramuscular immunoglobulin which may be used subcutaneously to treat primary antibody deficiency by replacing the low levels of antibodies (IgGs) in the blood. At the moment most patients in the UK receive their immunoglobulin therapy intravenously by injecting a dose every few weeks over a number of hours. However, in other countries such as Sweden, the subcutaneous route is routinely used and is especially useful for home therapy or for patients who cannot have their infusions intravenously for medical reasons. The product we are going to use in this study is virtually identical to that used in Sweden. This study has therefore been designed to see whether giving the product subcutaneously can safely maintain adequate blood levels of IgG.

Are there other ways of treating my condition?

Primary antibody deficiency is characterised by low levels of antibodies in the blood which help to fight infection. Therefore, the treatment for this condition usually involves replacing these antibodies in the form of intravenous immunoglobulins. A few patients already receive subcutaneous immunoglobulins by special arrangement with the manufacturers but at the moment there are no licensed subcutaneous preparations available. There are a number of intravenous products available however on the market and you may well be receiving one of these on a regular basis in order to be considered for the study.

Why have I been chosen?

The study will involve at least 40 patients each of whom have the same condition as you (primary antibody deficiency).

Who is organising the study?

The manufacturers of the 16% immunoglobulin that will be used in the study, Bio Products Laboratory (BPL), which is a part of the National Blood Authority, are organising the study. The study as a whole is expected to last about 2 years.

What are the possible benefits of taking part?

Intravenous immunoglobulin is a recognised treatment for primary antibody deficiency so the antibody level in your blood would be expected to rise if you took part in this study. In addition, the information that we get from this study will hopefully, in the future, give doctors more treatment options when treating patients with primary antibody deficiency.

What are the risks of taking part or what if something goes wrong?

Blood sampling may cause some discomfort and bruising. There may be a few extra blood samples taken for the purpose of the study than you would normally have, but this should not cause you any harm.

Some swelling and redness of the injection sites may occur but this should disappear within 24 hours. Serious side effects with subcutaneous immunoglobulin administration rarely occur but may include: injection site hardening, chest pain, shortness of breath, tremor, dizziness, facial swelling, mouth ulcers and joint pain. So far in BPL's experience, only 1 adverse reaction following intramuscular administration has been reported to have occurred in 61,000 doses.

Although all blood donations processed to make the 16% immunoglobulin used are screened to exclude the viruses of hepatitis B, hepatitis C or HIV, there remains the small risk of transmitting virus infection. The product is subjected during manufacturing to a solvent-detergent process which destroys these viruses and to date there has been no record of viral transmission from the product. At the end of the study, your blood would be tested to check that none of the above viruses have been passed on to you during the course of the study. If you do not wish these tests to be carried out then you should not take part in the study.

Bio Products Laboratory, operates to the Guidelines drawn up by the Association of the British Pharmaceutical Industry for subjects in clinical trials and will therefore treat any claim accordingly. A copy of these guidelines is available from your doctor on request. There would be no compensation provided by BPL for injury or medical conditions that are not related to this study.

What would I have to do if I decided to take part in the study?

Before the start of the study, you would be asked about your medical history and be given a complete medical examination. The doctor or nurse would take your blood pressure, pulse rate, temperature and respiration rate. You would also have some blood drawn from a vein in your arm for laboratory testing.

If you qualify for the study, you would receive your normal routine immunoglobulin for up to 3 further infusions. This is so we can collect some baseline information about you. If however, for medical reasons, you need to start the subcutaneous immunoglobulin infusions sooner then your doctor will arrange this for you.

When your routine treatments have finished, you would return to the clinic the following week and weekly thereafter to receive the 16% immunoglobulin subcutaneously. The first dose will be given at 100mg/kg bodyweight. Your doctor would take some blood from you at each visit in order to monitor the immunoglobulin levels. He can then decide to increase or decrease the dose each week in order to maintain a good level of immunoglobulin in your blood.

Before, every hour during and after each infusion your temperature, pulse, blood pressure and respirations would be monitored and the doctor or nurse would observe you for any side effects to the infusion and reaction to the injection sites. The infusion would be injected under the skin in two places at the same time either in your thigh or abdomen, whichever your doctor/nurse decides is more suitable.

These weekly infusions would continue for approximately six months (Phase 1) afterwhich you can decide whether to stay on the treatment until it has been marketed (Phase 2) or return to your previous therapy. If you decide to continue with the study treatment you would carry on with weekly infusions but also be asked to complete a weekly diary card and return to the clinic every 3 months for a check-up.

Routine study blood samples would be taken at the end of the study to confirm the lack of virus transmission.

Would there be any restrictions?

No change in diet is necessary but alcohol should not be taken for at least 12 hours before the first treatment.

Apart from any existing medicines which you are receiving from your doctor, you should avoid taking any medicine other than the study infusion for at least 24 hours prior to and during the study. Please inform the study doctor if you take any other medication during the study including ones that you would buy yourself.

What if I don't want to take part?

As a volunteer, you would be joining the study of your own free will, without any kind of pressure. You can leave the study before it starts or at any time later. This would not affect your medical care now or in the future. If you decide you do not want to stay in the study, you must tell your doctor or the study staff and they will tell you what to do.

The doctor may take you out of the study if it is in your best interest, with or without your consent. If you withdraw or are withdrawn from the study, you would continue to receive other appropriate treatments for your condition. You would be informed in a timely manner by your doctor of any new information which may influence your decision to continue in the study.

Would my records remain confidential?

It would be necessary for authorised staff from the Medical Department at BPL and possibly the Regulatory Authorities, such as the Medicines Control Agency, to go through your hospital notes. However, all information about you in this study would be handled with the strictest confidence. If the results of the study are published for scientific purposes, your name would not appear and no one would know your identity. If you agree to take part your GP will be informed.

Would I get paid for taking part?

You would not be paid to participate in this study. We would, however, pay reasonable travel expenses to and from the study centre as well as other study related expenses you may incur.

Who should I ask if I have any more questions?

Any questions you have about this study can be answered by Dr/Nurse ________________________ who may be reached by telephone on ____________________

If you have any questions about your rights as a research subject or about an injury related to the study you should contact Dr/Nurse __________________ as above.

In an emergency, please contact:

Name: ____________________________

Tel: ____________________________

APPENDIX 3: CONSENT FORM

(This part to be completed by the patient	*(circle one)*
1. Have you read the Patient Information Sheet? (please take a copy home with you to keep)	YES/NO
2. Have you had an opportunity to discuss the study and ask any questions?	YES/NO
3. Have you had satisfactory answers to all of your questions?	YES/NO
4. Have you received enough information about the study?	YES/NO
5. Who has given you an explanation about the study? Dr/Nurse ____________	
6. Do you understand that you are free to withdraw from the study: • At any time? • Without having to give a reason? • Without affecting your future medical care?	YES/NO
7. Sections of your medical notes relating to your participation in the study may be inspected by responsible individuals from BPL or from regulatory authorities. All personal details will be treated as strictly confidential. Do you give permission for these individuals to have access to your records?	YES/NO
8. Has the doctor discussed the circumstances when compensation may be due?	YES/NO
9. Have you had sufficient time to come to your decision?	YES/NO
10. Do you consent to your blood being tested for hepatitis B, C and HIV viruses?	YES/NO
11. Do you agree to take part in the study?	YES/NO

PATIENT *(Please sign below and date your own signature)*

Signed: ____________________ Date: ____________

Print Name: ____________________

INVESTIGATOR

Signed: ____________________ Date: ____________

Print Name: ____________________

APPENDIX 2(i): PAEDIATRIC PATIENT INFORMATION SHEET

A MULTI-CENTRE, OPEN STUDY TO ASSESS THE SAFETY AND EFFICACY OF A 16% IMMUNOGLOBULIN PRODUCT GIVEN VIA THE SUBCUTANEOUS ROUTE IN PRIMARY ANTIBODY DEFICIENT PATIENTS

This purpose of this leaflet is to tell you about a research study in which you can take part in if you wish. If there is anything which you do not understand or if you want some more information, please ask the doctor or the nurse. Please read these notes carefully, before starting the study, and keep them safe so that you may look at them again during the study.

Why is the study being done?

This study will test a specially treated blood product called immunoglobulin. It is normally given into the muscle (intramuscular) or through a vein (intravenous) to replace the low levels of antibodies (IgGs) which happens with your condition. At the moment most patients in Britain receive their treatment through a vein every few weeks over a number of hours. However, in other countries such as Sweden they inject the immunoglobulin under the skin (subcutaneous) which is useful if patients cannot have their infusions through a vein. The product we are going to use in this study is very similar to the one used in Sweden but we want to check that it can be given safely under the skin and also keep a good level of antibodies.

Are there other ways of treating my condition?

With primary antibody deficiency you get low levels of antibodies in the blood which help to fight infection. Therefore, the treatment for this usually involves replacing these antibodies in the form of immunoglobulins through a vein. There are a number of products available on the market and you might already be having one of these when you come to the hospital. A few patients however already receive their immunoglobulin under the skin by special permission .

Why have I been chosen?

The study will involve about 40 patients each of whom have the same condition as you.

Who is organising the study?

The people who make the immunoglobulin that will be used in the study, Bio Products Laboratory (BPL), which is a part of the National Blood Authority, are organising the study. The study as a whole is expected to last about 2 years.

What are the benefits of taking part?

Intravenous immunoglobulin is a common treatment for primary antibody deficiency so the antibody level in your blood should go up if you took part in this study. Also, the results that we get from this study will hopefully give doctors more treatment choices when treating patients like you in the future.

What are the risks of taking part or what if something goes wrong?

Taking blood may cause some discomfort and bruising. There may be a few extra blood samples taken during the study than you would normally have, but this should not cause you any harm.

Some swelling and redness where the skin is injected may happen but this should go within a day. It is very rare for serious side effects to happen but these may include: injection site hardening, pains in your chest, shortness of breath, trembling hands, feeling dizzy, swelling of the face, mouth ulcers and pains in your joints.

All the blood donations that make the product are screened for bugs (viruses) such as hepatitis B, hepatitis C and HIV. The product is specially treated to destroy these bugs and so far noone has caught any of them after using the product. At the end of the study, we would like to test your blood to check that neither of the hepatitis bugs have been given to you during the study. If you do not want these tests to be carried out then you should talk to your doctor about this.

The next bit is complicated but means that if you are harmed because of the study we would try to make up for it:

Bio Products Laboratory, works to the Guidelines drawn up by the Association of the British Pharmaceutical Industry for subjects in clinical trials and will therefore treat any claim accordingly. A copy of these guidelines is available from your doctor on request. There would be no compensation provided by BPL for injury or medical conditions that are not related to this study.

What would I have to do if I wanted to take part in the study?

Before the start of the study, you would be asked about any past illnesses and be examined by the doctor. The doctor or nurse would weigh you, take your blood pressure, temperature and measure your pulse and respiration rate. You would also have some blood taken from a vein in your arm for testing.

If you are able to go into the study, you would carry on with your usual treatment for up to 3 more times. This is so we can collect some baseline information. If you need to start the subcutaneous infusions before the 3 times are up your doctor will tell you.

After your usual treatments have finished, you would come back to the clinic the following week and every week from then on to receive your treatment subcutaneously. Your doctor may want to take some blood from you at some of these visits in order to check on your antibody levels. He/she can then decide what dose you need each time.

While you are having the infusion your temperature, pulse, blood pressure and respirations would be measured and the doctor or nurse would watch you for any side effects. You would have the infusion under the skin in two places at the same time either in your thigh or abdomen, whichever is best for you.

These weekly infusions would continue for approximately six months (Phase 1) afterwhich you can decide whether to stay on the treatment (Phase 2) or return to your previous therapy. If you decide to continue with the study treatment you would carry on with weekly infusions but also be asked to complete a weekly diary card and return to the clinic every 3 months for a check-up.

Blood would be taken from your arm again at the end of the study to make sure, as before, that none of the bugs had been passed to you during the study.

Would there be anything I can't do while I'm in the study?

You should carry on with your medicines that you are taking at the moment. Please let the study doctor know if you need to take any other medicines during the study including ones that your parents/guardians would buy from the shops.

What if I don't want to take part?

You don't have to take part in the study if you don't want to. You can also change your mind at any time before the study starts or at any time later. This would not affect the care you get from your doctor and you would carry on with your usual treatment. If you decide you do not want to stay in the study, you must tell your doctor and he/she will tell you what to do.

The doctor may take you out of the study if he/she thinks it would be better for you. He/she will also tell you if there is any new information which may help you decide whether to carry on with the study or not.

Would my notes remain secret?

People from BPL and possibly the Government will need to have a look at your notes. However, all information about you in this study would remain secret. If the results of the study are printed in a magazine for other doctors to see, your name would not appear and no one would know who you are. If you agree to take part your family doctor (GP) will also be told.

Would I get any money for taking part?

You would not be paid any money to take part in this study. We would, however, pay your fares to and from the hospital.

Who should I ask if I have any more questions?

Any questions you have about this study can be answered by Dr/Nurse ________________ who may be reached by telephone on ________________

In an emergency, please contact:

Name: ____________________________

Tel: ____________________________

APPENDIX 3(i): PAEDIATRIC CONSENT FORM

(This part to be completed by the patient/parent/guardian	*(circle one)*
1. Have you read the Patient Information Sheet?<br>(please take a copy home with you to keep)	YES/NO
2. Have you had a chance to talk about the study and ask any questions?	YES/NO
3. Have you had answers that you understand to all of your questions?	YES/NO
4. Have you received enough information about the study?	YES/NO
5. Who has talked to you about the study?<br>Dr/Nurse ______________	
6. Do you understand that you/your child can withdraw from the study:<br>• At any time?<br>• Without having to give a reason?<br>• Without affecting your future medical care?	YES/NO
7. Some of your medical notes may be looked at by people from BPL or from the Government. All personal details will be kept secret.<br>Are you willing to let these people to look at your notes?	YES/NO
8. Has the doctor told you when compensation may be due?	YES/NO
9. Have you had enough time to make your mind up?	YES/NO
10. Do you agree to your blood being tested for hepatitis B and C viruses?	YES/NO
11. Do you agree to take part in the study?	YES/NO

PATIENT/PARENT/GUARDIAN *(Please sign below and date your own signature)*

Signed: ______________________ Date: ____________

Print Name: ______________ Relationship: ____________
(if applicable)

INVESTIGATOR

Signed: ______________________ Date: ____________

Print Name: ______________________

APPENDIX 4: EXAMPLE GP LETTER

Our ref: SCIG01/BPL

«GPNAME»
«GPADDRESS»

Dear «GPNAME»

Your patient, «PATNAME», has volunteered to take part in an open study to assess the safety and efficacy of a 16% immunoglobulin preparation given via the subcutaneous route in primary antibody deficiency.

Your patient will receive weekly infusions of subcutaneous immunoglobulin at an initial dose of 100mg/kg. The dose will then be tailored to maintain an IgG level of at least 6g/l. The treatment duration will be 6 months but at the end of this period your patient may opt to participate in the follow-on safety phase of the study and continue to receive weekly infusions until such time they decide to withdraw or the product is marketed.

Your patient may be considered suitable to administer their infusions at home after a period of training. If this is the case, the study staff will contact you with the relevant details and seek your agreement before your patient commences home therapy.

Your patient will have undergone a medical examination, including blood tests, before being included in the study. He/she will have close medical monitoring during the infusion and at the end of the study he/she will have another thorough medical.

The trial is being carried out to Good Clinical Practice and has been approved by the Multi Centre Research Ethics Committee (MREC) and the Local Research Ethics Committee (LREC). If there is any information regarding your patient's health which may be relevant to participation in this study, please can you let me know.

If I can be of any further assistance, please do not hesitate to contact me on «INVESTNO»

Yours sincerely,

«INVESTNAME»
«INVESTTITLE»

APPENDIX 5: HOME THERAPY MANUAL

Patient Information

For patients who have primary antibody deficiency, there are several ways of replacing the missing antibodies such as by intramuscular injection or by intravenous infusion. In the past, slow subcutaneous infusions were given but these infusions proved cumbersome and entailed overnight stays in hospital. There is now a new method of replacing the antibodies by rapid subcutaneous infusion which is easy to learn and is advantageous for patients who would be suitable for home therapy.

From recent studies performed in Sweden, immunoglobulin replacement therapy by subcutaneous infusion has proved to be a safe, efficient, time-saving, cost-effective and convenient form of administration. Your doctor thinks that this method would be suitable for you to try. Before you can perform home therapy however you need to satisfy the following criteria:

1. A relative/friend who has also been trained in the techniques must be available to assist with each infusion performed at home

2. You and your relative/friend must be motivated to perform self-infusion

3. After a period of training by the study staff, both you and your relative/friend must be able to manage the infusion techniques

4. Your G.P. must agree to you performing home therapy

5. You must be contactable by telephone.

The training will take place over a period of 8-10 weeks when you come for your study infusions. You and your relative/friend will be shown how to draw the immunoglobulin up from the ampoule into the syringe, insert the butterfly needle under the skin in the thigh or abdomen and connect the syringe to the battery powered syringe driver. The study staff will also show you how to calculate the correct dose and infusion rate .

Materials Required

- Sterile paper towel
- Medi-swabs
- Needles for drawing up (19G)
- Syringes (10ml)
- Infusion set
- Butterfly needle (28G)
- Tape
- Cotton wool
- Sharps bin
- Syringe drivers (Graseby MS16A)
- Subcutaneous immunoglobulin
- Adrenaline

Temperature Record

You must record your temperature on the infusion diary prior to starting the infusion. If this is above the level specified by your study staff ring the clinic and ask for advice before infusing. Also, an infusion should not be administered if you are feverish or are unwell

Method

1. Check syringe drivers and set rate as prescribed
2. Wash hands
3. Clean surface and lay paper towel
4. Check immunoglobulin for correct dose and expiry date
5. Remove vial tops and draw up immunoglobulin with syringe and needle
6. Attach infusion needle to syringe and prime tubing
7. Prepare tape and wipe site (abdomen/thigh) to be used with medi-swab
8. Insert the needle into the abdomen/thigh as instructed and tape firmly in place
9. Check for any blood return by withdrawing the syringe plunger and by removing the syringe from the tubing
10. If blood returns re-site the needle and repeat 9
11. Once position is satisfactory with no blood return, place the syringe on the driver and secure in place
12. Switch on the driver and administer infusion
13. When the alarm sounds remove the needle and dispose of in the sharps bin
14. Repeat process if further dose is needed
15. At the end of the infusion dispose of all used materials, wash hands and record details

APPENDIX 6: HOME THERAPY CONSENT FORM

(This part to be completed by the patient/parent/guardian *(circle one)*

1. Have you read the home therapy information? YES/NO
2. Have you had an opportunity to discuss home therapy and ask any questions? YES/NO
3. Have you had satisfactory answers to all of your questions? YES/NO
4. Have you received enough information about home therapy? YES/NO
5. Who has given you an explanation about home therapy?

   Dr/Nurse ____________________

6. Do you have a relative or friend who is also willing to be trained on home therapy and who will be available to assist you with each home infusion? YES/NO
7. Has your GP been consulted and his agreement for you to commence home therapy been sought? YES/NO
8. Do you agree to be trained to infuse your/your child's subcutaneous immunoglobulin? YES/NO

PATIENT/PARENT/GUARDIAN *(Please sign below and date your own signature)*

Signed: ____________________ Date: ____________

Print Name: ____________________ Relationship: ____________
(if applicable)

RELATIVE/FRIEND *(Please sign below and date your own signature)*

Signed: ____________________ Date: ____________

Print Name: ____________________ Relationship: ____________
(if applicable)

INVESTIGATOR

Signed: ____________________ Date: ____________

Print Name: ____________________

APPENDIX 7: INFUSION RECORD

Maximum temperature for this patient before advice should be sought: [ ][ ].[ ] ^{0}C

If your temperature prior to the infusion is above this figure then you must contact the study staff for advice: Contact Number: ______________________________

Date	Temp ^{0}C	Site*	Dose per Site	Total Dose	Rate mm/hr	Batch No.	Comments/Problems

* A = Abdomen
T = Thigh

APPENDIX 8: LOCAL INVESTIGATOR'S RESPONSIBILITIES BASED ON THE ICH GUIDELINES FOR GOOD CLINICAL PRACTICE

1. LOCAL INVESTIGATOR

1.1 Investigator's Qualifications and Agreements

1.1.1 The Investigator(s) should be qualified by education, training, and experience to assume responsibility for the proper conduct of the study, should meet all the qualifications specified by the applicable regulatory requirement(s), and should provide evidence of such qualifications through up-to-date curriculum vitae and/or other relevant documentation requested by the sponsor, the LREC, and/or the regulatory authority(ies).

1.1.2 The Investigator should be thoroughly familiar with the appropriate use of the investigational product(s), as described in the protocol, in the current Investigator's Brochure, in the product information and in other information sources provided by the sponsor.

1.1.3 The Investigator should be aware of, and should comply with, GCP and the applicable regulatory requirements.

1.1.4 The Investigator should permit monitoring and auditing by the sponsor, and inspection by the appropriate regulatory authority(ies).

1.1.5 The Investigator should maintain a list of appropriately qualified persons to whom the Investigator has delegated significant study-related duties.

1.2 Adequate Resources

1.2.1 The Investigator should be able to demonstrate (e.g. based on retrospective data) a potential for recruiting the required number of suitable subjects within the agreed recruitment period.

1.2.2 The Investigator should have sufficient time to properly conduct and complete the study within the agreed study period.

1.2.3 The Investigator should have available an adequate number of qualified staff and adequate facilities for the foreseen duration of the study to conduct the study properly and safely.

1.2.4 The Investigator should ensure that all persons assisting with the study are adequately informed about the protocol, the investigational product(s), and their study-related duties and functions.

1.3 Medical Care of Study Subjects

1.3.1 A qualified physician (or dentist, when appropriate), who is an Investigator or a sub-Investigator for the study, should be responsible for all study-related medical (or dental) decisions.

1.3.2 During and following a subject's participation in a study, the Investigator should ensure that adequate medical care is provided to a subject for any adverse events, including clinically significant laboratory values, related to the study. The Investigator should inform a subject when medical care is needed for intercurrent illness(es) of which the Investigator becomes aware.

1.3.3 It is recommended that the Investigator inform the subject's primary physician about the subject's participation in the study if the subject has a primary physician and if the subject agrees to the primary physician being informed.

1.3.4 Although a subject is not obliged to give his/her reason(s) for withdrawing prematurely from a study, the Investigator should make a reasonable effort to ascertain the reason(s), while fully respecting the subject's rights.

1.4 Communication with LREC

1.4.1 Before initiating a study, the Investigator should have written and dated approval/favourable opinion from the LREC for the study protocol, written informed consent form, consent form updates, subject recruitment procedures (e.g., advertisements), and any other written information to be provided to subjects.

1.4.2 As part of the Investigator's written application to the LREC, the Investigator should provide the LREC with a current copy of the MREC approval documentation.

1.4.3 During the study the Investigator should provide to the LREC all documents subject to review.

1.5 Compliance with Protocol

1.5.1 The Investigator should conduct the study in compliance with the protocol agreed to by the sponsor and, if required, by the regulatory authority(ies) and which was given approval/favourable opinion by the MREC. The Investigator and the sponsor should sign the protocol, or an alternative contract, to confirm agreement.

1.5.2 The Investigator should not implement any deviation from, or changes of the protocol without agreement by the sponsor and prior review and documented approval/favourable opinion from the MREC of an amendment, except where necessary to eliminate an immediate hazard(s) to study subjects, or when the change(s) involves only logistical or administrative aspects of the study (e.g. change in monitor(s), change of telephone number(s)).

1.5.3 The Investigator, or person designated by the Investigator, should document and explain any deviation from the approved protocol.

1.5.4 The Investigator may implement a deviation from, or a change of, the proto :ol to eliminate an immediate hazard(s) to study subjects without prior MREC approval/favourable opinion. As soon as possible, the implemented deviation or change, the reasons for it, and, if appropriate, the proposed protocol amendment(s) should be submitted.

a) to the MREC for review and approval/favourable opinion,

b) to the sponsor for agreement and, if required,

c) to the regulatory authority(ies).

1.6 Investigational Product(s)

1.6.1 Responsibility for investigational product(s) accountability at the study site(s) rests with the Investigator.

1.6.2 Where allowed/required, the Investigator may/should assign some or all of the Investigator's duties for investigational product(s) accountability at the study site(s) to an appropriate pharmacist or another appropriate individual who is under the supervision of the Investigator.

1.6.3 The Investigator and/or a pharmacist or other appropriate individual, who is designated by the Investigator, should maintain records of the product's delivery to the study site, the inventory at the site, the use by each subject, and the return to the sponsor or alternative disposition of unused product(s). These records should include dates, quantities, batch/serial numbers expiration dates (if applicable), and the unique code numbers assigned to the investigational product(s) and study subjects. Investigators should maintain records that document adequately that the subjects were provided the doses specified by the protocol and reconcile all investigational product(s) received from the sponsor.

1.6.4 The investigational product(s) should be stored as specified by the sponsor and in accordance with applicable regulatory requirement(s).

1.6.5 The Investigator should ensure that the investigational product(s) are used only in accordance with the approved protocol.

1.6.6 The Investigator, or a person designated by the Investigator, should explain the correct use of the investigational product(s) to each subject and should check, at intervals appropriate for the study, that each subject is following the instructions properly.

1.7 Randomisation Procedures and Unblinding

The Investigator should follow the study's randomisation procedures, if any, and should ensure that the code is broken only in accordance with the protocol. If the study is blinded, the Investigator should promptly document and explain to the sponsor any premature unblinding,(e.g., accidental unblinding, unblinding due to a serious adverse event) of the investigational product(s).

1.8 Informed Consent of Study Subjects

1.8.1 In obtaining and documenting informed consent, the Investigator should comply with the applicable regulatory requirement(s), and should adhere to GCP and to the ethical principles that have their origin in the Declaration of Helsinki. Prior to the beginning of the study, the Investigator should have the MREC and LREC's written approval/favourable opinion of the written informed consent form and any other written information to be provided to subjects.

1.8.2 The written informed consent form and any other written information to be provided to subjects should be revised whenever important new information becomes available that may be relevant to the subject's consent. Any revised written informed consent form, and written information should receive the MREC and LREC's approval/favourable opinion in advance of use. The subject or the subject's legally acceptable representative should be informed in a timely manner if new information becomes available that may be relevant to the subject's willingness to continue participation in the study. The communication of this information should be documented.

1.8.3 Neither the Investigator, nor the study staff, should coerce or unduly influence a subject to participate or to continue to participate in a study.

1.8.4 None of the oral and written information concerning the study, including the written informed consent form, should contain any language that causes the subject or the subject's legally acceptable representative to waive or to appear to waive any legal rights, or that releases or appears to release the Investigator, the institution, the sponsor, or their agents from liability for negligence.

1.8.5 The Investigator, or a person designated by the Investigator, should fully inform the subject or, if the subject is unable to provide informed consent, the subject's legally acceptable representative, of all pertinent aspects of the study including the written information given approval/favourable opinion by the MREC and LREC.

1.8.6 The language used in the oral and written information about the study, including the written informed consent form, should be as non-technical as practical and should be understandable to the subject or the subject's legally acceptable representative and the impartial witness, where applicable.

1.8.7 Before informed consent may be obtained, the Investigator, or a person designated by the Investigator, should provide the subject or the subject's legally acceptable representative ample time and opportunity to inquire about details of the study and to decide whether or not to participate in the study. All questions about the study should be answered to the satisfaction of the subject or the subject's legally acceptable representative.

1.8.8 Prior to a subject's participation in the study, the written informed consent form should be signed and personally dated by the subject or by the subject's legally acceptable representative, and by the person who conducted the informed consent discussion.

1.8.9 If a subject is unable to read or if a legally acceptable representative is unable to read, an impartial witness should be present during the entire informed consent discussion. After the written informed consent form and any other written information to be provided to subjects, is read and explained to the subject or the subject's legally acceptable representative, and after the subject's legally acceptable representative has orally consented to the subject's participation in the study and, if capable of doing so, has signed and personally dated the informed consent form, the witness should sign and personally date the consent form. By signing the consent form, the witness attests that the information in the consent form and any other written information was accurately explained to, and apparently understood by, the subject or the subject's legally acceptable representative, and that informed consent was freely given by the subject or the subject's legally acceptable representative.

1.8.10 Both the informed consent discussion and the written informed consent form and any other written information to be provided to subjects should include explanations of the following:

a) That the study involves research.

b) The purpose of the study.

c) The study treatment(s) and the probability for random assignment to each treatment.

d) The study procedures to be followed, including all invasive procedures.

e) The subject's responsibilities.

f) Those aspects of the study that are experimental.

g) The reasonably foreseeable risks or inconveniences to the subject and, when applicable, to an embryo, fetus, or nursing infant.

h) The reasonably expected benefits. When there is no intended clinical benefit to the subject, the subject should be made aware of this.

i) The alternative procedure(s) or course(s) of treatment that may be available to the subject, and their important potential benefits and risks.

j) The compensation and/or treatment available to the subject in the event of study-related injury.

k) The anticipated prorated payment, if any, to the subject for participating in the study.

l) The anticipated expenses, if any, to the subject for participating in the study.

m) That the subject's participation in the study is voluntary and that the subject may refuse to participate or withdraw from the study, at any time, without penalty or loss of benefits to which the subject is otherwise entitled.

n) That the monitor(s), the auditor(s), the MREC and LREC, and the regulatory authority(ies) will be granted direct access to the subject's original medical records for verification of clinical study procedures and/or data, without violating the confidentiality of the subject, to the extent permitted by the applicable laws and regulations and that, by signing a written informed consent form, the subject or the subject's legally acceptable representative is authorising such access.

o) That records identifying the subject will be kept confidential and, to the extent permitted by the applicable laws and/or regulations, will not be made

publicly available. If the results of the study are published, the subject's identity will remain confidential.

p) That the subject or the subject's legally acceptable representative will be informed in a timely manner if information becomes available that may be relevant to the subject's willingness to continue participation in the study.

q) The person(s) to contact for further information regarding the study and the rights of study subjects, and whom to contact in the event of study-related injury.

r) The foreseeable circumstances and/or reasons under which the subject's participation in the study may be terminated.

s) The expected duration of the subject's participation in the study.

t) The approximate number of subjects involved in the study.

1.8.11 Prior to participation in the study, the subject or the subject's legally acceptable representative should receive a copy of the signed and dated written informed consent form and any other written information provided to the subjects. During a subject's participation in the study, the subject or the subject's legally acceptable representative should receive a copy of the signed and dated consent form updates and a copy of any amendments to the written information provided to subjects.

1.8.12 When a clinical study (therapeutic or non-therapeutic) includes subjects who can only be enrolled in the study with the consent of the subject's legally acceptable representative (e.g., minors, or patients with severe dementia), the subject should be informed about the study to the extent compatible with the subject's understanding and, if capable, the subject should sign and personally date the written informed consent.

1.8.13 Except as described in 1.8.14, a non-therapeutic study (i.e. a study in which there is not anticipated direct clinical benefit to the subject), should be conducted in subjects who personally give consent and who sign and date the written informed consent form.

1.8.14 Non-therapeutic studies may be conducted in subjects with consent of a legally acceptable representative provided the following conditions are fulfilled:

a) The objectives of the study can not be met by means of a study in subjects who can give informed consent personally.

b) The foreseeable risks to the subjects are low.

c) The negative impact on the subject's well-being is minimised and low.

d) The study is not prohibited by law.

e) The approval/favourable opinion of the MREC and LREC is expressly sought on the inclusion of such subjects, and the written approval/favourable opinion covers this aspect.

Such studies, unless an exception is justified, should be conducted in patients having a disease or condition for which the investigational product is intended. Subjects in these studies should be particularly closely monitored and should be withdrawn if they appear to be unduly distressed.

1.8.15 In emergency situations, when prior consent of the subject is not possible, the consent of the subject's legally acceptable representative, if present, should be requested. When prior consent of the subject is not possible, and the subject's legally acceptable representative is not available, enrolment of the subject should require measures described in the protocol and/or elsewhere, with documented approval/favourable opinion by the MREC and LREC, to protect the rights, safety and well-being of the subject and to ensure compliance with applicable regulatory requirements. The subject or the subject's legally acceptable representative should be informed about the study as soon as possible and consent to continue and other consent as appropriate (see 1.8.10) should be requested.

1.9 Records and Reports

1.9.1 The Investigator should ensure the accuracy, completeness, legibility, and timeliness of the data reported to the sponsor in the CRFs and in all required reports.

1.9.2 Data reported on the CRF, that are derived from source documents, should be consistent with the source documents or the discrepancies should be explained.

1.9.3 Any change or correction to a CRF should be dated, initialled, and explained (if necessary) and should not obscure the original entry (i.e. an audit study should be maintained): this applies to both written and electronic changes or corrections (see 1.18.4 (n)). Sponsors should provide guidance to Investigators and/or the Investigators' designated representatives on making such corrections. Sponsors should have written procedures to assure that changes or corrections in CRFs made by sponsor's designated representatives are documented, are necessary, and are endorsed by the Investigator. The Investigator should retain records of the changes and corrections.

1.9.4 The Investigator should maintain the study documents as specified in Essential Documents for the Conduct of a Clinical Study and as required by the applicable regulatory requirement(s). The Investigator should take measures to prevent accidental or premature destruction of these documents.

1.9.5 Essential documents should be retained until at least 2 years after the last approval of a marketing application in an ICH region and until there are no pending or contemplated marketing applications in an ICH region or at least 2 years have elapsed since the formal discontinuation of clinical development of the investigational product. These documents should be retained for a longer period however if required by the applicable regulatory requirements or by an agreement with the sponsor. It is the responsibility of the sponsor to inform the Investigator as to when these documents no longer need to be retained.

1.9.6 The financial aspects of the study should be documented in an agreement between the sponsor and the Investigator.

1.9.7 Upon request of the monitor, auditor, MREC, LREC, or regulatory authority, the Investigator should make available for direct access all requested study-related records.

1.10 Progress Reports

1.10.1 The Investigator should submit written summaries of the study status to the LREC annually, or more frequently, if requested by the LREC.

1.10.2 The Investigator should promptly provide written reports to the sponsor, the LREC and, where applicable, the institution on any changes significantly affecting the conduct of the study, and/or increasing the risk to subjects.

1.11 Safety Reporting

1.11.1 All serious adverse events (SAEs) should be reported immediately to the sponsor except for those SAEs that the protocol or other document (e.g., Investigator's Brochure) identifies as not needing immediate reporting. The immediate reports should be followed promptly by detailed, written reports. The immediate and follow-up reports should identify subjects by unique code numbers assigned to the study subjects rather than by the subjects' names, personal identification numbers, and/or addresses. The Investigator should also comply with the applicable regulatory requirement(s) related to the reporting of unexpected serious adverse drug reactions to the regulatory authority(ies) and the LREC.

1.11.2 Adverse events and/or laboratory abnormalities identified in the protocol as critical to safety evaluations should be reported to the sponsor according to the reporting requirements and within the time periods specified by the sponsor in the protocol.
1.11.3 For reported deaths, the Investigator should supply the sponsor and the LREC with any additional requested information (e.g., autopsy reports and terminal medical reports).

1.12 Premature Termination or Suspension of a Study

If the study is prematurely terminated or suspended for any reason, the Investigator should promptly inform the study subjects, should assure appropriate therapy and follow-up for the subjects, and, where required by the applicable regulatory requirement(s), should inform the regulatory authority(ies). In addition:

1.12.1 If the Investigator terminates or suspends a study without prior agreement of the sponsor, the Investigator should inform the institution where applicable, and the Investigator should promptly inform the sponsor and the LREC, and should provide the sponsor and the LREC a detailed written explanation of the termination or suspension.

1.12.2 If the sponsor terminates or suspends a study, the Investigator should promptly inform the institution where applicable and the Investigator should promptly inform the LREC and provide the LREC a detailed written explanation of the termination or suspension.

1.12.3 If the MREC or LREC terminates or suspends its approval/favourable opinion of a study, the Investigator should inform the institution where applicable and the Investigator should promptly notify the sponsor and provide the sponsor with a detailed written explanation of the termination or suspension.

1.13 Final Report(s) by Investigator

Upon completion of the study, the Investigator, where applicable, should inform the institution: the Investigator should provide the LREC with a summary of the study's outcome, and the regulatory authority(ies) with any reports required.

APPENDIX 9: DECLARATION OF HELSINKI

Recommendations guiding physicians in biomedical research involving human subjects

Adopted by the 18th World Medical Assembly, Helsinki, Finland, June 1964 and amended in Tokyo, 1975, in Venice, 1983, in Hong Kong, 1989, ***and in South Africa, October 1996***.

Introduction

It is the mission of the physician to safeguard the health of the people. His or her knowledge and conscience are dedicated to the fulfilment of this mission.

The Declaration of Geneva of the World Medical Association binds the physician with the words, "The health of my patient will be my first consideration", and the International Code of Medical Ethics declares that, "A physician shall act only in the patient's interest when providing medical care which might have the effect of weakening the physical and mental condition of the patient".

The purpose of biomedical research involving human subjects must be to improve diagnostic, therapeutic and prophylactic procedures and the understanding of the aetiology and pathogenesis of disease.

In current medical practice most diagnostic, therapeutic or prophylactic procedures involve hazards. This applies especially to biomedical research.

Medical progress is based on research which ultimately must rest in part on experimentation involving human subjects.

In the field of biomedical research a fundamental distinction must be recognised between medical research in which the aim is essentially diagnostic or therapeutic for a patient, and medical research, the essential object of which is purely scientific and without implying direct diagnostic or therapeutic value to the person subjected to the research.

Special caution must be exercised in the conduct of research which may affect the environment, and the welfare of animals used for research must be respected.

Because it is essential that the results of laboratory experiments be applied to human beings to further scientific knowledge and to help suffering humanity, the World Medical Association has prepared the following recommendations as a guide to every physician in biomedical research involving human subjects. They should be kept under review in the future. It must be stressed that the standards as drafted are only a guide to physicians all over the world. Physicians are not relieved from criminal, civil and ethical responsibilities under the laws of their own countries.

I. Basic principles

1. Biomedical research involving human subjects must conform to generally accepted scientific principles and should be based on adequately performed formed laboratory and animal experimentation and on a thorough knowledge of the scientific literature.

2. The design and performance of each experimental procedure involving human subjects should be clearly formulated in an experimental protocol which should be transmitted for consideration, comment and guidance to a specially appointed committee independent of the investigator and the sponsor provided that this independent committee is in conformity with the laws and regulations of the country in which the research experiment is performed.

3. Biomedical research involving human subjects should be conducted only by scientifically qualified persons and under the supervision of a clinically competent medical person. The responsibility for the human subject must always rest with a medically qualified person and never rest on the subject of the research, even though the subject has given his or her consent.

4. Biomedical research involving human subjects cannot legitimately be carried out unless the importance of the objective is in proportion to the inherent risk to the subject.

11. In the case of legal incompetence, informed consent should be obtained from the legal guardian in accordance with national legislation. Where physical or mental incapacity makes it impossible to obtain informed consent, or when the subject is a minor, permission from the responsible relative replaces that of the subject in accordance with national legislation.

Whenever the minor child is in fact able to give a consent, the minor's consent must be obtained in addition to the consent of the minor's legal guardian.

12. The research protocol should always contain a statement of the ethical considerations involved and should indicate that the principles enunciated in the present Declaration are complied with.

II. Medical research combined with professional care (clinical research)

1. In the treatment of the sick person, the physician must be free to use a new diagnostic and therapeutic measure, if in his or her judgement it offers hope of saving life, re-establishing health or alleviating suffering.

2. The potential benefits, hazards and discomfort of a new method should be weighed against the advantages of the best current diagnostic and therapeutic methods.

3. In any medical study, every patient - including those of a control group, if any - should be assured of the best proven diagnostic and therapeutic method. This does not exclude the use of inert placebo in studies where no proven diagnostic or therapeutic method exists.

4. The refusal of the patient to participate in a study must never interfere with the physician-patient relationship.

5. If the physician considers it essential not to obtain informed consent, the specific reasons for this proposal should be stated in the experimental protocol for transmission to the independent Committee.

6. The physician can combine medical research with professional care, the objective being the acquisition of new medical knowledge, only to the extent that medical research is justified by its potential diagnostic or therapeutic value for the patient.

III. Non therapeutic biomedical research involving human subjects (non-clinical biomedical research)

1. In the purely scientific application of medical research carried out on a human being, it is the duty of the physician to remain the protector of the life and health of that person on whom biomedical research is being carried out.

2. The subjects should be volunteers - either healthy persons or patients for whom the experimental design is not related to the patient's illness.

3. The investigator or the investigating team should discontinue the research if in his/her or their judgement it may, if continued, be harmful to the individual. . In research on man, the interest of science and society should never take precedence over considerations related to the well-being of the subject.

APPENDIX 10: STUDY FLOW CHART

PHASE 1
SAFETY & EFFICACY

PRESTUDY VISIT

Up to 28 days prior to infusion 1

↓ **UP TO 28 DAYS**

INFUSIONS 1-3

1-3 routine doses of current routine IVIG/SCIG. Final dose given 1 week prior to first study SCIG

↓ **1 WEEK**

INFUSIONS 4-30

- First dose of study SCIG given 1 week after last routine IVIG/SCIG.
- Initial dose: 100mg/kg/week.
- Repeated, weekly infusions of study SCIG at 100mg/kg/week or tailored to the patient's trough IgG levels - minimum target 6g/l.
- Option for home therapy after training

6 MONTHS

Selected patients attend clinic daily for 1 week on 2 occasions for **PHARMACOKINETIC EVALUATION** (see later)

OR

END OF STUDY
Complete study and return to previous therapy

ENTER PHASE 2
Remain on study SCIG

PHASE 2
SAFETY

Patient invited to take part in Phase 2 if completed Phase 1

↓

INFUSIONS 31 etc.

(until product marketed or withdraw for other reasons)

- Repeated weekly infusions of study SCIG
- Dose titrated to suit patient - minimum target 6g/l
- Clinic visits at 3 monthly intervals
- Patients complete weekly diary cards

PHARMACOKINETICS

Selected patients attend clinic for pharmacokinetic evaluation

↓

VISITS PK1 a-g & PK 2 a-g

- Daily blood sampling for 1 week post 1st dose of SCIG - visits PK 1 a-g
- Repeated for a further week 3 months later - visits PK 2 a-g

APPENDIX 11: STUDY PROCEDURES

	PHASE 1				PHASE 2	PHARMACOKINETICS	
	**Pre-study**	**Infusions 1-3 Routine IVIG/SCIG**	**Infusions 4-30 SCIG**	**End of Study**	**3 monthly SCIG**	**PK 1 a-g SCIG**	**PK 2 a-g SCIG**
Informed Consent	X						
Inclusion /Exclusion Criteria	X						
Medical History	X						
Physical Examination	X			X			
Vital Signs	X	X	X	X		X	X
Patient Satisfaction Questionnaire	X		$X^{inf\ 18}$	X			
Site Inspection		X	X			X	X
Injection training/check technique			X				
**Haematology**: Haemoglobin, Haematocrit, RBC, WBC, Neutrophils, Lymphocytes, Monocytes, Eosinophils, Basophils, Platelet Count, Haptoglobin, Reticulocytes	X	if clinically indicated	if clinically indicated	X	if clinically indicated	if clinically indicated	if clinically indicated
**Biochemistry**: Sodium, Potassium, Creatinine, LDH, ALT(and/or AST), GGT, Total Bilirubin, Alkaline Phosphatase, Haptoglobin	X	if clinically indicated	if clinically indicated	X	if clinically indicated	if clinically indicated	if clinically indicated
**Blood Group Serology:** ABO (D), Direct Coombs	X						
**Immunology**: IgG, IgA, IgM	X	X	X	X	if clinically indicated	X	X
**Liver Function**: ALT (and/or AST)			X*				
**Specific Antibodies**: Pneumococcus, HIB	X		X*				
**Virology**:HBsAg, HIV adults only(PCR), HCV (PCR)			$X^{**inf\ 4}$	X^{π}	X^{ϕ}		
**Parvovirus**: Parvovirus B19 (PCR)			$X^{\propto}$				
**Archive**: Store at -70^{0}C for 15 years			$X^{inf\ 4}$	X^{π}	X^{ϕ}		
**Repeat Test** : Store at -20^{0}C for repeat testing	X	X	X	X	XΨ	X	X

* 4 weekly samples
** Baseline sample stored at -70^{0}C and tested only if end of study sample positive
Ψ Repeat sample to be taken only if other samples clinically indicated

π Sample to be taken only if patient withdraws from Phase 1 or **not** entering Phase 2
ϕ Sample to be taken if patient withdraws or completes Phase 2
∝ Pre 1st dose &1 week post 1st dose

A MULTI-CENTRE, OPEN STUDY TO ASSESS THE SAFETY AND EFFICACY OF A 16% IMMUNOGLOBULIN PRODUCT GIVEN VIA THE SUBCUTANEOUS ROUTE IN PRIMARY ANTIBODY DEFICIENT PATIENTS

SCIG01

FINAL PROTOCOL

VERSION 2

SEPTEMBER 1999

BIO PRODUCTS LABORATORY
DAGGER LANE
ELSTREE
HERTS WD6 3BX

Tel: 0181 258 2200
Fax: 0181 258 2611

A MULTI-CENTRE, OPEN STUDY TO ASSESS THE SAFETY AND EFFICACY OF A 16% IMMUNOGLOBULIN PRODUCT GIVEN VIA THE SUBCUTANEOUS ROUTE IN PRIMARY ANTIBODY DEFICIENT PATIENTS

SCIG01

FINAL PROTOCOL
VERSION 2

SEPTEMBER 1999

MEDICAL DIRECTOR: Dr Clive H Dash
Bio Products Laboratory
Dagger Lane
Elstree
Herts WD6 3BX
Tel: 0181 258 2565
Fax: 0181 258 2611

CRA: Pauline Jackson
Senior Clinical Research Associate
Tel: 0181 258 2248
Fax: 0181 258 2611

A MULTI-CENTRE, OPEN STUDY TO ASSESS THE SAFETY AND EFFICACY OF A 16% IMMUNOGLOBULIN PRODUCT GIVEN VIA THE SUBCUTANEOUS ROUTE IN PRIMARY ANTIBODY DEFICIENT PATIENTS

SCIG01

FINAL PROTOCOL

VERSION 2

INVESTIGATOR DETAILS

NAME: ______________________ TITLE : ______________________

QUALIFICATIONS: ______________________

ADDRESS: ______________________

____

____

CO-INVESTIGATORS/CO-WORKERS DETAILS

NAME: ______________________ TITLE : ______________________

NAME: ______________________ TITLE : ______________________

NAME: ______________________ TITLE : ______________________

NAME: ______________________ TITLE : ______________________

NAME: ______________________ TITLE : ______________________

TABLE OF CONTENTS

1.0 LIST OF ABBREVIATIONS 1

2.0 SYNOPSIS 2

3.0 INTRODUCTION 4

4.0 STUDY OBJECTIVES 6

5.0 STUDY DESIGN 6

6.0 STUDY POPULATION 6
- 6.1 Source of Subjects 6
- 6.2 Inclusion Criteria 6
- 6.3 Exclusion Criteria 7

7.0 STUDY DRUGS 7
- 7.1 Presentation 7
- 7.2 Dose and route of administration 8
- 7.3 Storage 9
- 7.4 Concurrent Medication 10
- 7.5 Drug Accountability 11

8.0 STUDY SCHEDULE - PHASE 1 11
- 8.1 Pre-study Procedures 11
- 8.2 STUDY PROCEDURES 12
  - 8.2.1 Clinical Monitoring During Infusion 12
  - 8.2.2 Laboratory Measurements 13
  - 8.2.3 Laboratory Sampling in Children 15
- 8.3 End of Study Procedures 15
- 8.4 Blood Sample Collection, Handling and Labelling 16

9.0 HOME THERAPY PROCEDURES 17
- 9.1 Criteria for Home Therapy 17
- 9.2 Home Therapy Training 17
- 9.3 Clinical Monitoring during Home Therapy 18
- 9.4 Blood Sampling during Home Therapy 18

10.0 STUDY SCHEDULE - PHASE 2 19

11.0 STUDY SCHEDULE - PHARMACOKINETICS 20
- 11.1 Pharmacokinetic Procedures 20

12.0 EFFICACY MEASURES 20
- 12.1 Primary Efficacy Measurement 20
- 12.2 Secondary Efficacy Measurements 20

13.0 SAFETY MEASUREMENTS 21

14.0 WITHDRAWAL OF SUBJECTS 21

15.0 ADVERSE EVENTS 22

15.1 Definition of Serious Adverse Events ... 22
15.2 Detecting Adverse Events ... 22
15.3 Completing Adverse Event Forms ... 23
15.4 Assessment of adverse event intensity ... 23
15.5 Attribution to study drugs ... 24
15.6 Reporting Adverse Events ... 25
15.7 Adverse Event Follow-up ... 26

16.0 DATA ANALYSIS ... 26
16.1 Statistical Analysis ... 26
16.2 Definition of Evaluability ... 27
16.3 Dropouts ... 28
16.4 Interim Analysis ... 28

17.0 DOCUMENTATION ... 28
17.1 Required Pre-Study Documentation ... 28
17.2 Recording data in Case Record Forms ... 29
17.3 Error Correction ... 29
17.4 Signing Off and Return of Case Record Forms ... 30
17.5 Maintenance and Archiving of Study Records ... 30

18. STUDY CONDUCT ... 32
18.1 Adherence to the Protocol ... 32
18.2 Protocol amendments ... 32
18.3 Early Cessation of the Study ... 33
18.4 Monitoring Visits and Audit ... 33
18.5 Report and Publication ... 34
18.6 Obligations of the Investigator ... 35
18.7 Confidentiality ... 35

19.0 ETHICAL CONSIDERATIONS ... 35
19.1 CONSENT PROCEDURE FOR ADULTS ... 36
19.2 CONSENT PROCEDURE FOR CHILDREN ... 36
19.3 Compensation/Indemnity ... 37

20.0 INVESTIGATOR'S STATEMENT ... 38

21.0 REFERENCES ... 39

APPENDIX 1: ADMINISTRATION OF SUBCUTANEOUS INFUSION ... 40

APPENDIX 2 ADULT PATIENT INFORMATION LEAFLET ... 41

APPENDIX 3: PARENT/GUARDIAN INFORMATION SHEET ... 44

APPENDIX 4: CHILDREN'S INFORMATION LEAFLET ... 47

APPENDIX 5 : ADULT CONSENT FORM ... 49

APPENDIX 6: CHILDREN'S CONSENT FORM ... 50

APPENDIX 7: PATIENT INFORMATION LEAFLET FOR EXTRA BLOOD TESTS (PHARMACOKINETICS) ... 51

APPENDIX 8: PHARMACOKINETICS CONSENT FORM ... 52

APPENDIX 9: EXAMPLE GP LETTER 53
APPENDIX 10: HOME THERAPY MANUAL 54
APPENDIX 11: HOME THERAPY CONSENT FORM 56
APPENDIX 12: INFUSION RECORD 57
APPENDIX 13: LOCAL INVESTIGATOR'S RESPONSIBILITIES BASED ON THE ICH GUIDELINES FOR GOOD CLINICAL PRACTICE 58
APPENDIX 14: DECLARATION OF HELSINKI 70
APPENDIX 15: STUDY FLOW CHART 75
APPENDIX 16: STUDY PROCEDURES 77

1.0 LIST OF ABBREVIATIONS

AE	Adverse Event
ADR	Adverse Reaction
ALT	Alanine aminotransferase
AST	Aspartate aminotransferase
BPL	Bio Products Laboratory
CPMP	Committee on Proprietary Medicinal Products
CRF	Case Record Form
CRA	Clinical Research Associate
CTX	Clinical Trial Exemption
CV	Curriculum Vitae
EC	Ethics Committee
GCP	Good Clinical Practice
GGT	Gamma glutamyl transferase
GP	General Practitioner
HbsAg	Hepatitis B surface antigen
HCV	Hepatitis C virus
HIB	Haemophilus Influenzae type B
HIV	Human Immunodeficiency Virus
IgA	Immunoglobulin A
IgG	Immunoglobulin G
IgM	Immunoglobulin M
iu	International Units
ICH	International Conference on Harmonisation
IVIG	Intravenous immunoglobulin
LDH	Lactate dehydrogenase
LREC	Local Research Ethics Committee
MCA	Medicines Control Agency
MREC	Multicentre Research Ethics Committee
PCR	Polymerase Chain Reaction
RBC	Red Blood Cell
SAE	Serious Adverse Event
SCIG	Subcutaneous Immunoglobulin
SOP	Standard Operating Procedure
WBC	White Blood Cell

2.0 SYNOPSIS

A multi-centre, open study to assess the safety and efficacy of a 16% immunoglobulin product given via the subcutaneous route in primary antibody deficient patients.

Up to 40 adult and paediatric patients, aged 0 - no upper age limit, stable on intravenous immunoglobulin therapy (IVIG) or subcutaneous immunoglobulin therapy (SCIG), will be enrolled.

Patients will be eligible to enter the study if, in the investigator's opinion, their disease is stable. In order to collect baseline data, patients will continue to receive their usual immunoglobulin, whether IVIG or SCIG, for 3 infusions before commencing the study subcutaneous immunoglobulin. Starting one week after their final treatment they will receive the 16% immunoglobulin product subcutaneously at a dose of 100mg/kg bodyweight. Weekly infusions will then continue to be administered for a period of 6 months. The dose and frequency of the 16% immunoglobulin product given, in order to maintain an adequate serum IgG level, will be tailored to suit the patient. This will be decided by the investigator, but a minimum target IgG trough level of 6g/L will be aimed for.

There will be provision for patients to administer SCIG at home, if appropriate, after a period of training. These patients will be required to complete diary cards during each home infusion and to attend the clinic at 1-4 weekly intervals for blood sampling.

After 6 months of SCIG treatment (Phase 1), patients will then either return to their usual immunoglobulin treatment or continue with the 16% immunoglobulin in Phase 2 of the study which is a safety follow-on phase.

During Phase 2, patients will be asked to complete regular diary cards and return for clinic visits at 3 monthly intervals.

Patients will attend the clinic at the following time-points:-

STUDY PHASE	VISIT/INFUSION NUMBER	VISIT DETAILS
**Phase 1** **(CRF 1)**	Prestudy	Up to 28 days prior to study entry
	Infusions 1-3	3 infusions of patient's routine immunoglobulin at usual frequency and dose prior to study SCIG to collect baseline data
**Phase 1** **(CRF 2)**	Infusions 4 -30	Selected patients will be asked to attend daily clinic visits after first study SCIG infusion for 1 week and repeated after 3 months SCIG
**Pharmacokinetics**	PK 1 a-g (optional)<br>PK 2 a-g (optional)	Selected patients will be asked to attend daily clinic visits after first study SCIG infusion for 1 week and repeated after 3 months SCIG
**Phase 2** **(CRF 3)**	Infusions 31 etc<br>(until product is marketed)	Patients will be invited to take part in the safety follow-on phase. They will receive weekly SCIG infusions, complete regular diary cards and attend the clinic at 3 monthly intervals.

Primary Efficacy Measurement:

- The proportion of trough levels at each time point where the IgG $\geq$ 4g/L

Secondary Efficacy Measurements:

- The change in SCIG dose required in order to maintain trough levels at a minimum of 6g/L. The number of occasions when the dose is adjusted, the size of the dose change and the overall range of dose changes will be calculated.

- The proportion of trough levels at each time point where the IgG $\geq$ 6g/L.
- The time taken for each patient to reach a steady state IgG trough level. This will be defined as when three consecutive occasions occur when the IgG trough levels are within 1g/L of each other.
- The mean change in IgG trough level as compared to the baseline level at each time point.
- The overall number of infections occuring and days off work/school.

Safety Measurements:

- Number, type, severity and duration of adverse events occurring throughout the study.
- Laboratory monitoring of haematology, biochemistry and immunological and viral markers
- Monitoring of vital signs

Patient/parent/guardian satisfaction of the subcutaneous route will be assessed by way of a short questionnaire at the beginning, middle and end of treatment.

3.0 INTRODUCTION

Patients who are diagnosed with primary antibody deficiency usually require regular immunoglobulin replacement therapy in order to avoid serious infection. The efficacy of life-long intravenous immunoglobulin (IVIG) therapy is well established and is a major contributor to improved health and quality of life for these patients[1]. However, IVIG treatment for most patients requires regular visits to hospital where the infusion can take many hours to administer depending on the dose and tolerance to the treatment.

Intramuscular and slow subcutaneous infusions of immunoglobulins are alternatives to IVIG but are not without limitations. Intramuscular injections are often painful, the dose that can be safely delivered is limited and the side-effects can be severe. Slow subcutaneous infusions, on the other hand, while

benefiting from a better safety profile, are time-consuming and cumbersome usually requiring overnight infusions[2].

Rapid subcutaneous infusions of immunoglobulin preparations intended for intramuscular use were first described in 1991 by Gardulf et al[3]. Patients were given simultaneous infusions totaling 34-40ml/hr via dual portable pumps. In addition to there being very few (0.93%) mild systemic reactions experienced with this method of administration, patients were also able to infuse the immunoglobulin at home after a period of training.

BPL's Human Normal Immunoglobulin is mainly IgG and is indicated for prophylaxis against hepatitis A infection and replacement therapy in primary antibody deficiency. It is manufactured from venous plasma and is currently licensed for intramuscular use. This study will use an almost identical product, obtained from the plasma of screened donors in the United States. However, the composition of this 16% immunoglobulin product is such that it would also be suitable for subcutaneous use since it is virtually identical to the product used routinely in Scandinavia[3,4], Kabiglobulin. Both products are approximately 16% immunoglobulin, mercury-free and have a very low IgA content which contributes to their subcutaneous tolerance. In addition, they have a solvent/detergent step included in the manufacturing process which inactivates lipid-enveloped viruses such as HIV, hepatitis C (HCV) and hepatitis B (HBV). To date there have been no reports of these viruses being transmitted by BPL's intramuscular product.

The following study has therefore been designed to assess the efficacy and safety of giving a 16% immunoglobulin product via rapid subcutaneous infusion to patients with primary antibody deficiency.

4.0 STUDY OBJECTIVES

1. To determine the efficacy of giving patients with primary antibody deficiency, weekly subcutaneous infusions of a 16% immunoglobulin product at a dose tailored to achieve a minimum trough serum IgG level of 6g/L.

5.0 STUDY DESIGN

A multi-centre, open, safety and efficacy study of weekly subcutaneous 16% immunoglobulin.

6.0 STUDY POPULATION

6.1 Source of Subjects

The trial will be conducted as a multi-centre study. Up to a total of 40 adult and paediatric patients with a diagnosis of primary antibody deficiency will be enrolled. This number will allow for drop-outs and withdrawals. Patients can either be receiving IVIG or SCIG as their normal replacement therapy before entry into the study and a minimum number of 10 of each type will be aimed for in order for an adequate number to be analysed.

6.2 Inclusion Criteria

a) Diagnosis of primary antibody deficiency syndrome

b) Aged 0 - no upper age limit

c) Stable disease and has been receiving immunoglobulin (IVIG or SCIG) for the past 6 months

d) Written informed consent (patient/parent/guardian).

6.3 Exclusion Criteria

a) Known to be intolerant to IgA

b) Pregnant women or women who are breast feeding

c) History of clinically significant renal or hepatic disease or known renal or hepatic abnormalities

d) Unsuitable for the purposes of the study (in the opinion of the investigator)

e) Participation in another clinical trial within the last 30 days

f) History of allergic reactions to intravenous blood products

g) History of infection within the last 2 months, requiring IV antibiotics

h) At the beginning of the study, is known to require treatment with another blood product during the course of the study.

7.0 STUDY DRUGS

7.1 Presentation

The immunoglobulin used in this study is a liquid preparation containing 16% immunoglobulin, mainly immunoglobulin G (IgG, gammaglobulin).

The product is presented in a 5ml vial containing 750mg of protein. If available, 10ml vials containing 1500mg of protein will also be used.

1ml of solution contains the following approximate amounts:

Human protein (at least 95% IgG) 140-180mg/ml

Sodium Chloride	9mg/ml
Glycine	6mg/ml
Sodium Acetate	2mg/ml

The product is stored in containers which are drawn vials of neutral borosilicate glass with a 13mm neck. The closure is a 13mm diameter overseal, consisting of a snap-off polypropylene cap, a clear lacquered aluminium skirt and a halobutyl rubber wad.

7.2 Dose and route of administration

Patients enrolled will continue to receive their usual immunoglobulin infusion at the same dose and frequency for 3 treatments prior to receiving study SCIG in order for baseline data to be collected. However, where patients are currently receiving IVIG and are needing to change rapidly to SCIG due to poor venous access, a minimum of 1 baseline IVIG treatment would be acceptable. If patients are currently receiving SCIG they will not be required to undergo any IVIG treatments and can commence SCIG with the study product once they have completed three subcutaneous infusions of their usual immunoglobulin.

Once study product is commenced, the 16% immunoglobulin will be given by subcutaneous infusion via a portable syringe driver (Graseby, MS16A) at an initial starting dose of 100mg/kg bodyweight. Subsequent SCIG infusions will continue to be administered at weekly intervals for a period of 6 months and the dose will be tailored to suit the patient's trough levels of IgG. The dose and frequency of the 16% immunoglobulin product given, in order to maintain an adequate serum IgG level, will be decided by the investigator but a minimum target IgG trough level of 6g/L will be aimed for.

The 16% immunoglobulin product should be given by separate infusion and not mixed with other infusion material.

For an adult, the first SCIG infusion should be given at a slow rate of 10mls/hr increasing over the next 4-8 infusions by 1-2mls/hr until the recommended maximum rate of 20mls/hr is reached. Two sites may be simultaneously used at this rate, via 2 syringe drivers, giving a combined maximum rate of 40mls/hr.

For a child, the first infusion should be given at a slow rate of 5mls/hr increasing by 0.5-1mls/hr over the next 4-8 infusions until the recommended maximum rate of 10mls/hr is reached. Two sites may be simultaneously used at this rate, via 2 syringe drivers, giving a combined maximum rate of 20mls/hr.

Either the abdomen or the thigh can be used as the site of infusion for both adults and children. Most patients seem to prefer abdominal siting of the infusions although very young children tolerate the infusions better in the thigh than in the abdomen.

Instructions for administration of subcutaneous immunoglobulin can be found in Appendix 1.

7.3 Storage

The 16% immunoglobulin product should be stored in its carton in the dark and has a shelf life of 2 years if stored between 2-8°C or 1 week if stored at 25°C. DO NOT FREEZE.

The 16% immunoglobulin product is for single use only; any used materials and unused solution should be discarded by approved means.

The condition of date-expired or incorrectly stored product cannot be guaranteed. Such product may be unsafe and should not be used. Solutions which are cloudy or have deposits should not be used.

The dose volume for each vial size is specified on the label.

The 16% immunoglobulin product for clinical trial use should be stored separately from routine product, as product reconciliation has to be done in accordance with the ICH guidelines for GCP.

7.4 Concurrent Medication

The 16% immunoglobulin product is believed not to affect the immune response to bacterial vaccines but could reduce the response to some virus vaccines and toxoids. However it should be noted that attempts to vaccinate patients with primary antibody deficiency may not be completely effective even if immunoglobulin has not been administered.

With administration of the 16% immunoglobulin product, a broad spectrum of antibodies is passively administered. These antibodies will interfere with the response to live vaccines, especially the MMR (measles, mumps, rubella) vaccine and varicella vaccine. Such vaccines should therefore be given at least 3 weeks before or 3 months after administration of immunoglobulin. This does not apply to yellow fever vaccine however since the immunoglobulin product obtained from US plasma is unlikely to contain antibody to this virus.

All medications taken by the patient or administered to the patient during the study will be recorded as concurrent medication. In addition, any drugs prescribed by the investigator or the patient's G.P. throughout the trial will also be regarded as concurrent medication.

The following information on all concurrent medication must be recorded in the CRF:-

- the name of the drug and its pharmaceutical form
- the reason for treatment
- the dose
- the duration of treatment

7.5 Drug Accountability

It is essential that accurate drug accountability records are maintained to ensure that the total number of 16% immunoglobulin product vials dispatched, received and returned by a study site can be established.

This is facilitated by completion of dispatch notes (by BPL), receipt forms, stock control logs, dispensing/returns logs and returns forms (by Pharmacy or equivalent) and the recording at each infusion of the batch number and number of grams and vials given in the patient's notes and CRF (investigator or appointed personnel).

The 16% immunoglobulin product should be used solely as indicated in the protocol and must not be used on patients not in the trial without the prior agreement of BPL.

8.0 STUDY SCHEDULE - PHASE 1

8.1 Pre-study Procedures

Prior to enrolling a patient, the investigator should ensure that the patient fulfills the inclusion/exclusion criteria.

Potential subjects for the study, or their parent/guardian, will be given a detailed, oral presentation of the nature, purpose, risks and requirements of the study, in addition to receiving detailed information. They will be given adequate time to consider participating in the study and the opportunity to ask the study physician about any aspect of the study. Once satisfied, the patient/parent/guardian will be asked to sign a Consent Form. A letter will be sent to the patient's G.P.

Subsequently, the patient will be allocated a Subject Number, provided by BPL, for identification purposes and then undergo screening, which

will take place no more than 28 days prior to Infusion 1. The pre-study assessment will consist of:

- Medical history
- Physical examination including height, weight, sitting blood pressure, pulse, temperature and respiration
- Patient Satisfaction Questionnaire
- Blood samples as detailed in the following table:

**Haematology**	Haemoglobin, Haematocrit, RBC, WBC, Neutrophils, Lymphocytes, Monocytes, Eosinophils, Basophils, Platelet Count, Reticulocytes
**Blood Group Serology**	ABO (D), Direct Coombs' Test
**Biochemistry**	Sodium, Potassium, Creatinine, LDH, ALT (and/or AST), GGT, Total Bilirubin, Alkaline Phosphatase, Haptoglobin
**Immunology**	IgG, IgA, IgM
**Specific Antibodies**	Pneumococcus, HIB
**Repeat Test - Stored**	Separate serum aliquot to be stored at -20°C in case of repeat testing (5ml clotted)

8.2 STUDY PROCEDURES

8.2.1 Clinical Monitoring During Infusion

Vital signs (body temperature, respiration, pulse and sitting blood pressure) and adverse events since the last infusion, will be recorded prior to each infusion, at hourly intervals during the infusion and again at the end. The infusion site(s) will be inspected during and at the end of the infusion for signs of irritation. Any untoward irritation and/or swelling will be recorded as an adverse event in the CRF. The

patient/parent/guardian will also be asked to complete a Patient Satisfaction Questionnaire at approximately week 12.

8.2.2 Laboratory Measurements

The following immunology samples will be taken immediately prior to each infusion throughout Phase 1. In addition, for patients on IVIG, a post-infusion sample will be taken immediately after the last routine treatment:

**Immunology**	IgG, IgA, IgM

Once the patient has completed all routine treatments, the following baseline samples will be taken before any study 16% subcutaneous immunoglobulin product is administered:

**Parvovirus**<br>Parvovirus B19 (PCR)	5ml clotted
**Virology**<br>Baseline samples of HBsAg, HIV (PCR) for adults and HCV (PCR) will be stored at -70°C and tested at the end of the study if the patient is found to be positive	5ml clotted
**Archive**<br>Sample to be stored at -70°C for 15 years	5ml clotted

One week after the first study SCIG infusion has been given, the following sample for parvovirus will be taken:

**Parvovirus**<br>Parvovirus B19 (PCR)	5ml clotted

The following laboratory measurement will be taken at 4 weekly intervals throughout Phase 1:

**Liver Function**	ALT (and/or AST)
**Specific Antibodies**	Pneumococcus, HIB

The following laboratory measurements will only be assessed if clinically indicated throughout Phase 1:

**Haematology**	Haemoglobin, Haematocrit, RBC, WBC, Neutrophils, Lymphocytes, Monocytes, Eosinophils, Basophils, Platelet Count, Reticulocytes
**Biochemistry**	Sodium, Potassium, Creatinine, LDH, ALT (and/or AST), GGT, Total Bilirubin, Alkaline Phosphatase, Haptoglobin

At each timepoint, a repeat sample will be taken in case of laboratory error or loss of sample. In the case of clinically indicated samples, only when a sample is required to be taken will a repeat test sample also be taken at the same time:

**Repeat Test - Stored** Separate serum aliquot to be stored at -20°C in case of repeat testing	5ml clotted

During the study, any abnormal laboratory values that are considered to be clinically significant by the investigator must be repeated, using the stored sample, as soon as possible to rule out laboratory error.

Values outside the normal range for the laboratory, even those which are not considered to be clinically significant by the investigator must be commented on, on the laboratory report.

All adverse events will be recorded in the CRF.

Patients considered suitable for home therapy will follow the schedule laid out in section 9.

8.2.3 Laboratory Sampling in Children

Laboratory sampling procedures defined by the protocol must be adhered to in the case of all adult patients over the age of 18 years. Where children are involved, all baseline samples should be obtained wherever possible, in particular, immunology and virology (except HIV testing will not be performed in children). Thereafter, as many subsequent blood samples as the individual is prepared to undergo or if sampling is clinically indicated should be collected in order to obtain adequate safety and efficacy data. End of study virology samples (except HIV) must also be collected to exclude viral transmission.

8.3 End of Study Procedures

The following assessments will be performed on all patients at the end of Phase 1 one week following the final study SCIG:

- Physical examination including weight, sitting blood pressure, pulse, temperature and respiration
- Patient Satisfaction Questionnaire
- Blood samples as follows:

**Haematology**	Haemoglobin, Haematocrit, RBC, WBC, Neutrophils, Lymphocytes, Monocytes, Eosinophils, Basophils, Platelet Count, Reticulocytes
**Biochemistry**	Sodium, Potassium, Creatinine, LDH, ALT (and/or AST), GGT, Total Bilirubin, Alkaline Phosphatase, Haptoglobin
**Immunology**	IgG, IgA, IgM
**Virology**<br>(if not entering Phase 2)	HBsAg, HIV (PCR), HCV (PCR)<br>(5ml clotted)
**Archive - Stored**<br>(if not entering Phase 2)<br>Plasma sample to be stored at -	5ml clotted

70°C for 15 years	
**Repeat Test - Stored** Separate serum aliquot to be stored at -20°C in case of repeat testing	5ml clotted

8.4 Blood Sample Collection, Handling and Labelling

Blood samples for haematology and biochemistry will be tested locally. The labelling, handling and testing of samples will be performed according to local procedures. Each blood sample must have the following minimum information:

- Study Number (SCIG01)
- Subject Number
- Date and time of collection

Virology and Parvovirus PCR will be performed by Central Public Health Laboratories, Colindale, London. Samples will be sent by courier to arrive by 10:00am on the day following collection. For Parvovirus samples, the serum will be separated from clot within 2 hours of clot formation.

A 5ml aliquot of serum will be stored at -70°C as a baseline sample immediately prior to the first SCIG infusion at infusion 4 and again at the end of Phase 1. These must be clearly identified as above and kept at the centre for a minimum of 15 years from the time that the study is completed.

A separate 5ml aliquot of serum will be stored at the centre at -20°C or below, each time a study sample is obtained, in case of repeat sampling.

9.0 HOME THERAPY PROCEDURES

9.1 Criteria for Home Therapy

Self-infusion of SCIG will only be offered to suitable patients. The following criteria must be satisfied before a patient is to be considered eligible for home therapy:-

1. In the opinion of the investigator, the patient/parent/guardian must be motivated to perform the infusion.
2. After a period of training by the study staff, the patient/parent/guardian's ability to manage the infusion techniques must be satisfactory.
3. The patient's G.P. must agree to the patient performing home therapy.
4. A relative/friend who has also been trained in the techniques must be available to assist with each infusion performed at home.
5. The patient/parent/guardian must be contactable by telephone.

9.2 Home Therapy Training

A period of training will take place over 8-12 weekly infusions. The patient/parent/guardian will be trained to draw up the 16% immunoglobulin into a syringe, prime the tubing and insert the needle correctly, maintaining an aseptic technique throughout the procedure. The patient/parent/guardian will be instructed on how to use a syringe driver and also how to recognise adverse reactions and the appropriate action to take. Once the patient/parent/guardian is confident and proficient in all aspects of home therapy and when the investigator is satisfied that the patient/parent/guardian has reached the required standard to safely administer SCIG, they will be provided with a Home Therapy Manual (Appendix 5) and asked to sign a Home Therapy Consent Form (Appendix 6).

The criteria in section 9.1 must be satisfied before patient's can receive SCIG at home. In addition, the patient's G.P. having already agreed in principle, must also be informed when the patient commences home therapy. This must be documented in the patient's notes.

9.3 Clinical Monitoring during Home Therapy

Prior to each infusion, the patient/parent/guardian will record the temperature and detail the result on the diary card provided. If the patient's temperature is above the level pre-specified by the investigator and/or the patient is feverish, the SCIG should be delayed until the patient/parent/guardian has sought advice from the investigator using the contact number provided.

The following details will be routinely recorded on the Infusion Diary Card (Appendix 7):

- Infusion date
- Temperature (°C)
- Site(s) used (A=abdomen, T= thigh)
- Dose per site (mg)
- Total dose (mg)
- Rate (mm/hr)
- Batch number
- Comments/Adverse reactions

9.4 Blood Sampling during Home Therapy

If practical, the patient should return to the clinic on a weekly basis for the same blood samples as patients receiving their infusions in hospital. However, since home therapy is intended to provide the patient with a more flexible regimen, blood samples taken every 4 weeks should be scheduled as a minimum. If it is clinically indicated or if local

procedures require however, the patient will be asked to return for more frequent blood sampling. This will be discussed and agreed with the patient on an individual basis.

Therefore, for the purposes of the study, the following minimum blood samples should be obtained from adult patients on home therapy:

Timepoints	Blood Tests
Pre-study	All tests detailed in section 8.1
Infusions 1-3<br>IVIG (if applicable)	All tests detailed in section 8.2.2
Infusion 4<br>SCIG	All tests detailed in section 8.2.2
Infusions 8, 12, 16, 20, 24, 28<br>(i.e. every 4 weeks)	All tests detailed in section 8.2.2
End of Study	All tests detailed in section 8.3

If the patient attends the clinic for an unscheduled visit, additional blood samples for immunology, haematology and biochemistry should also be taken as clinically indicated as well as a repeat test sample.

10.0 STUDY SCHEDULE - PHASE 2

At the end of Phase 1, patients will be invited to enter the safety follow-on study (Phase 2) and continue receiving subcutaneous 16% immunoglobulin either in hospital or at home until such time that they wish to discontinue treatment or the product is available on the market. Patients will be asked to attend study visits at the clinic at 3 monthly intervals throughout Phase 2 when they will be asked whether they have experienced any adverse events since the last visit. If clinically indicated, blood sample for haematology, biochemistry and/or immunology will also be taken. If the patient decides to withdraw from Phase 2 before the product is marketed, a sample for virology and an archive blood sample will be taken to exclude viral transmission during Phase 2.

11.0 STUDY SCHEDULE - PHARMACOKINETICS

It is expected that only a handful of patients (maximum 12) will take part in the pharmacokinetic assessment and that these will comprise of adult patients only, living near to the centres involved. Patients who agree to take part will be asked to return to the clinic every week day for 1 week after the first study SCIG has been infused. This will be repeated for a further week, 3 months later.

11.1 Pharmacokinetic Procedures

Vital signs (body temperature, respiration, pulse and sitting blood pressure) and adverse events will be recorded at each visit. The following laboratory samples will be collected on each day that the patient attends for the pharmacokinetic part of the study:

**Immunology**	IgG, IgA, IgM
**Repeat Test - Stored**<br>Separate serum aliquot to be stored at -20°C in case of repeat testing	5ml clotted

If clinically indicated, blood samples for haematology and biochemistry will also be taken.

12.0 EFFICACY MEASURES

12.1 Primary Efficacy Measurement

- The proportion of trough levels at each time point where the IgG $\geq$ 4g/L

12.2 Secondary Efficacy Measurements

- The change in SCIG dose required in order to maintain trough levels at a minimum of 6g/L. The number of occasions when the dose adjusted, the size of the dose change and the overall range of dose changes will be calculated.

- The proportion of trough levels at each time point where the IgG ≥ 6g/L
- The time taken for each patient to reach a steady state IgG trough level. This will defined as when three consecutive occasions occur when the IgG trough levels are within 1g/L of each other.
- The mean change in IgG trough level as compared to the baseline level at each time point.
- The overall number of infections occurring and days off work/school

13.0 SAFETY MEASUREMENTS

- Number, type, severity and duration of adverse events occurring throughout the study.
- Laboratory monitoring of haematology, biochemistry and immunological and viral markers
- Monitoring of vital signs

Patient/parent/guardian's satisfaction of the subcutaneous route will be assessed by way of a short questionnaire at the beginning, middle and end of treatment.

14.0 WITHDRAWAL OF SUBJECTS

Patients will be informed that they are free to withdraw from the study at any time should they so wish without prejudicing their subsequent medical care.

The clinical investigator may remove a patient if, in his/her opinion, it is in the best interest of the patient. A patient may be withdrawn from the study for any of the following reasons:

- Withdrawal of consent - any patient may withdraw from the study at any time
- Deviation from the protocol
- Incidental illness
- An adverse experience

If there is a medical reason for withdrawal, the patient will remain under the supervision of the investigator until in satisfactory health. After receiving the patient's consent, his/her general practitioner will be informed. Every effort will be made to contact patients who fail to attend an appointment to ensure that they are in good health. In any eventuality, the investigator must inform the Medical Department at BPL and record the withdrawal on the CRF.

15.0 ADVERSE EVENTS

An adverse event is defined as any untoward sign, symptom, illness or clinically significant abnormal laboratory value which appears or worsens during the course of the trial and is **temporally** associated with the administration of the test drug. **All adverse events whether or not considered by the Investigator to be related to the study drug(s) must be described and recorded on the appropriate ADVERSE EVENT FORMS in the CRF**. Where possible, a diagnosis should be made.

15.1 Definition of Serious Adverse Events

A **Serious Adverse Event** is any untoward medical occurrence which at any dose:

- results in death
- is life threatening (patient at risk of death at time of event; not hypothetically life-threatening)
- results in persistent or significant disability
- requires or prolongs hospitalisation
- is a congenital anomaly or birth defect

15.2 Detecting Adverse Events

Adverse events should be elicited by careful questioning of the subject at each visit. The Investigator and others responsible for care of the subjects should institute any supplementary investigations of significant adverse events based on the clinical judgment of the likely causative factor. This may include seeking a further opinion from a specialist in the field of the adverse event. The company may suggest

special tests based on expert advice. If a serum sample is collected for assay of the test medication or for additional laboratory tests, the Investigator must ensure that the plasma sample is properly labelled and stored.

15.3 Completing Adverse Event Forms

Adverse Event Forms must be completed in a timely manner and contain the following information:

- subject number, initials and date of birth
- description of event (where possible a diagnosis should be made rather than just listing symptoms)
- **relevant** medical history
- study drug, dose and batch number and expiry date
- **all** concurrent medication (comment if suspected cause of event)
- intensity and causality must be assigned following the instructions below:
- action taken (e.g. drug discontinuation) and treatment given
- outcome
- signature of the study physician and date

15.4 Assessment of adverse event intensity

Intensity of adverse events will be assessed by a **nurse or physician**. The following guidelines should be used to assess intensity:

Mild:	Awareness of signs or symptoms that are easily tolerated.
Moderate:	Discomfort enough to cause interference with usual activity.
Severe:	Incapacitating with inability to do usual work.

15.5 Attribution to study drugs

Attribution of adverse effects to study drugs will be assessed by a **physician** according to the following criteria (based on Karch FE, Lasagna L. JAMA 1975; 234: 1236-1241) and recorded on the CRF.

Probable

A reaction that follows a reasonable temporal sequence from administration of the drug, and follows a known response pattern to the suspected drug.

The reaction cannot reasonably be explained by the know characteristics of the subject's clinical state or other modes of therapy administered to the subject.

Possible

Plausible temporal sequence

Follows a known response pattern to the suspected drug

The adverse event might have been produced by the subject's clinical state or other modes of therapy administered to the subject.

Unlikely

The current state of knowledge indicates that a relationship is unlikely

Unknown

It is not possible to assign adverse event to any of the above categories

Not related

In the opinion of the physician the event is unrelated to the study drug. Study drugs are defined as those investigational compounds or their controls used in a study.

In this study, the study drug is: 16% immunoglobulin

15.6 Reporting Adverse Events

The Investigator is responsible for prompt and complete reporting of all adverse events. This facilitates:

- a greater understanding of drug toxicity
- appropriate modification of study protocols
- adherence to regulatory requirements, thus protecting study subjects and prescribing physicians

The Investigator is responsible for complying with his Local Research Ethics Committee's (LREC) policy on adverse event reporting. BPL will inform the MCA in line with regulatory guidelines on adverse events occurring during the trial. The investigator retains the right to inform the MCA if he/she so desires, but must inform BPL so that duplicate reports to the MCA can be highlighted.

WHEN A ADVERSE EVENT OCCURS WHICH FULFILLS THE DEFINITION OF SERIOUS (above) THE INVESTIGATOR MUST IMMEDIATELY:

1. **Telephone or fax available details to the designated CRA or Medical Affairs Manager at BPL**
2. **Complete and sign the adverse event form and send it to the appropriate contacts (see below)**
3. **Inform the LREC of the adverse event where appropriate (i.e. if believed to be study drug related and is unexpected, or if it is a requirement of the adverse event reporting policy of the LREC).**

Mrs Pauline Jackson
Bio Products Laboratory
Dagger Lane
Elstree

Herts WD6 3BX

Telephone: 0181 258 2248

Fax: 0181 258 2611

or

Dr E W Gascoigne

Medical Affairs Manager

Bio Products Laboratory

Elstree

Herts WD6 3BX

Telephone: 0181 258 2622

Fax: 0181 258 2611

Out of hours, BPL Security will contact the Medical Affairs Manager

BPL will inform all Investigators of adverse reactions occurring during the study which would materially affect the safety of study subjects.

15.7 Adverse Event Follow-up

All adverse events will be followed up:

- to resolution

or

- until an underlying condition has been diagnosed

or

- until the patient's condition has stabilised

or

for a period of 28 days following administration of the study drug

16.0 DATA ANALYSIS

16.1 Statistical Analysis

This is a non-comparative study and therefore descriptive statistical techniques will be employed in the analysis of some of the data.

5. Every biomedical research project involving human subjects should be preceded by careful assessment of predictable risks in comparison with foreseeable benefits to the subject or to others. Concern for the interests of the subject must always prevail over the interests of science and society.

6. The right of the research subject to safeguard his or her integrity must always be respected. Every precaution should be taken to respect the privacy of the subject and to minimise the impact of the study on the subject's physical and mental integrity and on the personality of the subject.

7. Physicians should abstain from engaging in research projects involving human subjects unless they are satisfied that the hazards involved are believed to be predictable. Physicians should cease any investigation if the hazards are found to outweigh the potential benefits.

8. In publication of the results of his or her research, the physician is obliged to preserve the accuracy of the results. Reports of experimentation not in accordance with the principles laid down in this Declaration should not be accepted for publication.

9. In any research on human beings, each potential subject must be adequately informed of the aims, methods, anticipated benefits and potential hazards of the study and the discomfort it may entail. He or she should be informed that he or she is at liberty to abstain from participation in the study and that he or she is free to withdraw his or her consent to participation at any time. The physician should then obtain the subject's freely given informed consent, preferably in writing.

10. When obtaining informed consent for the research project the physician should be particularly cautious if the subject is in a dependent relationship to him or her or may consent under duress. In that case the informed consent should be obtained by a physician who is not engaged in the investigation and who is completely independent of this official relationship.

The primary efficacy measurement is defined as the proportion of the total number of trough levels at each time point where the IgG value is greater than or equal to 4g/L. Thus the number of occurrences that this occurs during the study will be calculated. This value of 4g/L has been chosen because from the literature it seems to be an appropriate level where infections start to decrease because of treatment efficacy. However, data from a previous clinical trial conducted by BPL using primary antibody deficiency patients previously treated with IVIG showed that the mean IgG level at baseline was 8g/L (95% CI = 6.1 - 9.9)[6]. During this study the aim was to therefore maintain the patient's IgG trough level within this 95% CI (i.e.6.1 - 9.9) and the number of patients that fall outside this target will be calculated. In addition, if the data is found to be normally distributed, an analysis of variance will be performed to determine the influence of time on trough IgG levels.

The sample size of 40 evaluable patients has been chosen so that an adequate number of patients receiving both IVIG and SCIG prior to study SCIG can be assessed. This number will also allow for drop-outs and withdrawals. While it would be preferable to have equal numbers in both groups, the study is not reliant upon this. However, a minimum of 10 patients receiving IVIG at baseline and 10 receiving SCIG will be aimed for when recruiting patients. These subgroups of patients will be analysed seperately.

The number, type, severity and duration of adverse events, together with monitoring of haematology and biochemical screens and viral markers will also be analysed and described.

16.2 Definition of Evaluability

Patients will be considered evaluable and included in the analysis if they satisfy the entry criteria and receive one or more study SCIG infusions.

16.3 Dropouts

All patients who drop-out or who are withdrawn from the study prior to the first study SCIG infusion will not be considered evaluable for efficacy. The patient's data will however be included in the safety analysis.

All drop-outs and withdrawals must be fully documented and their case record forms submitted to BPL.

16.4 Interim Analysis

If considered necessary by BPL, an interim analysis, using the above statistical tools, will be performed either after at least 10 patients have completed Phase 1 of the study or 20 patients have completed 3 months of SCIG treatment.

17.0 DOCUMENTATION

17.1 Required Pre-Study Documentation

Before the start of the study, BPL will require the following documentation:

- A signed copy of the protocol
- An information leaflet and/or consent form if different from that of the sponsor
- A signed financial agreement
- A signed curriculum vitae for each Investigator and principal co-Investigator
- A copy of laboratory normal ranges for tests required in the protocol
- Evidence of laboratory accreditation and/or performance in relevant National External Quality Assessment Schemes (NEQAS)

- LREC submission, constitution, written approval and composition.
- Signed confidentiality agreement

17.2 Recording data in Case Record Forms

All study data will be recorded on CRFs provided by BPL. These must be completed by the Investigator or a duly authorised assistant. CRFs must be completed in a legible manner using a black ball point pen. Entries made in pencil, colour ink or with a felt tip pen are not acceptable. Entries should be made directly and promptly.

Where data is absent, appropriate abbreviations should be entered instead of leaving blank fields, e.g:

• ND	=	Not done	should be entered where the required test was not performed. The reason should be stated, e.g. instrument failure and then signed and dated.
• NA	=	Not Applicable	should be entered where the CRF requires information which is not appropriate

Where problems arise with blood samples the following abbreviations may be entered:

• H	=	Haemolysed
• SH	=	Slightly haemolysed
• GH	=	Grossly haemolysed
• SS	=	Short sample. The sample is less than that outlined in the protocol
• BC	=	Broken container

17.3 Error Correction

- Errors should be corrected by drawing a single black line through the entry without obscuring the original data.

- Corrections should be recorded beside it and should be signed, dated and an explanation given (if necessary).
- Tippex should never be used

17.4 Signing Off and Return of Case Record Forms

The Investigator at each site must sign the completed CRFs to confirm the validity of the data. The Investigator is obliged to return the completed CRFs to BPL at the end of the study. A copy will be retained in the Investigator file.

17.5 Maintenance and Archiving of Study Records

The Investigator must maintain adequate records for the duration of the study.

Before the study begins the following documentation must be present in the Investigator file:

- CRF
- Investigator Brochure
- Signed final protocol and amendments
- Information sheet and consent form for study patients
- CVs for Investigators and co-Investigators
- Financial agreements between
  - Sponsor and Investigator
  - Investigator and other departments for conducting the study, e.g. laboratory/nursing staff/organisation (if applicable)
- Letter of indemnity (if requested)
- Confidentiality statements
- LREC composition, constitution, submission and letter of approval
- Relevant MREC documentation including submission correspondence and letter of approval
- Normal laboratory values
- Laboratory certification/ accreditation and/or NEQAS performance or validation of

analytical methods used in the study.

- Details of transport of test medication and study supplies

During the study the following documents must be added to the file:

- Updates on Investigators brochure (if applicable)
- Amendments (as applicable)
  - to protocols
  - to CRFs
  - information sheets
  - consent forms
- Dated approvals or opinions for any amendments
- Reports to LREC on study progress
- CVs for new Investigators and co-Investigators
- Updates on normal laboratory values
- Updates on technical procedures/tests
- Drug accountability records
- Initiation report
- Signed and dated completed CRFs
- Documentation of CRF corrections
- All correspondence concerning adverse event reports between
  - Sponsor and Investigator
  - Investigator and LREC
  - Investigator and MCA (if reported directly)
- Interim or annual reports
- Patient screening log
- Patient ID log
- Sample log
- Site signature log
- Final study report (if applicable)
- Correspondence including
  - letters
  - telephone calls
  - meeting notes and minutes
- Signed informed consent forms
- Relevant MREC correspondence

These records must be available for inspection upon reasonable request by the sponsor, members of the regulatory authorities or other authorised individuals.

The Investigator must make proper provision for archiving study documentation at the centre. Patient ID codes must be retained for a

period of 15 years after the issue of the final study report. Patient consent forms and other study related documentation must be retained for the maximum period of time permitted by the hospital.

18. STUDY CONDUCT

18.1 Adherence to the Protocol

Deviations from the protocol must not be made without the prior written approval of BPL and the MREC, except where there are logistical or administrative changes, or where they are implemented to eliminate an immediate threat or hazard to the health or safety of the patient. Where a deviation has been made to eliminate an immediate hazard, the Investigator must submit the implemented deviation and the reasons for it to the MREC and must notify relevant members of the Medical Department of BPL. All deviations must be adequately documented in the CRF.

18.2 Protocol amendments

When an amendment is necessary, the principal researcher must comply with the MREC policy on notification of amendments. If the amendment substantially alters the study design or increases the risk to the patients, the principal researcher must do the following:

1. Submit the amendment to the MREC for review and favourable opinion.
2. Notify BPL in writing of the MREC's opinion.
3. Where appropriate revise the consent form and patient information leaflet and have it approved by the MREC.
4. Where the amendment affects the risk/benefit ratio of continued participation for patients already enrolled in the study, informed

consent should be obtained using the new information leaflet/consent form.

5. Use the updated version of the information leaflet/consent form for new patients.

Similarly, each Investigator is obliged to inform their LREC of any protocol amendments.

18.3 Early Cessation of the Study

BPL reserves the right to stop the study if:

- Evidence is gained that patients are being exposed to an unacceptable risk
- For any reason, it is not possible to continue to supply the study material
- An advancement in knowledge makes the treatment redundant
- Recruitment is too slow to allow accrual of an adequate number of patients in a reasonable length of time

18.4 Monitoring Visits and Audit

The BPL Clinical Research Associate will monitor the study by telephone, correspondence, and regular visits to the investigative sites. He/she will ensure that:

- the facilities remain adequate
- the Investigator adheres to the protocol and ethical responsibilities
- source documents are legible and agree with entries in the CRF
- adverse events are adequately documented and reported
- study medication is properly stored and drug inventories are being maintained

- samples are identified, handled and stored appropriately

Data entered on the CRFs must be subject to validation. According to Good Clinical Practice, this requires that the study CRA should compare data on the CRF with the raw data, e.g. patient notes and laboratory data.

The study CRA will require access to all patient records to allow verification of the entries in the CRF.

The Investigator will agree to make himself available to correct or discuss any discrepancies.

An audit may also be carried out at the centre to ensure that the study has complied with Good Clinical Practice (GCP).

Access to documentation and facilities used during the study may be required by BPL appointed auditors or by regulatory authorities. In the event that an audit is scheduled by the regulatory authorities, the Investigator must notify BPL immediately.

18.5 Report and Publication

The key authors will be from those centres who have entered patients into the study during the recruitment period. Each centre will be responsible for determining the author from that centre. No more than two authors per centre. The centre that recruits the greatest number of patients will be cited first.

The Investigator is obliged to provide BPL with complete test results and all data and reports within 6 weeks of completion or termination of the study.

Should the Investigator wish to publish the results of the study, a copy of the manuscript will be provided to BPL at least 30 days prior to the expected date of submission to the intended publisher.

18.6 Obligations of the Investigator

The Investigator must conduct the study as outlined in the protocol and in accordance with the ICH guidelines for GCP. Please refer to Appendix I on Investigators Responsibilities at the back of the protocol.

18.7 Confidentiality

Information received pursuant to this study will be regarded as confidential at all times. The Investigator and co-Investigators will be required to sign a confidentiality statement.

19.0 ETHICAL CONSIDERATIONS

The study will be performed in accordance with the Guidelines of the Declaration of Helsinki on biomedical research involving human patients (South Africa revision 1996) and in accordance with ICH GCP guidelines. Before the study can begin the principal researcher must have received documentary evidence of approval of the study protocol from the MREC. Subsequently, to gain LREC approval, each Investigator must supply the following documentation for review:

- MREC application form
- MREC letter of approval
- Signed MREC response form
- Protocol
- Patient information leaflet and consent form
- Any advertisement for subject recruitment
- The Investigator Brochure
- Investigator's Curriculum Vitae

Once approval has been granted the Investigator is responsible for ensuring that he/she complies with the terms of the approval namely with respect to adverse event reporting, notification of amendments, interim and final reports on the progress of the study.

19.1 CONSENT PROCEDURE FOR ADULTS

Before the decision to participate is made by adult patients. The investigatoror or a duly authorised deputy, will provide both an oral and written full explanation of the study. If the investigator intends to use his own information leaflet, he must ensure that it contains all the information outlined in Appendix 8 (section 1.8.10). After the patient has been supplied with information and has had sufficient time to review it and ask questions, he/she will be included in the study only if the investigator is sure that the patient understands the implications of taking part. His/her agreement to participate will be documented by the patient signing and dating a consent form. The consent form will be stored at the investigator's site and a signed copy given to the patient.

19.2 CONSENT PROCEDURE FOR CHILDREN

Where children are invited to participate in the study, if in the opinion of the investigator he/she is of sufficient maturity to understand the nature of the study the investigator or a duly authorised deputy, will provide both an oral and written full explanation of the study. If the investigator intends to use his own information leaflet, he must ensure that it contains all the information outlined in Appendix 8 (section1.8.10). After the patient has been supplied with information and has had sufficient time to review it and ask questions, he/she will be included in the study only if the investigator is sure that the child understands the implications of taking part. His/her agreement to participate will be documented by the patient signing and dating a Consent Form, this will be stored at the investigator's site and a signed copy given to the patient.

Where patients are not of sufficient maturity to understand the full implication of taking part in the study the investigator or a duly authorised deputy, will provide both an oral and written full explanation of the study to the parent/guardian. After the parent /guardian has been supplied with information and has had sufficient time to review it and ask questions, he/she will be able to give consent for their child to be included in the study only if the investigator is sure that the parent/guardian understands the implications of their child taking part. His/her agreement to allow their child to participate will be documented by the parent/guardian signing and dating a consent form in addition to the investigator. The Consent Form will be stored at the investigator's site and a signed copy given to the parent/guardian.

In the event that the child does not wish to participate in the study this will preclude them entering even if parent/guardian consent is granted. It is expected that children over the age of 8 years will be of sufficient maturity to provide consent but children younger than this should also be given the opportunity if appropriate.

The subject's GP must be informed of the patient's participation in the study.

19.3 Compensation/Indemnity

Compensation will be paid by BPL according to the Guidelines drawn up by the Association of the British Pharmaceutical Industry if a patient is injured as a result of being in this study.

Compensation will not be provided for injury or medical conditions that are unrelated to this study.

BPL will indemnify the Institute and the Investigator with respect to any claim for personal injury or death brought against it resulting from the administration to volunteer subject of source materials supplied by BPL, provided the protocol and Investigator agreement have been

adhered to, and the event has not been occasioned by malpractice or negligence.

20.0 INVESTIGATOR'S STATEMENT

A multi-centre, open study to assess the safety and efficacy of a 16% immunoglobulin product given via the subcutaneous route in primary antibody deficient patients

I have carefully read this protocol and the Clinical Investigator's Brochure and I confirm that they contain all the information necessary to perform the study. I agree to carry out the study as outlined in this protocol.

Signature: ______________________________

Date: ______________

Name : ______________________________

Centre : ______________________________

21.0 REFERENCES

1. Williams PE, Todd AAM, Yap PL. IVIG Therapy in Primary and Secondary Immunodeficiency. Clinical Applications of Intravenous Immunoglobulin Therapy. Churchill Livingstone 1992: 63-92
2. Berger M, Cupps TR and Fauci AS. Immunoglobulin Replacement Therapy by Slow Subcutaneous Infusion. Ann Intern Med 1980; 93: 55-56
3. Gardulf A, Hammarstrom L, Smith CIE. Home Treatment of Hypogammaglobulinaemia by Rapid Infusion. The Lancet 1992; 338: 162-166
4. Waniewski J, Gardulf A, Hammarstrom L. Bioavailability of γ - Globulin After Subcutaneous Infusions in Patients with Common Variable Immunodeficiency. Journal of Clinical Immunology 1994; vol 14, No 2: 90-97
5. Brennan VM. Subcutaneous Immunoglobulin Therapy: A manual for administration of immunoglobulin by rapid subcutaneous infusion
6. Data on file, VIGPAD Clinical Report, Bio Products Laboratory, Dagger Lane, Elstree, Hertfordshire

APPENDIX 1: ADMINISTRATION OF SUBCUTANEOUS INFUSION

Materials Required

- Sterile paper towel
- Medi-swabs
- Needles for drawing up (19G)
- Syringes (10ml)
- Infusion set
- Butterfly needle (28G)
- Tape
- Cotton wool
- Sharps bin
- Syringe drivers (Graseby MS16A)
- Subcutaneous immunoglobulin
- Adrenaline

Assessment of the Patient

- Vital signs should be taken prior to each infusion and an infusion should not be administered if the patient has a fever or is unwell

Method

1. Check syringe drivers and set rate as prescribed
2. Wash hands
3. Clean surface and lay paper towel
4. Check immunoglobulin for correct dose and expiry date
5. Remove vial tops and draw up immunoglobulin with syringe and needle
6. Attach infusion needle to syringe and prime tubing
7. Prepare tape and wipe site (abdomen/thigh) to be used with medi-swab
8. Insert the needle into the abdomen/thigh as instructed and tape firmly in place
9. Check for any blood return by withdrawing the syringe plunger and by removing the syringe from the tubing
10. If blood returns re-site the needle and repeat 9
11. Once position is satisfactory with no blood return, place the syringe on the driver and secure in place
12. Switch on the driver and administer infusion
13. When the alarm sounds remove the needle and dispose of in the sharps bin
14. Repeat process if further dose is needed
15. At the end of the infusion dispose of all used materials, wash hands and record details

APPENDIX 2 ADULT PATIENT INFORMATION LEAFLET

A MULTI-CENTRE, OPEN STUDY TO ASSESS THE SAFETY AND EFFICACY OF A 16% IMMUNOGLOBULIN PRODUCT GIVEN VIA THE SUBCUTANEOUS ROUTE IN PRIMARY ANTIBODY DEFICIENT PATIENTS

This purpose of this leaflet is to tell you about a research study in which you are invited to take part. If there is anything which you do not understand, or if you require more information, please ask your doctor or nurse. Please read these notes carefully, before starting the study, and keep them safe so that you may look at them again. The study has been reviewed and approved by the hospital ethics committee.

Why is the study being done?
You have been asked to take part in a study to test a specially treated blood product. It is called immunoglobulin and is used to treat your condition (primary antibody deficiency) by replacing the low levels of antibodies in the blood. At the moment most patients in the UK receive their immunoglobulin treatment into a vein every few weeks over a number of hours. However, in other countries such as Sweden, they give it under the skin (subcutaneous). This can be useful for home therapy or for patients who cannot have their infusions into a vein for medical reasons. The immunoglobulin product that we will be using in this study is virtually identical to the Swedish one but we want to check that giving it under the skin is safe and keeps your condition controlled.

Are there other ways of treating my condition?
Primary antibody deficiency causes low levels of the antibodies in the blood which are there to help fight infection. Therefore, these antibodies need to be replaced. This is usually achieved by giving immunoglobulin treatment into a vein. A few patients already receive their immunoglobulin under the skin by special arrangement with the people who make it but at the moment there are no products available on the market that have a licence. There are a quite a few products available that are already licensed to be given into a vein however and you may well be receiving one of these already.

Why have I been chosen and who is organising the study?
The study will involve at least 40 patients from around the country, each of whom have the same condition as you. The study as a whole is expected to last about 2 years.

The organisers of the study are called Bio Products Laboratory (BPL), which is a part of the blood donor organisation, the National Blood Authority. They are the manufacturers of the immunoglobulin that will be used in the study.

What are the possible benefits of taking part?
Immunoglobulin therapy is a recognised treatment for primary antibody deficiency so the number of antibodies in your blood would be kept at a suitable level to control your condition. However, having the treatment under the skin instead of into a vein may give a better level of antibodies which also may not go up and down as much as sometimes happens with immunoglobulin treatment into a vein. Also, the results that we get from this study should give doctors more treatment choices in the future.

What are the risks of taking part or what if something goes wrong?
The blood tests may hurt slightly and cause slight bruising. You may need to have a few extra blood tests done for the purpose of the study than you would normally have, but this should not cause you any harm. You may get some swelling and redness where the immunoglobulin is injected but this should go away within a day. Serious problems do not happen very often but we need to let you know what they could be just in case - hard skin under the injection site, pains in the chest, arms or legs, feeling out of breath, shaking hands, feeling dizzy, puffy face and a sore mouth. However, so far, in BPL's experience, only 1 serious problem in 61,000 intramuscular doses has been reported following treatment.
Although all blood donations used to make the immunoglobulin are checked for hepatitis B, hepatitis C and HIV viruses, there is still a small risk that one of these could be passed on. The product is specially treated to destroy them however, so far there has been no reports of patients getting them from the product. At the end of the study, your blood would need to be tested to check that none of the viruses have been passed on to you during the course of the study. If you do not wish these tests to be carried out then you should not take part in the study.

If you are harmed because of the study, compensation would be provided without you having to demonstrate fault. Bio Products Laboratory, operates to the guidelines drawn up by the Association of the British Pharmaceutical Industry for subjects in clinical trials and will therefore treat any claim according to these guidelines. A copy is available from your doctor if you ask him/her.

What would I have to do if I decided to take part in the study?
Before the start of the study, you would be asked about your medical history and be given a complete medical examination. The doctor or nurse would take your blood pressure, pulse rate, temperature and respiration rate. You would also have a blood test.

If you are suitable for the study, you would carry on with your normal immunoglobulin treatment for up to 3 more times. This is so we can collect some baseline information. When your routine treatments have finished, you would start coming to the clinic one week later to receive the immunoglobulin subcutaneously. The first dose would be given at 100mg/kg bodyweight. After that, you would come back every week for your treatment and your doctor would take a blood test at each visit to decide what dose you should have each time. Some of the blood would also be saved while you are on the study in case it needs to be tested again. This would be discarded at the end of the study except for blood taken at the beginning and end of the study which would be kept for at least 15 years in a freezer in case we ever need to re-test it to re-check the results of this study. It would not be used for any other purpose than this such as another study.

Before the treatment, every hour during and again afterwards the nurse would take your temperature, blood pressure, pulse and respiration rates. You would also be watched for any side effects and the injection site would be checked. The treatment would be given under the skin in two places at the same time either in your thigh or abdomen whichever is best for you.

You would carry on with these infusions every week for about six months after which you would need to decide whether you want to carry on with the study or go back to your normal treatment. Your doctor will be able to advise you. If you carry on with the study treatment you would need to complete a weekly diary card and see the doctor every 3 months for a check-up. You would be able to carry on until the product has a licence which would then be the end of the study. You would not need any more blood tests during this time unless the doctor thought you needed them except for a test right at the end of the study to check that none of the viruses mentioned before had been passed on to you.

Would there be any restrictions?

You should not take any alcohol for at least 12 hours before the first treatment.

You should carry on with any medicines your doctor has told you to take but try not to take any other medicines the day before the first treatment and if possible during the study. If you need to take anything else, including ones that you can buy from the chemist yourself, please remember to tell the doctor.

What if I don't want to take part?

You don't have to take part in the study if you don't want to. If you decide to take part then you can change your mind at any point without giving a reason. This would not affect your medical care now or in the future. If, in the future, you decide you do not want to stay in the study, you must tell your doctor or the study staff and they will tell you what to do.

The doctor may decide to take you out of the study if it is in your best interest, with or without your consent. If you come out of the study, you would continue to receive other appropriate treatments for your condition. You would also be told by your doctor of any new information which may help you decide whether or not to carry on with the study.

Would my records be kept secret?

Staff from BPL and possibly the Regulatory Authorities, such as the Medicines Control Agency, would need to look at your hospital notes. However, all information about you in this study would be kept secret and if the results of the study are published, your name would not appear and no one would know your identity. If you decide to take part your GP would be told.

Would I get paid for taking part?

You would not be paid to take part in this study. However, reasonable travel expenses to and from the clinic as well as other study related expenses you may incur, will be paid.

Who should I ask if I have any more questions?

Any questions you have about this study can be answered by Dr/Nurse ______________________ who may be reached by telephone on ____________________

If you have any questions about your rights as a research subject or about an injury related to the study you should contact Dr/Nurse __________________ as above.

In an emergency, please contact:

Name: ____________________________

Tel: ____________________________

APPENDIX 3: PARENT/GUARDIAN INFORMATION SHEET

A MULTI-CENTRE, OPEN STUDY TO ASSESS THE SAFETY AND EFFICACY OF A 16% IMMUNOGLOBULIN PRODUCT GIVEN VIA THE SUBCUTANEOUS ROUTE IN PRIMARY ANTIBODY DEFICIENT PATIENTS

This purpose of this leaflet is to tell you about a research study in which your child is invited to take part. If you agree to allow your child to take part in the study yuo will be asked to sign a consent form and if your child is old enough to understnd the study then he/she will also be asked to sign the form after the study has been explained to heim/her and if he/she wants to take part. If there is anything which you do not understand or if you require more information, please ask the doctor or nurse. Please read these notes carefully, before your child starts the study, and keep them safe so that you may look at them again. The study has been reviewed and approved by the hospital ethics committee.

Why is the study being done?

Your child has been asked to tke part in a study to test a specially treated blood product. It is called immunoglobulin and is used to treat his/her condition (primary antibody deficiency) by replacing the low levels of antibodies in the blood. At the moment most patients in the UK receive their immunoglobulin treatment into a vein every few weeks over a number of hours. However, in other countries such as Sweden, they give it under the skin (subcutaneous). This can be useful for home treatment or for patients who cannot have their infusions into a vein for medical reasons. The immunoglobulin product that we will be using in this study is virtually identical to the Swedish one but we want to check that giving it under the skin is safe and keeps your child's condition controlled.

Are there other ways of treating my condition?

Primary antibody deficiency causes low levels of the antibodies in the blood which are there to help fight infection. Therefore, these antibodies need to be replaced. This is usually achieved by giving immunoglobulin treatment into a vein. A few patients already receive their immunoglobulin under the skin by special arrangement with the people who make it but at the moment there are no products available on the market that have a licence. There are quite a few products available that are already licensed to be given into a vein however and your child may well be receiving one of these already.

Why has my child be chosen and who is organising the study?

Thes study will involve at least 40 patients from around the country each of whom have the same condition as your child. The study as a whole is expected to last about 2 years.

The organisers of the study are called Bio Products Laboratory's (BPL), which is a part of the blood donor organisation, the National Blood Authority. They are the manufacturers of the immunoglobulin that will be used in the study.

What are the possible benefits of him/her taking part?

Immunoglobulin therapy is a recognised treatment for primary antibody deficiency so the number of antibodies in your child's blood would be kept at a suitable level to control his/her condition. However, having the treatment under the skin instead of into a vein may give a better level of antibodies which also may not go up and down as much as sometimes happens with immunoglobulin treatment into a vein. Also, the results that we get from this study should give doctors more treatment choices in the future.

What are the risks of taking part or what if something goes wrong?

The blood tests may hurt slightly and cause slight bruising. Your child may need to have a few extra blood tests done for the purpose of the study than he/she would normally have, but this should not cause him/her any harm. There may be some swelling and redness where the immunoglobulin is injected but this should go away within a day. Serious problems do not happen very often but we need to let you know what they could be just in case - hard skin under the injection site, pains in the chest, arms or legs, feeling out of breath, shaking hands, feeling dizzy, puffy face and a sore mouth. However, so far, in BPL's experience, only 1 serious problem in 61,000 intramuscular doses has been reporte following treatment.

Although all blood donations used to make the immunoglobulin are checked for HIV, hepatitis B and hepatitis C viruses, there is still a small risk that one of these could be passed on. The product is specially treated to destry them however, so far there has been no report sof patients getting them from the product. At the end of the study, your child's blood would need to be tested to check that neither hepatitis B nor hepatitis C have been passed on to him/her during the course of the study (HIV testing is not normally done on children). If you do not wish these tests to be carried out then you should not agree for him/her to take part in the study.

If your child is harmed because of the study compensation would be provided without you or your child having to demonstrate fault. Bio Products Laboratory, operates to the guidelines drawn up by the Association of the British Pharmaceutical Industry for subjects in clinical trials and will therefore treat any claim according to these guidelines. A claim can be made via the investigator, setting out details and nature of the claim which operates to the Clinical trial compensation guidelines as drawn up by ABPI. A copy of the guideline is available from the doctor if you ask him/her.

What would my child have to do if he/she took part in the study?

Before the start of the study, you and you child would be asked about his/her medical history and he/she would be given a complete medical examination. The doctor or nurse would take his/her blood pressure, pulse rate, temperature and respiration rate. He/she would also have a blood test.

If your child is suitable for the study, he/she would carry on with their normal immunoglobulin treatment for up to 3 more times. This is so we can collect some baseline information. When the routine treatments have finished, your child would start to receive the immunoglobulin under the skin, one week later. The first dose would be given at 100mg/kg bodyweight. After that, he/she would come back every week for the new treatment and the doctor would take some blood at some of the visits if necessary. Some of the blood would also be saved while your child is on the study in case it needs to be tested again. This would be discarded at the end of the study except for blood taken at the beginning and end of the study which would be kept for at least 15 years in a freezer in case we ever need to re-test it to re-check the results of this study. It would not be used for any other purpose than this such as another study. Further consent to perform test on these samples should it be necessary, would not be sought and the results of any further research tests performed on these samples would not be made available to parent/child.

Before the treatment, every hour during and again afterwards the nurse would take your child's temperature, blood pressure, pulse and respiration rates. He/she would also be watched for any side effects and the injection site would be checked. The treatment would be given under the skin in either the thigh or abdomen whichever is best for him/her.

Your child would carry on with these infusions every week for about six months after which you and your child would need to decide whether you want him/her to carry on with the study or go back to his/her normal treatment. The doctor will be able to give you both advice. If your child carries on with the study treatment, a weekly diary card would need to be completed and your child would see the doctor every 3 months for a check-up. Your child would be able to carry on until the product has a licence which would then be the end of the study. He/she would not need any more

blood tests during this time unless the doctor thought they were necessary except for a test right at the end of the study to check that none of the viruses mentioned before had been passed on and no further consent will be obtained or required for pre and/or completed samples.

Would there be any restrictions?
Your child should carry on with any medicines precribed by his/her doctor but try not to give him/her any other medicines the day before the first treatment and if possible during the study. If he/she does need to take anything else, including ones that you can buy from the chemist yourself, please remember to tell the doctor.

What if my child or I don't want him/her to take part?
Your child does not have to take part in the study. If you agree to your child taking part then you can change your mind at any point without giving a reason. This would not affect your child's medical care now or in the future. If, in the future, you decide you do not want your child to stay in the study, you must tell the doctor or the study staff and they will tell you what to do.

The doctor may decide to take your child out of the study if it is in his/her best interest, with or without your consent. If he/she comes out of the study, he/she would continue to receive other appropriate treatments for his/her condition. You would also be told by the doctor of any new information which may help you decide whether or not to carry on with the study.

Consent procedure for children
Children are legally dependent on their parents/guardians who take the legal responsibility for their welfare and safety and fully informed consent should be obtained from the legal guardian in accordance with national legislation. Children can consent if the Investigator is sure that the child understands the study requirements and implication of taking part. His/her agreement to take part will be documented by the patient signing and dating a consent form in addition to the parent/guardian. The Consent Form will be stored at the Investigator's site and a signed copy given to the patient.

Would my child's records be kept secret?
Staff from BPL and possibly the Regulatory Authorities, such as the Medicines Control Agency, would need to look at your child's hospital notes. However, all information about him/her in this study would be kept secret and if the results of the study are published, his/her name would not appear and no one would know his/her identity. If you and your child decide to take part his/her GP would be told.

Would my child get paid for taking part?
He/she would not be paid to take part in this study. However, reasonable travel expenses to and from the clinic as well as other study related expenses incurred, will be paid for.

Who should I ask if I have any more questions?
Any questions you have about this study can be answered by Dr/Nurse ________________________ who may be reached by telephone on ____________________

If you have any questions about your child's rights as a research subject or about an injury related to the study you should contact Dr/Nurse __________________ as above.

In an emergency, please contact:

Name: ______________________________ **Tel:** ______________________________

APPENDIX 4: CHILDREN'S INFORMATION LEAFLET

A MULTI-CENTRE, OPEN STUDY TO ASSESS THE SAFETY AND EFFICACY OF A 16% IMMUNOGLOBULIN PRODUCT GIVEN VIA THE SUBCUTANEOUS ROUTE IN PRIMARY ANTIBODY DEFICIENT PATIENTS

This leaflet tells you about a study which you can take part in if you want. If you do not understand what you have to do then please ask your doctor or nurse.

Why is the study being done?

In this study we want to test a medicine called an immunoglobulin. It comes from blood donors and is usually given to people, with the same illness as you, into their muscle or vein. At the moment most patients in Britain are given it into a vein every few weeks. But, in other countries such as Sweden they are given it under the skin if they cannot have it in a vein. We want to check that this medicine is safe to give under the skin and also that it helps your illness.

Why have I been picked?

There will be about 40 patients from different hospitals in Britain who will take part in the study. They will all have the same illness as you. The people who make the medicine are called Bio Products Laboratory (BPL). This is part of the same place that blood donors go to when they give blood.

What are the good and bad sides of taking part?

This treatment should make you feel better by helping you to fight coughs and colds. Also, the results will help doctors treat patients like you in the future. Taking blood may hurt a little bit and you may get a small bruise on your arm. You would need to have a blood test every month during the study. Also the doctor will save some of your blood each time in case the doctor needs to test it again. When you have your treatment, you may get a bit of swelling and redness where it goes in under the skin but this should go away by the next day. You shouldn't get any other problems but we have to tell you what they could be in case they happen. You could get a hard lump under the skin or a pain in your chest, arms or legs. You may get out of breath, feel dizzy or shaky, get a puffy face or a sore mouth. These are all very unlikely though.

All the blood that makes the medicine is tested for nasty bugs and is made in a special way that kills them. At the end of the study, we would like to test your blood to check that you haven't caught any of these bugs during the study. If you are hurt in any way because of the study it wouldn't be your fault and the people who make the medicine would pay for this if it happened.

What would I have to do?

Before it started your doctor would ask you about yourself and give you a check-up. You would be weighed, have your temperature taken and your heart beat and breathing checked. You would also need to have a blood test. You would then have your usual treatment for up to 3 more times. Then you would come to the hospital every week to get your medicine under the skin in your leg or tummy. Your doctor may want to take a blood test at some of these visits. While you are having the medicine you would have your temperature taken and your heart beat and breathing checked. The nurse would also watch you for any problems.

You would carry on like this for 6 months and then you would be able to go back to your normal treatment or carry on with this new one if you wanted. If you carried on you would

need to fill in a weekly diary and see the doctor every few months. You wouldn't need to have any more blood tests unless your doctor thought you needed them.

You don't have to take part in the study if you don't want to. Also, you can change your mind at any time once you have started. Remember to tell your doctor or nurse if you are worried about anything and they will help you.

Who should I ask if I have any more questions?

Any questions you have about this study can be answered by Dr/Nurse ______________________ who may be reached by telephone on ____________________

If you have any questions about your child's rights as a research subject or about an injury related to the study you should contact Dr/Nurse __________________ as above.

In an emergency, please contact:

Name: ____________________________ **Tel:** ____________________________

APPENDIX 5 : ADULT CONSENT FORM

(This part to be completed by the patient	*(circle one)*
1. Have you read the Patient Information Sheet?<br>(please take a copy home with you to keep)	YES/NO
2. Have you had an opportunity to discuss the study and ask any questions?	YES/NO
3. Have you had satisfactory answers to all of your questions?	YES/NO
4. Have you received enough information about the study?	YES/NO
5. Who has given you an explanation about the study?<br>Dr/Nurse ______________________	
6. Do you understand that you are free to withdraw from the study:<br>• At any time?<br>• Without having to give a reason?<br>• Without affecting your future medical care?	YES/NO
1. Sections of your medical notes relating to your participation in the study may be inspected by responsible individuals from BPL or from regulatory authorities. All personal details will be treated as strictly confidential.<br>Do you give permission for these individuals to have access to your records?	YES/NO
2. Has the doctor discussed the circumstances when compensation may be due?	YES/NO
3. Have you had sufficient time to come to your decision?	YES/NO
4. Do you consent to your blood being tested for hepatitis B, C and HIV viruses?	YES/NO
5. Do you agree to take part in the study?	YES/NO

PATIENT *(Please sign below and date your own signature)*

Signed: ______________________ Date: ______________

Print Name: ______________________

INVESTIGATOR

Signed: ______________________ Date: ______________

Print Name: ______________________

APPENDIX 6: CHILDREN'S CONSENT FORM

This form should be completed and signed by the patient if they are able to understand the study or if not, by the parent/guardian

	(circle one)
1. Have you read the Information Sheet? *(please take a copy home with you to keep)*	YES/NO
2. Have you had enough information about the study and had a chance to ask any questions?	YES/NO
3. Do you understand all the answers to your questions?	YES/NO
4. Who has talked to you about the study? Dr/Nurse ______________________	
5. Do you understand that you can withdraw from the study: • At any time? • Without having to say why? • Without affecting your care?	YES/NO
6. Some of your medical notes may be looked at by people from BPL or from the Government. They will be kept secret. Will you let these people look at your notes?	YES/NO
7. Has the doctor told you when compensation may be due?	YES/NO
8. Have you had enough time to make your mind up?	YES/NO
9. Do you agree to your blood being tested for bugs (hepatitis B and C viruses?)	YES/NO
10. Do you want to take part in the study?	YES/NO

PARENT/GUARDIAN *(Please answer the questions, sign below and date your own signature)*

Is your child able to understand the nature of the trial and give consent to take part? YES/NO

If YES, will the consent be verbal or written? Verbal/Written/NA

Signed: ______________________ Date: ______________________

Print Name: ______________________ Relationship: ______________________

CHILD *(Please sign below and add the date)*

Signed: ______________________ Date: ______________________

Print Name: ______________________

INVESTIGATOR

Signed: ______________________ Date: ______________________

Print Name: ______________________

APPENDIX 7: PATIENT INFORMATION LEAFLET FOR EXTRA BLOOD TESTS (PHARMACOKINETICS)

A MULTI-CENTRE, OPEN STUDY TO ASSESS THE SAFETY AND EFFICACY OF A 16% IMMUNOGLOBULIN PRODUCT GIVEN VIA THE SUBCUTANEOUS ROUTE IN PRIMARY ANTIBODY DEFICIENT PATIENTS

This purpose of this leaflet is to tell you about an extra part to the research study in which you are already taking part in. You do not have to do this extra part if you do not want to and if there is anything which you do not understand or if you require more information, please ask your doctor or nurse.

Why are the extra blood tests being done?
The reason we would like to do these extra blood tests is so we can follow, on a daily basis, what happens to the level of antibodies in your blood after you have had the first subcutaneous infusion and again after infusion 18, 3 months later. This will help us to understand more about how the immunoglobulin affects your condition. It will also allow us to check that the new treatment produces suitable levels of immunoglobulin in your blood.

What would I have to do?
If you agreed to the extra tests, you would need to have some blood taken every day for one week at about the same time each day. This would be done twice during the study after the first study subcutaneous infusion and again after infusion number 18 (3 months later) making a total of two weeks extra blood sampling. How and when the blood would be collected would be discussed with you by your doctor or nurse.

Are there any risks involved?
The blood tests may hurt slightly and cause slight bruising. Having the extra blood taken would not cause you any harm overall however and you can change your mind at any time about doing them without it affecting the rest of the study or your future medical care.

APPENDIX 8: PHARMACOKINETICS CONSENT FORM

(This part to be completed by the patient) *(circle one)*

1. Have you read the Patient Information Sheet? *(please take a copy home with you to keep)* YES/NO
2. Have you had an chance to discuss the extra blood tests and ask any questions? YES/NO
3. Have you had satisfactory answers to all of your questions? YES/NO
4. Have you received enough information about the extra blood tests? YES/NO
5. Who has given you an explanation about the extra blood tests?

   Dr/Nurse ____________________

6. Do you understand that you are free to stop the extra blood tests:
   - At any time?
   - Without having to give a reason? YES/NO
   - Without affecting the rest of the study or your future medical care?

1. Have you had enough time to come to your decision? YES/NO
2. Do you agree to the extra blood tests? YES/NO

PATIENT *(Please sign below and date your own signature)*

Signed: ____________________________ Date: _______________

Print Name: __________________________

INVESTIGATOR *(Please sign below and date your own signature)*

Signed: ____________________________ Date: _______________

Print Name: __________________________

APPENDIX 9: EXAMPLE GP LETTER

Our ref: SCIG01/BPL

«GPNAME»
«GPADDRESS»

Dear «GPNAME»

Your patient, «PATNAME», has volunteered to take part in an open study to assess the safety and efficacy of a 16% immunoglobulin preparation given via the subcutaneous route in primary antibody deficiency.

Your patient will receive weekly infusions of subcutaneous immunoglobulin at an initial dose of 100mg/kg. The dose will then be tailored to maintain an IgG level of at least 6g/l. The treatment duration will be 6 months but at the end of this period your patient may opt to participate in the follow-on safety phase of the study and continue to receive weekly infusions until such time they decide to withdraw or the product is marketed.

Your patient may be considered suitable to administer their infusions at home after a period of training. If this is the case, the study staff will contact you with the relevant details and seek your agreement before your patient commences home therapy.

Your patient will have undergone a medical examination, including blood tests, before being included in the study. He/she will have close medical monitoring during the infusion and at the end of the study he/she will have another thorough medical.

The trial is being carried out to Good Clinical Practice and has been approved by the Multi Centre Research Ethics Committee (MREC) and the Local Research Ethics Committee (LREC). If there is any information regarding your patient's health which may be relevant to participation in this study, please can you let me know.

If I can be of any further assistance, please do not hesitate to contact me«INVESTNAME» on «INVESTNO»

Yours sincerely,

«INVESTNO»
«INVESTNO»

APPENDIX 10: HOME THERAPY MANUAL

Patient Information

For patients who have primary antibody deficiency, there are several ways of replacing the missing antibodies such as by intramuscular injection or by intravenous infusion. In the past, slow subcutaneous infusions were given but these infusions proved cumbersome and entailed overnight stays in hospital. There is now a new method of replacing the antibodies by rapid subcutaneous infusion which is easy to learn and is advantageous for patients who would be suitable for home therapy.

From recent studies performed in Sweden, immunoglobulin replacement therapy by subcutaneous infusion has proved to be a safe, efficient, time-saving, cost-effective and convenient form of administration. Your doctor thinks that this method would be suitable for you to try. Before you can perform home therapy however you need to satisfy the following criteria:

1. A relative/friend who has also been trained in the techniques must be available to assist with each infusion performed at home

2. You and your relative/friend must be motivated to perform self-infusion

3. After a period of training by the study staff, both you and your relative/friend must be able to manage the infusion techniques

4. Your G.P. must agree to you performing home therapy

5. You must be contactable by telephone.

The training will take place over a period of 8-10 weeks when you come for your study infusions. You and your relative/friend will be shown how to draw the immunoglobulin up from the ampoule into the syringe, insert the butterfly needle under the skin in the thigh or abdomen and connect the syringe to the battery powered syringe driver. The study staff will also show you how to calculate the correct dose and infusion rate .

Materials Required

- Sterile paper towel
- Medi-swabs
- Needles for drawing up (19G)
- Syringes (10ml)
- Infusion set
- Butterfly needle (28G)
- Tape
- Cotton wool
- Sharps bin
- Syringe drivers (Graseby MS16A)
- Subcutaneous immunoglobulin
- Adrenaline

Temperature Record

You must record your temperature on the infusion diary prior to starting the infusion. If this is above the level specified by your study staff ring the clinic and ask for advice before infusing. Also, an infusion should not be administered if you are feverish or are unwell

Method

1. Check syringe drivers and set rate as prescribed
2. Wash hands
3. Clean surface and lay paper towel
4. Check immunoglobulin for correct dose and expiry date
5. Remove vial tops and draw up immunoglobulin with syringe and needle
6. Attach infusion needle to syringe and prime tubing
7. Prepare tape and wipe site (abdomen/thigh) to be used with medi-swab
8. Insert the needle into the abdomen/thigh as instructed and tape firmly in place
9. Check for any blood return by withdrawing the syringe plunger and by removing the syringe from the tubing
10. If blood returns re-site the needle and repeat 9
11. Once position is satisfactory with no blood return, place the syringe on the driver and secure in place
12. Switch on the driver and administer infusion
13. When the alarm sounds remove the needle and dispose of in the sharps bin
14. Repeat process if further dose is needed
15. At the end of the infusion dispose of all used materials, wash hands and record details

APPENDIX 11: HOME THERAPY CONSENT FORM

(This part to be completed by the patient/parent/guardian *(circle one)*

1. Have you read the home therapy information? YES/NO
2. Have you had an opportunity to discuss home therapy and ask any questions? YES/NO
3. Have you had satisfactory answers to all of your questions? YES/NO
4. Have you received enough information about home therapy? YES/NO
5. Who has given you an explanation about home therapy?

   Dr/Nurse ____________________

6. Do you have a relative or friend who is also willing to be trained on home therapy and who will be available to assist you with each home infusion? YES/NO
7. Has your GP been consulted and his agreement for you to commence home therapy been sought? YES/NO
8. Do you agree to be trained to infuse your/your child's subcutaneous immunoglobulin? YES/NO

PATIENT/PARENT/GUARDIAN *(Please sign below and date your own signature)*

Signed: ____________________ Date: ____________

Print Name: ____________________ Relationship: ____________
(if applicable)

RELATIVE/FRIEND *(Please sign below and date your own signature)*

Signed: ____________________ Date: ____________

Print Name: ____________________ Relationship: ____________
(if applicable)

INVESTIGATOR

Signed: ____________________ Date: ____________

Print Name: ____________________

APPENDIX 12: INFUSION RECORD

Maximum temperature for this patient before advice should be sought: ☐☐.☐ °C

If your temperature prior to the infusion is above this figure then you must contact the study staff for advice: Contact Number: ______________________________

Date	Temp °C	Site*	Dose per Site	Total Dose	Rate mm/hr	Batch No.	Comments/Problems

* A = Abdomen
T = Thigh

APPENDIX 13: LOCAL INVESTIGATOR'S RESPONSIBILITIES BASED ON THE ICH GUIDELINES FOR GOOD CLINICAL PRACTICE

1. LOCAL INVESTIGATOR

1.1 Investigator's Qualifications and Agreements

1.1.1 The Investigator(s) should be qualified by education, training, and experience to assume responsibility for the proper conduct of the study, should meet all the qualifications specified by the applicable regulatory requirement(s), and should provide evidence of such qualifications through up-to-date curriculum vitae and/or other relevant documentation requested by the sponsor, the LREC, and/or the regulatory authority(ies).

1.1.2 The Investigator should be thoroughly familiar with the appropriate use of the investigational product(s), as described in the protocol, in the current Investigator's Brochure, in the product information and in other information sources provided by the sponsor.

1.1.3 The Investigator should be aware of, and should comply with, GCP and the applicable regulatory requirements.

1.1.4 The Investigator should permit monitoring and auditing by the sponsor, and inspection by the appropriate regulatory authority(ies).

1.1.5 The Investigator should maintain a list of appropriately qualified persons to whom the Investigator has delegated significant study-related duties.

1.2 Adequate Resources

1.2.1 The Investigator should be able to demonstrate (e.g. based on retrospective data) a potential for recruiting the required number of suitable subjects within the agreed recruitment period.

1.2.2 The Investigator should have sufficient time to properly conduct and complete the study within the agreed study period.

1.2.3 The Investigator should have available an adequate number of qualified staff and adequate facilities for the foreseen duration of the study to conduct the study properly and safely.

1.2.4 The Investigator should ensure that all persons assisting with the study are adequately informed about the protocol, the investigational product(s), and their study-related duties and functions.

1.3 Medical Care of Study Subjects

1.3.1 A qualified physician (or dentist, when appropriate), who is an Investigator or a sub-Investigator for the study, should be responsible for all study-related medical (or dental) decisions.

1.3.2 During and following a subject's participation in a study, the Investigator should ensure that adequate medical care is provided to a subject for any adverse events, including clinically significant laboratory values, related to the study. The Investigator should inform a subject when medical care is needed for intercurrent illness(es) of which the Investigator becomes aware.

1.3.3 It is recommended that the Investigator inform the subject's primary physician about the subject's participation in the study if the subject has a primary physician and if the subject agrees to the primary physician being informed.

1.3.4 Although a subject is not obliged to give his/her reason(s) for withdrawing prematurely from a study, the Investigator should make a reasonable effort to ascertain the reason(s), while fully respecting the subject's rights.

1.4 Communication with LREC

1.4.1 Before initiating a study, the Investigator should have written and dated approval/favourable opinion from the LREC for the study protocol, written informed consent form, consent form updates, subject recruitment procedures (e.g., advertisements), and any other written information to be provided to subjects.

1.4.2 As part of the Investigator's written application to the LREC, the Investigator should provide the LREC with a current copy of the MREC approval documentation.

1.4.3 During the study the Investigator should provide to the LREC all documents subject to review.

1.5 Compliance with Protocol

1.5.1 The Investigator should conduct the study in compliance with the protocol agreed to by the sponsor and, if required, by the regulatory authority(ies) and which was given approval/favourable opinion by the MREC. The Investigator and the sponsor should sign the protocol, or an alternative contract, to confirm agreement.

1.5.2 The Investigator should not implement any deviation from, or changes of the protocol without agreement by the sponsor and prior review and documented approval/favourable opinion from the MREC of an amendment, except where necessary to eliminate an immediate hazard(s) to study subjects, or when the change(s) involves only logistical or administrative aspects of the study (e.g. change in monitor(s), change of telephone number(s)).

1.5.3 The Investigator, or person designated by the Investigator, should document and explain any deviation from the approved protocol.

1.5.4 The Investigator may implement a deviation from, or a change of, the protocol to eliminate an immediate hazard(s) to study subjects without prior MREC approval/favourable opinion. As soon as possible, the implemented deviation or change, the reasons for it, and, if appropriate, the proposed protocol amendment(s) should be submitted.

a) to the MREC for review and approval/favourable opinion,

b) to the sponsor for agreement and, if required,

c) to the regulatory authority(ies).

1.6 Investigational Product(s)

1.6.1 Responsibility for investigational product(s) accountability at the study site(s) rests with the Investigator.

1.6.2 Where allowed/required, the Investigator may/should assign some or all of the Investigator's duties for investigational product(s) accountability at the study site(s) to an appropriate pharmacist or another appropriate individual who is under the supervision of the Investigator.

1.6.3 The Investigator and/or a pharmacist or other appropriate individual, who is designated by the Investigator, should maintain records of the product's delivery to the study site, the inventory at the site, the use by each subject, and the return to the sponsor or alternative disposition of unused product(s). These records should include dates, quantities, batch/serial numbers expiration dates (if applicable), and the unique code numbers assigned to the investigational product(s) and study subjects. Investigators should maintain records that document adequately that the subjects were provided the doses specified by the protocol and reconcile all investigational product(s) received from the sponsor.

1.6.4 The investigational product(s) should be stored as specified by the sponsor and in accordance with applicable regulatory requirement(s).

1.6.5 The Investigator should ensure that the investigational product(s) are used only in accordance with the approved protocol.

1.6.6 The Investigator, or a person designated by the Investigator, should explain the correct use of the investigational product(s) to each subject and should check, at intervals appropriate for the study, that each subject is following the instructions properly.

1.7 Randomisation Procedures and Unblinding

The Investigator should follow the study's randomisation procedures, if any, and should ensure that the code is broken only in accordance with the protocol. If the study is blinded, the Investigator should promptly document and explain to the sponsor any

premature unblinding,(e.g., accidental unblinding, unblinding due to a serious adverse event) of the investigational product(s).

1.8 Informed Consent of Study Subjects

1.8.1 In obtaining and documenting informed consent, the Investigator should comply with the applicable regulatory requirement(s), and should adhere to GCP and to the ethical principles that have their origin in the Declaration of Helsinki. Prior to the beginning of the study, the Investigator should have the MREC and LREC's written approval/favourable opinion of the written informed consent form and any other written information to be provided to subjects.

1.8.2 The written informed consent form and any other written information to be provided to subjects should be revised whenever important new information becomes available that may be relevant to the subject's consent. Any revised written informed consent form, and written information should receive the MREC and LREC's approval/favourable opinion in advance of use. The subject or the subject's legally acceptable representative should be informed in a timely manner if new information becomes available that may be relevant to the subject's willingness to continue participation in the study. The communication of this information should be documented.

1.8.3 Neither the Investigator, nor the study staff, should coerce or unduly influence a subject to participate or to continue to participate in a study.

1.8.4 None of the oral and written information concerning the study, including the written informed consent form, should contain any language that causes the subject or the subject's legally acceptable representative to waive or to appear to waive any legal rights, or that releases or appears to release the Investigator, the institution, the sponsor, or their agents from liability for negligence.

1.8.5 The Investigator, or a person designated by the Investigator, should fully inform the subject or, if the subject is unable to provide informed consent, the subject's legally acceptable representative, of all pertinent aspects of the study including the written information given approval/favourable opinion by the MREC and LREC.

1.8.6 The language used in the oral and written information about the study, including the written informed consent form, should be as non-technical as practical and should be understandable to the subject or the subject's legally acceptable representative and the impartial witness, where applicable.

1.8.7 Before informed consent may be obtained, the Investigator, or a person designated by the Investigator, should provide the subject or the subject's legally acceptable representative ample time and opportunity to inquire about details of the study and to decide whether or not to participate in the study. All questions about the study should be answered to the satisfaction of the subject or the subject's legally acceptable representative.

1.8.8 Prior to a subject's participation in the study, the written informed consent form should be signed and personally dated by the subject or by the subject's legally acceptable representative, and by the person who conducted the informed consent discussion.

1.8.9 If a subject is unable to read or if a legally acceptable representative is unable to read, an impartial witness should be present during the entire informed consent discussion. After the written informed consent form and any other written information to be provided to subjects, is read and explained to the subject or the subject's legally acceptable representative, and after the subject's legally acceptable representative has orally consented to the subject's participation in the study and, if capable of doing so, has signed and personally dated the informed consent form, the witness should sign and personally date the consent form. By signing the consent form, the witness attests that the information in the consent form and any other written information was accurately explained to, and apparently understood by, the subject or the subject's legally acceptable representative, and that informed consent was freely given by the subject or the subject's legally acceptable representative.

1.8.10 Both the informed consent discussion and the written informed consent form and any other written information to be provided to subjects should include explanations of the following:

a) That the study involves research.

b) The purpose of the study.

c) The study treatment(s) and the probability for random assignment to each treatment.

d) The study procedures to be followed, including all invasive procedures.

e) The subject's responsibilities.

f) Those aspects of the study that are experimental.

g) The reasonably foreseeable risks or inconveniences to the subject and, when applicable, to an embryo, fetus, or nursing infant.

h) The reasonably expected benefits. When there is no intended clinical benefit to the subject, the subject should be made aware of this.

i) The alternative procedure(s) or course(s) of treatment that may be available to the subject, and their important potential benefits and risks.

j) The compensation and/or treatment available to the subject in the event of study-related injury.

k) The anticipated prorated payment, if any, to the subject for participating in the study.

l) The anticipated expenses, if any, to the subject for participating in the study.

m) That the subject's participation in the study is voluntary and that the subject may refuse to participate or withdraw from the study, at any time, without penalty or loss of benefits to which the subject is otherwise entitled.

n) That the monitor(s), the auditor(s), the MREC and LREC, and the regulatory authority(ies) will be granted direct access to the subject's original medical records for verification of clinical study procedures and/or data, without violating the confidentiality of the subject, to the extent permitted by the applicable laws and regulations and that, by signing a written informed consent form, the subject or the subject's legally acceptable representative is authorising such access.

o) That records identifying the subject will be kept confidential and, to the extent permitted by the applicable laws and/or regulations, will not be made publicly available. If the results of the study are published, the subject's identity will remain confidential.

p) That the subject or the subject's legally acceptable representative will be informed in a timely manner if information becomes available that may be relevant to the subject's willingness to continue participation in the study.

q) The person(s) to contact for further information regarding the study and the rights of study subjects, and whom to contact in the event of study-related injury.

r) The foreseeable circumstances and/or reasons under which the subject's participation in the study may be terminated.

s) The expected duration of the subject's participation in the study.

t) The approximate number of subjects involved in the study.

1.8.11 Prior to participation in the study, the subject or the subject's legally acceptable representative should receive a copy of the signed and dated written informed consent form and any other written information provided to the subjects. During a subject's participation in the study, the subject or the subject's legally acceptable representative should receive a copy of the signed and dated consent form updates and a copy of any amendments to the written information provided to subjects.

1.8.12 When a clinical study (therapeutic or non-therapeutic) includes subjects who can only be enrolled in the study with the consent of the subject's legally acceptable representative (e.g., minors, or patients with severe dementia), the subject should be informed about the study to the extent compatible with the subject's understanding and, if capable, the subject should sign and personally date the written informed consent.

1.8.13 Except as described in 1.8.14, a non-therapeutic study (i.e. a study in which there is not anticipated direct clinical benefit to the subject), should be conducted in subjects who personally give consent and who sign and date the written informed consent form.

1.8.14 Non-therapeutic studies may be conducted in subjects with consent of a legally acceptable representative provided the following conditions are fulfilled:

a) The objectives of the study can not be met by means of a study in subjects who can give informed consent personally.

b) The foreseeable risks to the subjects are low.

c) The negative impact on the subject's well-being is minimised and low.

d) The study is not prohibited by law.

e) The approval/favourable opinion of the MREC and LREC is expressly sought on the inclusion of such subjects, and the written approval/favourable opinion covers this aspect.

Such studies, unless an exception is justified, should be conducted in patients having a disease or condition for which the investigational product is intended. Subjects in these studies should be particularly closely monitored and should be withdrawn if they appear to be unduly distressed.

1.8.15 In emergency situations, when prior consent of the subject is not possible, the consent of the subject's legally acceptable representative, if present, should be requested. When prior consent of the subject is not possible, and the subject's legally acceptable representative is not available, enrolment of the subject should require measures described in the protocol and/or elsewhere, with documented approval/favourable opinion by the MREC and LREC, to protect the rights, safety and well-being of the subject and to ensure compliance with applicable regulatory requirements. The subject or the subject's legally acceptable representative should be informed about the study as soon as possible and consent to continue and other consent as appropriate (see 1.8.10) should be requested.

1.9 Records and Reports

1.9.1 The Investigator should ensure the accuracy, completeness, legibility, and timeliness of the data reported to the sponsor in the CRFs and in all required reports.

1.9.2 Data reported on the CRF, that are derived from source documents, should be consistent with the source documents or the discrepancies should be explained.

1.9.3 Any change or correction to a CRF should be dated, initialled, and explained (if necessary) and should not obscure the original entry (i.e. an audit study should be maintained): this applies to both written and electronic changes or corrections (see 1.18.4 (n)). Sponsors should provide guidance to Investigators and/or the Investigators'

designated representatives on making such corrections. Sponsors should have written procedures to assure that changes or corrections in CRFs made by sponsor's designated representatives are documented, are necessary, and are endorsed by the Investigator. The Investigator should retain records of the changes and corrections.

1.9.4 The Investigator should maintain the study documents as specified in Essential Documents for the Conduct of a Clinical Study and as required by the applicable regulatory requirement(s). The Investigator should take measures to prevent accidental or premature destruction of these documents.

1.9.5 Essential documents should be retained until at least 2 years after the last approval of a marketing application in an ICH region and until there are no pending or contemplated marketing applications in an ICH region or at least 2 years have elapsed since the formal discontinuation of clinical development of the investigational product. These documents should be retained for a longer period however if required by the applicable regulatory requirements or by an agreement with the sponsor. It is the responsibility of the sponsor to inform the Investigator as to when these documents no longer need to be retained.

1.9.6 The financial aspects of the study should be documented in an agreement between the sponsor and the Investigator.

1.9.7 Upon request of the monitor, auditor, MREC, LREC, or regulatory authority, the Investigator should make available for direct access all requested study-related records.

1.10 Progress Reports

1.10.1 The Investigator should submit written summaries of the study status to the LREC annually, or more frequently, if requested by the LREC.

1.10.2 The Investigator should promptly provide written reports to the sponsor, the LREC and, where applicable, the institution on any changes significantly affecting the conduct of the study, and/or increasing the risk to subjects.

1.11 Safety Reporting

1.11.1 All serious adverse events (SAEs) should be reported immediately to the sponsor except for those SAEs that the protocol or other document (e.g., Investigator's Brochure) identifies as not needing immediate reporting. The immediate reports should be followed promptly by detailed, written reports. The immediate and follow-up reports should identify subjects by unique code numbers assigned to the study subjects rather than by the subjects' names, personal identification numbers, and/or addresses. The Investigator should also comply with the applicable regulatory requirement(s) related to the reporting of unexpected serious adverse drug reactions to the regulatory authority(ies) and the LREC.

1.11.2 Adverse events and/or laboratory abnormalities identified in the protocol as critical to safety evaluations should be reported to the sponsor according to the reporting requirements and within the time periods specified by the sponsor in the protocol.

1.11.3 For reported deaths, the Investigator should supply the sponsor and the LREC with any additional requested information (e.g., autopsy reports and terminal medical reports).

1.12 Premature Termination or Suspension of a Study

If the study is prematurely terminated or suspended for any reason, the Investigator should promptly inform the study subjects, should assure appropriate therapy and follow-up for the subjects, and, where required by the applicable regulatory requirement(s), should inform the regulatory authority(ies). In addition:

1.12.1 If the Investigator terminates or suspends a study without prior agreement of the sponsor, the Investigator should inform the institution where applicable, and the Investigator should promptly inform the sponsor and the LREC, and should provide the sponsor and the LREC a detailed written explanation of the termination or suspension.

1.12.2 If the sponsor terminates or suspends a study, the Investigator should promptly inform the institution where applicable and the Investigator should promptly inform the LREC and provide the LREC a detailed written explanation of the termination or suspension.

1.12.3 If the MREC or LREC terminates or suspends its approval/favourable opinion of a study, the Investigator should inform the institution where applicable and the Investigator should promptly notify the sponsor and provide the sponsor with a detailed written explanation of the termination or suspension.

1.13 Final Report(s) by Investigator

Upon completion of the study, the Investigator, where applicable, should inform the institution: the Investigator should provide the LREC with a summary of the study's outcome, and the regulatory authority(ies) with any reports required.

APPENDIX 14: DECLARATION OF HELSINKI

Recommendations guiding physicians in biomedical research involving human subjects

Adopted by the 18th World Medical Assembly, Helsinki, Finland, June 1964 and amended in Tokyo, 1975, in Venice, 1983, in Hong Kong, 1989, and in South Africa, October 1996.

Introduction

It is the mission of the physician to safeguard the health of the people. His or her knowledge and conscience are dedicated to the fulfilment of this mission.

The Declaration of Geneva of the World Medical Association binds the physician with the words, "The health of my patient will be my first consideration", and the International Code of Medical Ethics declares that, "A physician shall act only in the patient's interest when providing medical care which might have the effect of weakening the physical and mental condition of the patient".

The purpose of biomedical research involving human subjects must be to improve diagnostic, therapeutic and prophylactic procedures and the understanding of the aetiology and pathogenesis of disease.

In current medical practice most diagnostic, therapeutic or prophylactic procedures involve hazards. This applies especially to biomedical research.

Medical progress is based on research which ultimately must rest in part on experimentation involving human subjects.

In the field of biomedical research a fundamental distinction must be recognised between medical research in which the aim is essentially diagnostic or therapeutic for a patient, and medical research, the essential object of which is purely scientific and without implying direct diagnostic or therapeutic value to the person subjected to the research.

Special caution must be exercised in the conduct of research which may affect the environment, and the welfare of animals used for research must be respected.

Because it is essential that the results of laboratory experiments be applied to human beings to further scientific knowledge and to help suffering humanity, the World Medical Association has prepared the following recommendations as a guide to every physician in biomedical research involving human subjects. They should be kept under review in the future. It must be stressed that the standards as drafted are only a guide to physicians all over the world. Physicians are not relieved from criminal, civil and ethical responsibilities under the laws of their own countries.

I. Basic principles

1. Biomedical research involving human subjects must conform to generally accepted scientific principles and should be based on adequately performed formed laboratory and animal experimentation and on a thorough knowledge of the scientific literature.

2. The design and performance of each experimental procedure involving human subjects should be clearly formulated in an experimental protocol which should be transmitted for consideration, comment and guidance to a specially appointed committee independent of the investigator and the sponsor provided that this independent committee is in conformity with the laws and regulations of the country in which the research experiment is performed.

3. Biomedical research involving human subjects should be conducted only by scientifically qualified persons and under the supervision of a clinically competent medical person. The responsibility for the human subject must always rest with a medically qualified person and never rest on the subject of the research, even though the subject has given his or her consent.

4. Biomedical research involving human subjects cannot legitimately be carried out unless the importance of the objective is in proportion to the inherent risk to the subject.

5. Every biomedical research project involving human subjects should be preceded by careful assessment of predictable risks in comparison with foreseeable benefits to the subject or to others. Concern for the interests of the subject must always prevail over the interests of science and society.

6. The right of the research subject to safeguard his or her integrity must always be respected. Every precaution should be taken to respect the privacy of the subject and to minimise the impact of the study on the subject's physical and mental integrity and on the personality of the subject.

7. Physicians should abstain from engaging in research projects involving human subjects unless they are satisfied that the hazards involved are believed to be predictable. Physicians should cease any investigation if the hazards are found to outweigh the potential benefits.

8. In publication of the results of his or her research, the physician is obliged to preserve the accuracy of the results. Reports of experimentation not in accordance with the principles laid down in this Declaration should not be accepted for publication.

9. In any research on human beings, each potential subject must be adequately informed of the aims, methods, anticipated benefits and potential hazards of the study and the discomfort it may entail. He or she should be informed that he or she is at liberty to abstain from participation in the study and that he or she is free to withdraw his or her consent to participation at any time. The physician should then obtain the subject's freely given informed consent, preferably in writing.

10. When obtaining informed consent for the research project the physician should be particularly cautious if the subject is in a dependent relationship to him or her or may consent under duress. In that case the informed consent should be obtained by a physician who is not engaged in the investigation and who is completely independent of this official relationship.

11. In the case of legal incompetence, informed consent should be obtained from the legal guardian in accordance with national legislation. Where physical or mental incapacity makes it impossible to obtain informed consent, or when the subject is a minor, permission from the responsible relative replaces that of the subject in accordance with national legislation.

Whenever the minor child is in fact able to give a consent, the minor's consent must be obtained in addition to the consent of the minor's legal guardian.

12. The research protocol should always contain a statement of the ethical considerations involved and should indicate that the principles enunciated in the present Declaration are complied with.

II. Medical research combined with professional care (clinical research)

1. In the treatment of the sick person, the physician must be free to use a new diagnostic and therapeutic measure, if in his or her judgement it offers hope of saving life, re-establishing health or alleviating suffering.

2. The potential benefits, hazards and discomfort of a new method should be weighed against the advantages of the best current diagnostic and therapeutic methods.

3. In any medical study, every patient - including those of a control group, if any - should be assured of the best proven diagnostic and therapeutic method. This does not exclude the use of inert placebo in studies where no proven diagnostic or therapeutic method exists.

4. The refusal of the patient to participate in a study must never interfere with the physician-patient relationship.

5. If the physician considers it essential not to obtain informed consent, the specific reasons for this proposal should be stated in the experimental protocol for transmission to the independent Committee.

6. The physician can combine medical research with professional care, the objective being the acquisition of new medical knowledge, only to the extent that medical research is justified by its potential diagnostic or therapeutic value for the patient.

III. Non therapeutic biomedical research involving human subjects (non-clinical biomedical research)

1. In the purely scientific application of medical research carried out on a human being, it is the duty of the physician to remain the protector of the life and health of that person on whom biomedical research is being carried out.

2. The subjects should be volunteers - either healthy persons or patients for whom the experimental design is not related to the patient's illness.

3. The investigator or the investigating team should discontinue the research if in his/her or their judgement it may, if continued, be harmful to the individual. . In research on man, the interest of science and society should never take precedence over considerations related to the well-being of the subject.

PHASE 2
SAFETY

Patient invited to take part in Phase 2 if completed Phase 1

INFUSIONS 31 etc.
(until product marketed or withdraw for other reasons)

- Repeated weekly infusions of study SCIG
- Dose titrated to suit patient - minimum target 6g/l
- Clinic visits at 3 monthly intervals
- Patients complete weekly diary cards

PHARMACOKINETICS

Selected patients attend clinic for pharmacokinetic evaluation

VISITS PK1 a-g & PK 2 a-g

- Daily blood sampling for 1 week post 1st dose of SCIG - visits PK 1 a-g
- Repeated for a further week 3 months later - visits PK 2 a-g

APPENDIX 16: STUDY PROCEDURES

	PHASE 1				PHASE 2	PHARMACOKINETICS	
	**Pre-study**	**Infusions 1-3 Routine IVIG/SCIG**	**Infusions 4-30 SCIG**	**End of Study**	**3 monthly SCIG**	**PK 1 a-g SCIG**	**PK 2 a-g SCIG**
Informed Consent	X						
Inclusion /Exclusion Criteria	X						
Medical History	X						
Physical Examination	X			X			
Vital Signs	X	X	X	X		X	X
Patient Satisfaction Questionnaire	X		$X^{inf\ 18}$	X			
Site Inspection		X	X			X	X
Injection training/check technique			X				
**Haematology**: Haemoglobin, Haematocrit, RBC, WBC, Neutrophils, Lymphocytes, Monocytes, Eosinophils, Basophils, Platelet Count, Reticulocytes	X	if clinically indicated	if clinically indicated	X	if clinically indicated	if clinically indicated	if clinically indicated
**Biochemistry**: Sodium, Potassium, Creatinine, LDH, GGT, Total Bilirubin, Alkaline Phosphatase, Haptoglobin	X	if clinically indicated	if clinically indicated	X	if clinically indicated	if clinically indicated	if clinically indicated
**Blood Group Serology**: ABO (D), Direct Coombs	X						
**Immunology**: IgG, IgA, IgM	X	X	X	X	if clinically indicated	X	X
**Liver Function**: ALT (and/or AST)			X*				
**Specific Antibodies**: Pneumococcus, HIB	X		X*				
**Virology**:HBsAg, HIV adults only(PCR), HCV (PCR)			X**$^{inf\ 4}$	X^{π}	X^{ϕ}		
**Parvovirus**: Parvovirus B19 (PCR)			$X^{\propto}$				
**Archive**: Store at -70°C for 15 years			$X^{inf\ 4}$	X^{π}	X^{ϕ}		
**Repeat Test** : Store at -20°C for repeat testing	X	X	X	X	XΨ	X	X

* 4 weekly samples
** Baseline sample stored at -70°C and tested only if end of study sample positive
Ψ Repeat sample to be taken only if other samples clinically indicated

π Sample to be taken only if patient withdraws from Phase 1 or **not** entering Phase 2
ϕ Sample to be taken if patient withdraws or completes Phase 2
∝ Pre 1st dose &1 week post 1st dose

A MULTI-CENTRE, OPEN STUDY TO ASSESS THE SAFETY AND EFFICACY OF A 16% IMMUNOGLOBULIN PRODUCT GIVEN VIA THE SUBCUTANEOUS ROUTE IN PRIMARY ANTIBODY DEFICIENT PATIENTS

SCIG01

FINAL PROTOCOL

VERSION 3

APRIL 2000

BIO PRODUCTS LABORATORY
DAGGER LANE
ELSTREE
HERTS WD6 3BX

Tel: 020 8258 2200
Fax: 020 8258 2611

A MULTI-CENTRE, OPEN STUDY TO ASSESS THE SAFETY AND EFFICACY OF A 16% IMMUNOGLOBULIN PRODUCT GIVEN VIA THE SUBCUTANEOUS ROUTE IN PRIMARY ANTIBODY DEFICIENT PATIENTS

SCIG01

FINAL PROTOCOL
VERSION 3

APRIL 2000

MEDICAL DIRECTOR: Dr Clive H Dash
Bio Products Laboratory
Dagger Lane
Elstree
Herts WD6 3BX
Tel: 020 8258 2565
Fax: 020 8258 2611

TABLE OF CONTENTS

1.0 LIST OF ABBREVIATIONS 1

2.0 SYNOPSIS 2

3.0 INTRODUCTION 4

4.0 STUDY OBJECTIVES 6

5.0 STUDY DESIGN 6

6.0 STUDY POPULATION 6

- 6.1 Source of Subjects 6
- 6.2 Inclusion Criteria 6
- 6.3 Exclusion Criteria 7

7.0 STUDY DRUGS 7

- 7.1 Presentation 7
- 7.2 Dose and route of administration 8
- 7.3 Storage 9
- 7.4 Concomitant Medication 10
- 7.5 Drug Accountability 11

8.0 STUDY SCHEDULE - PHASE 1 11

- 8.1 Pre-study Procedures 11
- 8.2 STUDY PROCEDURES 12
  - 8.2.1 Clinical Monitoring during Infusion 12
  - 8.2.2 Laboratory Measurements 13
  - 8.2.3 Laboratory Sampling in Children 14
- 8.3 End of Phase 1 Procedures 15
- 8.4 Blood Sample Collection, Handling and Labelling 16

9.0 HOME THERAPY PROCEDURES 17

- 9.1 Criteria for Home Therapy 17
- 9.2 Home Therapy Training 17
- 9.3 Clinical Monitoring during Home Therapy 18
- 9.4 Blood Sampling during Home Therapy 18

10.0 STUDY SCHEDULE - PHASE 2 19

11.0 STUDY SCHEDULE - PHARMACOKINETICS 20

- 11.1 Pharmacokinetic Procedures 20

12.0 EFFICACY MEASURES 20

- 12.1 Primary Efficacy Measurement 20
- 12.2 Secondary Efficacy Measurements 20

13.0 SAFETY MEASUREMENTS 21

14.0 WITHDRAWAL OF SUBJECTS 21

15.0 ADVERSE EVENTS 22

15.1 Definition of Serious Adverse Events 22
15.2 Detecting Adverse Events 23
15.3 Completing Adverse Event Forms 23
15.4 Assessment of adverse event intensity 23
15.5 Attribution to study drugs 24
15.6 Reporting Adverse Events 25
15.7 Adverse Event Follow-up 26
16.0 DATA ANALYSIS 26
16.1 Statistical Analysis 26
16.2 Definition of Evaluability 27
16.3 Dropouts 27
16.4 Interim Analysis 28
17.0 DOCUMENTATION 28
17.1 Required Pre-Study Documentation 28
17.2 Recording data in Case Record Forms 29
17.3 Error Correction 29
17.4 Signing Off and Return of Case Record Forms 30
17.5 Maintenance and Archiving of Study Records 30
18. STUDY CONDUCT 32
18.1 Adherence to the Protocol 32
18.2 Protocol amendments 32
18.3 Early Cessation of the Study 33
18.4 Monitoring Visits and Audit 33
18.5 Report and Publication 34
18.6 Obligations of the Investigator 35
18.7 Confidentiality 35
19.0 ETHICAL CONSIDERATIONS 35
19.1 CONSENT PROCEDURE FOR ADULTS 36
19.2 CONSENT PROCEDURE FOR CHILDREN 36
19.3 Compensation/Indemnity 37
21.0 REFERENCES 39
APPENDIX 1: ADMINISTRATION OF SUBCUTANEOUS INFUSION 40
APPENDIX 2 ADULT PATIENT INFORMATION LEAFLET 41
APPENDIX 3: PARENT/GUARDIAN INFORMATION SHEET 44
APPENDIX 4: CHILDREN'S INFORMATION LEAFLET 47
APPENDIX 5 : ADULT CONSENT FORM 49
APPENDIX 6: CHILDREN'S CONSENT FORM 50
APPENDIX 7: PATIENT INFORMATION LEAFLET FOR EXTRA BLOOD TESTS (PHARMACOKINETICS) 51
APPENDIX 8: PHARMACOKINETICS CONSENT FORM 52
APPENDIX 9: EXAMPLE GP LETTER 53

APPENDIX 10: HOME THERAPY MANUAL 54
APPENDIX 11: HOME THERAPY CONSENT FORM 56
APPENDIX 12: HOME THERAPY INFUSION DIARY CARD 57
APPENDIX 13: LOCAL INVESTIGATOR'S RESPONSIBILITIES BASED ON THE ICH GUIDELINES FOR GOOD CLINICAL PRACTICE 58
APPENDIX 14: DECLARATION OF HELSINKI 69
APPENDIX 15: STUDY PROCEDURES 74

1.0 LIST OF ABBREVIATIONS

AE	Adverse Event
ADR	Adverse Reaction
ALT	Alanine aminotransferase
AST	Aspartate aminotransferase
BPL	Bio Products Laboratory
CPMP	Committee on Proprietary Medicinal Products
CRF	Case Record Form
CRA	Clinical Research Associate
CTX	Clinical Trial Exemption
CV	Curriculum Vitae
EC	Ethics Committee
GCP	Good Clinical Practice
GGT	Gamma glutamyl transferase
GP	General Practitioner
HbsAg	Hepatitis B surface antigen
HCV	Hepatitis C virus
HIB	Haemophilus Influenzae type B
HIV	Human Immunodeficiency Virus
IgA	Immunoglobulin A
IgG	Immunoglobulin G
IgM	Immunoglobulin M
iu	International Units
ICH	International Conference on Harmonisation
IVIG	Intravenous immunoglobulin
LDH	Lactate dehydrogenase
LREC	Local Research Ethics Committee
MCA	Medicines Control Agency
MREC	Multicentre Research Ethics Committee
PCR	Polymerase Chain Reaction
RBC	Red Blood Cell
SAE	Serious Adverse Event
SCIG	Subcutaneous Immunoglobulin
SOP	Standard Operating Procedure
WBC	White Blood Cell

2.0 SYNOPSIS

A multi-centre, open study to assess the safety and efficacy of a 16% immunoglobulin product given via the subcutaneous route in primary antibody deficient patients.

A total of 40 patients (paediatric 0 – 15 years, adults 16+ years), stable on intravenous immunoglobulin therapy (IVIG) or subcutaneous immunoglobulin therapy (SCIG), will be enrolled.

Patients will be eligible to enter the study if, in the investigator's opinion, their disease is stable. In order to collect baseline data, patients will continue to receive their usual immunoglobulin, whether IVIG or SCIG, for 3 infusions before commencing the study subcutaneous immunoglobulin. Starting one week after their final treatment they will receive the 16% immunoglobulin product subcutaneously at a dose of 100mg/kg bodyweight. Weekly infusions will then continue to be administered for a period of 6 months. The dose and frequency of the 16% immunoglobulin product given, in order to maintain an adequate serum IgG level, will be tailored to suit the patient. This will be decided by the investigator, but a minimum target IgG trough level of 4g/L for paediatrics and 6g/Lfor adults will be aimed for.

There will be provision for patients to administer SCIG at home, if appropriate, after a period of training. These patients will be required to complete diary cards during each home infusion and to attend the clinic at 1-4 weekly intervals for blood sampling.

After 6 months of SCIG treatment (Phase 1), patients will then either return to their usual immunoglobulin treatment or continue with the 16% immunoglobulin in Phase 2 of the study which is a safety follow-on phase.

During Phase 2, patients will be asked to complete regular diary cards and return for clinic visits at 3 monthly intervals.

Patients will attend the clinic at the following time-points:-

STUDY PHASE	VISIT/INFUSION NUMBER	VISIT DETAILS
**Phase 1**<br>**(CRF 1)**	Prestudy	Up to 28 days prior to study entry
	Infusions 1-3	3 infusions of patient's routine immunoglobulin at usual frequency and dose prior to study SCIG to collect baseline data
**Phase 1**<br>**(CRF 2)**<br><br>**Pharmacokinetics**	Infusions 4 -30<br><br>PK 1 (optional)<br>PK 2 (optional)	Selected patients will be asked to attend for 5 consecutive clinic visits after first study SCIG infusion. This will be repeated after 3 months of SCIG
**Phase 2**<br>**(CRF 3)**	Infusions 31 etc<br>(until product is marketed)	Patients will be invited to take part in the safety follow-on phase. They will receive weekly SCIG infusions, complete regular diary cards and attend the clinic at 3 monthly intervals.

Primary Efficacy Measurement:

- The proportion of trough levels at each time point where the IgG $\geq$ 4g/L

Secondary Efficacy Measurements:

- The change in SCIG dose required in order to maintain trough

levels at a minimum of 6g/L. The number of occasions when the dose is adjusted, the size of the dose change and the overall range of dose changes will be calculated.

- The proportion of trough levels at each time point where the IgG $\geq$ 6g/L.
- The time taken for each patient to reach a steady state IgG trough level. This will be defined as when three consecutive occasions occur when the IgG trough levels are within 1g/L of each other.
- The mean change in IgG trough level as compared to the baseline level at each time point.
- The overall number of infections occurring and days off work/school.

Safety Measurements:

- Number, type, severity and duration of adverse events occurring throughout the study.
- Laboratory monitoring of haematology, biochemistry and immunological and viral markers
- Monitoring of vital signs

Patient/parent/guardian satisfaction of the subcutaneous route will be assessed by way of a short questionnaire at the beginning, middle and end of treatment.

3.0 INTRODUCTION

Patients who are diagnosed with primary antibody deficiency usually require regular immunoglobulin replacement therapy in order to avoid serious infection. The efficacy of life-long intravenous immunoglobulin (IVIG) therapy is well established and is a major contributor to improved health and quality of life for these patients[1]. However, IVIG treatment for most patients requires regular visits to hospital where the infusion can take many hours to administer depending on the dose and tolerance to the treatment.

Intramuscular and slow subcutaneous infusions of immunoglobulins are alternatives to IVIG but are not without limitations. Intramuscular injections are often painful, the dose that can be safely delivered is limited and the side effects can be severe. Slow subcutaneous infusions, on the other hand, while benefiting from a better safety profile, are time-consuming and cumbersome usually requiring overnight infusions[2].

Rapid subcutaneous infusions of immunoglobulin preparations intended for intramuscular use were first described in 1991 by Gardulf et al[3]. Patients were given simultaneous infusions totaling 34-40ml/hr via dual portable pumps. In addition to there being very few (0.93%) mild systemic reactions experienced with this method of administration, patients were also able to infuse the immunoglobulin at home after a period of training.

BPL's Human Normal Immunoglobulin is mainly IgG and is indicated for prophylaxis against hepatitis A infection and replacement therapy in primary antibody deficiency. It is manufactured from venous plasma and is currently licensed for intramuscular use. This study will use an almost identical product, obtained from the plasma of screened donors in the United States. However, the composition of this 16% immunoglobulin product is such that it would also be suitable for subcutaneous use since it is virtually identical to the product used routinely in Scandinavia[3,4], Kabiglobulin. Both products are approximately 16% immunoglobulin, mercury-free and have a very low IgA content, which contributes to their subcutaneous tolerance. In addition, they have a solvent/detergent step included in the manufacturing process which inactivates lipid-enveloped viruses such as HIV, hepatitis C (HCV) and hepatitis B (HBV). To date there have been no reports of these viruses being transmitted by BPL's intramuscular product.

The following study has therefore been designed to assess the efficacy and safety of giving a 16% immunoglobulin product via rapid subcutaneous infusion to patients with primary antibody deficiency.

4.0 STUDY OBJECTIVES

1. To determine the efficacy of giving patients with primary antibody deficiency, weekly subcutaneous infusions of a 16% immunoglobulin product at a dose tailored to achieve a minimum trough level of IgG of 4g/L for paediatrics and 6g/L for adults.

5.0 STUDY DESIGN

A multi-centre, open, safety and efficacy study of weekly subcutaneous 16% immunoglobulin.

6.0 STUDY POPULATION

6.1 Source of Subjects

The trial will be conducted as a multi-centre study. Up to a total of 40 adult and paediatric patients with a diagnosis of primary antibody deficiency will be enrolled. This number will allow for drop-outs and withdrawals. Patients can either be receiving IVIG or SCIG as their normal replacement therapy before entry into the study and a minimum number of 10 of each type will be aimed for in order for an adequate number to be analysed.

6.2 Inclusion Criteria

a) Diagnosis of primary antibody deficiency syndrome

b) Aged 0 - no upper age limit

c) Stable disease and has been receiving immunoglobulin (IVIG or SCIG) for the past 6 months

d) Written informed consent (patient/parent/guardian).

6.3 Exclusion Criteria

a) Known to be intolerant to IgA

b) Pregnant women or women who are breast feeding

c) History of clinically significant renal or hepatic disease or known renal or hepatic abnormalities

d) Unsuitable for the purposes of the study (in the opinion of the investigator)

e) Participation in another clinical trial within the last 30 days

f) History of allergic reactions to intravenous blood products

g) History of infection within the last 2 months, requiring IV antibiotics

h) At the beginning of the study, is known to require treatment with another blood product during the course of the study.

7.0 STUDY DRUGS

7.1 Presentation

The immunoglobulin used in this study is a liquid preparation containing 16% immunoglobulin, mainly immunoglobulin G (IgG, gammaglobulin).

The product is presented in a 5ml vial containing 750mg of protein. If available, 10ml vials containing 1500mg of protein will also be used.

1ml of solution contains the following approximate amounts:

Human protein (at least 95% IgG)	140-180mg/ml
Sodium Chloride	9mg/ml
Glycine	6mg/ml
Sodium Acetate	2mg/ml

The product is stored in containers which are drawn vials of neutral borosilicate glass with a 13mm neck. The closure is a 13mm diameter overseal, consisting of a snap-off polypropylene cap, a clear lacquered aluminium skirt and a halobutyl rubber wad.

7.2 Dose and route of administration

Patients enrolled will continue to receive their usual immunoglobulin infusion at the same dose and frequency for 3 treatments prior to receiving study SCIG in order for baseline data to be collected. However, where patients are currently receiving IVIG and are needing to change rapidly to SCIG due to poor venous access, a minimum of 1 baseline IVIG treatment would be acceptable. If patients are currently receiving SCIG they will not be required to undergo any IVIG treatments and can commence SCIG with the study product once they have completed three subcutaneous infusions of their usual immunoglobulin.

Once study product is commenced, the 16% immunoglobulin will be given by subcutaneous infusion via a portable syringe driver (Graseby, MS16A) at an initial starting dose of 100mg/kg bodyweight. Subsequent SCIG infusions will continue to be administered at weekly intervals for a period of 6 months and the dose will be tailored to suit the patient's trough levels of IgG. The dose and frequency of the 16% immunoglobulin product given, in order to maintain an adequate serum IgG level, will be decided by the investigator but a minimum target IgG trough level of 4g/L for paediatrics and 6g/L for adults will be aimed for.

The 16% immunoglobulin product should be given by separate infusion and not mixed with other infusion material.

For an adult, the first SCIG infusion should be given at a slow rate of 10mls/hr increasing over the next 4-8 infusions by 1-2mls/hr until the recommended maximum rate of 20mls/hr is reached. Two sites may be simultaneously used at this rate, via 2 syringe drivers, giving a combined maximum rate of 40mls/hr.

For a child, the first infusion should be given at a slow rate of 5mls/hr increasing by 0.5-1mls/hr over the next 4-8 infusions until the recommended maximum rate of 10mls/hr is reached. Two sites may be simultaneously used at this rate, via 2 syringe drivers, giving a combined maximum rate of 20mls/hr.

Either the abdomen or the thigh can be used as the site of infusion for both adults and children. Most patients seem to prefer abdominal siting of the infusions although very young children tolerate the infusions better in the thigh than in the abdomen.

Instructions for administration of subcutaneous immunoglobulin can be found in Appendix 1.

7.3 Storage

The 16% immunoglobulin product should be stored in its carton in the dark and has a shelf life of 2 years if stored between 2-8°C or 1 week if stored at 25°C. DO NOT FREEZE.

The 16% immunoglobulin product is for single use only; any used materials and unused solution should be discarded by approved means.

The condition of date-expired or incorrectly stored product cannot be guaranteed. Such product may be unsafe and should not be used. Solutions, which are cloudy or have deposits should not be used.

The dose volume for each vial size is specified on the label.

The 16% immunoglobulin product for clinical trial use should be stored separately from routine product, as product reconciliation has to be done in accordance with the ICH guidelines for GCP.

7.4 Concomitant Medication

The 16% immunoglobulin product is believed not to affect the immune response to bacterial vaccines but could reduce the response to some virus vaccines and toxoids. However it should be noted that attempts to vaccinate patients with primary antibody deficiency may not be completely effective even if immunoglobulin has not been administered.

With administration of the 16% immunoglobulin product, a broad spectrum of antibodies is passively administered. These antibodies will interfere with the response to live vaccines, especially the MMR (measles, mumps, rubella) vaccine and varicella vaccine. Such vaccines should therefore be given at least 3 weeks before or 3 months after administration of immunoglobulin. This does not apply to yellow fever vaccine however since the immunoglobulin product obtained from US plasma is unlikely to contain antibody to this virus.

All medications taken by the patient or administered to the patient during the study will be recorded as concomitant medication. In addition, any drugs prescribed by the investigator or the patient's G.P. throughout the trial will also be regarded as concomitant medication.

The following information on all concomitant medication must be recorded in the CRF:-

- the name of the drug and its pharmaceutical form
- the reason for treatment
- the dose
- the duration of treatment

7.5 Drug Accountability

It is essential that accurate drug accountability records are maintained to ensure that the total number of 16% immunoglobulin product vials dispatched, received and returned by a study site can be established.

This is facilitated by completion of dispatch notes (by BPL), receipt forms, stock control logs, dispensing/returns logs and returns forms (by Pharmacy or equivalent) and the recording at each infusion of the batch number and number of grams and vials given in the patient's notes and CRF (investigator or appointed personnel).

The 16% immunoglobulin product should be used solely as indicated in the protocol and must not be used on patients not in the trial without the prior agreement of BPL.

8.0 STUDY SCHEDULE - PHASE 1

8.1 Pre-study Procedures

Prior to enrolling a patient, the investigator should ensure that the patient fulfills the inclusion/exclusion criteria.

Potential subjects for the study, or their parent/guardian, will be given a detailed, oral presentation of the nature, purpose, risks and requirements of the study, in addition to receiving detailed information. They will be given adequate time to consider participating in the study and the opportunity to ask the study physician about any aspect of the study. Once satisfied, the patient/parent/guardian will be asked to sign a Consent Form. A letter will be sent to the patient's G.P.

Subsequently, the patient will be allocated a Subject Number, provided by BPL, for identification purposes and then undergo screening, which

will take place no more than 28 days prior to Infusion 1. The pre-study assessment will consist of:

- Medical history
- Physical examination including height, weight, sitting blood pressure, pulse, temperature and respiration
- Concomitant medication
- Patient Satisfaction Questionnaire
- Blood samples as detailed in the following table:

**Haematology**	Haemoglobin, Haematocrit, RBC, WBC, Neutrophils, Lymphocytes, Monocytes, Eosinophils, Basophils, Platelet Count, Reticulocytes
**Blood Group Serology**	ABO (D),<br>Direct Coombs' Test
**Biochemistry**	Sodium, Potassium, Creatinine, LDH, ALT (and/or AST), GGT, Total Bilirubin, Alkaline Phosphatase, Haptoglobin
**Immunology**	IgG, IgA, IgM
**Specific Antibodies**	Pneumococcus, HIB
**Repeat Test - Stored**	Separate serum aliquot to be stored at -20°C in case of repeat testing

8.2 STUDY PROCEDURES

8.2.1 Clinical Monitoring during Infusion

Vital signs (body temperature, respiration, pulse and sitting blood pressure) will be recorded prior to each infusion, at hourly intervals during the infusion and again at the end. The infusion site(s) will be inspected during and at the end of the infusion for signs of irritation. Any untoward irritation and/or swelling will be recorded as an adverse event in the CRF. Adverse events and any changes in concomitant medication since the last infusion will be recorded in the CRF. The

patient/parent/guardian will also be asked to complete a Patient Satisfaction Questionnaire at approximately week 12.

8.2.2 Laboratory Measurements

The following immunology samples will be taken immediately prior to each infusion throughout Phase 1. In addition, for patients on IVIG, a post-infusion sample will be taken immediately after the last routine treatment:

**Immunology**	IgG, IgA, IgM

Once the patient has completed all routine treatments, the following baseline samples will be taken before any study 16% subcutaneous immunoglobulin product is administered:

**Parvovirus**	Parvovirus B19 (PCR)
**Virology**	Baseline samples of HBsAg, HIV (PCR) for adults and HCV (PCR) will be stored at -70°C and tested at the end of the study if the patient is found to be positive
**Archive**	Sample to be stored at -70°C for 15 years

One week after the first study SCIG infusion has been given, the following sample for parvovirus will be taken:

**Parvovirus**	Parvovirus B19 (PCR)

The following laboratory measurement will be taken at 4 weekly intervals throughout Phase 1:

**Liver Function**	ALT (and/or AST)
**Specific Antibodies**	Pneumococcus, HIB

The following laboratory measurements will only be assessed if clinically indicated throughout Phase 1:

**Haematology**	Haemoglobin, Haematocrit, RBC, WBC, Neutrophils, Lymphocytes, Monocytes, Eosinophils, Basophils, Platelet Count, Reticulocytes
**Biochemistry**	Sodium, Potassium, Creatinine, LDH, ALT (and/or AST), GGT, Total Bilirubin, Alkaline Phosphatase, Haptoglobin

At each timepoint, a repeat sample will be taken in case of laboratory error or loss of sample. In the case of clinically indicated samples, only when a sample is required to be taken will a repeat test sample also be taken at the same time:

**Repeat Test - Stored**	Separate serum aliquot to be stored at -20°C in case of repeat testing

During the study, any abnormal laboratory values that are considered to be clinically significant by the investigator must be repeated, using the stored sample, as soon as possible to rule out laboratory error.

Values outside the normal range for the laboratory, even those that are not considered to be clinically significant by the investigator must be commented on. All comments. will be recorded in the CRF using the following abbreviations:

NP: Normal for patient

NCS: Not clinically significant

CS: Clinically significant

All adverse events will be recorded in the CRF.

Patients considered suitable for home therapy will follow the schedule laid out in section 9.

8.2.3 Laboratory Sampling in Children

Laboratory sampling procedures defined by the protocol must be adhered to in the case of all adult patients over the age of 18 years. Where children are involved, all baseline samples should be obtained wherever possible, in particular, immunology and virology (except HIV testing will not be performed in patients under 18 years). Thereafter, as many subsequent blood samples as the individual is prepared to undergo or if sampling is clinically indicated should be collected in order to obtain adequate safety and efficacy data. End of study virology samples (except HIV) must also be collected to exclude viral transmission.

8.3 End of Phase 1 Procedures

The following assessments will be performed on all patients at the end of Phase 1 one week following the final study SCIG:

- Physical examination including weight, sitting blood pressure, pulse, temperature and respiration
- Concomitant medication
- Adverse events
- Patient Satisfaction Questionnaire
- Blood samples as follows:

**Haematology**	Haemoglobin, Haematocrit, RBC, WBC, Neutrophils, Lymphocytes, Monocytes, Eosinophils, Basophils, Platelet Count, Reticulocytes
**Biochemistry**	Sodium, Potassium, Creatinine, LDH, ALT (and/or AST), GGT, Total Bilirubin, Alkaline Phosphatase, Haptoglobin
**Immunology**	IgG, IgA, IgM
**Virology** (if not entering Phase 2)	HBsAg, HIV (PCR), HCV (PCR)
**Archive - Stored**	(if not entering Phase 2) Plasma sample to be stored at -70°C for 15 years
**Repeat Test - Stored**	Separate serum aliquot to be stored at -

	20°C in case of repeat testing

8.4 Blood Sample Collection, Handling and Labelling

Blood samples for haematology and biochemistry will be tested locally. The labelling, handling and testing of samples will be performed according to local procedures. Each blood sample must have the following minimum information:

- Study Number (SCIG01)
- Subject Number
- Date and time of collection

Virology and Parvovirus PCR will be performed by Central Public Health Laboratories, Colindale, London. Samples will be sent by courier to arrive by 10:00am on the day following collection. For Parvovirus samples, the serum will be separated from clot within 2 hours of clot formation.

A 5ml serum aliquot will be stored at -70°C as a baseline sample immediately prior to the first SCIG infusion at infusion 4 and again at the end of Phase 1. These must be clearly identified as above and kept at the centre for a minimum of 15 years from the time that the study is completed.

A separate 5ml serum aliquot will be stored at the centre at -20°C each time a study sample is obtained, in case of need of repeat sampling. These samples will be destroyed at the end of the study after consultation with BPL.

9.0 HOME THERAPY PROCEDURES

9.1 Criteria for Home Therapy

Self-infusion of SCIG will only be offered to suitable patients. The following criteria must be satisfied before a patient is to be considered eligible for home therapy:-

1. In the opinion of the investigator, the patient/parent/guardian must be motivated to perform the infusion.
2. After a period of training by the study staff, the patient/parent/guardian's ability to manage the infusion techniques must be satisfactory.
3. The patient's G.P. must agree to the patient performing home therapy.
4. A relative/friend who has also been trained in the techniques must be available to assist with each infusion performed at home.
5. The patient/parent/guardian must be contactable by telephone.

9.2 Home Therapy Training

A period of training will take place over 8-12 weekly infusions. The patient/parent/guardian will be trained to draw up the 16% immunoglobulin into a syringe, prime the tubing and insert the needle correctly, maintaining an aseptic technique throughout the procedure. The patient/parent/guardian will be instructed on how to use a syringe driver and also how to recognise adverse reactions and the appropriate action to take. Once the patient/parent/guardian is confident and proficient in all aspects of home therapy and when the investigator is satisfied that the patient/parent/guardian has reached the required standard to safely administer SCIG, they will be provided with a Home Therapy Manual (Appendix 10) and asked to sign a Home Therapy Consent Form (Appendix 11).

The criteria in section 9.1 must be satisfied before patients can receive SCIG at home. In addition, the patient's G.P. having already agreed in principle, must also be informed when the patient commences home therapy. This must be documented in the patient's notes.

9.3 Clinical Monitoring during Home Therapy

Prior to each infusion, the patient/parent/guardian will record the temperature and detail the result on the Home therapy Infusion Diary Card provided. If the patient's temperature is above the level pre-specified by the investigator and/or the patient is feverish, the SCIG should be delayed until the patient/parent/guardian has sought advice from the investigator using the contact number provided.

The following details will be routinely recorded on the Home Therapy Infusion Diary Card (Appendix 12):

- Infusion date
- Temperature (°C)
- Site(s) used (A= abdomen, T= thigh)
- Dose per site (mg)
- Total dose (mg)
- Rate (mm/hr)
- Batch number
- Comments/problems

9.4 Blood Sampling during Home Therapy

If practical, the patient should return to the clinic on a weekly basis for the same blood samples as patients receiving their infusions in hospital. However, since home therapy is intended to provide the patient with a more flexible regimen, blood samples taken every 4 weeks should be scheduled as a minimum. If it is clinically indicated or if local procedures require however, the patient will be asked to return for

more frequent blood sampling. This will be discussed and agreed with the patient on an individual basis.

Therefore, for the purposes of the study, the following minimum blood samples should be obtained from adult patients on home therapy:

Timepoints	Blood Tests
Pre-study	All tests detailed in section 8.1
Infusions 1-3<br>IVIG (if applicable)	All tests detailed in section 8.2.2
Infusion 4<br>SCIG	All tests detailed in section 8.2.2
Infusions 8, 12, 16, 20, 24, 28<br>(i.e. every 4 weeks)	All tests detailed in section 8.2.2
End of Study	All tests detailed in section 8.3

If the patient attends the clinic for an unscheduled visit, additional blood samples for immunology, haematology and biochemistry should also be taken as clinically indicated as well as a repeat test sample.

10.0 STUDY SCHEDULE - PHASE 2

At the end of Phase 1, patients will be invited to enter the safety follow-on study (Phase 2) and continue receiving subcutaneous 16% immunoglobulin either in hospital or at home until such time that they wish to discontinue treatment or the product is available on the market. Patients will be asked to attend study visits at the clinic at 3 monthly intervals throughout Phase 2 when they will be asked whether they have experienced any adverse events since the last visit. If clinically indicated, blood sample for haematology, biochemistry and/or immunology will also be taken. If the patient decides to withdraw from Phase 2 before the product is marketed, vital signs will be recorded and a sample for virology and an archive blood sample will be taken to exclude viral transmission during Phase 2.

11.0 STUDY SCHEDULE - PHARMACOKINETICS

It is expected that only a handful of patients (maximum 12) will take part in the pharmacokinetic assessment and that these will comprise of adult patients only, living near to the centres involved. Patients who agree to take part will be asked to return to the clinic every weekday for 1 week after the first study SCIG has been infused. This will be repeated for a further week, 3 months later. Patients will be given a Patient Information Leaflet for extra Blood Tests (Pharmacokinetics)(Appendix 7) and will be asked to sign the Pharmacokinetic Consent Form (Appendix 8)

11.1 Pharmacokinetic Procedures

Vital signs (body temperature, respiration, pulse and sitting blood pressure) and adverse events will be recorded at each visit. The following laboratory samples will be collected on each day that the patient attends for the pharmacokinetic part of the study:

**Immunology**	IgG, IgA, IgM
**Repeat Test - Stored**	Separate serum aliquot to be stored at -20°C in case of repeat testing

If clinically indicated, blood samples for haematology and biochemistry will also be taken.

12.0 EFFICACY MEASURES

12.1 Primary Efficacy Measurement

- The proportion of trough levels at each time point where the IgG $\geq$ 4g/L

12.2 Secondary Efficacy Measurements

- The change in SCIG dose required in order to maintain trough levels at a minimum of 6g/L. The number of occasions when the

dose adjusted, the size of the dose change and the overall range of dose changes will be calculated.

- The proportion of trough levels at each time point where the IgG $\geq$ 6g/L
- The time taken for each patient to reach a steady state IgG trough level. This will defined as when three consecutive occasions occur when the IgG trough levels are within 1g/L of each other.
- The mean change in IgG trough level as compared to the baseline level at each time point.
- The overall number of infections occurring and days off work/school

13.0 SAFETY MEASUREMENTS

- Number, type, severity and duration of adverse events occurring throughout the study.
- Laboratory monitoring of haematology, biochemistry and immunological and viral markers
- Monitoring of vital signs

Patient/parent/guardian's satisfaction of the subcutaneous route will be assessed by way of a short questionnaire at the beginning, middle and end of treatment.

14.0 WITHDRAWAL OF SUBJECTS

Patients will be informed that they are free to withdraw from the study at any time should they so wish without prejudicing their subsequent medical care. In the event of a withdrawal, the investigator will aim to perform the End of Phase 1 Procedures (section 8.3) or Study Schedule Phase 2 (section 10.0) as appropriate.

The clinical investigator may remove a patient if, in his/her opinion, it is in the best interest of the patient. A patient may be withdrawn from the study for any of the following reasons:

- Withdrawal of consent - any patient may withdraw from the study at any time
- Deviation from the protocol
- Incidental illness
- An adverse experience

If there is a medical reason for withdrawal, the patient will remain under the supervision of the investigator until in satisfactory health. After receiving the patient's consent, his/her general practitioner will be informed. Every effort will be made to contact patients who fail to attend an appointment to ensure that they are in good health. In any eventuality, the investigator must inform the Medical Department at BPL and record the withdrawal on the CRF.

15.0 ADVERSE EVENTS

An adverse event is defined as any untoward sign, symptom, illness or clinically significant abnormal laboratory value which appears or worsens during the course of the trial and is **temporally** associated with the administration of the test drug. **All adverse events whether or not considered by the Investigator to be related to the study drug(s) must be described and recorded on the appropriate ADVERSE EVENT FORMS in the CRF**. Where possible, a diagnosis should be made.

15.1 Definition of Serious Adverse Events

A **Serious Adverse Event** is any untoward medical occurrence which at any dose:

- results in death
- is life threatening (patient at risk of death at time of event; not hypothetically life-threatening)
- results in persistent or significant disability
- requires or prolongs hospitalisation
- is a congenital anomaly or birth defect

15.2 Detecting Adverse Events

Adverse events should be elicited by careful questioning of the subject at each visit. The Investigator and others responsible for care of the subjects should institute any supplementary investigations of significant adverse events based on the clinical judgment of the likely causative factor. This may include seeking a further opinion from a specialist in the field of the adverse event. The company may suggest special tests based on expert advice. If a serum sample is collected for assay of the test medication or for additional laboratory tests, the Investigator must ensure that the plasma sample is properly labelled and stored.

15.3 Completing Adverse Event Forms

Adverse Event Forms must be completed in a timely manner and contain the following information:

- subject number, initials and date of birth
- description of event (where possible a diagnosis should be made rather than just listing symptoms)
- **relevant** medical history
- study drug, dose and batch number and expiry date
- **all** concurrent medication (comment if suspected cause of event)
- intensity and causality must be assigned following the instructions below:
- action taken (e.g. drug discontinuation) and treatment given
- outcome
- signature of the study physician and date

15.4 Assessment of adverse event intensity

Intensity of adverse events will be assessed by a **nurse or physician**. The following guidelines should be used to assess intensity:

Mild: Awareness of signs or symptoms that are easily tolerated.

Moderate: Discomfort enough to cause interference with usual activity.

Severe: Incapacitating with inability to do usual work.

15.5 Attribution to study drugs

Attribution of adverse effects to study drugs will be assessed by a **physician** according to the following criteria (based on Karch FE, Lasagna L. JAMA 1975; 234: 1236-1241) and recorded on the CRF.

Probable

A reaction that follows a reasonable temporal sequence from administration of the drug, and follows a known response pattern to the suspected drug.

The reaction cannot reasonably be explained by the know characteristics of the subject's clinical state or other modes of therapy administered to the subject.

Possible

Plausible temporal sequence

Follows a known response pattern to the suspected drug

The adverse event might have been produced by the subject's clinical state or other modes of therapy administered to the subject.

Unlikely

The current state of knowledge indicates that a relationship is unlikely

Unknown

It is not possible to assign adverse event to any of the above categories

Not related

In the opinion of the physician the event is unrelated to the study drug.

Study drugs are defined as those investigational compounds or their controls used in a study.

In this study, the study drug is: 16% immunoglobulin

15.6 Reporting Adverse Events

The Investigator is responsible for prompt and complete reporting of all adverse events. This facilitates:

- a greater understanding of drug toxicity
- appropriate modification of study protocols
- adherence to regulatory requirements, thus protecting study subjects and prescribing physicians

The Investigator is responsible for complying with his Local Research Ethics Committee's (LREC) policy on adverse event reporting. BPL will inform the MREC and MCA in line with regulatory guidelines on adverse events occurring during the trial. The investigator retains the right to inform the MCA if he/she so desires, but must inform BPL so that duplicate reports to the MCA can be highlighted.

WHEN A ADVERSE EVENT OCCURS WHICH FULFILLS THE DEFINITION OF SERIOUS (above) THE INVESTIGATOR MUST IMMEDIATELY:

1. **Telephone or fax available details to the designated CRA or Medical Affairs Manager at BPL**
2. **Complete and sign the adverse event form and send it to the appropriate contacts (see below)**
3. **Inform the LREC of the adverse event where appropriate (i.e. if believed to be study drug related and is unexpected, or if it is a requirement of the adverse event reporting policy of the LREC).**

Dr E W Gascoigne
Medical Affairs Manager
Bio Products Laboratory
Elstree
Herts WD6 3BX
Telephone: 020 8258 2622
Fax: 020 8258 2611

Out of hours, BPL Security will contact the Medical Affairs Manager

BPL will inform all Investigators of adverse reactions occurring during the study which would materially affect the safety of study subjects.

15.7 Adverse Event Follow-up

All adverse events will be followed up:

- to resolution

or

- until an underlying condition has been diagnosed

or

- until the patient's condition has stabilised

or

for a period of 28 days following administration of the study drug

16.0 DATA ANALYSIS

16.1 Statistical Analysis

This is a non-comparative study and therefore descriptive statistical techniques will be employed in the analysis of some of the data.
The primary efficacy measurement is defined as the proportion of the total number of trough levels at each time point where the IgG value is greater than or equal to 4g/L. Thus the number of occurrences that this

occurs during the study will be calculated. This value of 4g/L has been chosen because from the literature it seems to be an appropriate level where infections start to decrease because of treatment efficacy. However, data from a previous clinical trial conducted by BPL using primary antibody deficiency patients previously treated with IVIG showed that the mean IgG level at baseline was 8g/L (95% CI = 6.1 - 9.9)[6]. During this study the aim was to therefore maintain the patient's IgG trough level within this 95% CI (i.e.6.1 - 9.9) and the number of patients that fall outside this target will be calculated. In addition, if the data is found to be normally distributed, an analysis of variance will be performed to determine the influence of time on trough IgG levels.

The sample size of 40 evaluable patients has been chosen so that an adequate number of patients receiving both IVIG and SCIG prior to study SCIG can be assessed. This number will also allow for drop-outs and withdrawals. While it would be preferable to have equal numbers in both groups, the study is not reliant upon this. However, a minimum of 10 patients receiving IVIG at baseline and 10 receiving SCIG will be aimed for when recruiting patients. These subgroups of patients will be analysed seperately.

The number, type, severity and duration of adverse events, together with monitoring of haematology and biochemical screens and viral markers will also be analysed and described.

16.2 Definition of Evaluability

Patients will be considered evaluable and included in the analysis if they satisfy the entry criteria and receive one or more study SCIG infusions.

16.3 Dropouts

All patients who drop-out or who are withdrawn from the study prior to the first study SCIG infusion will not be considered evaluable for efficacy. The patient's data will however be included in the safety analysis.

All drop-outs and withdrawals must be fully documented and their case record forms submitted to BPL.

16.4 Interim Analysis

If considered necessary by BPL, an interim analysis, using the above statistical tools, will be performed either after at least 10 patients have completed Phase 1 of the study or 20 patients have completed 3 months of SCIG treatment.

17.0 DOCUMENTATION

17.1 Required Pre-Study Documentation

Before the start of the study, BPL will require the following documentation:

- A signed copy of the protocol
- An information leaflet and/or consent form if different from that of the sponsor
- A signed financial agreement
- A signed curriculum vitae for each Investigator and principal co-Investigator
- A copy of laboratory normal ranges for tests required in the protocol
- Evidence of laboratory accreditation and/or performance in relevant National External Quality Assessment Schemes (NEQAS)
- LREC submission, constitution, written approval and composition.

- Signed confidentiality agreement

17.2 Recording data in Case Record Forms

All study data will be recorded on CRFs provided by BPL. These must be completed by the Investigator or a duly authorised assistant. CRFs must be completed in a legible manner using a black ball point pen. Entries made in pencil, colour ink or with a felt tip pen are not acceptable. Entries should be made directly and promptly.

Where data is absent, appropriate abbreviations should be entered instead of leaving blank fields, e.g:

- ND = Not done — should be entered where the required test was not performed. The reason should be stated, e.g. instrument failure and then signed and dated.
- NA = Not Applicable — should be entered where the CRF requires information which is not appropriate

Where problems arise with blood samples the following abbreviations may be entered:

- H = Haemolysed
- SH = Slightly haemolysed
- GH = Grossly haemolysed
- SS = Short sample. The sample is less than that outlined in the protocol
- BC = Broken container

17.3 Error Correction

- Errors should be corrected by drawing a single black line through the entry without obscuring the original data.
- Corrections should be recorded beside it and should be signed, dated and an explanation given (if necessary).
- Tippex should never be used

17.4 Signing Off and Return of Case Record Forms

The Investigator at each site must sign the completed CRFs to confirm the validity of the data. The Investigator is obliged to return the completed CRFs to BPL at the end of the study. A copy will be retained in the Investigator file.

17.5 Maintenance and Archiving of Study Records

The Investigator must maintain adequate records for the duration of the study.

Before the study begins the following documentation must be present in the Investigator file:

- CRF
- Investigator Brochure
- Signed final protocol and amendments
- Information sheet and consent form for study patients
- CVs for Investigators and co-Investigators
- Financial agreements between
  - Sponsor and Investigator
  - Investigator and other departments for conducting the study, e.g. laboratory/nursing staff/organisation (if applicable)
- Letter of indemnity (if requested)
- Confidentiality statements
- LREC composition, constitution, submission and letter of approval
- Relevant MREC documentation including submission correspondence and letter of approval
- Normal laboratory values
- Laboratory certification/ accreditation and/or NEQAS performance or validation of analytical methods used in the study.
- Details of transport of test

medication and study supplies

During the study the following documents must be added to the file:

- Updates on Investigators brochure (if applicable)
- Amendments (as applicable)
  - to protocols
  - to CRFs
  - information sheets
  - consent forms
- Dated approvals or opinions for any amendments
- Reports to LREC on study progress
- CVs for new Investigators and co-Investigators
- Updates on normal laboratory values
- Updates on technical procedures/tests
- Drug accountability records
- Initiation report
- Signed and dated completed CRFs
- Documentation of CRF corrections
- All correspondence concerning adverse event reports between
  - Sponsor and Investigator
  - Investigator and LREC
  - Investigator and MCA (if reported directly)
- Interim or annual reports
- Patient screening log
- Patient ID log
- Sample log
- Site signature log
- Final study report (if applicable)
- Correspondence including
  - letters
  - telephone calls
  - meeting notes and minutes
- Signed informed consent forms
- Relevant MREC correspondence

These records must be available for inspection upon reasonable request by the sponsor, members of the regulatory authorities or other authorised individuals.

The Investigator must make proper provision for archiving study documentation at the centre. Patient ID codes must be retained for a period of 15 years after the issue of the final study report. Patient consent forms and other study related documentation must be retained for the maximum period of time permitted by the hospital.

18. STUDY CONDUCT

18.1 Adherence to the Protocol

Deviations from the protocol must not be made without the prior written approval of BPL and the MREC, except where there are logistical or administrative changes, or where they are implemented to eliminate an immediate threat or hazard to the health or safety of the patient. Where a deviation has been made to eliminate an immediate hazard, the Investigator must submit the implemented deviation and the reasons for it to the MREC and must notify relevant members of the Medical Department of BPL. All deviations must be adequately documented in the CRF.

18.2 Protocol amendments

When an amendment is necessary, the principal researcher must comply with the MREC policy on notification of amendments. If the amendment substantially alters the study design or increases the risk to the patients, the principal researcher must do the following:

1. Submit the amendment to the MREC for review and favourable opinion.
2. Notify BPL in writing of the MREC's opinion.
3. Where appropriate revise the consent form and patient information leaflet and have it approved by the MREC.
4. Where the amendment affects the risk/benefit ratio of continued participation for patients already enrolled in the study, informed consent should be obtained using the new information leaflet/consent form.

5. Use the updated version of the information leaflet/consent form for new patients.

Similarly, each Investigator is obliged to inform their LREC of any protocol amendments.

18.3 Early Cessation of the Study

BPL reserves the right to stop the study if:

- Evidence is gained that patients are being exposed to an unacceptable risk
- For any reason, it is not possible to continue to supply the study material
- An advancement in knowledge makes the treatment redundant
- Recruitment is too slow to allow accrual of an adequate number of patients in a reasonable length of time

18.4 Monitoring Visits and Audit

The BPL Clinical Research Associate will monitor the study by telephone, correspondence, and regular visits to the investigative sites. He/she will ensure that:

- the facilities remain adequate
- the Investigator adheres to the protocol and ethical responsibilities
- source documents are legible and agree with entries in the CRF
- adverse events are adequately documented and reported
- study medication is properly stored and drug inventories are being maintained
- samples are identified, handled and stored appropriately

18.6 Obligations of the Investigator

The Investigator must conduct the study as outlined in the protocol and in accordance with the ICH guidelines for GCP. Please refer to Appendix I on Investigators Responsibilities at the back of the protocol.

18.7 Confidentiality

Information received pursuant to this study will be regarded as confidential at all times. The Investigator and co-Investigators will be required to sign a confidentiality statement.

19.0 ETHICAL CONSIDERATIONS

The study will be performed in accordance with the Guidelines of the Declaration of Helsinki on biomedical research involving human patients (South Africa revision 1996) and in accordance with ICH GCP guidelines. Before the study can begin the principal researcher must have received documentary evidence of approval of the study protocol from the MREC. Subsequently, to gain LREC approval, each Investigator must supply the following documentation for review:

- MREC application form
- MREC letter of approval
- Signed MREC response form
- Protocol
- Patient information leaflet and consent form
- Any advertisement for subject recruitment
- The Investigator Brochure
- Investigator's Curriculum Vitae

Once approval has been granted the Investigator is responsible for ensuring that he/she complies with the terms of the approval namely with respect to adverse event reporting, notification of amendments, interim and final reports on the progress of the study.

19.1 CONSENT PROCEDURE FOR ADULTS

Before the decision to participate is made by adult patients. The investigator or a duly authorised deputy will provide both an oral and written full explanation of the study. After the patient has been supplied with information and has had sufficient time to review it and ask questions, he/she will be included in the study only if the investigator is sure that the patient understands the implications of taking part. His/her agreement to participate will be documented by the patient signing and dating a consent form. The consent form will be stored at the investigator's site and a signed copy given to the patient.

19.2 CONSENT PROCEDURE FOR CHILDREN

Where children are invited to participate in the study, if in the opinion of the investigator he/she is of sufficient maturity to understand the nature of the study the investigator or a duly authorised deputy, will provide both an oral and written full explanation of the study. After the patient has been supplied with information and has had sufficient time to review it and ask questions, he/she will be included in the study only if the investigator is sure that the child understands the implications of taking part. His/her agreement to participate will be documented by the patient signing and dating a Consent Form, this will be stored at the investigator's site and a signed copy given to the patient.

Where patients are not of sufficient maturity to understand the full implication of taking part in the study the investigator or a duly authorised deputy, will provide both an oral and written full explanation of the study to the parent/guardian. After the parent /guardian has been supplied with information and has had sufficient time to review it and ask questions, he/she will be able to give consent for their child to be included in the study only if the investigator is sure that the parent/guardian understands the implications of their child taking part. His/her agreement to allow their child to participate

will be documented by the parent/guardian signing and dating a consent form in addition to the investigator. The Consent Form will be stored at the investigator's site and a signed copy given to the parent/guardian.

In the event that the child does not wish to participate in the study this will preclude them entering even if parent/guardian consent is granted. It is expected that children over the age of 8 years will be of sufficient maturity to provide consent but children younger than this should also be given the opportunity if appropriate.

The subject's GP must be informed of the patient's participation in the study.

19.3 Compensation/Indemnity

Compensation will be paid by BPL according to the Guidelines drawn up by the Association of the British Pharmaceutical Industry if a patient is injured as a result of being in this study.

Compensation will not be provided for injury or medical conditions that are unrelated to this study.

BPL will indemnify the Institute and the Investigator with respect to any claim for personal injury or death brought against it resulting from the administration to volunteer subject of source materials supplied by BPL, provided the protocol and Investigator agreement have been adhered to, and the event has not been occasioned by malpractice or negligence.

20.0 INVESTIGATOR'S STATEMENT

A multi-centre, open study to assess the safety and efficacy of a 16% immunoglobulin product given via the subcutaneous route in primary antibody deficient patients

I have carefully read this protocol and the Clinical Investigator's Brochure and I confirm that they contain all the information necessary to perform the study. I agree to carry out the study as outlined in this protocol.

Signature: ______________________________

Date: ______________

Name : ______________________________

Centre : ______________________________

APPENDIX 15: STUDY FLOW CHART

PHASE 1
SAFETY & EFFICACY

PRESTUDY VISIT

Up to 28 days prior to infusion 1

UP TO 28 DAYS

INFUSIONS 1-3

1-3 routine doses of current routine IVIG/SCIG. Final dose given 1 week prior to first study SCIG

1 WEEK

INFUSIONS 4-30

- First dose of study SCIG given 1 week after last routine IVIG/SCIG.
- Initial dose: 100mg/kg/week.
- Repeated, weekly infusions of study SCIG at 100mg/kg/week or tailored to the patient's trough IgG levels - minimum target 6g/l.
- Option for home therapy after training

6 MONTHS

Selected patients attend clinic daily for 1 week on 2 occasions for **PHARMACOKINETIC EVALUATION** (see later)

OR

END OF STUDY
Complete study and return to previous therapy

ENTER PHASE 2
Remain on study SCIG

21.0 REFERENCES

1. Williams PE, Todd AAM, Yap PL. IVIG Therapy in Primary and Secondary Immunodeficiency. Clinical Applications of Intravenous Immunoglobulin Therapy. Churchill Livingstone 1992: 63-92
2. Berger M, Cupps TR and Fauci AS. Immunoglobulin Replacement Therapy by Slow Subcutaneous Infusion. Ann Intern Med 1980; 93: 55-56
3. Gardulf A, Hammarstrom L, Smith CIE. Home Treatment of Hypogammaglobulinaemia by Rapid Infusion. The Lancet 1992; 338: 162-166
4. Waniewski J, Gardulf A, Hammarstrom L. Bioavailability of γ - Globulin After Subcutaneous Infusions in Patients with Common Variable Immunodeficiency. Journal of Clinical Immunology 1994; vol 14, No 2: 90-97
5. Brennan VM. Subcutaneous Immunoglobulin Therapy: A manual for administration of immunoglobulin by rapid subcutaneous infusion
6. Data on file, VIGPAD Clinical Report, Bio Products Laboratory, Dagger Lane, Elstree, Hertfordshire

APPENDIX 1: ADMINISTRATION OF SUBCUTANEOUS INFUSION

Materials Required

- Sterile paper towel
- Medi-swabs
- Needles for drawing up (19G)
- Syringes (10ml)
- Infusion set
- Butterfly needle (28G)
- Tape
- Cotton wool
- Sharps bin
- Syringe drivers (Graseby MS16A)
- Subcutaneous immunoglobulin
- Adrenaline

Assessment of the Patient

- Vital signs should be taken prior to each infusion and an infusion should not be administered if the patient has a fever or is unwell

Method

1. Check syringe drivers and set rate as prescribed
2. Wash hands
3. Clean surface and lay paper towel
4. Check immunoglobulin for correct dose and expiry date
5. Remove vial tops and draw up immunoglobulin with syringe and needle
6. Attach infusion needle to syringe and prime tubing
7. Prepare tape and wipe site (abdomen/thigh) to be used with medi-swab
8. Insert the needle into the abdomen/thigh as instructed and tape firmly in place
9. Check for any blood return by withdrawing the syringe plunger and by removing the syringe from the tubing
10. If blood returns re-site the needle and repeat 9
11. Once position is satisfactory with no blood return, place the syringe on the driver and secure in place
12. Switch on the driver and administer infusion
13. When the alarm sounds remove the needle and dispose of in the sharps bin
14. Repeat process if further dose is needed
15. At the end of the infusion dispose of all used materials, wash hands and record details

APPENDIX 2 ADULT PATIENT INFORMATION LEAFLET

A MULTI-CENTRE, OPEN STUDY TO ASSESS THE SAFETY AND EFFICACY OF A 16% IMMUNOGLOBULIN PRODUCT GIVEN VIA THE SUBCUTANEOUS ROUTE IN PRIMARY ANTIBODY DEFICIENT PATIENTS

This purpose of this leaflet is to tell you about a research study in which you are invited to take part. If there is anything which you do not understand, or if you require more information, please ask your doctor or nurse. Please read these notes carefully, before starting the study, and keep them safe so that you may look at them again. The study has been reviewed and approved by the hospital ethics committee.

Why is the study being done?

You have been asked to take part in a study to test a specially treated blood product. It is called immunoglobulin and is used to treat your condition (primary antibody deficiency) by replacing the low levels of antibodies in the blood. At the moment most patients in the UK receive their immunoglobulin treatment into a vein every few weeks over a number of hours. However, in other countries such as Sweden, they give it under the skin (subcutaneous). This can be useful for home therapy or for patients who cannot have their infusions into a vein for medical reasons. The immunoglobulin product that we will be using in this study is virtually identical to the Swedish one but we want to check that giving it under the skin is safe and keeps your condition controlled.

Are there other ways of treating my condition?

Primary antibody deficiency causes low levels of the antibodies in the blood which are there to help fight infection. Therefore, these antibodies need to be replaced. This is usually achieved by giving immunoglobulin treatment into a vein. A few patients already receive their immunoglobulin under the skin by special arrangement with the people who make it but at the moment there are no products available on the market that have a licence. There are a quite a few products available that are already licensed to be given into a vein however and you may well be receiving one of these already.

Why have I been chosen and who is organising the study?

The study will involve at least 40 patients from around the country, each of whom have the same condition as you. The study as a whole is expected to last about 2 years.

The organisers of the study are called Bio Products Laboratory (BPL), which is a part of the blood donor organisation, the National Blood Authority. They are the manufacturers of the immunoglobulin that will be used in the study.

What are the possible benefits of taking part?

Immunoglobulin therapy is a recognised treatment for primary antibody deficiency so the number of antibodies in your blood would be kept at a suitable level to control your condition. However, having the treatment under the skin instead of into a vein may give a better level of antibodies which also may not go up and down as much as sometimes happens with immunoglobulin treatment into a vein. Also, the results that we get from this study should give doctors more treatment choices in the future.

What are the risks of taking part or what if something goes wrong?

The blood tests may hurt slightly and cause slight bruising. You may need to have a few extra blood tests done for the purpose of the study than you would normally have, but this should not cause you any harm. You may get some swelling and redness where the immunoglobulin is injected but this should go away within a day. Serious problems do not happen very often but we need to let you know what they could be just in case - hard skin under the injection site, pains in the chest, arms or legs, feeling out of breath, shaking hands, feeling dizzy, puffy face and a sore mouth. However, so far, in BPL's experience, only 1 serious problem in 61,000 intramuscular doses has been reported following treatment.

Although all blood donations used to make the immunoglobulin are checked for hepatitis B, hepatitis C and HIV viruses, there is still a small risk that one of these could be passed on. The product is specially treated to destroy them however, so far there has been no reports of patients getting them from the product. At the end of the study, your blood would need to be tested to check that none of the viruses have been passed on to you during the course of the study. If you do not wish these tests to be carried out then you should not take part in the study.

If you are harmed because of the study, compensation would be provided without you having to demonstrate fault. Bio Products Laboratory operates to the guidelines drawn up by the Association of the British Pharmaceutical Industry for subjects in clinical trials and will therefore treat any claim according to these guidelines. A copy is available from your doctor if you ask him/her.

What would I have to do if I decided to take part in the study?

Before the start of the study, you would be asked about your medical history and be given a complete medical examination. The doctor or nurse would take your blood pressure, pulse rate, temperature and respiration rate. You would also have a blood test.

If you are suitable for the study, you would carry on with your normal immunoglobulin treatment for up to 3 more times. This is so we can collect some baseline information. When your routine treatments have finished, you would start coming to the clinic one week later to receive the immunoglobulin subcutaneously. The first dose would be given at 100mg/kg bodyweight. After that, you would come back every week for your treatment and your doctor would take a blood test at each visit to decide what dose you should have each time. Some of the blood would also be saved while you are on the study in case it needs to be tested again. This would be discarded at the end of the study except for blood taken at the beginning and end of the study which would be kept for at least 15 years in a freezer in case we ever need to re-test it to re-check the results of this study. It would not be used for any other purpose than this such as another study.

Before the treatment, every hour during and again afterwards the nurse would take your temperature, blood pressure, pulse and respiration rates. You would also be watched for any side effects and the injection site would be checked. The treatment would be given under the skin in two places at the same time either in your thigh or abdomen whichever is best for you.

You would carry on with these infusions every week for about six months after which you would need to decide whether you want to carry on with the study or go back to your normal treatment. Your doctor will be able to advise you. If you carry on with the study treatment you would need to complete a weekly diary card and see the doctor every 3 months for a check-up. You would be able to carry on until the product has a licence which would then be the end of the study. You would not need any more blood tests during this time unless the doctor thought you needed them except for a test right at the end of the study to check that none of the viruses mentioned before had been passed on to you.

Would there be any restrictions?

You should not take any alcohol for at least 12 hours before the first treatment.

You should carry on with any medicines your doctor has told you to take but try not to take any other medicines the day before the first treatment and if possible during the study. If you need to take anything else, including ones that you can buy from the chemist yourself, please remember to tell the doctor.

What if I don't want to take part?

You don't have to take part in the study if you don't want to. If you decide to take part then you can change your mind at any point without giving a reason. This would not affect your medical care now or in the future. If, in the future, you decide you do not want to stay in the study, you must tell your doctor or the study staff and they will tell you what to do.

The doctor may decide to take you out of the study if it is in your best interest, with or without your consent. If you come out of the study, you would continue to receive other appropriate treatments for your condition. You would also be told by your doctor of any new information which may help you decide whether or not to carry on with the study.

Would my records be kept secret?

Staff from BPL and possibly the Regulatory Authorities, such as the Medicines Control Agency, would need to look at your hospital notes. However, all information about you in this study would be kept secret and if the results of the study are published, your name would not appear and no one would know your identity. If you decide to take part your GP would be told.

Would I get paid for taking part?

You would not be paid to take part in this study. However, reasonable travel expenses to and from the clinic as well as other study related expenses you may incur, will be paid.

Who should I ask if I have any more questions?

Any questions you have about this study can be answered by Dr/Nurse ________________________ who may be reached by telephone on ____________________

If you have any questions about your rights as a research subject or about an injury related to the study you should contact Dr/Nurse __________________ as above.

In an emergency, please contact:

Name: ____________________________

Tel: ____________________________

APPENDIX 3: PARENT/GUARDIAN INFORMATION SHEET

A MULTI-CENTRE, OPEN STUDY TO ASSESS THE SAFETY AND EFFICACY OF A 16% IMMUNOGLOBULIN PRODUCT GIVEN VIA THE SUBCUTANEOUS ROUTE IN PRIMARY ANTIBODY DEFICIENT PATIENTS

This purpose of this leaflet is to tell you about a research study in which your child is invited to take part. If you agree to allow your child to take part in the study yuo will be asked to sign a consent form and if your child is old enough to understnd the study then he/she will also be asked to sign the form after the study has been explained to heim/her and if he/she wants to take part. If there is anything which you do not understand or if you require more information, please ask the doctor or nurse. Please read these notes carefully, before your child starts the study, and keep them safe so that you may look at them again. The study has been reviewed and approved by the hospital ethics committee.

Why is the study being done?
Your child has been asked to take part in a study to test a specially treated blood product. It is called immunoglobulin and is used to treat his/her condition (primary antibody deficiency) by replacing the low levels of antibodies in the blood. At the moment most patients in the UK receive their immunoglobulin treatment into a vein every few weeks over a number of hours. However, in other countries such as Sweden, they give it under the skin (subcutaneous). This can be useful for home treatment or for patients who cannot have their infusions into a vein for medical reasons. The immunoglobulin product that we will be using in this study is virtually identical to the Swedish one but we want to check that giving it under the skin is safe and keeps your child's condition controlled.

Are there other ways of treating my condition?
Primary antibody deficiency causes low levels of the antibodies in the blood which are there to help fight infection. Therefore, these antibodies need to be replaced. This is usually achieved by giving immunoglobulin treatment into a vein. A few patients already receive their immunoglobulin under the skin by special arrangement with the people who make it but at the moment there are no products available on the market that have a license. There are quite a few products available that are already licensed to be given into a vein however and your child may well be receiving one of these already.

Why has my child be chosen and who is organising the study?
These study will involve at least 40 patients from around the country each of whom have the same condition as your child. The study as a whole is expected to last about 2 years.
The organisers of the study are called Bio Products Laboratory's (BPL), which is a part of the blood donor organisation, the National Blood Authority. They are the manufacturers of the immunoglobulin that will be used in the study.

What are the possible benefits of him/her taking part?
Immunoglobulin therapy is a recognised treatment for primary antibody deficiency so the number of antibodies in your child's blood would be kept at a suitable level to control his/her condition. However, having the treatment under the skin instead of into a vein may give a better level of antibodies which also may not go up and down as much as sometimes happens with immunoglobulin treatment into a vein. Also, the results that we get from this study should give doctors more treatment choices in the future.

What are the risks of taking part or what if something goes wrong?

The blood tests may hurt slightly and cause slight bruising. Your child may need to have a few extra blood tests done for the purpose of the study than he/she would normally have, but this should not cause him/her any harm. There may be some swelling and redness where the immunoglobulin is injected but this should go away within a day. Serious problems do not happen very often but we need to let you know what they could be just in case - hard skin under the injection site, pains in the chest, arms or legs, feeling out of breath, shaking hands, feeling dizzy, puffy face and a sore mouth. However, so far, in BPL's experience, only 1 serious problem in 61,000 intramuscular doses has been reported following treatment.

Although all blood donations used to make the immunoglobulin are checked for HIV, hepatitis B and hepatitis C viruses, there is still a small risk that one of these could be passed on. The product is specially treated to destroy them however, so far there has been no report sof patients getting them from the product. At the end of the study, your child's blood would need to be tested to check that neither hepatitis B nor hepatitis C have been passed on to him/her during the course of the study (HIV testing is not normally done on children). If you do not wish these tests to be carried out then you should not agree for him/her to take part in the study.

If your child is harmed because of the study compensation would be provided without you or your child having to demonstrate fault. Bio Products Laboratory, operates to the guidelines drawn up by the Association of the British Pharmaceutical Industry for subjects in clinical trials and will therefore treat any claim according to these guidelines. A claim can be made via the investigator, setting out details and nature of the claim which operates to the Clinical trial compensation guidelines as drawn up by ABPI. A copy of the guideline is available from the doctor if you ask him/her.

What would my child have to do if he/she took part in the study?

Before the start of the study, you and you child would be asked about his/her medical history and he/she would be given a complete medical examination. The doctor or nurse would take his/her blood pressure, pulse rate, temperature and respiration rate. He/she would also have a blood test.

If your child is suitable for the study, he/she would carry on with their normal immunoglobulin treatment for up to 3 more times. This is so we can collect some baseline information. When the routine treatments have finished, your child would start to receive the immunoglobulin under the skin, one week later. The first dose would be given at 100mg/kg bodyweight. After that, he/she would come back every week for the new treatment and the doctor would take some blood at some of the visits if necessary. Some of the blood would also be saved while your child is on the study in case it needs to be tested again. This would be discarded at the end of the study except for blood taken at the beginning and end of the study which would be kept for at least 15 years in a freezer in case we ever need to re-test it to re-check the results of this study. It would not be used for any other purpose than this such as another study. Further consent to perform test on these samples should it be necessary, would not be sought and the results of any further research tests performed on these samples would not be made available to parent/child.

Before the treatment, every hour during and again afterwards the nurse would take your child's temperature, blood pressure, pulse and respiration rates. He/she would also be watched for any side effects and the injection site would be checked. The treatment would be given under the skin in either the thigh or abdomen whichever is best for him/her.

Your child would carry on with these infusions every week for about six months after which you and your child would need to decide whether you want him/her to carry on with the study or go back to his/her normal treatment. The doctor will be able to give you both advice. If your child carries on with the study treatment, a weekly diary card would need to be completed and your child would see the doctor every 3 months for a check-up. Your child would be able to carry on until the product has a license which would then be the end of the study. He/she would not need any more blood tests during this time unless the doctor thought they were necessary except for a test right at

the end of the study to check that none of the viruses mentioned before had been passed on and no further consent will be obtained or required for pre and/or completed samples.

Would there be any restrictions?

Your child should carry on with any medicines prescribed by his/her doctor but try not to give him/her any other medicines the day before the first treatment and if possible during the study. If he/she does need to take anything else, including ones that you can buy from the chemist yourself, please remember to tell the doctor.

What if my child or I don't want him/her to take part?

Your child does not have to take part in the study. If you agree to your child taking part then you can change your mind at any point without giving a reason. This would not affect your child's medical care now or in the future. If, in the future, you decide you do not want your child to stay in the study, you must tell the doctor or the study staff and they will tell you what to do.

The doctor may decide to take your child out of the study if it is in his/her best interest, with or without your consent. If he/she comes out of the study, he/she would continue to receive other appropriate treatments for his/her condition. You would also be told by the doctor of any new information which may help you decide whether or not to carry on with the study.

Consent procedure for children

Children are legally dependent on their parents/guardians who take the legal responsibility for their welfare and safety and fully informed consent should be obtained from the legal guardian in accordance with national legislation. Children can consent if the Investigator is sure that the child understands the study requirements and implication of taking part. His/her agreement to take part will be documented by the patient signing and dating a consent form in addition to the parent/guardian. The Consent Form will be stored at the Investigator's site and a signed copy given to the patient.

Would my child's records be kept secret?

Staff from BPL and possibly the Regulatory Authorities, such as the Medicines Control Agency, would need to look at your child's hospital notes. However, all information about him/her in this study would be kept secret and if the results of the study are published, his/her name would not appear and no one would know his/her identity. If you and your child decide to take part his/her GP would be told.

Would my child get paid for taking part?

He/she would not be paid to take part in this study. However, reasonable travel expenses to and from the clinic as well as other study related expenses incurred, will be paid for.

Who should I ask if I have any more questions?

Any questions you have about this study can be answered by Dr/Nurse ____________________ who may be reached by telephone on ____________________

If you have any questions about your child's rights as a research subject or about an injury related

to the study you should contact Dr/Nurse ____________________ as above.

In an emergency, please contact:

Name: ____________________ **Tel:** ____________________

APPENDIX 4: CHILDREN'S INFORMATION LEAFLET

A MULTI-CENTRE, OPEN STUDY TO ASSESS THE SAFETY AND EFFICACY OF A 16% IMMUNOGLOBULIN PRODUCT GIVEN VIA THE SUBCUTANEOUS ROUTE IN PRIMARY ANTIBODY DEFICIENT PATIENTS

This leaflet tells you about a study which you can take part in if you want. If you do not understand what you have to do then please ask your doctor or nurse.

Why is the study being done?
In this study we want to test a medicine called an immunoglobulin. It comes from blood donors and is usually given to people, with the same illness as you, into their muscle or vein. At the moment most patients in Britain are given it into a vein every few weeks. But, in other countries such as Sweden they are given it under the skin if they cannot have it in a vein. We want to check that this medicine is safe to give under the skin and also that it helps your illness.

Why have I been picked?
There will be about 40 patients from different hospitals in Britain who will take part in the study. They will all have the same illness as you. The people who make the medicine are called Bio Products Laboratory (BPL). This is part of the same place that blood donors go to when they give blood.

What are the good and bad sides of taking part?
This treatment should make you feel better by helping you to fight coughs and colds. Also, the results will help doctors treat patients like you in the future. Taking blood may hurt a little bit and you may get a small bruise on your arm. You would need to have a blood test every month during the study. Also the doctor will save some of your blood each time in case the doctor needs to test it again. When you have your treatment, you may get a bit of swelling and redness where it goes in under the skin but this should go away by the next day. You shouldn't get any other problems but we have to tell you what they could be in case they happen. You could get a hard lump under the skin or a pain in your chest, arms or legs. You may get out of breath, feel dizzy or shaky, get a puffy face or a sore mouth. These are all very unlikely though.

All the blood that makes the medicine is tested for nasty bugs and is made in a special way that kills them. At the end of the study, we would like to test your blood to check that you haven't caught any of these bugs during the study. If you are hurt in any way because of the study it wouldn't be your fault and the people who make the medicine would pay for this if it happened.

What would I have to do?
Before it started your doctor would ask you about yourself and give you a check-up. You would be weighed, have your temperature taken and your heart beat and breathing checked. You would also need to have a blood test. You would then have your usual treatment for up to 3 more times. Then you would come to the hospital every week to get your medicine under the skin in your leg or tummy. Your doctor may want to take a blood test at some of these visits. While you are having the medicine you would have your temperature taken and your heart beat and breathing checked. The nurse would also watch you for any problems.

You would carry on like this for 6 months and then you would be able to go back to your normal treatment or carry on with this new one if you wanted. If you carried on you would need to fill in a weekly diary and see the doctor every few months. You wouldn't need to have any more blood tests unless your doctor thought you needed them.

You don't have to take part in the study if you don't want to. Also, you can change your mind at any time once you have started. Remember to tell your doctor or nurse if you are worried about anything and they will help you.

Who should I ask if I have any more questions?

Any questions you have about this study can be answered by Dr/Nurse _____________________ who may be reached by telephone on ___________________

If you have any questions about your child's rights as a research subject or about an injury related to the study you should contact Dr/Nurse __________________ as above.

In an emergency, please contact:

Name: __________________________ **Tel:** ___________________________

APPENDIX 5 : ADULT CONSENT FORM

(This part to be completed by the patient *(circle one)*

1. Have you read the Patient Information Sheet? YES/NO
(please take a copy home with you to keep)

2. Have you had an opportunity to discuss the study and ask any questions? YES/NO

3. Have you had satisfactory answers to all of your questions? YES/NO

4. Have you received enough information about the study? YES/NO

5. Who has given you an explanation about the study?

   Dr/Nurse ______________________

6. Do you understand that you are free to withdraw from the study:
   - At any time?
   - Without having to give a reason? YES/NO
   - Without affecting your future medical care?

1. Sections of your medical notes relating to your participation in the study may be inspected by responsible individuals from BPL or from regulatory authorities. All personal details will be treated as strictly confidential.

   Do you give permission for these individuals to have access to your records? YES/NO

2. Has the doctor discussed the circumstances when compensation may be due? YES/NO

3. Have you had sufficient time to come to your decision? YES/NO

4. Do you consent to your blood being tested for hepatitis B, C and HIV viruses? YES/NO

5. Do you agree to take part in the study? YES/NO

PATIENT *(Please sign below and date your own signature)*

Signed: ______________________ Date: ______________

Print Name: ______________________

INVESTIGATOR

Signed: ______________________ Date: ______________

Print Name: ______________________

APPENDIX 6: CHILDREN'S CONSENT FORM

This form should be completed and signed by the patient if they are able to understand the study or if not, by the parent/guardian

	(circle one)
1. Have you read the Information Sheet? (*please take a copy home with you to keep*)	YES/NO
2. Have you had enough information about the study and had a chance to ask any questions?	YES/NO
3. Do you understand all the answers to your questions?	YES/NO
4. Who has talked to you about the study? Dr/Nurse ____________________	
5. Do you understand that you can withdraw from the study: • At any time? • Without having to say why? • Without affecting your care?	YES/NO
6. Some of your medical notes may be looked at by people from BPL or from the Government. They will be kept secret. Will you let these people look at your notes?	YES/NO
7. Has the doctor told you when compensation may be due?	YES/NO
8. Have you had enough time to make your mind up?	YES/NO
9. Do you agree to your blood being tested for bugs (hepatitis B and C viruses?)	YES/NO
10. Do you want to take part in the study?	YES/NO

PARENT/GUARDIAN *(Please answer the questions, sign below and date your own signature)*

Is your child able to understand the nature of the trial and give consent to take part? YES/NO

If YES, will the consent be verbal or written? Verbal/Written/NA

Signed: ____________________ Date: ____________________

Print Name: ____________________ Relationship: ____________________

CHILD *(Please sign below and add the date)*

Signed: ____________________ Date: ____________________

Print Name: ____________________

INVESTIGATOR

Signed: ____________________ Date: ____________________

Print Name: ____________________

APPENDIX 7: PATIENT INFORMATION LEAFLET FOR EXTRA BLOOD TESTS (PHARMACOKINETICS)

A MULTI-CENTRE, OPEN STUDY TO ASSESS THE SAFETY AND EFFICACY OF A 16% IMMUNOGLOBULIN PRODUCT GIVEN VIA THE SUBCUTANEOUS ROUTE IN PRIMARY ANTIBODY DEFICIENT PATIENTS

This purpose of this leaflet is to tell you about an extra part to the research study in which you are already taking part in. You do not have to do this extra part if you do not want to and if there is anything which you do not understand or if you require more information, please ask your doctor or nurse.

Why are the extra blood tests being done?

The reason we would like to do these extra blood tests is so we can follow, on a daily basis, what happens to the level of antibodies in your blood after you have had the first subcutaneous infusion and again after infusion 18, 3 months later. This will help us to understand more about how the immunoglobulin affects your condition. It will also allow us to check that the new treatment produces suitable levels of immunoglobulin in your blood.

What would I have to do?

If you agreed to the extra tests, you would need to have some blood taken every day for one week at about the same time each day. This would be done twice during the study after the first study subcutaneous infusion and again after infusion number 18 (3 months later) making a total of two weeks extra blood sampling. How and when the blood would be collected would be discussed with you by your doctor or nurse.

Are there any risks involved?

The blood tests may hurt slightly and cause slight bruising. Having the extra blood taken would not cause you any harm overall however and you can change your mind at any time about doing them without it affecting the rest of the study or your future medical care.

APPENDIX 8: PHARMACOKINETICS CONSENT FORM

(This part to be completed by the patient) *(circle one)*

1. Have you read the Patient Information Sheet? *(please take a copy home with you to keep)* YES/NO

2. Have you had an chance to discuss the extra blood tests and ask any questions? YES/NO

3. Have you had satisfactory answers to all of your questions? YES/NO

4. Have you received enough information about the extra blood tests? YES/NO

5. Who has given you an explanation about the extra blood tests?

   Dr/Nurse ______________________

6. Do you understand that you are free to stop the extra blood tests:
   - At any time?
   - Without having to give a reason? YES/NO
   - Without affecting the rest of the study or your future medical care?

1. Have you had enough time to come to your decision? YES/NO

2. Do you agree to the extra blood tests? YES/NO

PATIENT *(Please sign below and date your own signature)*

Signed: ______________________ Date: ______________

Print Name: ______________________

INVESTIGATOR *(Please sign below and date your own signature)*

Signed: ______________________ Date: ______________

Print Name: ______________________

APPENDIX 9: EXAMPLE GP LETTER

Our ref: SCIG01/BPL

«GPNAME»
«GPADDRESS»

Dear «GPNAME»

Your patient, «PATNAME», has volunteered to take part in an open study to assess the safety and efficacy of a 16% immunoglobulin preparation given via the subcutaneous route in primary antibody deficiency.

Your patient will receive weekly infusions of subcutaneous immunoglobulin at an initial dose of 100mg/kg. The dose will then be tailored to maintain an IgG level of at least 6g/l. The treatment duration will be 6 months but at the end of this period your patient may opt to participate in the follow-on safety phase of the study and continue to receive weekly infusions until such time they decide to withdraw or the product is marketed.

Your patient may be considered suitable to administer their infusions at home after a period of training. If this is the case, the study staff will contact you with the relevant details and seek your agreement before your patient commences home therapy.

Your patient will have undergone a medical examination, including blood tests, before being included in the study. He/she will have close medical monitoring during the infusion and at the end of the study he/she will have another thorough medical.

The trial is being carried out to Good Clinical Practice and has been approved by the Multi Centre Research Ethics Committee (MREC) and the Local Research Ethics Committee (LREC). If there is any information regarding your patient's health, which may be relevant to participation in this study, please can you let me know.

If I can be of any further assistance, please do not hesitate to contact me«INVESTNAME» on «INVESTNO»

Yours sincerely,

«INVESTNO»
«INVESTNO»

APPENDIX 10: HOME THERAPY MANUAL

Patient Information

For patients who have primary antibody deficiency, there are several ways of replacing the missing antibodies such as by intramuscular injection or by intravenous infusion. In the past, slow subcutaneous infusions were given but these infusions proved cumbersome and entailed overnight stays in hospital. There is now a new method of replacing the antibodies by rapid subcutaneous infusion which is easy to learn and is advantageous for patients who would be suitable for home therapy.

From recent studies performed in Sweden, immunoglobulin replacement therapy by subcutaneous infusion has proved to be a safe, efficient, timesaving, cost-effective and convenient form of administration. Your doctor thinks that this method would be suitable for you to try. Before you can perform home therapy however you need to satisfy the following criteria:

1. A relative/friend who has also been trained in the techniques must be available to assist with each infusion performed at home

2. You and your relative/friend must be motivated to perform self-infusion

3. After a period of training by the study staff, both you and your relative/friend must be able to manage the infusion techniques

4. Your G.P. must agree to you performing home therapy

5. You must be contactable by telephone.

The training will take place over a period of 8-10 weeks when you come for your study infusions. You and your relative/friend will be shown how to draw the immunoglobulin up from the ampoule into the syringe, insert the butterfly needle under the skin in the thigh or abdomen and connect the syringe to the battery powered syringe driver. The study staff will also show you how to calculate the correct dose and infusion rate.

Materials Required

- Sterile paper towel
- Medi-swabs
- Needles for drawing up (19G)
- Syringes (10ml)
- Infusion set
- Butterfly needle (28G)
- Tape
- Cotton wool
- Sharps bin
- Syringe drivers (Graseby MS16A)
- Subcutaneous immunoglobulin
- Adrenaline

Temperature Record

You must record your temperature on the infusion diary prior to starting the infusion. If this is above the level specified by your study staff ring the clinic and ask for advice before infusing. Also, an infusion should not be administered if you are feverish or are unwell

Method

1. Check syringe drivers and set rate as prescribed
2. Wash hands
3. Clean surface and lay paper towel
4. Check immunoglobulin for correct dose and expiry date
5. Remove vial tops and draw up immunoglobulin with syringe and needle
6. Attach infusion needle to syringe and prime tubing
7. Prepare tape and wipe site (abdomen/thigh) to be used with medi-swab
8. Insert the needle into the abdomen/thigh as instructed and tape firmly in place
9. Check for any blood return by withdrawing the syringe plunger and by removing the syringe from the tubing
10. If blood returns re-site the needle and repeat 9
11. Once position is satisfactory with no blood return, place the syringe on the driver and secure in place
12. Switch on the driver and administer infusion
13. When the alarm sounds remove the needle and dispose of in the sharps bin
14. Repeat process if further dose is needed
15. At the end of the infusion dispose of all used materials, wash hands and record details

APPENDIX 11: HOME THERAPY CONSENT FORM

(This part to be completed by the patient/parent/guardian *(circle one)*

1. Have you read the home therapy information? YES/NO
2. Have you had an opportunity to discuss home therapy and ask any questions? YES/NO
3. Have you had satisfactory answers to all of your questions? YES/NO
4. Have you received enough information about home therapy? YES/NO
5. Who has given you an explanation about home therapy?

   Dr/Nurse ______________________

6. Do you have a relative or friend who is also willing to be trained on home therapy and who will be available to assist you with each home infusion? YES/NO
7. Has your GP been consulted and his agreement for you to commence home therapy been sought? YES/NO
8. Do you agree to be trained to infuse your/your child's subcutaneous immunoglobulin? YES/NO

PATIENT/PARENT/GUARDIAN *(Please sign below and date your own signature)*

Signed: ______________________ Date: ______________

Print Name: ______________________ Relationship: ______________
(if applicable)

RELATIVE/FRIEND *(Please sign below and date your own signature)*

Signed: ______________________ Date: ______________

Print Name: ______________________ Relationship: ______________
(if applicable)

INVESTIGATOR

Signed: ______________________ Date: ______________

Print Name: ______________________

APPENDIX 12: HOME THERAPY INFUSION DIARY CARD

Maximum temperature for this patient before advice should be sought: ☐☐.☐ °C

If your temperature prior to the infusion is above this figure then you must contact the study staff for advice: Contact Number: ______________________________

Date	Temp °C	Site*	Dose per Site	Total Dose	Rate mm/hr	Batch No.	Comments/Problems

* A = Abdomen
T = Thigh

APPENDIX 13: LOCAL INVESTIGATOR'S RESPONSIBILITIES BASED ON THE ICH GUIDELINES FOR GOOD CLINICAL PRACTICE

1. LOCAL INVESTIGATOR

1.1 Investigator's Qualifications and Agreements

1.1.1 The Investigator(s) should be qualified by education, training, and experience to assume responsibility for the proper conduct of the study, should meet all the qualifications specified by the applicable regulatory requirement(s), and should provide evidence of such qualifications through up-to-date curriculum vitae and/or other relevant documentation requested by the sponsor, the LREC, and/or the regulatory authority(ies).

1.1.2 The Investigator should be thoroughly familiar with the appropriate use of the investigational product(s), as described in the protocol, in the current Investigator's Brochure, in the product information and in other information sources provided by the sponsor.

1.1.3 The Investigator should be aware of, and should comply with, GCP and the applicable regulatory requirements.

1.1.4 The Investigator should permit monitoring and auditing by the sponsor, and inspection by the appropriate regulatory authority(ies).

1.1.5 The Investigator should maintain a list of appropriately qualified persons to whom the Investigator has delegated significant study-related duties.

1.2 Adequate Resources

1.2.1 The Investigator should be able to demonstrate (e.g. based on retrospective data) a potential for recruiting the required number of suitable subjects within the agreed recruitment period.

1.2.2 The Investigator should have sufficient time to properly conduct and complete the study within the agreed study period.

1.2.3 The Investigator should have available an adequate number of qualified staff and adequate facilities for the foreseen duration of the study to conduct the study properly and safely.

1.2.4 The Investigator should ensure that all persons assisting with the study are adequately informed about the protocol, the investigational product(s), and their study-related duties and functions.

1.3 Medical Care of Study Subjects

1.3.1 A qualified physician (or dentist, when appropriate), who is an Investigator or a sub-Investigator for the study, should be responsible for all study-related medical (or dental) decisions.

1.3.2 During and following a subject's participation in a study, the Investigator should ensure that adequate medical care is provided to a subject for any adverse events, including clinically significant laboratory values, related to the study. The Investigator should inform a subject when medical care is needed for intercurrent illness(es) of which the Investigator becomes aware.

1.3.3 It is recommended that the Investigator inform the subject's primary physician about the subject's participation in the study if the subject has a primary physician and if the subject agrees to the primary physician being informed.

1.3.4 Although a subject is not obliged to give his/her reason(s) for withdrawing prematurely from a study, the Investigator should make a reasonable effort to ascertain the reason(s), while fully respecting the subject's rights.

1.4 Communication with LREC

1.4.1 Before initiating a study, the Investigator should have written and dated approval/favourable opinion from the LREC for the study protocol, written informed consent form, consent form updates, subject recruitment procedures (e.g., advertisements), and any other written information to be provided to subjects.

1.4.2 As part of the Investigator's written application to the LREC, the Investigator should provide the LREC with a current copy of the MREC approval documentation.

Data entered on the CRFs must be subject to validation. According to Good Clinical Practice, this requires that the study CRA should compare data on the CRF with the raw data, e.g. patient notes and laboratory data.

The study CRA will require access to all patient records to allow verification of the entries in the CRF.

The Investigator will agree to make himself available to correct or discuss any discrepancies.

An audit may also be carried out at the centre to ensure that the study has complied with Good Clinical Practice (GCP).

Access to documentation and facilities used during the study may be required by BPL appointed auditors or by regulatory authorities. In the event that an audit is scheduled by the regulatory authorities, the Investigator must notify BPL immediately.

18.5 Report and Publication

The key authors will be from those centres who have entered patients into the study during the recruitment period. Each centre will be responsible for determining the author from that centre. No more than two authors per centre. The centre that recruits the greatest number of patients will be cited first.

The Investigator is obliged to provide BPL with complete test results and all data and reports within 6 weeks of completion or termination of the study.

Should the Investigator wish to publish the results of the study, a copy of the manuscript will be provided to BPL at least 30 days prior to the expected date of submission to the intended publisher.

1.4.3 During the study the Investigator should provide to the LREC all documents subject to review.

1.5 Compliance with Protocol

1.5.1 The Investigator should conduct the study in compliance with the protocol agreed to by the sponsor and, if required, by the regulatory authority(ies) and which was given approval/favourable opinion by the MREC. The Investigator and the sponsor should sign the protocol, or an alternative contract, to confirm agreement.

1.5.2 The Investigator should not implement any deviation from, or changes of the protocol without agreement by the sponsor and prior review and documented approval/favourable opinion from the MREC of an amendment, except where necessary to eliminate an immediate hazard(s) to study subjects, or when the change(s) involves only logistical or administrative aspects of the study (e.g. change in monitor(s), change of telephone number(s)).

1.5.3 The Investigator, or person designated by the Investigator, should document and explain any deviation from the approved protocol.

1.5.4 The Investigator may implement a deviation from, or a change of, the protocol to eliminate an immediate hazard(s) to study subjects without prior MREC approval/favourable opinion. As soon as possible, the implemented deviation or change, the reasons for it, and, if appropriate, the proposed protocol amendment(s) should be submitted.

a) to the MREC for review and approval/favourable opinion,
b) to the sponsor for agreement and, if required,
c) to the regulatory authority(ies).

1.6 Investigational Product(s)

1.6.1 Responsibility for investigational product(s) accountability at the study site(s) rests with the Investigator.

1.6.2 Where allowed/required, the Investigator may/should assign some or all of the Investigator's duties for investigational product(s) accountability at the study site(s) to an

appropriate pharmacist or another appropriate individual who is under the supervision of the Investigator.

1.6.3 The Investigator and/or a pharmacist or other appropriate individual, who is designated by the Investigator, should maintain records of the product's delivery to the study site, the inventory at the site, the use by each subject, and the return to the sponsor or alternative disposition of unused product(s). These records should include dates, quantities, batch/serial numbers expiration dates (if applicable), and the unique code numbers assigned to the investigational product(s) and study subjects. Investigators should maintain records that document adequately that the subjects were provided the doses specified by the protocol and reconcile all investigational product(s) received from the sponsor.

1.6.4 The investigational product(s) should be stored as specified by the sponsor and in accordance with applicable regulatory requirement(s).

1.6.5 The Investigator should ensure that the investigational product(s) are used only in accordance with the approved protocol.

1.6.6 The Investigator, or a person designated by the Investigator, should explain the correct use of the investigational product(s) to each subject and should check, at intervals appropriate for the study, that each subject is following the instructions properly.

1.7 Randomisation Procedures and Unblinding

The Investigator should follow the study's randomisation procedures, if any, and should ensure that the code is broken only in accordance with the protocol. If the study is blinded, the Investigator should promptly document and explain to the sponsor any premature unblinding,(e.g., accidental unblinding, unblinding due to a serious adverse event) of the investigational product(s).

1.8 Informed Consent of Study Subjects

1.8.1 In obtaining and documenting informed consent, the Investigator should comply with the applicable regulatory requirement(s), and should adhere to GCP and to the ethical principles that have their origin in the Declaration of Helsinki. Prior to the beginning of the study, the

Investigator should have the MREC and LREC's written approval/favourable opinion of the written informed consent form and any other written information to be provided to subjects.

1.8.2 The written informed consent form and any other written information to be provided to subjects should be revised whenever important new information becomes available that may be relevant to the subject's consent. Any revised written informed consent form, and written information should receive the MREC and LREC's approval/favourable opinion in advance of use. The subject or the subject's legally acceptable representative should be informed in a timely manner if new information becomes available that may be relevant to the subject's willingness to continue participation in the study. The communication of this information should be documented.

1.8.3 Neither the Investigator, nor the study staff, should coerce or unduly influence a subject to participate or to continue to participate in a study.

1.8.4 None of the oral and written information concerning the study, including the written informed consent form, should contain any language that causes the subject or the subject's legally acceptable representative to waive or to appear to waive any legal rights, or that releases or appears to release the Investigator, the institution, the sponsor, or their agents from liability for negligence.

1.8.5 The Investigator, or a person designated by the Investigator, should fully inform the subject or, if the subject is unable to provide informed consent, the subject's legally acceptable representative, of all pertinent aspects of the study including the written information given approval/favourable opinion by the MREC and LREC.

1.8.6 The language used in the oral and written information about the study, including the written informed consent form, should be as non-technical as practical and should be understandable to the subject or the subject's legally acceptable representative and the impartial witness, where applicable.

1.8.7 Before informed consent may be obtained, the Investigator, or a person designated by the Investigator, should provide the subject or the subject's legally acceptable representative ample time and opportunity to inquire about details of the study and to decide whether or not to

participate in the study. All questions about the study should be answered to the satisfaction of the subject or the subject's legally acceptable representative.

1.8.8 Prior to a subject's participation in the study, the written informed consent form should be signed and personally dated by the subject or by the subject's legally acceptable representative, and by the person who conducted the informed consent discussion.

1.8.9 If a subject is unable to read or if a legally acceptable representative is unable to read, an impartial witness should be present during the entire informed consent discussion. After the written informed consent form and any other written information to be provided to subjects, is read and explained to the subject or the subject's legally acceptable representative, and after the subject's legally acceptable representative has orally consented to the subject's participation in the study and, if capable of doing so, has signed and personally dated the informed consent form, the witness should sign and personally date the consent form. By signing the consent form, the witness attests that the information in the consent form and any other written information was accurately explained to, and apparently understood by, the subject or the subject's legally acceptable representative, and that informed consent was freely given by the subject or the subject's legally acceptable representative.

1.8.10 Both the informed consent discussion and the written informed consent form and any other written information to be provided to subjects should include explanations of the following:

a) That the study involves research.
b) The purpose of the study.
c) The study treatment(s) and the probability for random assignment to each treatment.
d) The study procedures to be followed, including all invasive procedures.
e) The subject's responsibilities.
f) Those aspects of the study that are experimental.
g) The reasonably foreseeable risks or inconveniences to the subject and, when applicable, to an embryo, fetus, or nursing infant.
h) The reasonably expected benefits. When there is no intended clinical benefit to the subject, the subject should be made aware of this.

i) The alternative procedure(s) or course(s) of treatment that may be available to the subject, and their important potential benefits and risks.

j) The compensation and/or treatment available to the subject in the event of study-related injury.

k) The anticipated prorated payment, if any, to the subject for participating in the study.

l) The anticipated expenses, if any, to the subject for participating in the study.

m) That the subject's participation in the study is voluntary and that the subject may refuse to participate or withdraw from the study, at any time, without penalty or loss of benefits to which the subject is otherwise entitled.

n) That the monitor(s), the auditor(s), the MREC and LREC, and the regulatory authority(ies) will be granted direct access to the subject's original medical records for verification of clinical study procedures and/or data, without violating the confidentiality of the subject, to the extent permitted by the applicable laws and regulations and that, by signing a written informed consent form, the subject or the subject's legally acceptable representative is authorising such access.

o) That records identifying the subject will be kept confidential and, to the extent permitted by the applicable laws and/or regulations, will not be made publicly available. If the results of the study are published, the subject's identity will remain confidential.

p) That the subject or the subject's legally acceptable representative will be informed in a timely manner if information becomes available that may be relevant to the subject's willingness to continue participation in the study.

q) The person(s) to contact for further information regarding the study and the rights of study subjects, and whom to contact in the event of study-related injury.

r) The foreseeable circumstances and/or reasons under which the subject's participation in the study may be terminated.

s) The expected duration of the subject's participation in the study.

t) The approximate number of subjects involved in the study.

1.8.11 Prior to participation in the study, the subject or the subject's legally acceptable representative should receive a copy of the signed and dated written informed consent form and any other written information provided to the subjects. During a subject's participation in the

study, the subject or the subject's legally acceptable representative should receive a copy of the signed and dated consent form updates and a copy of any amendments to the written information provided to subjects.

1.8.12 When a clinical study (therapeutic or non-therapeutic) includes subjects who can only be enrolled in the study with the consent of the subject's legally acceptable representative (e.g., minors, or patients with severe dementia), the subject should be informed about the study to the extent compatible with the subject's understanding and, if capable, the subject should sign and personally date the written informed consent.

1.8.13 Except as described in 1.8.14, a non-therapeutic study (i.e. a study in which there is not anticipated direct clinical benefit to the subject), should be conducted in subjects who personally give consent and who sign and date the written informed consent form.

1.8.14 Non-therapeutic studies may be conducted in subjects with consent of a legally acceptable representative provided the following conditions are fulfilled:

a) The objectives of the study can not be met by means of a study in subjects who can give informed consent personally.

b) The foreseeable risks to the subjects are low.

c) The negative impact on the subject's well-being is minimised and low.

d) The study is not prohibited by law.

e) The approval/favourable opinion of the MREC and LREC is expressly sought on the inclusion of such subjects, and the written approval/favourable opinion covers this aspect.

Such studies, unless an exception is justified, should be conducted in patients having a disease or condition for which the investigational product is intended. Subjects in these studies should be particularly closely monitored and should be withdrawn if they appear to be unduly distressed.

1.8.15 In emergency situations, when prior consent of the subject is not possible, the consent of the subject's legally acceptable representative, if present, should be requested. When prior consent of the subject is not possible, and the subject's legally acceptable representative is not

available, enrolment of the subject should require measures described in the protocol and/or elsewhere, with documented approval/favourable opinion by the MREC and LREC, to protect the rights, safety and well-being of the subject and to ensure compliance with applicable regulatory requirements. The subject or the subject's legally acceptable representative should be informed about the study as soon as possible and consent to continue and other consent as appropriate (see 1.8.10) should be requested.

1.9 Records and Reports

1.9.1 The Investigator should ensure the accuracy, completeness, legibility, and timeliness of the data reported to the sponsor in the CRFs and in all required reports.

1.9.2 Data reported on the CRF, that are derived from source documents, should be consistent with the source documents or the discrepancies should be explained.

1.9.3 Any change or correction to a CRF should be dated, initialled, and explained (if necessary) and should not obscure the original entry (i.e. an audit study should be maintained): this applies to both written and electronic changes or corrections (see 1.18.4 (n)). Sponsors should provide guidance to Investigators and/or the Investigators' designated representatives on making such corrections. Sponsors should have written procedures to assure that changes or corrections in CRFs made by sponsor's designated representatives are documented, are necessary, and are endorsed by the Investigator. The Investigator should retain records of the changes and corrections.

1.9.4 The Investigator should maintain the study documents as specified in Essential Documents for the Conduct of a Clinical Study and as required by the applicable regulatory requirement(s). The Investigator should take measures to prevent accidental or premature destruction of these documents.

1.9.5 Essential documents should be retained until at least 2 years after the last approval of a marketing application in an ICH region and until there are no pending or contemplated marketing applications in an ICH region or at least 2 years have elapsed since the formal discontinuation of clinical development of the investigational product. These documents should be retained for a

longer period however if required by the applicable regulatory requirements or by an agreement with the sponsor. It is the responsibility of the sponsor to inform the Investigator as to when these documents no longer need to be retained.

1.9.6 The financial aspects of the study should be documented in an agreement between the sponsor and the Investigator.

1.9.7 Upon request of the monitor, auditor, MREC, LREC, or regulatory authority, the Investigator should make available for direct access all requested study-related records.

1.10 Progress Reports

1.10.1 The Investigator should submit written summaries of the study status to the LREC annually, or more frequently, if requested by the LREC.

1.10.2 The Investigator should promptly provide written reports to the sponsor, the LREC and, where applicable, the institution on any changes significantly affecting the conduct of the study, and/or increasing the risk to subjects.

1.11 Safety Reporting

1.11.1 All serious adverse events (SAEs) should be reported immediately to the sponsor except for those SAEs that the protocol or other document (e.g., Investigator's Brochure) identifies as not needing immediate reporting. The immediate reports should be followed promptly by detailed, written reports. The immediate and follow-up reports should identify subjects by unique code numbers assigned to the study subjects rather than by the subjects' names, personal identification numbers, and/or addresses. The Investigator should also comply with the applicable regulatory requirement(s) related to the reporting of unexpected serious adverse drug reactions to the regulatory authority(ies) and the LREC.

1.11.2 Adverse events and/or laboratory abnormalities identified in the protocol as critical to safety evaluations should be reported to the sponsor according to the reporting requirements and within the time periods specified by the sponsor in the protocol.

1.11.3 For reported deaths, the Investigator should supply the sponsor and the LREC with any additional requested information (e.g., autopsy reports and terminal medical reports).

1.12 Premature Termination or Suspension of a Study

If the study is prematurely terminated or suspended for any reason, the Investigator should promptly inform the study subjects, should assure appropriate therapy and follow-up for the subjects, and, where required by the applicable regulatory requirement(s), should inform the regulatory authority(ies). In addition:

1.12.1 If the Investigator terminates or suspends a study without prior agreement of the sponsor, the Investigator should inform the institution where applicable, and the Investigator should promptly inform the sponsor and the LREC, and should provide the sponsor and the LREC a detailed written explanation of the termination or suspension.

1.12.2 If the sponsor terminates or suspends a study, the Investigator should promptly inform the institution where applicable and the Investigator should promptly inform the LREC and provide the LREC a detailed written explanation of the termination or suspension.

1.12.3 If the MREC or LREC terminates or suspends its approval/favourable opinion of a study, the Investigator should inform the institution where applicable and the Investigator should promptly notify the sponsor and provide the sponsor with a detailed written explanation of the termination or suspension.

1.13 Final Report(s) by Investigator

Upon completion of the study, the Investigator, where applicable, should inform the institution: the Investigator should provide the LREC with a summary of the study's outcome, and the regulatory authority(ies) with any reports required.

APPENDIX 14: DECLARATION OF HELSINKI

Recommendations guiding physicians in biomedical research involving human subjects

Adopted by the 18th World Medical Assembly, Helsinki, Finland, June 1964 and amended in Tokyo, 1975, in Venice, 1983, in Hong Kong, 1989, and in South Africa, October 1996.

Introduction

It is the mission of the physician to safeguard the health of the people. His or her knowledge and conscience are dedicated to the fulfilment of this mission.

The Declaration of Geneva of the World Medical Association binds the physician with the words, "The health of my patient will be my first consideration", and the International Code of Medical Ethics declares that, "A physician shall act only in the patient's interest when providing medical care which might have the effect of weakening the physical and mental condition of the patient".

The purpose of biomedical research involving human subjects must be to improve diagnostic, therapeutic and prophylactic procedures and the understanding of the aetiology and pathogenesis of disease.

In current medical practice most diagnostic, therapeutic or prophylactic procedures involve hazards. This applies especially to biomedical research.

Medical progress is based on research which ultimately must rest in part on experimentation involving human subjects.

In the field of biomedical research a fundamental distinction must be recognised between medical research in which the aim is essentially diagnostic or therapeutic for a patient, and medical research, the essential object of which is purely scientific and without implying direct diagnostic or therapeutic value to the person subjected to the research.

Special caution must be exercised in the conduct of research which may affect the environment, and the welfare of animals used for research must be respected.

Because it is essential that the results of laboratory experiments be applied to human beings to further scientific knowledge and to help suffering humanity, the World Medical Association has prepared the following recommendations as a guide to every physician in biomedical research involving human subjects. They should be kept under review in the future. It must be stressed that the standards as drafted are only a guide to physicians all over the world. Physicians are not relieved from criminal, civil and ethical responsibilities under the laws of their own countries.

I. Basic principles

1. Biomedical research involving human subjects must conform to generally accepted scientific principles and should be based on adequately performed formed laboratory and animal experimentation and on a thorough knowledge of the scientific literature.

2. The design and performance of each experimental procedure involving human subjects should be clearly formulated in an experimental protocol which should be transmitted for consideration, comment and guidance to a specially appointed committee independent of the investigator and the sponsor provided that this independent committee is in conformity with the laws and regulations of the country in which the research experiment is performed.

3. Biomedical research involving human subjects should be conducted only by scientifically qualified persons and under the supervision of a clinically competent medical person. The responsibility for the human subject must always rest with a medically qualified person and never rest on the subject of the research, even though the subject has given his or her consent.

4. Biomedical research involving human subjects cannot legitimately be carried out unless the importance of the objective is in proportion to the inherent risk to the subject.

5. Every biomedical research project involving human subjects should be preceded by careful assessment of predictable risks in comparison with foreseeable benefits to the subject or to others. Concern for the interests of the subject must always prevail over the interests of science and society.

6. The right of the research subject to safeguard his or her integrity must always be respected. Every precaution should be taken to respect the privacy of the subject and to minimise the impact of the study on the subject's physical and mental integrity and on the personality of the subject.

7. Physicians should abstain from engaging in research projects involving human subjects unless they are satisfied that the hazards involved are believed to be predictable. Physicians should cease any investigation if the hazards are found to outweigh the potential benefits.

8. In publication of the results of his or her research, the physician is obliged to preserve the accuracy of the results. Reports of experimentation not in accordance with the principles laid down in this Declaration should not be accepted for publication.

9. In any research on human beings, each potential subject must be adequately informed of the aims, methods, anticipated benefits and potential hazards of the study and the discomfort it may entail. He or she should be informed that he or she is at liberty to abstain from participation in the study and that he or she is free to withdraw his or her consent to participation at any time. The physician should then obtain the subject's freely given informed consent, preferably in writing.

10. When obtaining informed consent for the research project the physician should be particularly cautious if the subject is in a dependent relationship to him or her or may consent under duress. In that case the informed consent should be obtained by a physician who is not engaged in the investigation and who is completely independent of this official relationship.

11. In the case of legal incompetence, informed consent should be obtained from the legal guardian in accordance with national legislation. Where physical or mental incapacity makes it impossible to obtain informed consent, or when the subject is a minor, permission from the responsible relative replaces that of the subject in accordance with national legislation.

Whenever the minor child is in fact able to give a consent, the minor's consent must be obtained in addition to the consent of the minor's legal guardian.

12. The research protocol should always contain a statement of the ethical considerations involved and should indicate that the principles enunciated in the present Declaration are complied with.

II. Medical research combined with professional care (clinical research)

1. In the treatment of the sick person, the physician must be free to use a new diagnostic and therapeutic measure, if in his or her judgement it offers hope of saving life, re-establishing health or alleviating suffering.

2. The potential benefits, hazards and discomfort of a new method should be weighed against the advantages of the best current diagnostic and therapeutic methods.

3. In any medical study, every patient - including those of a control group, if any - should be assured of the best proven diagnostic and therapeutic method. This does not exclude the use of inert placebo in studies where no proven diagnostic or therapeutic method exists.

4. The refusal of the patient to participate in a study must never interfere with the physician-patient relationship.

5. If the physician considers it essential not to obtain informed consent, the specific reasons for this proposal should be stated in the experimental protocol for transmission to the independent Committee.

6. The physician can combine medical research with professional care, the objective being the acquisition of new medical knowledge, only to the extent that medical research is justified by its potential diagnostic or therapeutic value for the patient.

III. Non therapeutic biomedical research involving human subjects (non-clinical biomedical research)

1. In the purely scientific application of medical research carried out on a human being, it is the duty of the physician to remain the protector of the life and health of that person on whom biomedical research is being carried out.

2. The subjects should be volunteers - either healthy persons or patients for whom the experimental design is not related to the patient's illness.

3. The investigator or the investigating team should discontinue the research if in his/her or their judgement it may, if continued, be harmful to the individual. . In research on man, the interest of science and society should never take precedence over considerations related to the well-being of the subject.

APPENDIX 15: STUDY PROCEDURES

	PHASE 1				PHASE 2		PHARMACOKINETICS	
	**Pre-study**	**Infusions 1-3**<br><br>**Routine IVIG/SCIG**	**Infusions 4-30**<br><br>**SCIG**	**End of Phase 1**	**3 monthly**<br><br>**SCIG**	**End of Phase 2**	**PK 1**<br><br>**SCIG**	**PK 2**<br><br>**SCIG**
Informed Consent	X							
Inclusion /Exclusion Criteria	X							
Medical History	X							
Physical Examination	X			X				
Vital Signs	X	X	X	X		X	X	X
Patient Satisfaction Questionnaire	X		X (infusion 16)	X				
Site Inspection		X	X				X	X
Home therapy training			X					
Concomitant medications	X	X	X	X	X		X	X
Adverse event reporting	X	X	X	X	X		X	X
**Haematology**: Haemoglobin, Haematocrit, RBC, WBC, Neutrophils, Lymphocytes, Monocytes, Eosinophils, Basophils, Platelet Count, Reticulocytes	X	if clinically indicated	if clinically indicated	X	if clinically indicated		if clinically indicated	if clinically indicated
**Biochemistry**: Sodium, Potassium, Creatinine, LDH, GGT, Total Bilirubin, Alkaline Phosphatase, Haptoglobin	X	if clinically indicated	if clinically indicated	X	if clinically indicated		if clinically indicated	if clinically indicated
**Blood Group Serology**: ABO (D), Direct Coombs	X							
**Immunology**: IgG, IgA, IgM	X	X	X	X	if clinically indicated		X	X
**Liver Function**: ALT (and/or AST)	X		X (every 4 weeks)					
**Specific Antibodies**: Pneumococcus, HIB	X		X*					
**Virology**:HBsAg, HIV adults only(PCR), HCV (PCR)			X (infusion 4)	X$^{\pi}$	X$^{\phi}$			
**Parvovirus**: Parvovirus B19 (PCR)			X$^{\propto}$					
**Archive**: Store at -70°C for 15 years			X	X$^{\pi}$	X$^{\phi}$			
**Repeat Test** : Store at -20°C for repeat testing	X	X	X	X	XΨ		X	X

* 4 weekly samples
** Baseline sample stored at -70°C and tested only if end of study sample positive
Ψ Repeat sample to be taken only if other samples clinically indicated

π Sample to be taken only if patient withdraws from Phase 1 or **not** entering Phase 2
ϕ Sample to be taken if patient withdraws or completes Phase 2
∝ Pre 1st dose &1 week post 1st dose

Protocol Amendment Form

Title of Trial: **A MULTI-CENTRE, OPEN STUDY TO ASSESS THE SAFETY AND EFFICACY OF A 16% IMMUNOGLOBULIN PRODUCT GIVEN VIA THE SUBCUTANEOUS ROUTE IN PRIMARY ANTIBODY DEFICIENT PATIENTS**

Trial Code: SCIG01

From: Final Protocol, Version 2; September 1999

To: Final Protocol, Version 3; April 2000

Section/ Page No	Details of Amendment	Justification of Amendment
Throughout protocol	Deletion of: Tel: 0181 258 2565<br>Fax 0181 258 2611<br>Addition of: Tel 020 8258 2565<br>Fax 020 8258 2611	All telephone and fax numbers changed to reflect BT alterations to London numbers
Throughout protocol	Deletion of: 'concurrent medication<br>Addition of: concomitant medication	To reflect current BPL practice
Throughout protocol	Deletion of all references to the amount of blood to be collected for each sample.	To allow for difference in blood collection requirements between the centres.
	Removal of Pauline Jackson as responsible CRA	Pauline Jackson has left the company
i	Removal of complete page	Not required
2.0 Synopsis/Page 2	Deletion of: 'Upto 40 adult patients and paediatric patients, aged 0-no upper limit',<br>Addition of: 'A total of 40 patients (paediatric 0-15 years, adults 16+years)'	Clarification of the age range of paediatric and adult patients to be enrolled.

Protocol Amendment Form

Section/ Page No	Details of Amendment	Justification of Amendment
2.0 Synopsis/Page 2	Deletion of: 'but a minimum target IgG trough level of 6g/L will be aimed for.', Addition of: 'but a minimum target IgG trough level of 4g/L for paediatrics and 6g/L for adults will be aimed for.'	Lower trough level for paediatric patients recommended by principle investigator, Dr Gooi.
2.0 Synopsis/Page 3 (Table)	Deletion of complete section for Phase 1 and Pharmacokinetics Visit Details. Addition of: Selected patients will be asked to attend for 5 consecutive clinic visits after their first study SCIG infusion. This will be repeated after 3 months of SCIG.	Clarifications of visit details.
2.0 Synopsis/Page 3 (Table)	Deletion of PK1 a-g (optional) PK2 a-g (optional) Addition of PK1 (optional) PK2 (optional)	Clarification of protocol.
4.0 Study Objectives/ Page 6	Deletion of 'to achieve a minimum trough serum IgG level of 6g/L.' Addition of 'to achieve a minimum trough levels of IgG of 4g/L for paediatrics and 6g/L for adults'	To reflect the different trough levels required for paediatric and adult patients.
7.2 Dose and route of administration/ Page 8	Deletion of 'but a minimum target IgG trough level of 6g/L will be aimed for.' Addition of but a minimum target IgG trough level of 4g/L for paediatrics and 6g/L for adults will be aimed for.'	To reflect the different trough levels required for paediatric and adult patients.

Protocol Amendment Form

Section/ Page No	Details of Amendment	Justification of Amendment
8.1 Pre-study procedures /Page 12	Addition of concomitant medication to list of assessments to be performed pre-study	Omitted from protocol Version 2
8.2.1 Clinical Monitoring During Infusion/ Page 12	Addition of concomitant medication to list of assessments to be performed during infusion.	Omitted from protocol Version 2
8.2.2 Laboratory Measurements/ Page 14	Deletion of 'must be commented on, on the laboratory report'.<br>Addition of 'must be commented on. All comments will be recorded in the CRF using the following abbreviations:<br>NP: Normal for patient<br>NCS: Not clinically significant<br>CS: Clinically significant.	To clarify study procedures
8.2.3 Laboratory Sampling in Children/ Page 15	Deletion of '(except HIV testing will not be performed in children).'<br>Addition of ''(except HIV testing will not be performed in patients under 18 years).'	To clarify study procedures
8.3 End of Study Procedures /Page 15	Deletion of End of Study Procedures<br>Addition of End of Phase 1 Procedures	To clarify study procedures
8.3 End of Study Procedures /Page 15	Addition of concomitant medication and adverse events to list of assessments to be performed at end of Phase 1 Procedures	Omitted from protocol Version 2

Protocol Amendment Form

Section/ Page No	Details of Amendment	Justification of Amendment
11.0 Study Schedule – Pharmacokinetics /Page 20	Addition of Patients will be given a Patient Information Leaflet for extra Blood Tests (Pharmacokinetics) (Appendix 7) and will be asked to sign the Pharmacokinetic Consent Form (Appendix 8).	To clarify study procedures
14.0 Withdrawal of Subjects/Page 21	Addition of In the event of a withdrawal, the investigator will aim to perform the End of Phase 1 Procedures (section 8.3) or Study Schedule Phase 2 (section 10.0) as appropriate.	To clarify study procedures
19.1 Consent Procedure for Adults/Page 36<br>19.2 Consent Procedure for Children/Page 37	Deletion of: If the investigator intends to use his own information leaflet, he must ensure that it contains all the information outlined in Appendix 8 (section 1.8.10).	Investigator must only use the Patient Information Leaflet approved by the MREC.
Appendix 12/Page 57	Deletion of Infusion Record<br>Addition of Home Therapy Infusion Diary Card	Continuity of protocol phrases
Appendix 15/Page 76<br>Study Flow Chart	Deletion of complete appendix	No longer required
Appendix 16/page 77<br>Study Procedures	Addition of concomitant medication and adverse event monitoring	To clarify study procedures (copy of Study Procedures. 31-Mar-00 attached)

I certify that this amendment has been reviewed and found to be satisfactory.

Signed Kathryn [illegible] Date 7/4/00

Signed [illegible] Date 7/4/00

Signed [illegible] Date 10/04/00

Protocol Amendment Form

Title of Trial: **A MULTI-CENTRE, OPEN STUDY TO ASSESS THE SAFETY AND EFFICACY OF A 16% IMMUNOGLOBULIN PRODUCT GIVEN VIA THE SUBCUTANEOUS ROUTE IN PRIMARY ANTIBODY DEFICIENT PATIENTS**

Trial Code: **SCIG01** Version number: **AMENDMENT 2 – 25TH July 2001**

Section/ Page No	Details of Amendment	Justification of Amendment
		In order to comply with the Draft CPMP guidelines : 'Note for Guidance on the Clinical Investigation of Human Normal Immunoglobulin for Subcutaneous and Intramuscular use' 29th March 2001. Currently the study will not comply on the following points:
Synopsis/ Paragraph 2/ Page 2 of protocol<br><br>**Study Population/** Section 6.1/ Page 6 of protocol	**Up to a total of 50 adult and paediatric patients with a diagnosis of primary antibody deficiency will be enrolled.**<br><br>**The number has changed to 50 patients from 40 in order to achieve recruitment of at least 12 patients under 12 years of age. This shall allow for drop-outs to reach a minimum of 10 patients under 12 years of age.**	See sections 2.1.2; 2.2.1 (i)
**Laboratory Measurements/** Section 8.2.2/ Page 13 of protocol	**IgG levels of patients before starting any IgG treatment shall now be collected from patient notes where possible, to quantify 'hypo- or agammaglobulinemia'**	See section 2.1.2 (paragraph 1)

I certify that this amendment has been reviewed and found to be satisfactory.

Signed: C Dash Date: 25/07/01

Name: C. H. DASH Position: MEDICAL DIRECTOR

Protocol Amendment Notification

Title of Trial: **A MULTI-CENTRE, OPEN STUDY TO ASSESS THE SAFETY AND EFFICACY OF A 16% IMMUNOGLOBULIN PRODUCT GIVEN VIA THE SUBCUTANEOUS ROUTE IN PRIMARY ANTIBODY DEFICIENT PATIENTS**

Trial Code: **SCIG01** Version number: **AMENDMENT 2 – 25TH July 2001**

Investigator: **Dr H Gooi** Centre: **St James's University Hospital, Leeds**

Date issued: **1st August 2001**

**Section Page No**	**Details of Amendment**	**Justification for amendment**
		In order to comply with the Draft CPMP guidelines : 'Note for Guidance on the Clinical Investigation of Human Normal Immunoglobulin for Subcutaneous and Intramuscular use' 29th March 2001. Currently the study will not comply on the following points:
**Synopsis**/ Paragraph 2/ Page 2 of protocol<br><br>**Study Population**/ Section 6.1/ Page 6 of protocol	**Up to a total of 50 adult and paediatric patients with a diagnosis of primary antibody deficiency will be enrolled.**<br><br>**The number has changed to 50 patients from 40 in order to achieve recruitment of at least 12 patients under 12 years of age. This shall allow for drop-outs to reach a minimum of 10 patients under 12 years of age.**	See sections 2.1.2; 2.2.1 (i)
**Laboratory Measurements**/ Section 8.2.2/ Page 13 of protocol	**IgG levels of patients before starting any IgG treatment shall now be collected from patient notes where possible, to quantify 'hypo- or agammaglobulinemia'**	See section 2.1.2 (paragraph 1)

I certify that I have read this amendment and that I have and have duly informed the staff involved in the trial of the change/s outlined.

Signed: [signature] Date: 01/08/01

Name: H. C. Gooi Position: PRINCIPAL INVESTIGATOR

Address: ST. JAMES'S UNIVERSITY HOSPITAL LEEDS

Protocol Amendment Form

Title of Trial: **A MULTI-CENTRE, OPEN STUDY TO ASSESS THE SAFETY AND EFFICACY OF A 16% IMMUNOGLOBULIN PRODUCT GIVEN VIA THE SUBCUTANEOUS ROUTE IN PRIMARY ANTIBODY DEFICIENT PATIENTS**

Trial Code: **SCIG01** Version number: **AMENDMENT 2 – 25TH July 2001**

Section/ Page No	Details of Amendment	Justification of Amendment
		In order to comply with the Draft CPMP guidelines : 'Note for Guidance on the Clinical Investigation of Human Normal Immunoglobulin for Subcutaneous and Intramuscular use' 29th March 2001. Currently the study will not comply on the following points:
**Synopsis**/ Paragraph 2/ Page 2 of protocol<br><br>**Study Population**/ Section 6.1/ Page 6 of protocol	**Up to a total of 50 adult and paediatric patients with a diagnosis of primary antibody deficiency will be enrolled.**<br><br>**The number has changed to 50 patients from 40 in order to achieve recruitment of at least 12 patients under 12 years of age. This shall allow for drop-outs to reach a minimum of 10 patients under 12 years of age.**	See sections 2.1.2; 2.2.1 (i)
**Laboratory Measurements**/ Section 8.2.2/ Page 13 of protocol	**IgG levels of patients before starting any IgG treatment shall now be collected from patient notes where possible, to quantify 'hypo- or agammaglobulinemia'**	See section 2.1.2 (paragraph 1)

I certify that this amendment has been reviewed and found to be satisfactory.

Signed Kate Gillanders Date 26/07/01

Name Clinical Research Manager Position CRM

Protocol Amendment Notification

Title of Trial: **A MULTI-CENTRE, OPEN STUDY TO ASSESS THE SAFETY AND EFFICACY OF A 16% IMMUNOGLOBULIN PRODUCT GIVEN VIA THE SUBCUTANEOUS ROUTE IN PRIMARY ANTIBODY DEFICIENT PATIENTS**

Trial Code: **SCIG01**

Version number: **AMENDMENT 2 – 25TH July 2001**

Investigator: **Dr ~~H Gooi~~** A S BANSAL

Centre: **~~St James's University Hospital, Leeds~~** St Helier Hospital, Carshalton, Surrey.

Date issued: **1st August 2001**

Section Page No	Details of Amendment	Justification for amendment
		In order to comply with the Draft CPMP guidelines : 'Note for Guidance on the Clinical Investigation of Human Normal Immunoglobulin for Subcutaneous and Intramuscular use' 29th March 2001. Currently the study will not comply on the following points:
**Synopsis**/ Paragraph 2/ Page 2 of protocol<br><br>**Study Population**/ Section 6.1/ Page 6 of protocol	**Up to a total of 50 adult and paediatric patients with a diagnosis of primary antibody deficiency will be enrolled.**<br><br>**The number has changed to 50 patients from 40 in order to achieve recruitment of at least 12 patients under 12 years of age. This shall allow for drop-outs to reach a minimum of 10 patients under 12 years of age.**	See sections 2.1.2; 2.2.1 (i)
**Laboratory Measurements**/ Section 8.2.2/ Page 13 of protocol	**IgG levels of patients before starting any IgG treatment shall now be collected from patient notes where possible, to quantify 'hypo- or agammaglobulinemia'**	See section 2.1.2 (paragraph 1)

I certify that I have read this amendment and that I have and have duly informed the staff involved in the trial of the change/s outlined.

Signed: [signature] Date: 19/10/01

Name: Dr A S BANSAL Position: Consultant / HOD Immunology

Address: Dept of Immunology, St Helier Hospital, Carshalton, Surrey

Protocol Amendment Notification

Title of Trial: **A MULTI-CENTRE, OPEN STUDY TO ASSESS THE SAFETY AND EFFICACY OF A 16% IMMUNOGLOBULIN PRODUCT GIVEN VIA THE SUBCUTANEOUS ROUTE IN PRIMARY ANTIBODY DEFICIENT PATIENTS**

Trial Code: **SCIG01**
Version number: **AMENDMENT 2 – 25TH July 2001**

Investigator: **Dr H Chapel**
Centre: **John Radcliffe Hospital, Oxford**

Date issued: **1st August 2001**

Section Page No	Details of Amendment	Justification for amendment
		In order to comply with the Draft CPMP guidelines : 'Note for Guidance on the Clinical Investigation of Human Normal Immunoglobulin for Subcutaneous and Intramuscular use' 29th March 2001. Currently the study will not comply on the following points:
**Synopsis**/ Paragraph 2/ Page 2 of protocol<br>**Study Population/** Section 6.1/ Page 6 of protocol	**Up to a total of 50 adult and paediatric patients with a diagnosis of primary antibody deficiency will be enrolled.**<br>**The number has changed to 50 patients from 40 in order to achieve recruitment of at least 12 patients under 12 years of age. This shall allow for drop-outs to reach a minimum of 10 patients under 12 years of age.**	See sections 2.1.2; 2.2.1 (i)
**Laboratory Measurements/** Section 8.2.2/ Page 13 of protocol	**IgG levels of patients before starting any IgG treatment shall now be collected from patient notes where possible, to quantify 'hypo- or agammaglobulinemia'**	See section 2.1.2 (paragraph 1)

I certify that I have read this amendment and that I have and have duly informed the staff involved in the trial of the change/s outlined.

Signed: [signature] Date: 17/1/02

Name: HELEN CHAPEL Position: CONSULTANT IMMUNOLOGIST

Address: JOHN RADCLIFFE HOSPITAL

Protocol Amendment Notification

Title of Trial: **A MULTI-CENTRE, OPEN STUDY TO ASSESS THE SAFETY AND EFFICACY OF A 16% IMMUNOGLOBULIN PRODUCT GIVEN VIA THE SUBCUTANEOUS ROUTE IN PRIMARY ANTIBODY DEFICIENT PATIENTS**

Trial Code: **SCIG01** Version number: **AMENDMENT 2 – 25TH July 2001**

Investigator: **Dr Darbyshire** Centre: **Birmingham Children's Hospital**

Date issued: **1st August 2001**

Section Page No	Details of Amendment	Justification for amendment
		In order to comply with the Draft CPMP guidelines : 'Note for Guidance on the Clinical Investigation of Human Normal Immunoglobulin for Subcutaneous and Intramuscular use' 29th March 2001. Currently the study will not comply on the following points:
**Synopsis**/ Paragraph 2/ Page 2 of protocol<br><br>**Study Population**/ Section 6.1/ Page 6 of protocol	**Up to a total of 50 adult and paediatric patients with a diagnosis of primary antibody deficiency will be enrolled.**<br><br>**The number has changed to 50 patients from 40 in order to achieve recruitment of at least 12 patients under 12 years of age. This shall allow for drop-outs to reach a minimum of 10 patients under 12 years of age.**	See sections 2.1.2; 2.2.1 (i)
**Laboratory Measurements**/ Section 8.2.2/ Page 13 of protocol	**IgG levels of patients before starting any IgG treatment shall now be collected from patient notes where possible, to quantify 'hypo- or agammaglobulinemia'**	See section 2.1.2 (paragraph 1)

I certify that I have read this amendment and that I have and have duly informed the staff involved in the trial of the change/s outlined.

Signed: .... Date: ....

Name: DARBYSHIRE Position: ....

Address: ....

Protocol Amendment Notification

Title of Trial: **A MULTI-CENTRE, OPEN STUDY TO ASSESS THE SAFETY AND EFFICACY OF A 16% IMMUNOGLOBULIN PRODUCT GIVEN VIA THE SUBCUTANEOUS ROUTE IN PRIMARY ANTIBODY DEFICIENT PATIENTS**

Trial Code: **SCIG01** | Version number: **AMENDMENT 2 – 25TH July 2001**

Investigator: **Dr Egner** | Centre: **Northern General, Sheffield**

Date issued: **1st August 2001**

Section Page No	Details of Amendment	Justification for amendment
		In order to comply with the Draft CPMP guidelines : 'Note for Guidance on the Clinical Investigation of Human Normal Immunoglobulin for Subcutaneous and Intramuscular use' 29th March 2001. Currently the study will not comply on the following points:
**Synopsis**/ Paragraph 2/ Page 2 of protocol<br><br>**Study Population**/ Section 6.1/ Page 6 of protocol	**Up to a total of 50 adult and paediatric patients with a diagnosis of primary antibody deficiency will be enrolled.**<br><br>**The number has changed to 50 patients from 40 in order to achieve recruitment of at least 12 patients under 12 years of age. This shall allow for drop-outs to reach a minimum of 10 patients under 12 years of age.**	See sections 2.1.2; 2.2.1 (i)
**Laboratory Measurements**/ Section 8.2.2/ Page 13 of protocol	**IgG levels of patients before starting any IgG treatment shall now be collected from patient notes where possible, to quantify 'hypo- or agammaglobulinemia'**	See section 2.1.2 (paragraph 1)

I certify that I have read this amendment and that I have and have duly informed the staff involved in the trial of the change/s outlined.

Signed: [illegible] Date: [illegible]

Name: William Egner Position: [illegible]

Address: Dept Immunology, NGH Sheffield

Protocol Amendment Notification

Title of Trial: **A MULTI-CENTRE, OPEN STUDY TO ASSESS THE SAFETY AND EFFICACY OF A 16% IMMUNOGLOBULIN PRODUCT GIVEN VIA THE SUBCUTANEOUS ROUTE IN PRIMARY ANTIBODY DEFICIENT PATIENTS**

Trial Code: **SCIG01** Version number: **AMENDMENT 2 – 25TH July 2001**

Investigator: **Dr A Jones** Centre: **Great Ormond Street Hospital, London**

Date issued: **1st August 2001**

**Section Page No**	**Details of Amendment**	**Justification for amendment**
		In order to comply with the Draft CPMP guidelines : 'Note for Guidance on the Clinical Investigation of Human Normal Immunoglobulin for Subcutaneous and Intramuscular use' 29th March 2001. Currently the study will not comply on the following points:
**Synopsis**/ Paragraph 2/ Page 2 of protocol<br>**Study Population**/ Section 6.1/ Page 6 of protocol	**Up to a total of 50 adult and paediatric patients with a diagnosis of primary antibody deficiency will be enrolled.**<br>**The number has changed to 50 patients from 40 in order to achieve recruitment of at least 12 patients under 12 years of age. This shall allow for drop-outs to reach a minimum of 10 patients under 12 years of age.**	See sections 2.1.2; 2.2.1 (i)
**Laboratory Measurements**/ Section 8.2.2/ Page 13 of protocol	**IgG levels of patients before starting any IgG treatment shall now be collected from patient notes where possible, to quantify 'hypo- or agammaglobulinemia'**	See section 2.1.2 (paragraph 1)

I certify that I have read this amendment and that I have and have duly informed the staff involved in the trial of the change/s outlined.

Signed: Alison Jones Date: 3/12/01

Name: ALISON JONES Position: Consultant Immunologist

Address: Immunology Department, Great Ormond Street Hospital, London WC1N 3JH.

Protocol Amendment Notification

Title of Trial: **A MULTI-CENTRE, OPEN STUDY TO ASSESS THE SAFETY AND EFFICACY OF A 16% IMMUNOGLOBULIN PRODUCT GIVEN VIA THE SUBCUTANEOUS ROUTE IN PRIMARY ANTIBODY DEFICIENT PATIENTS**

Trial Code: **SCIG01**

Version number: **AMENDMENT 2 – 25TH July 2001**

Investigator: **Dr H Gooi**

Centre: **St James's University Hospital, Leeds**

Date issued: **1st August 2001**

Section Page No	Details of Amendment	Justification for amendment
		In order to comply with the Draft CPMP guidelines : 'Note for Guidance on the Clinical Investigation of Human Normal Immunoglobulin for Subcutaneous and Intramuscular use' 29th March 2001. Currently the study will not comply on the following points:
**Synopsis**/ Paragraph 2/ Page 2 of protocol<br><br>**Study Population**/ Section 6.1/ Page 6 of protocol	**Up to a total of 50 adult and paediatric patients with a diagnosis of primary antibody deficiency will be enrolled.**<br><br>**The number has changed to 50 patients from 40 in order to achieve recruitment of at least 12 patients under 12 years of age. This shall allow for drop-outs to reach a minimum of 10 patients under 12 years of age.**	See sections 2.1.2; 2.2.1 (i)
**Laboratory Measurements**/ Section 8.2.2/ Page 13 of protocol	**IgG levels of patients before starting any IgG treatment shall now be collected from patient notes where possible, to quantify 'hypo- or agammaglobulinemia'**	See section 2.1.2 (paragraph 1)

I certify that I have read this amendment and that I have and have duly informed the staff involved in the trial of the change/s outlined.

Signed: AR Exley

Date: 31-10-01

Name: Dr Andrew Exley

Position: Co-Investigator Consultant Immunologist.

Address: Papworth Hospital Cambridge CB3 8RE

Protocol Amendment Notification

Title of Trial: **A MULTI-CENTRE, OPEN STUDY TO ASSESS THE SAFETY AND EFFICACY OF A 16% IMMUNOGLOBULIN PRODUCT GIVEN VIA THE SUBCUTANEOUS ROUTE IN PRIMARY ANTIBODY DEFICIENT PATIENTS**

Trial Code: **SCIG01**

Version number: **AMENDMENT 2 – 25TH July 2001**

Investigator: **Dr H Gooi**

Centre: **St James's University Hospital, Leeds**

Date issued: **1st August 2001**

Section Page No	Details of Amendment	Justification for amendment
		In order to comply with the Draft CPMP guidelines : 'Note for Guidance on the Clinical Investigation of Human Normal Immunoglobulin for Subcutaneous and Intramuscular use' 29th March 2001. Currently the study will not comply on the following points:
**Synopsis**/ Paragraph 2/ Page 2 of protocol<br><br>**Study Population**/ Section 6.1/ Page 6 of protocol	**Up to a total of 50 adult and paediatric patients with a diagnosis of primary antibody deficiency will be enrolled.**<br><br>**The number has changed to 50 patients from 40 in order to achieve recruitment of at least 12 patients under 12 years of age. This shall allow for drop-outs to reach a minimum of 10 patients under 12 years of age.**	See sections 2.1.2; 2.2.1 (i)
**Laboratory Measurements**/ Section 8.2.2/ Page 13 of protocol	**IgG levels of patients before starting any IgG treatment shall now be collected from patient notes where possible, to quantify 'hypo- or agammaglobulinemia'**	See section 2.1.2 (paragraph 1)

I certify that I have read this amendment and that I have and have duly informed the staff involved in the trial of the change/s outlined.

Signed: [signature] Date: 12/11/01

Name: H. C. Gooi Position: CONS IMMUNOLOGIST

Address: LEEDS

Protocol Amendment Notification

Title of Trial: **A MULTI-CENTRE, OPEN STUDY TO ASSESS THE SAFETY AND EFFICACY OF A 16% IMMUNOGLOBULIN PRODUCT GIVEN VIA THE SUBCUTANEOUS ROUTE IN PRIMARY ANTIBODY DEFICIENT PATIENTS**

Trial Code: **SCIG01** Version number: **AMENDMENT 2 – 25TH July 2001**

Investigator: **Dr Heaney** Centre: **Hope Hospital, Salford**

Date issued: **1st August 2001**

Section Page No	Details of Amendment	Justification for amendment
		In order to comply with the Draft CPMP guidelines : 'Note for Guidance on the Clinical Investigation of Human Normal Immunoglobulin for Subcutaneous and Intramuscular use' 29th March 2001. Currently the study will not comply on the following points:
**Synopsis**/ Paragraph 2/ Page 2 of protocol<br><br>**Study Population**/ Section 6.1/ Page 6 of protocol	**Up to a total of 50 adult and paediatric patients with a diagnosis of primary antibody deficiency will be enrolled.**<br><br>**The number has changed to 50 patients from 40 in order to achieve recruitment of at least 12 patients under 12 years of age. This shall allow for drop-outs to reach a minimum of 10 patients under 12 years of age.**	See sections 2.1.2; 2.2.1 (i)
**Laboratory Measurements**/ Section 8.2.2/ Page 13 of protocol	**IgG levels of patients before starting any IgG treatment shall now be collected from patient notes where possible, to quantify 'hypo- or agammaglobulinemia'**	See section 2.1.2 (paragraph 1)

I certify that I have read this amendment and that I have and have duly informed the staff involved in the trial of the change/s outlined.

Signed: [signature] Date: 7 Dec 2001

Name: M R HAENEY Position: Consultant Immunologist

Address: Hope Hospital, Salford M6 8HD

Protocol Amendment Notification

Title of Trial: **A MULTI-CENTRE, OPEN STUDY TO ASSESS THE SAFETY AND EFFICACY OF A 16% IMMUNOGLOBULIN PRODUCT GIVEN VIA THE SUBCUTANEOUS ROUTE IN PRIMARY ANTIBODY DEFICIENT PATIENTS**

Trial Code: **SCIG01**

Version number: **AMENDMENT 2 – 25TH July 2001**

Investigator: **Dr H Longhurst**

Centre: **St.Bartholomews and The Royal London**

Date issued: **27th Marh 2003**

Section Page No	Details of Amendment	Justification for amendment
		In order to comply with the Draft CPMP guidelines : 'Note for Guidance on the Clinical Investigation of Human Normal Immunoglobulin for Subcutaneous and Intramuscular use' 29th March 2001. Currently the study will not comply on the following points:
**Synopsis**/ Paragraph 2/ Page 2 of protocol<br><br>**Study Population**/ Section 6.1/ Page 6 of protocol	**Up to a total of 50 adult and paediatric patients with a diagnosis of primary antibody deficiency will be enrolled.**<br><br>**The number has changed to 50 patients from 40 in order to achieve recruitment of at least 12 patients under 12 years of age. This shall allow for drop-outs to reach a minimum of 10 patients under 12 years of age.**	See sections 2.1.2; 2.2.1 (i)
**Laboratory Measurements/** Section 8.2.2/ Page 13 of protocol	**IgG levels of patients before starting any IgG treatment shall now be collected from patient notes where possible, to quantify 'hypo- or agammaglobulinemia'**	See section 2.1.2 (paragraph 1)

I certify that I have read this amendment and that I have and have duly informed the staff involved in the trial of the change/s outlined.

Signed: [signature] Date: 28/3/03

Name: HILARY LONGHURST Position: CONSULTANT IMMUNOLOGIST

Address: DEPT IMMUNOLOGY, ST BARTHOLOMEW'S HOSPITAL, LONDON EC1A 7BE

Protocol Amendment Form

Title of Trial: **A MULTI-CENTRE, OPEN STUDY TO ASSESS THE SAFETY AND EFFICACY OF A 16% IMMUNOGLOBULIN PRODUCT GIVEN VIA THE SUBCUTANEOUS ROUTE IN PRIMARY ANTIBODY DEFICIENT PATIENTS**

Trial Code: **SCIG01** Version number: **AMENDMENT 2 – 25TH July 2001**

Section/ Page No	Details of Amendment	Justification of Amendment
		In order to comply with the Draft CPMP guidelines : 'Note for Guidance on the Clinical Investigation of Human Normal Immunoglobulin for Subcutaneous and Intramuscular use' 29th March 2001. Currently the study will not comply on the following points:
**Synopsis**/ Paragraph 2/ Page 2 of protocol<br>**Study Population**/ Section 6.1/ Page 6 of protocol	**Up to a total of 50 adult and paediatric patients with a diagnosis of primary antibody deficiency will be enrolled.**<br>**The number has changed to 50 patients from 40 in order to achieve recruitment of at least 12 patients under 12 years of age. This shall allow for drop-outs to reach a minimum of 10 patients under 12 years of age.**	See sections 2.1.2; 2.2.1 (i)
**Laboratory Measurements**/ Section 8.2.2/ Page 13 of protocol	**IgG levels of patients before starting any IgG treatment shall now be collected from patient notes where possible, to quantify 'hypo- or agammaglobulinemia'**	See section 2.1.2 (paragraph 1)

I certify that this amendment has been reviewed and found to be satisfactory.

Signed...[signature]... Date...16/10/03

Name...C. TSAKONA... Position...Investigator...

Protocol Amendment Notification

Title of Trial: **A MULTI-CENTRE, OPEN STUDY TO ASSESS THE SAFETY AND EFFICACY OF A 16% IMMUNOGLOBULIN PRODUCT GIVEN VIA THE SUBCUTANEOUS ROUTE IN PRIMARY ANTIBODY DEFICIENT PATIENTS**

Trial Code: **SCIG01** Version number: **AMENDMENT 2 – 25TH July 2001**

Investigator: **Dr Vijayadurai** Centre: **Royal Preston Hospital**

Date issued: **1st August 2001**

Section Page No	Details of Amendment	Justification for amendment
		In order to comply with the Draft CPMP guidelines : 'Note for Guidance on the Clinical Investigation of Human Normal Immunoglobulin for Subcutaneous and Intramuscular use' 29th March 2001. Currently the study will not comply on the following points:
**Synopsis**/ Paragraph 2/ Page 2 of protocol<br><br>**Study Population**/ Section 6.1/ Page 6 of protocol	**Up to a total of 50 adult and paediatric patients with a diagnosis of primary antibody deficiency will be enrolled.**<br><br>**The number has changed to 50 patients from 40 in order to achieve recruitment of at least 12 patients under 12 years of age. This shall allow for drop-outs to reach a minimum of 10 patients under 12 years of age.**	See sections 2.1.2; 2.2.1 (i)
**Laboratory Measurements**/ Section 8.2.2/ Page 13 of protocol	**IgG levels of patients before starting any IgG treatment shall now be collected from patient notes where possible, to quantify 'hypo- or agammaglobulinemia'**	See section 2.1.2 (paragraph 1)

I certify that I have read this amendment and that I have and have duly informed the staff involved in the trial of the change/s outlined.

Signed: P. Vijayan Date: 17/11/2001

Name: P. VIJAYADURAI. Position: CONSULTANT

Address: ROYAL PRESTON HOSPITAL.

Protocol Amendment Notification

Title of Trial: **A MULTI-CENTRE, OPEN STUDY TO ASSESS THE SAFETY AND EFFICACY OF A 16% IMMUNOGLOBULIN PRODUCT GIVEN VIA THE SUBCUTANEOUS ROUTE IN PRIMARY ANTIBODY DEFICIENT PATIENTS**

Trial Code: **SCIG01**

Version number: **AMENDMENT 2 – 25TH July 2001**

Investigator: **Dr H Gooi**

Centre: **St James's University Hospital, Leeds**

Date issued: **1st August 2001**

Section Page No	Details of Amendment	Justification for amendment
		In order to comply with the Draft CPMP guidelines : 'Note for Guidance on the Clinical Investigation of Human Normal Immunoglobulin for Subcutaneous and Intramuscular use' 29th March 2001. Currently the study will not comply on the following points:
**Synopsis**/ Paragraph 2/ Page 2 of protocol<br><br>**Study Population**/ Section 6.1/ Page 6 of protocol	**Up to a total of 50 adult and paediatric patients with a diagnosis of primary antibody deficiency will be enrolled.**<br><br>**The number has changed to 50 patients from 40 in order to achieve recruitment of at least 12 patients under 12 years of age. This shall allow for drop-outs to reach a minimum of 10 patients under 12 years of age.**	See sections 2.1.2; 2.2.1 (i)
**Laboratory Measurements**/ Section 8.2.2/ Page 13 of protocol	**IgG levels of patients before starting any IgG treatment shall now be collected from patient notes where possible, to quantify 'hypo- or agammaglobulinemia'**	See section 2.1.2 (paragraph 1)

I certify that I have read this amendment and that I have and have duly informed the staff involved in the trial of the change/s outlined.

Signed: [signature] Date: 29/10/01

Name: DR P. C. WILLIAMS Position: Consultant Clinical Immunologist

Address: DEPT OF MED BIOCHEM & IMMUNOLOGY, UNIVERSITY HOSPITAL OF WALES, CARDIFF

Protocol Amendment Form

Title of Trial A MULTI-CENTRE, OPEN STUDY TO ASSESS THE SAFETY AND EFFICACY OF A 16% IMMUNOGLOBULIN PRODUCT (SUBGAM®) GIVEN VIA THE SUBCUTANEOUS ROUTE IN PRIMARY ANTIBODY DEFICIENT PATIENTS

Trial Code SCIG01

Version number amended A MULTI-CENTRE, OPEN STUDY TO ASSESS THE SAFETY AND EFFICACY OF A 16% IMMUNOGLOBULIN PRODUCT GIVEN VIA THE SUBCUTANEOUS ROUTE IN PRIMARY ANTIBODY DEFICIENT PATIENTS (Final Protocol version 3 April 2000)

Protocol Amendment Number 3

Section	Details of Amendment	Justification of Amendment
Laboratory Measurements<br>Throughout Protocol	Addition<br>IgG levels of patients during Phase II shall now be collected from patient notes, where possible. When a blood sample for virology is taken at the end of the trial or when the patient withdraws, a little extra blood (no more than 5mls) will be collected for analysis of plasma IgG levels.	IgG levels were monitored throughout Phase I of the trial, but were not initially considered necessary for Phase II, which was intended to provide safety follow up data until Subgam is marketed in the UK. On reflection, we now consider that IgG data during Phase II would be useful to affirm that the doses of Subgam administered to the patients continue to maintain plasma IgG at acceptable levels after long term (1-3.5 years) use of Subgam.<br>IgG levels are generally monitored on a regular basis by the study physicians, as part of the patients standard care, and as such are recorded in the patient notes. We would like to collect this data, where available, for inclusion in our analysis. We would also like to collect a little extra blood at the end of the study or when the patient withdraws from the study for analysis of plasma IgG levels to provide a final IgG level. This extra blood will be collected when a virology and archive sample is being taken.

<u>10.0 Study Schedule</u><br>Phase II (Page 19)	<u>The following:</u><br>If the patient decides to withdraw from Phase II before the product is marketed, vital signs will be recorded and a sample for virology and an archive sample will be taken.<br><u>Should be replaced by:</u><br>If the patient decides to withdraw from Phase II or the trial ends, vital signs will be recorded. A sample for virology, and an archive sample will be taken, together with a little extra blood (no more than 5mls) for analysis of IgG levels.
<u>Appendix 15 Study Procedures</u><br>Flowchart (Page 74)	The study procedures flow chart should now indicate that extra blood will be collected at the end of Phase II for measurement of IgG levels.
<u>Consent forms</u><br>Adult and Child	New consent form (CF incorporating amendment 3_29-Jan-2004) asking patients to consent to the collection of IgG data from their patient notes where possible, as well as having a little extra blood taken (no more than 5mls) when they provide a virology and archive sample at the end of the study.<br>To this, will be attached the original consent form (version3.april2000) should they wish to read it
<u>Patient information leaflets</u><br>Adult, Child and parent/guardian	New patient information leaflet (PIL incorporating amendment 3_29-Jan-2004) informing patients about the collection of IgG data from their patient notes where possible, as well as having a little extra blood taken (no more than 5mls) when they provide a virology and archive sample at the end of the study.<br>To this, will be attached the original patient information leaflet (version3.april2000) should they wish to read it
<u>CRF</u>	An additional CRF page will be inserted for collection of IgG data. (Title: Phase II IgG blood results)

List of documents to be approved

Approval (please initial)

Protocol Amendment 3 – 29th January 2004	CJ	NJ	[illegible]
Consent form (Child) – CF incorporating amendment 3_29-Jan-2004	CJ	NJ	KG
Consent form (adult) – CF incorporating amendment 3_29-Jan-2004	CJ	NJ	KG
Patient information leaflet (adult) - PIL incorporating amendment 3_29-Jan-2004	CJ	NJ	KG
Patient information leaflet (child) - PIL incorporating amendment 3_29-Jan-2004	CJ	NJ	KG
Patient information leaflet (parent/guardian) - PIL incorporating amendment 3_29-Jan-2004	CJ	NJ	KG

I certify that the documents listed above have been reviewed and found to be satisfactory.

Signed...[signature]... Date 2 February 2004

Name C. DASH Position MEDICAL DIRECTOR

Signed...[signature]... Date 2 February 04

Name Nicholas Johnson Position Clinical Project Leader

Signed K. Gillander Date 2 February 2004

Name K. GILLANDERS Position CLINICAL RESEARCH MANAGER

PATIENT INFORMATION LEAFLET - ACCESS TO EXTRA BLOOD RESULTS AND TO TAKE A LITTLE EXTRA BLOOD AT THE END OF PHASE II.

The purpose of this leaflet is to tell you about an extra part to the research study in which you are already taking part. If there is anything which you do not understand or if you require more information, please ask your doctor or nurse. Read these notes carefully, and keep them in a safe place so that you may look at them again. Please note that this extra part to the research study has been reviewed and approved by the appropriate ethics committee(s). The original patient information leaflet (version3.april2000) that you received at the beginning of the study is attached to this leaflet, should you wish to read it again.

Why are the extra blood results being collected for Phase 2 of the trial?

The reason that we would like to collect extra blood test results and to take a little extra blood is so that we can measure the level of antibodies in your blood after you have had a subcutaneous infusion of Subgam®. This will help us to understand more about how Subgam® can help to treat your condition. It will also allow us to check that Subgam® produces suitable levels of immunoglobulin (antibodies) in your blood.

What would I have to do?

During Phase II of the study, the doctors and nurses at your hospital may have taken blood samples to measure the antibodies in your blood. The results of these tests will have been recorded in your notes. We would like to use this data from your notes in our study. In addition, we would like to take a little extra blood (no more than 5mls; approximately 1 teaspoon full) when you are providing a virology and archive sample at the end of the study (or if you decide to withdraw from the study) to measure the levels of antibodies in your blood.

Are there any risks involved?

Apart from giving a little extra blood when you are already providing a virology and archive sample at the end of the trial you will only be having your routine blood tests, which are part of your standard care. These may cause minor discomfort and slight bruising.

CHILDREN'S INFORMATION LEAFLET - ACCESS TO EXTRA BLOOD RESULTS AND TO TAKE A LITTLE EXTRA BLOOD AT THE END OF PHASE II.

This leaflet tells you about an extra part to the research study in which you are already taking part. If there is anything which you do not understand anything please ask your doctor or nurse. The original children's information leaflet (version3.april2000) that you were given at the beginning of the study is attached to this leaflet, if you want to read it again.

Why are the extra blood results being collected for Phase 2 of the trial?
The reason that we would like to collect extra blood test results and to take a little extra blood is so that we can measure the level of antibodies in your blood after you have had a subcutaneous infusion of Subgam®. The results will help doctors treat patients like you in the future.

What would I have to do?
During Phase II of the study, the doctors and nurses at your hospital may have taken blood samples to measure the antibodies in your blood. The results of these tests will have been recorded in your notes. We would like to use these results from your notes in our study. We would also like to take a little extra blood (about a teaspoon full) when you are giving a virology and archive sample at the end of the study (or if you decide to withdraw from the study) to measure the levels of antibodies in your blood.

Are there any risks involved?
Apart from giving a little extra blood when you are already having blood taken for a virology and archive sample at the end of the trial you will only be having your routine blood tests, which are part of your standard care. Taking blood may hurt a little bit and you may get a small bruise on your arm.

A MULTI-CENTRE, OPEN STUDY TO ASSESS THE SAFETY AND EFFICACY OF A 16% IMMUNOGLOBULIN (SUBGAM®) PRODUCT GIVEN VIA THE SUBCUTANEOUS ROUTE IN PRIMARY ANTIBODY DEFICIENT PATIENTS

PARENT/GUARDIAN INFORMATION LEAFLET - ACCESS TO EXTRA BLOOD RESULTS AND TO TAKE A LITTLE EXTRA BLOOD AT THE END OF PHASE II.

The purpose of this leaflet is to tell you about an extra part to the research study in which your child is already taking part. If there is anything which you do not understand or if you require more information, please ask the doctor or nurse. Read these notes carefully, and keep them in a safe place so that you may look at them again. Please note that this extra part to the research study has been reviewed and approved by the appropriate ethics committee(s). The original parent/guardian information leaflet (version3.april2000) that you received at the beginning of the study is attached to this leaflet, should you wish to read it again.

Why are the extra blood results being collected for Phase 2 of the trial?

The reason that we would like to collect extra blood test results and to take a little extra blood is so that we can measure the level of antibodies in your child's blood after he/she has had a subcutaneous infusion of Subgam®. This will help us to understand more about how Subgam® can help to treat your child's condition. It will also allow us to check that Subgam® produces suitable levels of immunoglobulin (antibodies) in their blood.

What would I have to do?

During Phase II of the study, the doctors and nurses at your child's hospital may have taken blood samples to measure the antibodies in their blood. The results of these tests will have been recorded in their notes. We would like to use this data from your child's notes in our study. In addition, we would like to take a little extra blood (no more than 5mls; approximately 1 teaspoon full) when they are providing a virology, and archive sample at the end of the study (or if he/she decides to withdraw from the study). This is to measure the levels of antibodies in their blood.

Are there any risks involved?

Apart from giving a little extra blood when they are providing a virology, and archive sample at the end of the study your child will only be having routine blood tests, which are part of their standard care. These may cause minor discomfort and slight bruising.

A MULTI-CENTRE, OPEN STUDY TO ASSESS THE SAFETY AND EFFICACY OF A 16% IMMUNOGLOBULIN PRODUCT (SUBGAM ®)GIVEN VIA THE SUBCUTANEOUS ROUTE IN PRIMARY ANTIBODY DEFICIENT PATIENTS

ADULT CONSENT FORM

ACCESS TO EXTRA BLOOD RESULTS AND TO TAKE EXTRA BLOOD AT THE END OF PHASE II

(This part to be completed by the patient) *(circle one)*

1. Have you read the Patient Information Leaflet detailing the additional data that we wish to collect for the study (**PIL incorporating amendment 3_29-Jan-2004**) *(please take a copy of the information leaflet home with you to keep)* YES/NO

2. Have you been provided with a copy the original consent form and patient information leaflet (**version3.april2000**) *(please take a copy of these forms home with you to keep)* YES/NO

3. Have you had an opportunity to discuss the changes to the study and ask any questions? YES/NO

4. Have you had satisfactory answers to all of your questions? YES/NO

5. Have you received enough information about the changes to the study? YES/NO

6. Who has given you an explanation about the changes to the study?

   Dr/Nurse ______________________

7. Do you understand that you are still free to withdraw from the study:
   - At any time?
   - Without having to give a reason?
   - Without affecting your future medical care?

   YES/NO

8. Sections of your medical notes relating to your participation in the study may be inspected by responsible individuals from BPL or from regulatory authorities. All personal details will be treated as strictly confidential. Do you give permission for these individuals to have access to your records? YES/NO

9. Has the doctor discussed the circumstances when compensation may be due? YES/NO

10. Have you had sufficient time to come to your decision? YES/NO

11. Do you still agree to take part in the study ? YES/NO

PATIENT *(Please sign below and date your own signature)*

Signed: ______________________ Date: ______________

Print Name: ______________________

INVESTIGATOR

Signed: ______________________ Date: ______________

Print Name: ______________________

A MULTI-CENTRE, OPEN STUDY TO ASSESS THE SAFETY AND EFFICACY OF A 16% IMMUNOGLOBULIN PRODUCT (SUBGAM ®)GIVEN VIA THE SUBCUTANEOUS ROUTE IN PRIMARY ANTIBODY DEFICIENT PATIENTS

CHILDREN'S CONSENT FORM ACCESS TO EXTRA BLOOD RESULTS AND TO TAKE EXTRA BLOOD AT THE END OF PHASE II

This form should be completed and signed by the patient if they are able to understand the study or if not, by the parent/guardian

(circle one)

1. Have you read the Patient Information Leaflet detailing the additional data that we wish to collect for the study **(PIL incorporating amendment 3_29-Jan-2004)** *(please take a copy of the information leaflet home with you to keep)* YES/NO

2. Have you been provided with a copy the original consent form and patient information leaflet **(version3.april2000)** *(please take a copy of these forms home with you to keep)* YES/NO

3. Have you had enough information about the changes made to the study and had a chance to ask any questions? YES/NO

4. Do you understand all the answers to your questions? YES/NO

5. Who has talked to you about the study? Dr/Nurse ____________

6. Do you understand that you can still withdraw from the study: YES/NO
   - At any time?
   - Without having to say why?
   - Without affecting your care?

7. Some of your medical notes may be looked at by people from BPL or from the Government. They will be kept secret. Will you let these people look at your notes? YES/NO

8. Has the doctor told you when compensation may be due? YES/NO

9. Have you had enough time to make your mind up? YES/NO

10. Do you still want to take part in the study? YES/NO

PARENT/GUARDIAN *(Please answer the questions, sign below and date your own signature)*

Is your child able to understand the nature of the trial and give consent to take part? YES/NO
If YES, will the consent be verbal or written? Verbal/Written/NA

Signed: ____________ Date: ____________

Print Name: ____________ Relationship: ____________

CHILD *(Please sign below and add the date)*

Signed: ____________ Date: ____________

Print Name: ____________

INVESTIGATOR

Signed: ____________ Date: ____________

Print Name: ____________

Protocol Amendment Notification

Title of Trial A MULTI-CENTRE, OPEN STUDY TO ASSESS THE SAFETY AND EFFICACY OF A 16% IMMUNOGLOBULIN PRODUCT (SUBGAM ®) GIVEN VIA THE SUBCUTANEOUS ROUTE IN PRIMARY ANTIBODY DEFICIENT PATIENTS

Trial Code SCIG01 **Version number amended** Version3.April2000

Protocol Amendment Number 3 **EUDRACT No** Not Applicable

Investigator: Dr Bansal Centre: St Helier Hospital

D	D	M	M	M	Y	Y	Y	Y
1	3	M	A	Y	2	0	0	4

Date issued to site:

Section	Details of Amendment	Justification of Amendment
Laboratory Measurements Throughout Protocol	Addition<br>IgG levels of patients during Phase II shall now be collected from patient notes, where possible. An additional sample will be taken at the end of the study.	IgG levels were monitored throughout Phase I of the trial, but were not initially considered necessary for Phase II, which was intended to provide safety follow up data until Subgam is marketed in the UK. On reflection, we now consider that IgG data during Phase II would be useful to affirm that the doses of Subgam administered to the patients continue to maintain plasma IgG at acceptable levels after long term (1-3.5 years) use of Subgam.<br>IgG levels are generally monitored on a regular basis by the study physicians, as part of the patients standard care, and as such are recorded in the patient notes. We would like to collect this data, where available, for inclusion in our analysis. We would also like to collect a sample at the end of the study or when the patient withdraws from the study for analysis of plasma IgG levels to provide a final IgG level.

10.0 Study Schedule<br>Phase II (Page 19)	**The following:**<br>If the patient decides to withdraw from Phase II before the product is marketed, vital signs will be recorded and a sample for virology and an archive sample will be taken<br>**Should be replaced by:**<br>If the patient decides to withdraw from Phase II or the trial ends, vital signs will be recorded and an additional blood sample for virology, immunology and an archive sample will be taken.
Appendix 15 Study Procedures<br>Flowchart (Page 74)	The study procedures flow chart should now indicate that a blood sample will be collected at the end of Phase II for measurement of IgG.
Consent forms<br>Adult and Child	New consent form (CF incorporating amendment 3_29-Jan-2004) asking patients to consent to the collection of IgG data from their patient notes where possible, as well as having an additional blood sample taken at the end of the study.<br>To this, will be attached the original consent form (version3.april2000) should they wish to read it
Patient information leaflets<br>Adult, Child and parent/guardian	New patient information leaflet (PIL incorporating amendment 3_29-Jan-2004) informing patients about the collection of IgG data from their patient notes where possible, as well as having an additional blood sample taken at the end of the study.<br>To this, will be attached the original patient information leaflet (version3.april2000) should they wish to read it
CRF	An additional CRF page will be inserted for collection of IgG data. (Title: Phase II IgG blood results)

List of documents to be read

List of documents to be read	When Read (please initial)
Protocol Amendment 3-29th January 2004	AB 14/5/04
Consent form (Child) – CF incorporating amendment 3_29-Jan-2004	AB 14/5/04
Consent form (adult) – CF incorporating amendment 3_29-Jan-2004	AB 14/5/04
Patient information leaflet (adult) - PIL incorporating amendment 3_29-Jan-2004	AB 14/5/04
Patient information leaflet (child) - PIL incorporating amendment 3_29-Jan-2004	AB 14/5/04
Patient information leaflet (parent/guardian) - PIL incorporating amendment 3_29-Jan-2004	AB 14/5/04

I certify that I have read this amendment and that I have and have duly informed the staff involved in the trial of the change/s outlined.

Signed.......[signature]....... Date.......14/5/04.......

Name....Dr A S BANSAL.......... Position....Consultant (head of Dept)

Protocol Amendment Notification

Title of Trial A MULTI-CENTRE, OPEN STUDY TO ASSESS THE SAFETY AND EFFICACY OF A 16% IMMUNOGLOBULIN PRODUCT (SUBGAM ®) GIVEN VIA THE SUBCUTANEOUS ROUTE IN PRIMARY ANTIBODY DEFICIENT PATIENTS

Trial Code SCIG01

Version number amended Version3.April2000

Protocol Amendment Number 3

EUDRACT No Not Applicable

Investigator Dr Chapel

Centre Oxford Radcliffe Hospital

Date issued to site:

D	D	M	M	M	Y	Y	Y	Y
2	6	A	P	R	2	0	0	4

Section	Details of Amendment	Justification of Amendment
Laboratory Measurements Throughout Protocol	Addition<br>IgG levels of patients during Phase II shall now be collected from patient notes, where possible. An additional sample will be taken at the end of the study.	IgG levels were monitored throughout Phase I of the trial, but were not initially considered necessary for Phase II, which was intended to provide safety follow up data until Subgam is marketed in the UK. On reflection, we now consider that IgG data during Phase II would be useful to affirm that the doses of Subgam administered to the patients continue to maintain plasma IgG at acceptable levels after long term (1-3.5 years) use of Subgam.<br>IgG levels are generally monitored on a regular basis by the study physicians, as part of the patients standard care, and as such are recorded in the patient notes. We would like to collect this data, where available, for inclusion in our analysis. We would also like to collect a sample at the end of the study or when the patient withdraws from the study for analysis of plasma IgG levels to provide a final IgG level.

10.0 Study Schedule<br>Phase II (Page 19)	**The following:**<br>If the patient decides to withdraw from Phase II before the product is marketed, vital signs will be recorded and a sample for virology and an archive sample will be taken<br>**Should be replaced by:**<br>If the patient decides to withdraw from Phase II or the trial ends, vital signs will be recorded and an additional blood sample for virology, immunology and an archive sample will be taken.
Appendix 15 Study Procedures<br>Flowchart (Page 74)	The study procedures flow chart should now indicate that a blood sample will be collected at the end of Phase II for measurement of IgG.
Consent forms<br>Adult and Child	New consent form (CF incorporating amendment 3_29-Jan-2004) asking patients to consent to the collection of IgG data from their patient notes where possible, as well as having an additional blood sample taken at the end of the study.<br>To this, will be attached the original consent form (version3.april2000) should they wish to read it
Patient information leaflets<br>Adult, Child and parent/guardian	New patient information leaflet (PIL incorporating amendment 3_29-Jan-2004) informing patients about the collection of IgG data from their patient notes where possible, as well as having an additional blood sample taken at the end of the study.<br>To this, will be attached the original patient information leaflet (version3.april2000) should they wish to read it
CRF	An additional CRF page will be inserted for collection of IgG data. (Title: Phase II IgG blood results)

List of documents to be read | When Read (please initial)

List of documents to be read	When Read (please initial)
Protocol Amendment 3-29th January 2004	lc
Consent form (Child) – CF incorporating amendment 3_29-Jan-2004	l
Consent form (adult) – CF incorporating amendment 3_29-Jan-2004	lc
Patient information leaflet (adult) - PIL incorporating amendment 3_29-Jan-2004	lc
Patient information leaflet (child) - PIL incorporating amendment 3_29-Jan-2004	lc
Patient information leaflet (parent/guardian) - PIL incorporating amendment 3_29-Jan-2004	lc

I certify that I have read this amendment and that I have and have duly informed the staff involved in the trial of the change/s outlined.

Signed........[illegible]........ Date........26/4/04........

Name........H. CHAPPA........ Position........CONSULTANT........ + PI.

Protocol Amendment Notification

Title of Trial A MULTI-CENTRE, OPEN STUDY TO ASSESS THE SAFETY AND EFFICACY OF A 16% IMMUNOGLOBULIN PRODUCT (SUBGAM ®) GIVEN VIA THE SUBCUTANEOUS ROUTE IN PRIMARY ANTIBODY DEFICIENT PATIENTS

Trial Code SCIG01

Version number amended Version3.April2000

Protocol Amendment Number 3

EUDRACT No Not Applicable

Investigator: Dr Duddridge

Centre Leicester Royal Infirmary

Date issued to site:

D	D	M	M	M	Y	Y	Y	Y
0	1	J	U	N	2	0	0	4

Section	Details of Amendment	Justification of Amendment
Laboratory Measurements Throughout Protocol	Addition<br>IgG levels of patients during Phase II shall now be collected from patient notes, where possible. An additional sample will be taken at the end of the study.	IgG levels were monitored throughout Phase I of the trial, but were not initially considered necessary for Phase II, which was intended to provide safety follow up data until Subgam is marketed in the UK. On reflection, we now consider that IgG data during Phase II would be useful to affirm that the doses of Subgam administered to the patients continue to maintain plasma IgG at acceptable levels after long term (1-3.5 years) use of Subgam.<br>IgG levels are generally monitored on a regular basis by the study physicians, as part of the patients standard care, and as such are recorded in the patient notes. We would like to collect this data, where available, for inclusion in our analysis. We would also like to collect a sample at the end of the study or when the patient withdraws from the study for analysis of plasma IgG levels to provide a final IgG level.

<u>10.0 Study Schedule</u><br>Phase II (Page 19)	**The following:**<br>If the patient decides to withdraw from Phase II before the product is marketed, vital signs will be recorded and a sample for virology and an archive sample will be taken<br>**Should be replaced by:**<br>If the patient decides to withdraw from Phase II or the trial ends, vital signs will be recorded and an additional blood sample for virology, immunology and an archive sample will be taken.
<u>Appendix 15 Study Procedures</u><br>Flowchart (Page 74)	The study procedures flow chart should now indicate that a blood sample will be collected at the end of Phase II for measurement of IgG.
<u>Consent forms</u><br>Adult and Child	New consent form (CF incorporating amendment 3_29-Jan-2004) asking patients to consent to the collection of IgG data from their patient notes where possible, as well as having an additional blood sample taken at the end of the study.<br>To this, will be attached the original consent form (version3.april2000) should they wish to read it
<u>Patient information leaflets</u><br>Adult, Child and parent/guardian	New patient information leaflet (PIL incorporating amendment 3_29-Jan-2004) informing patients about the collection of IgG data from their patient notes where possible, as well as having an additional blood sample taken at the end of the study.<br>To this, will be attached the original patient information leaflet (version3.april2000) should they wish to read it
<u>CRF</u>	An additional CRF page will be inserted for collection of IgG data. (Title: Phase II IgG blood results)

List of documents to be read

When Read (please initial)

Protocol Amendment 3-29th January 2004	C
Consent form (Child) – CF incorporating amendment 3_29-Jan-2004	C
Consent form (adult) – CF incorporating amendment 3_29-Jan-2004	C
Patient information leaflet (adult) - PIL incorporating amendment 3_29-Jan-2004	C
Patient information leaflet (child) - PIL incorporating amendment 3_29-Jan-2004	C
Patient information leaflet (parent/guardian) - PIL incorporating amendment 3_29-Jan-2004	C

I certify that I have read this amendment and that I have and have duly informed the staff involved in the trial of the change/s outlined.

Signed.......[signature]....... Date.......11th June 2004.......

Name.......MICHAEL [illegible]....... Position.......CONSULTANT.......

Protocol Amendment Notification

Title of Trial: **A MULTI-CENTRE, OPEN STUDY TO ASSESS THE SAFETY AND EFFICACY OF A 16% IMMUNOGLOBULIN PRODUCT GIVEN VIA THE SUBCUTANEOUS ROUTE IN PRIMARY ANTIBODY DEFICIENT PATIENTS**

Trial Code: **SCIG01**

Version number: **AMENDMENT 2 – 25TH July 2001**

Investigator: **Dr Duddridge**

Centre: **Leicester Royal Infirmary**

Date issued: **1st August 2001**

Section Page No	Details of Amendment	Justification for amendment
		In order to comply with the Draft CPMP guidelines : 'Note for Guidance on the Clinical Investigation of Human Normal Immunoglobulin for Subcutaneous and Intramuscular use' 29th March 2001. Currently the study will not comply on the following points:
**Synopsis**/ Paragraph 2/ Page 2 of protocol<br><br>**Study Population/** Section 6.1/ Page 6 of protocol	**Up to a total of 50 adult and paediatric patients with a diagnosis of primary antibody deficiency will be enrolled.**<br><br>**The number has changed to 50 patients from 40 in order to achieve recruitment of at least 12 patients under 12 years of age. This shall allow for drop-outs to reach a minimum of 10 patients under 12 years of age.**	See sections 2.1.2; 2.2.1 (i)
**Laboratory Measurements/** Section 8.2.2/ Page 13 of protocol	**IgG levels of patients before starting any IgG treatment shall now be collected from patient notes where possible, to quantify 'hypo- or agammaglobulinemia'**	See section 2.1.2 (paragraph 1)

I certify that I have read this amendment and that I have and have duly informed the staff involved in the trial of the change/s outlined.

Signed: [signature] Date: 17/12/01

Name: DR MICHAEL [illegible] Position: CONSULTANT CLINICAL IMMUNOLOGIST

Address: DEPT IMMUNOLOGY, LEICESTER ROYAL INFIRMARY, LEICESTER, LE1 5WW

Protocol Amendment Notification

Title of Trial A MULTI-CENTRE, OPEN STUDY TO ASSESS THE SAFETY AND EFFICACY OF A 16% IMMUNOGLOBULIN PRODUCT (SUBGAM ®) GIVEN VIA THE SUBCUTANEOUS ROUTE IN PRIMARY ANTIBODY DEFICIENT PATIENTS

Trial Code SCIG01

Version number amended Version3.April2000

Protocol Amendment Number 3

EUDRACT No Not Applicable

Investigator Dr Darbyshire

Centre Birmingham Children's Hospital

Date issued to site:

D	D	M	M	M	Y	Y	Y	Y
1	0	A	P	R	2	0	0	4

Section	Details of Amendment	Justification of Amendment
Laboratory Measurements Throughout Protocol	Addition<br>IgG levels of patients during Phase II shall now be collected from patient notes, where possible. An additional sample will be taken at the end of the study.	IgG levels were monitored throughout Phase I of the trial, but were not initially considered necessary for Phase II, which was intended to provide safety follow up data until Subgam is marketed in the UK. On reflection, we now consider that IgG data during Phase II would be useful to affirm that the doses of Subgam administered to the patients continue to maintain plasma IgG at acceptable levels after long term (1-3.5 years) use of Subgam.<br>IgG levels are generally monitored on a regular basis by the study physicians, as part of the patients standard care, and as such are recorded in the patient notes. We would like to collect this data, where available, for inclusion in our analysis. We would also like to collect a sample at the end of the study or when the patient withdraws from the study for analysis of plasma IgG levels to provide a final IgG level.

10.0 Study Schedule<br>Phase II (Page 19)	**The following:**<br>If the patient decides to withdraw from Phase II before the product is marketed, vital signs will be recorded and a sample for virology and an archive sample will be taken<br>**Should be replaced by:**<br>If the patient decides to withdraw from Phase II or the trial ends, vital signs will be recorded and an additional blood sample for virology, immunology and an archive sample will be taken.
Appendix 15 Study Procedures<br>Flowchart (Page 74)	The study procedures flow chart should now indicate that a blood sample will be collected at the end of Phase II for measurement of IgG.
Consent forms<br>Adult and Child	New consent form (CF incorporating amendment 3_29-Jan-2004) asking patients to consent to the collection of IgG data from their patient notes where possible, as well as having an additional blood sample taken at the end of the study.<br>To this, will be attached the original consent form (version3.april2000) should they wish to read it
Patient information leaflets<br>Adult, Child and parent/guardian	New patient information leaflet (PIL incorporating amendment 3_29-Jan-2004) informing patients about the collection of IgG data from their patient notes where possible, as well as having an additional blood sample taken at the end of the study.<br>To this, will be attached the original patient information leaflet (version3.april2000) should they wish to read it
CRF	An additional CRF page will be inserted for collection of IgG data. (Title: Phase II IgG blood results)

List of documents to be read | When Read (please initial)

Protocol Amendment 3-29th January 2004	14/4/04
Consent form (Child) – CF incorporating amendment 3_29-Jan-2004	14/4/04
Consent form (adult) – CF incorporating amendment 3_29-Jan-2004	14/4/04
Patient information leaflet (adult) - PIL incorporating amendment 3_29-Jan-2004	14/4/04
Patient information leaflet (child) - PIL incorporating amendment 3_29-Jan-2004	14/4/04
Patient information leaflet (parent/guardian) - PIL incorporating amendment 3_29-Jan-2004	14/4/04

I certify that I have read this amendment and that I have and have duly informed the staff involved in the trial of the change/s outlined.

Signed.......[signature]....... Date.......26/6/04.......

Name.......P. J. DARWIN....... Position.......Consultant.......

Protocol Amendment Notification

Title of Trial A MULTI-CENTRE, OPEN STUDY TO ASSESS THE SAFETY AND EFFICACY OF A 16% IMMUNOGLOBULIN PRODUCT (SUBGAM ®) GIVEN VIA THE SUBCUTANEOUS ROUTE IN PRIMARY ANTIBODY DEFICIENT PATIENTS

Trial Code SCIG01

Version number amended Version3.April2000

Protocol Amendment Number 3

EUDRACT No Not Applicable

Investigator Dr Egner

Centre Northern General Hospital, Sheffield

Date issued to site:

D	D	M	M	M	Y	Y	Y	Y
1	9	A	P	R	2	0	0	4

Section	Details of Amendment	Justification of Amendment
Laboratory Measurements Throughout Protocol	Addition<br>IgG levels of patients during Phase II shall now be collected from patient notes, where possible. An additional sample will be taken at the end of the study.	IgG levels were monitored throughout Phase I of the trial, but were not initially considered necessary for Phase II, which was intended to provide safety follow up data until Subgam is marketed in the UK. On reflection, we now consider that IgG data during Phase II would be useful to affirm that the doses of Subgam administered to the patients continue to maintain plasma IgG at acceptable levels after long term (1-3.5 years) use of Subgam.<br>IgG levels are generally monitored on a regular basis by the study physicians, as part of the patients standard care, and as such are recorded in the patient notes. We would like to collect this data, where available, for inclusion in our analysis. We would also like to collect a sample at the end of the study or when the patient withdraws from the study for analysis of plasma IgG levels to provide a final IgG level.

10.0 Study Schedule<br>Phase II (Page 19)	**The following:**<br>If the patient decides to withdraw from Phase II before the product is marketed, vital signs will be recorded and a sample for virology and an archive sample will be taken<br>**Should be replaced by:**<br>If the patient decides to withdraw from Phase II or the trial ends, vital signs will be recorded and an additional blood sample for virology, immunology and an archive sample will be taken.
Appendix 15 Study Procedures<br>Flowchart (Page 74)	The study procedures flow chart should now indicate that a blood sample will be collected at the end of Phase II for measurement of IgG.
Consent forms<br>Adult and Child	New consent form (CF incorporating amendment 3_29-Jan-2004) asking patients to consent to the collection of IgG data from their patient notes where possible, as well as having an additional blood sample taken at the end of the study.<br>To this, will be attached the original consent form (version3.april2000) should they wish to read it
Patient information leaflets<br>Adult, Child and parent/guardian	New patient information leaflet (PIL incorporating amendment 3_29-Jan-2004) informing patients about the collection of IgG data from their patient notes where possible, as well as having an additional blood sample taken at the end of the study.<br>To this, will be attached the original patient information leaflet (version3.april2000) should they wish to read it
CRF	An additional CRF page will be inserted for collection of IgG data. (Title: Phase II IgG blood results)

List of documents to be read

When Read (please initial)

Protocol Amendment 3-29th January 2004	
Consent form (Child) – CF incorporating amendment 3_29-Jan-2004	
Consent form (adult) CF incorporating amendment 3_29-Jan-2004	
Patient information leaflet (adult) - PIL incorporating amendment 3_29-Jan-2004	
Patient information leaflet (child) - PIL incorporating amendment 3_29-Jan-2004	
Patient information leaflet (parent/guardian) - PIL incorporating amendment 3_29-Jan-2004	

I certify that I have read this amendment and that I have and have duly informed the staff involved in the trial of the change/s outlined.

Signed.......[illegible]....... Date....19/4/04....

Name.......[illegible]....... Position....Consultant [illegible]....

Protocol Amendment Notification

ó

Title of Trial A MULTI-CENTRE, OPEN STUDY TO ASSESS THE SAFETY AND EFFICACY OF A 16% IMMUNOGLOBULIN PRODUCT (SUBGAM ®) GIVEN VIA THE SUBCUTANEOUS ROUTE IN PRIMARY ANTIBODY DEFICIENT PATIENTS

Trial Code SCIG01

Version number amended Version3.April2000

Protocol Amendment Number 3

EUDRACT No Not Applicable

Investigator: DR A JONES

Centre: GREAT ORMOND STREET HOSPITAL

Date issued to site:

D	D	M	M	M	Y	Y	Y	Y
1	1	M	A	Y	2	0	0	4

Section	Details of Amendment	Justification of Amendment
Laboratory Measurements Throughout Protocol	Addition<br>IgG levels of patients during Phase II shall now be collected from patient notes, where possible. An additional sample will be taken at the end of the study.	IgG levels were monitored throughout Phase I of the trial, but were not initially considered necessary for Phase II, which was intended to provide safety follow up data until Subgam is marketed in

10.0 Study Schedule<br>Phase II (Page 19)	**The following:**<br>If the patient decides to withdraw from Phase II before the product is marketed, vital signs will be recorded and a sample for virology and an archive sample will be taken<br>**Should be replaced by:**<br>If the patient decides to withdraw from Phase II or the trial ends, vital signs will be recorded and an additional blood sample for virology, immunology and an archive sample will be taken.	the UK. On reflection, we now consider that IgG data during Phase II would be useful to affirm that the doses of Subgam administered to the patients continue to maintain plasma IgG at acceptable levels after long term (1-3.5 years) use of Subgam.<br>IgG levels are generally monitored on a regular basis by the study physicians, as part of the patients standard care, and as such are recorded in the patient notes. We would like to collect this data, where available, for inclusion in our analysis. We would also like to collect a sample at the end of the study or when the patient withdraws from the study for analysis of plasma IgG levels to provide a final IgG level.
Appendix 15 Study Procedures<br>Flowchart (Page 74)	The study procedures flow chart should now indicate that a blood sample will be collected at the end of Phase II for measurement of IgG.	
Consent forms<br>Adult and Child	New consent form (CF incorporating amendment 3_29-Jan-2004) asking patients to consent to the collection of IgG data from their patient notes where possible, as well as having an additional blood sample taken at the end of the study.<br>To this, will be attached the original consent form (version3.april2000) should they wish to read it	
Patient information leaflets<br>Adult, Child and parent/guardian	New patient information leaflet (PIL incorporating amendment 3_29-Jan-2004) informing patients about the collection of IgG data from their patient notes where possible, as well as having an additional blood sample taken at the end of the study.<br>To this, will be attached the original patient information leaflet (version3.april2000) should they wish to read it	
CRF	An additional CRF page will be inserted for collection of IgG data. (Title: Phase II IgG blood results)	

List of documents to be read

ö

	When Read (please initial)
Protocol Amendment 3-29th January 2004	AMJ
Consent form (Child) – CF incorporating amendment 3_29-Jan-2004	AMJ
Consent form (adult) – CF incorporating amendment 3_29-Jan-2004	AMJ
Patient information leaflet (adult) - PIL incorporating amendment 3_29-Jan-2004	AMJ
Patient information leaflet (child) - PIL incorporating amendment 3_29-Jan-2004	AMJ
Patient information leaflet (parent/guardian) - PIL incorporating amendment 3_29-Jan-2004	AMJ

I certify that I have read this amendment and that I have and have duly informed the staff involved in the trial of the change/s outlined.

Signed: Alison Jones   Date: 11/5/04

Name: ALISON JONES   Position: CONSULTANT IMMUNOLOGIST

RECEIVED
25 JUN 2004

MD020303
Version 4

Protocol Amendment Notification

Title of Trial A MULTI-CENTRE, OPEN STUDY TO ASSESS THE SAFETY AND EFFICACY OF A 16% IMMUNOGLOBULIN PRODUCT (SUBGAM ®) GIVEN VIA THE SUBCUTANEOUS ROUTE IN PRIMARY ANTIBODY DEFICIENT PATIENTS

Trial Code SCIG01

Version number amended Version3.April2000

Protocol Amendment Number 3

EUDRACT No Not Applicable

Investigator: Dr Exley

Centre: Papworth Hospital

Date issued to site:

D	D	M	M	M	Y	Y	Y	Y
1	3	M	A	Y	2	0	0	4

Section	Details of Amendment	Justification of Amendment
Laboratory Measurements Throughout Protocol	Addition<br>IgG levels of patients during Phase II shall now be collected from patient notes, where possible. An additional sample will be taken at the end of the study.	IgG levels were monitored throughout Phase I of the trial, but were not initially considered necessary for Phase II, which was intended to provide safety follow up data until Subgam is marketed in the UK. On reflection, we now consider that IgG data during Phase II would be useful to affirm that the doses of Subgam administered to the patients continue to maintain plasma IgG at acceptable levels after long term (1-3.5 years) use of Subgam.<br>IgG levels are generally monitored on a regular basis by the study physicians, as part of the patients standard care, and as such are recorded in the patient notes. We would like to collect this data, where available, for inclusion in our analysis. We would also like to collect a sample at the end of the study or when the patient withdraws from the study for analysis of plasma IgG levels to provide a final IgG level.

10.0 Study Schedule<br>Phase II (Page 19)	**The following:**<br>If the patient decides to withdraw from Phase II before the product is marketed, vital signs will be recorded and a sample for virology and an archive sample will be taken<br>**Should be replaced by:**<br>If the patient decides to withdraw from Phase II or the trial ends, vital signs will be recorded and an additional blood sample for virology, immunology and an archive sample will be taken.
Appendix 15 Study Procedures<br>Flowchart (Page 74)	The study procedures flow chart should now indicate that a blood sample will be collected at the end of Phase II for measurement of IgG.
Consent forms<br>Adult and Child	New consent form (CF incorporating amendment 3_29-Jan-2004) asking patients to consent to the collection of IgG data from their patient notes where possible, as well as having an additional blood sample taken at the end of the study.<br>To this, will be attached the original consent form (version3.april2000) should they wish to read it
Patient information leaflets<br>Adult, Child and parent/guardian	New patient information leaflet (PIL incorporating amendment 3_29-Jan-2004) informing patients about the collection of IgG data from their patient notes where possible, as well as having an additional blood sample taken at the end of the study.<br>To this, will be attached the original patient information leaflet (version3.april2000) should they wish to read it
CRF	An additional CRF page will be inserted for collection of IgG data. (Title: Phase II IgG blood results)

List of documents to be read

	When Read (please initial)
Protocol Amendment 3-29th January 2004	AE
Consent form (Child) – CF incorporating amendment 3_29-Jan-2004	AE
Consent form (adult) – CF incorporating amendment 3_29-Jan-2004	AE
Patient information leaflet (adult) - PIL incorporating amendment 3_29-Jan-2004	AE
Patient information leaflet (child) - PIL incorporating amendment 3_29-Jan-2004	AE
Patient information leaflet (parent/guardian) - PIL incorporating amendment 3_29-Jan-2004	AE

I certify that I have read this amendment and that I have and have duly informed the staff involved in the trial of the change/s outlined.

Signed: AR Exley Date: 2/6/4

Name: AR Exley Position: Consultant Immunologist

Protocol Amendment Notification

Title of Trial A MULTI-CENTRE, OPEN STUDY TO ASSESS THE SAFETY AND EFFICACY OF A 16% IMMUNOGLOBULIN PRODUCT (SUBGAM ®) GIVEN VIA THE SUBCUTANEOUS ROUTE IN PRIMARY ANTIBODY DEFICIENT PATIENTS

Trial Code SCIG01 | **Version number amended** Version3.April2000

Protocol Amendment Number 3 | **EUDRACT No** Not Applicable

Investigator Dr Gooi | Centre St James Hospital, Leeds

Date issued to site:

D	D	M	M	M	Y	Y	Y	Y
0	8	A	P	R	2	0	0	4

Section	Details of Amendment	Justification of Amendment
Laboratory Measurements Throughout Protocol	Addition<br>IgG levels of patients during Phase II shall now be collected from patient notes, where possible. An additional sample will be taken at the end of the study.	IgG levels were monitored throughout Phase I of the trial, but were not initially considered necessary for Phase II, which was intended to provide safety follow up data until Subgam is marketed in the UK. On reflection, we now consider that IgG data during Phase II would be useful to affirm that the doses of Subgam administered to the patients continue to maintain plasma IgG at acceptable levels after long term (1-3.5 years) use of Subgam.<br>IgG levels are generally monitored on a regular basis by the study physicians, as part of the patients standard care, and as such are recorded in the patient notes. We would like to collect this data, where available, for inclusion in our analysis. We would also like to collect a sample at the end of the study or when the patient withdraws from the study for analysis of plasma IgG levels to provide a final IgG level.

10.0 Study Schedule<br>Phase II (Page 19)	**The following:**<br>If the patient decides to withdraw from Phase II before the product is marketed, vital signs will be recorded and a sample for virology and an archive sample will be taken<br>**Should be replaced by:**<br>If the patient decides to withdraw from Phase II or the trial ends, vital signs will be recorded and an additional blood sample for virology, immunology and an archive sample will be taken.
Appendix 15 Study Procedures<br>Flowchart (Page 74)	The study procedures flow chart should now indicate that a blood sample will be collected at the end of Phase II for measurement of IgG.
Consent forms<br>Adult and Child	New consent form (CF incorporating amendment 3_29-Jan-2004) asking patients to consent to the collection of IgG data from their patient notes where possible, as well as having an additional blood sample taken at the end of the study.<br>To this, will be attached the original consent form (version3.april2000) should they wish to read it
Patient information leaflets<br>Adult, Child and parent/guardian	New patient information leaflet (PIL incorporating amendment 3_29-Jan-2004) informing patients about the collection of IgG data from their patient notes where possible, as well as having an additional blood sample taken at the end of the study.<br>To this, will be attached the original patient information leaflet (version3.april2000) should they wish to read it
CRF	An additional CRF page will be inserted for collection of IgG data. (Title: Phase II IgG blood results)

List of documents to be read

List of documents to be read	When Read (please initial)
Protocol Amendment 3-29th January 2004	HCG
Consent form (Child) – CF incorporating amendment 3_29-Jan-2004	HCG
Consent form (adult) – CF incorporating amendment 3_29-Jan-2004	HCG
Patient information leaflet (adult) - PIL incorporating amendment 3_29-Jan-2004	HCG
Patient information leaflet (child) - PIL incorporating amendment 3_29-Jan-2004	HCG
Patient information leaflet (parent/guardian) - PIL incorporating amendment 3_29-Jan-2004	HCG

I certify that I have read this amendment and that I have and have duly informed the staff involved in the trial of the change/s outlined.

Signed......[signature]...... Date....08/04/04....

Name....H. C. Gooi.... Position....CONSULTANT CLINICAL IMMUNOLOGIST

Protocol Amendment Notification

Title of Trial A MULTI-CENTRE, OPEN STUDY TO ASSESS THE SAFETY AND EFFICACY OF A 16% IMMUNOGLOBULIN PRODUCT (SUBGAM ®) GIVEN VIA THE SUBCUTANEOUS ROUTE IN PRIMARY ANTIBODY DEFICIENT PATIENTS

Trial Code SCIG01

Version number amended Version3.April2000

Protocol Amendment Number 3

EUDRACT No Not Applicable

Investigator Dr Haeney

Centre Hope Hospital, Salford

Date issued to site:

D	D	M	M	M	Y	Y	Y	Y
1	3	A	P	R	2	0	0	4

**Section**	**Details of Amendment**	**Justification of Amendment**
Laboratory Measurements Throughout Protocol	Addition<br>IgG levels of patients during Phase II shall now be collected from patient notes, where possible. An additional sample will be taken at the end of the study.	IgG levels were monitored throughout Phase I of the trial, but were not initially considered necessary for Phase II, which was intended to provide safety follow up data until Subgam is marketed in the UK. On reflection, we now consider that IgG data during Phase II would be useful to affirm that the doses of Subgam administered to the patients continue to maintain plasma IgG at acceptable levels after long term (1-3.5 years) use of Subgam.<br>IgG levels are generally monitored on a regular basis by the study physicians, as part of the patients standard care, and as such are recorded in the patient notes. We would like to collect this data, where available, for inclusion in our analysis. We would also like to collect a sample at the end of the study or when the patient withdraws from the study for analysis of plasma IgG levels to provide a final IgG level.

10.0 Study Schedule<br>Phase II (Page 19)	**The following:**<br>If the patient decides to withdraw from Phase II before the product is marketed, vital signs will be recorded and a sample for virology and an archive sample will be taken<br>**Should be replaced by:**<br>If the patient decides to withdraw from Phase II or the trial ends, vital signs will be recorded and an additional blood sample for virology, immunology and an archive sample will be taken.
Appendix 15 Study Procedures<br>Flowchart (Page 74)	The study procedures flow chart should now indicate that a blood sample will be collected at the end of Phase II for measurement of IgG.
Consent forms<br>Adult and Child	New consent form (CF incorporating amendment 3_29-Jan-2004) asking patients to consent to the collection of IgG data from their patient notes where possible, as well as having an additional blood sample taken at the end of the study.<br>To this, will be attached the original consent form (version3.april2000) should they wish to read it
Patient information leaflets<br>Adult, Child and parent/guardian	New patient information leaflet (PIL incorporating amendment 3_29-Jan-2004) informing patients about the collection of IgG data from their patient notes where possible, as well as having an additional blood sample taken at the end of the study.<br>To this, will be attached the original patient information leaflet (version3.april2000) should they wish to read it
CRF	An additional CRF page will be inserted for collection of IgG data. (Title: Phase II IgG blood results)

List of documents to be read

	When Read (please initial)
Protocol Amendment 3-29th January 2004	MRH
Consent form (Child) – CF incorporating amendment 3_29-Jan-2004	MRH
Consent form (adult) – CF incorporating amendment 3_29-Jan-2004	MRH
Patient information leaflet (adult) - PIL incorporating amendment 3_29-Jan-2004	MRH
Patient information leaflet (child) - PIL incorporating amendment 3_29-Jan-2004	MRH
Patient information leaflet (parent/guardian) - PIL incorporating amendment 3_29-Jan-2004	MRH

I certify that I have read this amendment and that I have and have duly informed the staff involved in the trial of the change/s outlined.

Signed: MR Harney Date: 13 APRIL 04

Name: MR HARNEY Position: CONS. IMMUNOLOGIST

MD020303
Version 4

Protocol Amendment Notification

Title of Trial A MULTI-CENTRE, OPEN STUDY TO ASSESS THE SAFETY AND EFFICACY OF A 16% IMMUNOGLOBULIN PRODUCT (SUBGAM ®) GIVEN VIA THE SUBCUTANEOUS ROUTE IN PRIMARY ANTIBODY DEFICIENT PATIENTS

Trial Code SCIG01 — **Version number amended** Version3.April2000

Protocol Amendment Number 3 — **EUDRACT No** Not Applicable

Investigator Dr Longhurst — Centre St Bartholomew's Hospital, London

Date issued to site:

D	D	M	M	M	Y	Y	Y	Y
1	4	A	P	R	2	0	0	4

Section	Details of Amendment	Justification of Amendment
Laboratory Measurements Throughout Protocol	Addition<br>IgG levels of patients during Phase II shall now be collected from patient notes, where possible. An additional sample will be taken at the end of the study.	IgG levels were monitored throughout Phase I of the trial, but were not initially considered necessary for Phase II, which was intended to provide safety follow up data until Subgam is marketed in the UK. On reflection, we now consider that IgG data during Phase II would be useful to affirm that the doses of Subgam administered to the patients continue to maintain plasma IgG at acceptable levels after long term (1-3.5 years) use of Subgam.<br>IgG levels are generally monitored on a regular basis by the study physicians, as part of the patients standard care, and as such are recorded in the patient notes. We would like to collect this data, where available, for inclusion in our analysis. We would also like to collect a sample at the end of the study or when the patient withdraws from the study for analysis of plasma IgG levels to provide a final IgG level.

10.0 Study Schedule<br>Phase II (Page 19)	**The following:**<br>If the patient decides to withdraw from Phase II before the product is marketed, vital signs will be recorded and a sample for virology and an archive sample will be taken<br>**Should be replaced by:**<br>If the patient decides to withdraw from Phase II or the trial ends, vital signs will be recorded and an additional blood sample for virology, immunology and an archive sample will be taken.
Appendix 15 Study Procedures<br>Flowchart (Page 74)	The study procedures flow chart should now indicate that a blood sample will be collected at the end of Phase II for measurement of IgG.
Consent forms<br>Adult and Child	New consent form (CF incorporating amendment 3_29-Jan-2004) asking patients to consent to the collection of IgG data from their patient notes where possible, as well as having an additional blood sample taken at the end of the study.<br>To this, will be attached the original consent form (version3.april2000) should they wish to read it
Patient information leaflets<br>Adult, Child and parent/guardian	New patient information leaflet (PIL incorporating amendment 3_29-Jan-2004) informing patients about the collection of IgG data from their patient notes where possible, as well as having an additional blood sample taken at the end of the study.<br>To this, will be attached the original patient information leaflet (version3.april2000) should they wish to read it
CRF	An additional CRF page will be inserted for collection of IgG data. (Title: Phase II IgG blood results)

List of documents to be read

List of documents to be read	When Read (please initial)
Protocol Amendment 3-29th January 2004	ih
Consent form (Child) – CF incorporating amendment 3_29-Jan-2004	ih
Consent form (adult) – CF incorporating amendment 3_29-Jan-2004	ih
Patient information leaflet (adult) - PIL incorporating amendment 3_29-Jan-2004	ih
Patient information leaflet (child) - PIL incorporating amendment 3_29-Jan-2004	ih
Patient information leaflet (parent/guardian) - PIL incorporating amendment 3_29-Jan-2004	ih

I certify that I have read this amendment and that I have and have duly informed the staff involved in the trial of the change/s outlined.

Signed: HJ Longhurst   Date: 16/4/04

Name: H J LONGHURST   Position: CONSULTANT IMMUNOLOGIST / PRINCIPAL INVESTIGATOR

Protocol Amendment Notification

Title of Trial A MULTI-CENTRE, OPEN STUDY TO ASSESS THE SAFETY AND EFFICACY OF A 16% IMMUNOGLOBULIN PRODUCT (SUBGAM ®) GIVEN VIA THE SUBCUTANEOUS ROUTE IN PRIMARY ANTIBODY DEFICIENT PATIENTS

Trial Code SCIG01

Version number amended Version3.April2000

Protocol Amendment Number 3

EUDRACT No Not Applicable

Investigator: Dr Tsakona

Centre The Guest Hospital, Dudley

Date issued to site:

D	D	M	M	M	Y	Y	Y	Y
1	2		1	0	2	0	0	4

* aware of the changes long time ago (early this year) but returning the form now - APOLOGIES!

Section	Details of Amendment	Justification of Amendment
Laboratory Measurements Throughout Protocol	Addition<br>IgG levels of patients during Phase II shall now be collected from patient notes, where possible. An additional sample will be taken at the end of the study.	IgG levels were monitored throughout Phase I of the trial, but were not initially considered necessary for Phase II, which was intended to provide safety follow up data until Subgam is marketed in

10.0 Study Schedule
Phase II (Page 19)

The following:
If the patient decides to withdraw from Phase II before the product is marketed, vital signs will be recorded and a sample for virology and an archive sample will be taken
Should be replaced by:
If the patient decides to withdraw from Phase II or the trial ends, vital signs will be recorded and an additional blood sample for virology, immunology and an archive sample will be taken.

Appendix 15 Study Procedures
Flowchart (Page 74)

The study procedures flow chart should now indicate that a blood sample will be collected at the end of Phase II for measurement of IgG.

Consent forms
Adult and Child

New consent form (CF incorporating amendment 3_29-Jan-2004) asking patients to consent to the collection of IgG data from their patient notes where possible, as well as having an additional blood sample taken at the end of the study.
To this, will be attached the original consent form (version3.april2000) should they wish to read it

Patient information leaflets
Adult, Child and parent/guardian

New patient information leaflet (PIL incorporating amendment 3_29-Jan-2004) informing patients about the collection of IgG data from their patient notes where possible, as well as having an additional blood sample taken at the end of the study.
To this, will be attached the original patient information leaflet (version3.april2000) should they wish to read it

CRF

An additional CRF page will be inserted for collection of IgG data. (Title: Phase II IgG blood results)

up data until Subgam is marketed in the UK. On reflection, we now consider that IgG data during Phase II would be useful to affirm that the doses of Subgam administered to the patients continue to maintain plasma IgG at acceptable levels after long term (1-3.5 years) use of Subgam.
IgG levels are generally monitored on a regular basis by the study physicians, as part of the patients standard care, and as such are recorded in the patient notes. We would like to collect this data, where available, for inclusion in our analysis. We would also like to collect a sample at the end of the study or when the patient withdraws from the study for analysis of plasma IgG levels to provide a final IgG level.

List of documents to be read

When Read (please initial)

Protocol Amendment 3-29th January 2004	C.P.T.
Consent form (Child) – CF incorporating amendment 3_29-Jan-2004	C.P.T.
Consent form (adult) – CF incorporating amendment 3_29-Jan-2004	C.P.T.
Patient information leaflet (adult) - PIL incorporating amendment 3_29-Jan-2004	C.P.T
Patient information leaflet (child) - PIL incorporating amendment 3_29-Jan-2004	C.P.T.
Patient information leaflet (parent/guardian) - PIL incorporating amendment 3_29-Jan-2004	C.P T.

I certify that I have read this amendment and that I have and have duly informed the staff involved in the trial of the change/s outlined.

Signed... Date....12/10/04.....

Name....DR C.P. TSAKONA.. Position......CONSULTANT...

Protocol Amendment Notification

Title of Trial A MULTI-CENTRE, OPEN STUDY TO ASSESS THE SAFETY AND EFFICACY OF A 16% IMMUNOGLOBULIN PRODUCT (SUBGAM ®) GIVEN VIA THE SUBCUTANEOUS ROUTE IN PRIMARY ANTIBODY DEFICIENT PATIENTS

Trial Code SCIG01

Version number amended Version3.April2000

Protocol Amendment Number 3

EUDRACT No Not Applicable

Investigator: Dr Vijayadurai

Centre: Royal Preston Hospital

Date issued to site:

D	D	M	M	M	Y	Y	Y	Y
1	3	M	A	Y	2	0	0	4

Section	Details of Amendment	Justification of Amendment
Laboratory Measurements Throughout Protocol	Addition<br>IgG levels of patients during Phase II shall now be collected from patient notes, where possible. An additional sample will be taken at the end of the study.	IgG levels were monitored throughout Phase I of the trial, but were not initially considered necessary for Phase II, which was intended to provide safety follow up data until Subgam is marketed in the UK. On reflection, we now consider that IgG data during Phase II would be useful to affirm that the doses of Subgam administered to the patients continue to maintain plasma IgG at acceptable levels after long term (1-3.5 years) use of Subgam.<br>IgG levels are generally monitored on a regular basis by the study physicians, as part of the patients standard care, and as such are recorded in the patient notes. We would like to collect this data, where available, for inclusion in our analysis. We would also like to collect a sample at the end of the study or when the patient withdraws from the study for analysis of plasma IgG levels to provide a final IgG level.

10.0 Study Schedule<br>Phase II (Page 19)	**The following:**<br>If the patient decides to withdraw from Phase II before the product is marketed, vital signs will be recorded and a sample for virology and an archive sample will be taken<br>**Should be replaced by:**<br>If the patient decides to withdraw from Phase II or the trial ends, vital signs will be recorded and an additional blood sample for virology, immunology and an archive sample will be taken.
Appendix 15 Study Procedures Flowchart (Page 74)	The study procedures flow chart should now indicate that a blood sample will be collected at the end of Phase II for measurement of IgG.
Consent forms<br>Adult and Child	New consent form (CF incorporating amendment 3_29-Jan-2004) asking patients to consent to the collection of IgG data from their patient notes where possible; as well as having an additional blood sample taken at the end of the study.<br>To this, will be attached the original consent form (version3.april2000) should they wish to read it
Patient information leaflets<br>Adult, Child and parent/guardian	New patient information leaflet (PIL incorporating amendment 3_29-Jan-2004) informing patients about the collection of IgG data from their patient notes where possible, as well as having an additional blood sample taken at the end of the study.<br>To this, will be attached the original patient information leaflet (version3.april2000) should they wish to read it
CRF	An additional CRF page will be inserted for collection of IgG data. (Title: Phase II IgG blood results)

List of documents to be read

	When Read (please initial)
Protocol Amendment 3-29th January 2004	DVL
Consent form (Child) – CF incorporating amendment 3_29-Jan-2004	PV
Consent form (adult) – CF incorporating amendment 3_29-Jan-2004	PV
Patient information leaflet (adult) - PIL incorporating amendment 3_29-Jan-2004	DVL
Patient information leaflet (child) - PIL incorporating amendment 3_29-Jan-2004	PV
Patient information leaflet (parent/guardian) - PIL incorporating amendment 3_29-Jan-2004	DVL

I certify that I have read this amendment and that I have and have duly informed the staff involved in the trial of the change/s outlined.

Signed: P Vijay [signature] Date: 2/F/04.

Name: P. VIJAYADURAI Position: CONSULTANT.

MD020303
Version 4

Protocol Amendment Notification

Title of Trial A MULTI-CENTRE, OPEN STUDY TO ASSESS THE SAFETY AND EFFICACY OF A 16% IMMUNOGLOBULIN PRODUCT (SUBGAM ®) GIVEN VIA THE SUBCUTANEOUS ROUTE IN PRIMARY ANTIBODY DEFICIENT PATIENTS

Trial Code SCIG01

Version number amended Version3.April2000

Protocol Amendment Number 3

EUDRACT No Not Applicable

Investigator Professor Warner

Centre Southampton General Hospital

Date issued to site:

D	D	M	M	M	Y	Y	Y	Y
1	[illegible]	0	4		2	0	0	4

Section	Details of Amendment	Justification of Amendment
Laboratory Measurements Throughout Protocol	Addition<br>IgG levels of patients during Phase II shall now be collected from patient notes, where possible. An additional sample will be taken at the end of the study.	IgG levels were monitored throughout Phase I of the trial, but were not initially considered necessary for Phase II, which was intended to provide safety follow up data until Subgam is marketed in the UK. On reflection, we now consider that IgG data during Phase II would be useful to affirm that the doses of Subgam administered to the patients continue to maintain plasma IgG at acceptable levels after long term (1-3.5 years) use of Subgam.<br>IgG levels are generally monitored on a regular basis by the study physicians, as part of the patients standard care, and as such are recorded in the patient notes. We would like to collect this data, where available, for inclusion in our analysis. We would also like to collect a sample at the end of the study or when the patient withdraws from the study for analysis of plasma IgG levels to provide a final IgG level.

10.0 Study Schedule<br>Phase II (Page 19)	**The following:**<br>If the patient decides to withdraw from Phase II before the product is marketed, vital signs will be recorded and a sample for virology and an archive sample will be taken<br>**Should be replaced by:**<br>If the patient decides to withdraw from Phase II or the trial ends, vital signs will be recorded and an additional blood sample for virology, immunology and an archive sample will be taken.
Appendix 15 Study Procedures Flowchart (Page 74)	The study procedures flow chart should now indicate that a blood sample will be collected at the end of Phase II for measurement of IgG.
Consent forms<br>Adult and Child	New consent form (CF incorporating amendment 3_29-Jan-2004) asking patients to consent to the collection of IgG data from their patient notes where possible, as well as having an additional blood sample taken at the end of the study.<br>To this, will be attached the original consent form (version3.april2000) should they wish to read it
Patient information leaflets<br>Adult, Child and parent/guardian	New patient information leaflet (PIL incorporating amendment 3_29-Jan-2004) informing patients about the collection of IgG data from their patient notes where possible, as well as having an additional blood sample taken at the end of the study.<br>To this, will be attached the original patient information leaflet (version3.april2000) should they wish to read it
CRF	An additional CRF page will be inserted for collection of IgG data. (Title: Phase II IgG blood results)

List of documents to be read

List of documents to be read	When Read (please initial)
Protocol Amendment 3-29th January 2004	
Consent form (Child) – CF incorporating amendment 3_29-Jan-2004	
Consent form (adult) – CF incorporating amendment 3_29-Jan-2004	
Patient information leaflet (adult) - PIL incorporating amendment 3_29-Jan-2004	
Patient information leaflet (child) - PIL incorporating amendment 3_29-Jan-2004	
Patient information leaflet (parent/guardian) - PIL incorporating amendment 3_29-Jan-2004	

I certify that I have read this amendment and that I have and have duly informed the staff involved in the trial of the change/s outlined.

Signed [signature] Date 27 . 4 . 0[illegible]

Name J D WIDDNER Position PROF OF CHILD HEALTH

Protocol Amendment Notification

Title of Trial A MULTI-CENTRE, OPEN STUDY TO ASSESS THE SAFETY AND EFFICACY OF A 16% IMMUNOGLOBULIN PRODUCT (SUBGAM ®) GIVEN VIA THE SUBCUTANEOUS ROUTE IN PRIMARY ANTIBODY DEFICIENT PATIENTS

Trial Code SCIG01

Version number amended Version3.April2000

Protocol Amendment Number 3

EUDRACT No Not Applicable

Investigator Dr Williams

Centre University Hospital of Wales, Cardiff

Date issued to site:

D	D	M	M	M	Y	Y	Y	Y
1	8	A	P	R	2	0	0	4

Section	Details of Amendment	Justification of Amendment
Laboratory Measurements Throughout Protocol	Addition<br>IgG levels of patients during Phase II shall now be collected from patient notes, where possible. An additional sample will be taken at the end of the study.	IgG levels were monitored throughout Phase I of the trial, but were not initially considered necessary for Phase II, which was intended to provide safety follow up data until Subgam is marketed in the UK. On reflection, we now consider that IgG data during Phase II would be useful to affirm that the doses of Subgam administered to the patients continue to maintain plasma IgG at acceptable levels after long term (1-3.5 years) use of Subgam.<br>IgG levels are generally monitored on a regular basis by the study physicians, as part of the patients standard care, and as such are recorded in the patient notes. We would like to collect this data, where available, for inclusion in our analysis. We would also like to collect a sample at the end of the study or when the patient withdraws from the study for analysis of plasma IgG levels to provide a final IgG level.

10.0 Study Schedule<br>Phase II (Page 19)	**The following:**<br>If the patient decides to withdraw from Phase II before the product is marketed, vital signs will be recorded and a sample for virology and an archive sample will be taken<br>**Should be replaced by:**<br>If the patient decides to withdraw from Phase II or the trial ends, vital signs will be recorded and an additional blood sample for virology, immunology and an archive sample will be taken.
Appendix 15 Study Procedures<br>Flowchart (Page 74)	The study procedures flow chart should now indicate that a blood sample will be collected at the end of Phase II for measurement of IgG.
Consent forms<br>Adult and Child	New consent form (CF incorporating amendment 3_29-Jan-2004) asking patients to consent to the collection of IgG data from their patient notes where possible, as well as having an additional blood sample taken at the end of the study.<br>To this, will be attached the original consent form (version3.april2000) should they wish to read it
Patient information leaflets<br>Adult, Child and parent/guardian	New patient information leaflet (PIL incorporating amendment 3_29-Jan-2004) informing patients about the collection of IgG data from their patient notes where possible, as well as having an additional blood sample taken at the end of the study.<br>To this, will be attached the original patient information leaflet (version3.april2000) should they wish to read it
CRF	An additional CRF page will be inserted for collection of IgG data. (Title: Phase II IgG blood results)

List of documents to be read — When Read (please initial)

List of documents to be read	When Read (please initial)
Protocol Amendment 3-29th January 2004	PEW
Consent form (Child) – CF incorporating amendment 3_29-Jan-2004	PEW
Consent form (adult) – CF incorporating amendment 3_29-Jan-2004	PEW
Patient information leaflet (adult) - PIL incorporating amendment 3_29-Jan-2004	PEW
Patient information leaflet (child) - PIL incorporating amendment 3_29-Jan-2004	PEW
Patient information leaflet (parent/guardian) - PIL incorporating amendment 3_29-Jan-2004	PEW

I certify that I have read this amendment and that I have and have duly informed the staff involved in the trial of the change/s outlined.

Signed: [signature] Date: 18 April 2004

Name: DR P E WILLIAMS Position: Consultant Clinical Immunologist
